# Identification of candidate chemosensory genes in *Bactrocera cucurbitae* based on antennal transcriptome analysis

**DOI:** 10.3389/fphys.2024.1354530

**Published:** 2024-02-19

**Authors:** Jing Jing Wang, Chao Ma, Yang Yue, Jingfang Yang, Li Xiang Chen, Yi Ting Wang, Chen Chen Zhao, Xuyuan Gao, Hong Song Chen, Wei Hua Ma, Zhongshi Zhou

**Affiliations:** ^1^ State Key Laboratory for Biology of Plant Diseases and Insect Pests, Institute of Plant Protection, Chinese Academy of Agricultural Sciences, Beijing, China; ^2^ National Nanfan Research Institute, Chinese Academy of Agricultural Sciences, Sanya, China; ^3^ Henan Agricultural University, Henan, China; ^4^ Guangxi Key Laboratory for Biology of Crop Diseases and Insect Pests, Institute of Plant Protection, Guangxi Academy of Agricultural Sciences, Nanning, China; ^5^ Hubei Insect Resources Utilization and Sustainable Pest Management Key Laboratory, College of Plant Science and Technology, Huazhong Agricultural University, Wuhan, China

**Keywords:** Bactrocera cucurbitae, chemosensory genes, transcriptome, insect olfaction, pest management

## Abstract

The melon fly, *Bactrocera cucurbitae* (Coquillett) (Tephritidae: Diptera), is an invasive pest that poses a significant threat to agriculture in Africa and other regions. Flies are known to use their olfactory systems to recognise environmental chemical cues. However, the molecular components of the chemosensory system of *B. cucurbitae* are poorly characterised. To address this knowledge gap, we have used next-generation sequencing to analyse the antenna transcriptomes of sexually immature *B. cucurbitae* adults. The results have identified 160 potential chemosensory genes, including 35 odourant-binding proteins (OBPs), one chemosensory protein (CSP), three sensory neuron membrane proteins (SNMPs), 70 odourant receptors (ORs), 30 ionotropic receptors (IRs), and 21 gustatory receptors (GRs). Quantitative real-time polymerase chain reaction quantitative polymerase chain reaction was used to validate the results by assessing the expression profiles of 25 ORs and 15 OBPs. Notably, high expression levels for *BcucOBP5/9/10/18/21/23/26* were observed in both the female and male antennae. Furthermore, *BcucOROrco/6/7/9/13/15/25/27/28/42/62* exhibited biased expression in the male antennae, whereas *BcucOR55* showed biased expression in the female antennae. This comprehensive investigation provides valuable insights into insect olfaction at the molecular level and will, thus, help to facilitate the development of enhanced pest management strategies in the future.

## Introduction

The olfactory system is essential for insect reproduction and survival (Song et al., 2020) and is involved in a wide variety of insect behaviours, including host location, identification of oviposition sites and mates, and avoidance of predators (Hansson and Stensmyr, 2011; Leal, 2013; Suh et al., 2014; Lin et al., 2015; Wang et al., 2016). Furthermore, the antennae, which house different types of sensilla (Steinbrecht, 1996; Shanbhag et al., 1999), serve as the primary organs for olfactory sensing and, thus, play a crucial role in a variety of different behaviours (Liu J. et al., 2021; Liu X. et al., 2021). Insect olfaction relies on the olfactory receptor neurones (ORNs) in the sensilla (Zhu et al., 2021). Odourant molecules reach the dendrites of ORNs through the sensillary lymph (Leitch et al., 2015; Hu et al., 2022), with the assistance of odourant-binding proteins (OBPs) and chemosensory proteins (Pelosi et al., 2014; Mei et al., 2018; Pelosi et al., 2018; Zhou et al., 2021). The OBP-odour complex is then transported to specific odourant receptor proteins (ORs) located on the dendritic membrane of ORNs (Christine et al., 2014; Andersson et al., 2013; Sun et al., 2020), triggering the conversion of chemical signals into electrical signals. These signals are eventually processed by the central nervous system (CNS), leading to appropriate insect behavioural responses (Wicher and Miazzi, 2021; Wu et al., 2022). Orco, a highly conserved olfactory co-receptor present in various insect species, plays a crucial role in OR localisation and odour signal reception (Fan et al., 2015; Li et al., 2016). During the odour-recognition process, ORs form tetramers with Orco, acting as channels for odour-gated ions in response to odourants and pheromones (Koutroumpa et al., 2016; Sun et al., 2019; Task et al., 2020). Other olfactory genes are involved in signal recognition and play a role in insect olfaction. Sensory neuron membrane proteins (SNMPs) belong to the CD36 protein family (Li et al., 2021) and are essential for sensory pheromone detection (Vogt et al., 2009; Cassau and Krieger, 2021). Ionotropic receptors (IRs), members of the ionotropic glutamate receptor (iGluR) gene family (Zhang et al., 2019), play a key role in odour identification, as well as in sensing acids, salts, aldehydes, ammonia, temperature, humidity, and taste (Chen et al., 2015; Enjin et al., 2016; Knecht et al., 2016; Frank et al., 2017). While gustatory receptors (GRs) are responsible for gustatory substance detection (Xing et al., 2021) and can be categorised as CO_2_, sugar, and bitter receptors (Kwon et al., 2007; Sato et al., 2011; Ning et al., 2016; Xu, 2020).

The melon fly, *Bactrocera cucurbitae* (Coquillett) (Diptera: Tephritidae) is native to India and is widely distributed in tropical and subtropical regions worldwide (Dhillon et al., 2005; Mir et al., 2014). Currently, *B. cucurbitae* control primarily relies on the use of insecticides. However, this approach is becoming increasingly ineffective due to the development of insecticide resistance and the negative impacts of pesticide residues on human health and the environment (Aktar et al., 2009; Curl et al., 2020; Shivaramu et al., 2022). To explore alternative control methods, it is crucial to understand the molecular mechanisms underlying fly behaviour. Female *B. cucurbitae* use host-plant volatiles as cues to locate suitable hosts (Vargas et al., 2018; Piñero et al., 2020; Jaime et al., 2021). Although the role of these volatiles in host-seeking behaviour has been documented, the molecular basis of chemoreception in *B. cucurbitae* remains poorly understood. The identification of olfaction-related genes, is thus required to enable the elucidation of the olfactory perception mechanism in this species.

In this investigation, transcriptomic analysis of the antennae from immature male and female adult *B. cucurbitae* was conducted. The analysis identified 160 candidate chemosensory genes, including 70 odourant receptors (ORs), 35 odourant-binding proteins (OBPs), one chemosensory protein (CSP), 30 ionotropic receptors (IRs), 21 gustatory receptors (GRs), and three sensory neuron membrane proteins (SNMPs). To further investigate the expression patterns of these genes, quantitative real-time polymerase chain reaction (qPCR) was performed for 15 OBPs and 25 ORs. The results provide a foundation for future functional studies on chemoreception in *B. cucurbitae*. Ultimately, understanding the molecular mechanisms involved in the fly olfactory system could contribute to the development of novel and sustainable approaches for pest control.

## Materials and methods

### Insect rearing

*B. cucurbitae* specimens were collected from rotting fruits in the fields surrounding cities in Guangxi province, China. They were then placed in rearing cages (30 × 30 × 30 cm) until they emerged as adults. These insects were maintained for over 20 generations under controlled conditions, including a temperature of 26°C ± 1°C, relative humidity of 70% ± 10%, and a photoperiod of 14 h light and 10 h dark (14L:10D). They were provided with water and a forage mixture consisting of yeast and sugar at a ratio of 1:2.

Newly emerged male and female adults that were sexually immature and between 4 and 6 days of age were selected for the experiments. The antennae of these individuals were carefully grasped by their roots using tweezers and transferred to Eppendorf tubes. Additionally, to study the gene expression profiles in the different tissues, samples of the male antennae (M-T), female antennae (F-T), head, thorax, abdomen, legs, and wings were also collected. All the collected samples were immediately frozen in liquid nitrogen and stored at −80°C until RNA extraction for further analysis.

### RNA extraction, cDNA library construction, and sequencing

RNA was extracted from the collected samples using a TRIzol reagent kit (Invitrogen), according to the manufacturer’s instructions. The RNA quality was assessed using an Agilent 2,100 Bioanalyzer and agarose gel electrophoresis. Eukaryotic mRNA was enriched using oligo (dT) beads, whereas prokaryotic mRNA was enriched by removing the rRNA using the Ribo-Zero™ Magnetic Kit (Epicentre). The enriched mRNA was fragmented and reverse transcribed into cDNA using random primers. Second-strand cDNA synthesis was performed, and the resulting fragments were purified and sequenced by ligating Illumina adapters. The cDNA libraries were sequenced using the Illumina Novaseq6000 platform (Gene Denovo Biotechnology Co., Guangzhou, China).

### Transcriptomic profiling

Clean reads were obtained using Perl scripts, as follows: (1) adapters were removed, (2) low-quality reads were removed (base numbers with a mass value Q ≤ 20 accounted for more than 50% of the whole read), (3) reads containing more than 10% N were removed, and (4) all A-base reads were removed. The Q20, Q30, GC content, and sequence duplication levels for the clean data were then determined. Genomic localisation analysis of the clean reads was mapped to *B. cucurbitae* using HISAT2 (Kim et al., 2015), and Stringtie (Pertea et al., 2015) was used to reconstruct the transcripts. RSEM (Li and Dewey, 2011) was used to calculate the expression levels of all genes in each sample.

### Sequence analysis and phylogenetic analysis

The open reading frames (ORFs) of the candidate chemosensory genes were identified using the NCBI ORF finder (https://www.ncbi.nlm.nih.gov/orffinder/), and similarity searchers performed using the National Center for NCBI-BLAST server. The TMHMM Server Version 2.0 (http://www.cbs.dtu.dk/services/TMHMM) was used to predict TMDs, and Signal IP4.1 (http://www.cbs.dtu.dk/services/SignalP/) was used to predict signal peptide sequences. Amino acid sequence alignment was performed using the ClustalW method in MEGA v6.0. The OBP dataset contained 52 sequences from *Drosophila melanogaster*, 34 from *Bactrocera correcta*, 43 from *Bactrocera dorsalis*, 33 from *Bactrocera latifrons*, 25 from *Zeugodacus tau*, 26 from *Anastrepha ludens*, 33 from *Rhagoletis pomonella*, 28 from *Calliphora stygia*, and 35 from *Ceratitis capitata*. The OR dataset contained 37 sequences from *D. melanogaster*, 38 from *B. correcta*, 36 from *B. dorsalis*, 40 from *B. latifrons*, 46 from *Bactrocera minax,* 39 from *Z. tau*, 26 from *A. ludens*, 53 from *R. pomonella*, 50 from *C. stygia*, and 42 from *C. capitata*. The GR dataset contained 39 sequences from *D. melanogaster*, 6 from *B. correcta*, 18 from *B. dorsalis*, 18 from *B. latifrons*, 4 from *Z. tau*, 10 from *A. ludens*, 14 from *R. pomonella*, 4 from *C. stygia*, and 12 from *C. capitata*. The IR dataset contained 26 sequences from *D. melanogaster*, 9 from *B. correcta*, 11 from *B. dorsalis*, 10 from *B. latifrons*, 11 from *Z. tau*, 7 from *A. ludens*, 9 from *R. pomonella*, 7 from *C. stygia*, and 10 from *C. capitata*. The CSP dataset contained two sequences from *D. melanogaster*, 1 from *B. correcta*, 2 from *B. dorsalis*, 1 from *B. latifrons*, 1 from *Z. tau*, 1 from *A. ludens*, 1 from *R. zephyria*, 1 from *R. pomonella*, 1 from *C. stygia*, 1 from *C. capitata,* 1 from *Procecidochares utilis*, 1 from *Lucilia sericata*, 1 from *Lucilia cuprina*, and 1 from *Bactrocera oleae*. The SNMP dataset contained 2 sequences from *D. melanogaster*, 3 from *B. correcta*, 3 from *B. dorsalis*, 2 from *B. latifrons*, 3 from *Z. tau*, 1 from *A. ludens*, 2 from *R. zephyria*, 2 from *R. pomonella*, 3 from *C. stygia*, 2 from *C. capitata*, 2 from *L. sericata*, 2 from *L. cuprina*, 2 from *B. oleae*, and 2 from *Bactrocera neohumeralis.*

Phylogenetic trees were constructed using the neighbour-joining method and p-distance modelling, and the trees were adjusted using FigTree v1.4.4. The candidate chemosensory genes were named “*BcucOBP*”, “*BcucCSP*”, “*BcucSNMP*”, “*BcucOR*”, “*BcucGR*”, and “*BcucIR*”, followed by a numeral indicating the coding region length. All amino acid sequences from the *B. cucurbitae* and other insects used in the phylogenetic analyses are listed in Supplementary Material S3.

### Quantitative real-time PCR validation

For expression verification, 25 ORs and 15 OBPs were selected based on their high abundance levels from fragments per kilobase of exon per million reads mapped (FPKM), as measured using (Supplementary Material S4). Total RNA was isolated from each tissue and normalised to a concentration of 1 μg/μL. First-strand cDNA synthesis was performed using a commercial kit (Transgen Biotech, Beijing, China). A qPCR assay was conducted using a 20 μL master mix containing TransStart^®^ TipGreen qPCR SuperMix, cDNA templates, primers, and water. Real-time quantitative PCR was performed using an ABI 7500 instrument (Applied Biosystems). The PCR program consisted of cycles of denaturation, annealing, extension, and amplification (Transgen Biotech, Beijing, China). RPL32 was used as an internal reference for normalisation. Primers were designed using Primer Premier 5.0, and their efficiencies were verified. Each experiment included three biological and three technical replicates. The expression levels were quantified using the 2^−ΔΔCT^ method, with RPL32 as the reference gene (Livak et al., 2001).

### Statistical analysis

Data analysis was performed using the 2^−ΔΔCT^ method, and data were analysed using SAS 9.0 (SAS Institute Inc., Cary, NC, USA). Statistical significance was analysed using ANOVA followed by Tukey’s multiple comparison test. Statistical significance was set at p < 0.05. All figures were generated using OriginPro 9.1 (Northampton, Massachusetts, USA).

## Results

### Transcriptome overview

The sequencing data generated for all samples in this study ranged from 37 to 42 million clean reads. Overall, the transcriptomic data were highly valid, with a minimum sample accuracy of 97%. The Q30 score, which represents a 0.1% base error rate, ranged from 92.95% to 100% (Supplementary Material S1). The Q20 score, which indicates a 1% base error rate, ranged from 97.23% to 100%. The GC content of the reads was determined to be 41.23%. The quality control measures ensured that all samples met the necessary criteria for the subsequent analyses.

After mapping the reads to the reference genome, an average total mapped value of approximately 87% was obtained for all samples (Supplementary Material S2). This indicated that a substantial proportion of the reads were successfully aligned to known genes. In total, 13,473 genes were identified from the sequencing data. These included 12,984 reference and 489 novel genes. The presence of novel genes suggests that previously uncharacterised genetic elements could be discovered in the melon fly transcriptome.

Furthermore, the high Pearson correlation coefficient values (R^2^ ≥ 0.97) observed for the three biological replicates for the male and female antennae indicated strong reproducibility between samples. This confirms the reliability of the experimental setup and strengthens confidence in the subsequent analysis and data interpretation.

### Characterisation of odourant binding proteins

**FIGURE 1:**
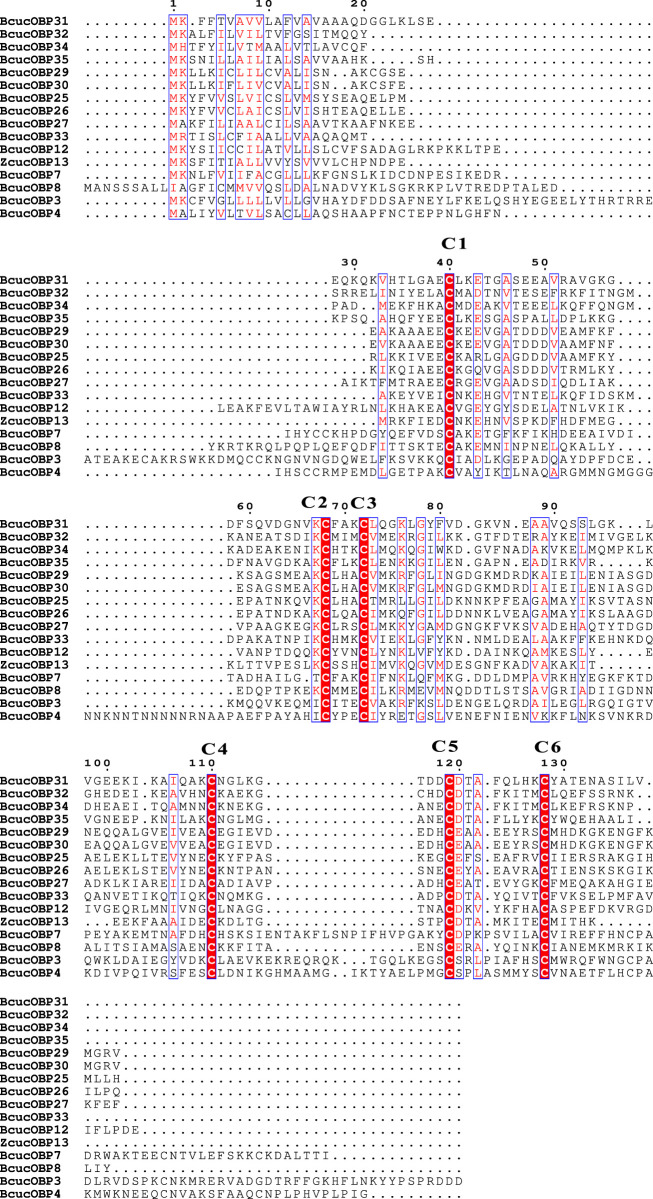
Multiple alignment of the OBPs identified in *Bactrocera cucurbitae*.

In this study, 35 candidate odourant binding protein (OBP) transcripts were identified in *B. cucurbitae*. These OBPs ranged in length from 130 to 313 amino acids. Sequence analysis revealed that 33 of these unigenes had putative full-length ORFs, 32 of which were predicted to contain a signal peptide. OBPs are highly conserved when compared to odourant receptors (ORs). The sequence alignment results demonstrated the presence of 16 classical OBPs with six conserved cysteines. These included *BcucOBP3, BcucOBP4, BcucOBP7, BcucOBP8, BcucOBP12, BcucOBP13, BcucOBP25, BcucOBP26, BcucOBP27, BcucOBP29, BcucOBP30, BcucOBP31, BcucOBP32, BcucOBP33, BcucOBP34,* and *BcucOBP35* (Figure 1).

**FIGURE 2:**
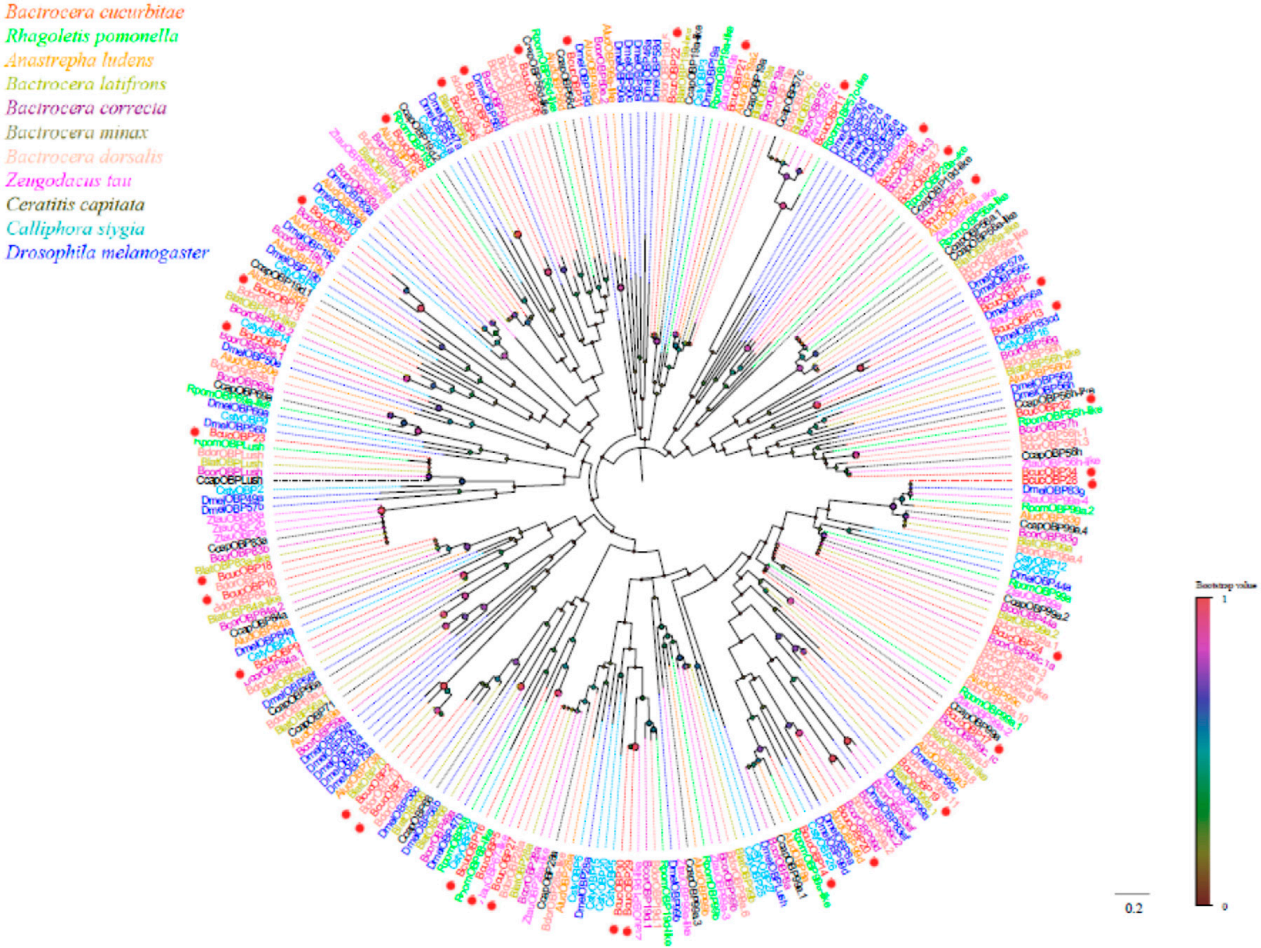
Neighbor joining phylogenetic tree of the candidate *BcucOBPs* with known Diptera OBP sequences.

Phylogenetic analysis revealed that the *B. cucurbitae* OBPs formed distinct branches and shared homology with OBPs from other Diptera species. These branch groupings were supported by high bootstrap values, indicating robustness. Notably, *BcucOBP23* was clustered with other Dipteran Lush OBPs, whereas *BcucOBP27* formed a clade with OBP28a. Furthermore, *BcucOBP9* and *BcucOBP10* were clustered within the OBP84a branch, and *BcucOBP13/32/34* were grouped in the OBP56 h branch. These specific groupings suggest the potential functional significance of OBPs in *B. cucurbitae* (Figure 2). Information including the unigene reference, length, and best Blastx hit for all 35 OBPs is listed in Table 1.

**TABLE 1:** The Blastx match of the Bactrocera cucurbitae candidate odorant binding proteins.

Unigene ID	NAME	Length	ORF (aa)	Status	Signal peptide	Blastx best-hit	Species	E-value	Accession
ncbi_105211198	BcucOBP1	1101	313	Complete ORF	Y	odorant-binding protein	Bactrocera cucurbitae	8e-176	QKN21211.1
ncbi_105213021	BcucOBP2	1223	263	Complete ORF	N	general odorant-binding protein 70 isoform X3	Bactrocera cucurbitae	0	XP_011183827.1
ncbi_105216511	BcucOBP3	924	251	Complete ORF	Y	odorant-binding protein	Bactrocera cucurbitae	2e-160	QKN21208.1
ncbi_105221282	BcucOBP4	887	223	Complete ORF	Y	odorant-binding protein	Bactrocera cucurbitae	1e-137	QKN21209.1
ncbi_105216980	BcucOBP5	770	216	Complete ORF	Y	odorant-binding protein	Bactrocera cucurbitae	2e-99	QKN21207.1
ncbi_105215210	BcucOBP6	882	210	Complete ORF	Y	general odorant-binding protein 56a	Bactrocera cucurbitae	6e-162	XP_011187314.1
ncbi_105209256	BcucOBP7	670	201	Complete ORF	Y	general odorant-binding protein 66	Bactrocera cucurbitae	2e-145	XP_011177871.1
ncbi_105215142	BcucOBP8	783	184	Complete ORF	Y	general odorant-binding protein 19d	Bactrocera cucurbitae	9e-131	XP_011187219.1
ncbi_105213577	BcucOBP9	710	177	Complete ORF	Y	general odorant-binding protein 84a	Bactrocera cucurbitae	6e-129	XP_011184797.1
ncbi_105213584	BcucOBP10	1053	173	5ʹ 3ʹ missing	Y	general odorant-binding protein 84a	Bactrocera cucurbitae	2e-106	XP_011184805.1
ncbi_105213953	BcucOBP11	981	172	Complete ORF	N	general odorant-binding protein 57c-like	Bactrocera cucurbitae	1e-85	XP_011185378.1
ncbi_105211201	BcucOBP12	678	165	Complete ORF	Y	general odorant-binding protein 56a	Bactrocera cucurbitae	8e-117	XP_011180838.1
ncbi_105211192	BcucOBP13	546	163	Complete ORF	Y	odorant-binding protein 56h	Bactrocera cucurbitae	2e-83	XP_011180830.1
ncbi_105213514	BcucOBP14	667	159	Complete ORF	Y	general odorant-binding protein 99a-like	Bactrocera cucurbitae	2e-115	XP_011184701.1
ncbi_105215156	BcucOBP15	717	158	Complete ORF	Y	general odorant-binding protein19d-like isoform X1	Bactrocera cucurbitae	3e-111	XP_011187239.1
ncbi_105217004	BcucOBP16	465	154	5′missing	N	general odorant-binding protein 67-like	Bactrocera cucurbitae	1e-99	XP_011190103.1
ncbi_105213511	BcucOBP17	636	149	Complete ORF	Y	general odorant-binding protein 99a-like	Bactrocera cucurbitae	5e-93	XP_011184698.1
ncbi_105218029	BcucOBP18	528	149	Complete ORF	Y	general odorant-binding protein 83a-like isoform X2	Bactrocera cucurbitae	5e-105	XP_028901212.1
ncbi_105209141	BcucOBP19	596	148	Complete ORF	Y	general odorant-binding protein 99a-like	Bactrocera cucurbitae	2e-105	XP_011177680.1
ncbi_105213513	BcucOBP20	590	148	Complete ORF	Y	odorant-binding protein	Bactrocera cucurbitae	8e-90	QKN21228.1
ncbi_105215137	BcucOBP21	637	148	Complete ORF	Y	general odorant-binding protein 19a	Bactrocera cucurbitae	6e-92	XP_011187213.1
ncbi_105215136	BcucOBP22	850	147	Complete ORF	Y	general odorant-binding protein 19a	Bactrocera cucurbitae	1e-101	XP_011187212.1
ncbi_105208502	BcucOBP23	669	145	Complete ORF	Y	general odorant-binding protein lush isoform X1	Bactrocera cucurbitae	2e-101	XP_011176687.1
ncbi_105212568	BcucOBP24	1025	143	Complete ORF	Y	odorant-binding protein 99a	Bactrocera cucurbitae	8e-83	XP_011182910.1
ncbi_105215159	BcucOBP25	589	143	Complete ORF	Y	general odorant-binding protein 19d	Bactrocera cucurbitae	1e-99	XP_011187242.1
ncbi_105215161	BcucOBP26	588	143	Complete ORF	Y	general odorant-binding protein 19d	Bactrocera cucurbitae	6e-98	XP_011187246.1
ncbi_105218953	BcucOBP27	706	143	Complete ORF	Y	odorant-binding protein 28a	Bactrocera cucurbitae	7e-89	XP_011193147.1
ncbi_105217972	BcucOBP28	818	142	Complete ORF	Y	general odorant-binding protein 99a	Bactrocera cucurbitae	7e-90	XP_011191566.1
ncbi_105215158	BcucOBP29	700	141	Complete ORF	Y	general odorant-binding protein 19d	Bactrocera cucurbitae	6e-74	XP_011187241.1
ncbi_105215160	BcucOBP30	694	141	Complete ORF	Y	general odorant-binding protein 19d	Bactrocera cucurbitae	4e-74	XP_011187244.1
ncbi_105211197	BcucOBP31	649	138	Complete ORF	Y	general odorant-binding protein 56d-like	Bactrocera cucurbitae	2e-77	XP_011180835.1
ncbi_105211263	BcucOBP32	447	137	Complete ORF	Y	general odorant-binding protein 56h-like	Bactrocera cucurbitae	6e-95	XP_011180911.1
ncbi_105211191	BcucOBP33	462	135	Complete ORF	Y	general odorant-binding protein 56d-like	Bactrocera cucurbitae	3e-94	XP_011180828.1
ncbi_105211193	BcucOBP34	619	135	Complete ORF	Y	general odorant-binding protein 56h-like	Bactrocera cucurbitae	9e-93	XP_011180831.1
ncbi_105211200	BcucOBP35	446	130	Complete ORF	Y	general odorant-binding protein 56d-like	Bactrocera cucurbitae	3e-77	XP_011180837.1

Conserved residues are highlighted using white letters with a red background, alignment positions are framed in a blue box if the corresponding residues are identical or similar, and six conserved cysteine residues are labelled with red stars.

Rpom, *Rhagoletis pomonella* (N = 29); Alud, Anastrepha ludens (N = 22); Blat, Bactrocera latifrons (N = 32); Bcor, Bactrocera correcta (N = 34); Bdor, Bactrocera dorsalis (N = 42); Ztau, Zeugodacus tau (N = 24); Ccap, Ceratitis capitata (N = 35); Csty, Calliphora stygia (N = 19); Dmel, *Drosophila melanogaster* (N = 52). The candidate BcucOBPs are indicated by red circles.

**FIGURE 3:**
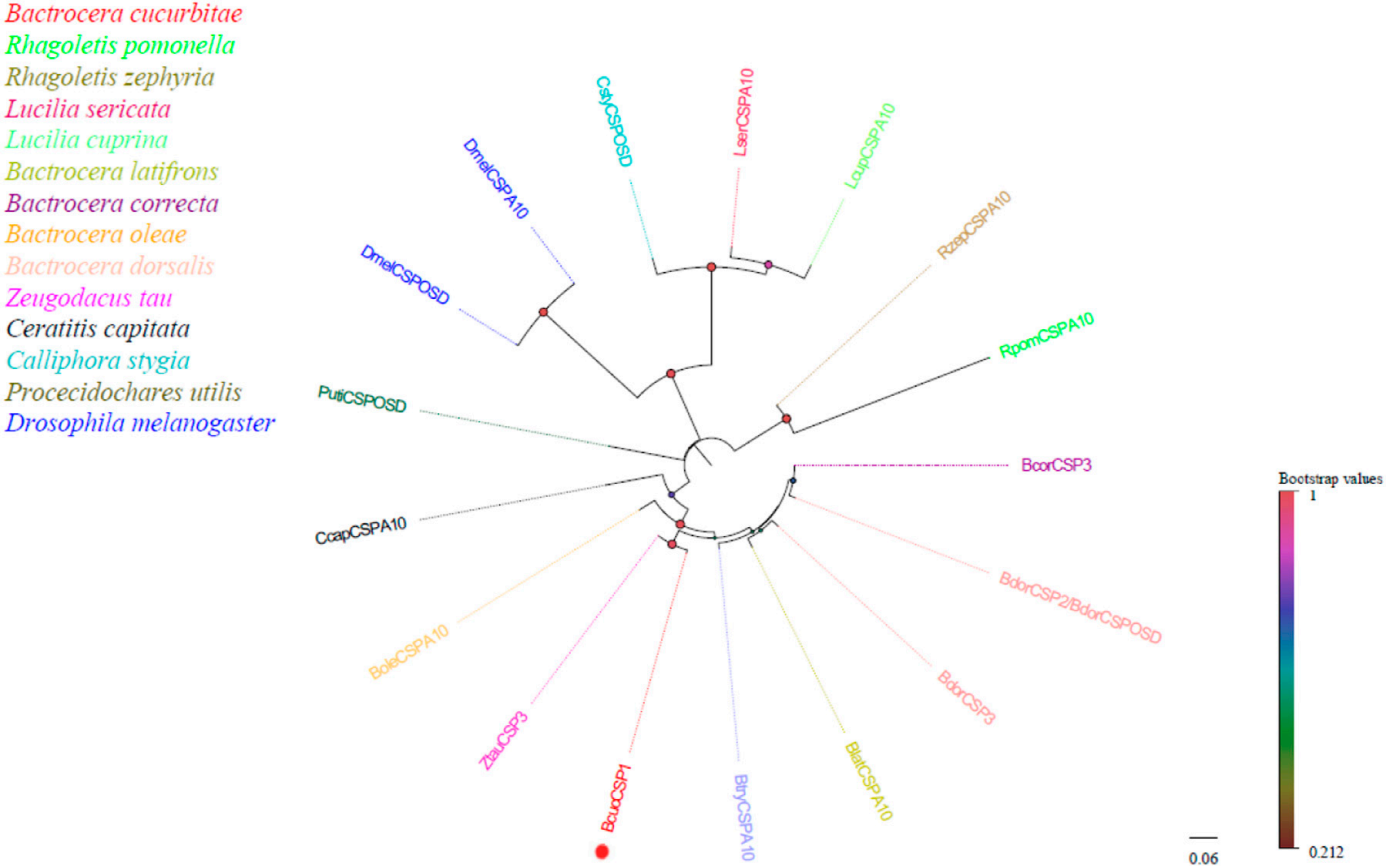
Neighbor joining phylogenetic tree of candidate *BcucCSP* with known Diptera CSP sequences. Rpom, *Rha*goletis pomonella (N = 1); Rzep, Rhagoletis zephyria (N = 1); Lser, *Lucilia sericata* (N = 1); Lcup, *Lucilia cuprina* (N = 1); Blat, Bactrocera latifrons (N = 1); Bcor, Bactrocera correcta (N = 1); Bole, Bactrocera oleae (N = 1); Bdor, Bactrocera dorsalis (N = 1); Ztau, Zeugodacus tau (N = 1); Ccap, Ceratitis capitata (N = 1); Csty, Calliphora stygia (N = 1); Puti, Procecidochares utilis (N = 1); Dmel, *Drosophila melanogaster* (N = 2). Candidate BcucCSP is indicated by red circles.

In this study, a candidate chemosensory protein (CSP) transcript was identified for *B. cucurbitae*, which was 2,263 bp in length and encoded 573 amino acids. Furthermore, it was found to exhibit a characteristic protein domain (A10/OSD) typically found in the CSP family. Homology analysis revealed that all *B. cucurbitae* CSPs were clustered with CSPs from other Dipteran species (Figure 3). This clustering indicates their evolutionary relationships and suggests functional similarities among these CSPs. For detailed information on the identified CSPs, including the unigene reference, length, and best Blastx hit, please refer to Supplementary Material S5.

**FIGURE 4:**
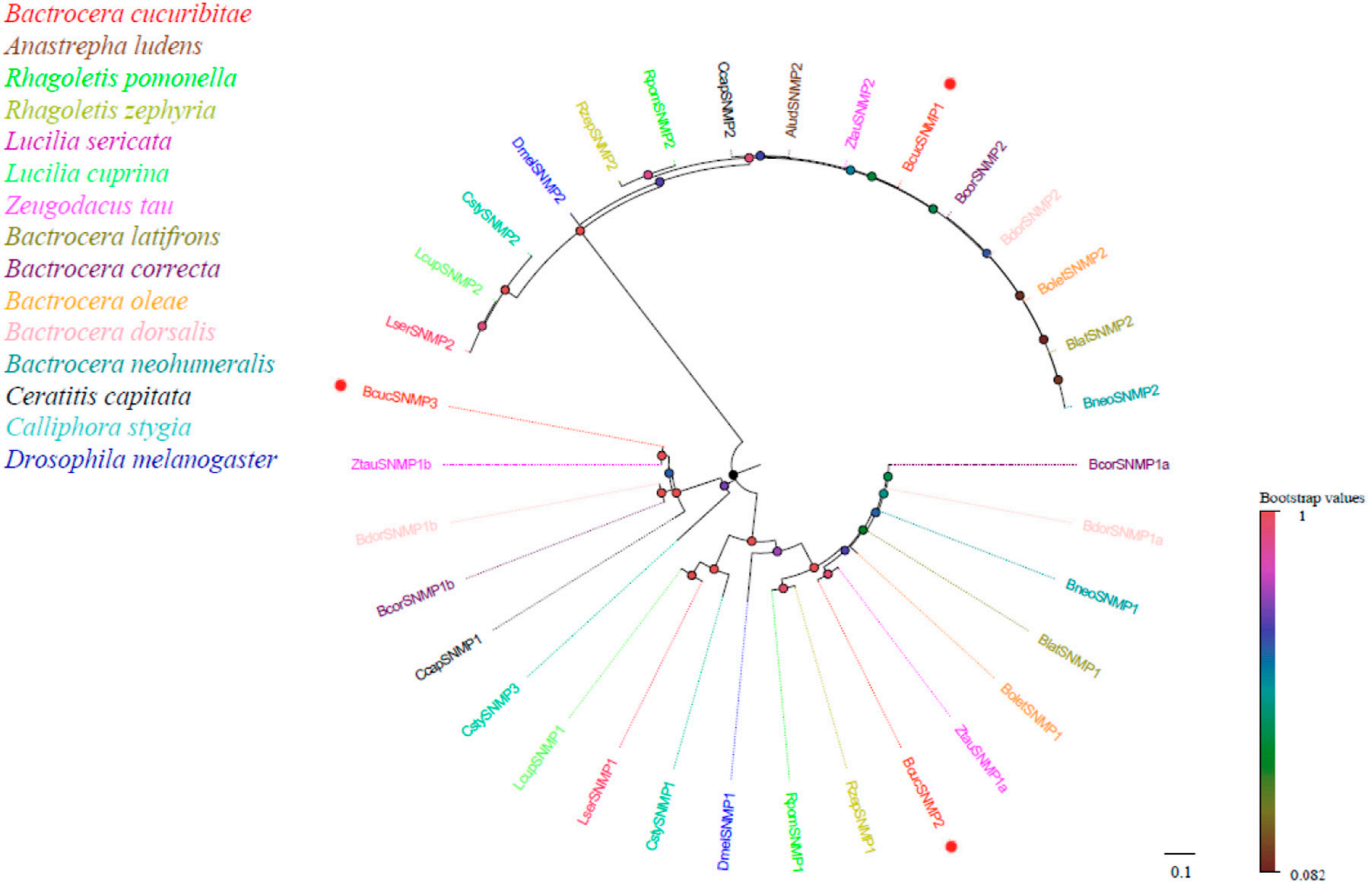
Neighbor joining phylogenetic tree of candidate BcucSNMPs with known Diptera SNMP sequences.

Four candidate sensory neuron membrane protein (SNMP) transcripts were identified in the antennal transcriptome of B. cucurbitae. These SNMP transcripts varied in length, ranging from 1777 to 2,263 bp, and encoded proteins consisting of 496–573 amino acids. Conserved domain annotation indicated that these SNMPs belonged to the CD36 family, which is consistent with their known functional role, and phylogenetic analysis revealed that all B. cucurbitae SNMPs were clustered together with the SNMPs from other Diptera species (Figure 4). This clustering indicates evolutionary relatedness and suggests similar functions across these species. The SNMPs can be further classified into two major groups: SNMP1 and SNMP2. Within SNMP1, two subgroups have been identified: SNMP1a and SNMP1b. This classification provides a high level of insight into the potential functional diversity of the SNMP family. Information, including the unigene reference, length, and best Blastx hits for all SNMPs, is listed in Supplementary Material S5.

Alud, Anastrepha ludens (N = 1); Rpom, *Rhagoletis pomonella* (N = 2); Rzep, Rhagoletis zephyria (N = 2); Lser, *Lucilia sericata* (N = 2); Lcup, *Lucilia cuprina* (N = 2); Ztau, Zeugodacus tau (N = 3); Blat, Bactrocera latifrons (N = 2); Bcor, Bactrocera correcta (N = 3); Bole, Bactrocera oleae (N = 2); Bdor, Bactrocera dorsalis (N = 3); Bneo, Bactrocera neohumeralis (N = 2); Ccap, Ceratitis capitata (N = 2); Csty, Calliphora stygia (N = 3); Dmel, *Drosophila melanogaster* (N = 2). The candidate BcucSNMPs are indicated by red circles.

**FIGURE 5:**
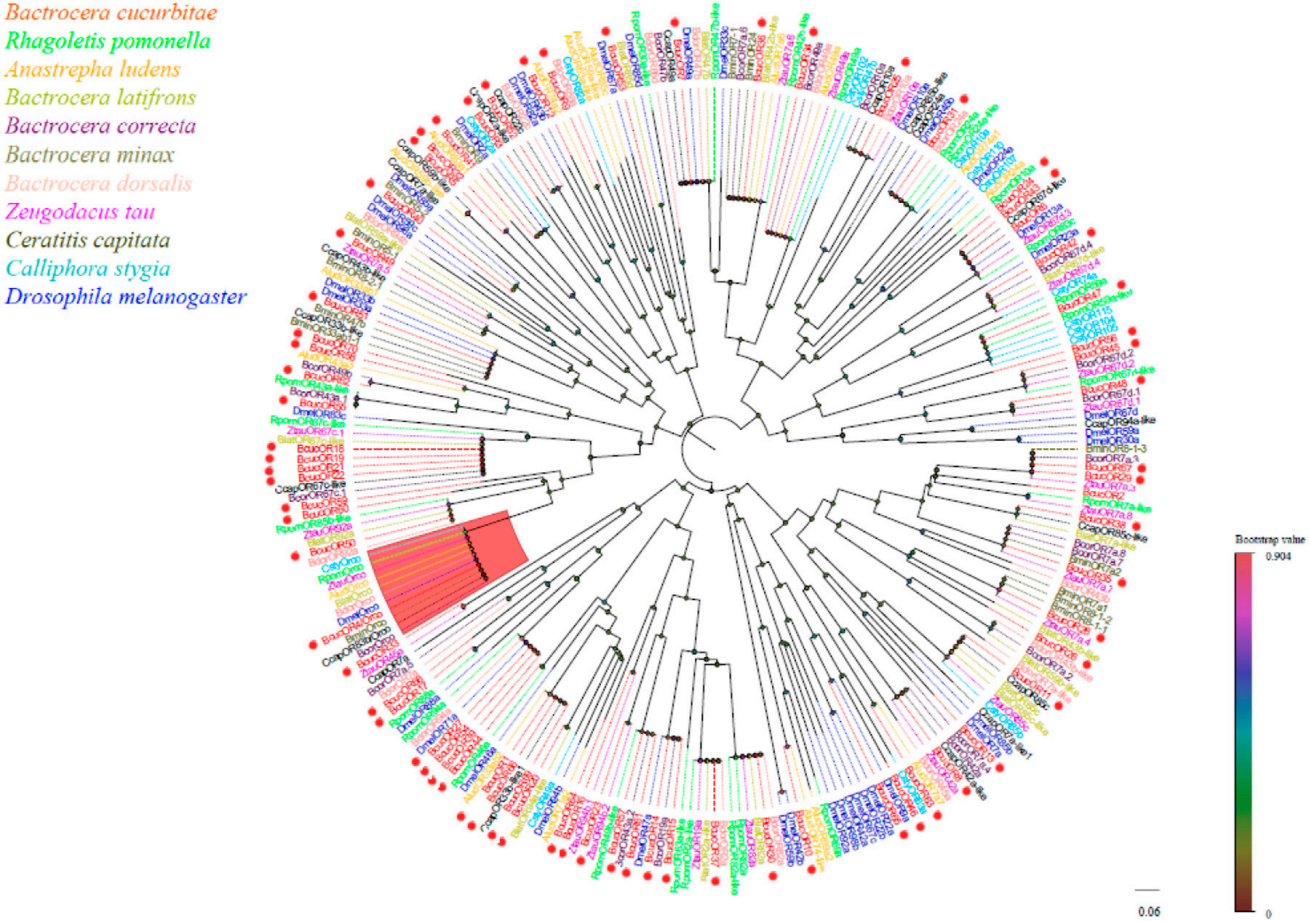
Neighbour joining phylogenetic tree for the candidate BcucORs with known Diptera OR sequences. Rpom, *Rhagoletis pomonella* (N = 26); Alud, Anastrepha ludens (N = 18); Blat, Bactrocera latifrons (N = 16); Bcor, Bactrocera correcta (N = 34); Bmin, Bactrocera minax (N = 14); Bdor, Bactrocera dorsalis (N = 18); Ztau, Zeugodacus tau (N = 22); Ccap, Ceratitis capitata (N = 20); Csty, Calliphora stygia (N = 18); Dmel, *Drosophila melanogaster* (N = 37). The candidate BcucORs are indicated by red circles.

Seventy OR transcripts were identified in the antennal transcriptome of B. cucurbitae. Among these, 61 putative ORs were predicted to have a full-length ORFs ranging from 233 to 757 amino acids. These ORs have two-eight transmembrane domains which are characteristic of odourant receptors. Phylogenetic analysis revealed that BcucORs display a high degree of differentiation, with most clustering into different clades (Figure 5). These clades were well supported by high bootstrap values, indicating a robust grouping. Notably, the Orco branch exhibited relatively high conservation as it possessed a complete ORF and seven transmembrane domains, consistent with the typical characteristics of ORs. BcucOrco, the orthologue of Orco in other insects, clustered with Orco from various species, such as ZtauOrco, BlatOrco, BcorOrco, BminOrco, BdorOrco, CcapOrco, CstyOrco, RpomOrco, AludOrco, and DmelOrco. Furthermore, a specific increase in the number of ORs has been observed in B. cucurbitae. These expansions were evident in branches, such as BcucOR2/8/29/35/38/67, BcucOR45, and BcucOR48, suggesting potential functional diversification within these specific receptor groups. Additional information, including the unigene reference, length, and best Blastx hits for all identified ORs, is shown in Table 2.

**TABLE 2:** The Blastx match of the *Bactrocera cucurbitae* candidate odorant receptors.

Unigene ID	NAME	Length	ORF (aa)	Status	TMD	Blastx best-hit	Species	E-value	Accession
ncbi_105209795	BcucOR1	2160	753	Complete ORF	11	odorant receptor 33b-like	Bactrocera cucurbitae	0	XP_028894539.1
ncbi_105211893	BcucOR2	2256	717	Complete ORF	7	odorant receptor 7a	Bactrocera cucurbitae	0	XP_011181871.2
ncbi_105211501	BcucOR3	1719	572	Complete ORF	11	odorant receptor 45a-like	Bactrocera cucurbitae	0	XP_028895522.1
ncbi_105213127	BcucOR4(ORco)	1950	473	Complete ORF	6	odorant receptor Orco	Bactrocera cucurbitae	0	XP_011183998.1
ncbi_105213189	BcucOR5	1494	470	Complete ORF	6	odorant receptor 83a-like	Bactrocera cucurbitae	0	XP_011184142.1
ncbi_105208959	BcucOR6	1504	449	Complete ORF	6	odorant receptor 13a	Bactrocera cucurbitae	0	XP_011177369.1
ncbi_105221416	BcucOR7	1636	443	Complete ORF	7	odorant receptor 47b isoform X1	Bactrocera cucurbitae	0	XP_028902114.1
ncbi_105212743	BcucOR8	1455	442	Complete ORF	6	odorant receptor 42a-like	Bactrocera cucurbitae	0	XP_011183261.1
ncbi_105210773	BcucOR9	1731	439	3′missing	4	odorant receptor 74a-likeisoform X1	Bactrocera cucurbitae	0	XP_028895116.1
ncbi_105217681	BcucOR10	1619	423	Complete ORF	6	odorant receptor	Bactrocera cucurbitae	0	QKN21155.1
ncbi_105218549	BcucOR11	1570	417	Complete ORF	5	odorant receptor 85c	Bactrocera cucurbitae	0	XP_028900206.1
ncbi_105213947	BcucOR12	1378	416	Complete ORF	6	odorant receptor 13a-like	Bactrocera cucurbitae	0	XP_011185366.1
ncbi_105220945	BcucOR13	1353	415	Complete ORF	7	odorant receptor 63a-like	Bactrocera cucurbitae	0	XP_011195820.2
ncbi_105220946	BcucOR14	1248	415	Complete ORF	7	odorant receptor 63a-like	Bactrocera cucurbitae	0	XP_028901880.1
ncbi_114803926	BcucOR15	1350	415	Complete ORF	6	odorant receptor 63a-like	Bactrocera cucurbitae	0	XP_028895659.1
ncbi_114803654	BcucOR16	1330	414	Complete ORF	6	odorant receptor 94b-like	Bactrocera cucurbitae	0	XP_028894386.1
ncbi_105212630	BcucOR17	1372	407	Complete ORF	4	odorant receptor	Bactrocera cucurbitae	0	QKN21159.1
ncbi_105217632	BcucOR18	1398	406	Complete ORF	6	odorant receptor 67c-like	Bactrocera cucurbitae	0	XP_011191024.1
ncbi_105217623	BcucOR19	1367	405	Complete ORF	7	odorant receptor 67c-like	Bactrocera cucurbitae	0	XP_028899203.1
ncbi_114803650	BcucOR20	1218	405	Complete ORF	8	odorant receptor 94a-like	Bactrocera cucurbitae	0	XP_028894353.1
ncbi_114804556	BcucOR21	1362	405	Complete ORF	6	odorant receptor 67c-like	Bactrocera cucurbitae	0	XP_028899204.1
ncbi_105210660	BcucOR22	1251	404	Complete ORF	6	odorant receptor 67c	Bactrocera cucurbitae	0	XP_011180049.1
ncbi_105210453	BcucOR23	1883	403	Complete ORF	6	odorant receptor 94a	Bactrocera cucurbitae	0	XP_011179733.1
ncbi_105212913	BcucOR24	1227	402	5ʹ missing	5	odorant receptor 74a	Bactrocera cucurbitae	0	XP_011183574.2
ncbi_105213489	BcucOR25	1602	402	Complete ORF	7	odorant receptor 10a	Bactrocera cucurbitae	0	XP_028897005.1
ncbi_105210232	BcucOR26	1399	400	Complete ORF	5	odorant receptor 42b-like	Bactrocera cucurbitae	0	XP_028894798.1
ncbi_105215540	BcucOR27	1364	400	Complete ORF	4	odorant receptor 43b-like isoform X2	Bactrocera cucurbitae	0	XP_028898283.1
ncbi_114805259	BcucOR28	1554	400	Complete ORF	6	odorant receptor 43b-like	Bactrocera cucurbitae	0	XP_028901710.1
ncbi_105209393	BcucOR29	1515	399	Complete ORF	6	odorant receptor 85a-like	Bactrocera cucurbitae	0	XP_028894284.1
ncbi_105211957	BcucOR30	1784	398	Complete ORF	6	odorant receptor 82a	Bactrocera cucurbitae	0	XP_011181975.1
ncbi_105217276	BcucOR31	1197	398	Complete ORF	8	odorant receptor 24a	Bactrocera cucurbitae	0	XP_011190505.1
ncbi_105213190	BcucOR32	1191	396	Complete ORF	5	odorant receptor 83a-like	Bactrocera cucurbitae	0	XP_028896796.1
ncbi_105211500	BcucOR33	1338	394	Complete ORF	6	odorant receptor 45a-like	Bactrocera cucurbitae	0	XP_011181253.2
ncbi_105211514	BcucOR34	1462	394	Complete ORF	6	odorant receptor 49a-like	Bactrocera cucurbitae	0	XP_011181266.1
ncbi_105212018	BcucOR35	1303	394	Complete ORF	5	odorant receptor 7a-like	Bactrocera cucurbitae	0	XP_011182064.1
ncbi_105213568	BcucOR36	1675	394	Complete ORF	5	odorant receptor 42b-like	Bactrocera cucurbitae	0	XP_011184786.1
ncbi_105215418	BcucOR37	1646	393	Complete ORF	5	odorant receptor 2a-like	Bactrocera cucurbitae	0	XP_011187627.2
ncbi_105216468	BcucOR38	2363	392	Complete ORF	6	odorant receptor 7a-like	Bactrocera cucurbitae	0	XP_011189274.1
ncbi_114803660	BcucOR39	1179	392	Complete ORF	7	odorant receptor 94a	Bactrocera cucurbitae	0	XP_028894417.1
ncbi_114803842	BcucOR40	1481	390	Complete ORF	7	odorant receptor 59a-like	Bactrocera cucurbitae	0	XP_028895352.1
ncbi_105210903	BcucOR41	1170	389	Complete ORF	6	odorant receptor 94a-like	Bactrocera cucurbitae	0	XP_011180402.1
ncbi_105214898	BcucOR42	1381	388	Complete ORF	6	odorant receptor 67d-like	Bactrocera cucurbitae	0	XP_011186895.2
ncbi_105214906	BcucOR43	1391	388	Complete ORF	6	odorant receptor 67d-like isoform X1	Bactrocera cucurbitae	0	XP_011186909.1
ncbi_105219152	BcucOR44	1536	388	Complete ORF	8	odorant receptor 46a isoform X1	Bactrocera cucurbitae	0	XP_028900650.1
ncbi_114804387	BcucOR45	1495	388	Complete ORF	6	odorant receptor 67d-like	Bactrocera cucurbitae	0	XP_028898362.1
ncbi_105209392	BcucOR46	1254	387	Complete ORF	7	odorant receptor 2a-like, partial	Bactrocera cucurbitae	0	XP_011178078.2
ncbi_105216084	BcucOR47	1317	387	Complete ORF	5	odorant receptor 59a-like	Bactrocera cucurbitae	0	XP_011188666.1
ncbi_114804388	BcucOR48	1304	386	Complete ORF	6	odorant receptor 67d-like	Bactrocera cucurbitae	0	XP_028898363.1
ncbi_105214859	BcucOR49	1181	385	Complete ORF	7	odorant receptor 59a-like isoform X1	Bactrocera cucurbitae	0	XP_011186833.1
ncbi_105209394	BcucOR50	1282	384	Complete ORF	4	odorant receptor	Bactrocera cucurbitae	0	QKN21160.1
ncbi_105209796	BcucOR51	1580	384	Complete ORF	5	odorant receptor 33b-like	Bactrocera cucurbitae	0	XP_011178699.1
ncbi_105219209	BcucOR52	1167	383	Complete ORF	6	odorant receptor 22c	Bactrocera cucurbitae	0	XP_011193492.1
ncbi_105221489	BcucOR53	1152	383	Complete ORF	5	odorant receptor 59a-like	Bactrocera cucurbitae	0	XP_011196821.1
ncbi_105218035	BcucOR54	1156	382	Complete ORF	6	odorant receptor 94a-like	Bactrocera cucurbitae	0	XP_028899745.1
ncbi_105209925	BcucOR55	1233	378	Complete ORF	5	odorant receptor 43a	Bactrocera cucurbitae	0	XP_028894643.1
ncbi_105221490	BcucOR56	1207	378	Complete ORF	5	odorant receptor59a-like	Bactrocera cucurbitae	0	XP_011196822.1
ncbi_105214995	BcucOR57	1291	377	Complete ORF	6	odorant receptor30a-like	Bactrocera cucurbitae	0	XP_011187028.2
ncbi_105220104	BcucOR58	1365	376	Complete ORF	6	odorant receptor Or2-like	Bactrocera cucurbitae	0	XP_028901445.1
ncbi_114803843	BcucOR59	1307	376	5ʹ missing	6	odorant receptor 59a-like	Bactrocera cucurbitae	0	XP_028895353.1
ncbi_105220102	BcucOR60	1562	375	Complete ORF	6	odorant receptor Or2-like isoform X2	Bactrocera cucurbitae	0	XP_028901447.1
ncbi_114804259	BcucOR61	1226	374	Complete ORF	6	odorant receptor 30a-like	Bactrocera cucurbitae	0	XP_028897668.1
ncbi_105221215	BcucOR62	1277	371	Complete ORF	6	odorant receptor 49b	Bactrocera cucurbitae	0	XP_028893520.1
ncbi_105212742	BcucOR63	1342	365	5ʹ missing	6	odorant receptor 7a-like	Bactrocera cucurbitae	0	XP_028896301.1
ncbi_105209389	BcucOR64	1068	355	Complete ORF	6	odorant receptor7a-like	Bactrocera cucurbitae	0	XP_028894335.1
ncbi_105209391	BcucOR65	999	332	5ʹ 3ʹ missing	6	odorant receptor2a-like	Bactrocera cucurbitae	0	XP_011178077.1
ncbi_105209792	BcucOR66	831	276	5ʹ 3ʹ missing	3	odorant receptor 33b-like	Bactrocera cucurbitae	0	XP_011178696.1
MSTRG.3140	BcucOR67	1574	234	5ʹ 3ʹ missing	2	odorant receptor	Bactrocera cucurbitae	0	QKN21124.1
ncbi_105210511	BcucOR68	822	233	Complete ORF	3	odorant receptor85b-like	Bactrocera cucurbitae	0	XP_011179822.1
ncbi_114805287	BcucOR69	643	214	5ʹ 3ʹ missing	3	odorant receptor 33b-like	Bactrocera cucurbitae	0	XP_028901739.1
ncbi_105220556	BcucOR70	530	93	5ʹ missing	2	odorant receptor 43a-like	Bactrocera cucurbitae	0	XP_028901711.1

Twenty-one GR transcripts in the antennal transcriptome of B. cucurbitae were identified. Of these, 10 BcucGRs were found to have putative full-length open reading frames (ORFs) with six to seven transmembrane domains. These ORFs encode proteins ranging from 330 to 434 amino acids in length.

**FIGURE 6:**
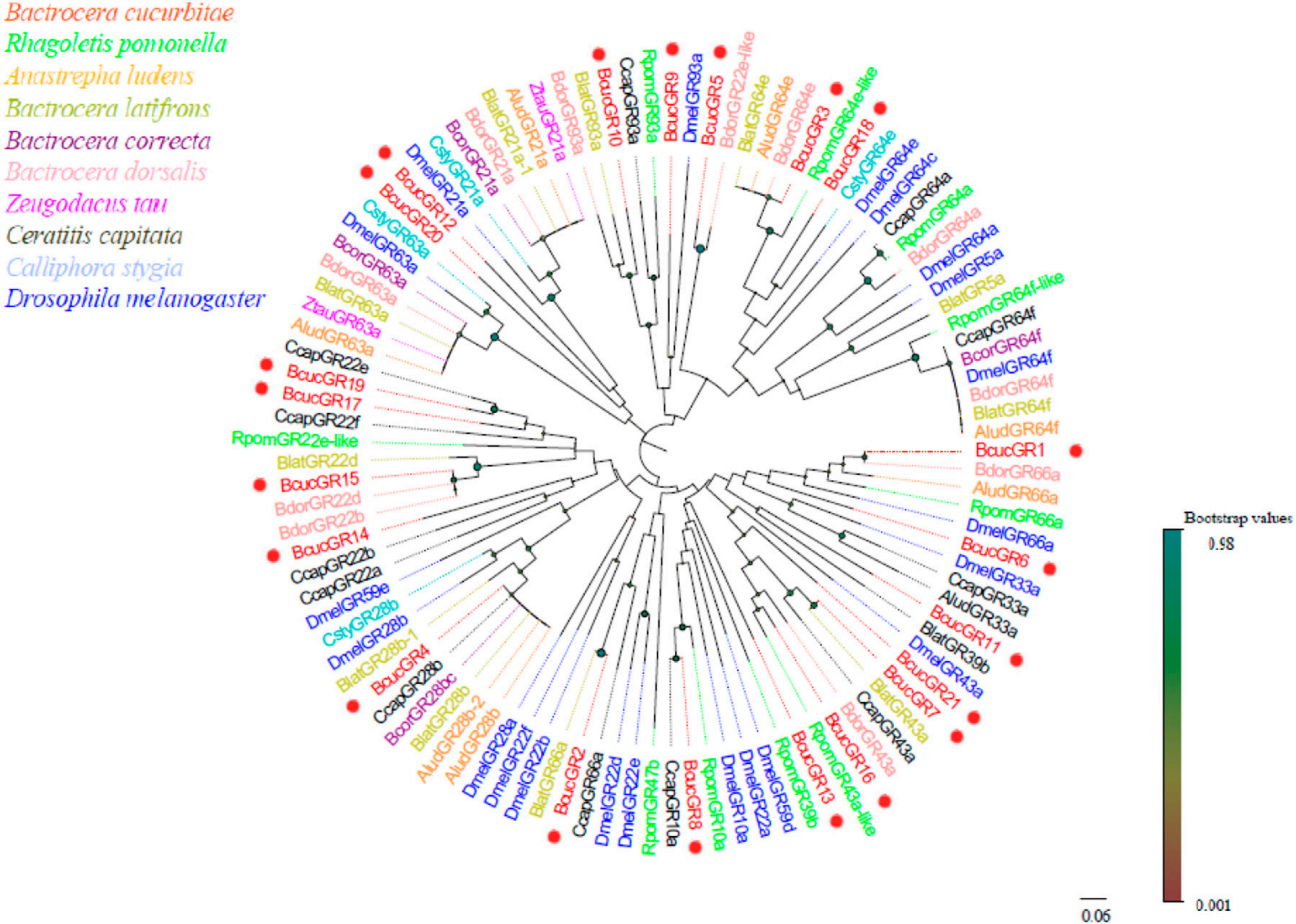
Neighbour joining phylogenetic tree showing the candidate BcucGRs with known Diptera GR sequences. Rpom, *Rhagoletis pomonella* (N = 26); Alud, Anastrepha ludens (N = 20); Blat, Bactrocera latifrons (N = 25); Bcor, Bactrocera correcta (N = 8); Bdor, Bactrocera dorsalis (N = 34); Ztau, Zeugodacus tau (N = 6); Ccap, Ceratitis capitata (N = 8); Csty, Calliphora stygia (N = 4); Dmel, *Drosophila melanogaster* (N = 59). The candidate BcucGRs are indicated by red circles.

Based on phylogenetic analysis, BcucGRs were classified into three subfamilies: sugar, bitter, and saponin receptors (Figure 6). Within these subfamilies, specific clustering patterns were observed among the BcucGRs. For instance, BcucGR7/16/21 showed homology and clustered with the GR43a sugar receptors, while the BcucGR3 and BcucGR18 were grouped with the GR64a, GR64c, GR64e, and GR64f sugar receptors. Furthermore, BcucGR8 was as a GR10a sugar receptor and BcucGR5/14/15/17/19 were identified as co-receptors of BcucGRs and clustered with the GR22a-f sugar receptors. BcucGR12 and BcucGR20 were also identified as homologues of GR21a, which mediates CO_2_ recognition, while BcucGR1/2/9/10 correspond to the GR66a, GR33a, and GR93a bitter receptors, respectively. Notably, BcucGR4 formed a GR28b branch, indicating its classification as a saponin receptor. For detailed information on the identified GRs, including the unigene reference, length, and best Blastx hit, please refer to Supplementary Material S5.

**FIGURE 7:**
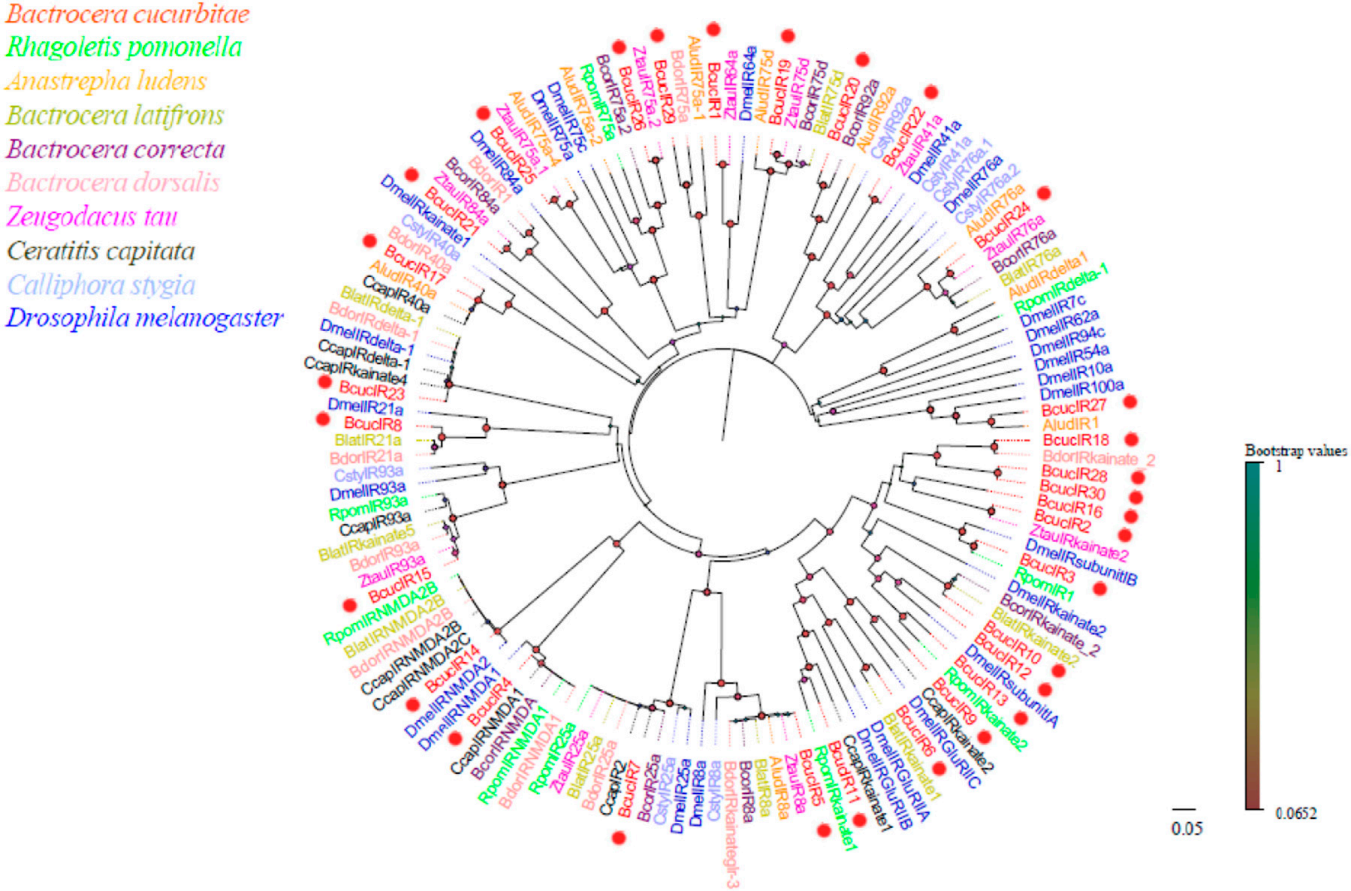
Neighbour joining phylogenetic tree for the candidate BcucIRs with known Diptera IR sequences. Rpom, *Rhagoletis pomonella* (N = 9); Alud, Anastrepha ludens (N = 10); Blat, Bactrocera latifrons (N = 10); Bcor, Bactrocera correcta (N = 9); Bdor, Bactrocera dorsalis (N = 11); Ztau, Zeugodacus tau (N = 11); Ccap, Ceratitis capitata (N = 10); Csty, Calliphora stygia (N = 8); Dmel, *Drosophila melanogaster* (N = 26). The candidate BcucIRs are indicated by red circles.

Thirty IR transcripts were identified in the antennal transcriptome of *B. cucurbitae*, and they ranged from 1,249 to 5,216 bp in length, and encode proteins with 365–1,425 amino acids. Phylogenetic analysis revealed that the *BcucIRs* clustered together with other IRs, indicating their evolutionary relatedness (Figure 7). The phylogenetic tree showed that IR genes exhibited relatively close evolutionary distances, suggesting a certain degree of conservation. Specifically, *BcucIR2/3/6/9/10/11/12/13/16/17/18/23/28/30* were found to cluster with IRkainate clades in the phylogenetic tree, while *BcucIR4* and *BcucIR14* formed the IRNMDA branch. *BcucIR5* and *BcucIR7* were associated with the coreceptors IR25a and IR8a, respectively, and clustered with conserved branches. *BcucIR1* clustered with DmelIR64a, *BcucIR21* clustered with DmelIR84a, and *BcucIR25/26/29* were grouped with IR75a. Information, including the unigene reference, length, and best BlastX hit for all identified IRs can be found in Supplementary Material S5.

RT-qPCR analysis of the 25 ORs and 15 OBPs in different B. cucurbitae tissues, including the male and female antennae, heads, thorax, abdomen, legs, and wings, revealed distinct tissue expression profiles.

**FIGURE 8:**
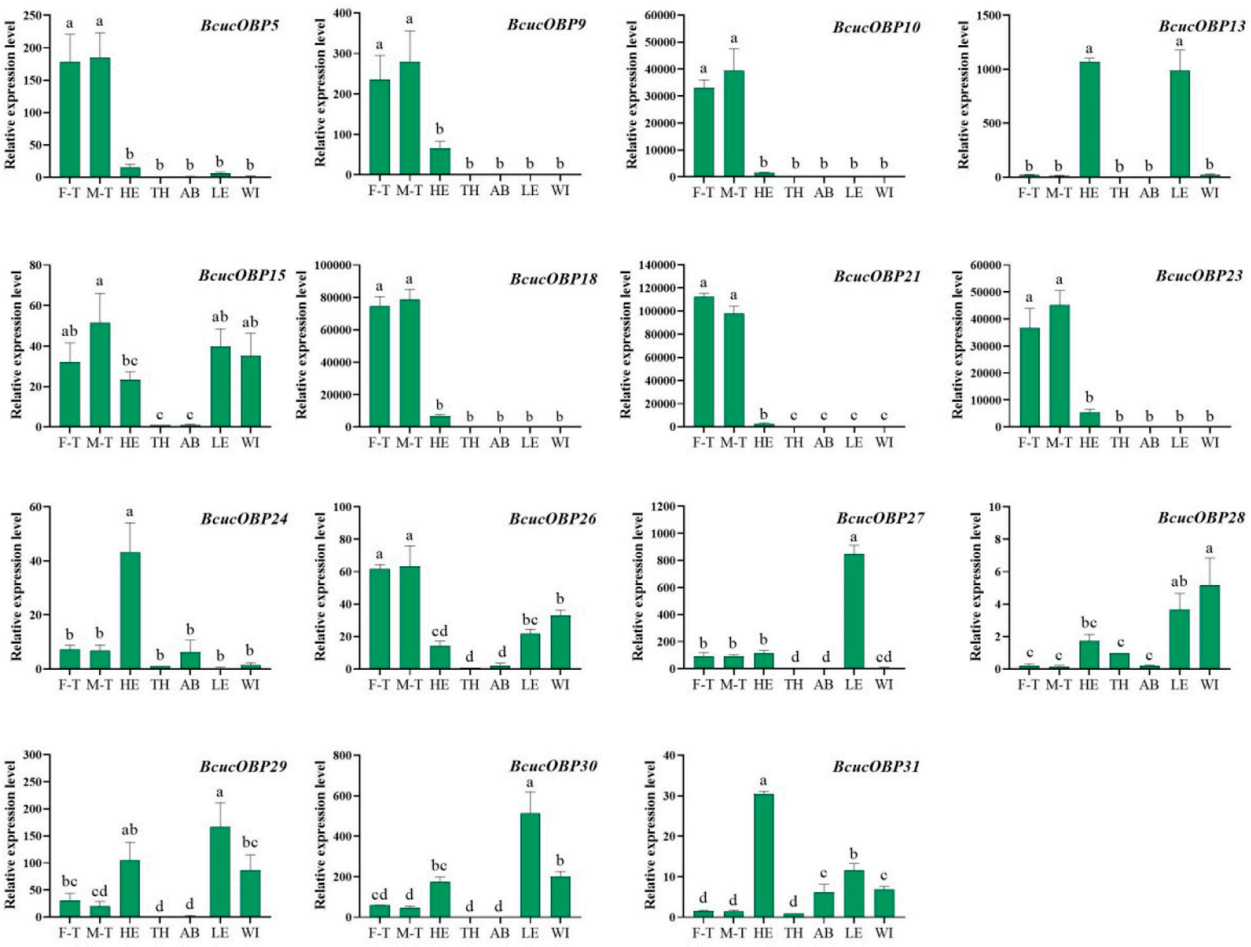
Expression profiles for 15 OBPs in adult male antennae (M–T), female antennae (F–T), head (HE), thorax (TH), abdomen (AB), legs (LE), and wings (WI) using qPCR. The relative expression level is indicated as the mean ± SE (N = 3). Different capital letters indicate significant difference between tissues (*p* < 0.05, ANOVA, LSD).

Among the OBPs, BcucOBP5/9/10/18/21/23/26 showed significantly higher expression in the male and female antennae than in other tissues. BcucOBP13 exhibited the highest expression in the head and legs, whereas BcucOBP24 and BcucOBP31 were significantly more highly expressed in the head and BcucOBP27 and BcucOBP30 were significantly expressed in the legs (Figure 8).

**FIGURE 9:**
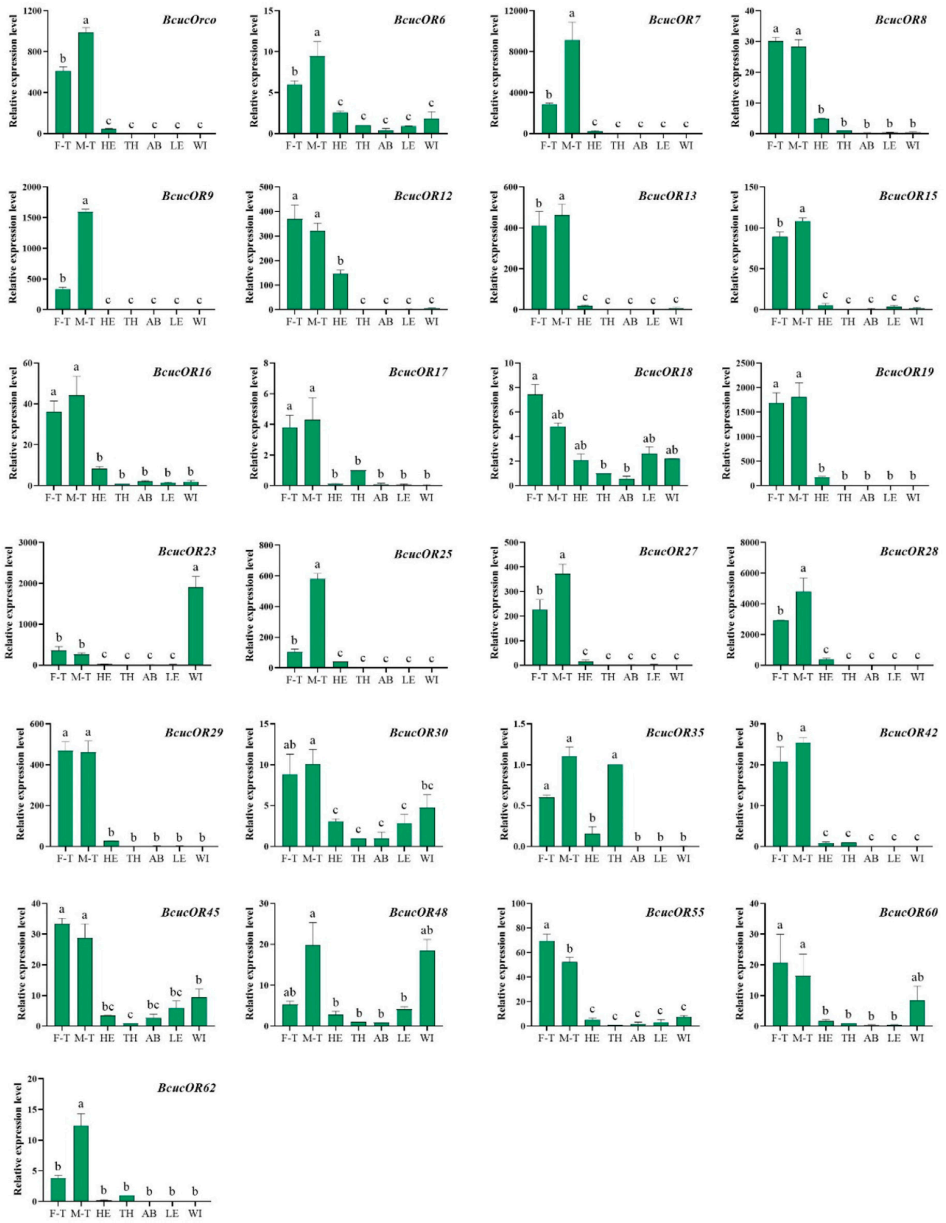
Expression profiles for 25 ORs in adult male antennae (M–T), female antennae (F–T), head (HE), thorax (TH), abdomen (AB), legs (LE), and wings (WI) using qPCR. The relative expression level is indicated as mean ± SE (N = 3). Different capital letters indicate significant differences between tissues (*p* < 0.05, ANOVA, LSD).

In terms of OR expression, *BcucOR23* was significantly expressed in the wings, *BcucOR35* showed significantly higher expression in the antennae and thoraxes, and *BcucOR48* was significantly expressed in the antennae and wings. *BcucOR18* and *BcucOR30* were expressed in all the examined tissues. The remaining ORs are predominantly expressed in the antennae. Among them, the expression levels of *BcucOrco/6/7/9/13/15/25/27/28/42/62* were significantly higher in the male than in the female antennae. In contrast, *BcucOR55* exhibited a significantly higher level of expression in the female antennae when compared with the male antennae (Figure 9).

The results provide insights into the tissue-specific expression patterns of ORs and OBPs in *B. cucurbitae* and highlight their potential roles in olfactory perception and signalling.

## Discussion

*B. cucurbitae* is a significant pest that feeds on plants and many countries now use quarantines to aid in its control (Shivaramu et al., 2022). Although some plant-based compounds have been identified that can help with the control and quarantine of this pest (Miller et al., 2004; Vargas et al., 2018; Piñero et al., 2020), research to elucidate the chemical communications in *B. cucurbitae* has been limited. *B. cucurbitae* olfaction research, in particular, has been hindered by the absence of transcriptome data for its primary olfactory organs, the antennae (Elfekih et al., 2016). While this study has tried to address this, only OBP and GR were reported on, and data on OR, which plays a key role in olfactory signal transduction, was lacking. There is thus a need for more complete transcriptome data to aid with future *B. cucurbitae* olfactory research.

The antenna is the major organ for insect olfactory sensing, and genes highly expressed in the antennae may be involved in olfactory recognition. The OBP involved in *B. cucurbitae* host selection can be screened by in vitor, purify and functionally verify the odourant binding protein in the antenna of *B. cucurbitae*, and this can provide a theoretical foundation for the development of *B. cucurbitae* attractants. Although Wu et al. (2020) carefully analysed and compared the transcriptomes of five *Bactrocera* species, the tissue-specific expression of *B. cucurbitae* was not fully elucidated. To address this, transcriptomic sequencing analysis of the antennae from adult male and female *B. cucurbitae* was conducted. In total, 160 potential olfactory receptor proteins were identified, including 70 ORs, 35 OBPs, 1 CSP, 30 IRs, 21 GRs, and 3 SNMPs. Compared with the previous results, this study found more OBP, OR, GR, less CSP and IR. Different genes found from those reported may be caused by transcriptome analysis methods and different strains of insects. These findings significantly enhance our understanding of the olfactory-related profile of *B. cucurbitae* and provide crucial insights into its chemosensory mechanisms. Additionally, we have employed quantitative polymerase chain reaction (qPCR) to validate the expression patterns of 25 ORs and 15 OBPs in various *B. cucurbitae* tissues, enabling the functional roles of these olfactory genes to be assessed.

The ability of an insect to recognise odours relies heavily on their OBPs (Barbosa et al., 2018), as these small, water-soluble proteins facilitate the transport of odour molecules through the sensory lymph and serve as connections between external environmental stimuli and odour receptors (Pelosi et al., 2006; Leal, 2013; Han et al., 2020). In this study, 35 candidate OBPs were identified in the antennal transcriptome of *B. cucurbitae*. This is lower than the number found in *D. melanogaster* (52 OBPs; Vieira and Rozas, 2011) but similar to the number found in *Zeugodacus. papayae* (35 OBPs; Wu et al., 2020), *B. correcta* (34 OBPs; Wu et al., 2020), *A. ludens* (31 OBPs; Segura-Leon et al., 2022), *Z. tau* (33 OBPs; Wu et al., 2020), and *B. dorsalis* (31 OBPs; Wu et al., 2020), and higher than that in *C. stygia* (28 OBPs; Leitch et al., 2015) and *B. minax* (25 OBPs; Cheng et al., 2020). Furthermore, the number of *BcucOBPs* was different from that previously reported (13 and 38 OBPs), and this wide variation in the number of OBPs may be attributed to the differences in the physiological stages during transcriptome analysis, sequencing methodologies, sequencing depth, sample preparation, and coverage of the sequencing platform (Wu et al., 2015; Liu et al., 2016; Cheng et al., 2020).

Phylogenetic analysis showed that *BcucOBP23* forms a clade with other Dipteran LUSH proteins known to have a specific binding affinity for the pheromone 11-cis vaccenyl acetate. This suggests that *BcucOBP23* is involved in pheromone recognition (Laughlin et al., 2008; Wu et al., 2020). Additionally, *BcucOBP23* was highly expressed in both male and female antennae, indicating a potential role in pheromone recognition by melon flies. Furthermore, *BcucOBP27* was also identified, and clustered with other Diptera species in the OBP28a clade and is associated with olfactory sensitivity modulation (Larter et al., 2016).

The expansion of OBP gene families is a common occurrence, and orthologues of the OBPs play similar roles in olfactory functions (Vieira et al., 2007; Rihani et al., 2021). In *B. cucurbitae*, the expansion of the OBP19d (*BcucOBP8/15/25/26/29/30*), OBP56a (*BcucOBP6* and *BcucOBP12*), OBP56d (*BcucOBP31/33/35*), OBP56 h (*BcucOBP13/32/34*), OBP84a (*BcucOBP9* and *BcucOBP10*), and OBP99a (*BcucOBP14/19/24/28*) branches was observed. In particular, OBP84a is involved in detecting organic acids and amines (Larter et al., 2016), while OBP56 h modulates mating behaviour (Swarup et al., 2011).

Previously, OBPs were thought to be exclusively expressed in olfactory organs and solely involved in chemoreception (Damberger et al., 2013; Shorter et al., 2016; Rihani et al., 2021). However, recent reports have shown that OBPs are expressed in multiple insect organs, including the legs (Yasukawa et al., 2010; Chen et al., 2019; Gu et al., 2019), proboscis (Mitaka et al., 2011; Rihani et al., 2016), labellum and tarsus (Jeong et al., 2013; Sparks et al., 2014), wings (Galindo and Smith, 2001; Calvello et al., 2005), reproductive organs (Findlay et al., 2008; Takemori and Yamamoto, 2009; Sun et al., 2012; Costa-Da-Silva et al., 2013; Sepil et al., 2019), abdomen (Xu et al., 2010; Andersson et al., 2014), eyes (Zhu et al., 2016; Rihani et al., 2021), and digestive tract (Ishida et al., 2013; Benoit et al., 2017). These OBPs reportedly have unconventional roles, including involvement in taste perception (Yasukawa et al., 2010; Jeong et al., 2013), immune response (Benoit et al., 2017), and humidity detection (Sun et al., 2018).

In this study, high expression levels for *BcucOBP5/9/10/18/21/23/26* were observed in the antennae of both male and female *B. cucurbitae*. As few studies have investigated the functions of *BcucOBPs,* their functions in the host recognition of *B. cucurbitae* were investigated by gene cloning, construction of a prokaryotic expression vector, expression and purification of recombinant proteins, fluorescence competitive binding experiment, RNAi, and other technologies.

CSPs, another class of soluble proteins found in the sensory lymph, are also highly expressed in insects (Forêt et al., 2007). A CSP was identified in the study of the *B. cucurbitae* antennal transcriptome, and a BLASTX comparison revealed its high level of similarity to CSP genes in other insects. However, our understanding of the evolution and function of CSPs is currently limited to data from only a few species (Yi et al., 2013; Wu et al., 2019; Xu et al., 2019; Wang et al., 2021). Further comprehensive research on CSPs will thus be required to elucidate their diverse functions.

SNMPs were initially discovered in the pheromone-sensitive neurones of Lepidoptera (Rogers et al., 2001) and are associated with pheromone recognition (Vogt et al., 2009; Gomez-Diaz et al., 2016; Cassau and Krieger, 2021). They are generally categorised into two families, SNMP1 and SNMP2, with SNMP1 further divided into the SNMP1a and SNMP1b subtypes (Jiang et al., 2016). Three SNMPs were identified in the *B. cucurbitae* transcriptome, however, the function of *BcucSNMPs* in the sexual communication of *B. cucurbitae* needs to be further studied.

In the insect olfactory system, ORs are known to play a central role (Leal, 2013). *B. cucurbitae* has 70 putative ORs, which is similar to the number in *D. melanogaster* (63 ORs; Gao and Chess, 1999) and *Anopheles gambiae* (76 ORs; Hill et al., 2002), but higher than the number in *Z. papayae* (41 ORs; Wu et al., 2020), *B. correcta* (39 ORs; Wu et al., 2020), *B. minax* (53 ORs; Cheng et al., 2020), *Z. tau* (39 ORs; Wu et al., 2020), *B. dorsalis* (35 ORs; Wu et al., 2015), *C. stygia* (50 ORs; Leitch et al., 2015), *Glossina morsitans* (50 ORs; Obiero et al., 2014), and *A. ludens* (42 ORs; Segura-Leon et al., 2022). It seems that *Bactrocera* species requires homologous ORs to detect specific odorants. Highly differentiated and expanded ORs indicate the continuous evolution of insect odor detection. The expansion of OR7a, OR67d, and OR74a suggests their involvement in pheromone acceptance and insect behaviour. Tissue expression profiling has shown that all ORs are expressed in the antennae, with differences between the male and female antennae indicating their potential roles in sex pheromone recognition and other behaviours (Lin et al., 2015; Wu et al., 2020). Future studies should focus on the functions of these OR genes to provide potential molecular targets for the development of new insect behaviour regulators.

Phylogenetic tree analysis revealed that *BcucOrco* is clustered into a specific co-receptor lineage with other Dipteran species. The function of Orco has been studied in a large variety of insects, may be potentially involved in oviposition, mating, or social behavior (Fan et al., 2022; David et al., 2023; Liu et al., 2023). The olfactory function of Orco can be further confirmed by RNAi technology and EAG reaction analysis. For example, the mating rate and response to sex pheromone of *BoleOrco* decreased significantly after injecting dsRNA (Tsoumani et al., 2020). Xu et al. (2022) used the CRISPR/Cas9 system to knockout *BdorOrco*, olfactory preference assays and oviposition behavior are significantly affected. These research can provide a reference for the functional verification of *BcucOrco*.

There were 30 IRs identified in *B. cucurbitae*, which is fewer than in *D. melanogaster* (66 IRs; Rytz et al., 2013), but similar to the number in *A. gambiae* (30 IRs; Hill et al., 2002), *Z. papayae* (23 IRs; Wu et al., 2020), *B. correcta* (23 IRs; Wu et al., 2020), *Z. tau* (23 IRs; Wu et al., 2020), *C. stygia* (22 IRs; Leitch et al., 2015), *B. dorsalis* (23 IRs; Wu et al., 2020), and higher than the number in *A. ludens* (17 IRs) (Segura-León et al., 2022). *BcucIR5* and *BcucIR7* belong to the conserved IR25a/IR8a co-expression group. *BcucIR1* may detect acids while *BcucIR21* may recognise phenylacetic acid and phenylacetaldehyde, while *BcucIR25/26/29* may be involved in acetic acid sensing and the enhanced perception of fermented fruits (Ai et al., 2010; Grosjean et al., 2011; Gorter et al., 2016).

There were 21 candidate GRs identified in *B. cucurbitae*, which is fewer than in *D. melanogaster* (59 GRs; Robertson et al., 2003), but similar to the number in *A. ludens* (24 GRs; Segura-León et al., 2022), and *C. stygia* (21 GRs; Leitch et al., 2015), and higher than the number in *A. gambia*e (10 GRs; Hill et al., 2002), *Z. papayae* (9 GRs; Wu et al., 2020), *Z. tau* (6 GRs; Wu et al., 2020), *B. correcta* (9 GRs; Wu et al., 2020), and *B. dorsalis* (6 GRs; Wu et al., 2015). Phylogenetic analysis grouped *BcucGRs* into different clades with specific putative functions. *BcucGRs* include sugar receptors (*BcucGR3/5/7/8/14/15/16/17/18/19*), bitter receptors (*BcucGR1/2/7)*, CO_2_ receptors (*BcucGR12* and *BcucGR12*), and saponin receptors (*BcucGR4*) (Lee et al., 2009; Apostolopoulou et al., 2016; Poudel and Lee, 2016; Rass et al., 2019; Sang et al., 2019).

In summary, the expression patterns and observed gene expansion for the ORs, IRs, and GRs indicates that they are involved in odour detection, pheromone reception, and host plant selection in *B. cucurbitae* (Kurtovic et al., 2007; Liu et al., 2018; MacWilliam et al., 2018; Rebecca and John, 2016; Ono et al., 2021; Baumgartner et al., 2022; De Obaldia et al., 2022).

## Conclusion

The extensive set of chemosensory genes identified in this study will provide a solid foundation for future research into the molecular communications of *B. cucurbitae* and advances our understanding of olfactory mechanisms in Diptera. Moreover, the discovery of high antenna and sex-biased chemosensory expression proteins will also help to guide functional studies of key olfactory genes in *B. cucurbitae* **.**

## Data Availability

The datasets presented in this study can be found in online repositories. The names of the repository/repositories and accession number(s) can be found below: https://www.ncbi.nlm.nih.gov/bioproject/PRJNA1019880.
