# Supplementary material for: Identification of candidate chemosensory genes in Bactrocera cucurbitae based on antennal transcriptome analysis
Source: Front Physiol. 2024 Feb 19;15:1354530. doi: 10.3389/fphys.2024.1354530 (PMC10910661; doi:10.3389/fphys.2024.1354530)
Supplement: Supplementary file 1 [file Table1.docx]

Supplementary Material S1

Table1 Date statistics of *Bactrocera cucurbitae* antennal transcriptome

| Sample | Raw reads | Clean  reads | Valid  bases(%) | Clean bases | Q20(%) | Q30(%) | GC content(%) |
| --- | --- | --- | --- | --- | --- | --- | --- |
| F-1 | 40256220 | 38685052 | 96.10 | 2.6GB | 97.23 | 92.95 | 41.84 |
| F-2 | 40261284 | 38898716 | 96.62 | 2.64GB | 97.31 | 93.00 | 42.00 |
| F-3 | 38918462 | 37996894 | 97.63 | 2.56GB | 97.58 | 93.47 | 41.31 |
| M-1 | 38817130 | 37608644 | 96.89 | 2.52GB | 97.49 | 93.48 | 41.24 |
| M-2 | 43039494 | 42083448 | 97.78 | 2.84GB | 97.55 | 93.47 | 41.23 |
| M-3 | 39255816 | 38107760 | 97.08 | 2.58GB | 97.50 | 93.32 | 41.84 |
