# Supplementary material for: Identification of candidate chemosensory genes in Bactrocera cucurbitae based on antennal transcriptome analysis
Source: Front Physiol. 2024 Feb 19;15:1354530. doi: 10.3389/fphys.2024.1354530 (PMC10910661; doi:10.3389/fphys.2024.1354530)
Supplement: Supplementary file 2 [file Table2.docx]

Supplementary Material S2

Table 2 Comparison between sequencing reads and reference genome

| Sample | Total reads^a^ | Total mapped  reads^b^ (%) | Multiple mapped  reads^c^(%) | Unique mapped  reads^d^(%) |
| --- | --- | --- | --- | --- |
| F-1 | 36742486 | 30825764 (83.90%) | 299822 (0.82%) | 30525942 (83.08%) |
| F-2 | 37881986 | 32923773 (86.91%) | 312940 (0.83%) | 32610833 (86.09%) |
| F-3 | 36613520 | 32984079 (90.09%) | 304898 (0.83%) | 32679181 (89.25%) |
| M-1 | 36058276 | 30551288 (84.73%) | 286182 (0.79%) | 30265106 (83.93%) |
| M-2 | 40854562 | 36033702 (88.20%) | 331831 (0.81%) | 35701871 (87.39%) |
| M-3 | 37138440 | 33104612 (89.14%) | 300906 (0.81%) | 32803706 (88.33%) |

Note: a: The number of reads removed from the above ribosome after filtration of each sample sequence was the most statistical;

b: The number of reads located on the reference genome was statistically statistically higher than 70%, indicating that there was no contamination in the sequencing data and the reference genome or reference sequence was selected appropriately.

c: Number statistics of reads with multiple comparison locations in the sequence;

d: There are statistics on the number of reads in the unique comparison position in the reference sequence.
