# Supplementary material for: Identification of candidate chemosensory genes in Bactrocera cucurbitae based on antennal transcriptome analysis
Source: Front Physiol. 2024 Feb 19;15:1354530. doi: 10.3389/fphys.2024.1354530 (PMC10910661; doi:10.3389/fphys.2024.1354530)
Supplement: Supplementary file 3 [file Table3.docx]

Supplementary Material S3: The Blastx match of *Bactrocera cucurbitae* candidate chemosensory proteins, sensory neuron membrane proteins, gustatory receptors and ionotropic receptors

| **Unigene ID** | ***NAME*** | **Length** | **ORF**  **(aa)** | **Status** | **Signal**  **peptide** | **Blastx best-hit** | ***Species*** | **E-value** | **Ident** | **Accession** |
| --- | --- | --- | --- | --- | --- | --- | --- | --- | --- | --- |
| **ncbi_105208856** | ***ZcucCSP1*** | **2263** | **573** | **5' 3' missing** | **Y** | **putative odorant-binding protein** | ***Zeugodacus cucurbitae*** | **0** | **100%** | **QKN21231.1** |
| **ncbi_105221014** | ***ZcucSNMP1*** | **2263** | **573** | **5' 3' missing** | **Y** | **sensory neuron membrane protein 2** | ***Zeugodacus cucurbitae*** | **0** | **100%** | **XP_028901954.1** |
| **ncbi_105210080** | ***ZcucSNMP2*** | **1887** | **537** | **5' 3' missing** | **Y** | **sensory neuron membrane protein 1a** | ***Zeugodacus cucurbitae*** | **0** | **100%** | **XP_011179120.1** |
| **ncbi_105212190** | ***ZcucSNMP3*** | **1777** | **496** | **5' 3' missing** | **Y** | **sensory neuron membrane protein 1b** | ***Zeugodacus cucurbitae*** | **0** | **100%** | **QKN21194.1** |

The Blastx match of *B. cucurbitae* candidate chemosensory proteins and sensory neuron

The Blastx match of *Bactrocera cucurbitae* candidate gustatory receptors

| **Unigene ID** | **NAME** | **Length** | **ORF**  **(aa)** | **Status** | **TMD** | **Blastx best-hit** | **Species** | **E-value** | **Ident** | **Accession** |
| --- | --- | --- | --- | --- | --- | --- | --- | --- | --- | --- |
| **ncbi_105221169** | ***ZcucGR1*** | **1929** | **526** | **5' 3' missing** | **6** | **gustatory receptor for bitter taste 66a** | ***Zeugodacus cucurbitae*** | **0** | **100%** | [**XP_011196225.1**](https://www.ncbi.nlm.nih.gov/protein/XP_011196225.1?report=genbank&log$=prottop&blast_rank=1&RID=TDG1E8YY013) |
| **ncbi_105210038** | ***ZcucGR2*** | **1482** | **493** | **5' 3' missing** | **8** | **gustatory receptor for bitter taste 66a-like** | ***Zeugodacus cucurbitae*** | **0** | **100%** | [**XP_011179059.1**](https://www.ncbi.nlm.nih.gov/protein/XP_011179059.1?report=genbank&log$=prottop&blast_rank=1&RID=TDGWKD14016) |
| **ncbi_105211608** | ***ZcucGR3*** | **1607** | **468** | **5' 3' missing** | **6** | **gustatory receptor for sugar taste 64e-like isoform X1** | ***Zeugodacus cucurbitae*** | **0** | **100%** | **XP_028895660.1** |
| **ncbi_105210855** | ***ZcucGR4*** | **1726** | **445** | **3' missing** | **8** | **putative gustatory receptor 28b isoform X5** | ***Zeugodacus cucurbitae*** | **0** | **100%** | [**XP_011180327.1**](https://www.ncbi.nlm.nih.gov/protein/XP_011180327.1?report=genbank&log$=prottop&blast_rank=1&RID=TDHUTPEZ013) |
| **ncbi_105214213** | ***ZcucGR5*** | **1305** | **434** | **Complete ORF** | **7** | **gustatory receptor for bitter taste 22e-like** | ***Zeugodacus cucurbitae*** | **0** | **100%** | **XP_011185808.1** |
| **ncbi_105213493** | ***ZcucGR6*** | **1979** | **426** | **3' missing** | **6** | **putative gustatory receptor 22c** | ***Zeugodacus cucurbitae*** | **0** | **100%** | **XP_011184670.1** |
| **ncbi_105216807** | ***ZcucGR7*** | **1279** | **423** | **Complete ORF** | **7** | **gustatory receptor for sugar taste 43a** | ***Zeugodacus cucurbitae*** | **0** | **100%** | [**XP_011189783.1**](https://www.ncbi.nlm.nih.gov/protein/XP_011189783.1?report=genbank&log$=prottop&blast_rank=1&RID=TDK26CPJ016) |
| **ncbi_105213488** | ***ZcucGR8*** | **1230** | **409** | **Complete ORF** | **7** | **gustatory receptor 10a** | ***Zeugodacus cucurbitae*** | **0** | **100%** | **XP_011184666.1** |
| **ncbi_105213853** | ***ZcucGR9*** | **1373** | **405** | **5' 3' missing** | **7** | **gustatory receptor 8a-like isoform X1** | ***Zeugodacus cucurbitae*** | **0** | **100%** | [**XP_011185249.1**](https://www.ncbi.nlm.nih.gov/protein/XP_011185249.1?report=genbank&log$=prottop&blast_rank=1&RID=TDM8N557013) |
| **ncbi_105213330** | ***ZcucGR10*** | **1212** | **403** | **Complete ORF** | **7** | **gustatory receptor for bitter taste 93a** | ***Zeugodacus cucurbitae*** | **0** | **100%** | **XP_011184365.1** |
| **ncbi_105219523** | ***ZcucGR11*** | **1194** | **397** | **Complete ORF** | **6** | **gustatory receptor 23a-like** | ***Zeugodacus cucurbitae*** | **0** | **100%** | **XP_011194023.1** |
| **ncbi_105213855** | ***ZcucGR12*** | **2076** | **389** | **5' 3' missing** | **4** | **gustatory receptor 8a-like isoform X1** | ***Zeugodacus cucurbitae*** | **0** | **100%** | [**XP_028897227.1**](https://www.ncbi.nlm.nih.gov/protein/XP_028897227.1?report=genbank&log$=prottop&blast_rank=1&RID=TDP3AJUU01N) |
| **ncbi_105213491** | ***ZcucGR13*** | **1161** | **386** | **Complete ORF** | **7** | **putative gustatory receptor 59d** | ***Zeugodacus cucurbitae*** | **0** | **100%** | [**XP_011184668.1**](https://www.ncbi.nlm.nih.gov/protein/XP_011184668.1?report=genbank&log$=prottop&blast_rank=1&RID=TDPEPXX8013) |
| **ncbi_105214216** | ***ZcucGR14*** | **1104** | **367** | **Complete ORF** | **7** | **putative gustatory receptor 22a** | ***Zeugodacus cucurbitae*** | **0** | **100%** | **XP_028897457.1** |
| **ncbi_114804224** | ***ZcucGR15*** | **1241** | **362** | **5' 3' missing** | **6** | **putative gustatory receptor 22b** | ***Zeugodacus cucurbitae*** | **0** | **100%** | **XP_028897434.1** |
| **ncbi_105216832** | ***ZcucGR16*** | **1053** | **350** | **Complete ORF** | **6** | **gustatory receptor for sugar taste 43a-like** | ***Zeugodacus cucurbitae*** | **0** | **100%** | [**XP_028899050.1**](https://www.ncbi.nlm.nih.gov/protein/XP_028899050.1?report=genbank&log$=prottop&blast_rank=1&RID=TDS09P0P013) |
| **ncbi_105214214** | ***ZcucGR17*** | **1032** | **343** | **Complete ORF** | **6** | **gustatory receptor for bitter taste 22e-like** | ***Zeugodacus cucurbitae*** | **0** | **100%** | [**XP_028897456.1**](https://www.ncbi.nlm.nih.gov/protein/XP_028897456.1?report=genbank&log$=prottop&blast_rank=1&RID=TDSFT259013) |
| **ncbi_105211607** | ***ZcucGR18*** | **1169** | **335** | **5' 3' missing** | **6** | **gustatory receptor for sugar taste 64c-like** | ***Zeugodacus cucurbitae*** | **0** | **100%** | **XP_028895662.1** |
| **ncbi_105214215** | ***ZcucGR19*** | **993** | **330** | **Complete ORF** | **6** | **gustatory receptor for bitter taste 22e-like** | ***Zeugodacus cucurbitae*** | **0** | **100%** | [**XP_011185810.1**](https://www.ncbi.nlm.nih.gov/protein/XP_011185810.1?report=genbank&log$=prottop&blast_rank=1&RID=TDTA8BFG016) |
| **ncbi_105210898** | ***ZcucGR20*** | **744** | **239** | **5' missing** | **5** | **putative gustatory receptor 28b** | ***Zeugodacus cucurbitae*** | **0** | **100%** | **XP_011180397.1** |
| **ncbi_114804508** | ***ZcucGR21*** | **731** | **220** | **5' missing** | **3** | **gustatory receptor for sugar taste 43a-like** | ***Zeugodacus cucurbitae*** | **0** | **100%** | **XP_028899051.1** |

The Blastx match of *Bactrocera cucurbitae* candidate ionotropic receptors

| **Unigene ID** | **NAME** | **Length** | **ORF**  **(aa)** | **Status** | **TMD** | **Blastx best-hit** | **Species** | **E-value** | **Ident** | **Accession** |
| --- | --- | --- | --- | --- | --- | --- | --- | --- | --- | --- |
| **ncbi_105211160** | ***ZcucIR1*** | **5216** | **1425** | **5' 3' missing** | **3** | **ionotropic receptor** | ***Zeugodacus cucurbitae*** | **0** | **99%** | [**QKN21168.1**](https://www.ncbi.nlm.nih.gov/protein/QKN21168.1?report=genbank&log$=prottop&blast_rank=2&RID=T5J93SXC016) |
| **ncbi_105208469** | **ZcucIR2** | **3564** | **1082** | **5' 3' missing** | **2** | **glutamate receptor ionotropic ,kainate 2** | ***Zeugodacus cucurbitae*** | **0** | **100%** | [**XP_011176626.1**](https://www.ncbi.nlm.nih.gov/protein/XP_011176626.1?report=genbank&log$=prottop&blast_rank=1&RID=T5JXCCAJ013) |
| **ncbi_105218571** | **ZcucIR3** | **3380** | **1006** | **5' 3' missing** | **3** | **glutamate receptor 1** | ***Zeugodacus cucurbitae*** | **0** | **100%** | **XP_011192554.1** |
| **ncbi_105221596** | **ZcucIR4** | **3076** | **985** | **5' 3' missing** | **3** | **glutamate receptor subunit 1** | ***Zeugodacus cucurbitae*** | **0** | **100%** | **XP_011196996.1** |
| **ncbi_105214572** | **ZcucIR5** | **2929** | **943** | **3' missing** | **3** | **glutamate receptor** | ***Zeugodacus cucurbitae*** | **0** | **100%** | [**QKN21163.1**](https://www.ncbi.nlm.nih.gov/protein/QKN21163.1?report=genbank&log$=prottop&blast_rank=2&RID=T5N3W9Z9013) |
| **ncbi_105208530** | **ZcucIR6** | **3533** | **940** | **5' 3' missing** | **3** | **glutamate receptor ionotropic, kainate 2** | ***Zeugodacus cucurbitae*** | **0** | **100%** | [**XP_011176721.1**](https://www.ncbi.nlm.nih.gov/protein/XP_011176721.1?report=genbank&log$=prottop&blast_rank=1&RID=T5NT5YTS016) |
| **ncbi_105209636** | **ZcucIR7** | **3302** | **940** | **5' 3' missing** | **3** | **ionotropic receptor (IR25a)** | ***Zeugodacus cucurbitae*** | **0** | **100%** | [**XP_011178452.1**](https://www.ncbi.nlm.nih.gov/protein/XP_011178452.1?report=genbank&log$=prottop&blast_rank=1&RID=T5PASSD1016) |
| **ncbi_105213077** | **ZcucIR8** | **2872** | **932** | **5' 3' missing** | **5** | **ionotropic receptor 21a** | ***Zeugodacus cucurbitae*** | **0** | **100%** | **XP_011183925.1** |
| **ncbi_105218922** | **ZcucIR9** | **2956** | **926** | **5' 3' missing** | **4** | **glutamate receptor ionotropic, kainate 2** | ***Zeugodacus cucurbitae*** | **0** | **100%** | [**XP_011193096.1**](https://www.ncbi.nlm.nih.gov/protein/XP_011193096.1?report=genbank&log$=prottop&blast_rank=1&RID=T5S9V0CX016) |
| **ncbi_105209186** | **ZcucIR10** | **3188** | **925** | **5' 3' missing** | **4** | **glutamate receptor ionotropic，kainate 2 isoform X2** | ***Zeugodacus cucurbitae*** | **0** | **100%** | **XP_011177767.1** |
| **ncbi_105208467** | **ZcucIR11** | **3254** | **917** | **5' 3' missing** | **3** | **glutamate receptor ionotropic, kainate 1** | ***Zeugodacus cucurbitae*** | **0** | **100%** | [**XP_011176625.1**](https://www.ncbi.nlm.nih.gov/protein/XP_011176625.1?report=genbank&log$=prottop&blast_rank=1&RID=T5T9EAX0016) |
| **ncbi_105214273** | **ZcucIR12** | **3719** | **904** | **5' 3' missing** | **4** | **glutamate receptor ionotropic，kainate 2 isoform X1** | ***Zeugodacus cucurbitae*** | **0** | **100%** | [**XP_011185912.1**](https://www.ncbi.nlm.nih.gov/protein/XP_011185912.1?report=genbank&log$=prottop&blast_rank=1&RID=T5TX0MAG013) |
| **ncbi_105214272** | **ZcucIR13** | **2879** | **903** | **5' 3' missing** | **3** | **glutamate receptor ionotropic, kainate 2** | ***Zeugodacus cucurbitae*** | **0** | **100%** | [**XP_011185911.1**](https://www.ncbi.nlm.nih.gov/protein/XP_011185911.1?report=genbank&log$=prottop&blast_rank=1&RID=T5UFTCR4016) |
| **ncbi_105217288** | **ZcucIR14** | **3569** | **902** | **5' 3' missing** | **3** | **glutamate receptor ionotropic, NMDA 2B** | ***Zeugodacus cucurbitae*** | **0** | **100%** | **XP_011190524.1** |
| **ncbi_105209182** | **ZcucIR15** | **3234** | **870** | **5' 3' missing** | **6** | **ionotropic receptor 93a** | ***Zeugodacus cucurbitae*** | **0** | **100%** | [**XP_011177762.1**](https://www.ncbi.nlm.nih.gov/protein/XP_011177762.1?report=genbank&log$=prottop&blast_rank=1&RID=T5VNSK7K013) |
| **ncbi_105209184** | **ZcucIR16** | **3981** | **861** | **5' 3' missing** | **3** | **glutamate receptor ionotropic, kainate 2** | ***Zeugodacus cucurbitae*** | **0** | **100%** | **XP_011177764.1** |
| **ncbi_105212100** | **ZcucIR17** | **3039** | **830** | **5' 3' missing** | **3** | **ionotropic receptor 40a isoform X1** | ***Zeugodacus cucurbitae*** | **0** | **100%** | **XP_028895918.1** |
| **ncbi_105219487** | **ZcucIR18** | **2899** | **805** | **5' 3' missing** | **3** | **glutamate receptor ionotropic kainate 2** | ***Zeugodacus cucurbitae*** | **0** | **100%** | [**QKN21183.1**](https://www.ncbi.nlm.nih.gov/protein/QKN21183.1?report=genbank&log$=prottop&blast_rank=3&RID=T5XKXVP501N) |
| **ncbi_105214604** | **ZcucIR19** | **2511** | **724** | **5' 3' missing** | **3** | **ionotropic receptor** | ***Zeugodacus cucurbitae*** | **0** | **100%** | **QKN21171.1** |
| **ncbi_105213333** | **ZcucIR20** | **2302** | **721** | **5' 3' missing** | **2** | **ionotropic receptor** | ***Zeugodacus cucurbitae*** | **0** | **100%** | [**QKN21175.1**](https://www.ncbi.nlm.nih.gov/protein/QKN21175.1?report=genbank&log$=prottop&blast_rank=3&RID=T5YR3FK6016) |
| **ncbi_105219288** | **ZcucIR21** | **2218** | **698** | **5' 3' missing** | **6** | **ionotropic receptor 1** | ***Zeugodacus cucurbitae*** | **0** | **100%** | **XP_028900766.1** |
| **ncbi_114804748** | **ZcucIR22** | **2124** | **673** | **5' 3' missing** | **3** | **ionotropic receptor** | ***Zeugodacus cucurbitae*** | **0** | **100%** | [**QKN21167.1**](https://www.ncbi.nlm.nih.gov/protein/QKN21167.1?report=genbank&log$=prottop&blast_rank=2&RID=T5ZUTPJ601N) |
| **ncbi_105210737** | **ZcucIR23** | **2337** | **661** | **5' 3' missing** | **4** | **glutamate receptor ionotropic, delta-1** | ***Zeugodacus cucurbitae*** | **0** | **100%** | **XP_011180180.1** |
| **ncbi_105210679** | **ZcucIR24** | **2235** | **660** | **5' 3' missing** | **1** | **ionotropic receptor IR76a** | ***Zeugodacus cucurbitae*** | **0** | **100%** | **QKN21172.1** |
| **ncbi_105210861** | **ZcucIR25** | **2108** | **637** | **5' 3' missing** | **6** | **ionotropic receptor75a-like** | ***Zeugodacus cucurbitae*** | **0** | **100%** | **XP_011180343.2** |
| **ncbi_105210866** | **ZcucIR26** | **1985** | **621** | **5' 3' missing** | **4** | **ionotropic receptor75a-like** | ***Zeugodacus cucurbitae*** | **0** | **100%** | [**XP_011180358.1**](https://www.ncbi.nlm.nih.gov/protein/XP_011180358.1?report=genbank&log$=prottop&blast_rank=1&RID=TDDBV6BU013) |
| **ncbi_105218820** | ***ZcucIR27*** | **1819** | **575** | **3' missing** | **4** | **glutamate receptor** | ***Zeugodacus cucurbitae*** | **0** | **100%** | **XP_028900401.1** |
| **ncbi_105220512** | ***ZcucIR28*** | **1786** | **566** | **5' missing** | **2** | **glutamate receptor ionotropic， kainate 2** | ***Zeugodacus cucurbitae*** | **0** | **100%** | [**XP_011195022.1**](https://www.ncbi.nlm.nih.gov/protein/XP_011195022.1?report=genbank&log$=prottop&blast_rank=1&RID=TDE6Y2U2016) |
| **ncbi_105220077** | ***ZcucIR29*** | **1913** | **450** | **5' 3' missing** | **2** | **ionotropic receptor 75a** | ***Zeugodacus cucurbitae*** | **0** | **100%** | [**XP_028901428.1**](https://www.ncbi.nlm.nih.gov/protein/XP_028901428.1?report=genbank&log$=prottop&blast_rank=1&RID=TDEJ2TNN013) |
| **ncbi_105220282** | ***ZcucIR30*** | **1249** | **365** | **5' 3' missing** | **3** | **glutamate receptor ionotropic kainate 2** | ***Zeugodacus cucurbitae*** | **0** | **100%** | **XP_011195022.1** |
