# Supplementary material for: Identification of candidate chemosensory genes in Bactrocera cucurbitae based on antennal transcriptome analysis
Source: Front Physiol. 2024 Feb 19;15:1354530. doi: 10.3389/fphys.2024.1354530 (PMC10910661; doi:10.3389/fphys.2024.1354530)
Supplement: Supplementary file 4 [file DataSheet2.docx]

Supplementary Material S4: Primers used for qPCR expression analyse

| *BcucOBP5*-F-q | GAACTACATCTACATCCCTGCC |
| --- | --- |
| *BcucOBP5*-R-q | CGCCTTCAAATCGCTCAAC |
| *BcucOBP9*-F-q | TGCATTTGTGGCACTTCC |
| *BcucOBP9*-R-q | CATTCTCGCTGCTCCTTGT |
| *BcucOBP10*-F-q | TGCCAGTTTCACAATACCGT |
| *BcucOBP10*-R-q | TAAGAAGCACATGCCCGTT |
| *BcucOBP13*-F-q | GTCATGGATGAGTCGGGCA |
| *BcucOBP13*-R-q | AGCGGTATCACAAGGCGTT |
| *BcucOBP15*-F-q | TGCTACAAAAATGCTCCCAA |
| *BcucOBP15*-R-q | CGGTTACACCATCTTCACTCAA |
| *BcucOBP18*-F-q | TGAAACCTATCCGCCACC |
| *BcucOBP18*-R-q | ACATCACCATCACTGAACTCCT |
| *BcucOBP21*-F-q | TTGCGGTATTCATTGCGTT |
| *BcucOBP21*-R-q | CCTTCGTGTCTGGAACTTTACCTTC |
| *BcucOBP23*-F-q | CTGGCGGGAACGGTAACT |
| *BcucOBP23*-R-q | CCTCCTGCATCTCAGGTGG |
| *BcucOBP24*-F-q | CCGAAGGTTTCAAAACACAA |
| *BcucOBP24*-R-q | GAATCCACGATAGGCCCA |
| *BcucOBP26*-F-q | GTGGTTTTACTTGCCGGTT |
| *BcucOBP26*-R-q | CATTCTTGTCACATCATCATCG |
| *BcucOBP27*-F-q | ACAAAGAGGAGGCCATCAA |
| *BcucOBP31*-R-q | ATCAGACAGGAGCGTAAGCA |
| *BcucOBP28*-F-q | TGGCTTTGGTTGCGGGTAA |
| *BcucOBP32*-R-q | TGGTGCGTTTGTGTGGTGG |
| *BcucOBP29*-F-q | AGCTCCTCAAAATTTGCCTG |
| *BcucOBP29*-R-q | CATCGCCTCAACATCATCG |
| *BcucOBP30*-F-q | CAAAGGGAAGGAGAATGGC |
| *BcucOBP30*-R-q | AGAGTACAGAGAAACAGCCGAA |
| *BcucOBP31*-F-q | ATTCTTCACCGTTGCCGTT |
| *BcucOBP31*-R-q | CACCTTTTGCTTTTGCTCTTC |
| *BcucOrco*-F-q | ACGTACTCTTCTGCTCGTGG |
| *BcucOrco*-R-q | TCAGTGGGTTCCTTCTCCTC |
| *BcucOR6*-F-q | CTTGGAAAGCGTCGAGAAA |
| *BcucOR6*-R-q | AGCGGTGAACAAATAGGTGA |
| *BcucOR7*-F-q | GTGGAGGAAGTTAAAAGAGACG |
| *BcucOR7*-R-q | TTCAAACGCTGTGAGGCAT |
| *BcucOR8*-F-q | AACGCAGGCAATCACACA |
| *BcucOR8*-R-q | GAATGAAGCAATTATGAACTCCGAG |
| *BcucOR9*-F-q | CTCGTTTCGGGTGTTCGT |
| *BcucOR9*-R-q | TGCTCAATTCGTCTGTCGTT |
| *BcucOR12*-F-q | TTAATTGGCTATTTCGGTCAA |
| *BcucOR12*-R-q | AAAACGATGGGTTCCTGC |
| *BcucOR13*-F-q | CTAACGAATGTGAACGAAGAAA |
| *BcucOR13*-R-q | AAACTAATCAAGGTGGCGAA |
| *BcucOR15*-F-q | ACATGTTTGTCGGGGCAT |
| *BcucOR15*-R-q | GATATACTCAAGAGTTCGCTGATGT |
| *BcucOR16*-F-q | TGCATCTCTAATGTTTCACTGG |
| *BcucOR16*-R-q | ATCTCGAACTCGCCCTCTT |
| *BcucOR17*-F-q | TTCGCAATACCCAACTCCA |
| *BcucOR17*-R-q | CGCCTTCGTTATCTCCCC |
| *BcucOR18*-F-q | TGCTTGGTACTTTGCCTCAT |
| *BcucOR18*-R-q | TGCCAGTGCTCAATTCTTTAA |
| *BcucOR19*-F-q | TCAAGTGGACACCGAACGA |
| *BcucOR19*-R-q | CGCAAATCCCCAAAATAATA |
| *BcucOR23-*F-q | TGCGGCAGTAAGAAGAAGG |
| *BcucOR23*-R-q | GCGAATATGATGAGTGAGGTG |
| *BcucOR25*-F-q | TATGTGCAAGGACGGGAGC |
| *BcucOR25*-R-q | GCGGTGAAGATGTAGGTGAG |
| *BcucOR27*-F-q | CTTGACGGAACGTGTGAGTC |
| *BcucOR27*-R-q | GCCAACGATCTTGGAAATC |
| *BcucOR28*-F-q | TAATGGAGGACAGTGCCAAG |
| *BcucOR28*-R-q | GAAGAGTTTAAGTGCGACGAA |
| *BcucOR29*-F-q | TGAGCGATGAGGAGAAACAC |
| *BcucOR29*-R-q | GTACAATGGCAACAACAGAGAA |
| *BcucOR30*-F-q | GACGGTCAATGTGGGTGCA |
| *BcucOR30*-R-q | ACTCGAAGTCGAAGGGGAA |
| *BcucOR35*-F-q | AAATTGTCAGCATTGTCGAGA |
| *BcucOR35*-R-q | CCAGCAAATAAGAGGCGTG |
| *BcucOR42*-F-q | TGGCACGAGAATACAAACAAG |
| *BcucOR42*-R-q | TACGGACAGTGGCAAGACG |
| *BcucOR45*-F-q | GAAGCAGATGATAAGGAAGACC |
| *BcucOR45*-R-q | TTCAGCATAGCGATGAGTAGAG |
| *BcucOR48*-F-q | AATATCAAGCAGTGGGACAAA |
| *BcucOR48*-R-q | CAACGCCAGCAAAGAAGA |
| *BcucOR55*-F-q | TAATTGTGGCAATGGCGT |
| *BcucOR55*-R-q | TCATAGAGCGGAGAGCGTG |
| *BcucOR60*-F-q | CGCTTACGATACCCCCTG |
| *BcucOR60*-R-q | AAATTGCTTGAAAATTCTCCA |
| *BcucOR62*-F-q | GACGGTCAATGTGGGTGCA |
| *BcucOR62*-R-q | ACTCGAAGTCGAAGGGGAA |
| *BcucRPL32*-F-q | ATCGTGAATACTGCGGTGAA |
| *BcucRPL32*-R-q | CGACTGTTGGGGTTGGTTAGA |
