# Supplementary material for: Identification of candidate chemosensory genes in Bactrocera cucurbitae based on antennal transcriptome analysis
Source: Front Physiol. 2024 Feb 19;15:1354530. doi: 10.3389/fphys.2024.1354530 (PMC10910661; doi:10.3389/fphys.2024.1354530)
Supplement: Supplementary file 5 [file DataSheet1.docx]

Supplementary Material S1: Amino acid sequences of *Bactrocera cucurbitae* and other insects used in phylogenetic

>BcucOBP1

MSLTMDEERHRDLLEARQQQLQQEQARPALTAAVLRSCMKETELSMAELHRFRLSLLSRDPEVLNGTNDPTTELPNLSVMEEFSEYSEIDGIVYEPEVEINASHDNALEYEDPFTFKSNENTDESLQCFAYCLYEQLGLISKGVYMEEELFAKLYAIVGRERHLVKECMNLNTNNKCESSYKMHLCYARLKTLEEENRLRKLMEDAYNGERDDEESEEIPQAETTQETYVMNEIGQTEST

FPVEEDAITENVGIKKKLKKLKKKSKRVDADAKFSE

>BcucOBP2

MISNVVYRAYIGFIILDIASILVTDAHGNFFTKVLHFIGSQNRCLNPPRTARRVESFIQECQEDVKNKLISEAYYILKNEVNEDYSALDINEENISGTKSPPTTSSAPSVIHTIEDNSNLESRHDVLYQFEPDKRHVTHLAQHFRRISYDPRSGIYHPTLVPAEEKRLAGCLLHCVYAKNNAIDKFGWPTLDGLVNFYSEGVNEHSFFMATLRSANLCLHAISIKYNINRRKLPKRGESCDLAFDVFDCISDQITGYCLNQYE

>BcucOBP3

MKCFVGLLLLLVLLGVHAYDFDDSAFNEYLFKELQSHYEGEELYTHRTRREATEAKECAKRSWKKDMQCCKNGNVNGDQWELFKSVKKQCIADLKGEPADQAYDPFDCEKMQQVKEQMICITECVAKRFKSLDENGELQRDAILEGLRGQIGTVQWKLDAIEGYVDKCLAEVKEKREQRQKTGQLKEGSCSRLPIAFHSCMWRQFWNGCPADLRVDSPKCNKMRERVADGDTRFFGKHFLNKYYPSPRDDD

>BcucOBP4

MALIYVLTVLSACLLAQSHAAPFNCTEPPNLGHFNIHSCCRMPEMDLGETPAKCVAYIKTLNAQARGMMNGMGGGNNKNNTNNNNNRNAAPAEFPAYAHICYPECIYRETGSLVENEFNIENVKKFLNKSVNKRDKDIVPQIVRSFESCLDNIKGHMAAMGIKTYAELPMGCSPLASMMYSCVNAETFLHCPAKMWKNEEQCNVAKSFAAQCNPLPHVPLPIG

>BcucOBP5

MSQASNMKANFGCFIVIFVCVCYASAEDESVDCTKPPRFIPLHMCCPVPDLSTEELMEQCAEFAKPPPPPPIGRGGPAKFEHSHHHPPHMRGLHPHPCLIECIFNKTEVIGENGEPDVDKFSALLDTTVKDNEEMAAIMEEAFEECTEKASELKAKIAEKISKYPEFAEKMANHRMQAACSPFGAMLMTCVNMETFKNCPASVWNDSTECNTVRDFINECKRV

>BcucOBP6

MDSKLIVFTALVLAAMVELPTFPGVEARSASIKMSFDMSVSEDQHRINPEMVRLCALETDLSMDELRKFNENDFSNATKNTQCFTHCLYEHMGVVRNGVFVERDLISLLGDVTDPKRMLERECLGQYSDNKCERAFLIHQCYRAGQRMMMPSNHQLEPVDQINEEDNAEEESTANLSKSDMAMTPAHRMAPNSERQLMIKKNLTKRLPKKQTINQGLEEE

>BcucOBP7

MKNLFVIIFACGLLLKFGNSLKIDCDNPESIKEDRIHYCCKHPDGYQEFVDSCAKETGFKFIKHDEEAIVDITADHAILGTCFAKCIFNKLQFMKGDDLDMPAVRKHYEGKFKTDPEYAKEMTNAFDHCHSKSIENTAKFLSNPIFHVPGAKYCDPKPSVILACVIREFFHNCPADRWAKTEECNTVLEFSKKCKDALTTI

>BcucOBP8

MANSSSALLIAGFICMMVVQSLDALNADVYKLSGKRKPLVTREDPTALEDYKRTKRQLPQPLQEFQDFITTSKTECAKEMNINPNELQKALLYEDQPTPKEKCMMECILKRMEVMNQDDTLSTSAVGRIADIIGDNNALITSIAMASAENCKKFITAENSCERAYQINKCIANEMKMRKIKLIY

>BcucOBP9

MSNCNAFVALPYMIMLLYCSVRMSMQDRAKNNGDIFVQHKEQRECVAPILVQTNDSISNEGADVLDMCNSSFSIPMDYIVQFNTNGALSETADKTGMCFLRCYFEKMGLIKAWQLNKDLIMQTMRPIKADSIEFCEPTAKQEVNACVRTYGIAKCLMKRGFDDTCNQTVAQNSFRAY

>BcucOBP10

MSELLEMVNYRKVELALSLLLLGVLAGTTADAQSDATDNKLDKQQSMETTTPMAEAANASGFDFQDVVRTCNASFTIPLEYIQRFNETAELPNTTDKTGMCFLKCYMEKTGLLRNWQLNSTLIRQTMWPATGDSIPVCQNEGSRETCPCKRTYAIAKCLMLRALVDARNKPIV

>BcucOBP11

MYQFGAHEKHATTITISSGKGMSLSGLTWSVFLVVFVVLVLPPAAVTLTPTAPSRSFMEACQVKHNVTLEELDEFPTDPNPDEIDMKFKCYADCLLNGMGFMDKNGKLDAEAMHEWGILDDESYDNMLECKAANDLEDDPCEYSFGMMLCARM

>BcucOBP12

MKYSIICCILATVLLSLCVFSADAGLRKPKKLTPELEAKFEVLTAWIAYRLNLKHAKEACVGEYGYSDELATNLVKIKVANPTDQQKCYVNCLYNKLVFYKDDAINKQAMKESLYEIVGEQRLMNIVNGCLNAGGTNACDKVYKFHACASPEFDKVRGDIFLPDE

>BcucOBP13

MKSFITIALLVVYSVVVLCHPNDPEMRKFIEDCNKEHNVSPKDFHDFMEGKLTTVPESLKCSSHCIMVKQGVMDESGNFKADVAKAKITEEKFAAAIDECKDLTGSTPCDTAMKITECMITHK

>BcucOBP14

MPQAMKYTAFIIIILFSTLIALINADHEETTEDDFLSAGQRCFQRERLAASYQRRFDNFEYPDEEPVHRYVHCIWTELKLWNDRTGFNVEHIAALYRDKANTEVLVPILSECNRNTQNEPTLRWCYKAFKCVLNSRVGQWFKEDVGRKLHERRVGNHVG

>BcucOBP15

MASADAKPPHYSALRVVAEAAIADCYEDSAQSVKVQITDEGFDEIVKGSRTNLTHNAKCIRYCIMRKNGLLNNDNSIDKENILQIFQIIHPQIEKDLLLNVLQKCSQEADKQANNCERAFVTSSCILQELSEDGVTDI

>BcucOBP16

MCCPEPDLSTKELMEQCAEYAGQSHPYQCLIECILNKTEVLGGNGEPDVDKFSALLDTTVKDNEEMAAIMEEAFETCTEKLSELKTKIAEKMSQNPEYAEKMANHRMQAGCLPYGAVLMNCVHTETFENCPASVWNDSTECNTVRDFIKECKHE

>BcucOBP17

MKFFIVILAVVALAYAEDDWVPKNAAEIKVIRQECFKDFPLSEEYIQKMKSFEYPDEEPVRKYLLCTAKKLGVFCEHQGYHADRVAKQFKMDLDEAEVLAIAEGCVDKNVEGSSADVWAYRGHKCLMASKIGDRVKAYIKKTVEEAKKQ

>BcucOBP18

MHALNSLLATLLWFGFLSNVIWAQKELRRDETYPPPELLKALQPVHDSCVAKIGVTEEAIKEFSDGDVHEDELLKCYMYCVFEETDVLHEDGEVHLEKILDSLPESMHVIALHMGKKCLYPKGDNKCERAFWLHRCWKEADPKVIYHKI

>BcucOBP19

MKNFIAFCLILAVTSAEYVVKNEENLQQYRRECATKLKVPEDHIQQFRKWQFPNDSVTQCYLKCVFEKFGLFNAETGFNVEYIHQQLQGAQVAPPGDADHDDVVHDKIAACVDSNEQGSNACEWAYRGGVCFIKENLQLVKHSVKQQA

>BcucOBP20

MLKTIIIKWAVVICVLINLCTKTTAIPISEQEEASHRLHKAHGICIQNITSPTSTDDDTSTRQLVGAYVRCIATNVGLWNDATGYNAKQVAKFFIKERNENEVMTLVDYCNQKHKQADLDLWAYEAYRCATAGRMGAWLSAYVRSAKL

>BcucOBP21

MSNKINLFVLVAVFIALVLRSDQVFGGATEEQMIAAGKLMRDVCLPKFNKITPEVADGIKEGKVPDTKDVKCYINCVMEMMQTMKKGKFLYESSLKQVDLLMPDSYKDEYRNGLGKCKDAAVGIKNNCDASYALLICMRDNITKFVFP

>BcucOBP22

MHFCKVFLTTSVLAFLFATPISAGVTEEQMWATAKLMRDVCLPRFPKISIELANQLRDGNIPDDNKDVKCYINCVLEMMQTMKKGKFLYDASLKQVDLVLPDSYKDDYRGGLLKCKDAAVGIKKDNCEAAYTILKCLRGEIKKFIFP

>BcucOBP23

MLVKINAFKYFLTLLACTGVNAITMQQFESSLDMMRNGCAPKFKVATEILDNLRAGEFIENNGELKCYTRCVAQLAGTVTKKGEFSVQKALAQIPIILPPEMQEAAKAALNACKDVQKSYKDSCERVFYTTKCVRDYDPATFKFP

>BcucOBP24

MKYIVAVLLAALVAMAAAEEYKIRNQDDLLKARKECMEAKKVPTEHIEKFKKFEFPDDEVTRCYIECIFNKFQLFSPTEGFKTQNLIAQLGQNKENKDAVKADIEKCADKNEQKSDSCTWAYRGFKCFISKNLPLVQESLKKN

>BcucOBP25

MKYFVVSLVICSLVMSYSEAQELPMRLKKIVEECKARLGAGDDDVAAMFKYEPATNKQVKCLHACTMRLLGILDKNNKPFEAGAMAYIKSVTASNAELEKLLTEVYNECKYFPASKEGCEFSEAFRVCIIERSRAKGIHMLLH

>BcucOBP26

MKYFVVCLAICSLVISHTEAQELLEKIKQIAEECKGQVGASDDDVTRMLKYEPATNDKAKCLQACIMKQFGILDDNNKLVEAGAMAYIKSLAAGDAELEKLSTEVYNECKNTPANSNECEYAEAVRACTIENSKSKGIKILPQ

>BcucOBP27

MAKFILIAALCILSAAVTKAAFNKEEAIKTFMTRAEECRGEVGAADSDIQDLIAKVPAAGKEGKCLRSCLMKKYGAMDGNGKFVKSVADEHAQTYTDGDADKLKIAREIIDACADIAVPADHCEATEVYGKCFMEQAKAHGIEKFEF

>BcucOBP28

MKTQLILLLACVALVAGKFQIRTAQDALAAHEACHDEFRIPEDIYEKYLNYEFPPHKRTNCYVKCFVERMGLFTEEKGFDEKAIIAQFTAKSSKNLAKVSHGLEKCIDHNEHDSDTCTWANRVFSCWISVNRPIVRKTYIEN

>BcucOBP29

MKLLKICLILCVALISNAKCGSEEAKAAAEECKEEVGATDDDVEAMFKFKSAGSMEAKCLHACVMKRFGLINGDGKMDRDKAIEILENIASGDNEQQALGVEIVEACEGIEVDEDHCEAAEEYRSCMHDKGKENGFKMGRV

>BcucOBP30

MKLLKIFLIVCVALISNAKCSFEEVKAAAEECKEEVGATDDDVAAMFNFESAGSMEAKCLHACVMKRFGLMNGDGKMDRDIAIEILENIASGDEAQQALGVEVVEACEGIEVDEDHCEAAEEYRSCMHDKGKENGFKMGRV

>BcucOBP31

MKFFTVAVVLAFVAVAAAQDGGLKLSEEQKQKVHTLGAECLKETGASEEAVRAVGKGDFSQVDGNVKCFAKCLQGKLGYFVDGKVNEAAVQSSLGKLVGEEKIKAIQAKCNGLKGTDDCDTAFQLHKCYATENASILV

>BcucOBP32

MKALFILVILTVFGSITMQQYSRRELINIYELACMADTNVTESEFRKFITNGMKANEATSDIKCMIMCVMEKRGILKKGTFDTERAYKEIMIVGELKGHEDEIKEAVHNCKAEKGCHDCDTAFKITMCLQEFSSRNK

>BcucOBP33

MRTISLCFIAALLVAAQAQMTAKEYVEICNKEHGVTNTELKQFIDSKMDPAKATNPIKCHMKCVIEKLGFYKNNMLDEALAAKFFKEHNKDQQANVETIKQTIQKCNQMKGADPCDTAYQIVTCFVKSELPMFAV

>BcucOBP34

MHTFYILVTMAALVTLAVCQFPADMEKFHKACMDEAKVTEEELKQFFQNGMKADEAKENIKCHTKCLMQKQGIWKDGVFNADAKVKELMQMPKLKDHEAEITQAMNNCKNEKGANECDTAFKITMCLKEFRSKNP

>BcucOBP35

MKSNILLAILIALSAVVAAHKSHKPSQAHQFYEECLKESGASPALLDPLKKGDFNAVGDKAKCFLKCLENKKGILENGAPNEADIRKVRKVGNEEPKNILAKCNGLMGANECDTAFLLYKCYWQEHAALI

>CcapOBP99a-like1

MKYFILILAAVVLAQAQDDWKIKSANEVNDIRRECHKEHPFNEELQKHEEVLRFPDEDVVRNYEVCVFTKWGVFDPETGFKKDRLVRQFEPVLKREEIDEIIGRCADKNEQGSPVDVWVYRFQQCVSRSEIPPNFLKIIGKL

>CcapOBP19d.1

MEIPKFFFIFAIIITGSKMTLVVADSKPPHYSSLRLMAEDAIGDCYEDAARSVKVEITDEGFDELLKGSRDNLSRNAKCLRYCIMRKNGLLNELNSVDEEKIIQIFQIIHPQIEREKLLSVIRKCSEETEKQTDNCERAFLAAKCILLELKEDGVTDI

>CcapOBP99a-like2

MKYFIVILAAVVLAQAADDDWVPKTPEEFNAIRRECHKEFPFSKELQKQEDELDFSDDETVRKYEVCVFRKWGIIDADDTFHGERLVKQFEAVLDGVEGIEQKVNNCVDKNEQGSPIDVYASRIQQCIDKTDIAPKLLKVIGKL

>CcapOBP56h

MQKIYLLTIMAALASVAVSQLPADVEKYHRACLDESKVSDDELKQFFQNGMRASDATENIKCHNKCMMQKMGIFKDGVFDADAKMKELMQNPSMKGHEAEIKQALNDCKNEKGANECDTSFKITMCLKDFSKPQHA

>CcapOBP99a-like3

MKYFFLILAAFLFAQAQDDWKIKSANEVNDIRRECHKEHPFNEELQKHEEVLRFPDEDVVRNYEVCVFTKWGVFDAETGFKKDRLVRQFEPVLKREEIDEIIGRCADKNEQGSPVDVWVYRFQQCVSRSEIPPNFLKIIGKL

>CcapOBP99a-like4

MKYFILLLAAVLLAQAEGEWKVKTVDEFNDMRRDCHKEIPFSEELQKKEEVLRFPDEEVVRKYLLCIAKTWEVFDEENGFNMDRLIVEFELLLNRDEVLPIAKRCVDKNEQGSSPDVWVYRVQQCVGKTKLGPLFLEALGKLSK

>CcapOBPA10

MFRLIVALALILSIYHAPSVHGAPHPPTTAAPLVANQGSYDTKFDNIDLDEVLSQERLLRNYIKCLENLGPCTPDSKMLKGFAHFRDTPRRSINRLRQMLGEAEAWLSEGDAFLDRQSSGGLGTLGADIRSIRQLPFSLFSGEGQRQQPERHWRADSFGSGY

>CcapOBP56a

MKSFGLLQSFLVILIIGPISELLAADDKTARESCIKEVNLSAADANSVRGTAMISKLVQNNSESLKCFQLCYYKQLGFLDASGKTNGQKVLEYMSQASGITDTAKLVAALSGCESVNGASQCDKVYQFEKCALGKLGV

>CcapOBPA5

MLHAINRYLLIFLAVNSVSAEDTEAERLFRDLEIVPDVLDEPPKELLKIEYSNGLNVVGGEELTPTETKDEPKIYWPADPATYYTVVMTNPDIPTRQNPATREWLHWLVVNIPGTDIAQGYVIDPYIGPLNPKESGLVRNVFLIYKQPEKQVFDEPLINNTNVAGHERFSTMNFAKKYKMELVAGNVFQSRWDEYVPLIHKQFGISV

>CcapOBP69a

MNYRRLVILLIIYQCVLLRNVQSLEVPKHMRSGAKKLTNLCIKETGVTEDLFIEAQETGKMPNNQRLKCFIHCVLDKIGLIDADNIVHLDNLLEILPPEFVPIVEELHTTCGTQSGADGCETAFLTTECYIKTNPVILKLLFTTFSE

>CcapOBP83a

MYILRTILGALLWCSVLLNLIWAQKELRRDETYPPPELLEALRPVHDKCVAKTGVTEEAIKEFSDGEIHEDEPLKCYMYCVFEETDVLHEDGEVHLEKLLDSLPNSMHNIALHMGKKCLYPKGDTKCERAFWLHRCWKESDPKHYFLI

>CcapOBP19d-like

MKLILTFLLVCGLIISNTEANDLVDKVTKLAQECKGSTGASDADYENLLNYQAPNTDKGKCLTECVLKKSGALGENNKINEAVMVEFIKAGAAGDASIEKIGMEGLQACLKVPQHPNACENAAALNACFTKNLRAKGFKGLPA

>CcapOBP56h-like

MKSYFALTLFVISCAVVMCHPPPDPEMRKYIDECTKDHNVTPKEFHEFMEGKAASPSENLKCSSHCVMLKQGIIDEAGNFKPDVAKSKMPDDKLAAAIDDCKDLSGSSACDTAFKITECVLSHK

>CcapOBP19a-like

MWATAKLMRDVCLPRFPKIATELADQLRDGNIPDNNKDVKCYINCVLEMMQTMKKGKFLYEASLKQVDLILPDSYKDDYRAGLLKCKDASAHIKKDNCESAYTVLKCLRGEIKKFIFP

>CcapOBP84a

MGITCKLLKKTMLDHRHLNIILLLLQFGIMSSGAADAQTNLTNNASDQLQAVENTTPMVEEEHEMGFDFDAVVRTCNASFAIPLELIQRFNETAELPNTTDKTGMCFLKCYMEGTGLLRNWQLNRSLIRQTMWPATGDSIPVCQEEGSRESCPCKRTYAIAKCLMIRALVDARNKPIV

>CcapOBP28a

MAKFILIAALCILSAALTKAAFNKEEAIKTFMTRAEECRGEAGAADSDIQDLITKSPAAGKEGKCMRSCIMKKYGVMDSNGKFVKSVAMEHTKTYTDGDADKLKIANEIIDACADIAVPDDHCEATELYGKCFMEQTKAHGIDKFEF

>CcapOBP71

MLKRSSASRAGHLGVTLLLFYFSTCCSLKCRTEDGPSESELKRITRSCMRKIGESLRPSNGGSQGYSNNYNHYGPLHQSNFGGQYGGNSRYDYNYDYDDDNDQYNGNGNSNNYNSNNNNGNNNNGNNNYDRDHDRGNRNVLQQRNRDRQQNGNSDNSRSSSSSSSSSGNNHGNGGRYDNNNGKSNGGNGGSGGGSGNNGGGGGSRNTGSGGSSGSGQRGHTQNHSNNNNNNNNSNNSNNNNRSANNDTADASCVVHCFFEELNMLNSDDYPDRYKVQYGLTRDLRDRELRNFYTDTIQDCFHYLESQRRRDKCHYSRDLINCMTEYAKVNCDDWQEFNMVFN

>CcapOBP56d

MKFFTVAIVLAFVAAAAAQELIKLTDEQKQKVHALGAECIKETGAAEEAVRAAGKGDYSQVDGKVKCFAKCLQGKLGYVEDGKLNEAAIQASLGKIVGDEQIKAIQAKCNGVKGADDCDTAFELHKCYLAENANIQI

>CcapOBP99a

MKFLIVVLAVVALAYADDEWTVKQAAEIKEIRQECLKENPLGDEYIQKMKSFEYPDEEPVRKYLLCTAKKLGIFCVHEGYHVDRIAKQFKMDLDEAEVVAIAEGCVDKNEQGSSADVWAYRGHKCLMASKIGDRVKSYIKKNVEEAKKQ

>CcapOBP99a.1

MTEAMKCSTIAFTWIIILLFNFCNFIGADFEEKTEEDFLTASERCFQRERLAASYQRQFDNFVYPDEAPVHRYVHCIWNELKLWNDRTGFNVEHIAALYRDKANTEVLVPILSDCNRNTNNAPTLKWCYNAFKCVLNSRVGQWFKEDVERKLHERRTGNHVA

>CcapOBP99a.2

MKYIVAVLLALFALAAAEEYKLRNQDDLLKARKECMEAKKVPAEHIEKYKKFEFPDDEVTRCYIECIFNKFQLFSPTEGFKTQNLIAQLGHNKENKDAVKADIEKCADKNEQKSDSCTWAYRGFKCFISKNLPLVQESLKKN

>CcapOBP99a.3

MKFCLALLSLFFAVVVADHGDHSDYVVKTGEDLARYRDQCVAKLSIPADLVEKYKKWEYPDDEKSRCYLKCVLESFGLFDDAKGFDVHKVHHQLGGGDVDHSNELHGKIENCAKEGDAAGEDACTRAYRGALCFFKENLALVKQNVASK

>CcapOBP19a

MLPRMNLFVLPAVLIVLTLNTDRVYGGATEEQMIAAGKLMRDVCLPKFKVPTDVADGIKDGIVPETKEVKCYINCILEMMQTMKKGKFLYESSLKQVDLLMPDDYKDQYKSGFAACKDSPNGIKNNCDASYALLICMRDKITKFVFP

>CcapOBP99a.4

MKIQLILLLACVALVAGKFELRTAQDALAAHEACRDEFRIPDDIYEKYLNYEFPPHKRTNCYVKCFVERMGLFTEEKGFDEKAIIAQFTAKNSKNLAKVSHGLEKCLDHNEHDSDTCTWANRVFSCWISVNRPIVRKTYIAN

>CcapOBP57c

MFQFGEVGKNTTTKSEMMLASNGRGMSLPGLTWLVLLAAILMFFLPIDKVAASPTASTRSFMEACQVKHNITMEELDEFPDEPDADEVEMKFKCYAHCLLFGMGLLDENGKLNVEYMHDVGILTDPSYESMLECKAANDMEDDPCEYSFGMMLCARMLGTEEGGSEENIEDIEEEVEAKEEEERRK

>CcapOBP56a.1

MKSFIVCCMLATTLLSFCVLSTEAGLRKPKKLTPELEAKFEVLTAWIAYRLNLKHAKEACIGEYGFTDELATNLVKIKVANPSANEKCYVNCLYNKLVFYKDSTINKQAMKESLYEIVGEERLMNIVNSCMNAGGANDCDKVYKFHACASPEFDKVRSDIFLPDE

>CcapOBP56a-like

MKSSIICCILATVVLSFCVLSTEAGLRKPKKLTPELEAKFEVLTAWIAYRLNLKHAKEACIGEYGFTDELATNLVKIKVANPSANEKCYVNCLYNKLVFYKDSTINKQAMKESLYEIVGEERLMNIVNSCMNAGGANDCDKVYKFHACASPEFDKVRSDIFLPDE

>CcapOBP19d.2

MYSNKTLLFASLICLLAVESINALNANSYKLSGKRKPLATREDATIIEDYKRTKRQLSQPMQEFQAFITTSKGQCASEMGFKANEMEKSLLYEELPTPKEKCMMECILKRMEVMDRDNMLSTPAIGRIADIIGNNNALITSIAMASADNCKKFITAEDPCERAYQINKCIATEMKMRKIKLIY

>CcapOBP68

MGNLAIIIFAFGLALKPTSSLKIDCDNPDSIQEDHIHYCCKHPDGYQEFIDACVKETGFQYGKPEEEAMVDITVDRAITGTCFGKCVFSKLEFLKDNELDMSAVRKHFEDKFKTDPEYAREMINAFDHCHDKSVEHTTKFLSNPLFRATNGEFCDPRSSVILACVIREFFHNCPADRWSKTEECSTVLEFSKKCKDALTTI

>CcapOBP56d-like

MNSNIFLITFLVLFALAAANELKSHKPARNHKIYEDCMKESGASVAQLEALKKGDFNSIDNKAKCFLKCLEDNKGILTNGMPNEAGIRKKVHAPPAGNGVSKDLLAKCNGLKGADECDTAYQIYKCFMQEKVPLI

>CcapOBPLush

MQEKYKAMILNTNGIKYFFTLLACASVGAVTMQQFEASLDMMRNGCAPKFKVSVDILDKLRAGEFTENNNDLRCYTRCIAQLAGTLTKKGDFSVQKALAQIPIILPPEMQDAAKEALNACKDVQKNYKESCDRVFYTTKCVRDYDPTTFKFP

>BcorOBP99d

MFIPRFLIIPITIVFCVLISFPMRVKAVPISEHEEASRRLYKAHGFCVQNSSTPTSIDKEYRSTKQEPAAYVRCVASNMGLWTDASGYQAKRVAKFFIKEHNENEVMTVVDYCNQKHQQADLDLWAYEAYRCATAGRMGIWLRAYVVSAKL

>BcorOBP99c.2

MKYFMCIVILAVVALVQADDWSPKTVEEIKSIREECMKQVPSTDEEFQKRKENDYPDVESVRKYALCNSKGWGLYKEGKGFYADRVAEQFKDDMPEDEIKTIVHDCDEKTKEDTDDERCYHLLKCVMTTKLGDHVKSLIKRSE

>BcorOBP99c.1c

MKFLLVIFAVVSQIYAEDEWTPKNMAELNVIRQECLKEFPLSEEYIQKIKNFEYSDEEPARKVLLCTVKKLGVFCEREGYNADRVAKQFKMDLDEAEALAIVEGCLDKNLEGSSAAVWAYRGHECVMASKLGDRVKAYFLKSGEELKK

>BcorOBP99c.1a

MKFFIVILAVVALAYADEEWVPKNVAQIKAIRQECIKDFPLSEEYIQKMKNFEYPDEEPVRKYLLCTAKKLGVFCEHEGYHADRVAKQFKMDLDEAEVLAIAEGCADKNVEGSSADVWAYRGHKCVMASKIGERVKAYIQKSVEEAKKH

>BcorOBP99b

MKFCLALLSLLMVVVFAVADHADHTDYVVKTKEDLIRYRDECVSKLSIPSDLVDKYKEWSFPDDEKTHCYLKCILEKFELFDAAKGFDAHKIHHQLQGANADHSDATHGAIENCAKEASENSDDACVRAYRGFTCFLKNNAQLVQAGVEKSSK

>BcorOBP84a.2

MINHRLLCLALSLVLLGFLAGARAHPETDATDNKLDKQQSMETTTPSAGAANETGFDFQEVVRTCNASYTIPLEYIQQFNETAELPNITDKTGMCFLRCYMEKTGLLRDWQLNPTLIRQTMWPATGDSLPVCQNEGSRETCPCKRTYAIAKCLMLRALVDARNKPLV

>BcorOBP84a.1

MSNDNVFVMLPLMVILLCCSIMVSVQDRAKDNGDIFVQHNEQRECVAPIIVQANGSVSSEGMDVAHICNNSFSIPSDYIVQFNRNGDLPETVDKTGMCFIRCYFEKAGLIKNWQLNKDLIMQTMWPIKADSIAICEPEAKQEMNACVRSYAIAKCLMERGFQDTCNDTVA

>BcorOBP83g

MNTQLILLLACVALVAGKFQIRTAQDALDAHEACHEEYRVPEDIYQKFLNYEFPAHKRTNCYVKCFVERMGLFTEEKGFDEKAIIAQFTAKSSKNLAKVSHGLEKCLDHNEHDSDTCTWANRVFSCWISVNRPIVRRTYIEN

>BcorOBP83ef

MNFREASQVLLLIAVAYCIQMVSSAATTKATEQDVDSDTEILRKCLREVGSKDLVGELQKVARYSKWTSEEVPCFTRCLASMKHWFDADESKWNKQQIADDLGADMYNYCRYELDRYNEDSCEFAYTGLRCLKQAELYTLETYKNIVSCASELNVTMKELQKYAAFPTKEVVPCLFQCLAEKMNFYTPTYEWNFDNWVQAFGPMRQDRTASNVCKVSAEQIKTRDKCQWMYEEYNCLERLNYNTDGSYPLESTTLSAV

>BcorOBP83b

MHSGKTLLGTLLWIGFLLNLIWAQKELRRDETYPPPELLKELRPVHDSCVAKTGVTEEAIKEFSDGDVHEDELLKCYMYCVFEETDVLHEDGEVHLEKILDKLPESMHVIALHMGKKCLYPKGDNKCERAFWLHRCWKEADPKHYFLI

>BcorOBP83a

MVLTGIRRGQAFHAFLIVALLSSLTLMHVQAQEPRRDDKWPPPAVLKMAKIFHDICVEKTGVTEEAIKEFSDGQIHEDEALKCYMNCLFHEIDVVDDNGDVHLETLFNTVPGTVRNQLINMAKECEHPEGDTLCHKAWWFHQCWKKADPVHYFLP

>BcorOBP69a

MNTKQFVFLVIIYQYTFYTGVTTLEVPKHMVSGVKKLTNICIKESGVSEDLFKDIRATGELPNNQNLKCFMHCVLDKIGLIDGDNIVHLDNLIEIMPPEFVPIIERLHTTCGTKSGADGCETAFLTIECYIKKEPIISKMLFSTFED

>BcorOBP59a

MSKRSLACLSFVPLWISVFFLYCSVCYALKCRTDDGPSESELKRITRNCMRKIGENVHSNGGGSMGSNNHQNHPYGSLHQSNFGSQHGGNSRYDYNYDYDDNADQYYGNNPNNNNYNNNNYNNNNGNHDRDRNVLQQRNRDRQQNNRSDNNRSASSASSNNNGGRYDNNDGNGNRGGGGGNGDGGSRGGNGGANSGGRNSGGNGGSGNSQRGFNQNNSNNNNNGNNRNGKNDTADVACVVHCFFDELNMLNSDDYPDRYKVQYGLTRDLRDRELRNFYTDTIHDCFQYLESQRRRDKCHYSRDLINCMTEYAKVNCDDWQEFNVVFN

>BcorOBP57c

MYQFGAHEKRATTAATMNATVLAVGGGKEMSIPGLTWLVLLAVIVVFALPAGAVALTPTAPTRSFVEACQVKHNITLQELDEFPTDPSPEDIDMKFKCYADCLLNGMGFMDTNGKLDAEGLHEWGILNDESYENMLECKAANDMEDDPCEYSFGMMLCARMLNSEEENYYSDAVDDVAEERRRK

>BcorOBP57h

MQKFYILTIVAALVTLAVCQLPADMEKFHKACLDEAKVTDEQMKQFFQNGMKASDATENIKCQLKCMMQKQGIWKDGVFDADAKIKDLVQNPKFKGTEADLRKAINNCKNEKGANECDTVFKISMCIKEFLV

>BcorOBP56g

MKTFVTIALLVVGSAVVLCNPHDPEMRAYIEECNKEHNVSPKDFHDFIEGKLTTVPDNIKCSNQCLMMKQGIMDESGNFKPDVAKAKVKDDKLAAAVDECKGLSGSTPCETAFKITSCMLSKN

>BcorOBP56d

MKFFAVAVLLAFVAVVAAQEGVGKLTEEQKQKVHAAASECLKETGASEEAIHALLKGDDSQVDGKVKCFAKCTLGKLDLVQNGKVNEEKVQNVLGKLIGEEKAKAAQAKCNGLKGTDECDTAYQIRQCYSAGHESFIF

>BcorOBP56c

MYYTLSLLAVLLCATVQNAKSRTITISMNMSLIMNVERRKELLETNQQQLQQEQARTALTAAVLRSCMKETELSMAELHRFRLSLLTRDPELNSTDDMSTELPNLYDMEEDDLVSEETELDGIIYEPQPDINEANKNALDYEVPLTIRSDGKTDESLQCFAYCLYEQLGLISKGVYMDEELFAKLYAIVGRERHLVKECMNLNTNNKCESLYKMHLCYARLKTLEEENRIRKVMVSAYPGERDEAGGEEIPEVETTQETYAVEETETTQPTYTVDENAKIEDFGVKKLMKQLKKKLKLMDADEQLRELLQNWNEA

>BcorOBP56a

MKSSIICCILATVLLSLCVFNADAGLRKPKKLTPELESKFEVLTAWIAYRLNLKHAKEACVGEYGFSDELATNLVKIKVANPSDREKCYVNCLYNKLVFYKDDAINKQAMKESLFEIVGEQRLLNIVNGCLNAGGTNACDKVYKFHACASPEFDKVRSDIFLPDE

>BcorOBP50e.2

MIPLFILLSTWLLTEKCYAEQNNQTDCSRLPKSIAPHICCRFPEPFEGQILDECHRLHNEFGQCFVECLFGRADICRHGKCSYQRANKYLDNKFVLQQTIFKDIYKSAFKKCIEKGNGLLVPLIERFRRNGCHPVPELIRFCVRNEMFISCPNGYWNEKLMRCTKKQQFIRHCTKEN

>BcorOBP50e.1

MAFAYILTVLTACVFAHTHAAAFNCSEPPNLGHFDVHSCCRMPEVDLGETPAKCAAHIKTLNAQARNMMNGMGGNNNNNNNTNNNNKMNNPAAEFPAYAHICYPDCIYRETGALVENEFNMENVKKFLNKNVNKRDKDIIPQIARSFQSCLDYIKGHMAAVGIKSYAKLPMGCSPLASMMYSCVNAETFLHCPAKMWKNEEPCNVAKSFAAQCNPLPHVPLPIG

>BcorOBP50c

MKCFLVFLLLFVLLCVNAYDFDDSAFNEYLFKELQSHYEEDEVSHRTRREATDANECSKRNWKKDMQCCKGGNVNGDQLELFKSVKKQCIADLKGEPVDDAVDPFDCEKMQQVKEKMICITECVAKNFKSLDEHGELQREAILEGLRGQIGTVQWKLDAIEGYVDTCLAEVKEKREQKQKAGELKEEGCSRSPLVFHSCMWRQFWNGCPAELRVDSPKCNKLRERVANGDTRFFGKHFLHKYYPNPHDEE

>BcorOBP49a

MKAYFGYFVVIFVFVCHASADDETVDCTKPPRFVPPHMCCPVPDVSTDELKKQCAEYNKPPPPPPMGRGGPPKFDRPHHMHHPPPCVIDCIFNNTEVMGANGEPDVDKFSALLDTAVQDNEEMAAVMEESFETCVGMLSELKAKMAEKASKHPEFAGRMGNCSPVSGMLMMCVNMETFKNCPASAWNDSTECNAARNFFKQCKFPKDGN

>BcorOBP44a

MKYIVAVLLAALVAMAAAEEYKIRNQDDLLKARKECMEAKKVPTEHIEKFKKFEFPDDEVTRCYIECIFNKFQLFSPTEGFKTQNLIAQLGQNKENKDAVKADIEKCADKNEQKSDSCTWAYRGFKCFISKNLPLVQESLKKN

>BcorOBP28a

MAKFILFAALCILSAAVSKAAFNKEEAVKNFMTKAEECRGEVGAADSDIQDIIAKVPAASKEGKCLRSCLMKKYGAMDSNGKFVKSVADQHAQDFTDGDADKLKTAREIIDACADIAVPDDHCEATEVYGKCFMEQAKAHGIQKFDF

>BcorOBP19d.3

MKYFVVFLAICSLAISYTEADEFGEKAKKIAEECKAQVGASDDDVARMFKYEPSANDKAKCLNACTLKKLGTLDENNKVVDSAVVAYVKQVSGGDAEFEKLSMEIYSECKNTPENSNECEFAEAFRQCVLDGAKSKGVKILPQV

>BcorOBP19d.2

MKYFVVCLAICSLVISHTEAQELLEKIKQIAEECKGQVGASDDDVTRMLKYEPATNDKAKCLQACIMKQFGILDDNNKLVEAGAMAYIKSLAAGDAELEKLSTEVYNECKNTPANSNECEYAE

>BcorOBP19d.1

MKILYICLIVCAALISNAKCDHEKAKAVANECKEEVGATDDEVESFLKFEAAETMTAKCLGACVIKRFGLMNGDGKIDREKSIEILEIIANGNEEQQALGVEVLDACADIDVNEDHCEAAEEYRSCMHAKAKEIGFEMGRV

>BcorOBP19c

MVKSSSALLIVGFICLMSLQSLTALSEDADKLSEKRKPLMTREDPSTLEDYKRTKRQLPQPLQEFQDFVTTSKTQCAKEMNINPNELQKSLLYEDQPTSIEKCMMECVLKRIEVMSKDDTLSTTAIGHIADIIGDNNALITSIAMASAENCKKFITAEDSCERAFQINKCIAAEMKMRKIKLIY

>BcorOBP19b.2

MAEAAIEDCYEDSAQSVKVQITDESFDEILKGSRTNLSHNAKCLRYCIMRKNGLLSVDNSIDEENILQIFEIIHPQIKKDSLLDVLHKCSREMNKQTDNCERAFVATSCILRELQADGVTDI

>BcorOBP19b.1

MNKVLTLFAAVVLMIATRKVLAEEIMKVPMGLALETIEPFAMNCELNPEKAHMEDLILNKEDADLTTKCLRSCLMEQFELFVEDSTDVNTEKLLSWMVLLYSEKIDELRAISNGCNEKNVEMGITDKCEVAHSFAMCMLKEMKEREYEIPEVEQ

>BcorOBP19a

MLNKINSFVLATVFIALVLHSDQVSGGATEEQMISAGKLMRDVCLPKFNKVSPEVADGIKEGNVPDTKDVKCYINCIMEMMQTMKKGKFLYESALKQIDLLMPDNYKDDYRNGLAKCKDVTSGIKNNCDASYTLLICMRDNISKFLFP

>BcorOBP8a

MPQAMKCTAFVFIILFAALFAHINADYEEKTEDDFLSAGERCFQRERLAASYQRRFDNFDYPDEEPVQRYVHCIWTELKLWNDRTGFNVEHIAALYRDKANTEVLVPILSDCNRNAQNEPTLKWCYRAFKCVLNSRVGQWFKEDVGRKLEERRVGNHVA

>BcorOBPLush

MCLKINVFKYFLTLLACTGVSAVTMQQFETSLDMMRNGCAPKFKIATEILDNLRAGEFVENNGDLKCYTRCIAQLAGTVTKKGDFSVQKALAQIPIILPPEMQGPAKEALNACKDVQKNYKESCDKVFYTTKCVRDFDPATFKFP

>DmelOBP99b

MKVLIVLLLGLAFVLADHHHHHHDYVVKTHEDLTNYRTQCVEKVHASEELVEKYKKWQYPDDAVTHCYLECIFQKFGFYDTEHGFDVHKIHIQLAGPGVEVHESDEVHQKIAHCAETHSKEGDSCSKAYHAGMCFMNSNLQLVQHSVKV

>DmelOBP99a

MKVFVAICVLIGLASADYVVKNRHDMLAYRDECVKELAVPVDLVEKYQKWEYPNDAKTQCYIKCVFTKWGLFDVQSGFNVENIHQQLVGNHADHNEAFHASLAACVDKNEQGSNACEWAYRGATCLLKENLAQIQKSLAPKA

>DmelOBP56e

MKVFFVFAALAALSLASAVGLTDSQKAEAKQRAKACVKQEGITKEQAIALRSGNFADSDPKVKCFANCFLEQTGLVANGQIKPDVVLAKLGPIAGEANVKEVQAKCDSTKGADKCDTSYLLYKCYYENHAQF

>DmelOBP19c

MKPSTPVAAIPLMTIVVAVLLQTHCVRGQTQAFDLAKLLPKTGTEPIWAVIDRNLPQVQELVTAARMECIQKLQLPRDQRPLGKVTNPSEKEKCLVECVLKKIKLMDADNKLNVGQVEKLTSLVTQDNKMAIAVSSSMAQACSRGISSKNPCEVAHLFNQCISRQLERNNVKLVW

>DmelOBP58b

MLRIGFVICVIISLRLNGLVAVRVHCRHMERIHEENIHHCCKHQDGHDDVTESCAKQTNFRLPSPNEEAIVDVTVDQAMVGTCWAKCVFDHYNLMENNTLDMDKVRSYYKRYHQTDPEYATEMLNAYEKCHTQSEEATEKFLSLPIVRAFSTAKFCKPTSSIIMSCVIYNFFHNCPASRWSNTTECVETLAFARKCKDVLTTM

>DmelOBP83a

MALNGFGRRVSASVLLIALSLLSGALILPPAAAQRDENYPPPGILKMAKPFHDACVEKTGVTEAAIKEFSDGEIHEDEKLKCYMNCFFHEIEVVDDNGDVHLEKLFATVPLSMRDKLMEMSKGCVHPEGDTLCHKAWWFHQCWKKADPKHYFLP

>DmelOBP44a

MKNAVAILLCALLGLASASDYKLRTAEDLQSARKECAASSKVTEALIAKYKTFDYPDDDITRNYIQCIFVKFDLFDEAKGFKVENLVAQLGQGKEDKAALKADIEKCADKNEQKSPANEWAFRGFKCFLGKNLPLVQAAVQKN

>DmelOBP28a

MQSTPIILVAIVLLGAALVRAFDEKEALAKLMESAESCMPEVGATDADLQEMVKKQPASTYAGKCLRACVMKNIGILDANGKLDTEAGHEKAKQYTGNDPAKLKIALEIGDTCAAITVPDDHCEAAEAYGTCFRGEAKKHGLL

>DmelOBP57e

MLDQLTLCLLLNFLCANVLANTSVFNPCVSQNELSEYEAHQVMENWPVPPIDRAYKCFLTCVLLDLGLIDERGNVQIDKYMKSGVVDWQWVAIELVTCRIEFSDERDLCELSYGIFNCFKDVKLAAEKYVSISNAK

>DmelOBP56h

MKFTLFCIALAAFLSMGQCNPDFRQIMQQCMETNQVTEADLKEFMASGMQSSAKENLKCYTKCLMEKQGHLTNGQFNAQAMLDTLKNVPQIKDKMDEISSGVNACKDIKGTNDCDTAFKVTMCLKEHKAIPGHH

>DmelOBP57c

MLKLWLICILTVSVVSIQSLSLLEETNYVSDCLASNNISQAEFQELIDRNSSEEDDLENTDRRYKCFIHCLAEKGNLLDTNGYLDVDKIDQIEPVSDELREILYDCKKIYDEEEDHCEYAFKMVTCLTESFEQSDEVTEAGKNTNKLNE

>DmelOBP56d

MKFLIVLSVILAISAAELQLSDEQKAVAHANGALCAQQEGITKDQAIALRNGNFDDSDPKVKCFANCFLEKIGFLINGEVQPDVVLAKLGPLAGEDAVKAVQAKCDATKGADKCDTAYQLFECYYKNRAHI

>DmelOBP84a

MFHSLYLIGILSLIWVAAQDIVPDDPEVQMQMHAMFYTARVACADENLIPYVRACAVIAFLILSPNCARALQDHAKDNGDIFIINYDSFDGDVDDISTTTSAPREADYVDFDEVNRNCNASFITSMTNVLQFNNTGDLPDDKDKVTSMCYFHCFFEKSGLMTDYKLNTDLVRKYVWPATGDSVEACEAEGKDETNACMRGYAIVKCVFTRALTDARNKPTV

>DmelOBP19d

MSHLVHLTVLLLVGILCLGATSAKPHEEINRDHAAELANECKAETGATDEDVEQLMSHDLPERHEAKCLRACVMKKLQIMDESGKLNKEHAIELVKVMSKHDAEKEDAPAEVVAKCEAIETPEDHCDAAFAYEECIYEQMKEHGLELEEH

>DmelOBP83b

MVKYPLILLLIGCAAAQEPRRDGEWPPPAILKLGKHFHDICAPKTGVTDEAIKEFSDGQIHEDEALKCYMNCLFHEFEVVDDNGDVHMEKVLNAIPGEKLRNIMMEASKGCIHPEGDTLCHKAWWFHQCWKKADPVHYFLV

>DmelOBP83cd

MQMKSGILIALCLCLSLNEGLALLEHEGETINRCIQNYGGLTAENAERLERFKEWSDSYEEIPCFTRCYLSEMFDFYNNLTGFNKDGIVGVFGRPVYEACRKKLELPFESGESSCKHAYEGFHCITNMESHPFTVIDNMPNISPSAKDAMKDCLQDVHQDEWKSFDAFAYYPVNEPIPCFTRCFVDKLHIFEEKTRLWKLEAMKQNLGIPAKGARIRTCHRHRGRDRCATYYKQFTCYAMAV

>DmelOBP56a

MNSYFVIALSALFVTLAVGSSLNLSDEQKDLAKQHREQCAEEVKLTEEEKAKVNAKDFNNPTENIKCFANCFFEKVGTLKDGELQESVVLEKLGALIGEEKTKAALEKCRTIKGENKCDTASKLYDCFESFKPAPEAKA

>DmelOBP83ef

MSSPRAVLVSLFLICSQALADLSGDAQTLEKCLRQLSSPESIAGDLRKLERYSSWTREEVPCLMRCLAREKGWFDVEENKWRLKQLTEDLGADVYNYCRFELRRMGSDGCSFAYRGLRCLKQAEMHAGTSLSTLLQCSRQLNATNVELLQYSKLKSKEPIPCLFQCFADAMGFYDPDGNWRLENWKQAFGPSGNEDQSSGADYSGCRLSGTQREVALSKCSWMYHEYKCWERVNGNKLVEDNEEQ

>DmelOBP57d

MPEKMSLRLVPHLACIIFILEIQFRIADSNDPCPHNQGIDEDIAESILGDWPANVDLTSVKRSHKCYVTCILQYYNIVTASGEIFLDKYYDTGVIDELAVAPKINRCRYEFRMETDYCSRIFAIFNCLRQEILTKS

>DmelOBP99c

MDLSEEEALQIAQSCVDDNAQKNPTDVWAFRGHQCMMASKIGDKVRAFVKAKAEEAKKKAA

>DmelOBP69a

MVARHFSFFLALLILYDLIPSNQGVEINPTIIKQVRKLRMRCLNQTGASVDVIDKSVKNRILPTDPEIKCFLYCMFDMFGLIDSQNIMHLEALLEVLPEEIHKTINGLVSSCGTQKGKDGCDTAYETVKCYIAVNGKFIWEEIIVLLG

>DmelOBP56g

MRATFALTLLLGCLSGILAQANIDSSVSKELVTDCLKENGVTPQDLADLQSGKVKAEDAKDNVKCSSQCILVKSGFMDSTGKLLTDKIKSYYANSNFKDVIEKDLDRCSAVKGANACDTAFKILSCFQAAN

>DmelOBP57a

MFNTRLAIFLLLIVVSLSQAKESQPFDFFEGTYDDFIDCLRINNITIEEYEKFDDTDNLDNVLKENVELKHKCNIKCQLEREPTKWLNARGEVDLKSMKATSETAVSISKCMEKAPQETCAYVYKLVICAFKSGHSVIKFDSYEQIQEETAGLIAEQQADLFDYDTIDL

>DmelOBP57b

MFIYRLVFIAPLILLLFSLAKARHPFDIFHWNWQDFQECLQVNNITIGEYEKYARHETLDYLLNEKVDLRYKCNIKCQLERDSTKWLNAQGRMDLDLMNTTDKASKSITKCMEKAPEELCAYSFRLVMCAFKAGHPVIDSE

>DmelOBP18a

MKVVCSIAVLWICLITMWQSAGRVNAEGCLKHHNLTSAQVQAVAPSTPVADVPVAVKCYSRCLIQDYFGDDGKIDLQKVGKRGSQEDHVILSQCKQQFDGVTNLDTCDYPYLILQCYFKGKQSGTIAS

>DmelOBP8a

MMRRSQIGLLSRLLLLLLVVELTPPAIPVPMRSSPQSLALLRARDQCGRELTAAQRLQLDRMQFEDAAHVRHYLHCFWSRLQLWLDETGFQAQRIVQSFGGERRLNVEQALPAINGCNAKTSSRGSGAQTVVDWCFRAFVCVLATPVGEWYKRHMSDVINGNA

>DmelOBP19a

MKFHLLLVCVAISLGPIPQSEAGVTEEQMWSAGKLMRDVCLPKYPKVSVEVADNIRNGDIPNSKDTNCYINCILEMMQAIKKGKFQLESTLKQMDIMLPDSYKDEYRKGINLCKDSTVGLKNAPNCDPAHALLSCLKNNIKVFVFP

>DmelOBP99d

MNHLRLEIICWSCLLIAMAVSTEAASVWKLPTAQMVYEDLEKCRQESQEEDAATLRCLVKKLGLWTDESGYNARRIAKIFAGHNQMEELMLVVEHCNRMEQDTSHLDDWAFLAYRCATSGQFGHWVKDFMSQKEVER

>DmelOBP56b

MKLIYLLVVFLIFALSELVAGQSAAELAAYKQIQQACIKELNIAASDANLLTTDKEVANPSESVKCYHSCVYKKLGLLGDDGKPNTDKIVKLAQIRFSSLPVDKLKSLLTSCGTTKSAATCDFVYNYEKCVVKGISA

>DmelOBP22a

MRVLLAFVLLLGLSVLATKEPEEVKIVSECAKENNVHRKKALDLLMSYRLKKKTHNVMCFINCIFERTNILQKVKEKVVKENHNCDSIKDADKCAESFQKFQCLVKIEMKVRGIDRG

>DmelOBP56c

MYFRASLMALLCLTLSEFVSKAWTRSLSVSLNMSMTRTLVPDPPNGTENKLSQEMLRACMRRTEISMSQLKLFHMSLMNSDYNNDNDIAPTPVQSIGDVNNLGDLDFNGNSQMPYLDLKHNEPLQCFVSCLYETLDLDRYNVLLEEAFKNQVQTIIQHEKAEIKECSDLQGKTRCEAAYKLHLCYNHLKTLEAEQRIREILERTEAENEGFGPEGSDFIDGIQHSGEAMTTAKSE

>DmelOBP56f

MKVFLLFIFISAIWLQAFCMKSSEKIKACLKRQLGYTITENTKFDAKEDSLQSKCFYHCLLEVKGVIANDAISSEQPRKVLEKKYGITDTDELEKAEEKCHSIKASGKCELGYEILKCYQSITKH

>DmelOBP19b

MMQCSRMTTTLKMTNLLLAVACAAVLMGSATADEEEGSMTVDEVVELIEPFGDACTPKPSRENIVEMVLNKEDAKHETKCFRHCMLEQFELMPEDQLQYNEDKTVDMINMMFPDREDDGRRIVKTCNEELKAEQDKCEAAHGIAMCMLREMRSSGFKIPEIKE

>DmelOBP49a

MLSKSQLLLLVVGFCLNAAVSADVDCSKRPSFVNPKTCCPMPDFVTAELKQKCIKFDMTPPPPPDGEASGSFESKRRHHHPHPPPCFFSCIFNETGIYQNRKLDEAKLNAYLQEVFEDSSDLQTTATQAFTTCATKVADFEANLPPRPAPSPPPGFPMCPHDAGHLMGCVFRNMMKNCPDSIRNDSQQCTDMKEFFTKCKPPRGPPPSAEDM

>DmelOBP56i

MVVCVQRTQVQAGPIKDQCMAAAGITAQDVANRHETDDPGHSVKCFFRCFLENIGIIADNQIIPGAFDRVLGHIVTAEAVERMEATCNMIKSETSHDESCEFAWQISECYEGVRLSDVKKGQRTRNHRG

>DmelOBP59a

MKQLIFLLICLSCGTCSIYALKCRSQEGLSEAELKRTVRNCMHRQDEDEDRGRGGQGRQGNGYEYGYGMDHDQEEQDRNPGNRGGYGNRRQRGLRQSDGRNHTSNDGGQCVAQCFFEEMNMVDGNGMPDRRKVSYLLTKDLRDRELRNFFTDTVQQCFRYLESNGRGRHHKCSAARELVKCMSEYAKAQCEDWEEHGNMLFN

>DmelOBP47a

MNRVLVLLLVLKMFALSESRFAKININLGLTVADESPKTITEEMIRLCGDQTDISLRELNKLQREDFSDPSESVQCFTHCLYEQMGLMHDGVFVERDLFGLLSDVSNTDYWPERQCHAIRGNNKCETAYRIHQCQQQLKQQQQNLLATKEVEVTTTPAGSDETKP

>DmelOBP51a

MKVFIGLVLLLAVTTLSSALFESEANECAKKLGITPDYFENFPHSSRVKCFYHCQMEKLEIIANGVVTPFDLKVLNISPESYDKYGVKVKPCLKLSHRDKCELGYLVFQCLKREFNL

>DmelOBP50aMRTGRILVALIFLGLIIPFRAAKCRAAPKSVQNVHVCCSAPLPNWGVFNRECHKSAIQASCRLDCDFNASSVLQGNRLIQAKVRPMLERAFSNEPTIDAYESNFAKCSTVVRSKYQELSPLSRQSDACDRHALFYSLCAYARLIFTCPDKMWQRNNRMCQEAKAYAKKCPWPALKMFMRNT

>DmelOBP50c

MARHIALLICSLLAMAGCDPIDVDCTRRQDFNIVKDCCVYPTFRFDQFKSQCGKYMPVGAPRISPCLYECIFNKTNTVVDGAIHPDNARLMLEKLFGNQDFEEAYFNGLMGCSDSVQEMISNRRSRPQRKTEQCSPFSLFYGICAQRYVFNHCPSSSWSGTESCEMARLQNMNCSKPSRGSSHRL

>DmelOBP73a

MRITQLLCISCMVITSIDAVEYLIRFETKKAKCLNPPRTARKVESVIRECQDEVRNKLVNEAYEILKEQVSQNQPPIDPNDDSIDFIWPSVPEAPSLDHSPNISQYEYIVYDEPEPQRHVARLMRNIRRLDVASSGIYHPTLVPLEDKRIAGCLLHCVYAKNNAIDQRGWPTLDGLVHFYSEGVHEHGFFMATLRSVNLCLRTMTARYGVNRKELPKKGESCDLAFDVFDCISDQITGYCLDQYSRY

>DmelOBP83g

MQSQSLLLIVAAVATFLVAQTTAKFLLKDHADAEKAFEECREDYYVPDDIYEKYLNYEFPAHRRTSCFVKCFLEKLELFSEKKGFDERAMIAQFTSKSSKDLSTVQHGLEKCIDHNEAESDVCTWANRVFSCWLPINRHVVRKVFA

>DmelOBP58c

MKCTILLSFFSLIWFAGGIKIDCENTEAINEDHIHYCCKHPDGHNDLIEGCARETNFTLPNQNEEALVDITADRAIRGTCFGKCVFSKLNLMKDNNLDMDAVRSLFTERFPDDPEYAKEMINAFDHCHGKSEENTSMFLSKPLFKQMSKQFCDPKSSVVLACVIRQFFHNCPADRWSKTKECEDTLAFSKKCQDSLATL

>DmelOBP50d

MLHKLTWVLIFIPAFRAADPICSQRPDVTALRNCCKLPNLDFSSFNSKCSQYLVNGVHISPCSFECIFRAANALNGTHLVMENIEKMMKTILGSDEFVHVYLDGFRSCGNQEKVLIKAMKRRRVPITGKCGSMAIMYGLCAHRYVYRNCPESVWSKSATCNEAREYSIRCDDM

>DmelOBP50b

MSSVLHLLGFLWLPLLVYSVSNDMGGLQKCTELLNTHKLVYCCGKSFLDKFPFVGSNCTPFWDDYGPCRYECLYRHWDLLDQDNKIKKPELYLMITSLYSPLNGYDKYGAAFKAAHETCEALGSRHADFLLLYSNQVADKMGMASSTCLPYAMLHAQCTMVYLTANCPRENWIDDPKCNSLQKLLSSCTKKLDEKTNALKGKDEELTDNGCGHIDSEGSNLLMACFLTLMIAKFISDH

>DmelOBP47b

MSPSQLLVIFASLALNTRLVFGQATIDCQRPPQLVDPALCCKDGGRDQVAEQCAQRILGTANGQKAGGPPSLDTAACLAECILTSSKYIDEPQKLNLANIRSDLSAKFSNDTLYVETMTMAFSKCEPQSQRRLAMIMQQQQQVQQQKTQQQQPRCSPFSAIVLGCTYMEYFKNCPDHRWTPNAQCTLAKAYVTQCGLGA

>DmelOBP46a

MCSQLFAFLLLLLTAFVTGRSTPPALDEDCELNSVDTMHDFCCDLHDESPQFSDCQMEWHEKIPYETDEEEQTYMFCTAECSFNSTNFLGRDRRSLNLNEVKEHLESDLVNDADIKLLYDTYVKCDKHALSLMPHKGVKQLSKRLSRLGCHPYPGLVLECVANEMILHCPTKRFRQTAQCEETRNHLKQCMQYLKYKS

>DmelOBP50e

MHKYIICFGFLLIILECSLASFNCSAPPNFNNFDINTCCRTPELDMGDVPQKCHKYVSGLKSANSKYPSYAHLCYPDCIYRETGAMVNGKIKVNRVKQYLEEHVHRRDQEIVSHIVQSFESCLSNVKGHMKSLNIESYKVLPHGCSPFAGIIYSCVNAETFLNCPQQMWKNEKPCNLAKQFAEQCNPLPHVPLPSS

>DmelOBP93a

MYVYNLLFVVIVFSYCAKSFNYTSCDHAKQPKFLSSCCDVQKNDKAINSCRKSLLGNNSTNSNGEVRNLKSDKVALHACIAECSFRTNGFLLSNGTVNTQALQKSYQQRYKNDPNMSQLMLKSLNSCTDYARKRVQEFQWMPKKGDCDFYPATLLACVMEKVYINCPTSKWKNTSDCTAMWKYLVACDDVASNKKK

>DmelOBP58d

MVNIVCYWTFLILVAVSKAQDNEETTAVAISSGDLTEDKCNTSRAGCCSELYIGEEEDLVKCFVIHSPKLPVDGDADIGKTLRFLSCFVECLYKQKKYIGKSDTINMKMVKLDAEKTFVDRPKEKDYHIAMFEFCRKDAVGVYNLLKASPGAKVLLKGACRPYLLMVFMCISDYHQKHECPYFRWEGTAKAGTKDMCENAKAECYQIDGITLPTKSPA

>DmelOBP85a

MSPGSVVFSMFDLSIKCCQLTRPSLDKGNSECRKSLNLPAHRKFNFAELYTINMCIEECNFIGCGYIEIDPPFRLDLANIRTNLQTIAPQPQNESIPFLVDAYRKCELFRSSHGRRFTLHLPDIEFIEEPCNPFALQITICVRIHAMQKCPSEFYVDSDECRLAREYFTQCVGDIETNLA

>DmelOBPLush

MKHWKRRSSAVFAIVLQVLVLLLPDPAVAMTMEQFLTSLDMIRSGCAPKFKLKTEDLDRLRVGDFNFPPSQDLMCYTKCVSLMAGTVNKKGEFNAPKALAQLPHLVPPEMMEMSRKSVEACRDTHKQFKESCERVYQTAKCFSENADGQFMWP

>BdorOBP99a.1

MKYIVAVLLAALVAMAAAEEYKIRNQDDLLKARKECMEAKKVPTEHIEKFKKFEFPDDEVTRCYIECIFNKFQLFSPTEGFKTQNLIAQLGQNKENKDAVKADIEKCADKNEQKSDSCTWAYRGFKCFISKNLPLVQESLKKN

>BdorOBP56d.1

MNFLAVAVLIVFVTVAVAQEGVGKLTEEQKQKVHAAAAECFKETGASEDAVRALLKGDDSQVDGKVKCFAKCTLGKLDLLQNGKVNEEKVQNILGKLIGDEKAKAAQAKCNGLKGTDECDTAYQIRQCYAAGHESFVF

>BdorOBP70

MLWMNCFLLSYIYNRCLNPPRTARRVESFIQECQEDVKNKLISEAYYILKSQIKNKNTALDITVDNISEINSAPAPVSVPSIIFHTIEENSKLASQHDAYQFQPERQQVTSLMNHIRRISYNPRSALYYPTLVPAEEKRLAGCLLHCVYAKNNAIDKLGWPTLDGLVNFYSEGVNEHGFFMATLRSVNLCLHAITVKYNIDRRKLPKRGESCDLAFDVFDCISDHLTGYCLNQYE

>BdorOBP56h.1

MQKFYILTIIAALVTLAVCQLPADLEKFHKACMDEAKVTDEQMRQFFQNGMKASDATENIKCQMKCMMQKQGIWKDGVFDADAKIKELVQNPKFKGKEAELTKAINNCKNEKGANECDTVFKISMCIKEFMTQNNL

>BdorOBP99a.2

MKYFMFIVILAVVALVQADDWSPKTVDDIKKIREECMKQVPSSDEEFQKRKENDYPDVESVRKYALCNSKGWGLYKEGKGFYPDRVAEQFKDDMPEDEIKAIVNDCDEKTKEETDDERCYHLLKCVMSTKLGDHIKDLVKRLE

>BdorOBP83a

MHSRKTLLGTLLWIGFLLNLIWAQKELRRDETYPPPELLKELRPVHDSCVAKTGVTEEAIKEFSDGDVHEDELLKCYMYCVFEETDVLHEDGEVHLEKILDKLPESMHVIALHMGKKCLYPKGDNKCERAFWLHRCWKEADPKHYFLI

>BdorOBP28a

MAKFILFAALCILSAAVSNAAFNKEEAIKNFMTRAEECRGEVGAADSDIQDIVAKVPASSKEGKCLRSCLMKKYGAMDSNGKFVKSVADQHAQDFTDGDADKLKTAREIIDACADIAVPDDHCEATEVYGKCFMDQAKAHGIQKFDF

>BdorOBP84a.1

MSNGNVFVMLPLTIIQLYCGIMVSAQDRAKDNGDIFVQHKEQRECVAPIMVQANGSVSSEGMDVAHICNNSFSIPSDYIVQFNRNGDLPEIVDKTGMCFIRCYFEKAGLIKNWQLNKDLIMQTMWPIKADSIAICEPEAKQEMNACVRSYAIAKCLMKRGFQDTCNDTVA

>BdorOBP99a.3

MKFFIVILAVVALAYADEEWVPKNVAQIKAIRQECIKDFPLSEEYIQKMKNFEYPDEEPVRKYLLCTAKKLGVFCEHEGYHADRVAKQFKMDLDEAEVIAIAEGCADKNVEGSSADVWAYRGHKCVMASKIGERVKAYIQKSVEEAKKH

>BdorOBP56d.2

MKFFAVAVLLAFVAVAAAQEGVGKLTEEQKQKARALGTECLKETGASEEAIRALIKGDDSQVDGKVKCFSKCMQEKLGFVENGKVNEEKVQNFLGKLIGEENAKATQAKCNDLKGTDECDTAFQIRQCYAAGHEGLDF

>BdorOBP19a

MLNKINSFVLATVFVALVLHSDQVSGGATEEQMISAGKLMRDVCLPKFSKISPEVADGIKEGNVPDTKDVKCYINCIMEMMQTMKKGKFLYESALKQIDLLMPDDYKDDYRNGLAKCKDVTSGIKNNCDASYALLICMRDNISKFLFP

>BdorOBP19d.1

MKILNICLIVCVALISNAKCNYEEAKAVANECKGEVGATDDELETILKMEAAETTTEKCLGACVMKRFGAMNGDGKFDREKAMEILAIIADGNEEQHALGVEVLDACADIDVNEDHCEAAEEYRTCMHAKAKEIGFVVGRV

>BdorOBP19d.2

MKCFVVFLAICSFAISFSEADEFGEKVKKIAEECKGQVGASDDDVARLFKYEPAANDKAKCLTACTMKKLGTMDENNKVVEAGAIAYIKQLSGGDAEFEKLSLETYNECKSTPESSNECEYAEAFRQCVLESAKSKGLKILPQV

>BdorOBP84a.2

MINHRLLILALSLVLLGFLAGTRAHPETDATNNKLDKQQSMEMTTPTAGAANETGFDFEEVVRTCNASYTIPLEYIQQFNETAELPNITDKTGMCFLKCYMEKTGLLRDWQLNPTLIRQTMWPATGDSLPVCQNEGSRETCPCKRTYAIAKCLTLRALVDARNKPLV

>BdorOBP19d.3

MEILIVLQILAVSSVFTITIAAAESEPPHYSSLRVMAEAAIEDCYEDSAQSVKVQITDESFDEILKGSRTNLSHSAKCLRYCIMRKNGLLSMDNSIDEENILQIFEIIHPQIKKDSLLDVLYKCARETDKQTDNCERAFVATSCILRELQADGITDI

>BdorOBP56h

MKSFVTIALLVVGSAVVLCNPHDPEMRGYIEDCNKEHNVSPKDFHDFIEGKLTTVPENMKCSSQCIMVKQGIMDESGNFKPDAAKAKMKEDKLVAAVDECKDLSGSTPCDTAFKITSCMLSKK

>BdorOBP99a.4

MNTQLILLLACVALVAGKFQIRTAQDALDAHEACHEEYRVPEDIYQKFLNYEFPAHKRTNCYVKCFVERMGLFTEEKGFDEKAIIAQFTAKSSKNLAKVSHGLEKCLDHNEHDSDTCTWANRVFSCWISVNRPIVRRTYIEN

>BdorOBPLush

MYLKINALKYFLTLLACTAVSAVTMQQFETSLDMMRNGCAPKFKIATEILDNLRAGEFIENNGDLKCYTRCIAQLAGTVTKKGDFSVQKALAQIPIILPPEMQDPAKEALNACKDVQKNYKESCDKVFYTTKCVRDFDPATFKFP

>BdorOBP19d.4

MVKSSSALLIAGFICLMSLQSLTALSEDADKLSEKRKPLMTREDPSTLEDYKRTKRQLPQPLQEFQDFVTTSKTECAKEMNINPNELQKSLLYEDQPTSIEKCMMECVLKRIEVMSKDDTLSTTTIGHIADIIGDNNALITSIAMASAENCKKFITAEDSCERAFQINKCIAAEMKMRKIKLIY

>BdorOBP99a.5

MKFFLVILAVVTQTYAEDEWRPKNMAELNAIRQECFKEYPLSEEQLQKIKNFEYTDEEPARKVLLCTVKKLGVFCEREGYNADRVAKQFKMDLDEAEALAIVEGCLDKNLEGSSADVWAYRGHECVVASKIGDRVKAYFLKSKK

>BdorOBP57c

MYQFGAHEKRATTAATMSATVLAAGGGKGMSIPGLTWLLLAVIVVFALPPGAVALTPTAPTRSFVEACQVKHNITLQELDEFPTDPSPEDIDMKFKCYADCLLNGMGFMDSNGKLDAEGLHEWGILNDESYENMLECKAANDMEDDPCEYSFGMMLCARMLNSEEENYYSDEVDEAAEERRRK

>BdorOBP99a.6

MKFCLALLSLLMVVVFAVADHAGHTDYVVKTNEDLIRYRDECVSKLSIPSDLVDKYKEWSFPDDEKTRCYLKCVLEKFELFDAAKGFDVHNIHHQLVGANADHSDATHGAIENCAKEAAGDDACVRAYNGFTCFLKNNAQLVQAGVEKSSK

>BdorOBP69a.1

MNTKQFVFLVIIYQYTFYTGVTTLEVPKHMVSGVKKLTNICIKESGASEDLFKDIRATGELPNNQNLKCFMHCVLDKIGLIDDDNIVHLDNLIEIMPPDFVPIIEQLHTTCGTKSGADGCETAFLTIECYIKKEPIISKMLFSTFAD

>BdorOBP99a.7

MKFFIVILAVVALVYAKDEWVPKTEAELKVIVKECLKDFPLNNEQLQKYTTFQQPDEEPIRKYMLCTAKGVGFFSEHEGYHVDRVAKQFKLDLDEAEVAVITEGCADKNAEGSSVDVWAYRGHKCVMASKIGERLRVYIQNLKKEAKKH

>BdorOBP68

MKAYFGYFVVIFVFVCHASADDETVDCTKPPRFVPPHMCCPVPDVSTDELKEQCAEYNKPPPPPPMGRGGPPKFDRRHHPHHTPPCVIDCIFNNTEVMGANGEPDVDKFSALLDTAVKDNEEMAAVMEESFETCVGMLSELKAKMAEKASKHPEFADRMGNCSPVSGMLMMCVNIETFKNCPASAWNDSTECNATRNFFKQCKFPKDGN

>BdorOBP6

MVLTGTRRGQAFHAFLIVALSSSLTLMHVQAQEPRRDDKWPPPAVLKMAKIFHDICVEKTGVTEEAIKEFSDGQIHEDEALKCYMNCLFHEIDVVDDNGDVHLETLFNTVPGTVRNQLINMAKECEHPEGDTLCHKAWWFHQCWKKADPVHYFLP

>BdorOBP56h.2

MPLTLLCFIFALLVVAQADMTPLQLMDACNKESGITKEELQQYFDSQMDPAKATNAIKCHMKCVSEKLGFYKNNMLDDTLTIKYLNENNMAPKASVNNVKQSIQKCNQMKGANTCDTAYQIMTCFKSQPIFT

>BdorOBP19d.5

MYFCKAIFISSLLAFLYATPISAGITEEQMWATAKLMRDVCLPRFPKISIELANQLRDGNIPDNNKDVKCYINCVLEMMQTMKKGKFLYEASLKQVDLVLPDSYKDDYRAGLLKCKDASAGIKKDNCEAAYTILKCLRGEIKKFIFP

>BdorOBP69a.2

MKSYGLYQLLFAVFICSAITQLHAADDKAAREACIAEAKLTATDANQVRVAAVISKLIQNDSEALKCFQLCYYKQLGLIDAAGKTNAAKTLEYMSQVSGITDTSKLAAALGTCESVKGSSGCDRLYQFEKCALAKLGV

>BdorOBP99a.8

MKFFIVILAHVALAYAEDEWMPKNMAELNVIRQECLKDFPLNDEYIEKMKNFEYPDEEPVRKYLLCTVKRFGIFREGEGYNIDRVAKQFKMDLDEAEALAIVEGCVDKNTEGSSDDVWVYRCRKCVMASKIGDRVKAKSRE

>BdorOBP99a-like

MKFFIVILAVVALAYAKDEWVPNTEAELKVIAKDCIKDFPLSNEQVQKYTTYQHPDEESLRKRMLCAIKKAGFFSEHEGYHADRIAKQFQIDFHEAEVAAIAERCADKNVEGSSVDVWAYRGHKCVMTSKIGERLKARNQKIEQ

>BdorOBP56a-like

MKSSIVCYLLATVVLSLCVFNADAGLRKPKKLTPELEAKFEVLTAWIAYRLNLKHAKEACVGEYGFSDELATNLVKIKVANPSDREKCYVNCLYNKLVFYKDDAINKQAMKESLYEIVGEQRLLNIVDGCLNAGGTNACDKVYKFHACASPEFDKVRSDIFLPDE

>BdorOBPA10

MLRFVAASVLICAVYHVTTTSAAPHPPTTAAPLVANQAAYDTKFDNIDLDEVLNQERLLRNYIKCLENTGPCTPDSKMLKEILPDAISTDCAKCSEKQRLGSAKVTHFLIDNRPEDWARLEQIYDPQGNYRLNYLAAKDKGDGVEKTTEAVTKTQA

>BdorOBP56a.1

MKSSNICCILATVLLSLCVFNAEAGLRKPKKLTPELEAKFEVLTAWIAYRLNLKHAKEACVGEYGFSDELATNLVKIKVANPSDREKCYVNCLYNKLVFYKDDAINKQAMKESLYEIVGEQRLLNIVDGCLNAGGTNACDKVYKFHACASPEFDKVRSDIFLPDE

>BdorOBP99a.9

MKFFIVMLAVVTLAYAEDEWMPKNDAEVSVIRQECIKDFPLSEEQLQKFRIFEYPEEEALRKYLLCVTKAVGIFTEHEGYHADRVAKQFNINLDEAEVTIIAEGCADKNVEGSSADVWAYRIHKCVMASKLGERVKAYIQNLKKEAKKH

>BdorOBPA5.1

MWHKVKRIVIIFLAVNCASAEDTEAEKLFRELEVVPDILDEPPKELLKIEYDGGLVVGRGEEFTPTQTKDEPKVDWTAEPDAFYTIIMTNPDIPTRQNPATREWLHWLVVNIPGTDLAKGYVLDPYIGPLNPKESGLVRNVFVVFKQLGKQEFDEPILNNTNVAGHERFSSKGFAKKYDMELVAGNIFTSRWDEYVTLLHKQFGIIK

>BdorOBPA5.2

MWQRILCFFLAARCAVAVDSDVEKLFRDMEVVSDILDEPPKEMLKIEYNDGLEVGNGEEFTPTQTKDEPKLYWTSEPDAYYTVIMVNPDIPTRQNPLLREWLHWLVVNVPGVDIAKGDIIDPYIGPMAPKMSGVLRYVFLIYKQPGKQVFDEAKITNTDVTGHEKFSSMGFAGKYNMELVAGNLFQARWDELVPSLHKQFGISL

>BdorOBP56h.3

MMKDLFILGILSTLYSMAVCNEMMADEKFELPCLIEANLTEADFKKFRSNGLKANEANANIKCMAKCLMEKREVLKKGVFDPEKVYADLIRLPELKGLEDQIKEAINICKTEKGANDCDTAFKITMCLREFKSRNI

>BdorOBP56d.3

MKSYILLVALISLSAVAAHQLKSNKSTQAHQFYEDCLKESGASAAQLDALKKGDFNAVDDKAKCFLKCLQNKKGILENGVPNEAAIHKVMTPAVGNSPPKNTLAKCNGLKGANECDTAFQIYKCYRQEHVGLI

>BdorOBP99a.10

MQFFIVILVVIALAYAEDEWIPKTEAELEVIAQECLKDFPLSKEQLQKFSSFEYPDEEPIRKYMLCTAKRVGFFTEHEGYHADRVAKQLKMDLDEAEIVAINDGCADKNVEGSSADVWAYRVHKCVMASKIGEHGKAYFQK

>BdorOBP99a.11

MKTVIALCLLLAVTSAEYVVKNEENLQQYRRECATELKVPAEHIEQFRKWQFPNDAVTQCYLKCVFEKFGLFDAVTGFNVEHIHQQLQGAEVAPPGDADHDDVVHDKIAVCVDTNEQGSNACEWAYRGGVCFIKENLQLVKHSVKPQA

>BdorOBP28a-like

MAKLILFAALCILSAAVSKAAFNKEEAIKNFMTKAEECRGEVGAADSDIQNIVAKLPVVSKEGKCLHSCLMKKYGVMDSNGKFVKSVADQHAQDVTDGDADKLKTAREIIDACADIAVPDDHCEAAEVYGKCFVKQAIAHGFQKFDF

>BlatOBP99a-like

MKXFLAFLAHFALAYAEDEWRPKNVAELNAIRQECIKDFPLSDEYIQKMKNFEYPDEEPVRKYLLCTVKKFGIFREDEGYNVNRVAKQFKMDLDEAEALAIVEGCVDKNIEGSSDDVWAYRCRKCVLASKIGDRVKAKSKEESYKQ

>BlatOBP57c

MYQFGAHEKHATTTAIMSATVLPAGGGKGMPIPGLMWLVLLAVIVGFTLPPGAVALTPTAPTRSFVEACQVKHNITLQELDEFPTDPSPEDIDMKFKCYADCLLNGMGFMNTNGKLDAEALHEWGILNDESYENMLECKAANDMEDDPCEYSFGMMLCARMLNSEENDYNSDEVDEAAEERRRK

>BlatOBP99a-like1

MKFFLLILAVISRTYAEEEWRPKNAAEVNVIRQECLKDFPLSEEELQKVKNFEYSDEEPARKVLLCTVKKLGVFCERDGYNVDRVTKQFKMDLDEAEALSIVEGCMDKNLEGSSADVWAYRGHECVVASKIGERVKAYFLKNGEKPKK

>BlatOBP99b

MKFCLALLSLLLAVVFAVADHAGHSDYVVKTKEDLIRYRDECVSKLSIPSDLVDKYKEGSFPDDEKTHCYLKCVLEKFELFDESKGFDVHKIHHQLEGANADHSDATHGAIENCAKEAAGDDACVRAYRGFTCFLKNNAQLVQDGVKKSSK

>BlatOBP99a-like2

MXQAMKCTAFVFILLFAALFAHINADYEEKTEDDFLSAGERCFQRERLAASYQRRFDNFDYPDEEPVQRYVHCIWTELKLWNDRTGFNVEHIAALYRDKANTEVLVPILSDCNRNAQNEPTLKWCYRAFKCVLNSRVGQWFKEDVGRKLEERRXGNHVA

>BlatOBP99a-like3

MXQAMKCTAFVFILLFAALFAHINADYEEKTEDDFLSAGERCFQRERLAASYQRRFDNFDYPDEEPVQRYVHCIWTELKLWNDRTGFNVEHIAALYRDKANTEVLVPILSDCNRNAQNEPTLKWCYRAFKCVLNSRVGQWFKEDVGRKLEERRXGNHVA

>BlatOBP56h-like

MKSFVTIALLVVGSAVVLCNPNDPEMRGYIEDCNKEHNVSLKDFHDFMEGKLTTVPDNLKCSSQCIMVKQGIMDESGNFKPDVAKAKVKDDKLVAAVDGCKDLSGSTPCDTAFKITACMLSKK

>BlatOBP70

MISNVVCRAYIALILLDKSGILAKDSHSFTKNRCLNPPRTARRVESFIQECQEEVKNKLISEAYYILKSQVKNENTALDITVDNISEINSAPAPVPVQSIIFHTIEENSKLASQHDAYQFQPERQQVTSLMNHIRRISYNARSALYYPTLVPAEEKRLAGCLLHCVYAKNKAIDKLGWPTLDGLVNFYSEGVNEHGFFMATLRSVNLCLHAITIKYNIDRRKLPKRGESCDLAFDVFDCISDQLTGYCLNQYE

>BlatOBPLush

MQQFETSLDMMRNGCAPKFKIATEILDNLRAGEFVENNGDLKCYTRCIAQLAGTVTKKGDFSVQKALAQIPIILPPEMQGPAKEALNACKDVQKNYKESCDKVFYTTKCVRDFDPATFKFP

>BlatOBP19a-like

MMYFCKAIFTTSILAFLFLTPISAGITEEQMWATAKLMRDVCLPRFPKINIELANQLRDGNIPDDNKDVKCYINCVLEMMQTMKKGKFLYEASLKQVDIVLPDSYKDDYRAGLLKCKDASAGIKKDNCEAAYTILKCLRGEIKKFIFP

>BlatOBP56a-like

MKXSIICCILATVLLSLCVFSAEAGLRKPKKLTPELEAKFEVLTAWIAYRLNLKHAKEACVGEYGFSDELATNLVKIKVANPNDREKCYVNCLYNKLVFYKDDAINKQAMKESLYEIVGEQRLLNIVDGCLNAGGXNACDKVYKFHACASPEFDKVRSDIFLPDE

>BlatOBP56h-like1

MQKFYILTIVAALVTLAVCQLPADLEKFQKACLDEAKVTDEQMRQFFQNGAKASDATENIKCLMKCMMQKQGIWKDGVFDADAKIKELVQHPKFKGKEADLTKALNDCKNEKGANECDTVFKINMCIKEFMTQNNL

>BlatOBP56a-like1

MKSSIICCILATVLLSLCVFSAEAGLRKPKKLTPELEAKFEVLTAWIAYRLNLKHAKEACVGEYGFSDELATNLVKIKVANPNDREKCYVNCLYNKLVFYKDDAINKQAMKESLYEIVGEQRLLNIVDGCLNAGGANACDKVYKFHACASPEFDKVRSDIFLPDE

>BlatOBP56d-like1

MKFFAVAILLAVVAISAAQEGVGKLTEEQKQKAHAVGTDCLKETGASEEAIHALIKGDDSQVDSKVKCFTXCMLEKLGFIENSKVNEEKVQNFLGKLIGEEKAKATQDKCNGLKGTDECDFAYQIRQCYSSGYSGFAF

>BlatOBP56d-like2

MKFFAVAVLLAFVAVAAAQEGAGMLTQEQIQKVHTLSTECLKETGASEEAIRALIKGDDSQVDGKVKCFTKCMLDKLGYVENGKVNEEKVQNILGKLIGMEKAKATQAKCNGLKGADECDTAYQIRKCYSAGYNGFVF

>BlatOBP83a-like

MHSRKTLLGTLLWIGFLLNLIWAQKELRRDETYPPPELLKELRPVHDSCVAKTGVTEEAIKEFSDGDVHEDELLKCYMYCVFEETDVLHEDGEVHLEKILDKLPESMHVIALHMGKKCLYPKGDNKCERAFWLHRCWKEADPKHYFLI

>BlatOBP84a-like

MINHRLLSLALSLLLLGFLAGTWAYPEIDATDNKLDKQQPMETTTPNAGAANETGFDFLEVVRTCNASYTIPLEYIQQFNETAELPNITDKTGMCFLKCYMEKSGLLRDWQLNPTLIRQTMWPATGDSLPVCQNEGSRETCPCKRTYAIAKCLMLRALVDARNKPLV

>BlatOBP19d-like

MEILIVLPILAVSSVFTITIAAAESEPPHSSSLRVMAEAAIEDCYEDSAQSVKVQITDESFDEILKGSRTNLSHNAKCLRYCIMRKNGLLSVDNSIDEENILQIFEIIHPQIKKDSLLDVLHKCSRETDKQTDNCERAFVATSCILRELQADGVTDI

>BlatOBP28a

MAKFILFAALCILSAAVSKAAFNKEEAMKNFMTKAEECRGEVGAADSDIQDIIAKVPAASKEGKCLRSCLMKKYGAMDSNGKFVKSVADQHAQDFTDGDADKLKTAREIIDACADIAVPDDHCEATEVYGKCFMEQAKAHGIQKFDF

>BlatOBP56a

MDSKWIVLAGAILASIGNLPPFPAVEARSASVKMSLDLSVSEDQHRKTADMVRLCALETDLSMDDLRRFSENDFSNVTKATQCFTHCLFEHMGLVSNGIFVERDIISFLGDVTDPKRMLERECLGHFSDNKCETAFLIHQCYQSGQRLMRPPYNQPEPVDQLNEEPNRKEETRAMDLSTTDMAMPSAHRMSPDAEHQLLIKNILAKRLPRKQNIKQGLEEE

>BlatOBP66

MKNFVLIILSVSLVLKLSKSLKIDCDNPESIKEDRIHYCCKHPDGYQGVVDSCAKETGFKFIKHDEEAMVDITVDHAITGTCFGKCVFNKLEFMKGNDLDMTAVRAHFENKSKADPEYAKEMINAFDHCHGKSVENTAKFLSNPIFRQANAEFCDPKPAVILACVIREFFHNCPADRWAKTEECNTVLEFSKKCKDALTTI

>BlatOBP84a

MSNRNVFVMIPFTIILLYCDIMVSVEDRAKDNGDIFVQHREQRECVAPIMVHANGSNSSEDMDVAHICNNSFSIPSDYIVQFNRNGDLPETVDKTGMCFIRCYFEKAGLIKNWQLNKDLIMQTMWPIKADSIAFCEPEEKQEMNACVRSYAIAKCLMKRGFQDTCNDTVA

>BlatOBP99a

MNTQLILLLACVALVAGKFQIRTAQDALDAHEACHEEYRVPEDIYQKFLNYEFPAHKRTNCYVKCFVERMGLFTEEKGFDEKAIIAQFTAKSSKNLAKVSHGLEKCLDHNEHDSDTCTWANRVFSCWISVNRPIVRRTYIEN

>BlatOBP56h-like2

MPLILLCFMAALFGFAQADMSPLELMDACNKESGISKAELQEYFDSQMDPAKATNAIKCHMKCVSEKFGFYKSNMLDETVTIKYLNENNMAPKASVNTVKQSIQQCNQLKGANSCDTAFQIMSCFKSQPIFT

>BlatOBP56h-like3

MMKDIFILMILAALYSIVVCKEMIADEKYELACLIEAHVTEADLKKFLSNGLKANEANANIKCMEKCIMEKREIIKKGVFDPEKAYAEIIRMPELKGQEDHIKEAINVCKTEKGANDCDTAFKIAMCLEEFKSRNA

>BlatOBP99a.1

MKTVIACLLLAVTSAEYVVKNEENLQQFRRECATELKVPAEHIEQFRKWQFPNDSXTQCYLKCVFEKFGFDAETGFNVEHIHQQLQGAEVAPPGDADHDDVIHDKIAACVDTNEQGSNACEWAYRGGVCFIKENLQLVKHSVKPQA

>BlatOBP99a.2

MKYIVAVLLAALVAMAAAEEYKIRNQDDLLKARKECMEAKKVPTEHIEKFKKFEFPDDEVTRCYIECIFNKFQLFSPTEGFKTQNLIAQLGHNKENKDAVKADIEKCADKNEQKSDSCTWAYRGFKCFISKNLPLVQESLKKN

>BlatOBP68

MKAYFGYFVVIFAFVCHASAQDETVDCTKPPRFVPPHMCCPVPDVSTDELNEQCAEYNKPPPPPPVGRGGPPKFGRRHHPHHPPPCVIDCIFNNTEVMGANGEPDVDKFSALLDTAVQDNEEMAAVMEESFETCVGMLSELKAKIAEKASKHPEFAGRMGNCSPVSGMLMMCVNIETFKNCPASAWNDSTECNATRDFFKQCKFPKDGN

>BlatOBP19d

MVKSSSALIIICLMSLQSLTALSEDADKLSGKRKPLMTREDPSTLEDYKRTKRQLPQPLQEFQDFVTTSKTECAKEMNINPNELQKSLLYEDQPTSIEKCMMECILKRIEVMSKDDTLSTTAIGYIADIIGDNNALITSIAVASAENCKKFITAEDSCERAFQINKCIATEMKMRKIKLIY

>BlatOBP19a

MLNKINSFVLATVFIALVLYSDQVSGGATEEQMISAGKLMRDVCLPKFNKISPEVADGIKEGNVPDTKDVKCYINCIMEMMQTMKKGKFLYESALKQIDLLMPDNYKDDYRNGLAKCKDVTSGIKNNCDASYALLICMRDNISKFLFP

>BlatOBP56a.1

MKSHGLYQLLFAVFICSTITQLHAADDKAAREACIVEAKLTATDANQVRGAAVISKLIQNDSEALKCFQLCYYKKLGLIDSAGKTNAANTLAYMSQVSGISDTNKLATALGTCESVKGSSGCDRLYQFEKCALAKLGV

>BlatOBP56d-like3

MKSFILVAALIALSTVAAHELKSNKSTQVHQFYDDCLKQSGASAAQLDALKKGDFNAVDDKVKCFLKCVQNKKGILANGIPNEIAIHKVLKPAVGNSAPKDILAKCNGLNGANECDTAFQIYKCYWQEHVILI

>AludOBP56h2

MNSFITVALILVFSATTLCQPHDPEMRKIVEECNKEHNVSPKDFHEFMEGKLATPSNDLKCSMQCAMVKQGIMNESGTFNADAAKAKMPTDSKLASAIDACKNEAGSSPCDTAAKITQCLMAHK

>AludOBP99a3

MKYFIVILAAVVLAQAQDDWKIKSANEVNDIRRECHKEHPFNEELQKHEEVLRFPDEDVVRNYEVCVFTKWGVFDAETGFKKDRLVRQFEPVLKREEIDEIIGRCADKNEQGSPVDVWVYRFQQCVSRSEIPPNFLKIIGKL

>AludOBP73a

MLKEEVYKDYQPLDANDDNIQLMRDPPTSVPDSAAFQGTGSGSSAQSAASASSLTTAEGFDLPLGYDSYQVKPSKRRLTRLMHSIRRITYDQPNNIYHPTLVPFEDKRIAGCLLHCVYAKNNAIDKLGWPTLDGLVDFYSEGVNEHGFFMATLRSVNLCLRAVTMKYRINRHKLPERGESCDLAFDVFDCISDQITGYCLDQYK

>AludOBP19b

MKMLQTVRIFTGTILLIFTGNVLADEMMNLPMGLLIEAVEPYAINCDPKPELEHAQELFLNKEDAQHTTKCLRRCLMDQFELFIDGGTQVNSEKLVGYMLLAYPDKMEELNEISNGCNEQNEEMGIDEICEVAHVFGMCMLKEMQSREYEIPKVVE

>AludOBP59a

MSKRSVVFNFCRTGISVLLLVSTCYALKCRTDDGPSEVELKRITRSCMRKIGENAHSSKGGSKSSSNNRHYGPLHQSNFGAQYNSNSSYDYNYDYEDDDNDRYNSNNNNKYNNNNYNYNNGNYERDQGNRNALQQRNRERQQNNRGDSNRSVSSASSAGGNNNGGRHENGNGNGGSGGGNGSGSGSGNSQRSYGQRNSNSNNNNKSSNNDTADATCVVHCFFEEMNMLNSDDYPDRYKVQYGLTRDMRDRELRNFYTDTIQDCFHYLESQRRRDKCHYSRDLINCMSEYAKVNCDDWQEFNMVFN

>AludOBP19a2

MSRKLIFCSLITVFIILVLQVTRISGGATEEQMIAAGKLMRDVCLPKFSKVSTEVADGIKLGNVPDTKDVKCYINCVMEMMQTMKKGKFLLESSLKQVDLLMPDDYKDEYRNGLTNCKDAANGIKNNCDASYALLICMRDQIKKFMFP

>AludOBP56a

MKSTISCCILATVLLCLCATSTDAVLLCLCTTNTDAFGRKPKKLNPELEAKFDVLTAWIAYRLNLKHAKEACVGEYGFTDELATNLVKIKVANPHDREKCYVNCLYTKLVFYKNNSINTQAMKESLSEIVGEDRLLNIVNSCLNVGGANDCDKVYKFHACASPEFDKVRGDIFQPDE

>AludOBP49a2

MHLSAKMLPTITFSTVWLLAAICRAEQKTTTDCSKLPKSIAPQSCCRFPEPFQNPILNECYNLHSDIGQCFVECLFNRSGICRQGKCSYQRAIDYLEREIALQQSAFKDIYKRAFKKCIAKANDVLGNIVKRFSRHGCHPLPEIIRFCVRNEMFTSCPKSYWNDKVAGCSKKRDFIRNCIKDN

>AludOBP28a

MAKLILIAIFCVLSGTLSKAFNKEEAIKQFVTRMEECREEVGATSSDIEELVKKLPASGKEGKCLRACLMKKYGVMNDGGKYIKAVALEHAATYADGDETKMKTATEIIDACAGTAVPDDPCEAAEVYGHCFMEQAKAHGIEKFEF

>AludOBP19c

MPAIFVSKPSSAQNVGHYKSVKWLASTTSDPIRQAFVNHEIVKKMSNSNNKTLVHILLCLATIQSLTALDADLYKLSGKRKPLVTREDPSDLDDYKRIKRHLPQPMQPMQEFQDFITSMKVQCATEMGFKPNEYEKSLLHEDQPTPKEKCLMECILKRMEVMDKNNSLSTPAIGRIADIIGSNNALITSIAMASAENCKKFINAEDSCERAFQINKCIANEMKMRKVKLIY

>AludOBP83a

MVLNGIWRAQAFYVFLIVALSSSLTLMHVQAQEPRRDDKWPPPAVLKMAKVFHDICVGKTGVTEEAIKEFSDGQIHDDEALKCYMNCLFHEIDVVDDNGDVHLETLYDTVPGTVRDKLINMAKDCVHPEGDTLCHKAWWFHQCWKKADPVHYFLP

>AludOBP99a-like

MKFYVFLIALFGLVFPEVKSISRVEVTKHRLGCLIQNPNACEYFVKILIFEYPDVPDVHSYMDCVPDKLGLWDSKTGQPNVDRVAAFFSVGPNNAEDMEIIKNCVAKAEGAEVHGLPGHLCILNSRLRDNLKK

>AludOBP50e

MEFTYTLVVLSACLLSYAHAAAFNCSEPPNLGAFDIHSCCRMPEINLGDAPAKCSAYIKELTTQMANMNGVGGSKQTENNKNMNNQNNPDAAAVEYPAYAHVCYAECMYRETGSMVENEFNMDNVEKFLNKSVSKGDKDIVPQIVRSFEACLNNIKGHLEAVGIKTHAKLPMGCSPFASLMFSCVNAETFLNCPAKMWKNDHNCNVAKSFAAQCNPLPHVPLPTANWLKP

>AludOBP99c

MHKPLTNSAECINMRQPRLNNSVLLRVKTSNKSQQHTKIKMKYFIIAILAIVALAHAEDEWKVKTAADIKVIRQECIKEFPLSEEDIQKMKNFEYPDEEPVRKYLLCTAKKLGIFCAHEGYHADRVAKQFKMDLDEAEVLAIAQGCADKNEQGSSADVWAYRGHKCLMASKVGEKVKAFIKKRMEEAQKQ

>AludOBP84a

MELKISKMATCRYYRTYLDIVVSLIIVVAISFTYAQTNSKNNGVDEQPSIEMTTAMADDGSAQGFDYKEVVRICNASFTVPLEYMQRFNETAELPNTTDKTGMCFMRCYMENAGLLRNWQLNRALIMQTMWPATGDSIPVCQNEGSRETCPCKRAYAIAKCLMIRALVDARNKPIV

>AludOBP99d

MPTAIGAAAASEEPVRRLRKAHKICAQEISPPATDESDNTQIAGAYLRCMTNSMGLWTDGKGYNAKRVAKFFSKQRNENEIVVVVDHCNQQHKQADLNLWAFEAYRCATAGRMGTWLGEYLLSAKI

>AludOBP99b

MRLFATTVASFALLHTLQRIMKFFIIAFLSLIALVLADHDHDHAGHSDYVVKTNEDLVEARKQCVSKLSIPDDLVEKYRKWEYPDDEKSRCFLKCIFEQFGLYDDEKGFDIHKIHHQLEGDKVDHSGDVHAKIENCAKEGADAADACTRAYRGITCFFKNNLSLVKQSVGSA

>AludOBP83g

MKLQLILLLACLALAAAKFQLRTAEDAIIAHEECRDEFRIPEDIYQKYLNYEFPAHKRTNCYVKCFVEKMGLFTEEKGFDEKAIIAQFTAKSSKNLAKISHGLEKCIDHNEHDSDTCTWANRVFSCWISVNRPIVRRTYIAK

>AludOBP50a

MKCLVSLLLLAALLGVNAYEFDDSTFNEYLFKELQSLQDDDIADDLPTHRARRETEVENSAEKEAKECAKHNWKKDMHCCKGSNVNDEQLELFMNVKKECIAELKGEPADDAYDPFNCDKMQQVKEQMICVAECVGKKFHSIDENGQFKRDVILEQLRKQIGDVQWKKDAIEGYVDKCLAEVKEKHEQLEKAGKLSEGCSRCPLAFSGCMWREFWNGCPAELHVDTPKCNKLRERVTKNDIQFFGKHLLFKYYPNSME

>AludOBP8a

MRSTTLAFIVFLFTFDAFSAADFEEKSEDDFLSASERCFERERLPSSYQRRFDNFEYPDEEIVHRYVHCIWKKLELWNDRTGFNVEHIAALYRDKANTEVLVPILSDCNRNAQNESKLKWCYDAFKCMLDSRVGQWFKEDVGRKLHDTKKANHVD

>AludOBP19d2

MTEAAIEDCYEDAAQKVKVEITDEGFEELLKGSRDNLMHNTKCLRYCIMRKNGLFNASNSLDKEKLVDIFEIIHPQVEKEKLLNVLQKCAEETEKETDNCERASVAAMCVLDELKGEGVTNI

>AludOBP56d

MKVFVVAIAFAFVAAAVAQDGLSLSEEQIQKVHALAGECIKETGTNEAAVRKLRAGDFSEVDENVKCFAKCFQERLGYVKNGSVDEAAVNKSLGPLAGEEKVKAVQAKCNGATGSNDCDAALERYKCYYGENVKNLS

>ZtauOBP99a

MKYIVAVLLAALVAMAAAEEYKIRNQDDLLKARKECMEAKKVPTEHIEKFKKFEFPDDEVTRCYIECIFNKFQLFSPTEGFKTQNLIAQLGQNKENKDAVKADIEKCADKNEQKSDSCTWAYRGFKCFISKNLPLVQESLKKN

>ZtauOBP56d-like

MKFFTVAVVLAFVAVAAAQDGGLNLSEEQKQKVHALGAECLKETGASEEAVRAVGKGDYSQVDGNVKCFAKCLQGKLGYFVDGKVNEPAVESSLGKLVGKEKIKAIQEKCNGVKGTDDCDTALLLHKCYATENASILV

>ZtauOBP19d

MKYFVVCLAICSLVIAHTEAQELLEKVKQIAEECKGQVGASDDDVTRMLKYEPATNDKAKCLQACIMKQFGILDDNNKLVEAGAMAYIKSLAAGDAELEKLSTEVYNECKNTPASSNECEYAEAVRVCTIENSKSKGIKLLPQ

>ZtauOBP56h

MKSFITIALLVVYSVVVLCHPNDPEMRKFIEDCNKEHNVSPKDFHDFMEGKLTTVPESLKCSSHCIMVKQGVMDESGNFKADVAKAKITEEKFAAAIDECKDLTGSTPCDTAMKITECMIAHK

>ZtauOBP24

MIFRVASQAVLLIAAAYCIQMVSSAATTKAMERDVGSDTEILRKCLHEVGSNDVVGELQKVARYSKWTSEEIPRFTRCLASMKRWFDADESKWNKQQIADDLGADMFNYCRYELDRYNEDSCEFAYTGLRCLKQAELYTLETYKNILSCATELNVTMKELQKYAAFPTKEVVPCLFQCLAEKMDFYTPTYEWNFDKWVKAFGPTRQDRTASNVCKVSPEQINTRDKCEWMYEEYNCLERLNYNTDGSYPMETTTLSAVITSKKPDAVEAAVKEAS

>ZtauOBP83a

MYALNSLLATLLWFGFMSNVIWAQKELRRDETYPPPELLKALQPVHDSCVAKIGVTEEAIKEFSDGDVHEDELLKCYMYCVFEETDVLHEDGEVHLEKILDSLPESMHVIALHMGKKCLYPKGDNKCERAFWLHRCWKEADPKHYFLI

>ZtauOBP99a-like

MPQAMKCTAFIFTILFSALIALINADFEETTEDDFLSAGERCFQRERLAASYQRRFDNFEYPDEEPVHRYVHCIWTELKLWNDRTGFNVEHIAALYRDKANTEVLVPILSECNRNTQNEPTLRWCYKAFKCVPNSRVGQWFKEDVSRKLHERRVGKSCRMSFFVMIKALHKNTHIFVDIRR

>ZtauOBP56a-like

MKSSISCCMLATVLLSLCVFSAEAGLRKPKKLTPELEAKFEVLTAWIAYRLNLKHAKEACVGEYGYSDELATNLVKIKVANPTDQQKCYVNCLYNKLVFYKDNAINKQAMKESLYEIVGEQRLMNIVNGCLNAGGTNACDKVYKFHACASPEFDKVRGDIFLPDE

>ZtauOBP19d-like

MKLLKICLILCVALISNAKCGSEEAKAAAEECKEEVGATDDDVEAMFKFESAGSMEAKCLHACVMKRFGLMNGDGKMDRDKAIEILENIASGDDEQQALGVEIVEACEGIEVDEDHCEAAEEYRSCMHDKGKENGFKMGRV

>ZtauOBP57c

MYQFGAHEKHATTITISSGNGMSLSGLTWSVLLVVFVMLVLPPAAVTLTPTSASRSFMEACQVKHNVTLEELDEFPTDPNPNEIDMKFKCYADCLLNGMGFMDKNGKLDAEAMHEWGILDDESYDNMLECKAANDLEDDPCEYSFGMMLCARMLSSGEEDYSGELDVEGDEK

>ZtauOBP99a-4

MKTQLILLLACVALVAGKFQIRTAQDALAAHEACHDEFRIPEDIYEKYLNYEFPPHKRTNCYVKCFVERMGLFTEEKGFDEKAIIAQFTAKSSKNLAKVSHGLEKCIDHNEHDSDTCTWANRVFSCWISVNRPIVRKTYIEN

>ZtauOBP99a-3

MKFFVALLSLIFAVVLASAHDHAGHSDYVVKTNEDLLRYRDECVSQLSIPADLVEKYKQWSFPDDEKTHCYLKCILEKFELFDAAKGFDVHKIHHQLEGDKVDHSDATHGAIENCAKEAAAAGDDACVRAYRGFNCFLKDNIQLVQAGVEKSAK

>ZtauOBP99a-2

MKFFIVILAVVALAYAEDDWVPKNAAEIKVIRQECFKDFPLSEEYIQKMKSFEYPDEEPVRKYLLCTAKKLGVFCEHQGYHADRVAKQFKMDLDEAEVLAIAEGCVDKNVEGSSADVWAYRGHKCLMASKIGDRVKAYIKKSVEEAKKQ

>ZtauOBP67-like

MSQASNMKADFGYFVVVFVCVCYASAEDESVDCTKPPRFIPLHTCCPVPDLSTEELMEQCAEFAKPPPPPPIGRGGPAKFEHSHHHPPHMRGLHPHPCLIECIFNKTEVIGENGEPDVDKFSALLDTTVKDNEEMAAIMEEAFETCTEKLSELKTKIAEKMSQNPEYAEKMANHRMQAACSPFGAMLMTCVNMETFKNCPASVWNDNTECNTVRDFINECKRV

>ZtauOBP19a

MSNKINRFVLVAVFIALVLRSDQVFGGATEEQMMAAGKLMRDVCLPKFNKITPEVADGIKEGNVPDTKDVKCYINCVMEMMQTMKKGKFLYESSLKQVDLLMPDSYKDEYRNGLGKCKDAAVGIKNNCDASYALLICMRDNISKFVFP

>ZtauOBP28a-like

MAKFILIAALCILSAAVTKAAFNKEEAIKTFMTRAEECRGEVGAADSDIQDLIAKVPAAGKEGKCLRSCLMKKYGAMDGNGKFVKSVADEHAQTYTDGDADKLKIAREIIDACADIAVPADHCEATEVYGKCFMEQAKAHGIEKFEF

>ZtauOBP19d-like4

MHTFKILPIFAVLSSIYTIAMVSADAKPPHYSALRVMAEAAIADCYEDAAQSVKVQITDEGFDEIVKGSRTNLSHNAKCIRYCIMRKNGLLNNDNSIDKENILQIFQIIHPQIEKDILLNVLQKCSQEADKQDNNCERAFMTSSCILQELSEDGVTDI

>ZtauOBP19d-like3

MNFCKVFLTTSVLAFLFATPISAGVTEEQMWATAKLMRDVCLPRFPKISIELANQLRDGNIPDNNKDVKCYINCVLEMMQTMKKGKFLYDASLKQVDLALPDSYKDDYRGGLLKCKDAAVGIKKDNCEAAYTILKCLRGEIKKFIFP

>ZtauOBP19d-like2

MKYFVVSLVICSLVISYSEAQELPMRLKKIVEECKARLGAGDDDVAAMFKYEPATNKQVKCLHACTMRLLGILDKNNKPFEAGAMAYIKSVTASNAELEKLLTEVYNECKYIPASKEGCEFSEAFRVCIIERSRAKGIHMLLH

>ZtauOBP84a-like2

MSNRNAFVALPYMIMLLYCSVRMSMQDRAKNNGDIFVQHKEQRECVAPILVQTNDSISNEGADVLHMCNSSFSIPMGYIVQFNTNGALSETVDKTGMCFLRCYFEKMGLIKAWQLNKDLIMQTMRPIKADSIEFCEPTAQQEVNACVRTYGIAKCLMKRGFDDTCNQTVAQNSFRAY

>ZtauOBP84a-like1

MVNHRQVELALSVLLLGALAGTTADAQSDATDNKLDKQQSMETTTPMAEAANASGFDFQDVVRTCNASFTPLEYIQRFNETAELPNTTDKTGMCFLKCYMEKTGLLRNWQLNSTLIRQTMWPATGDSIPVCQNEGSRETCPCKRTYAIAKCLMLRALVDARNKPIV

>ZtauOBP56h-like

MHTFYILVTMATLVTLAVCQFPADMEKFHKACMDEAKVTEDELKQFFQNGMKADEAKENIKCHTKCLMQKQGIWKDGVFNADAKVKELMQMPKLKGHEAEITQAMNNCKNEKGANECDTAFKITMCLKEFRSKNP

>ZtauOBP2b

MHALNSLLATLLWFGFPSNLIWAQKELRRDETYPPPELLKALQPVHDSCVAKIGVTEEAIKEFSDGDVHEDELLKCYMYCVFEETDVLHEDGEVHLEKILDSLPESMHVIALHMGKKCLYPKGDNKCERAFWLHRCWKEADPNQYLPI

>ZtauOBP2a

MHALNSLLATLLWFGFLSNVIWAQKELRRDETYPPPELLKALQPVHDSCVAKIGVTEEAIKEFSDGDVHEDELLKCYMYCVFEETDVLHEDGEVHLEKILDSLPESMHVIALHMGKKCLYPKGDNKCERAFWLHRCWKEADPNQYLPI

>RpomOBP56h-like

MRALYVLIGLTMLVASVTFVMCQSSDETDKWNRIACLSESNTTEDEVKKFFENGMKASEATNSIKCHVKCLLEKQGILNKSVYNADVAIKQLMKIPAMKGHETEVKQAVNSCKNEKGANYCDTAFKICMCIKEFKAQM

>RpomOBP84a-like

MTSINACIQLTLTFLLLYCCNMVRAQSRPKDNGDIYVQHKTQSVMAGITADQTDIPIAAQTYGSNTFNVEEVMSTCNASFSIPMDYIVQFNTTGELSDTTDKTGMCFIRCFLEKSGLIKNWHLNKDLIMQTMWSIIADSVELCESESESEVNACVRTYAIAKCLMKRTLEAAGNQTLT

>RpomOBP56h-like1

MKALYVLVGLTALVASVTFVMCQSSDEMDKWNRIACLSESNTTEDEVKKFVENGMKANEATNNIKCHFKCWLEKKGIFNISVYNADAAIKQLMKIPAMSGHEIEVKQAVNRCRNERGASDCDTAFKICMCIKEF

>RpomOBP84a-like1

MVRAQSRPKDNGDIYVQHKTQSVMAGITADQTDIPIAAQTYGSNTFNVEEVMSTCNASFSIPMDYIVQFNTTGEMSDTTDKTGMCFIRCFLEKSGLIKNWHLNKDLIMQTMWSIIADSVELCESESESEVNACVRTYAIAKCLMKRTLEAADNQTLT

>RpomOBP56h-like2

MKALYVLVGLTALVASVTFVMCQSSDEMDKWNRIACLSESNTTEDEVKKFVENGMKANEATNNIKCHFKCWLEKKGIFNISVYNADAAIKQLMKIPAMSGHEIEVKQAVNRCRNERGASDCDTAFKICMCIKEFKECEFKSTADFFAV

>RpomOBP56h-like3

MKALYVLIGLTALFASVTFVMCQSSDEMDKWNRIACLSESNTTEDEVKKFVEIGMKANEATNNIKCHFKCWLEKKGILNKSVYNADAAIKQLMKIPAMSGYETEVKQAVNRCRNERGASECDTAFKICMCIKEFK

>RpomOBP57c-like

MYQFEVNRKKAIAKEATHSNGLRMALPIPAWLMLLTAVMLFLSSLGGVNATPAASTKSFMEACQERNNITQEELDQFPDDPSPEDVEQKFKCYANCLLDGMGILDENGKIMADSMHEWGILSDESYENMIECKAANDMEDDPCEYSFGIVICARVNIEHHSDEIEPIEEERRK

>RpomOBPLush

MLFGLNIIRNFLPLLACIIGVSAVTMQQFETSLDMMRNGCAPKFKIPVELLDRLRDGDFVENNSELKCYTRCVAQLAGTLTKKGDFSVQKALAQIPIILPPEMQDTAREAMNACKEVQKNYKESCERVFYTTKCVRDYAPDTFKFP

>RpomOBP99a-like

MKRTTFAYIIFLFTFCMPISADFEEKTEDDFLTASEHCYERERLPASYQRRFDNFDYPNEEPVHRYVHCIWTELKLWNDRTGFNVEHIAALYRDKANTEVLVPILSDCNRNAQNEPTLKWCFNAFKCVLNSRVGQWFKEDVGRKLQDTKTTNHLG

>RpomOBP28a-like

MKYFVVFLFICGLATSYAQLEEVVKKAQKIAGDCKEEVGATDADVEAMFKHEPAGNDKAKCLNACVLKNFGFLDADNKLVKDVGVDFLKSISGGDAAYEKLGTEILDACKDTPAGGNNCETSEALRTCLIEKSQEKGFKLPF

>RpomOBP19d-like

MKSYFICLVVCAVFTSYVECDLEKFKAVAEECKEEVGATDEDVETMINHEPAGTPEAKCLHACAMKKFGLMNDDGKLDKERAIEILKIIKSDDEEQQNLGVEIVDACADIDVNEDHCEAAEEYRTCMHAKAAENGFTLGRV

>RpomOBP19d-like1

MRVLSVLTICLAISLLPVSMLADPRPPHYDSLRTMAEAAVEDCYEDADQNVKVEITDEGFDELLRGSRDNLLRNTKCLRYCIMRKNGLINAGNSIDEDRVVEIFKIIHPQIEKEKLIKVLQKCSEETEKQIDNCERAFAAATCVLGELKGQGVTDI

>RpomOBP19a-like

MLSKFIAFILITVFIVLALQTKPISSGATEEQMFAAGKLMRDVCLPKFPKVSTEVADGIKLGNIPDTKDVKCYINCVLEMMQTMKKGKFLLESSLKQIDLLMPDDYKDDYRAGLQTCKDAVNGIKNNCDAAYALLICIRGEIKRFIFP

>RpomOBP56d-like

MKYFATVIVLTFVAVAVGQDVLKLSAEQKEKVHLLAGECAKETGASKEAILALRQGDFSQVDDKVKCFSKCFQERLGYLVNGVVNEEAVQKSLGPLAGEEQVKAVQAKCNGVKGANDCETAFELYKCYYGEKATVLA

>RpomOBP68

MKASFVYFAVICVFVHYANAQDTTSVDCTKKPRHVPPHMCCPVPDLTTDELMQKCAQFAGPPPPRPPSSEEPPMRGHPHPHGPHMHPCFMECIFNQTEVIGEDGELNADKFEQLLNTVVKDDREMAIIMKESFNACSANANELKTKIAEEIEKNPEFSKQKFQPLCSPFSAMTMGCIKMQTFQNCPSSTWNDNEECNAFRKFIMECKKPK

>RpomOBP19d-like2

MKSYFICLVVCAVFTSYVECDLEKFKAVAEECKEEVGATDEDVETMINHEPAGTPEAKCLHACAMKKFGLMNDDGKLDKERAIEILKIIKSDDEEQQNLGVEIVDACADIDVNEDHCEAAEEYRTCMHAKAAENGFTLGRV

>RpomOBP28a-like1

MAKLVLIVAFCILNAAVSKAAFNKEEAIKNFMTNVEECRGEVGAAASDIEDLIKKAPAASKEGKCLRSCMMKKYGVMDDGGKFVKSVALEHASTFTDGDAEKMKTANEITDACAGIAEPDDQCEAAEVYSKCFMEQAKAHGLDKFEF

>RpomOBP19d

MRFHKREDYYKSESFLISTSEFTDQQAYKKFIKKMTSLRNKMLYTAILCLAAIQSLNALDTNPYKLSGKRKPLMTRQNSLELGDYKRTKRQLTEPMQEFQDFITNSKTQCANGMGFSADELQKSLLYEDQPTRKEKCLIECILKRMEVMDKDNTLSTAAIGRIANIVGNNNPLITSIAMATADNCKKFINAEDSCERAHQINICIATEMKMRKIKLIY

>RpomOBP69a-like

MNTKQFVFLLIVYQCCFLAIVKPLEVPKHMKSGAKKLTNTCMKETGATEEMFTEAKRTEQLPGDNRLKCFMHCMLDKIGLIDNENIIHLDNLLEIMPPEFLPMIEQLHTTCGTKSGADGCETAFLTVDCYIKTNPVILKLLVVTVSD

>RpomOBP56h-like4

MKSFITIAFVLISSVVVMCEPPNPQMRQYIEDCNKEHNVSPKDFHEFNEGKLSSPSEDLKCSIHCFMVKQGSMDEAGTFKPDVAKTGMPNDDKFAAAVDACKDKTGSSACETAFKITQCLISHK

>RpomOBP56a-like

MKSTTSYCILVAVLFCLCVSNTEAFGRKAKKLNPELEAKFDVLTAWIAYRLNLKHAKEACIGEYGFTDELATSLVKVQVANPSDREKCYVNCLYTKLVFYNNNQINTQAMKESLIEIVGEERLLQIVNSCLNAGGANDCDKVYKFHACASPEFDKVRGDIFQPDE

>RpomOBP99a

MKYIAAVLLALFALAAAEEYKLRNQDDLLKARKECVEAKKVPVDHIEKYKKFEFPDDEVTRCYIECIFNKFQLFSPTEGFKTHNLIAQLGQSKENKDAVKADIEKCADKNEQKSDSCTWAYRGFKCFISKNLPLVQESLKKN

>RpomOBP99b

MKYFIAFLSLIVVVLAHHDNSEHHADYVVKSKEDLAHARDHCVSKLTIADDLVEKYKKWEYPDDEKTHCYLKCIFEELGLYDDEKGFDVHKVHHQVAGDKVDHSDDLHKTIENCAKEGADSDDSCVRAYRGGMCLINNNLTLVKQSFVSD

>RpomOBP99a.1

MKYFIAILAVVALAHAEEEWNVKTAADIKVIRQECIKEFPLSQEYIQKMKNFEYPDEEPVRKYLLCTAKKLGIFCEHQGYHADRVAQQFKMDLDEAEVLAIAESCVDNNQQGSSPDVWAYRGHKCLMASKVGEKVKAYIKKRTEDAQKQ

>RpomOBP99a.2

MMKLLQLILLLAYVALVTARFQIRTAEDARIAHGECRAEFNIPDEVYEKFLNYEFVAHRRTNCYVKCFTEKMGLFTEEKGFDEKAIIAQFTGKNTKNLAKVSHGLEKCIDHNEHESDTCTWANRVFSCWISVNRPIVRKAYIQN

>RpomOBP19a-like1

MWATAKLMRDVCLPRFPKISIELADQLRDGNIPDNNKDVKCYINCVLEMMQTMKKGKFLFDASLKQVDLILPDSYKEDYRAGLMRCKDASVGIKNNCESAYTILKCLRGEITKFIFP

>RpomOBP56d-like1

MKLFAAAIVLAFVAVTVAQNAPRLSLDQMLKVIALADECTKETGTSKEAILALQQGDFSQVDDKVKCFSKCFQERLGFLVNGAVNEEAVQKSLGPLAGEEKVKAIQAKCNGVKGADDCDTAFEWYKCYYEEKGLA

>RpomOBP56a-like1

MKLTISCCILATILFFLCATNTDAFVRKPKKLKPELEAKFDVLTAWIAYRLNLKHAKEACIGEYGFTDQLATSLVKVQVENPSDREKCYVNCLYTKLIFYNNNQINTQAMKESLSEIVGEERLLQIVNSCLTAGGANDCDKVYKFHACASPEFDKVRGDIFLPDE

>RpomOBP68-like

MECIFNKTEVIGEDGELQGDNFMELLKTAVKDDEEMIAVMEESYDTCFEKSTDAKAKIADKMKKDPEFAQRITKPKFQSCSPFGAMIMTCINFETFQNCPTSAWNDSEECNAFRKFVTQC

>CstyOBP14

MKKYILIEAAASVVVAVLLLMPILSFAQKPRRDENYPPPDFLKKFSIIHDVCVEKTGVTEAAIKEFSDGDIHEDPALKCYMNCIFHEVNAVDDDGEVHYDKMRRLIPDSLIEFIKPIIDACETHIPKGGTQCDRAWSWHVCFKESDPVHYFLL

>CstyOBP11

MYRLQFYLIYFSLIFLGSIETLQKAKDNGDTYIVKEEKFLKDIKQSTEPASLTTTSSPGNSNIVNLDDMVSTCNTSFSIPMQYYVKFNKTGELPDLVDKTGMCFIRCVFEKSGFIENWKLNAEKIRANIWPAMGDTIEVCEKEEAKEKNACVRTYAIAKCLTLRSLVDARNNPV

>CstyOBP27

MKVFVAILALVACVSAEEWTVKTDDQIKEISTECLKEHPLSAEQINKIKNFVYPDEEEVRQYLLCAVVKSGVFCTHEGYDAGRVAKQIKMDLDEEEVKKAVEDCIAKFPKGDKANDVVVLETHTCLMSSTTGEKLKELLKKRRETAEKHE

>CstyOBP26

MKAATTTALLILSFNLVILTAAQSKTSDEFPEKSEKCFQQEHVPQAYKARFQSFQYPNEEIVHKYIHCVSTELGIWDNTNGFNVEKISQQYRGRANDEVVIPVISKCNQDNQNRNKDLWCYRSFLCILNTQVGEWFKEDVRRKQQA

>CstyOBP25

MKVFVAILALVACVSAEEWTPKNGEQIKEVRIECLKEHPLSPEQMNKMKNFEFPNEEAVRKYLLCTAEKMDIFCSHQGYHADRIAKQFKMDMDEEEVQKLVEDCIAKFPKGDKPNDVVAYEGHSCFMSSAIGDKLKNYIKKRHEAAQQHE

>CstyOBP23

MFKSVISLILLSASLQCYTAAVIDCQRPPQLVDPAKCCLDGGRDEVTEVCAQRMGITGGPSDAPPTVETATCLAECILSESKYMQKPETLELSLIKSDLQSKFANDTIYAQTMAEAFQKCQPSAQRKMKAFKQIPLGNVALQRGCSPFAGMVLGCTYMEYFKNCPAHRWTDNEECSLAKQFVTQCSLGA

>CstyOBP20

MKFLVVFAVLVLAVTNIRAQLTKVEAIAIATGCKEETGASDADFEAMLKHQPADSKEGKCLRACTFKKLGVIGDDGKMLKDAAVELSKSFVKDEEKKKLVAGAIEACNDLKVSADHCEAAEEYGQCLKKEFDSKGISAADDLL

>CstyOBP16

MKVYITLAVVCLIASAVAHHELSEEEKAKIKVHFQECVKQENVSEEEATKLRNKDFANATPGMKCFGACFFEKVGILKDNVVQEDVVLAKLVPHYGEENVKKVMEKCKNEKGADRCETGFKIYECVEKAKAELGH

>CstyOBP15

MKFLIVSGFLILAACSIRAELTKEEAIAIATDCKEEAGASDADFEAMVTHQPAISPEGKCMHACALKKFGVLSDEGKLLKDAALELSESLIKNEEKKALIAEIVETCDQLEVSDDHCEAAEEYGQCWRNEFEAKGISPDEDLV

>CstyOBP12

MKVFFVILVLTVAALADHHEGHDHHDHDHHVHHESHDYVVKHHDDLVKFRDECSTKLKISPELMEKYKSWEYPDDEATHCYMKCIFEHFGFFDEHKGFDVHKIHHQLVGEHVTVDHNDETHHKIEHCADKNTQGSDACTWAYRGGMCFIRSNLQLVKGSVHKH

>CstyOBP10

MAFKAFYRLQLFHTILIVLSLMATWTRAQQPRRDSDWPPPSILKMAKPFHDICVEKTAVTEEAIKEFSDGQIHEDEALKCYMNCLFHEFEVVDDNGDVHLETLFKNIPGSIRDLLMKASENCVHPEGDTLCHKAWWFHQCWKKADPVHYFLI

>CstyOBP9

MKFWIIATIFLVITILMVSNNNAFEIPEHLKKHAKKLHKRCQNQTETPEDVIRESLTGVLPKNKNFECYVQCLFDIIGIMDENNIIRIDLLMQVLPDEMHSTLTRLAEVCGTKEGNDKCSVAYNTLQCYVDNNPLMIKNNLEFLFD

>CstyOBP8

MKLFILCMALVLAAVEVKSDKKEELRNVEKVCREENNITEEELQSAVKSGFKEEPREALKCYMKCILEKLGQWKNGAFEENVAKKFLQDIPALKDHQDIIEKTLNECKIQKGVNECDTAYLISKCFMERNPRVM

>CstyOBP7

MKASIAILIALFALVSAEYKLRNQEDLMKARKECMEAKKVSPELIEKYKKFDFPDDEVTRCYIECIFEKFQLFDPKDGFKNENLITQLGHGKENKDEVKADVEKCADKNEQKTDSCAWAFRGFKCFISKNLPLVMESLKKN

>CstyOBP6

MAKLFVTLAILSVFGGVLVKGFDKKAAVTAFMAKMDDCKAEVGAKDSDVEELVGKKPSSTMEGKCLRSCLMKKYEVMDNTGKFVKDVAVTHAHKYTDGSEDRMKIAHEIIDACSSIEVADDHCEAAEQYGKCFKEQAVAHNIKDDFDY

>CstyOBP5

MQFLFVFTFLILAACNIKAELSKDEATAIALACKEEAGASDADFEAMIKHDPTETPEDKCMRACVFKKFGVMSHDGKMIKDAALELSKSLMKDAEKREIVVGVIDTCEKLVVSDDHCEAAAEYRHCWRKELAGKGVSSAEDLF

>CstyOBP4

MNLLPKLLICFTIIALSYADEDGMSVKDIAEALISFGEDCDPKPEEEHIIEVVKNVPDAQYTSKCFRHCLMVQFEIIQEGSQVMDKEKTLDMMGSMFSDRKDDLGEIIDECNSKNEAIAEKCENAHAHGMCMLDLMKERGFDIPDLKDE

>CstyOBP3

MILKQYLVFLIGAILCLCKVSDVIGGATEEQMWAAGNLMRDVCAPKFPKVTKEIADGIREGNLPNEKDPKCYVNCILEMMQTMKKGKFLYEGSLKQVEILMPDHFKEEYRTGLAKCKDAAVGIKNNCDAAYAIFTCLRAEITRFVFP

>CstyOBP2

MKLFSKLIILKFAILCLSCIQLTSAITMEQFEQSLDMMRNGCAPKFKVSLEQLDKLRNGYFDESSSELKCYTKCVAQLAGTVTKKGDFSISKATAQIPIILPPEIQETAKAALASCKEIQKDYKESCDRIFYVTKCVRDFAPDIFKFP

>BcucCSP1

MLRFIAATVLICAVYHVATTSAAPHPPTTAAPLVGNQPAYDTKFDNIDLDEVLNQERLLRNYIKCLENTGPCTPDSKMLKEILPDAISTDCAKCSEKQKLGSAKVTHFLIDNRPEDWARLEQIYDPSGNYRLAYLAAKDNKGDGAEKPTEAVTKALA

>ZtauCSP3

MLRFIAATVLICAVYHVATTSAAPHPPTTAAPLVGNQPAYDTKFDNIDLDEVLNQERLLRNYIKCLENTGPCTPDSKMLKEILPDAISTDCAKCSEKQRLGSAKVTHFLIDNRPEDWARLEQIYDPSGNYRLAYLAAKDNKGDGAEKPTEAVTKAEA

>BcorCSP3

MLRFVAASVLICAVYHVATTSAAPHPPTTAAPLLANQAAYDTKFDNIDLDEVLNQERLLRNYIKCLENTGPCTPDSKMLKEILPDAISTDCAKCSEKQRLGSAKVTHFLIDNRPEDWARLEQIYDPQGNYRLNYLAAKDKGDGMEKTTEAVTKTQA

>BdorCSP2/BdorCSPOSD

MLRFVAASVLICVVYHVATISAAPHPPTTAAPLVANQAAYDTKFDNIDLDEVLNQERLLRNYIKCLENTGPCTPDSKMLKEILPDAISTDCAKCSEKQRLGSAKVTHFLIDNRPEDWARLEQIYDPQGNYRLNYLAAKDKGDGVEKTTEAVTKTQA

>BdorCSP3

MLRFIAASVLICAIYHVTITSAAPHPPTTAAPLVANQAAYDTKFDNIDLDEVLNQERLLRNYIKCLENTGPCTPDSKMLKEILPDAISTDCAKCSEKQRLGSAKVTHFLIDNRPEDWARLEQIYDPQGNYRLNYLAAKDKGDGVEKTTETVTKPQA

>BtryCSPA10

MLRFIAASVLICAVYHVATTSAAPHPPTTAAPLVANQAAYDTKFDNIDLDEVLNQERLLRNYIKCLENTGPCTPDSKMLKEILPDAISTDCAKCSEKQRLGSAKVTHFLIDNRPEDWARLEQIYDPSGNYRLNYLAKDKGDGVEKTTEAATKTQA

>BlatCSPA10

MLRFIVASVLICAVYHVAITSAAPHPPTTAAPLVANQATYDTKFDNIDLDEVLNQERLLRNYIKCLENTGPCTPDSKMLKEILPDAISTDCAKCSEKQRLGSAKVTHFLIDNRPEDWARLEQIYDPPGNYRLNYLAAKDKSDGVEKTTEAVTKPEA

>BoleCSPA10

MLRFVAALVLICTVYHVATTTAAPHPPTTAAPLLANQAAYDTKFDNIDLDEVLSQERLLRNYIKCLENTGPCTPDSKMLKEILPDAISTDCAKCSEKQRLGSARVTHFLIDNRPEDWARLEQIYDPQGNYRLAYLAEKDKSDGMEKTTEAVTKPQA

>CcapCSPA10

MFRLIVALALILSIYHAPSVHGAPHPPTTAAPLVANQGSYDTKFDNIDLDEVLSQERLLRNYIKCLENLGPCTPDSKMLKEILPDAVSTDCAKCSEKQKLGSAKVTHFLIDNRPEDWARLEQIYDPSGNYRLAYLAEKDKGNSQSDTGEQTPSEAVTKTNEA

>RzepCSPA10

MRFLVAFVLIYIAYYVAGIGAAPHPPATTSAPLVANQATYDTRFDNIDLDEVLSQERLLRNYIKCLENTGPCTSDSKMLKEILPDAISTDCVKCSPKQKSGSAKVTHFLIDNRPEDWARLEQIYDPQGSYRLAYLAEKAKNGDDKQNTTEAETKA

>PutiCSPOSD

MLRFIGICILFSILYNQVRVNGAPHAQTTTAAPAPATQLVSNQGTYDTRFDSIDLDEVLSQERLLRNYIKCLESTGPCTPDSKMLKEILPDAISTDCAKCSPKQKSGSAKVTHFLIDNRPEDWVRLEQIYDPQGNYRLAYLAEKMKDGTNKPNGDGTEMETKS

>LserCSPA10

MSHLICLGVVLTLVTLIQAVPHPPATTAAPLKQTYDNKFDNIDIDEILGQERLLNNYIKCLEGLGPCTPDAKMLKETLPDAIMTNCAKCTERQKYGSDKVTHHLIDNRPADWNRLEKIYDPEGSYRKAYLMQKENTTTKVDKEEENPAKD

>CstyCSPOSD

MSHLIWLGVLFALVVIIQAVPHPPATTAAPLKQTYDNKFDNIDLDEILGQERLLNNYIKCLEGLGPCTPDGKMLKETLPDAIMTNCAKCTEHQKYGSDKVTHHLIDNRPKDWERLEKIYDPEGSYRKAYLMQKENTTTKDDNASKD

>LcupCSPA10

MSHLICLGVLLSLVVLIQAIPHPPATTAAPLKQTYDNRFDNIDIDEILGQERLLNNYIKCLEGLGPCTPDAKMLKETLPDAIMTNCAKCTERQKYGSDKVTHHLIDNRPEDWNRLEKIYDPEGSYRKAYLMQKENTTIKIDKDENPAKD

>DmelCSPOSD

MGQPGFRRAIGHFSLVVALMCTTCFQVEGLPHPPATSPSPMMERMVEQAYDDKFDNVDLDEILNQERLLINYIKCLEGTGPCTPDAKMLKEILPDAIQTDCTKCTEKQRYGAEKVTRHLIDNRPTDWERLEKIYDPEGTYRIKYQEMKSKANEEP

>RpomCSPA10

MRFLVAFVLIYIAYYVAGIGAAPHPPATTSAPLVANQATYDTRFDNIDLDEVLSQERLLRNYIKCLENTGPCTSDSKMLKGFSYFRDTPRRYINRLRQMLAEAEVWLSEGDALLDRQSAGGLGALGADLRSARQLPLSLLSGEG

>DmelCSPA10

MGQPGFRRAIGHVSLVVALMCTTCFQVEGLPHPPATSPSPMMERMVEQAYDDKFDNVDLDEILNQERLLINYIKCLEGTGPCTPDAKMLKEILPDAIQTDCTKCTEKQRYGAEKVTRHLIDNRPTDWERLEKIYDPEGTYRIKYQEMKSKANEEP

>BcucSNMP1

MTTAKIAKTLFPGPIPWYSTKMLHWSLIVSFLGLLTAALGAYCGWFLFPTMVDKKVEESVIIADGSEQYKRFVQLPQPLTFKVYIFNVTNAHKIQHGALPIVEEIGPYVYRQYRRKKVKHFSRDGSKISYVQDQHFEFDEEASAPYTQSDHIVVLNMHMNAFLQVFEREITDIFQGFANRLNHRLNRTPGVRVLKRLMERIRGKPPYKWMYNIFNEIFKQVKSVLTIAENDPGLSLLLVHLNANLKAVFNDPKSMFLDTTVREFLFDGVRFCINTNGIAKAICNQIKEGGSKTIRELSDGSLAFSFFNHKNGTGNEVYEVHTGKGDAQRVLEIQKLDDSHNLQVWLNGAEGETSMCNQINGTDASSYPPFRKRGDSMYIFSADICRSVELFYQSDIQYQGIPGFRYSIGENFINDIGPEHDNECFCVDKLANVIKRKNGCLYAGALDLTTCLDAPVILTLPHMLGASNEYTKMIRGLRPDAKKHQTFVDVQHLTGTPLQGGKRVQFNMFLKSINRISITENLTTVLMPAIWVEEGIQLNSEMVAFFKKKLINSLKTLNIIHWASICGGVGVAAICLIYYVIQRRKPEAEVAPLK

>BcucSNMP2

MKTMRVQRHKLLIASVSAMLFGVIFGWVGFPKILKMMIKKQVSLKPGTEIRDLWTQTPFPLHFYIYVFNITNPEEVINGEKPNLQEVGPFVFDEWKDKYDLADDPIEDSISYNMRNTFYYNEKASKGLTGEELITIPHPLLVPISVVVQRERAAMLDLVAKAINIVFAGEKAVITVKFMDLFFRGLYVDCSSPEFAAKALCTAFYTGEVKQARQVNSTHFLFSFMGNNNHTDGGRFTVCRGVKNFSKLGKVIRFGDEPTLDMWAGDECNEFIGTDSTIFAPFMTKEQGLWAFTPDVCRSFGAVYRRKSSYHGMPAMRYHMDLGDIKADPSLHCFCDDPEDIDSCPPKGTMNLEPCVGAPIIASMPHFYNGDPRLLEEVNGLSPNEHDHAVFIDFELTSGTPFQAAKRLQFNFDMEPVEKIEPTKNLRKMIFPIFWVEEGVALNKTFTNMLKYTLFLGLKFNSALRWSLITMALVGLMSTCYLYYKKSDSLDITVPPKAITELTNKVEDVKPVPDKRSHPPTIAQADLSNPRDTRNTF

>BcucSNMP3

MFKKVLIGSAIALVLGIFIGFVGFPKLLKKMIKGQLNLKPGSEPRQMWEKFPIAVNFSIYVFNVTNPDEVQNGGKPRLQEVGPFVFEEWKDKYDLEDFEDEDAVAYNMRNTFIFRPDLGLSGEDLIVMPHPLIQVMSIAVKRDKEALINMIAEGIGALFKPTTPFVRAPFMDIFFRGIDVDCSVEHFAATAICLNFHTGAVKGAEKVNETHFKFSLMGGANHTDAGRYKVARGVKVSHDIGRVLEFDDSEELSVWDGDECNQFRGTDTTIFAPLLKPEEGLWSFAADLCRSLGAEFEKKTSYAGIPAYYYTIDLGDPKNDPDKHCFCKDYPDDCPPKGTMDLTLCNEAPMIVSLPHFFKADPKLVADVDGLNPQEDKHGVFIIFERISGTPLSAAKRLQFSLSVMPVPEIEVMKNLRTLTMPLFWVEEAASLDKTWTDMLKKKVFLVIKINNVFKWISTIFGALGLIVSLYMLYGKNQLTTTDVTPTTEVNTIDKH

>BcorSNMP2

MFHWSLFVSLLGLLTAALGAYCGWFLFPNMVDKKVEESVIIADGSEQYKRFVQLPQPLTFKVYIFNVTNAHKIQQGAIPIVEEIGPYVYRQYRRKKVKHFSRDGSKISYVQDQHFEFDEEASAPYTQSDHIVVLNMHMNAFLQVFEREITDIFQGFANRLNHRLNRTPGVRVLKRLMERIRGKRKSVLTIAENDPGLSLLLVHLNANLKAVFNDPKSMFLDTTVREFLFDGVRFCINTNGIAKAICNQIKEGGSKTIRELSDGSLAFSFFNHKNGTGNEVYEVHTGKGDAQRVLEIQKLDDSHNLQVWLNGSEGETSMCNQINGTDASSYPPFRKRGDSMYIFSADICRSVQLFYQSDIQYQGIPGFRYSIGENFINDIGPEHDNECFCVDKLANVIKRKNGCLYAGALDLTTCLDAPVILTLPHMLGASNEYTKMIRGLRPDAKKHQTFVDVQHLTGTPLQGGKRVQFNMFLKSINRISITENLTTVLMPAIWVEEGIQLNSEMVAFFKKKLINSLKTLNIIHWASICGGIGVAAICLIYYVIQRKPEAEVAPLK

>BcorSNMP1b

MFKKILIGSAIALVLGIFVGFIGFPKLLNKMIKGQLNLKPGSEPRQMWEKFPIALNFSIYVFNVTNPDEVQNGGKPRVQEVGPFVFEEWKDKYDLEDFEDEDAVAYNMRNTFIFRPDLGLGAEELIVMPHPLVQIMAIAVKRDKEALINMISEGLQALFKPTTPFVRAPFMDIFFRGIDVDCSIDHFAAKAICLNFHTGAIKGAEKVNATHFKFSLFGGANHTDAGRYKVARGVKVSRDIGRVLEFDDSDELSVWDGDECNQFRGTDTTIFAPLMKPEEGLWSFAADLCRSLGAEFEKKTTYAGIPAYYYTIDLGDPKNDPDKHCFCKDYPDDCPPKGTMDLTLCNEAPMIVSLPHFFKADPQLVADVDGVDPVEEKHGVFIVFERISGTPLSAAKRLQFSLSVMPVPEVEVMKNLRTLTMPLFWVEEAASLDKTWTDMLKKKVFLVIKINNIFKWMSTIFGALGLAISLYMLFGRNQITTTNVTPTTETSTIDKH

>BcorSNMP1a

MRVERNKLLIASVAAMLFGVIFGWVGFPKILKMMIKKQVSLKPGTEIRDLWTQTPFPLHFYIYVFNITNPDEVLNGEKPNLQEIGPFVFDEWKDKYDLVDDPLEDSISFNMRNTFYFNEKDSKGLTGEELITIPHPLLVPISVVVQRDRAAMLDLVAKAINIVFAGQKAVITVKFMDVFFRGLYVDCSSTEFAAKALCTAFYTGEVKQARQVNSTHFLFSFMANNNHTDGGRFTVCRGVKNVSKLGKVIRFGEEPTLDIWGGEECNEFIGTDSTIFAPFMTKEQGLWAFTPDVCRSFGATYKRKSSYHGMPAMRYHMDLGDIKADPSLHCFCDDPEDVDSCPPKGTMNLEPCVGAPIMASMPHFFNADPKLLEEVNGLSPNEKDHAVFIDFELTSGTPFQAAKRLQFNMDMEPVEKIEPTKNLRKMIFPLFWIEEGVALNKTFTNMLKYTLFLGLKFNSALRWSLITMALVGLMSTSYLYYKKSDSLDITVPPKAIKELANKVEDVKPLPPEDKKPVPPIAQADLSNRRDAMDRF

>CstySNMP3

MILLLLPIKIYKLLAIGCVVLITVAVISILGFPKLMKMMIKKEINLKPNSETRQMWQEFPIPVIFSIYVFNVTNPKAVENGDKPQLKEMGPFVFEEWKDKYDIADIEEEDAVEFNMRNTFILRPDLGLSGEEIVTMPHPLIQFVAISIKREKAAMIEMIAEGLQDIFKPTSAFIQAPFMDIFFRGFDVDCSDSDSFAASAICLNFHTGSVKGARQVNETHFKFSLLGASNHSDAGRFKVSRGIKNNRMLGQVLEFEGDEELNVWPGEECNKLKGTDSTIFAPLMKPSEGLWTFSADLCRALGPQYQKKVKYNGLPALRYTMDFGDVKNEPENQCFCKDYPDNCPAKGTMDLSLCNETPMIASMPHFLNGDAKLLQDVEGLQPDEMKHGIHIDFEIISGTPLSIAKRLQFNLDVEPIEQLAVMSKLKPLVMPLFWVEE

>CcapSNMP2

MTTAKIAKSLFPATVPWYSSKMIHWSLIVSFMGVLVAALGAYCGWFLFPNMVDKKVEESVIIADGSEQYKRFVQLPQPLTFKVYIFNVTNAQRIQQGAIPIVEEIGPYVYRQYRRKKVKHFSRDGSKISYVQDQHFEFDEEASAPYTQSDRIVVLNMHMNAFLQVFEREITDIFQGFANRLNHRLNRTPGVRVLKRLMDRIRGKRKSVLTIAENDPGLALLLVHLNANLKAVFNDPKSMFLDTTVREFLFDGVRFCINPNGIAKAICNQIKEGGSKTIRELSDGSLAFSFFNHKNGTGKEVYEVHTGKGDAQRVLEIQKLDDSHNLQVWLNGSEGETSMCNQINGTDASSYPPFRKRGDSMYIFSADICRSVELFYQSDIQYQGIPGFRYSIGENFINDIGPEHDNECFCVDKLANVIKRKNGCLYAGALDLTTCLDAPVILTLPHMLGASNEYTKMIRGLRPDAKKHQTFVDVQHLTGTPLQGGKRVQFNMFLKSINRIAITENLTTVLMPAIWVEEGIQLNSEMVAFFKKKLINSLKTLNIIHWASICGGIGVAAICLIYYVIQRRKPVAEVAPLK

>CcapSNMP1

MYKKILIGSAIALVFGIFVGFIGFPKLLKKMIKGQLNLKPGSEPREMWEKFPIALNFSVYVFNVTNPDEVQNGGKPHLQEVGPFVFEEWKDKYDLEDFEDEDAVAYNMRNTFIFRPDLGLSGEDFIVMPHPLIQIMAIAVKRDKEVMINMISEGIEALFKPTTPFVRAPFMDIFFRGIDVDCSVDHFAATAICLNFHTGAVKGAVKVNETHFKFSLMGGANHTDAGRYKVARGVKVNRDIGRVLEFDDSDELSVWDGDECNRFRGTDTTIFAPLMKPEEGLWSFAADLCRSLGAEYEKKTKYAGIPAYYYTIDLGDPKNDPELHCFCRDYPDDCPPKGTMDLTLCNEAPLIVSLPHFFKADPQLVADVDGLNPQEEKHGVFIIFESISGTPLSAAKRLQFSLSVMPVPEVEVMKSLRKLTMPLFWVEEAASLDKTWTDMLKKKVFLVIKINNIFKWMSTIFGACGLFVSLYMLYGKNQITTTNVTPTTEVNTIEKH

>CstySNMP2

NQIKDGGSKTIRELKDGSLAFSFFNHKNGTGHDVYEVHTGKGDAKRVLEIQKLDDNHHLQVWLNSSDNNGTSKCNQINGTDASMYPPFRQKGDSMYIFSADICRSVQLFYQKEITYKGIPGYRYSIGENFVNDIGPEHDNECFCVDKLTNIIKRKNGCLYAGALDLTTCLDAPVILTLPHMLGASNEYTKTIRGLNPDAKKHQTFVDVQHLTGVPLQGGKRVQFNMFLKSIN

>CstySNMP1

MQINRNKLLIVSGAVFVFAIMNGWLIFPKILKFILKKQVNLKPGSDVRELWADTPFPLHFYFYVFNITNPNEFLNGAKPNLQEIGPFVFDEWKSKYDLEDDEEEDTVTYHMRNTFIFNAKASAPLTGEEVITMIHPLIAPIAVLVLRERAAMMELIVKAIDIVFRDQKGVITAPFMDIFFRGFSVDCSSEEFASKALCTAFYTGEVKQAVQVNDTHFLFSFMKAANHTDAGKFTACRGVKNIHKMGKIILFQDEPEQDIWPGDACNQFIGTDSTVFPPFLHKDEGLWAFTPDLCRSLGAHFVRKTTYAGLPASFFTLEFGDIKSTPELHCFCDDPDDPETCPPKGTMSLQACTGGPIIASLPHFYNADPKLLEGVNGLNPNEHDHAIFIDFELMSGTPFRAGKRLQFSMDIEPVEAIEQVSKVPRVVLPLFWVEEGVALNKTYTNMLKYTLFLGLRFNAGLRWTLITLSLLGLMGGGYLYMKKTDSLDITIPSKDNKVSNVKPVTDITNSVEHKPPAIAQADLTNQKVEKF

>ZtauSNMP2

MQQAGHNGAQTKRFSMRQLQQQPQQLSSPPPQLRRIFVQRLDVATVAPFNTSANKPKIAKTLFPGPIPWYSTKMLHWSLIVSFLGLLTAALGAYCGWFLFPTMVDKKVEESVIIADGSEQYKRFVQLPQPLTFKVYIFNVTNAHKIQHGALPIVEEIGPYVYRQYRRKKVKHFSRDGSKISYVQDQHFEFDEEASAPYTQSDHIVVLNMHMNSVLTIAENDPGLSLLLVHLNANLKAVFNDPKSMFLDTTVREFLFDGVRFCINTNGIAKAICNQIKEGGSKTIRELSDGSLAFSFFNHKNGTGNEVYEVHTGKGDAQRVLEIQKLDDSHNLQVWLNGSEGETSMCNQINGTDASSYPPFRKRGDSMYIFSADICRSVELFYQSDIQYQGIPGFRYSIGENFINDIGPEHDNECFCVDKLANVIKRKNGCLYAGALDLTTCLDAPVILTLPHMLGASNEYTKMIRGLRPDAKKHQTFVDVQHLTGTPLQGGKRVQFNMFLKSINRISITENLTTVLMPAIWVEEGIQLNSEMVAFFKKKLINSLKTLNIIHWASICGGVGVAAICLIYYVIQRRKPEAEVAPLK

>ZtauSNMP1b

MFKKILIGSAIALVLGIFIGFVGFPKLLKKMIKGQLNLKPGSEPRQMWEKFPIAVNFSIYVFNVTNPDEVQNGGKPRLQEVGPFVFEEWKDKYDLEDFEDEDAVAYNMRNTFIFRPDLGLSGEDLIVMPHPLIQVMSIAVKRDKEALINMIAEGIGALFKPTTPFVRAPFMDIFFRGIDVDCSVDHFAATAICLNFHTGAVKGAEKVNETHFKFSLMGGANHTDAGRYKVARGVKVSHDIGRVLEFDDSEELSVWDGDECNQFRGTDTTIFAPLLKPEEGLWSFAADLCRSLGAEFEKKTSYAGIPAYYYTIDLGDPKNDPDKHCFCKDYPDDCPPKGTMDLTLCNEAPMIVSLPHFFKADPKLVADVDGLNPQEDKHGVFIIFERISGTPLSAAKRLQFSLSVMPVPEIEVMKNLRTLTMPLFWVEEAASLDKTWTDMLKKKVFLVIKINNVFKWISTIFGALGLIVSLYMLYGKNQLTTTDVTPTTEVNTIDKH

>ZtauSNMP1a

MKTMRVQRHKLLIASVSAMLFGVIFGWVGFPKILKMMIKKQVSLKPGTEIRDLWTQTPFPLHFYIYVFNITNPEEVINGEKPNLQEVGPFVFDEWKDKYDLADDPIEDSISYNMRNTFYYNEKASKGLTGEELITIPHPLLVPISVVVQRERAAMLDLVAKAINIVFAGEKAVITVKFMDLFFRGLYVDCSSPEFAAKALCTAFYTGEVKQARQVNSTHFLFSFMGNNNHTDGGRFTVCRGVKNFSKLGKVIRFGDEPTLDMWAGDECNEFIGTDSTIFAPFMTKEQGLWAFTPDVCRSFGAVYRRKSSYHGMPAMRYHMDLGDIKADPSLHCFCDDPEDIDSCPPKGTMNLEPCVGAPIMASMPHFYNADPRLLEEVNGLNPNEHDHAVFIDFELTSGTPFQAAKRLQFNFDMEPVEKIEPTKNLRKMIFPIFWVEEGVALNKTFTNMLKYTLFLGLKFNSALRWSLITMALVGLMSTCYLYYKKSDSLDITVPPKAITELTNKVEDVKPVSDKRSHPPTIAQADLSNPRDTRNTF

>BdorSNMP2

MFHWSLFVSLLGLLIAALGAYCGWFLFPNMVDKKVEESVIIADGSEQYKRFVQLPQPLTFKVYIFNVTNAHKIQQGAIPIVEEIGPYVYRQYRRKKVKHFSRDGSKISYVQDQHFEFDEEASAPYTQSDHIVVLNMHMNAFLQVFEREITDIFQGFANRLNHRLNRTPGVRVLKRLMERIRGKRKSVLTIAENDPGLSLLLVHLNANLKAVFNDPKSMFLDTTVREFLFDGVRFCINTNGIAKAICNQIKEGGSKTIRELSDGSLAFSFFNHKNGTGNEVYEVHTGKGDAQRVLEIQKLDDSHNLQVWLNGSEGETSMCNQINGTDASSYPPFRKRGDSMYIFSADICRSVQLFYQSDIQYQGIPGFRYSIGENFINDIGPEHDNECFCVDKLANVIKRKNGCLYAGALDLTTCLDAPVILTLPHMLGASNEYTKMIRGLRPDAKKHQTFVDVQHLTGTPLQGGKRVQFNMFLKSINRISITENLTTVLMPAIWVEEGIQLNSEMVAFFKKKLINSLKTLNIIHWASICGGIGVAAICLIYYVIQRRKPEAEVAPLK

>BdorSNMP1b

MFKKILIGSAIALVLGIFVGFIGFPKLLNKMIKGQLNLKPGSEPRQMWEKFPIALNFSIYVFNVTNPDEVQNGGKPRVQEVGPFVFEEWKDKYDLEDFEDEDAVAYNMRNTFIFRPDLGLGAEELIVMPHPLVQIMAIAVKRDKEALINMISEGLQALFKPTTPFVRAPFMDIFFRGIDVDCSIDHFAAKAICLNFHTGAIKGAEKVNSTHFKFSLFGGANHTDAGRYKVARGVKVSRDIGRVLEFDDSDELSVWDGDECNQFRGTDTTIFAPLMKPEEGLWSFAADLCRSLGAEFEKKTTYAGIPAYYYTIDLGDPKNDPDKHCFCKDYPDDCPPKGTMDLTLCNEAPMIVSLPHFFKADPQLVADVDGVDPVEEKHGVFIVFERISGTPLSAAKRLQFSLSVMPVPEVEVMKNLRTLTMPLFWVEEAASLDKTWTDMLKKKVFLVIKINNVFKWMSTIFGALGLAISLYMLFGRNQITTTNVTPTTEASTIDKH

>BdorSNMP1a

MKVERHKLLIASVAAMLFGVIFGWVGFPKILKMMIKKQVSLKPGTEIRDLWTQTPFPLHFYIYVFNITNPDEVMNGEKPNLQEIGPFVFDEWKDKYDLADDPLEDSISFNMRNTFYFNEKDSKGLTGEELITIPHPLLVPISVVVQRDRAAMLDLVAKAINIVFAGQKAVITVKFMDVFFRGLYVDCSSTEFAAKALCTAFYTGEVKQARQVNSTHFLFSFMANNNHTDGGRFTVCRGVKNVSKLGKVIRFGDEPTLDIWAGEECNEFIGTDSTIFAPFMTKEQGLWAFTPDVCRSFGATYKRKSSYHGMPAMRYHMDLGDIKADPSLHCFCDDPEDVDSCPPKGTMNLEPCVGAPIMASMPHFFNADPKLLEEVNGLSPNEKDHAVFIDFELTSGTPFQAAKRLQFNMDMEPVEKIEPTKNLRKMIFPLFWIEEGVALNKTFTNMLKYTLFLGLKFNSALRWSLITMALVGLMSTSYLYYKKSDSLDITVPPKAIKELANKVEDVKPLPPEDKKPVPPIAQADLSNRRDAMDRF

>BoletSNMP1

MKVERHKLLIASVAALLFGVIFGWVGFPKILKMMIKKQVSLKPGTEIRDLWTQTPFPLHFYIYVFNITNPDEVMKGEKPNLQEIGPFVFDEWKDKYDLVDDPLEDSISFTMRNTFYFNEKNSKGLTGEELITIPHPLLVPISVVVQRERAAMLDLVAKAINIVFAGQKAVITAKFMDVFFRGLYVDCSSPEFAAKALCTAFYTGEVRQARQVNSTHFLFSFLANNNHTDGGRFTVCRGVKNVSKLGKVIRFGDEPTLDIWGGEECNEFIGTDSTIFAPFMTKEQGLWAFTPDLCRSFGAVYKRKSSYHGMPAMRYHMDLGDIKADPSLHCFCDDPEDIESCPPKGTMNLEPCVGGPIIASMPHFYNADPKLLEEVNGLSPNEKDHAVFIDFELTSGTPFQAAKRLQFNMDMEPVEKIEPTKDLRKMIFPLFWIEEGVALNKTFTNMLKYTLFLGLKFNSALRWSLITMALVGLMSTCYLYYKKSDSLDITVPPKAIKERTSKVEDVKPLPPADKKPIPPIAQADLSNRRDTTNRF

>BoletSNMP2

MTTAKIAKSLFPGSIPWYSTKMFHWSLIVSLMGLLTAALGAYCGWFLFPSMVDKKVEESVIIADGSEQYKRFVQLPQPLTFKVYIFNVTNAHKIQQGAIPIVEEIGPYVYRQYRRKKVKHFSRDGSKISYVQDQRFEFDEVASAPYTQSDHIVVLNMHMNAFLQVFEREITDIFQGFANRLNHRLNRTPGVRILKRLMERIRGKRKSVLTIAENDPGLSLLLVHLNANLKAVFNDPKSMFLDTTVREFLFDGVRFCINTNGIAKAICNQIKEGGSKTIRELSDGSLAFSFFNHKNGTGNEVYEVHTGKGDAQRVLEIQKLDDSHNLQVWLNGSEGETSMCNQINGTDASSYPPFRKRGDSMYIFSADICRSVQLFYQSDIQYQGIPGFRYSIGENFINDIGPEHDNECFCVDKLANVIKRKNGCLYAGALDLTTCLDAPVILTLPHMLGASNEYTKMIRGLRPDAKKHQTFVDVQHLTGTPLQGGKRVQFNMFLKSINRISITENLTTVLMPAIWVEEGIQLNSEMVAFFKKKLINSLKTLNIIHWASICGGIGVAAICLIYYVIQRRKPEAEVAPLK

>BlatSNMP1

MKVERHKLLIASVAAMLFGVIFGWVGFPKILKMMIKKQVSLKPGTEIRDLWTQTPFPLHFYIYVFNITNPDEVMNGEKPNLQEIGPFVFDEWKDKYDLVDDPLEDSISFNMRNTFYFNEKDSKGLTGEELITIPHPLLVPISVVVQRDRAAMLDLVAKAINIVFAGQKAVITVKFMDVFFRGLYVDCSSAEFAAKALCTAFYTGEVKQARQVNSTHFLFSFMGNNNHTDGGRFSVCRGVKNVSKLGKVIRFGDEPTLDIWGGDECNEFIGTDSTIFAPFMTKEQGLWAFTPDVCRSFGATYKRKSSYHGMPAMRYHMDLGDIKADPSLHCFCDDPEDVDSCPPKGTMNLEPCVGAPIMASMPHFFNADPKLLEEVNGLSPNEKDHAVFIDFELTSGTPFQAAKRLQFNLDMEPVEKIEPTKNLRKMIFPLFWIEEGVALNKTFTNMLKYTLFLGLKFNSALRWSLITMALVGLMSTCYLYYKKSDSLDITVPPKAIKELANKVEDVKPLPPEDKKPVPPIAQADLSNRRDAMDRF

>BlatSNMP2

MFHWSLIVSLLGLLTAALGAYCGWFLFPNIVDKKVEESVIIADGSEQYKRFVQLPQPLTFKVYIFNVTNAHKIQQGAIPIVEEIGPYVYRQYRRKKVKHFSRDGSKISYVQDQHFEFDEEASAPYTQSDHIVVLNMHMNAFLQVFEREITDIFQGFANRLNHRLNRTPGVRVLKRLMERIRGKRKSVLTIAENDPGLSLLLVHLNANLKAVFNDPKSMFLATTVREFLFDGVRFCINTNGIAKAICNQIKEGGSKTIRELSDGSLAFSFFNHKNGTGNEVYEVHTGKGDAQRVLEIQKLDDSHNLQVWLNGSEGETSMCNQINGTDASSYPPFRKRGDSMYIFSADICRSVQLFYQSDIQYQGIPGFRYSIGENFINDIGPEHDNECFCVDKLANVIKRKNGCLYAGALDLTTCLDAPVILTLPHMLGASNEYTKMIRGLRPDAKKHQTFVDVQHLTGTPLQGGKRVQFNMFLKSINRISITENLTTVLMPAIWVEEGIQLNSEMVAFFKKKLINSLKTLNIIHWASICGGIGVAAICLIYYVIQRRKPEAEVAPLK

>BneoSNMP1

MKVERHKLLIASVAAMLFGVIFGWVGFPKILKMMIKKQVSLKPGTEIRDLWTQTPFPLHFYIYVFNITNPDEVMNGEKPNLQEIGPFVFDEWKDKYDLADDPLEDSISFNMRNTFYFNEKDSKGLTGEELITIPHPLLVPISVVVQRDRAAMLDLVAKAINIVFAGQKAVITVKFMDVFFRGLYVDCSSTEFAAKALCTAFYTGEVKQAKQVNSTHFLFSFMANNNHTDGGRFTVCRGVKNVSKLGKVIRFGDEPTLDVWGGEECNEFIGTDSTIFAPFMTKEQGLWAFTPDVCRSFGATYKRKSSYHGMPAMRYHMDLGDIKADPSLHCFCDDPEDVDSCPPKGTMNLEPCVGAPIMASMPHFFNADPKLLEEVNGLSPNEKDHAVFIDFELTSGTPFQAAKRLQFNMDMEPVEKIEPTKNLRKMIFPLFWIEEGVALNKTFTNMLKYTLFLGLKFNSALRWSLITMALVGLMSTCYLYYKKSDSLDITVPPKAIKELANKVEDVKPLPPEDKKPVPPIAQADLSNRRDAMDRF

>BneoSNMP2

MFHWSLIVSLLGLLTAALGAYCGWFLFPNMVDKKVEESVIIADGSEQYKRFVQLPQPLTFKVYIFNVTNAHKIQQGAIPIVEEIGPYVYRQYRRKKVKHFSRDGSKISYVQDQHFEFDEEASAPYTQSDHIVVLNMHMNAFLQVFEREITDIFQGFANRLNHRLNRTPGVRVLKRLMERIRGKPPYKWMYNIFNEIFKQVKSVLTIAENDPGLSLLLVHLNANLKAVFNDPKSMFLDTTVREFLFDGVRFCINTNGIAKAICNQIKEGGSKTIRELSDGSLAFSFFNHKNGTGNEVYEVHTGKGDAQRVLEIQKLDDSHNLQVWLNGSEGETSMCNQINGTDASSYPPFRKRGDSMYIFSADICRSVQLFYQSDIQYQGIPGFRYSIGENFINDIGPEHDNECFCVDKLANVIKRKNGCLYAGALDLTTCLDAPVILTLPHMLGASNEYTKMIRGLRPDAKKHQTFVDVQHLTGTPLQGGKRVQFNMFLKSINRISITENLTTVLMPAIWVEEGIQLNSEMVAFFKKKLINSLKTLNIIHWASICGGIGVAAICLIYYVIQRRKPEAEVAPLK

>AludSNMP2

MQQASHVGAFAMRHSTHQQQKLQQLRSTPHSRRIFVHELHGGATVARFDSSAYQSNIAKSLFPVSVPWYSSKMIHWSLIVSFIGLLTAALGAYCGWFLFPSMVDKKVEESVIIADGSEQYKRFVQLPQPLTFKVYIFNVTNAQKIQQGAIPIVEEVGPYVYRQYRRKKVKHFSRDGSKISYVQDTRFEFDEEASAPYTQSDRIVVLNMHMNAFLQVFEREITDIFQGFANRLNHRLNHTPGVRILKRLMERIRGKRKSVLQISENDPGLSLLLVHLNANLKAVFNDPKSMFLDTTVREFLFDGVRFCINTNGIAKAICNQIKEGGSKTIRELSDGSLAFSFFNHKNGTGKEVYEVHTGKGDAQRVLEIQKLDDSHNLQVWLNGSEGETSMCNQINGTDASSYPPFRKRGDSMYIFSADICRSVELFYQSDIQYQGIPGFRYSIGENFINDIGPEHDNECFCVDKLANVIKRKNGCLYAGALDLTTCLDAPVILTLPHMLGASNEYTKMIRGLRPDAKKHQTFVDVQHLTGTPLQGGKRVQFNMFLKSINRISITENLSTVLMPAIWVEEGIQLNSEMVAFFKKKLINSLKTLNIIHWASLCGGIGVAAICLIYYVVQRRRPETEVAPLK

>RzepSNMP2

MQQTGHVYSHIIRGSTRPPYQPQYQRPPPPQPRRVFVQDFQVDATAPAFDSSAYKSNIAKSLFPGSVPRCSSKMLHWSLIVSFIGLLTAALGAYCGWFLFPSMVDKKVEENVIIADGSEQYKRFVQLPQPLIFKVYIFNVTNAQKVQQGAIPIVQEVGPYVYRQYRRKKVKHFSRDGSKISYVQDQHFEFDEEASAPYTQSDHIVVLNMHMNAFLQVFEREITDIFQGFANRLNHRLNHTPGVRILKRLMERIRGKRKSVLQISENDPGLSLLLVHLNANLKAVFNDPKSMFLDTTVREFLFDGVRFCINPNGIAKAICNQIKEGGSKTIREMSDGSLAFSFFNHKNGTGKEVYEVHTGKGDARRVLEIQKLDDSHNLQVWLNGSEGETSMCNQINGTDASSYPPFRKRGDSMYIFSADICRSVELFYQSDIQYQGIPGFRYSIGENFINDIGPEHDNECFCVDKLANVIKRKNGCLYAGALDLTTCLDAPVILTLPHMLGASNEYTRMIRGLRPDAKKHQTFVDVQHLTGTPLQGGKRVQFNXTIKSEELRPINTLPNLEKILETVVKNQLLNYIESNKILIKEQSGFRANHSCETALNLILSQQWKEDLNSKKTILAMFFGP

>RzepSNMP1

MQVQRQKLLIASVAAMAFGLLFGWVGFPKILKAMIKKQVSLKPGTEIRDLWTQTPFPLHFYIYVFNITNPDEVINGGKPNLQEIGPFVFDEWKDKYDLVDDAVEDSISFNMRNTFYYNAKASNGLTGEELITIAHPLLVPISVVVQRDRAALLDLAAKAISIVFAGEKAVITAKFMDLFFRGLYVDCSSEEFASKALCTAFYTGEVKQAKQVNATHFLFSFMANNNHTDAGRFTVCRGVKNVSKLGKVIRFGDEPTLDIWPGDECNEFIGTDSTIFAPFMTKEQGLWAFTPDLCRSVGALYKRKSSYHGMPAMRYHMDLGDIKSDPSLHCFCDDPEDIETCPPKGTMNLEPCVGAPIIASMPHFYNADPSLLEQVNGLSPNEKDHAVFIDFELTSGTPFQAAKRLQLNLDMEPVEKIEPVRNLRKMILPLFWVEEGVALNKTFTNMLKYTLFLGLKVNSALRWTLITMALVGFMSTSYLYYKKSDSLDITVPPKVIPEPSNKVEDVKPAADKLNAPSITQADLSNRRLPVDRF

>RpomSNMP1

MQVQRQKLLIASVAAMAFGLLFGWVGFPKILKAMIKKQVSLKPGTEIRDLWTQTPFPLHFYIYVFNITNPDEVINGGKPNLQEIGPFVFDEWKDKYDLVDDAVEDSISFNMRNTFYYNAKASNGLTGEELITIAHPLLVPISVVVQRERAAMLDLVAKAISIVFAGEKAIITAKFMDLFFRGLYVDCSSEEFASKALCTAFYTGEVKQAKQVNATHFLFSFMANNNHTDAGRFTVCRGVKNVSKLGKVIRFGDEPTLDIWPGDECNEFIGTDSTIFAPFMTKEQGLWAFTPDLCRSFGALYKRKSSYHGMPAMRYHMDLGDIKSDPSLHCFCDDPEDIETCPPKGTMNLEPCVGAPIIASMPHFYNADPSLLEQVNGLSPNEKDHAVFIDFELTSGTPFQAAKRLQFNLDMEPVEKIEPVRNLRKMILPLFWVEEGVALNKTFTNMLKYTLFLGLKFNSALRWTLITMALVGFMSTSYLYYKKSDSLDITVPPKVIPEPSNKVEDVKPAADKVNAPSITQADLSNRRLPVDRF

>RpomSNMP2

MTTANIAKSLFPGSVPRCSSKMLHWSLIVSFIGLLTAALGAYCGWFLFPSMVDKKVEENVIIADGSEQYKRFVQLPQPLIFKVYIFNVTNAQKVQQGAIPIVQEVGPYVYRQYRRKKVKHFSRDGSKISYVQDQHFEFDEEASAPYTQSDHIVVLNMHMNAFLQVFEREITDIFQGFANRLNHRLNHTPGVRILKRLMERIRGKRKSVLQISENDPGLSLLLVHLNANLKAVFNDPKSMFLDTTVREFLFDGVRFCINPNGIAKAICNQIKEGGSKTIREMSDGSLAFSFFNHKNGTGKEVYEVHTGKGDARRVLEIQKLDDSHNLQVWLNGSEGETSMCNQINGTDASSYPPFRKRGDSMYIFSADICRSVELFYQSDIQYQGIPGFRYSIGENFINDIGPEHDNECFCVDKLANVIKRKNGCLYAGALDLTTCLDAPVILTLPHMLGASNEYTRMIRGLRPDAKKHQTFVDVQHLTGTPLQGGKRVQFNMFLKTINRISITENLTTVLMPAIWVEEGIQLNSEMVAFFKKKLINSLKTLNIIHWASLCGGIGVAAICLIYYVIQRRKPEAEVAPLK

>DmelSNMP2

MIHWSLIVSALGVCVAVLGGYCGWILFPNMVHKKVEQSVVIQDGSEQFKRFVNLPQPLNFKVYIFNVTNSDRIQQGAIPIVEEIGPYVYKQFRQKKVKHFSRDGSKISYVQNVHFDFDAAASAPYTQDDRIVALNMHMNAFLQVFEREITDIFQGFANRLNSRLNQTPGVRVLKRLMERIRGKRKSVLQISENDPGLALLLVHLNANLKAVFNDPRSMSVSTSVREYLFDGVRFCINPQGIAKAICNQIKESGSKTIREKSDGSLAFSFFGHKNGSGHEVYEVHTGKGDPMRVLEIQKLDDSHNLQVWLNASSEGETSVCNQINGTDASAYPPFRQRGDSMYIFSADICRSVQLFYQTDIQYQGIPGYRYSIGENFINDIGPEHDNECFCVDKLANVIKRKNGCLYAGALDLTTCLDAPVILTLPHMLGASNEYRKMIRGLKPDAKKHQTFVDVQSLTGTPLQGGKRVQFNMFLKSINRIGITENLPTVLMPAIWVEEGIQLNGEMVAFFKKKLISTLKTLNIVHWATLCGGIGVAVACLIYYIYQRGRVVEPPVK

>DmelSNMP1

MQVPRVKLLMGSGAMFVFAIIYGWVIFPKILKFMISKQVTLKPGSDVRELWSNTPFPLHFYIYVFNVTNPDEVSEGAKPRLQEVGPFVFDEWKDKYDLEDDVVEDTVSFTMRNTFIFNPKESLPLTGEEEIILPHPIMLPGGISVQREKAAMMELVSKGLSIVFPDAKAFLKAKFMDLFFRGINVDCSSEEFSAKALCTVFYTGEIKQAKQVNQTHFLFSFMGQANHSDSGRFTVCRGVKNNKKLGKVVKFADEPEQDIWPDGECNTFVGTDSTVFAPGLKKEDGLWAFTPDLCRSLGAYYQHKSSYHGMPSMRYTLDLGDIRADEKLHCFCEDPEDLDTCPPKGTMNLAACVGGPLMASMPHFYLGDPKLVADVDGLNPNEKDHAVYIDFELMSGTPFQAAKRLQFNLDMEPVEGIEPMKNLPKLILPMFWVEEGVQLNKTYTNLVKYTLFLGLKINSVLRWSLITFSLVGLMFSAYLFYHKSDSLDINSILKDNNKVDDVASTKEPLPSANPKQSSTVHPVQLPNTLIPGTNPATNPATHHKMEHRERY

>LserSNMP1

MQINRNRLLIVSGGVLVFAIMNGWLIFPKILKLVLKKQVNLKPGSDIRELWENTPFPLHFYFYVFNITNPDEFLNGAKPNLQEIGPFVFDEWKSKYDLEDNDEEDTVTYHMRNTFIFNAKASAPLTGEEVITMVHPLIAPIAVVVLREKAAMMDLIVKAIEIVFRGHKGVLTAPFMDIFFRGFYVDCSSEEFASKALCTAFYTGDVKQAQQVNDTHFLFSFMRAANNSDAGKFTACRGVKNVHKMGKIILFQDEPELDVWAGDACNQLIGTDSTVFPPFLHKDEGLWAFTPDLCRSLGAHYVRKSSYSGLPASFFSLDFGDLKNTPELHCFCDDPDDPESCPPKGTMSLQACNGAPILASLPHFYNADPKLQEGVNGLNPNEHDHAIYIDFELMSGTPLRAGKRLQFSLDLEPVEAIEQVSKVPRVVLPMFWVEEGAALNKTYVNMLKYTLFLGLRFNVGLRWTLITLSLLGLMGGGYLYMKKTDSLDITLPNKDNKVSNVKPVSATTAPVEIKPPPHPLAQADLTNQKVEKF

>LserSNMP2

MFKVFHWSLMGAGLGLLVSMLGFYCGWFLFPTMIHKKVEESVIIADGSEQYKRFIQTPQPLTFKVYIFNVTNPHKVQQGALPIVKEIGPYVYKQYRAKKVKHFSHDGSKITFVQDQLYTFDAEASAPFKESDHIVALNMHMNAFLQVFEREITDILQGFANRINSRLNRTPGVRVLKRLMDRIRGKRKSVLQIGENDPSLALLLVHLNANLKAIFNDPKTMFVHTTVKEYLFDGVRFCINPQGLAKAICNQIKDGGSKTIRELKDGSLAFSFFNHKNGTGHDVYEVHTGKGDAMRVLEIQKLDDNHHLQVWLNSSDNNGTSKCNQINGTDASMYPPFRQKGDSMYIFSADICRSVQLFYQKEIMYKGIPGYRYSIGENFVNDIGPEHDNECFCVDKLTNIIKRKNGCLYAGALDLTTCLDAPVILTLPHMLGASNEYTKTIRGLNPDAQKHQTFVDVQHLTGVPLQGGKRVQFNMFLKSINRITITENLTTVLMPAIWVEEGIELNGEMVTFFKKRLINTLKTLNVVQWAALFGGIGVAAICLIYYVVQSRKPAAVVEAPLK

>LcupSNMP2

MHTKGAKKEKKQRRSNEKQWLNKRRRVLRMGIISKQKFNFKHIKSDSEIKSVIIADGSEQYKRFIQTPQPLTFKVYIFNVTNPHKVQQGALPIVKEIGPYVYKQYRAKKVKHFSHDGSKITFVQDQLYTFDAEASAPFRESDHIVALNMHMNAFLQVFEREITDILQGFANRINSRLNRTPGVRVLKRLMDRIRGKRKSVLQIGENDPSLALLLVHLNANLKAIFNDPKTMFVHTTVKEYLFDGVRFCINPQGLAKAICNQIKDGGSKTIRELKDGSLAFSFFNHKNGTGHDVYEVHTGKGDAMRVLEIQKLDDNHHLQVWLNSSDNNGTSKCNQINGTDASMYPPFRQKGDSMYIFSADICRSVQLFYQKEIMYKGIPGYRYSIGENFVNDIGPEHDNECFCVDKLTNIIKRKNGCLYAGALDLTTCLDAPVILTLPHMLGASNEYTKTIRGLNPDAKKHQTFVDVQHLTGVPLQGGKRVQFNMFLKSINRITITENLTTVLMPAIWVEEGIELNGEMVTFFKKRLINTLKTLNVVQWAALFGGIGVAAICLIYFVVQRRKAVAVVEAPLK

>LcupSNMP1

MQINRNRLLLVSGGVFVFAIMNGWLIFPKILKFVLKKQVNLKPGSDIRELWENTPFPLHFYFYVFNITNPDEFLNGAKPNLQEIGPFVFDEWKSKFDLEDDDEEDTVTYHMRNTFIFNAKASAPLTGEEVITMVHPLIAPIAVVVLREKAAMMELIVKAIEIVFRGHKGVLTAPFMDIFFRGFYVDCSSEEFASKALCTAFYTGDVKQAQQVNDTHFLFSFMQAANNSDAGKFTACRGVKNVHKMGKIILFQDEPELDVWAGDACNQLIGTDSTVFPPFLHKDEGLWAFTPDLCRSLGAHYVRKSSYSGLPASFFSLDFGDLKNTPELHCFCDDPDDPESCPPKGTMSLQACNGAPILASLPHFYNADPKLQEGVNGLNPNEHDHAIYIDFELMSGTPLRAAKRLQFSLDLEPVEAIEQVSKVPRVVLPMFWVEEGAALNKTYVNMLKYTLFLGLRFNVGLRWTLITLSLLGLMGGGYLYMKKTDSLDITIPNKDNKVSNVKPVSATTAPVEMKPAPHSLANADLTNQKVEKF

>BcucOR1

MDSSIDTVNTFKRLFFFWRILGFTTNHNKYLIRLYDIFVTIFATFAFPLHLALGVIFADDKEVVFTNLAIGISTFACTAKHLMLRPQLSKVIAVNRILQKLDERVQSDEDTHYYIKQMREKCIFMMHFFTVVYFSVAVMAVLSALWSGKVLYPAYVVVDWHGSTWKYLAVMLFQIYGLNMQIVQNLTNDAYGPMILCLLSGHVHLLSRRILRIGHEHETEVERNYAELVHCIDDYKVLMRVVERVISSSYMVQFTAVGINVVVGLIYLLFFADNLFAYCYYVFHILAIMIEIFPCCYYGSMVQAEFHALSYAIFRSNWLSQSRTFRRAAVTFTELSLKDVTVTAGGMMKIHLDSFFKTCKMGYSIFTGKHTKHLRLVYDILMNTVVTFGFTAHLVLGIILSTNQDQFFTNLVIGIASVSCVFKHLLYRFRMPEMQRINEILGQLDDRVRTKEDYDYYKRLMERPCNFMVNFFTRCYFAVSITALIMALLTGELLYPAFIPLQWRTSVFKYAVGLLFQFVAVSLQIVQNIANDAYGPVLLCMLSGHVHLLSNRVSRIGHDKPESVKDNYKELSLCIEDHKLLMSTTKAVEHMVSASYLVQFGGVGINLCIGLVYLLFFADNYFAYVYYTIHITAIMIELFPCCYYGSMLECEFHDLSYAIFSCNWPMQPRPFRRNIVNFTELTLREVALYAGGMVRINLDSFFATCKTGYSFFTVIQSMK

>BcucOR2

MLKTWCNSSINLLKSVATVQQQVRTTFVLKRKYDPLLHKTNAKPKKLRAKHFIYELVEDTNVKRRPNLEVVLKTYVEGVGDKGDVVSVRPNFAYNKLLLPGLAVYKTDENVALYAKTEDEKKTVSHSSAFAQRTVNVIERFTLAVVMNKDQPWVVEPWHIKASLRKAGIHCPEDCITMPEARIEGPDMNKEGKEFYCTITVNNLEKARLRCRIHHWSTDPSERLPYVPEYWKMTSEPLFGSENSKEALAEKVEEKYVLFGGIVGGEPRAPYAMCGGQVMRRLPPTFIPTTPTSQPLMTSPRPRPNLKPAPVFRLSNTLLQRMFELVTGRGTGNATSKDAFVYFFKGCTIMGISPPKNAGPLYYMWSLIVNLVCIITSPITGIVGFANKYLQDIITTAQFLSGLQAGLNLIGLPVKCATVTFALKRLRGMESTLAIMDARYTRPEDVALIRKAAVMGNRLVLIFGTSYFIYMLFTVLPPLINGNPPLSVWIPFVYENQSTMHFCVQIFYDLFIMFFVLCHQTLYDSYGPVYIYVISTHFQLLVRRVGNLGTDATKSKDDNMKELVDCVVTHQQILELLATIEPVISTTMFTQFLIISSILCVTMVNMFFFADRSTQFASTLYFLCVLLQTSPCCYFATELKADSEKLPLAIFHCNWVEQDQRFRKVIIYFMHHAQISVELMAMKLFPINVGTNISLAKFSFTLFTFLKEMGIGQETTN

>BcucOR3

MYKYLRIQHFSFQVIGINLWAQRDQRIMSAPCRYCCWTLATAIITLLMGCYIYTSEQDKAIQVLTVFLQGVLSVIKSGMFVARGKRFIKLIRSLDTLADEENVKKGREWKQENDWQQWIVHIYYICCTCTGILYTTVSTIILLYSLCFKEHTELILPFEAAFPFDTGDLFFFTASYIWCLSMIIYAIHAIVAMDSLFSFVRIGLSIELGGSKGIFSHPERYATVMIGVIVWAVALFTYTMEYLADVDKIVAAMTINVQLCLTTSKNFIFLARRERFLRLNEALERLALTGNNIERELWNTSNRRVLPITMAYSISCQMTVSICVLLPILKLLYYYIWHNEVVLTLPLPGIFPYDYTVPFYFILTTILSVLLVYFCVYTICAVDGLFGWFVYNISAHLQIMRLKLEQLLQLHVDDPNFQRDLVALVNYHRQIIDLSLELDALYAPIIFLEVTSSSLPICFLAYQLSYLSDPASVPFMCLLMSSIVIQLMIYCFGGEKVQNECDQLCENIYLLIPWHKLPPKHCRLLLNPFIRSQRVLVLTGYFFTANRSLLVWIFRTAGSFTALLFALKEKEV

>BcucOR4/Orco

MQPSKYVGLVADLMPNIRLMKYSGLFMHNFTGGSGLFKKIYSSIHLVLVVVQFLLILVNLALNAEEVNELSGNTITVLFFTHSITKFVYLAVSQKNFYRTLNIWNQVNSHPLFAESDARYHAIALAKMRKLFTLVMLTTVATAVAWTTITFFGESVKFAVDKETNSTITVEIPRLPIKSFYPWNAGAGMFYMISFAFQCYYLLFSMVHSNLCDVLFCSWLIFACEQLQHLKGIMKPLMELSASLDTYRPNSAALFRSLSANSKSELINNEEKEPTDLDISGVYSSKADWGAQFRAPSTLQTFNGMNGTNPNGLTRKQEMMVRSAIKYWVERHKHVVRLVAAIGDTYGGALLLHMLTSTIMLTLLAYQATKVTGVNVYAFTTIGYLGYALAQVFHFCIFGNRLIEESSSVMEAAYSCHWYDGSEEAKTFVQIVCQQCQKAMSISGAKFFTVSLDLFASVLGAVVTYFMVLVQLK

> BcucOR5

MSPPSKRNIGLTGSCTNPNGCISNEKRSDLFLYVRWLLFFSAIRPIPFDKHLPRRMHGHSVLVNVIWEIFLYLVVLHILVLFIITIYLNYDNGDLEFLISCGIQVLIYLWAILIKVTFRRIYPELVNGIVDFVNEEYVQHSALGFTYVTMKECVDRVNGGIRIFVPCCFSAVIYRFILPIIYNDRSLPLPCWYPVNYKAPFIFQILYFFQILAQLQMSAAFTVSSVYFISLCFLLSGQFDVLNCSLKNIVATTYIYMGASKHELIELRDNERIPGEEINEFFVAKELPFDLDCLPHILNPADTARTRSFREAFNYALGSCVKQHNFILNALLKLERLYNLLWLFKTLDVTLSICMGTFDVVKSSDEKSFLQLLSLGQYLFLGLWEIFMICYAGEIIYVNSQRCDEALLRSPWHLHLREVRADFLLFLMNAQRAFKLTGGKFYPLALEKFRGIVSTSFSFYTLLQNLDERN

>BcucOR6

MLFNPKPLKDPISFRFPLQCIWLKLNGSWPLRPQRSGEFERYFRWLYSVWAWYVVAMVGITIGFQSAFLVKTFGDIMVTTENGCTTFMGVLNFVRLLHLRLHQREFQQLLARFVKDIWITSSSQPIVERACARNMRVFQVISALQSCLITMYCLLPLVELYMLTVNTAPDVLESVEKPFPYKMLFPYDANYGWRYALTYLFTAWAGVCVVTTLFAEDSLFGFFTTYTCGQFRILHIQIDNIIPDSYAATRAGRGTEADFQRESVRRLDRIAGKHCVLFNFVRSMEEFFSPILLVNFLISTILICMVGFQLVTGKNMFIGDYVKFLVYILSSLSQLFVLCWNGDNIIQNSLEMANHLYACNWECGVEVAATNADNKRADEQVKMAAPTVYYTTNNAFRKKLQFMIMRSQRQTCITALKFSVLSLSSFSGLISSSMSYFALLQSFNEDEEN

>BcucOR7

MISLSTQATINNTISTHNSYLSNDSNTHLKHTASKLRTILAPYRVLKEMLRSGEAVHPPHTCLFYFRAYIRLLGLWPAERAVENPLYYAFNVLIMLLFGFFTVTIICDLYEASSDFVLFGEDLVVVLGLCLIFFKMILFRLGNADTDIIINEFDALHVKHFNESHDSPRNRRTRQWQRSFFFGEMCFFSGFYILSLFLFAAMSLQPLLSQQILPFRCKFPFGLDDPDEHPMGFVCVYFFQCFCTLYMLVAIVVMDSLGGNSFNQTTLNLRILCENMRNLGNGSTSELVVWRKLKETVEFHQQIIKLMNRINQTFYWNYVSQMGASTFMICLTAFEALLAQDKPMVALKFQTYMFSAFMQLLYWCWMGNRTYYDSMEVATAAYEVRTWYRHSPLLQRQLIFIIKRAQKPLEFRAKPLFGFTFASFTSILSTSYSYFTLLRTMSD

>BcucOR8

MLCKMSKLQPQLTAVMAKDVKTAQRNTNVQRKPLSVLEAKNLVKTLSEETVEEDVVQPVSTQDTTKYLFKAAFLMGIVMPSRYRALYVLHSFWVNFLTTFYFPIGFTLIFFTLSDEINISNLLTSLQVIFDVYGGSAKFIVMMCMLEKLRATQAITQQLDRRCRAADEIAELQKMVRFGQKVVIFYLTIFLCYSGSTFMASFFSGYPPYSLYFPFLKWRRSHSEFIIASFLEFVIMDLACLQQTVNDGYPVIYINMMLCHMKILQLRVQKLGNNTALTLEEHLSELKLCIKDHQLLIELYDIISPIVSVTLFIQFTLSAVCIGTTLINIVIFANEFQTQVACCFFILAVLIEIFPACYFSQCLIDESDNLSDVIFHSKWVEQSKEYRKLMIFFLQRSQRPMFLTAGKLFPVTLSSFVSIAKFSFSLYTFIEKMNLKERLGIE

>BcucOR9

MSRFLDGFFPKVEIENMTNSSYLPIMRYLPASYHKPLLPNGRHPPIDWQIYGFICSNCWPLAAHITKARYIVDIMVTIAQFMSESMVLIGEGIVMHDNLDNISFVCTVLAPNLILIEMMLRAYNIIYRRSSFRKHIEEFYKKIYVQRTWNPDLFEQIRRQQLPTKYSTCTYIITLVTYVYVPISGLIKNERLVPFPIRFSFDYTVPWPRYLVFLAMSIWTGFAVVGPLVAEPNLLAMQILHLNGRYSLLLQDLRKISKESIVEHERLKGKDTLLVTQRFRYRLFEIIRRNVELNEFAKSLQEQYSFRVFVMMAMSATLLCVLGFLTATLGLTAQNIRFVSWIIGKVVELLIFGRLGTTLSTTTDELSTSYYCCDWEDVILHSTDAEENKKLMKLIALAVHLNSNPFRLTGLNFSVVNYETVVSILRGAGSYFTVIYAYR

>BcucOR10

MSFRAYDMKTFLVYPEFALNLACFESFFWSENQYLHKSTTWYRLKRLIYIIITIILLVHICAVTASAFVPRVTPEAEAYSPSDSKLFEVLAIVSYFCCSFYKMWNIFWRRDDICRVMEELKALFPSIAKQKRLAELNESKEGKIGSGGIYRLEYYEEKSRTIMQFITRYFMFAYVAYNSIPVMQLCFAVITQQEHITYRAQANAWYPWHNHNDHSSFMGFMLSYLTQAIVEYTSIAFVMSGEFIFCFFTTQMLMHYNYLCSALSALDASAPDAVRQLKALISYHTHLLRLSKLINSIFNLTFALDLIITTFAISLMGLAIVLVNFADALMFSAGFSFFLLLGYLFCNNGDEILRETMQINSAIFYSNWYEGSPEYRRLIIFFIMRTKTPCQYQAYGYTPLSMETYMRILKLSYQMFTSVRAIE

>BcucOR11

MSKIIRFEAFLRIPNFFYRSVGVDLWNTDGGPLQDAVFYFGLLNVNVWLLSELVFAVLMVSKNFIQATMTLSYAGFVLVGSIKMYFMWRKKAEMKRFLQLMNAIFPRTEQQQKIMHLRRHLRQSTIVMSCFAMMFMVLIWTYNLYPYMQRQIYDRLLDVRSINKTLPYESYIPWNWHEHWTFYLYYTLQSIAGYHAASAQIASDLVLCAMATQMIMHYEYVAQKITEYQPQVGADSGSKAELSNGKELNSQSFTTLENEAYCRDMKFLCDIIAYHANVLSLSDIMNEVLGVPLLVNFMTSSFVLCFVGFQMTMDAEPDYMVKLFLFLFSSLVQICLICQYGQLLIDSSSNIAHAVYNHDWVHSHVHYQRMLVLVAVRAQKPAMLQATSFVRISRGTITDIMQISYKFFTLIRTMYSN

>BcucOR12

MDYFVPLQFDNRPIKLPIQITGYKFNCLWPLKEDASVLWRLFNNVCLSVSVLCYIGTILGEFTFIAENISDIPAVAECLCTSFMGVQYIIRIFVLLSRQRPLRQLLRNFYRDIYFTDADDAALCKEINSIIRFINIFTQFYYVPMVLILGLYVYEVASVGMASPDKPFIYRMSFRWYDAQAPLQFIITAIYSGWLTISCVTIWTAEDYTLCMVLCHASFRYKKLRLDLEQLLETARADLRSGETRCTNLNLHIAFRRRLCDIFQRQQRLNGFVAEAKAHFTHQIFYVMSFGVLLLCVVSFQFQSSPISVEWSKYISWIISQTSQFLLIGYFGQMLMDETTELRNSFYCCRWEDLLALGDLRSNKLLLGDVQFAIMNSQEPIVFDGMKFFPLTYSTVSSALRSAVSYFMFLNTMSEN

>BcucOR13

MILEKQEEIFEHNYNRIKVLFRAAYSIGVNLMAPSKIKDALKIVHVGLIMLAFLSVYTQYCYLMRNINSIPLIAESIYTALQVLSGTSKLIYFFFTHRTFYRLLDQTLTHDIIYQMEIFQHDFPINQMLKREIDVIMNRVWRTTRFLVLLFFYACFTVFANYLVAAILINLYHQLKETPDYQFILPLPALYTFWESKGMTFPYYHMQMFITTAAMHVSAMTGASFDGTFVVLCQHAAGLMEVHNLLVQHATSPDIPPERRVEYLRYVIKTYQRVNNFMLEIQTIYRHISLAQFLLSLISFGFMLFEANYGIRPNVNNLIRMIMYIMATGTQITIYCYHGQALTNVNEEIPVVYYNCNWYDENKTFKHLIRMMIMRTNKDFYLEVSWFTLMNFATLISLFRASGSYYLILKNFQED

>BcucOR14

MVFEDVEEIYRRNYNSIKVLFQVSFGLGVNLTAPSKIKDALKLFNVILVVASLLSMYAHWCYLIRHFENIPLLAETVCTALQTLISAFKMIYFLFRQHNFYRLLDQALKHVIIREIEIFKHDFPINQQLKREIDEIMNGVWQNARRQILFYFCCCVGIVCNYFFGAFLVNLYHQLKKTPDYKYVLPFPELYPFWEDKGMTFPYYPIQMFISSSAVYIAGMCAVSFDGVFIVLCQHAVGLVRVHNLLVLRSTSPLIPVERRVEYLRYTIFTYQRIYTYVQQIQTSFKHISLSQFVLSLIVFGFVLFEMSFGLESSIIIFIRMIMYISAGGTQIIIYCYNGQALTSVSEEIPMAFYNCNWYEECEKFKQLLRMMIMRTHRYFYLEVSWFTLMNLATLIALFRMSGSYFLLLRNLQES

>BcucOR15

MYSSTEIKELRTRNYWQKRELQRVACTIGLNLNTKTKLKGWWRIITVFFIATSCVALYPHLLMIKHAQGDIPLIAETSTTALQTTTALIKMAYMMLTQHKFRKLLQKVETHELLQDIEIFKTDMPIKASLKKEIIAIMDNNWKEMRGQLLFNVGTCVGVLCNYFFYALFKNLYHHLQGTPNYVHILPFTGYPMFLDKGMDSPYYVLDMFVGACSLIAAGMGAVSFQGSFLALCKHSCGLVQVLCLLLLRSTSPLVPKQQRVKYLHYCIVQHQRTLEYINEVNQLFKHILLSQFVHGLVIYGFVLFEMNYGLESDKVTFIRMIMYLAAALTGDCMHYVNGQFLVNELENIPLACYSCDWCHETDEFKMILKMIIMRSNKTFCLEISWFAVMSLATLMGVFKASGSYFVLLRDMNES

>BcucOR16

MATKKCSSNSSTSISRISAARFIIFTLKAIGLWQWTADSSQLANSRLSYLFKLQRVHGLILHIPISFTYITLMFTAVLLS

QELEEISSVLHVLLTEFALVVKILHIWHLRNATWRYMDELANEPMYAFRRQCEWTKWQHAQRSFAIIAYTYILGSLAVVVFGCIGVMLTPADVYILPFNFYIPFEWRHPRKYWYAWTYSSLCMLLTCISNVSLDMIFCYFMFHLSLLYKLIGWRLTALRRPKSTRGRVRDGGGEDESEVIDQMAEIFQMHMKLKRLTKKCEALVSVPVLAQIVLSAFILCFSGYRLQQMDNMENVGILISTIQFASVMILQIFLPCHYGDAVNEYSNALTNDIFNSDWTTFDVPSRKFMILYMELLKRPANLKAANFFKIGLPVFAKTMNNAYSIFALLLNMNK

>BcucOR17

MALRQEKYGTAKLDDLCDILHPVQRYLRLNYLDFRRVNGRFAIPNSKLLNICLILAVFDCIGNCIKCIKAMNAGEITKAQEIFAVFGMGFVMTMRGLMLALNRVQLSNFYNKIDCIFPRSAHLQQHMAVEKVHSYIKRRFYIMHTLMTVTVAAFLTTPGVKFMVFHDFDSDDSVADEYHVNPSWLPFGLKDKVSTYPYVYIYESVLAAAAVNMIITWDEVFVVLISQLCMYYEYLGRLLEEMNVQDALDPTKLDAFYEQLHEYIYMHQYLNKLAVEFNDLFNFSILFSDAGIATSICFNIVLITDATDYLQMVTYTSPLFVEVWLIYDAAKWGTMLETVTGRINEILYEQKWYESSVRFGKYTMMWMQSTNEPFRLTAFNMFYVNMKHFQDMMMLAYQMLTFLKSKS

>BcucOR18

MLPPFKSREHAPTVQDFVYVPLFQIRFMGVKLFKWTPEERTSKLQITLMGTFCVFATFNFVSMMLFVIYDELPTSLDITEFILFWGFALNAMMKGGTMVFFRHEIESVLKGLIARHPKTEAERVAFQLVPYYKTINASNKYLSIWHLSITSIFALHPMVASILGYIWREDKNDAYVYTLPFMMGYYYDTNHPFPYAISYFIQCCGAFYMSLLFLSGDLLLISMVQLVNMHLEYLIYRIESFQPTGMDADMKVLGPLLEYHNEILDYAERIDGTFSLATLLNYGGSCLVLCLIGLQIVLGSEALSVIKFIGFLVSTIVQVFFVSYFGNNLKELSTGISDAFYNHPWYDGNYKYIRMLVLPIARAQRYAHLTAFKFFEISMDSFKSLCTTSYQFFTLLRTSIEEEGFQ

>BcucOR19

MKPAFKNSEFSPTVPDFVDIPLYLLTFIGAKLFKWTPNDPKSKSGIILLVIFSGLTVYNFTSMMRFILFENLETLLDITECILFWGFALNGLMKGGTLLCFRHEIESILKGLIVRHPKTKEERLEFQLVPYYKTINASNKYLSIWHLSITSIFILHPVLTAIYGYVNREDENEDFDYVLPFMMNYFYDVNHPLPYAISYFMQCCSAYHMSLLFLSGDLLLISMVQLVNMHLYLISRIESFQPTGTDDDIKVLGPLVEYHIEILDYTERIDNTFSLATLLNYGGTCLVLCLIGLQIVQGSDTVSVIKFIGFLVSTIVQVYFVSHFGNNLKDLSTGISDAYYNHPWYTGNYKYMRMLVLPIARSQRYAHLTAFKFFVISMDSFKSLCTLSYQFFTLLRTKIENNGA

>BcucOR20

MTTDECSSSMSRISGARYLIVTLKAIGLWQWTGDSSHLGNTGIPFLLKLQRAHGLILHVPISFTFITLMFTAVLLSHDLEEISSVFHILLTEFSLVVKILHIWRQGSAAWHYMDELAHEPMYALRRQCEWTKWQRAQRSFAIIAYSYILGSLAVVVFGCIGVMLTPADVYVLPFNYYVPFEWRHPRKYWYAWAYSSLGILLTCISNITLDMIYCYFMFHLSLLYKLIGWRLTALRRPKSMGYVDMGEPDVTKQMIEIFQMHMKVKRLTTQCETLVSVPVLAQIILSAFILCFSGYRLQHMKNMENVGILISTIQFASVMILQIFLPCYYGNAVTEYSNALTNDIFNSDWTTFDVPARKFMILYMELLKRPATLKAANFFNIGLPVFAKTMNNAYSIFALLLNMNK

>BcucOR21

MKPAFKNSEFSPTVPDFVDIPLYLLTFIGAKLFKWTPNDPKSKSGIILLVIFSGLTVYNFTSMMRFILFENLETLLDITECILFWGFALNGLMKGGTLLCFRHEIESILKGLIVRHPKTKEERLEFQLVPYYKTINASNKYLSIWHLSITSIFILHPVLTAIYGYVNREDENEDFDYVLPFMMNYFYDVNHPLPYAISYFMQCCSAYHMSLLFLSGDLLLISMVQLVNMHLGYLISRIESFQPTGTDDDIKVLGPLVEYHIEILDYTERIDNTFSLATLLNYGGTCLVLCLIGLQIVQGSDTVSVIKFIGFLVSTIVQVYFVSHFGNNLKDLSTGISDAYYNHPWYTGNYKYMRMLVLPIARSQRYAHLTAFKFFVISMDSFKSLCTLSYQFFTLLRTKIENNGA

>BcucOR22

MSSEARTFSEFIRIPIRFYQTIGEDLYEHRSPYRTRRLLLKSLLYIGFINFNLLVVGEIIYFIKALNSFATVLEATGVAP

CIGFSFVADFKQMALTAHRQTLREHLDQMEELFPKTTAQQVEYKLPQRERVMRRVMAVFTLLCLAYTSTFSLYPALKASVQYWLLSAPVYERNFGFAIWYPYNATQKTWVYWLTYMGQVHGAYLAGVAFLSADLILVASVTQLCMHFDYISCCLEDFAGATPKRSAQEDLQYLQALVVKHAKCLELSEHVNSIFSFSLLLNFLTASLTICFIGFQVTASSTEDIVKYIIFLTASLVQVFVVCYYGDELMTASLRVGDAAYNQNWFDCDRRYKRLLTIMIMRSQKPACIRAPTFPPISFRTYMKVISMSYQFFALLRTTYSGKGN

>BcucOR23

MDKLAVLSSRIFPSDPSKGKIGSIEYNVWLAQLFGVPVVGLKAESPLMRIALGIYGVLLTLLVTFIYTGFEIYDMILCWPNLDDLSQNICLSLTHIAGVLKVINILYRLDEVAHVVRRIEYSAKTYVISKRQLVAFYRGEFENKIPLTIYASLVGFTGVLGIIYLFYNPVGVAGQIFPYRVKLPDWMPFGLQLAYMGISVLVFALQIVSIDYLNVTMINQIRFQLKILNLAFEELKLDCGSKKKAHFNHDKRLQTIVEHHCLLRDLRIDVEEIFRMPVLLQFFTSLIIFAMTGFQAIVKTENSNGAALIYCYCGCIFCELFVYCWFGHEVSEQSKTLTTSGYSSHWFEFDQRFKKSLLIFMCNSQTPFVFTAGGFMSLSLPSFTGILSKSYTVIALLRQVYSR

>BcucOR24

RFITNVMLYRPRLNNGKLIPLSWPIVAYRLLNNICWPLRDHASLLERLFDRFFWSLGFFIFVQHNDAELRYILVNNNNLDEMLICGPTYLILVEIHLRAFQLGLNKEAFKRFLQKFYAEIYIDEPAHPKLYASIQKRLRPIWFYSLLYFSTLSSYVIMPFVNYLNNVKAPLYKMYYPFDITPNPIYVAVVLSNIWVGFTVITMVSGEDNILSEVMLHLNGRFLLLQQKLGQDAERLLHAGDDRNIADDLRQRIVEAIEENVRLYKFAEDFEREFSFRLFVSLSFSAGLICVLGFKVYTNPMASFGFMFWICAKVMEMLLIGQLGSSLIYTTNEISSTFYKSHWELVIQKSSDTNANVRLMKTLLLAITTSQKPFVLTGFNYFSVSLTAVLKILQGAGSYFTFLTSMRK

>BcucOR25

MNFRFLSRTFPLRDYYFYVPKLCLGALGFWPLDTCEPGAFNVWAWVNLIILTIGVVTEMHAGCLALRTDLELALDTLCPAGTSAVTLLKMALIYYYRQDLAWVLKRMRDLVYERDVSINSVKKHIVRAHAVMAARLNFIPFVMGFITCTSYNLKPLLMTLILYVQGREPMWKLPFNMTMPPFLLRAPYFPLTYIFTAYTGYITIFMYGGCDAFYYEFCSNTAALLELLQNDLKSILSFGGDKFTLTAEESTVLEWRLVQFIKRHNDIIELTRFFCKRYTVITLAHFVSAGLVIGASIFDLMTFTGFGIVIYIGYTIAVLGQLFIYCYGGSMVAESSVQLATVAFGCDWHACNPRLRRYVLMIIIRSQRAISMSVPFFSPSLVTFTSILQTSGSIIALASSFK

>BcucOR26

MRKLIDLLYGRGAAKFESSESFQLLFKSWTFFGFTTMQPYRLGHLLHMCLCWYCLALCPVAFYMGYIQTLRTAPVSIQLNLLQATVNVVGLQLKCIVILILKTHLRSATPIFAHLDERCHSAESREEIKNCVVYCTRLFAIVGFMYHFYATLLYLQALVLHTYPLNTWLPFTDYIHHPTIKYFLHFIIEVFHISFQLSLQATNDVFPPIYIRNLRTHLNLLTKRVSRLGKNTEFTDEQNYDELVDCIVTHQELLEAKNIVASVCSITVFIQFIVVAIANCISLLNFFVFADRLEQLSTFSYYISVLIQIMPTCYQASMLEEDSAKLPNAIFHCNWLGMDKRCRKLIIYFMQCAQEDITFVAFKLFKINMTTNLSIAKFAFTLYTFMNNMGFGQNMKDLLE

>BcucOR27

MRNIADLYYGRGKDDFGTSESFVLLFRSWRLLGFITAKPYRITNLMLVVFIWACLIFSPITYFGGLVLAMKELPITVVLSILGVAINCLALPTKAVYIRANLYRLYDVETIFKRLDARYQRPQDQLLIRDLVKSCTRIFGIFFIVYWIYGIASALAGLFAHKYPHDSYVPFIDWMPDSNLKFWLHFSFEVVYAQIMLQTNVTNDSFPAIYIHAIRTHIKLLTERVSRLGTNPDLSEQENFDELVDCIVTHQEVLKISKIVGSILSLTTFFQFTVYAAIVCICMLNMFVFGDATTKLVTVSYIIPVLWQTIPTCYQASMLVTDCTKLPLAIFHSNWLALDKRCHKLIIYFMQRTQDEISFTAIKMFQINLGTNLSIAKFSFTLYTFINEMGFGETLKERLE

>BcucOR28

MRTITDLLYGRGAAKFESNESFQPIFQSWSMIGITTLKSNRTRDVLHMCFCWFCILLSPFSFYMGYVQTLRTKPITDQLSLLQAIFNVTGLPLKAIFIKISQTHLRTTEAIFVRLDERCQSAESREQIKKCVVLSARIFTVVGSVYHLYGSTTILQAFFTDNYPLQTWLPFTDYIPQPTIKYYSHFMFEVFHIYFLLTVQFTNDVFPAIYIRNLRTHMNLLTERVSRLGSNPEFTDDQNFNELVDCIATHQDLLVVKDIVESVCSVTVFIEFVAVATAHCICMLNFFVFADRFQQMVILSYYLGVIMQIMPVCYQASMIMEDSAKLPDAIFHCNWLAMDKRSRKLIIYFIHRAQENMTFVALKLFKIDMTTNLSIVKFAFTLYTFMNNMGFGQNMKELLE

>BcucOR29

MQRLSELLYERVESDCETNKPFKLLFYFWTWIGIKSKPRGFLSTLHMVCVWIMFFFTPFLATVGFIRKWKVSTVTECLSTLQAFINAIAASAKAFAVLMYFKRIKNVEPIMKDLDERYKKPHERQQISDCVASCTRLYASIWFIYYLYGNMSILTAIVLHKQPFGGWYPFLDVIPNPTVHFYSCFIFETCYMYLLLTAQYLNDLFPTLYMRTIRTHIQLLRERVSQVGADPDMSDEEKHQQLIDCIDIHQQILKVVNIVGSICSPTIFIQFSVVAIVHCICMVNIFIFADNLNLMITIIYYITVGMEILPTCYEASTLEMESSKLPVSIFHSNWLALDMRGRKLIIFFIRRAQVDVSFVAMQMFKINLQTYLVIAKFSFTLYTFVNEMGFGQNIKDLME

>BcucOR30

MPEDLFRIQRNCLRLMGHQDIYDDDNETAGDEQKSKSWQQRCFRHSQTLKYALLLLLMMSAQLPMMDYIIYHIDDLALATACLSIVFTNVLTVIKTSTFLAYKREFKSLMAEFERMYDELHEAGAKRCLVTVNVGAKRFVKLYFYSVSCTGLYFTIKPLVSMIWAKFQTKPLVLELPMPMRFPFDFESPPGYQFAYIYTILITIVVVMHATSVDGLFVSFTTNLRGHFQALQYFIETNTYDKSDALIQRELRIYVHYHVRLLELSQSVQRIFKPIIFGQFLMTSLQVCVIIYQLVTNMGVIMEMVVYCTFLSSILLQLLIYCYGAEFLKIESSAVGTAVQMSQWYNLPPRHRHVLRLMMLRSQREIIISAGFYEASLANFMSILKAAMSYITFIQSIE

>BcucOR31

MLLKFLSQNYPTEKNVFLIPKFALRIVGFYPGDSKSRLMHAWLIFNMFVLVYGSYAEFMFGVHYLSIDVVRALDALCPVASSIMAIVKLSFLWWHRAELNCIIKRVAELTAEQKSPLKSYYKHRYFTTATRLSAAVLCFGTTTSTLYTIRAAMVNYSSYLREEKIPYETPFKMIFPQPLLSMPIFPLTFILSHWHGYITVAGFAGTDGLFLCFCKYIGTLLKALQFDMKDLLSDVDSVTRKSTSEYEFRESLKLIIARHNEIIDLVKRFSAVMSGVTLAHFVTSSVIIGTCVVDVLLFSDLSGIFVYSVHTMAVTSELFLYCLGGTVVIECSSQLATAAYDSQWYTHSVEVQRMVLLIIIRAQRSLVVKVPFFAPSLPALASILRFTGSLIALVKSVV

>BcucOR32

MVLHILVLFIITIYLKYDNGDLEFLISCGIQVLLYLWAILIKVTFRRIYPDLVNGIVDFVNEEYVVHSALGFTYVTMKECVDRVNRGIRIFVVCCFAAVIFRIILPIIFNERTLPLPCWYPLDYEAPIIFQIAYFFQTVAQLQLSAAFSISSVYFIAVCF

LLSGQFDVLHCSLKNIVATTYIYMGASRNELIELRDNERIPGEEINEFFVAKELPFDLDCLPHILNPADTARTRNFREAFNYALESCINQHLFILNALRKLERLYNMVWFFKMFEVTLAVCMCTFDMVKSSGGKPLLQIFALSQYVCLALWEIFMICYAGEIIYVNSQRCDEALMRSPWYLHHREVRIDILLFLMNAQRAFKLTAGFYPLRLERFRGIITTSFSFLTLLQNMDERT

>BcucOR33

MFKSDLGIKSYFHLQKFTFRRLGIDMTTREARVTQICFLIIQIFALASILIPIAVYSWQHIDDIAEVSNAMAPFMQATISLWKVWRVIYRRKEMAELCENIYLISAKASKLELAHLIQENNRERIMNTAYYYSVLNTGVMALTAPVVVSFIQYLRLGEFSYITALKATYPIDYARPLNYFLIWLWTAVAIYGVIYVSVPVDSLYSWYIHNLVGNFKILQSKLVSAESIAESTADLGKRRELIYYCVAYHQRLITMSEQLNIIYQPIVFVQFSLNGLQICFLAYQIGSGVVAMVDLPFLLLFLISVGIQLMIYCYGGQHLQNESVNVSKSIYQTINSSPWPNELRKVLLISLMRAQKPCKLTGIFFDVDLPLFLAVWRTAGSYVTLLRSVDQKTM

>BcucOR34

MDFVHFFWFPNALYRVVGYDFQQLARAHWRQVIMKAFLIFTTISGICTRIYMLFQLRELILNGDILNSIRLGVYISYAIDSNVKFFVFLLNAQRLRVIYQTLYNEYPVTPIERKLYQVDKYSFKRAHLMMVVYLSVTNSILLGPMLQSIFMYFVNLFHYGYAVAEFPYLHPTPVLYNFNYCTPHYYILIYISEYLNGHFCTTTNLGADLYVCTFAGQFCMQLEYLGNSLETYEPRVENSKTDCEFLMEWIRKHQLMLDLCCELNEVFGTTLLFKLISNCTVFCAIVVQLKLEGFGIGFFNFLSFFFVTVAQFFMVCQYGQKLITISEDLSLCAYKNRWYNGSQTYKILLFNIIARAQKPVKLTARGFQPISLATFQIVMTMTYRVFAVLQRALD

>BcucOR35

MFDLIKGRGRTVFASRDAVIYLFNSFRYIGFNPPATYRVPYFMYSAIITFFAVVFSPVIFNVGWLRDRNKLSVMEILTCVQASLNVMAVPLKCITLAMAHKRLRGIEPMVTELDERYTTAEDKAKIKQCAVTGNRLVFGFAISYFLYETLTVVSALVGGHAPLSLWIPNVDWHRSTWEYWLQVSFDSAVLFFLLYHQVLNDSYPAVYIYIIRTQVQLLASRVEKLGYDEQKSVDENYQELLECIVIHQKILKIVSIVESVVSITVFTQFLVAALILGVTMINIFIFADLTTKIASSTYFFCVLLQTSPTCYHASYLLADCDELRIAIFHCNWIAQNKRFNNLLIYFLHRSQDSIPFFALKLVPINLATNLSIAKFSFTLFTFIQEMGLGENLKG

>BcucOR36

MPNLIRVGGACIYKSRDSLTYLFKIFTFVGINPSEQQSRKYYWLYYSYSLTVNFICCLFSPLSFHIGYIKLWHVLNNNQLLAAIQNAVQVTGIPIKILVITWYMKRLRQAFKILDQLDVNYTQHEDLAKIRECVRRCRKIVLIFCLPYYSFELSTIALGLLQKRAPLAAWVPFLDGQRAAWEYWTIVLWDTFVMFILLSYQLGGDTYPLIFLNIIRTHVQLLVTRVSRLGRDGALSADEHYAELLACIRTHVQIVSIANIVAPVISVTLFTQFATTATTLLTWFGNVEYPENIISFAFFSCQLLQIFPCCSSASQLIADCERLPDAIFHCNWVDQDRRFRRAILFFLQRTQKPMRFWCLKLFVVKLETSVAIGKFAFSLYTFIQESDVGRKSNN

>BcucOR37

MSLIINSWDAFKYHWRVWDLSGFRGPRRQSIWYIPHKLYMIVITLLFPIYYPTCFTVESLLADNLNDFCEVIYIAMADVTLNIKFLTLFIVRQQLLELRPILKRLDARAKTEEEIGVLQDGIDSAKKCFLIILRLFYSAFVTSQLMVIFSGEARLMYPAWYPFNYKATRTKFWIAYGYQTIGFLVQCTQACSVDTYPQAYMRVLTAHIRALSLRIEAIGRKSFNCDSSEFIPLTKDEMKRNYDELVSCIKDHKTIIELFSTIQKPISGTAMAQFVCTGVAQCTIGVYMLYVGFNISIMLNMAVFFVSVTMETLILCYYGDMFCQECEQLSKAIYNCNWTVQSSEFKKALCFFLLRSQRVNVLMAGNWIPVRLPTFVMVVKSSYSIFTLLSSFK

>BcucOR38

MFDLVKGRGRNVFASRDAVIYLFNTFRFVGLNPPPQCRFLYYFYSGIITLFVVLLSPIIFNVGWIRDRNVLSVMEILNCVQAALNVVGVPIKSITLAMSLERLRSVEPLLSKLDARYTEPEDVAKIRSCAIIGNRIVFGYIISYMMYETLTVVSALLGGHAPLTLWIPYVDWHRSMWEYWLQVTFDGAMLFFLLFHQILNDSYPAVYIYIIRTHVQLLSNRVKRLGTANKSQDETYHELQDCIVTHQEILRLVRVVEPIISLTLFVQFFIAAAILGTTMINIFIFADFATRIASLTYLFCVLLQTSPTCYYATHLQSDCQDLTMSIFHSNWLAQGKRFNTLLLYFLHRSQADIPLFALKLIPINLSTSVSIAKFSFTLYTFIQKMGVGKNLK

>BcucOR39

MTQTSAAERRERIGIARVLMCALQFLGLWPMWNERPQSAASTQCSIWLRRYYRYLLHVPLTFTYNTLMWIEALTRWERADHILYISITEVGMMALALNFWRMERCAWHFMHELNHSDCLALRNDVERDWWCGQQRLFARIVVCYIGGGAGVLFTAFGATLFMSGYSLPYDYWLPFEWHNAQNYWYAYGYEVIAMSLTCISNVTMDMLLCYYLFHVALLYKLIGMRLMALQHLSEPLAVQQLMNIIELHKRVKRLTTQCESLVSVPILVQIVLSAFILCLSAYRLQSMQISENPGQFLAMLQFASVLTLQIFLPCYFANEITINSDALTTCVYNSSWEQFSPSTRKQINLYMELLKRPAHIKAGDFFLVGLPVFTKTMNNAYSLLALLLNMSK

>BcucOR40

MSATEAPISGVTVFHFHDVTWRYVGQKPPTEKLHRYLYYIYSLLLNVIITIGYPTHLMIGLIQSESKSNMFKNMSISFTCLACSIKTFAFWWRLAEVQKIYAIISKLDKHIAHSDDYRLYKTIVLRRAQRVLYFILFIALGAAFSSEVATIIGGLLVEWRLMYPAYFPFDVERSFWGYAVAHIYQCIGVTVQIFQNLINDTFPPLALAMLAGQVRLLNLRVARVGHVASDASVRRQPSNSEFLLCVEDYKDLLEFRIAIQRICSIGTFVQILVTAINMGVVIVYLIFYVDGIFAYIYYIVFLIAMPLEIFPLCYYGTSVQMEFEQLTYAIFSCNWVFENATFKQDLRIFTEQSLRKQIVIAGGMFAVNLDTFFATLKGAYSLFAVVVQMK

>BcucOR41

MKLEQFDNISGGRRVIKILKLLGLWHYGGVFRMPYLLYSGLLHSSFTIPYTIMMCLDVVQASNLEKFTNTMYMTLTELGLVAKLVNVWIYSRLLVDFFAAFTNDKLYKLQDAEEQLSWRRTQGNYARIAFLYFAMSLGALASAFVGVLYSEDYELPFPYAPPFDWRTPRGYWYAYFYELLAMPVTCLSNCAFDMIQCYMLLQLSLCFKLISGRLERMGALREDSASRGFSEFRLHREFVDIVRLHARTKKLSQQCQTYISFPFLIQIICSSFVLCFSAYRLQKVPIQENPTQFLTLVQANLIMVLQIFIPCYCGNEIIQHSSGLNNATYNAEWFRCSPKMRKYLVIYMEMLQRPVRVRAGDFFDISLAIFTKTMNNTYSLMALLLNMNK

>BcucOR42

MTAERVRPTDSFAKLIKMVRLISSLVGADVSTVNYRVNIITVILILCIVIYFIFTATTVASVFSEDWTFLLEASCMMGSGLQGCTKLISGIFRTKDVSSMRLELEEIYRTYESKGQSYCKVLNESCDRVRKVIKMVGYIYASNIAGIILLTTVLMLTSDRKIYIMQFFIPGVDADTSFGYLLTTAVHMVVFLAGSFGLFGGDLFFLIYLGQPELFRDILILKVEELNEAVAQKDDNIESLLINIIEWHQYYMDFNNRCNDIFYYIITMQILTSGISIICTMYIILMGDWPGAYLYILVALCGLYLYCIIGTRIQTSSETFFEELYNINWYELDIKKQKMMILILMKSQNPSEIKIAGVLPLSVETALQITKSMYGIFTMMLQVMAEEL

>BcucOR43

MAVKAIRPTETFTKILNFFHLICSLVGADLSNDNYRVNIITVIVILSIIIYFIFTATTVASVFSEDWTYMLEASCMVGSVLQGCTKLISAFIFKNKICGMRAELERLYAEYEVKGDEYVKTLNKSCERMWQITKVVGQMYLYAAVGIVVTIIYFVIATTQRVYVMHFFIPGIDVNTQTGYLITLGVHAVVFMSGAFGLFAGDLFILLFLTQPMLFVDLLVLKVKALNEAAAQKTNAVQRLLIDIIEWHQYYTDYNKRCNHLFYYIISVQIITSGISIICTLYIILMGDWPGAYMYILIAFSGLYLYCILGTKIQDCNSAFCNELCNINFYDLEVKSQEMIVLIIMKAQNPVEIKVGGFLPLSVQTALKITKTIYGIFTMMIRFLEEEQ

>BcucOR44

MDGAKGFVNSFYKTQYIIFKLLGLFSFPADYSSFWHYMHKLYFWHVAIFWMLLFDISMWIQIIGNISNLNEIINVFYLFSMAIAVMAKFVRIRLKNNSYAAMFARMHDNDLLPVNAAELEKFTQSSHLSCRVRDSYMYLSLTSLTLIFVTKLIAEPGELPLSIYIPISVETFWRYLIAYLFQFISLSLCCWLNISFDSLGASLFIYLKGQLDILANRLENIGMNLDMDDNMINRQLKDCIQHYVKLRNITEIMEDLLSIPMSVQMMSSVLVLVANFYAMTFLTDPSDYVTFIKFLVYQLCMLSQIFMLCYFANEVSLRSAELSYSLYSSEWTRCSQINRRLMLLMMAQFDVPMRIKTINRCYSFNLPAFTSIINSSYSYYALLKKMKD

>BcucOR45

MKRQTSPSDIYYKMLSVIRFCSRRIGCDIIAEDYKINLNTMVVIVAIGAYYLCSVHTIMKYIATDWTVLLDVFSPVSCTTQGMVKLISVLLYPELYRKLALDIGLIYEKYQELGAAYKEKLLEWNTNMKKLLIAIAIVYFLTALLILCTPIVLYIFKGERHLILLCQVPGFEVDTFHGYWVTNAFNALCVFIAAFGLYAGDLYLILFLTHSIFFRDILTLKIDDLHKLIEADDKEDRQTKLVKDIVEWHQYYLEFNDKCNLLFFWTISAHIICTTLGILSTLLIAMLKDWPGAYAYLLVCFIWLYMYCILGTRVEINNDRFCTGIYDINWYALDVRNQNTIRLMLMQSQAPKNITIAGVEPLSVSTALKITRTIYSLVMMVLRFQNKQ

>BcucOR46

IGSVDNLQAIVNNFTIFQLTILHPGENTSKMSHKPLLTATTLDTKEAFSYIWYCWGFFGMYPDLYERRLNWIYLILLNLYCGVIYPLLYIGSLLTPMDLNQKLANISVAVPILYTLGKHVIIVYYLRKDLPKALRQLQALDRLAERRPKDREYMKRMVKNCHWIFLASSVSFWFALLSYGVLEMFRHKLPFEGWVPFDWQRTEFAYVCACALQLFGLGIETTNAICCDTYAVTYLVLLVAHLRILNRRIARAGNTGDGSDAENYRELVACVEYHKECISYYNSIRPTLSGTCFIQFLSTGLGLSMPAIAFVGGSFSFSHVIKFLIIFGAIIIEVAPCCWFMDEVMAEMHKLTNSMFSCRWYDQNLKYRKALIIFMQRSQIAHPVLAGNIIPVSLETFTNIIKFAFSLFTLLNQLSHN

>BcucOR47

MSPSPLSLPPPPPPLAAVDTRSFFKLHWTCFRVLGINAPSSNTYYSVLLQVLVTLCYPFHLALALFSSPDASINIQNLTVCVTCVACSMKFVFYATRLSRIRELESIIAALDARARSLCERRYFVQLRKELRRITICFLCIYTVVGVTAELMFIFHNERNLLYPAWFPFDWRASNLKFYAAHSYQIVGISYQLLQNFVNDCFPTMALALLSAHIKLLGIRVSQIGHETASLGANEAELLHCIKDQEQLYNVLNIIQNIISLPMFLQFTVTAINLCLGMAALLYFVDAPFDRLYYLAYLLALPLQIFPICYYGTTFQLLFDKLHVEMFASNWVEQTHKFRKHMILFCERSLMSQTAMAGGIVRIHLDTFISTCKAAYSLLAVIMKMNE

>BcucOR48

METKTRPSDNFHKLLKIIRISSSLIGVDVIDENYKFNYVIGFVMVAIAWNFMCSIYSIYKDVTTDWTVLLDVFSPISCAAQGTIKLCSLMLYPKLYRELAMDLVEIYKKYQAVGQKYETKLFEWNKSMKNILIIGALVYFLSSLLALIAPIFLYIFKGERHLIIMCQMPYVDVATDHGYFITIGYNLLCVFVAAFGLYGADLYVFLFLTHSIFFYDIFALKVGDLHEILRQDNKDKRIKPLINDIAQWHQYYLDFNDKCNQIFFWSITAHILCTTLGILSTLLIIMLKYWPGAYPYIFVCFVWLYMYCILGTRVETCNDQFCDGIYDINWYDLDISDQKTVSLMLLQSQVPRLITIAGIEPLSVNTALKITRSIYSLVMMVMQFNE

>BcucOR49

MAYEAKPPGVKQLFRTHWTVWKWLGQVIHPQYPKLHIAYTVLLNVGFSIGYPLHLLLGLLNLKSLQEVLLNLTISVPVAVCTLKYFNIWRNLDKVRHLEQTYNTLYARIDHPEEWLYYRKIIIPYALKVLHLFYFICVGTAITSELTLLIVGFAYEWRLMYPAYFPFDPYASTGGYVAAHLFQIIGLLVQLAENLVSDTYGGMCLSLLAGHAHLLGQRVARIGYDEQKTQEENNRELVDCIVDHNMLFDCHRTLTDIIGLGLFMQIISASLIMGVVIIYLIFFVGNSFEYVYYGLFLFACIMEVFPTCYYATYFEIEFEKLTYMMFSCNWMDQNRQFKQNLIVCVEQSLNTRYFHVGGMFRINLQIFFATCKGAYSVLAVALRLK

>BcucOR50

MNAIERNTNFTRFTAGPVRYFKFIGICLQPPEMRSYKYTRILIVLTILLMFLHQIGYILTPGRTFAEQSTAAGLLNYTTVSGGKILFLIYNRHLLLNSHCQLAALYPSASLERRYKLEHYLRIYAHVQTLLYNFFKYILIIYLLYPLVQSCYDLWSNGVYSYLMPTLFWYPVPLEQSLLVYIVYFLFACFCSFCAGLIILSADLCLFSSVSQLMLHLDLLAQRISELQPAEPESLSALKAIIEYHQKILILAHDVNSTFAPTILFSLASSSFILCFSAYQLLDDVSFIFALKVLLLLGYEMKQVVITCYYGDKLMDSSANLFNAVYAHNWADGSPAYKRLVLFMLVRTYRPIALKVAGISDVSLITLKQVLSTSYQIFTVLKTA

>BcucOR51

MSSSNPSLQSVNSVVLYREFWLCWHAVGISTAYQKHLCALYDLLINVLVTIFYPIHLIVGLFLNPTPADLFQNLSITTCFVCSVKHYLLRRKLPQIRVVQALLADLDKRVEDAEERAYFEKQLVVGAKNVVKLFSIAYGGANMAAISATLLSKERRLMYPAWLPFQWQASTFSYCAAVIYQIAGVTIQIVQNLANDIYPPMSLCIIAGHVHLLALRVAKVGRDGKKSLKQHNQSLIECIEDHKKLVRIFELTQETLSQAQLAQFISSGLNMCIVLFYLIFYVDNVFSYIYYAVYFVSMAIELLPSCFYGSMLIYEFQQLPSAIFKCGWLGQSREFYQNQRIFVQLTLKEIVPLAGGVIGIQLDSFLGTCKVAYSLYTVCNRMK

>BcucOR52

MRRLLGPQVPIERSFFRIPRFSARIAAFWPQTFNRSLYWLTALRFCVNTFAVAVGGIGETLYGFVYLHDLFSALEAFCPGITKVISLLKMTIFFVRRKRWLHVIDGMRQLLLLDTSAEKRRIMEPLASFGSVLSFVLLISGSVTNTFFNILPLLKMGYYKSQSLDVPLLLPFNVILPEMFVNWPYYPATYLVLTLSGAMTVFSFSAVDGFFLCACVYTSALFRILQHDIRGAFAELQEQEHSTLVQNMRIQHRLAVLIERHNKIIDLCSDFASEFSLIILMHFLSASLVLCFSILDLMLNSTSIGVLTYIFYSIAALTQLVLYCIGGTYVSESSLKVAEVIYDIDWYKCDVRTRRMLLIMICRSQKAKTIQVPFFTPSLPAFRS

>BcucOR53

MNNSPPVDSRQFFRTHWRLWLLLGCVREPVHYQLLYRLYSTVVNALIMLFYPGTILIALYNSANLTDFLQTLPICAAALACSAKYISYYRRLGLVRQAEQIFNALDEQVLLPEDREFYAGIHRGTNLILNTLRGLCIFFMAITVMAFASSIEERGLAFAIELPFDWRKSSVAYVGAVGLELLLLSCDLLQSLANDSFPAIALCVLSNHTRLLGARLARIGHTSKDVQANIREMQRCIIDHQRLYRLQAIIEEIISMPVFIQYAVTAFQDCFTLITFIFYTNTVSDKVLYLTYLLALQLQIFPTCYYGTACAQSMDDLQQEIYASNWVEQNQVYRRLVTIFSQRTLKSTTTYAAGLIPIHLSAFVKTLQGAYSFYTFVDGVRKV

>BcucOR54

MAIDTMVNFRTIVRILTLSGLWRGMKGSRFERYSRYYQLFMHFIVTFGFILLLSLEIIYSDGLDHAIDVLKYLLVVMALGIKVLNAWYYTRQITEVMYEWENSELFVLRNDDEKQMWAKTQKTFRKLGMTSFGLGFNSAMCALLGVLLMGATEQPYALWMPTNWRDKYYWQMYIYQCLSMPFICFSNVTNDVFQAYLLLHLTLCFRVISMRLERLANAGADGAITAELMNDIKMHQRVKEMAISCEHIISLSMLTQITLTFLIICFIIYNLANANFREDPVHCLAMLQYALIVSLQMFLPCYYGNELTLESEKLSINLYSSDWTGMSAYNRRFIFHYMESLKKPLVLHAGSFFEIGIPIFAKAMNNAYSLLALLLNVNDDEQ

>BcucOR55

MSTAVEDNPLLSINVRLWKSLSVLFARDWRRCVAFVAPICLLNAMQCVYLYQQWGDLATFILNTFFAVSVFNALLRTCLIIKNRDKFEALMEELVTLYDNIEESGDDYAKSVLAAATKSARNISIFNLSASFSDLIVAMAYPLFQEQRIHPFGVALPGIDVTRSPLYEIIYIGQLSFPFTLSSMYMPYVSLFASFAMFAKATLQILQNNLKNLCDNMKTKTEEELFELLRTNIAYHARIARYVSDFNELVTYIVLIEFLLFSCVICSLLFCINITTSTAEKISIVMYIGTMLYVLFTYYWQANGILEMSLLVSDAAYEMQWYNCSPRFKRTLLIFIGRTQKPLQIRVGQMYPMTMEVFQSLLNTSYSYFTLLHNLYND

>BcucOR56

MNQPINSNVFFKIHWLGFRICGGDSTVNKYRLVYLAYALMVTVLVTVCYPLHLALALFRNGSLGGNIKNLAVCVTCIACSSKFLIYTRKLRIMHEIEQTFAELDARVSSEVERKYFARLRISVRNVVSVFVCAYAAVGMTAELAFLLSKERSLLYPAWFPFDWRASTRNFYVANVYQFVGISYQIFQNFIDDTFPPITCCLLSGHIKLLGIRVSRIGYDCVNRQDNERELVRCIKDQKNLYKLFVLLQEVMSWPMFIQLTVTAFNICVAMFVMLFFVDTPFERLYYLVYFVSMPLQIFPICYYGSSLQHLFSQLQYDVFRCNWTDQTPRFKKQMMLFTERALKTTTALAGGMIKIHLDTFFATVKGAYSLFAVIMKVK

>BcucOR57

MSFCEELFNWSLVFMNRAGYFDRHRRASVLLIPPISLCVAAFYRTYILRNDFDEVIVNLFKISGATVTTMRAFVVMYKSQAFLDFFVFVEKWYEEQQRDGEEVTLRKTHEFTQKIRKAAKTLLIVTMIILSYMVLIQLLATVGIGYRKLIVDVAFPGIDFYISPNWEMMSFLQCLYVAPFTFVSYVSYLCLTLIAISFGIFLMKDLQFKLENMNDMTDLEALNCIKKCVKAHVMIIKYHNHLEALFSVGSFADVCIFGIIPCVIIVLSTMDHDISMLIADIQMAAMVMTSTFIFFWVANNFVIESENIANAAYNCNWVDRDKEFRKYIPLIIGNSQKPLQLTAGGIKPINMEFFLTIVRCTYSLFTVLFTMKTGGDS

>BcucOR58

MPSYQNLPSYSVNVKTFVKLGLIESSNPTRRFLLGLILIVTYIGQLTNLFWTWNVDIGETGITNFHVLALMTHCFLRFMVIVRKDKKFERFFEGIEQWYTDIERNGDPQIVSTLQEITDKTQKLTRLSFYASTVAALAVFIYSFSFEGRRLLLTVQYPFFDVLQTPYFEFFFLIQVVWLLPTFLLIYLPFTNIFLTSLMFGELTLKDLCSKIRKIQSENEMTMLNEFKECIAYHNKIINFRNDLEYLISIDGFFHVALFSLMLCMLLFFLSLVQDYRQILSALAFISFNFYVIGITYYYANNFSNESLKIANAAYDTPWYEGNSELRKCVQIMIARSQRPLELKSGGLYPMTLENFQAILRMSYSYFSMLQGFSQQ

>BcucOR59

MTLSGLTVFRFNNANLRYLGLVTPTNRRYRYVYYLYSLLLNTLISICYPAHLMIGLFQMEQKTDIFKNICVIFTVLACIIKTFALWWRLADIEAIYAIIVKLDARITQPEDVHFYTSDTLRRAKHVQYFLILLGVCAFAASEAATIFAGIMGEWRLMYPAYFPFDISASAWHYTAAHLYQAAGIILIFQNLSNDSFPAMALTLLAGHTRLLGKRIARIDDAAVSVAQLQRCIEDYRNLLDLRLTIQSFIAVSAFVQLLSTGVNMCVVIFYVIFYVNDILWYIYYMVYLVAMPFEIFPLCYYGTCMQLEFEQLTYAIFSCNWLDQSAVFKRNLRIFTEQTLRNQIVVTGGMFPVNLETFFATLKGAYSLFAVVKQMK

>BcucOR60

MASYQNLPLYSVNIKAFVKLGLIESNNSTRRFLLGIIIIVTYIGQLTNMFRTWDVDIGETGMNFHVLALVTHYLLRFIIIVRKEKKFERLFQGIEPWYTDIERHGDPHIVSILQKITQKTQRLTRLSFYASVVATLATFIYSLSFDERRLLVTVQYPFFDVLQTPFFEFFFLIQMVWLVPTSLLVYLSFTNIFLTSLMFGELILKDLCLKLRNIRSENEMTMLKEFKDCIAYHNKIIDLCGDIEDLISMDAFFHVTSFGMMLCMLLFFLSMIDDLELIPAVLVMMGFDMYLIGFSYYYANNLATESLEVANAAYDTPWYRGNLEMRKCVLIMIARSQNPLQITAGGLYPMTMENFQAILRISYSYFSLLQGVSQQ

>BcucOR61

MRFCEELFNWSFTFMKRVGYFDQHRRAWLLLISPISLCITAFYCTYVNRNDFDEVIVNLFKISFATVTTIRAFVVMHKAQAFLDFFVSVDKWFQELQCDSDEMTLKRTHEFVRKVNKASKALLIITTIILSYIILVQLLVTVGIGYRKLIVDVAFPGINLYESPYWEIVSILECLVIAPFTFFSYVSYLLLTLIAISFGIFLMKDLQCKFESMNDMTEQEALKCIKQCVKDHVLILKYHHDLEVLFSVGSFADVSFFGLIPCVIIVFVTMDHDMSLLIVDIQLSVMVMISTFIIFWVANIFCYESENIANAAYNCNWVDRNKEFRKYIPLIIGNSQKPLQLTAGGLKPINMEFFLTMLRCSYSLFTVLFTMKTE

>BcucOR62

MFDDLQLIHMSVRILRFWSLIYEHTWRRYVCLSMTTFLVFTQLYYMFRTSEGIVSIIRNSYMLVLWFNTILRAYLLLYDREKYEKLLSDLEKFNFDLKQSKDFYIQDLLAEVNKTGKYMAQGNLFLGLLTCFGFGFYPFFVNERVLPFGSMIPGVDEYKSPFYEFWYFYQMIITPMGCCMYIPYTSLIVGCIMFGIVMCKALQFRLKTLHRVRHDEPLIRKHVRECIRYQLCIIDYIARVNSLTTYIFLLEFLAFGTLLCALLFLLIIVDSSAQAIIVCAYIAMIFAQILSLYWYANELREQNLAIAAAAYDTEWFTFPIPVQKYILLMILRAQKPPAIMVGNTQPITLELFQSLLNASYTYFTLLKRVYT

>BcucOR63

MPKHRKWLYALYSLIPNVLVTIWLPLSFVFSYATMSTEELVPSSLLTSIQVAINVIGCSVKIVVMAFLLPKLRTANVFMDRLDARCRVEAEIAELRKLVKQGNRFVVLFAMSYWSYASSTFLGSVIFGRPPYALYNPLIDWRKSKLEFIAASIMEFALMDVACFQQVVDDSYAVIYVCVLRTHMNILLVRLSRLATDDETNLDANLEELKLCIIDHKNLLGLYDVVAPIISLTIFIQFMITASILSATLINIFIFAEQLSAQIACCFYILAVVVEIFPLCYYAQCLMDDSERLSQQIFHSNWVAQDVRFRKMLVFFMQRTQRVMELNAGKIFPITLGSFLNIAKFSFSLYTLIKKMGIRERLGLE

>BcucOR64

MHPTRRHRLPYYIYSGCINISLGVFLPATMIAKLFFIENLSQLIGLLYLGVTLTMATAKQWSLWLHRSKLLAVNQYLDKLDARCMPHAVDRQHIRTAIRICHLYYAGYMFVYELCSSGFAYIGFTLRQLVYDGWFPQFYADEATNLTVTLIYQNFAVMSFFVLQNVNNDMYPQCYLAMMIGHLRALTARISRIGKDGVLSEDENIAELTNCIEDHKNLLGYFACIRPVISRTIFMQFGITAFVLCLTAVNYVAFERDAAQMLIAATYIFAVLIEALPCCWYVNSLMEECGELTTALYNCQWYDQNRKFRKMLIIFMQRSQRTMLLMAGDLVPITLQTFLNVSCLGVQILEGVDFY

>BcucOR65

MNNKQDAVGRLDSSDALRYVWLFWRITGIHPTAKYRGIYWLYSLLLNISSSVLFIAFYVVTFFISTDLLETLTNLSVMVPLIYTSTKHLVVFYHIRGKLPQAAFHLQALDRRVELEPAACEHLRRLVQRCHRIFLAALAGIGVCLALYALVGIARHKLPFEGWLPFDWEHSLNAYILACAYQLFCLSVQSIYALCSDTYSIIYLLLLVAHLRILNARIARIGGACAAQCGEVANYQQLADCVRDHWECMKCISPTIAATIFVQFLSTAFALCTAAVAFVNADSSVEQLMKFLPYLLVVLCEIAPCCWLMDEAALEMLKLTNALFSCCCSDGN

>BcucOR66

MVRQLDTRAIFTRLFLTWRVLGVIDWPFHRHLRLVYDILTNTVVTVCFPLHLLVGIVFSTNQEEFFTNTVQGIASVSCVLKHVLYRFRMREMQRINEILGQLDDRVRTNEDYDYYKHHMERPCNFMVNFFTRCYFAVSIMALVTALFTGKLLYPAFIPLQWRTSVFKYAMTLLYQFTSVSLQAVQNIANDAYGPVVLCMLSGQVHLLSNSVSRVGHDKHDSVEDNYKELSLCIEDHKLLMSTTMAAESMASARYIVQFGGTCKTGYSFFTVIQSMK

>BcucOR67

MQRLSELLYERVESDCETNKPFKLLFYFWTWIGIKSKPRGFLSTLHMVCVWIMFFFTPFLATVGFIRKWKVSTVTECLSTLQAFINAIAASAKAFAVLMYFKRIKNVEPIMKDLDERYKKPHERQQISDCVAACTRLYASIWFIYYLYGNMSILTAIVLHKQPFGGWYPFLDVIPNPTVHFYSCFIFETCYMYLLLTAQYLNDLFPTLYMRTIRTHIQLLRERVSQVGADPDMSDEEKHQQLIDCIDIHQQILKVVNIVGSICSPTIFIQFSVVAIVHCICMVNIFIFADNLNLMITIIYYITVAMEILPTCYEASTLEMESSKLPVSIFHSNWLALDMRGRKLIIFFIRRAQVDVSFVAMQMFKINLQTYLVIAKFSFTLYTFVNEMGFGQNIKDLME

>BcucOR68

MAGKIFPFRITLPAWLPFYMQVAYIGVTDFMFAVQIVTVDYLNISMINLLRCHLNIIKSTFDELILDEQNARRDMKRDPNARMADIVEHHCILKSVRDDVEYIFNLAVLLQFFTSLIVSAVTGFQATMHSSNSNSMMIMYFYCFCIFTELFGYCWFGQEVNEQNNTLAARGYGSSWYHFDQRFRKSLAIFLLNAQQPFNFTGGGFIDLSLPSFTNVMSKAYSFIAVLHRMYGR

>BcucOR69

MLHQLDTRAIFTPIFIHWRVLGIIHWPFQRHLRLVYDILLNTVVTVCFPLHLIVGIIFSTNQDQFFTNLVMGIASVSCLFKHMLYRFRMEEMQRINEIFGQLDDRVRTKEDYDYYKRLMERPCNFMVNFFTRCYCAVSITALTTALVTGELLYPAFVPLQWRTSVFKYAVSLLFQFAGISLQAVQNIANDAYGPVVLCMLSGHVHLLSNRVSRV

>BcucOR70

FLPQRYVSDFNELVTYIVLIEFLLFSCVICSLLFCINITTSTAEKISIVMYIGTMLYVLFTYYWQANGILEMSLLVSDAA

YEMQWYNCSPRFKRTLLIFIGRTQKPLQIRVGQMYPMTMEVFQSLLNTSYSYFTLLHNLYND

>CcapOR83b/Orco

MQPSKYVGLVADLMPNIRLMKYSGLFMHNFTGGSGLFKKIYSSMHLVLVLVQFLLILVNLALNAEEVNELSGNTITVLFFTHCITKFIYLAVTQKQFYRTLNIWNQVNSHPLFAESDARYHSIALAKMRKLFTLVMLTTVVSAVAWTTITFFGESVKFAFDKDTNSSITVEIPRLPIKSFYPWNAGSGMFYIISFAFQCYYLLFSMVHSNLCDVLFCSWLIFACEQLQHLKGIMKPLMELSASLDTYRPNSAALFRSLSANSKSELINNEEKEPTDLDVSGIYSSKADWGAQFRAPSTLQTFNGMNGTNPNGLTRKQEMMVRSAIKYWVERHKHVVRLVAAIGDTYGGALLLHMLTSTIMLTLLAYQATKITGVNVYAFTTVGYLCYALAQVFHFCIFGNRLIEESSSVMEAAYSCHWYDGSEEAKTFVQIVCQQCQKAMSISGAKFFTVSLDLFASVLGAVVTYFMVLVQLK

>CcapOR7a

MDKLRAVIFDRVGLSTKDSFDLLYLNWWLNGNTSWKPHRLGHILHMTICWCLKFFAPVTYFKGFLIALSTSTITTALYNLQATLDVMVAPFKAVVIAKHMHRLRTLTEVFNRLDDRYHNPRERAQIDEGVIICRQIICFYCAVYSGYAVMTWLGALIAGKMPHYLWFPYFDSIPNETLRYWLQFTFEALFIHFMLNVSYTNDVFPVIYMRALRTHVKLLAERVSRVGSNPELSAEEHHRELVDCIVAHREILYIVDVVGAITSLTIFLQFAMAAATLCACMLNVLIFAERIGQIITIIYYMGVLLQTGGSCYQASMLEAESSSLATAIFHCNWLNLDKRSRTLLVYFMQRAQEDIAFTALKLFQINLKTNLSLAKFSFTLYTFMNEMGLGNDLAKSQS

>CcapOR85c

MCENIESFEAFLRIPSFFYRSVGVDLWNTNGGSIQRFIFYFGFLNVNLWLLSELIFAIITVSENFIQATMTLSYAGFVLVGSIKMYFMWRKKTEMTQFLKLMDEIFPRTAEQQKMMNLRRHLRQSTIVMSGFALIFMILIWTYNLYPFMQRQIYDCWLDTRSINKTLPYESYIPWNWHNHWSFYLYYVLQSIAGYHSAAGQIASDLVLCAMATQMIMHYEYVSHKIRSRYRGERKCVDSKSVSCLNALNHWTEEQVATHKDMRWLCETIAYHSNLLSLSDVMNDVLGVPLLVNFMTSSFVICFVGFQMTMDAEPDYMVKLFLFLFSSLAQIYLICHYGQLLIDASINVAAAVYDQDWFDLNVRYQHMLVLVVARAQKPAMLKATNFVRISRGTLTDIMQISYKFFTLIRTMYSD

>CcapOR7a-like

MHRVMEYIFGRRRLVVKSGTNDSFELLFLIWKIIGVEHSRSYGFFQLFHVFCCWALLLYSPAAYNMGFLRALKTLPMASALNILQTDINVSILLFKVVIIKFHLKRLRSLRDIFKRLDERYHNPEERAQIDESVAICRRIIYIYIFVYFTFAFLSWITAIMAGELIYSLWLPFVELIPHQGWQYWARFSVEAFYLYFLILVCLICDVYPAVYIRAIRTHVHLLAGRISRLGSNPDLSAEENHQELVDCILSHQELMRVVEVVSAVTSLTLFLQFTVAAMILCVCMLNALIFADRAGQIMTVGYYMGVLLQTGGACFQASMLEAECVKLPLAIFHCQWLNLDRHSRSLLTFFMQRAQVNVCFTAIKLFQINLRTNLSLAKFSFTLYTFMNEMGFGGDTNEKIS

>CcapOR85b-like

MAPYFHTREPAATIPDFVGIPFFLISLNGMQLFKWTPNEEASRRKLLLITAFSVIVTYDCVSMLSVFAFVKLERLDYTTFALYWGYALNSLMKGGTLWFGRRQLEFILKSMVEKHPKTIAERQEYHLVAYFTKIKSFNKYLTIFHLCTTSLFNIQPMVSSIVEYMGRQDKEEEFKYKLPFIMYYYYNERQPVLYLFSYFLQCMGGFYMSYLFLGGDLLLMTLVHLVNMHFEYLIRRIESLQPTEDSEKDLNLLGPLVTYHLEILDYVKKIDATFSLSILLNYIASCLCLCLLGLQIVMGSDLVTVVKFFAFLVSTMVHVYYISHFGNNLIDLSTGISDAFYNHPWTNAKYKYSRMLVLPIARAQRYAHLTAFQFFEISMHSFKSLCTTSYQFFTLIRTSLEEDFH

>CcapOR7a-like1

MFDLLKGRGYRELNSRDALIYLFNMLSFVGLNPTAHCRLLYYFYGSIITLFVVVLSPLIFNIGWIRDRNVLSIMEILNCVQAALNVIGVPIKSIALMLCLDRIHSVEPLLLKLDAHYSKWDDMLRIRQCAIMGNRLVFSYIVPYMMYETLTVVSAVLGGHAPLTLWLPYVDWHRSSREYWLQVCFDAITLFYLLCHQIINDSYPAVYIYIVRTHVQLLERRVSRLGYVPQKSEHENCQELQECIVTHQEILRLVHTIQPIISITMFVQFIIAAAIMSITMINIFIFADLATRLASFVYLICVVLQTAPSCHQASYLQGDCEKLSSSIFHCNWIAQDKQFKKLLIYFLQRSQADMPLIALKMLPINLATNVSIAKFSFSLYTFIQKMGLGAHLND

>CcapOR2a-like

MDKLEALSSRIFPSDPSIGKIGSIEYNVWLAQLFGVPVLGLKKETPRMRIALAVYGVVATLVVTFLYTGFEIYDMIFCWPNLDKLTQNICLSLTHVAGALKVINIIYRLKEVAGVVRKIEYAARYYVISKNQLKAFYRGEFENKIPLTIYASLVGFTGILGIAYLLHNPTGVAGEIFPYRVKLPHWMPFGLQLAYMGFSVLVFALQIVAIDYLNVTMINQIRFQLKILNLAFEELKFVSGQAAHELSLDRRLRTIVDHHNLLRNLRNEVEEIFRLPVLVQFFTSLIIFAMTGFQAIVKSENSNGASLIYCYCGCIFCELFVYCWFGNEVSEQSKTLTTSGYNCHWYQFGPRYKKSLLIFMFNSQKPIVFTAGGFMALSLPSFTGILSKSYTVIALLRQFYGR

>CcapOR67c-like

MPFAFQQLCFELQLSLKYSVPAMPLKLANNEPAATIQDFVGIPLFLLTFMGVKLFKWTPEEASSKRQLIMLGVFCVFATYNFATMILYIMYEPLNSSLDITEIILFWGFSLNGMMKLAIMILYRNELKSILRGLGARHPQTAEERSIYRLVPYYNKILIYNKYLAAWHLSITTLFSFHPLVASILGYIFRRDSSDGYDFTLPFMMWYYYDTTKPILYIFSYVVQTFGAFWMSLLFLSGDLLLISLVHLVNMHFDYLIRHIESFQPNGTDEDMKVLGPLLAYHQEILDYAERIDSTFSLGTLLNYAGSCLVLCLIGLQIVLGSEFLKVVKFIAFLVSTIVQVFFVSYFGNNLMDLSIGMSDAFYNHPWYDGNYRYSRMLVLPIARAQRYAHLTAFKFFEISMDSFKSLCTTSYQFYTLLRTSLEEEAG

>CcapOR94a-like

MDLQEYDNNSGGRRIIQVMKLLGLWYYEGSAKMPYLLYSCLLHFTISIPFTIFMAMDVVHATDLEKFTNIMYLTLTELGMVAKLFNVWFYAKLLVDFFETLSGDKYFELREKDERLKWQHAQRTYARIVLFYVFIGLGAMFTGFVGVLFSAKYELPFPYAPPFNWHTPHGYWCAYLYELLAMLITFFANYGFDMIQCYMLLQLSLCFKLICGRLECMGELRSGTAVSRGFSEQQLYRQFVDIVKLHARIKNLSRLCQTYISFPFLIQIMCSSFVLCFSAYRLQKLSILSDPMQFLTFVQVNLIMILEIFLPCYYGNEVIAQSSALNNATYNSEWFRCSPGLRKYLVIYMAMLQRPLRVRAADFFDISLEIFTNTMKNTYSLMALLLNMNN

>CcapOR33b-like

MLKNVFIFKPAGNAAVDSVACFDIFWMCWKLNGIAVNSNKWYITLYDIAVNIFITIFYPIHLTVGLFMVPTLADVFKNLAINITDVACSTKHYLFRYKLPKIRELQRLLKQLDERVLAPNEREYFDKKIRLGVRNIMLLFCASYAADALASAIDVLSKNERELMYPAWFPFDWSANRFTYYGAVFYQIFGVSLQIVQNLAHDTFAPVGLCVISGQVRLLAMRVSKVGYDESKSLAQNEQELNECIEDHKKLLRIFDLMQDVFWYTQLVQFSSVGLNICLTVVFLLLFVDNLFGYVYYTVYFISMAIELLPACYYGSNMQEEFQNLPYAIFKCNWIPQRRGFQQNLRIFTELSHKQLTPTAGGIINIHLTSFVATCKMAYSLYTMLMNI

>CcapOR33b-like1

MFQRQIETRAIFRRLFMTWRVLGIILWPFNKYLRIIYDILMNIFITFAFPVHLTLGVIFSSNQEQFFTNLIIGIASVSCTFKHLLWRSRLAEMQQINEILAQLDDRVRVREDYEYYKRSIERLCNFMINFFTRCYFSVGVTALFIALITGELLYPAFMPLQWRTSFWNYVAAILFQFVGVMLQIVQNIANDVYGPVVLCMISGHVHLLANRVSRVGHDTEENTQSNYEELSKCIEDHKLLMSTSKTVERIASLSYLVQFVAVGINLCIGLVYLLFFADNYFAYVYYTIHITAIMIELFPCCYFGSMLECEFHDLSYAIFSSNWPTQPRPFRRNVVSFTEMTLREVTMYAGGMIRINLDSFFATCKMGYSFFTVIQTMK

>CcapOR67d-like

MAAEKVSPSESFAKIVKIFRLICSLVGADVCDVNYRINIVTAIVIFCIIIYFIFTATTVASVFAENWEYMLEASCMVGSVLQGITKLTSGIAFAKEICAMRFELEDLYRLYETRGEEYTAVLHLSCKRVWQVIKMVGQIYLAAGVGILFMTAIFIVATDEKVYIMHFFIPGLDVHTQMGYLLTMAVHTVVFLAGAFGLFAGDLFFLLFLGQPMLFLDLLTLKVQALNVAADRCSNEAERLLIDIIEWHQYYTDYNRRCNHLFYYIITMQIVTSGISIICTLYILLLGDWPGAYLYIFVAFSGLYLYCIMGTKIQICNDAFCEELYNIDWYKLNVKSQKMLVFILKKSQKPAEIKVGGFLPLSVQTALSITKTIYGIFTMMLRFLDEEN

>CcapOR85c-like

MSIIIRFEEFLRLPSFFSRNIGIILWGQRGKLFDRFMFYFSSINLFLTLLAELWYIISTISTDFITAIMGLSYVSFVVLAEVKFYYLIKYDMKVSTVLKRLNALFPHTKEEQENIQLIKYLKMSKFYTLFYTVTFMLVIWTYNLYTVSQRFIYTKILQVREIERELPYPAIYFWNWQDNWSYFMLYISQSLAGWHATCAQILTDLLICILISHLIMHYDHIARSLLNYQSKFAELYGKESTMKCMPKLARVMMEERAVRADMKFLADIIAYHTELLSLTESLNDVFGVPLFMKFMSSSAIICFLGFQMTVNRGFDLLTKLALFFILSVLQVYLICHFGQLLIDASTNVSTALYSQDWTNADVRYQKMLVLIIKRAQRSATLKATNFIIISRATMTEIMQMSYKFFALIRTMYND

>CcapOR10a

MKFKFLSRTFPLRDYYFYVPQLCLGSMGFWPMDTCRQQAANVGAWMNLIILAIGVFTEIHAGCTVLRTDLELALDTLCPAGTSAVTLLKMTLIYYYRQDLAWVLERMRSLVYERDVSINPIKKRIIRAHAVMAARLNFIPFVMGFITCTSYNLKPLLITLILYMQGQQPMWKLPFNMTMPAFLLRAPYFPFTYIFTAYTGYITIFMYGGCDAFYFEFCSNAAALLKLLQEDLKSIVSFEEQLVFTAQESTLLEWRLVRFIMRHNDIIELTRFFCKRYTIITLAHFVSAGLVIGASIFDLMTFTGFGIVIYIAYTIAVLGQLFIYCYGGSLVAESSVQLATVAFGCDWYACNPKLRRYVLMIIIRSQRAISMSVPFFSPSLITFTSILQTSGSIIALASSFK

>CcapOR43b-like

MRKIGDLCYGRGKNNVYIKESFRLLFFSWSLTGIAPTKTPRLFNTIFMIICWCGILMCPYCFIAGAVNSMKTSVITVTLVNLQAALNGIALPLKAITIAVNVKRLRSIDNIFKELDNSYTDPVHHELIKKSVMRCTRLFVVFLTVYWLYGITSCTAALFSHKYPHSMQIPFIDWLPDSDVKYWLHYILEASYFFFLLLVNLTNDVFPAIYIKAIRTHLYLLTERVSTIGKKSETTAEQNYDTLVECIISHQKLLRISDTVGDVISKSIFFQLAVYSTILCICMLNMLIFADTTYILVTLVYLIPVLSQTIPSCYQASMLEAESTKLSVAIFHTNWWNLDKRCHKLLIYFIQRSQQEMVFTAVKLFQISLKTNLTIAKFSFTLYTFINKMGIGETWKN

>CcapOR59b-like

MSNLLQRLLNQLLPSRTTQKSIDVVKQVSPSLSNAELIRIQFERATRTPKERSAPVGRPYQAVHDIHSRDGLIYLYRSFSALGVLMPDKHKILYCLYALLPLGLITFYLPISFALSYFYLDYSTVKIGNLLTSVQVFIASIVGGVKLIVMAFKLPKLRASEAIMHQLDARCKDEDEIEVLRKVVRQGNRVFVLVLICNLIYSTSTFLAAASKGRPPYNLYNPVVDWRKSKGAFLWAALWEFILMDGLCTEEAITDSYAPIFVCIMRAHMKTLLMRIQKLGSNPERTLDENYEDLKMCIKDHKLLLELFDVVHPIISTTYFLQFMTTSLMVGCTLLNIMIFAVDNLARVGHLAYVMALLMEVYPLCYYGQSLLDDSNRLANTIFHANWIKQNEKFRKMLVVFTQHTQKPMELLAGKT

>CcapOR42a-like

MNRFRKQSARAAETENNKLALALSKEKLSGLKNGEEQRIARKPAKPASAVREKQGEFGNVFFSLTFLVASPASSKEATNYFFKAAFGMGIMLPTRHRILYILYSFAVNSMATLYFPIGFTLIFFTLPEDDLDVSNLLTSLQVTFDVYAGSIKLIIMAFLLGKLRTSEIVFQQLDNRCRTPDEMNELRKMQQFGRKVIIFYMTIFLIYSSSTFLGSVTFGYPPYSLYFPFLKWRRSRIEFIIASLLEFLIMDLACLQQTVNDGCPVVYVNILRTHMKILRSRVEKLCTNAALTKEQNLLELKLCIKDHQLLLELYEIIASIVSITLFLQFTVSAICVGTTLINFVIFANGFSTRVACFCFILAVLIEIYPICYYSQCLITESEGLSDVIFHSNWIEQNKEYRQLLIFFIQNAQRPMSLTAGKLYPVTLSNFISIAKFSFSLYTFIEKMNLKERLGIE

>CcapOR49a

MDFEKIFWLPNTLYLVVGYDFRQVSKSYLKKILMTAFLILTNITGICIRIYMLIQLRELVLSGDMLNSFRLGVYISYAFDSIVKFFGFLHNAHRLRKIYESLATEFPQTFSEQQFYQVHKYSFNRSRILIFAYLSVTNSILLGPIVQSIIMYIIDAFLYGLSGAKFQCLHPTPITYNFNFCSPRYYIPIYIVEYLNGHFLTTTSLGTDLYVCTFAAQVCMHLKYLGNSLEGYEPSADNSKADCAYLKEWIKKHQLMLRLCADINDVFGTTLLFKLISNCTVFCIIVVQLKLEGFGWGFLNFLCFFFVTVAQFFMVCHFGQKLINTSEDVSLCAYKNRWYNGSKAYKTLLFTIIARSQKSCKLTAKGFQPISLQTFQIVMTMTYRAFAVLQRALD

>CcapOR24a

MSIKFLTQSYPTEKSLFLIPKFVLRIVGFYPEQEKSTIRRNAWTMFNLIMLIYGSYAEFMYGVHYLSIDAVRALDALCPVASSIMSVVKLSFLWWHRVELERLIRRVSVLTAEQDSRLKNNYKRRYFTIATRFSAALLCLGTCTSTLYTIRAALANYFSYVRGENVPYETPFKMIFPQTLLSKWIFPVTFTFSHWHGYITVAGFTGTDGLFLCFCMYFGTLLKALQIDLKDLLKDMDCGQHEGLSERDIEECMKKTVMRHNEIIDLIGDFSAVMSSITLTQFVLSSVIIGTSVVDMLLFSDYGILLYFVHALAVTTELFLYGIGGTTVIECSSQLATAVYDSNWYSHNVEVKKMVLFMILRTQRSLVIKVPFFAPSLPALTSILRFTGSLIALVKSVV

>CcapOR22c

MLQPLLGSQVPIEQSFFRIPRISARIAGFWPQPAVRPRTWLTVLRFCVNTFAVAVGGFGEVTYGFFYLYDLFSALEAFCPGVTKVISLLKMTIFFGRHERWQRVIHGLHTLLLLDTSAGKRRIMEPLASFASVLSFVLLASGSLTNTFFNVLPLLKMAYFKWRALDMQLLLPFNVILPEVLVNLPYYPATYLVLTLSGAMTVFTFSAVDGFFLCACVYATALFRILQHDIRNAFAELQEQESSSFEQNMRIQHRLSVLVERHNKIIDLCSDFAAEFSLIILMHFLSAALVLCFSILDLMLNSASIGVLIYIFYSIAALTQLVLYCIGGTYVSESSLSIAEVIYDVDWYKCDVRTRRMLLLMMCRAQRAKTIAVPFFTPSLPAFRSIVSTAGSYITLLKTFI

>BcorOR67d.4

MTIKHIRPTASFAKLVKTVRFISSLVGADVSTENYQVNIITIIVIICIIMYFIFTATTVASVFAENWTYLLEASCMVGSVLQGITKLISGISRTNEVSGMRLELEELYRVYESKGESYCKIMNACCDRVWQIIKMVGLIYGAAIVGNLLLTSFMVFFTNQKIYIMHFFIPGVDVETSFGYLVTTALHSVCFLAGAFGLFGGDLFFLIYLGQPELFRDILILKVHELNEAAAQKDNKTESLLISIIEWHQYYTDYNERCNELFYYIITMQILTSGVSIVFTMYIILMGDWPGAYLYILIALSGLYLYCIIGTNIQTCNETFFEELYNINWYELDVKGRKMMILVLMKSQNPSEIKIGGVLPLSVQTALQITKTIYGIFTMMLGFLDEEE

>BcorOR67d.2

MKTQKQPSDMYYKLLSVIRFCSRTIGVDITAEDYKINSNTYIVIGAIIAYYFCAINMIAKYILTDLTVLLDVFSPVSCTTQGAVKLMSALLYPKLYRKLATDIGQIYEKYQQMGREYQQKLLEWNKNMKKILFACAIVYSITAMLILSTPIVMYILKGERHLILLCEVPGFEADSYYGYWVNNAFNLLCVMIAAFGLYAGDLYLLLFLTH

SIFFYDILVLKINDLHKLLEDEDKEDRQTKIVNDIVEWHQYYLDFNDTCNLLFFWTISAHIICTTTGILSTLLIIMLKDWPGAYTYLFVCFLWLYMYCILGTRVEICNDQFCTGVYDINWYALDVRNQNTIRLMLMQSQAPRNITIAGVEPLSVSTALKITRTIYSLVMMVLRLQNK

>BcorOR67d.1

MQTKTRPSDNFCKLLKIIRLSSSLVGIDVIDENFKFNYVVAFVLVAIAWNFTISIYTIWKDVKTDWTVLLDVFSPISCATQGVVKIISILLYPKLYRELATDLVNIYNKYQALGTKYETKLFEWNQSMKNILIIGGLVYFVSAVLALVTPLFLYIFKGERHLIIMCQMPYVDLDTDHGYFITIGYNILCVFVAAFGLYGADLYVFLFLTHSIFFYDIFALKIEDLHEVLHENNQDTRIKAMVNDIAGWHQYYLDFNDKCNQIFFWTITSHILCTILGILTTLLIIMLKYWPGAYPYIFVCFVWLYMYSILGTRVEICNDQFCDGIYDINWYDLDVSDQKTVSLMLMESQVPRIITIAGIEPLSVNTALKITRSIYSLAMMVVQFNE

>BcorOR67c.1

MMPSFKGSEAAPTVPDFVDIPLFQIKFMGAKLFKWTPDEPRSKLQITLLGTFCVFATFNFTSMLLFVISDELETSLDITEFILFWGFALNAMMKGGTMVCFRRDIEFVLKGLIARHPKTEEEREAFQLVPYFRTINASNKYLSIWHLSITLIFALHPMVSSLLRYIWRGDTNESYDFTFPFMMAYYYDTNQPLPYAVSYFIQCCGAFYMSLLFLSGDLLLISMVQLVNMHFGYLIYKIESFQPTGTDADMRMLGPLLEYHNEILDYAERIDSTFSLATFLNYVGSCLVLCLIGLQIVLGSDALSVIKFVGFLVSTIVQVFFVSYFGNNLKDLSTGISDAFYNHPWYDANYKYTRMLVLPIARSQRYAHLTAFKFFEISMDSFKSLCTTSYQFYTLLRTRVEDEGV

>BcorOR49b

MFDDLQLIHMSVRILRFWSLIYEHTWRRYVCLSMTTFLVFTQLYYMFRTSEGIDSIIRNSYMLVLWFNTILRAYLLLYDREKYEKLLSDLETFYYDLKRSKDAYIQDLLVEVNTTGKYMARGNLFLGLLTCFGFGLYPLFATERVLPFGSMIPGVEEYKSPFYEFWYIYQMVITPMGCCMYIPYTSLIVAFIMFGIVMCKALQFRLKTLHRVRHIESLIHKNVRECIRYQLSIIDYITRVNALTTYIFLLEFLAFGTLLCALLFLLIIVDSSAQAIIVCAYIAMIFAQILSLYWYANELREQVIAIAAAAYDTEWFTFPIPVQKYILLMILRAQKPPAIMVGNTQPISLELFQSLLNASYTYFTLLKRVYT

>BcorOR49a

MDFVQFFWFPNALYRIVGYDFQQLPRAHWRQALMKAFLIFTTISGICTRIYMLFQLRELILSGDILNSFRLGVYISYAIDSNVKFFVFLLNAKRLRVIYQSLSNEYPMTSMEQKLYQVDKYSFKRARIMIVSYLSVTNSILIGPMLQSIFMYIIDLFRYGYAAAAFLYLHPTPMSYNFNYCTPHYYILIYISEYLNGHFCTTTNLGTDLYVCTFAGQFCMQLEYLGSSLEAYEPSLDNSKADCEFLMKWIRKHQLMLDLCSELNEVFGTTLLFKLISNCAVFCIIVVQLKLEGFGFGFLNFLSFFLVTVAQFFMVCQYGQKLITISENLALCAYKNRWYNGSQTYKTLLFNIIARAQKPARLTAKGFQPISLATFQIVMTMTYRVFAVLQRALD

>BcorOR47b

MISLSSKATISNTIASHNSYLTNHSYTHLKHTASKLQTILAPYRVLKEMLRCGEAVQPPHTCLFYFRFYIRLLGLWPAKRAAENQLYYFYNLFIMVLFSFFMATIICDLYEASSDFVLLGEDLVVVLGLSLIFFKMILFRMGNVDTDIIINEFDALHIKHARGLSEDSRNRRILQWQRNFFFGEMCLFSGFYILSLLLFAAMSLQPLLSQQTLPFRCKFPFGLNDPDEHPIAFVCVYFFQYFCTLYMLVAIVVMDSLGGNSFNQTTLNLRILCENIRHLGIVAAGASSSTSEAVAWRELREAVEFHQKIIGLMNRINQTFYWNYVSQMGASTFMICLTAFEALLAQDKPMVAMKFQTYMFSAFMQLLYWCWMGNRTYYDSMEVATAAYEIRAWYEHSPRLQRQLMFIIKRAQKPLEFRAKPLFGFTFASFTSILSTSYSYFALLRTMSD

>BcorOR43a.2

MQFCSELFNWSLAFIRRIGYFDQHRLAWFYLMSPIFLCFMAYYRTYIIRNDLDEVFVNLFKLSGVTTTTIRAFIVMYKAEKFLNFFESVDKWYLELQREGDDVTFKKAHEFTQKIKKTSKTVLILSGITMLYISFIQLLATVGVGYKRLLIDVAFPGVDLYESPLWEIMSILQSLWIGPLIFFSYVSYLCLTVIAIAFGIFLMKNLQSKLEGMNEMTDEEALKCIKKCVKDHVTIIKYHRDLEVLFSVGSFADVCIFAIIPCVIIVISTMDHDMSLLIADIQLSFMVMISTFIIFWMANNFCCESANIAKSAYNCSWENRNKEFRKYIPLIIIASQKPLQLTAGGLKPINMEFFLTMVRCTYSLFTVLFTMKTEGDS

>BcorOR43a.1

MVNAVVDNPMLSVNVKLWQFLSVLFARDWRRCAVFVAPVCLLNAMQFVYLYQQWGDLATFILNTFFAVSVFNALLRTCLIIKNRDKFEALMEELVTLYDDIQDSGDDYAKSVLAAATKSARNISIFNLSASFSDLVVAMAYPLFQQQRVHPFGVALPGIDVTRSPLYELIYIGQLSFPFTLSSMYMPYVSSFATFSMFGKAALQILQNNLRNLCDDMKSKTEEELFETLRKNIAYHARIARYVSDFNELVTYMVLIEFLLFSCVICSLLFYINITTSTAEKISIVMYIGTMLYVLFTYYWQANGVLEMSLLVSDAAYEMQWYNCSPHFKRTLLIFIARTQNPLQIRVGQMHPMTMEVFQSLLNNAYSYFTLLHNLYND

>BcorOR42a

MRQSPRQATADLPKIGLALALHNDIATQRDIKIKASSSAKALSENIEEEAAEHHVSSQDTTNYLFKSAFGMGLVMPSRYRALYVFYGFLVNFFTTFYFPIGFTLILFTLPDDVNISNLLTSLQVTFDVYGGSAKIIIMKFVLEKLRATQILTQRLDKRCRASDEVEELRQMVRFGKKVVIFYLTIFLCYSASTFLASISSGYPPYSLYFPFLKWRRSRTEFIIASLLEFIIMDFACLQQTVNDGYPVIYINMLRCHMKILQFRVEKLGTNPTLTQVEQLSELKLCIKDHQLLIELYDTIAPIISITLFIQFALSAVCIGTALINIVIFANEFQTQVACSFFILAVLIEIYPACYFSQCLINESDKLADVIFHSNWIEQSPEYRKLIIFFLQRSQRPMFLTAGKLFPVTLSSFVSIAKFSFSLYTFIEKMNLKERFGIE

>BcorOR19a

MTLKINSWDAFKYHWRVWDLSGFRGPQRQSVWYIPYKLYTIAITLLFSIYYPICFTVESFLADNLNDFCEVIYIAMADMTLNIKFLTLFIVRRQLLELRPILKRLDARAKTEEEMNVLQEGIDSAKKCFLIILRLFYSAFVTSQLMVIFSAEARLMYPAWYPFDYQASRTKFWIAYGYQTIGFLVQCTQAVSVDTYPQAYMRVLTAHIRALSLRIERIGRKNFSGASSELMCSKEDEMKRNYDELVSCIKDHKKIIELFSIIQKTISGTSMAQFVCTGAAQCTIGVYMLYVGFNISIMLNMAIFFVSVTMETLILCYCGDLFCQECEELSKAIYNCNWTVQSSEFKKVLCFFLFRSQRVNVLRAGNWIPVRLPTFVVVVKSSYSIFTLLSSFK

>BcorOR10a

MNFRFLSRTFPLRDYYFYVPKLCLGALGFWPLDTSVLNASNVWAWVNLIILTIGVFTEIHAGCTVLKTDLELALDTLCPAGTSAVTLLKMALIYYYRKDLAWVLKRMHDLVYERDVSINTVKKHIVRAHAVMAARLNFIPFVMGFITCTSYNLKPLLMTLILYMQGQEPMWKLPFNMTMPSFLLHAPYFPLTYIFTAYTGYITIFMYGGCDAFYFEFCSNTAALLELLQKDLESIIKFDQLILTMEESTVLEWRLVQFIKRHNDIIELTRFFCKRYTVITLAHFVSAGLVIGASIFDLMTFTGFGIVIYIGYTIAVLGQLFIYCYGGSMVAESSVQLATVAFGCDWHACNPRLRRYVLMIIMRSQRAISMSVPFFAPSLITFTSILQTSGSIIALASSFK

>BcorOR7a.8

MFELLTGRGIRNVASKDALIYFFKGCTIVGISPPKNAGPLYYMWSFLVNAACIITGPITATVGFVMKYMQNIITTVQFLNGLQASLNLIGLPVKCLTVTSALRRLRGMEPTLAALDARYTRPEDVALIRKAALMGNRMVFFFGTSYLMYMLFTVIPPLISGKAPLSVWIPFYDEHQSTMHFFGQIVYDLFLMGFVLFHQVLYDSYGSVYIYVISTHIQLLVRRVGRLGTDATQSKDDNLNELVDCVVTHQQILELLATIEPIISKTIFTQFLIISSILCVTMVNMFFFADRSTQIASTLYFLCVLLQTSPCCYFATELKADSEKLPLAIFHCNWPEQDRRFRKVILYFMHHAQLSIELMAMQLFPINVATNISLAKFSFTLFTFIKEMGIGQEAKK

>BcorOR7a.7

MFDLIKGRGRTVFASRDAVIYLFNSFRFLGFNPPAKYRLPYFMYSAIITFFSVLFSPVIFNVGWLRDRNKLSVMEILTCVQASLNVMAVPLKCITLAMAHKHLRGIEPMVTELDERFPTPEDKVKIKQCAVTGNRLVFGFAVSYFMYETLTVVSALVGGHAPLSLWIPNVDWHRSTWEYWLQVSFDTAVLFFLLYHQVLNDSYPAVYIYIIRTQVQLLTSRVEKLGYDEQKSVDENYQELLECIVIHQKILKIVKIVESVVSITVFTQFLVAAAILGVTMINIFIFADLTTKIASVTYFFCVLLQTSPTCYHASYLLDDCDQLRIAIFQCNWIAQNKRFNNLLIYFLHRSQDSMPFFALKLVPINLATNLSIAKFSFTLFTFIQEMGLGENLKG

>BcorOR7a.6

MSKILRVRGATVYKSRDALTYIFNIFTFVGTNPLENRSQRYYSLYYFYSFTVNFICCLFCPLSFHIGYIKLRHVLTNSQLLAAIQNAVQVSGIPIKILIITWYMKRLRHAFEILDELDVNYTRREDLAKIRECVRRCRKIVLIFCFPYYSFELSTIALGVVQNRAPLAAWVPFLDGQRAAWEYWTIVLWDAFVMFILLCHQLGSDTYPPIFINIIRTHIQLLITRVNRLGRAGALTADEHYEELLGCIRSHVQIVSIAKIVAPVISVTLFTQFATTATTLLNWLGNVEYPENIISLAFFSCQLLQILPCCSSASQLIADCERLPDAIFHCNWVDQDRRFRRAILFFLQRTQNLIRFSCLKLFDVKLETSVAIGKFAFSLYTLLEETKVGTDTEN

>BcorOR7a.5

MRKIADLFYGRGKDDFETTDSFALLSRSFAAIGFLPKLPKRTVDVIHQLICWSCVLSCPYLFLSGVVKTMHSLPITIVLAHLGVAINTIVFPLKAIYIKANIDRLDDIGKIFKALDKRYQRPQDQMQIRDSVKTCTRIFLVFCMVYWLFGTSSWLVALRTHEYPNGNNLPFIDWLPESNLRFWLHFIFEIVFLHELIQMSLTMDSFPALYIRALRTHINLLTDRVSRLGLNPDFSDQENFEELVDCIVSHQELLQISDTVGKILSLTTFFQFTIYAVILCVCMLNMFVFGEASTKLVTLVYLLPVFWQTIPTCYQASMLEADSAKLPLAIFHCNWLALDKRCHKLIIYFMQRAQEEISFTAIQLFVINLRTNLSIAKFSFTLYTFINEMGFGETLKDRLE

>BcorOR7a.4

MRRLTDLLYGRGATKFETNDGFQLLFQCWSLVGIKPLKLYRLRGMLHMCFCWFLLLLCPFTFFMGYLHTLETEPLTVQLNILQAICNIIGLPLKAIAITILLTHLRSAEPIFARLDARYESVASREQIKNCVVVSTRLLASVGLMFHFYGITTYLQALLTRGYPMGEWLPFTDYILRPTIRYWAHFIFEVFHVAFLLTVQTSMDVFPAVYIRNLRTHLKLLTERVSHLGENPEFTDEENFDELVDCIVTHQELLEAKNILSSVCSITLFVQFVIAAIALCIALLNFFVFADTVQRVVTLLYYLGLIMQITPTCYQASMMEVDSAKLPDAIFHCNWLAMDKRSRKLIIYFIHRAQEKISFVALKLFNINLTTNLSIIKFGFSLYTFMNNVGFGQNLKELLE

>BcorOR7a.3

MQRFSDFIYGRVKSDCETNKPFKVLLAFYGLIGLKAKPHGFLPTLHMVFFCVAYAYAPFLAIVGFLRYQKTATVTESLSALQAFINAIFAAAKSIAVLVNFKRFQSVEPIMKSLDERYKTPQERRKITDCVADCTRLYAAMGFIYYLYGLINILTALIIHKQPFGGWYPFLDWINNPTVHFYSCFSCETWYLYFLLTAQYLHDVYPTLYMRTIRAHMQLLRERIGRIGMDPEKSVDENNKELIDCIATHQQILQVVDMVRSVCSPTIFMQFVCVALVHCVCMVNIFIFADTLNRVITMWYYLMVATQILPTCYEASTLEMESSKLPVAIFHCNWLALDKRGRKLILFFIHHAQEEVTFVAMHLFEINMRTYLSIAKFSFTLYTFANEMRFGQNMKELVE

>BcorOR7a.2

MRSFRDLYYGRGGEEFETNESFQMISSTWAILGIKSLKPYGFIRTLHVAFGYIGLVMCPVVFVVGFIQVTKQTSSMTVILTALQVSLNSLGLPVKAFVRVIYLDRLRSVEPIFKGLDERYQNPQGRFAIRDNVIQSAHLFVTLLALYLAHCTVSLPTSLYMHTQPLNIWLPLVDWIPHPTIQFWSHFVIEVVYVYFLLMAQCMNDIYPAVYIKAIRTHISLLVDRVSRLGENPELTDEDNYHELIDCVRSHQELLQISSAVGSVVSITLFIQFLVAATVLCVCMLNLFLFADASHRAQTIVYYLCVLMQTSLPCFYASMLEMDCAKLPDAIFHCNWWKMNKRSRSVLIYFLHRAQKEISFVALKFFQINVGTFLSIAKFSFSLYTFMNQMGLGQNIKNQLE

>BcorOrco

MQPSKYVGLVADLMPNIRLMKYSGLFMHNFTGGSGLFKKIYSSIHLVLVLVQFLLILVNLALNAEEVNELSGNTITVLFFTHSITKFIYLAVSQKNFYRTLNIWNQVNSHPLFAESDARYHAIALAKMRKLFTLVMLTTVASAVAWTTITFFGESVKFAFEKETNSTITVEIPRLPIKSFYPWNAGAGMFYIISFAFQCYYLLFSMVHANLCDVLFCSWLIFACEQLQHLKGIMKPLMELSASLDTYRPNSAALFRSLSANSKSELINNEEKEPTDLDISGVYSSKADWGAQFRAPSTLQTFNGMNGANPNGLTRKQEMMVRSAIKYWVERHKHVVRLVAAIGDTYGGALLLHMLTSTIMLTLLAYQATKITGVNVYAFTTIGYLGYALAQVFHFCIFGNRLIEESSSVMEAAYSCHWYDGSEEAKTFVQIVCQQCQKAMSISGAKFFTVSLDLFASVLGAVVTYFMVLVQLK

>BminOR24

MSKILRVSRAKIYKSRDAVSYLFNIFTFLGTNPLEHRSRRYYFLYYFYSFTLNFISLLYCPLSFHIGYIKLTHVLTNSQLLTAIQNAIQVSGIPIKVVAITWYMKRLQHAVEILDELDVNYTQREDLAKIRECVRRCRKVILLFCLPYYSFGISTIALGVLQNQAPLTVWVPFLDGKRAAWEYWTIVLWDTLVMFILLCHQLGNDTYPPIFIKVISTHMQLLVTRVNRLGRPGALTADKHYEELLACIRSHVHIVSIAKIVAPVISVTLFTQFATTATTLLNWFGDMEYPENIISLAFFCCQLVQILPCCLSASQLIADCERLPDAIFHCNWMDQDRRFRRAILFFLQRTQTPIRFSCLKLFDANLETSVAIGKFAFSLYTLIHEAEDGGKTDN

>BminOrco

MQPSKYVGLVADLMPNIRLMKYSGLFMHNFTGGSGLFKKIYSSIHLVLVLVQFLLILVNLAMNAEEVNELSGNTITVLFFTHCITKFVYLAVSQKNFYRTLNIWNQANSHPLFAESDARYHAIALAKMRKLFTLVMLTTVASAVAWTTITFFGESVKFALDKETNSTITVEIPRLPIKSFYPWNAGAGMFYMISFVFQCYYLLFSMVHSNLCDVLFCSWLIFACEQLQHLKGIMKPLMELSASLDTYRPNSAALFRSLSANSKSELINNEEKEHTDLDISGVYSSKADWGAQFRAPSTLQTFNGMNGMNGTNPNGLTRKQEMMVRSAIKYWVERHKHVVRLVAAIGDTYGGALLLHMLTSTIMLTLLAYQATKITGVNVYAFTTVGYLGYALAQVFHFCIFGNRLIEESSSVMEAAYSCHWYDGSEEAKTFVQIVCQQCQKAMSISGAKFFTVSLDLFASVLGAVVTYFMVLVQLK

>BminOR9

MVHREHDNISGGRGVITVLKLLGLWHYEGAMRIPYILFSGLLHSICTIPYTIMMCLDVLQATDLKKFTNTMYMTLTELCLVVKLVNVWCYSRLLVDFFAAFEHDKLYQLQDVEERLNWRRPQRNFARVVFIYITVSFSAMVSAFIGVLYREDYELPYPYAPPFDWRTPRGYWYAYFYELLAMPITGLSNCAFDMIQSYMLLQLSLCFKLISARLACMGALQEDGASSGFCEVKFHREFVDIVNLIMTLQIFLPCYCGNEIIQHSGSLNNAIYSTEWFRCSPRMRKYLIIYMEMLQRPVCVRAGNFFEISLDTVAIYPACVRAGTEVRNVQRVACATNHGQTIKATMTYELPGFPCIFAQPLLTLDHLMTVLARDRVGPLRLHTGANVLHSRHGIINALRKVIN

>BminOR8-2-1

MRNVLNLFYRRGKDDFETNESFVLLFRSWSSVGFIPKKPKRIADIIHQLICWTCVIICPYIYFSGVIETMSYLPITIVLANLGAAINCIAFPLKAFYIKANIDRLHDVGTIFKDLDGRYQRPQDQMQIRDLVTNSRRIFAVSFILCWFYATLSGLVALFAHEYPHGNNLPFIDWLPESNFKFWLHFTFEVMFLQYLVQVNLTNDSFPAIYIRAIRTHVSLLTDRVSRLGSNPDLNDQENFEELVDCIVSHQKLLQISDTVGTILSLTTFFQFTIYAAIICVCMLNMFIFGNATTKVVTVVYLIPVFWQTIPTCYQASMLEGDCAKLPLAIFHSNWLALDKRCHKLIIYFMQRTQKEISFTAIKLFQINLRTNLSIAKFSFTLYTFINAMDFGKTR

>BminOR8-1-3

MQKFSDVLYGRVESDCDTNKPFKTLLHLYGLIGIKPKPKGFLPTLHMVIVWMAFGFTPLLSIVGFIRFQKTATITESLTRLQAVINAIFIVVKSLVVLVNLKRLENVEPVMKSLDERYNTTQERQQISDCVAACTRLYASMGCLYYSYGTLSILSALISHKQPFGVWYPFLDLISNPTIYFYTCLLLEACYGYFLLAAQYLHDIYPTLYMRTLRTQIQLLRARISRLGEDPDMSDKENHKELVECIDTHQKILQVVDMVGSVCSPTIFIQFSVVAIVHCICMVNLFIFADTINKVITILYYATVGMQILPTCYEASTLEMESSKLPDSIFHCNWLALDKRGRRLITFFIQRAQVEVSFVAIQMFEINLRTYVAIFEEAFTYGKSFTVH

>BminOR8-1-2

MTVILTTLQATLNVQALPLKATVASIYLNRLRSVESIFKSLDARYQSPQGRFAIKDSVMKSAHLFFIVSVSYFTYGTISWLSSVFTHTQPLNIWLPFVDWIPQPTIRFWMHFIFEVLYVHFLLIIQFTNDVYSVIYLKALRTHITLLAERVSKLGENPEFNDDDNYEELIDCVRSHQELLHLVGSVLSLTIFLQFTVAAVILCVCMLNIFIFADASHQAITIVYYVCVMLQTLPACYQASMLKADSTNLPNAIFHCNWLAFDKRSRRLLIYFLHRAQEEISFLAAKLFEINLGTNLSRKGIIPAKRPYISMGYSQVIIFEMLRSV

>BminOR8-1-1

MFQDQVRLPLQRRITSPENTRNMRKLTDLLYGRGAAKFESNESFQLIFQCWSLFGIKPLKQYRSGRLLHMCFCWFCLILCPFSFYMGYLQTLQTAPVMVQLSLLQATVNVLGLPLKAIVITIFQTHLRSAEPIFVRLDERYQSTESREQIKNCVALSTRLFTIVGFMYHLYGGITYFQALVTNNYPLRTWLPFTDYIPQPTIRYWAHFMFEVFHMAFLLSVQFTMDVFPAIYIRNLRTHLNLLTERVSQLGGNPDFTDEQNYDELVDCIIMPTCYQASMIEEYSTKLPDAIFHCNWLAMDKRCRKLTIYFMHRAQENVTFVALKLFKINLTTNLSVSKSCCKEQQ

>BminOR7-1

MSKILRVSRAKIYKSRDAVSYLFNIFTFLGTNPLEHRSRRYYFLYYFYSFTLNFISLLYCPLSFHIGYIKLTHVLTNSQLLTAIQNAIQVSGIPIKVVAITWYMKRLQHAVEILDELDVNYTQREDLAKIRECVRRCRKVILLFCLPYYSFGISTIALGVLQNQAPLTVWVPFLDGKRAAWEYWTIVLWDTLVMFILLCHQLGNDTYPPIFIKVISTHMQLLVTRVNRLGRPGALTADKHYEELLASKIVAPVISVTLFTQFATTATTLLNWFGDMEYPENIISLAFFCCQLVQILPCCLSASQLIADCERLPDAIFHCNWMDQDRRFRRAILFFLQRTQTPIRFSCLKLFDANLETSVAIGKFAFSLYTLIHEAEDGGKTDN

>BminOR5-2

MPATETTISGVSVFHFHDVTLQYLGLMPPTKKLYRYLYYVYSLVLNIIITVGYPTHLMIGLIKSENKSDVFKNMSINFTCSACSIKIFAFWWRLAEVQKIYAIISKLDKHIVRSTDYELYKIYALRRAQHVLYFILVIGLGAAISSEVATIIGGFLGEWRLMYPAYFPFDIERSLWGYPIAHIYQCFGVTAQIFQNLINDTLPPMALAMLAGHVRLLNVRVARIGHDTSVVSVRKQACNTEFLLCVEDYKALLEFRVAIQRLCSLGTFVQILVTAINMGVVIFYLIFFVNGIFTYIYYVVFLIAMPLEIFPLCYYGTSVQMEFEELTYGIFSCNWMDQNAAFKKNLRIFAEQSLRTQIVIAGGMFAVNLDTFFGTLKGAYSLFTVVVQMK

>BminOR5-1

MSFEEKPPGVKELFRTHWTVWKWLGQVTHPEYPKLHIAYTILLNISFSIGYPLHLLLGQLNLKTMQDVLLNLTISVPVAVCTLKYFNIWRNLAKVRHLEQMFNTLYARIDHPEEWIYYRKVIIPYALKVLHLFYFICVGTAITSELTLLIMGFAYEWRLMYPAYFPFDPYATKAGYVTAHVFQIIGLMVQLAENLVSDTYGGMCLTLLAGHANLLGQRVASIGYDERKTQEENNRELVDCIIDHNVLFDCHRTLGDIIGFGLFVQITSASLIMGVVIIYVIFFVGNAFEFVYYALFLFACIMEVFPTCYYATYFEIEFEKLTYQMFSCNWMDQNREFKRNLIVCVEQSLKTRYFRVGGMFRINLQIFFATCKGAYSVLAVALRLK

>BminOR47b

MISLSSKATIDNSISSHNSYLTNKSYTHLKHTASKLQTILAPYRVLKEMLRYGEAVHPPHTCLFYFRAYIRLLGLWPTERTAENPLYYFYNVLIMILFSFFMLYLIFFKMALFRMRNGETDIIISEFDALHVQHANLLRDGPRNQRILQWQRRFFFGEICFFSGFYILSLFLFAAMSLQPLLSQQTLPFRCKFPFGLDDPDEHPMAFACVYFFQCFCTLYMLVAIVVMDSLGGNSFNQTTLNLRILCENIRHLGESAAGTAGACSITSELVVWRELREAVEFHQKIIGLMNRINQTFYWNYVSQMGASTFMICLTAFEALLAQDKPMVAMKFQTYMFSAFMQLLYWCWMGNRTYYDSMEVATAAYDVHAWYEHSPLLQRQIMFIIKRAQKPLEFRAKPLFGFTFASFTSILSTSYSYFALLRTMSDMAGSNQVLQSVDSVVLYRAFWLCWHAVGISTAYNKYFCGLYNLLINVLVTIFYPIHLFLGLFLNPTPADL

FQNLSITITCFICSIKHYLLRRKLPQIHTVQVLLAELDKRVEGADEHAYFQQQLVVGAKNVVKLFSIAYGGANMAAISATLLSKERRLLYPAWLPFRWEASTFNYCAAVIYQIAGVTIQIVQNLANDIYPPMSLCIIAGHVHLLALRVAKVGEDAKKSMKQHNQALIRCIEDHKKLVRIFELTQDTLSQAELAQFISSGLNMCIVLFYLIFYVDNVFAYIYYAVYFVSMAIELLPSCFYGSMLIYEFQQLPSAIFKCSWLGQSREFYQNQRIFVQVSLKKIVPLAGGVIGIQLNSFLGTCKMAYSLYTVCNRMK

>BminOR33ab1-1

MGKKLLVVKSSVNTPVDTVACFDIFWMCWKLMGISINSNKWYVTLYDIVVNIFVTIFYPIHLTVGLFLVPVLSDVFKNLAIIITDVACSTKHYLFRYKLSKIREIQRLLKELDDRVVAQDERDYFNKSIRSAVRRMMLIFCVSYAGDTLASALEVLAKKDRELMYPAWFPFDWSANRYTYYAAVIYQIVGVSLQITQNLAHDTFAPVSLCVMSGQVRLLAMRVSKVGYDESKTLAEHEEELNECIEDHKKLLRIFDLLQDVFWYTQLVQFSSVGLNICLTVVFLLQFADNIFAYLYYTAYFISMALELLPACYYGSKMQEEFQDLPYAIFKSNWIAQRKSFQQNLRIFTELSKKQLTPTAGGIINIHLTSFVATCKMAYSLYTVLMNMK

>BminOR7a2

MTNQMPIVDFKSTHNSLSTMFDLIKGRRRTVFASRDAVIYLFNSFRYVGFNPPAKYRLPYFLYSAIITFFAVLFSPVIFNVGWLRDRNKLSVMEILTCVQASLNVMAVPLKCIALAMAMNRLRGIEPMVTELDERYTTPEDRAKIKQCAVTGNRLVFGFAVSYFLYETLTVISALVGGHAPLSLWIPNVDWHRSTWEYWLQVSFDAAVLFFILYHQVLNDSYPAVYIYIIRTQIQLLTSRVEKLGYDDQKSADENYQELSECIVIHQKILKIVKIVESVVSITVFTQFLVAAAILGVTMINIFIFADLTTKIASVTYFFCVLLQTSPTCYHASYLLADCDELRIAIFQCNWIAQNKRFNNLLIYFLHRSQDSIPFFALKLVPINLATNLSTSFREGY

>BminOR7a1

MFELITGRGIGIASSKDAFIYFFKGCTIVGLSPPKNSGLLYYMWSFSVNAICIIISPITGPVGFVIKYLQNTITTVQFLSGLQAALNLIGLPVKCSTVTHALKRLRGIEPTLTIMDARYTRPEDVALIRKAALMGNRLVFFFGTSYFMYMLFTVIPPLINGKAPLSVWIPFFDEHQSRIHICVQIVYDLFTMFFVLFHQSLYDSYGSVYIYVISTHLQLLVRRVGRLGTDATKSQDDNMKELVDCVVTHQQILELLATIEPIISTTMFTQFLIISSILCVTMVNMFFFADRSTQLASTLYFLCVLLQTSPCCYFATELKADSEQLPLAIFHCNWVEQDQHFRKVILYFMHHAQLSIELMAMQLFPINVATNISLAKFSFTLFTFIKEMGIGQNAKN

>BdorOR7a-like

MRSFLDLYYGRGSEEFETNESFQMIFSCWALLGIKPLKPYGFLRSLHVAFGCICLFMCPVFFAVGFMQVTQNSSMTIILTTLQATLNALGLPLKTFVAYFYIDRLRSVEPIFKSLDGRYQNPHGRFARREHVIQSAHLFVTLLALYFAYCTVSWLTSIFTHTQPLNTWLPLVDWIPHSTTQYWLHFVIEVVYVYFLLINQGMNDVYPAVYIKALRTHIILLADRVSRLGENPELTDEDNYKELIDCVRSHQELLQISRAVGSVFSITIFIQFTIAAAILCVCMLNLFLFADTNHRAITIVYYGCVLMQTLLTCYQASMLEVDCEKLPIAIFHCNWWNMDKRSRRLLIYFLHRAQQEISFVAVKFFKINLGTFLSIAKFSFTLYTFMNQMGVGENIKHQLD

>BdorOR49a

MDFVQFFWFPNALYRIVGYDFQQLPRAHWRKALMKAFLIFTTISGICTRIYMLFQLRELILSGDILNSFRLGVYISYAIDSNVKFFVFLLNAKRLRVIYQSLSNEYPMTSMEQKLYQVDKYSFKRARIMIVSYLSVTNSILIGPMLQSIFMYIIDLFRYGYAAAAFSYLHPTPMSYNFNYCTPHYYILIYISEYLNGHFCTTTNLGTDLYVCTFAGQFCMQLEYLGSSLEAYEPSMDNSKADCKFLMEWIRKHQLMLDLCSELNEVFGTTLLFKLISNCAVFCIIVVQLKLEGFGFGFLNFLSFFFVTVAQFFMVCQYGQKLITISENLALCAYKNRWYNGSQTYKTLLFNIIARAQKPARLTAKGFQPISLATFQIVMTMTYRVFAVLQRALD

>BdorOR47b

MISLSSKATISNTIASHNSYLTNHSYTHLKHTASKLQTILAPYRVLKEMLRCGEAVQPPHTCLFYFRSYIRLLGLWPAKRAAENQLYYFYNLLIMVLFSFFMATIICDLYEASSDFVLLGEDLVVVLGLYLIFFKMILFRMGNVDTDIIINEFDALHIKHARGLSGGPRNRRILQWQRSFFFGEMCFFSGFYILSLLLFAAMSLQPLLSQQTLPFRCKFPFGLNDPDEHPIAFVCVYFFQCFCTLYMLVAIVVMDSLGGNSFNQTTLNLRILCENIRHLGIVAAGASSSTSEAVAWRELREAVEFHQKIIGLMNRINQTFYWNYVSQMGASTFMICLTAFEALLAQDKPMVAMKFQTYMFSAFMQLLYWCWMGNRTYYDSMEVATAAYEIRAWYEHSPLLQRQLMFIIKRAQKPLEFRAKPLFGFTFASFTSILSTSYSYFALLRTMSD

>BdorOrco

MQPSKYVGLVADLMPNIRLMKYSGLFMHNFTGGSGLFKKIYSSVHLVLVLVQFLLILVNLALNAEEVNELSGNTITVLFFTHSITKFIYLAVSQKNFYRTLNIWNQVNSHPLFAESDARYHAIALAKMRKLFTLVMLTTVASAVAWTTITFFGESVKFAFEKETNSTITVEIPRLPIKSFYPWNAGAGMFYIISFAFQCYYLLFSMVHANLCDVLFCSWLIFACEQLQHLKGIMKPLMELSASLDTYRPNSAALFRSLSANSKSELINNEEKEPTDLDISGVYSSKADWGAQFRAPSTLQTFNGMNGTNPNGLTRKQEMMVRSAIKYWVERHKHVVRLVAAIGDTYGGALLLHMLTSTIMLTLLAYQATKITGVNVYAFTTIGYLGYALAQVFHFCIFGNRLIEESSSVMEAAYSCHWYDGSEEAKTFVQIVCQQCQKAMSISGAKFFTVSLDLFASVLGAVVTYFMVLVQLK

>BdorOR43b-like

MLSRNMRSFFDLYYGRGSEEFETNESFQLILWCWALIGVKPWKPYGLFRSLQMAFCWICLLMCPFTCIVGFIQVSKQTSSMTVILTTLQTTLQSVSLPLKALVTAFYLDRLRSVEPIFKTLDGRYQNPQSRFAITNNVIQSTHLFVTLVVSYFTYGTISWLSSAFTHTQPLNIWLPFFHWIPHPTIHFWLHFVIEVVYLHYLLIVQCMNDLYPAVYIKALRTHITLLADRVSRLGENPELTDEDNYQELSDCVRSHQELLQISRAVGSVISITLFIQFTIAATVLCVCMLNLFLFADASHRVITIVYYLCVLSQTSLTCYQASMLELDCEKLPDAIFHCHWWDMDKRSRRLLIYFLHSAQKEISFVAVKFFKINLGTNLSIAKFSFSLYTFMNQVGVGQDVKHEFD

>BdorOR92a

MNAIERNTNFTRFTAGPVRYFKFLGILLQQPEMPHSKYQRLLIVVTIALMFLHQIGYILEPGRTFAEQSAAAGLLNYTTVSGGKILFLVYNRRLLLSNHCQLAALYPSAAVERHYKLEHYLRIYAHVQTLLYNFFKYILIVYITYPIVQSFYDLWSSGVYSYIMPTLFWYPVPLEQSLFVYIVYLLFACFCSFCAGLIILSADLCLFSSVSQLMLHLDLLAQRIKELQPAEEGSLSALKAIIEYHQKILTIAKDVNSIFAPSILFSLASSSFILCFSAYQLLDDVSFIFALKVFLLLGYEMKQVVITCYYGDKLMDSSANLFTAVYAHNWTDGSPVYERLVLFMLVRTYRPIALKVAGISDVSLITLKQVLSTAYQIFTVLKTT

>BdorOR7a

MRKIADLFYGRGKHDFETTESFVLLSRSFAAIGFLPKIPKRIVDVIHQLICWSCIFSCPYLFVSGVVKTMHSLPITIVLAHLGVAINSIVFPLKAVYIKANIDRVDDIRKIFNALDKRYQRPQDQMQIRDSVKTCTRIFVVFCIVYWLFGISSWLVALCIHEYPHGNNLPFIDWLPESNLRFWLHFIFEVVFLHELLQMSLTMDSFPALYIRALRTHMNLLSDRVSRLGLNPDFSDQENFEELVDCIVSHQEILQISDTVGKILSLTTFFQFTVYAAILCVCMLNMFVFGDASTKLVTLVYLLPVFWQTTPTCYQASMLEADSAKLPLAIFHCNWLALDKRCHKLIIYFMQRAQQEISFTAIQLFVINLRTNLSIAKFSFTLYTFINGMGFGETLKDRLE

>BdorOR82a

MPEDLFRIQRNCLRVMGHQDIFDNNEASSSDEQKSKSKRQRRCFRHWQALKYVLLLLFMVSAQLPMMNYIIYHIDDLALATACLSIVFTNVLTVIKTSTFLTYKREFKSLMAEFESMYDELQEAGAKKCLVTVNVGAKRFVKLYFGACTSTGLYFTINPLVSMIWAKFQAKPIPLELPMPMRFPFDFESTPGYEFAYIYTVFITIVVVMHATSVDGLFVSFTTNLRGHFQALQYFIETNTFDKSEALLQRELGIYVQYHVRLLGLAQSVQRVFKPIIFGQFLMTSLQVCVIIYQLVMNMGVIMEMVVYCTFLSSILLQLLIYCYGAEFLKTESSAVSTAIQMSQWYNLPPRHRHVLRLMMLRSQREIIISAGFYEASLANFMSILKAAMSYITFIQSIE

>BdorOR43b

MRKIADLFYGRGKDDFETTESFVLLFRGWAAVGFLPKIPKRIVDIIHQIICWCSILTCPVWYFAGLIDMMDDLPITLLLSNLGVAINCIALPLKAIYIKNNMNHLHDINLLFKRLDERYQTPEENIQIRESVKTSTRIFAACCTLYWFFGISSGLVPLFAHEYPHGNVFPFIDWLPEGNFQYWLHSIVEIINLQYLLHLQSINDSFPAVYIRNIRTHIRLLTDRVSRLGLDPDLSDQQNFEELVDCIVSHQEILVISDTVGSILSLTTFFQFTVYAALICVCMLNMFIFGDLKVKVSTLIYLIPVIWQTVPTCYQASMLETDCSKLPEAIFHCNWLALNKRCHKLIIYFMQRTQEEICFTAIKLFQINLGTNLSIAKFSFTLYTFIKEMGLDAHYNQK

>BdorOR22c

MRRLLGPQVPIERSFFRIPRFSARVAGFWPQSTNRHRSWLTALRFYVNTFAVAVGGFGEVSYGFVYLYDLFSALEAFCPGITKVISLLKMTIFFGRHKRWQYVINSMHQLLLLDTSAEKRRIVEPLASFGSALSFVLLLSGSLTNTFFNILPLLKMGYYKWQSLEVELLLPFNVILPEMFVNWPYYPATYLVLTLSGAMTVFTFSAVDGFFLCACVYTSALFRMLQHDIRNAFAELQELEHSTLAQNMRIQHRLTVLVERHNKIIDLCSDFASEFSLIILMHFLSASLVLCFSILDLLLNSSSVGVLTYIFYSIAALTQLVLYCIGGTYVSESSLKVAEVIYDTDWYKCDVRTRRMLLLMICRAQKAKTIQVPFFTPSLPAFRSIVSTAGSYITLLKTFI

>BdorOR45a

MENFADVDKIVAAMTINVQLFTTSGKNFIFLARRKRFLRLNEALERLALKGNKYERELWNATNRPVLPITTAYSVSCQLTVNICVLLPIFKLLFYYIWYNEVVLTLPLPGIFPYDYTLPLYYILTTILTVLLVQLCANTITVVDGLFGWFVYNISAHLQIMRLKLEQLLQLHVDDPNFHRDFVALINYHREIIDLALELDAVYAPIIFLEVTSASLPICFLAYQLSYLSDPANVPFICLLLSSIVLQLMIYCFGGEKVQNECDQLSENIYLLIPWHKLPPKHSRLLLIPFIRSQRVLVLTGYFFTANRSLLVWVSYNKNS

>BdorOR88a

MAPQQEVFGAKSKLCAIEDLCAIEHPYQRYLGLKYVEFKRVNGRLVIPKSNILNFLLFLAVVDCTGNVIKTAIAINDRDVTKAQEVFAVFGMGLVMTMRGFMLGLNRGKLLKMFNAIDRIFPRSEHLQQHMEVEKVHNYIKKRFFYLHWFLTVSVCGFIFMPFVKFMAFHGFKSDAPVSEEFHVNASWLPFGVKDKVSTYPYIYVYELFLATAASHMLVVWDQIFVILISQLCMYYEYLGKLLAEMNVPDAMDPTKSDAVFKQLHDYIYMHQYLNNLAVQLNDLFNFSILSSDAGIAISICFNVVLITEAKNNLQIINYTIPLFVEVWLIYDASKWGQMLETVTARINERIYEQQWYDSSIRFGKYTLMWIQSTNEPFRLTVFNMFYVNMKHFQDMMMLAYQLLTFLKAKG

>BdorOR94b

MAVKKWSPRNTSSMSRTASANIIIAVLKALGYWQWTRDPHQPYIEKVERAYRIVLHTTLPFTFIALMLTGVLLSRDLDEIGSILHVLLTEFSLIVKTLHIWRKGGVAWRFMHEVANDPIYDLRQQSEWTKWQQAQRSFAIVSNTYFVAATTVVVFACIGAMMTPADVYVLPMNIYVPFDWHHPRRYWYAWTYNTIASLMTATANAMLDLVNCYFMFHLSLLYKLIGWRLSALRRSANEPPVIEQMSEIFQMHMKVRRLTTECETLVSIPVFSQIILSSFILCFCGYRLQQMEIMENLSMLFSTVEFATVMAVQIFLPCYFGNKVTESSDALTDEIFNSDWTTFDVPTRRFMILYMELLKKPANLMSVNYFIIGVDIFAKTMKNAYSIFALVLNMNN

>BdorOR10a

MNFRFLSRTFPLRDYYFYVPKLCLGALGFWPLDTSAPNASNVWAWVNLIILTIGVFTEIHAGCTVLKTDLELALDTLCPAGTSAVTLLKMALIYYYRKDLAWVLKRMRDLVYERDVSINTVKKHIVRAHAVMAARLNFIPFVMGFITCTSYNLKPLLMTLILYMQGQEPMWKLPFNMTMPSFLLHAPYFPLTYIFTAYTGYITIFMYGGCDAFYFEFCSNTAALLELLQNDLKSIIKFDQLSLTTEESTVLEWRLVQFIKRHNDIIELTRFFCKRYTVITLAHFVSAGLVIGASIFDLMTFTGFGIVIYIGYTIAVLGQLFIYCYGGSMVAESSVQLATVACGCDWHACNPRLRRYVLMIIMRSQRAISMSVPFFAPSLITFTSILQTSGSIIALASSFK

>BdorOR2a

MTLKINSWDAFKYHWRVWDLSGFRGPQRQSVWYIPYKLYTIAITLLFPIYYPICFTVESFLADNLNDFCEVIYIAMADMTLNIKFLTLFIVRRQLLELRPILKRLDARAKTEEEMNVLQEGIDSAKKCFLIILRLFYSAFVTSQLMVIFSAEARLMYPAWYPFDYQASRTKFWIAYGYQTIGFLVQCTQACSVDTYPQAYMRVLTAHIRALSLRIERIGRQNFSGVSSELMCSKENEMKRNYEELVSCIKDHKTIIELFSTIQKPISGTSMAQFVCTGVAQCTIGVYMLYVGFNISIMLNMAVFFVSVTMETLILCYYGDLFCQECEELSKAIYNCNWTVQSSEFKKVLCFFLFRSQRVNVLMAGNWIPVRLPTFVMVVKSSYSIFTLLSSFK

>BdorOR85d

MSKKMIEFGAFMSTANFWYSFNGIVAYDDIYRQPGDAPQQKSFAARFTTVLRQIYSLIGLVNLIWVLIIEASFVVVNFIENSDFLQAARNFTFMGFVIVSILKILSNLRQRSRLSILMQKLYEIYPKQSTDQPPYELQSHLSHYRRIGFMHAFTHAFTVGTYNFLPMINYLFLAPLLQHTDVVRELPYYCWVPFEWRDNWLYYPLYVSQVCASLTGLGGYLASDLLFCAATVQLIIHFRKLARDIEAYQAGCSCATADVCTQQAQRDLDFLSAAVYYHSHTLALCQLINEIFGLPVLINFISTSFVICFLAFQFSIGVPLDSMVALVSYMICCLVQFYMICSYGQELITTSENIGHAVYNHNWLVADVRYKKMLILIIRRAQKPAILKATTFVNISMGTLTDLLQLSYKFFALIRTMYAR

>BdorOR24a

MFLKFLSQSYPTEENVFLIPRFALRIAGFYPGDGNSRRMQAWLIFNFVVLVYGSYAEFMFGIHYLSIDVVRALDALCPVASSIMSVVKLAFLWWHREELDRLIKRVTELIATQNSRLKLADKRRYFTIATRLSASVLFFGTTTSTLYTIRAGIVNYLSHLRGEEIPYETPFKMIFPEPLISMPIFPLTFIFSHWHGYITVAGFAGTDGLFLCFCMYIGTLLKALQYDTKDLLSDVGCGERKHSSEAEIKESLKMIIARHNEIIDLVKRFSAVMSGITLGHFVTSSAIIGTCVVDMLLFSDYGVLVYLVHTMAVSTELFLYCLGGTVVIECSSQLATAVYDSNWYTHTVDVQRMVLLIIIRAQRSLVLKVPFFAPSLPALTSVSKQLRLYPRNMLHQV

>BdorOR94a

MWAQHIIKLELKMTPSAAEQQERIGVARVLMHFLQILGAWPILPEHHHQNARSTQCRTWLARNYRYLLHLPLTFTYNTLMWVEALTRWERADHILYISITEVGMMALTLNFWRLDQRAYHFMHELCYSDHLALRNQAERQWWRAKQRSFTRIAVCYIGGGAGVLCTAFGATLLVNGYSLPYDYWLPFEWHNAQNYWYAYGYELVAMSLTCIANVTMDMMLCYYLFHVALLYKLIGIRLMALQHLSERLAVQQLINIIKLHKRVKRLTAQCEVLVSLPILVQIVLSVFILCLSAYRLQSMQINENPGQFFAMLQFASVLTLQIFLPCYFANEITINSDALTTCVYNSNWEEFSPPTRKLMNLYMELMKRPEQIKAGNFFLVGLPVFTKTMNNAYSLLALLLNMSK

>BlatOR2a-like

MTLKINSWDAFKYHWRVWDLSGFRGPQRQSVWYIPYKLYTIAITLLFPIYYPICFTVESFLADNLNDFCEVIYIAMADMTLNIKFLTLFCVRRQLLELRPILKRLDARAKTEEEMNVLQEGIDSAKKCFLIILRLFYSAFVTSQLMVIFSAEARLMYPAWYPFDYQASRRKFWIAYGYQTIGFLVQCTQACSVDTYPQAYMRVLTAHIRALSLRIERIGRQNFSGASSELTCSKEDEMNRNYDELVSCIKDHKTIIELFSTIQKPISGTSMAQFVCTGVAQCTIGVYMLYVGFNISIMLNMAVFFVSVTMETLILCYYGDLFCQECEELSKAIYNCNWTVQSSEFKKVLCFFLFRSQRVNVLMAGNWIPVRLPTFVMVVKSSYSIFTLLSSFK

>BlatOR85c-like

MSNIIRFEAFLRIPSFFYRSVGIDLWNTNGGSLQSAVFYISLFNVNVWLLSELIFAVLMFTKNFIQATMTLSYAGFVLVGSIKMYFMWRKKAEMTRFLQLMNAIFPRTEPQQKMMNLRSHLRQCTIVMSAFAMIFMILIWTYNLYPYMQRQIYDCWLHMRSVNKTLPYESYIPWNWHDHWTFYLYYTSQSIAGYHSASGQIASDLVLCAM

ATQIIMHYEYVAQRITEYQPQALKGAVRDVKESELYRKDMRFLCDIXEYHANVLSLSDIMNEVLGVPLLVNFMTSSFVICFVGFQMTMDAEPDYMVKLFLFLFSSLFQIYLICHYGQQLIDASNNVSRAVYNHDWVHSHVHYQRMLVLVTARAQKPAMLKATSFVRISRGTLTDIMQISYKFFTLVRTMYNN

>BlatOR85d

MSNQIIHFESFNTLANIFYTSIGLDAYQKAGQRTNNIRRQLLSIFFIITIANMNITLLSELLYIFMAFANNNNFVEATMLSSFVGFVIVGDFKIYSIWRLRARITAMMQALHALYPQTLAEQRQYEVQRVLKRYQRFAYAFVMLHELLVWSYNLFPLLXYFIYEXWLALRVVGKTLPYNCWTPFDWHVNBWRYYLMYLTQIAAGQACLSG

QLANDLLLSAVSVQLIMHYRQLARRIELHVAGGGGDGDGGGSGSKWRXAATNVSRZHDLCFLRSIIAYHQQILNYFYTPSRLSQALNDVFGNSLFISFASTALIICFVLFQITIGANIDAIVMLAFFLFCSLVQIFLICFYAQQILEASEYISFAVYNHNWFDSDLRYRKMLVYIMARAQKPSKLQATSLVTVSMPTMTNTHGLFDKVVQNDP

>BlatOR43b-like

MRKIADLFYGRGKDDFETTESFELLFRSWTAIGFLPKLPKRIVDIIHQIICWCCIFTCPVWYFAGVIDMMNDLPITILLSNLGVAINCLALPLKAFYIKVNMNHLCDVNLMFKRLDERYQTPQEKMQIRETVKISTRIFAACFTLYAFYCIPSRLLPLFTHEYPQGNRFPFIDWLPEGNLKYWLHSIIEMLYFQYLLQLQCTNDSYPAIYIHSIRTHIRLLTDRVSRLGLDPDLSDQQNYEELVDCIVSHQEILVISNTVGSILSLTTFFQFTVYAALICVCMLNLFIFGDLKTRVSTLFYLAPVIWQTVPTCYQASMLQTDCSKLPEAIFHCNWLALDKRCHKLIIYFMQRXQKXIXFTAIXLFVINLRTNLSIAKFSFTLYTFIKQMGLDTHHN

>BlatOR59b-like

MRSFFDLYYGRGSEEFETNDSFPLILWCWALIGVKPLKPYGFFRLLQMAFCWFCLFMSPVVFIIGFIQLTKQTSSMAVIFTTLQASLNSLGLPLKAFVTAFYLDRLRSVEPIYKSLDARYQNPQARFAIRNNVTQSTHMFITLVVSYFTYGTVAWLSSAFTRTQTLNIWLPFIDWIPHPTIHFWLHFLIEVVYLYFLLITQCMNDLYPAVYIKAIRTHITLLTDRVSRLGEDPELTDEDNYHELVDCVRSHQELLQISRAVGSVISITLFIQFTIAATXLCVCMLNLFLFADASNRVITIVYYLCVLTQTSLACYQASMLELDCARLPDAIFHCNWWDMDKRSRRLLIYFMHRAQKEISFVAIKFFKINLGTNLSIAKFSFSLYTFMNQMGVGQDVKHEFKSVG

>BlatOR7a-like

MFDLVKGRGRTVFASRDAVIYLFNTFRFMGLNPPSHCRLLYYFYGSIVTLFAVPLSPLIFNVGWIRDRNILSVMEILNCVQAALNVIGVPIKSITLALSLGRLRSVEPLLSKLDARYTEPEDLAKIRACAITGNRIVFGYIISYMMYETLTMVTALLGGHAPLTLYIPYVDWHRSVWEYWLQASFDGAMLFFLLFHQILNDSYPAVYIYIIRTQVQLLTNRVRRLGTANKSQEETYHELQDCIITHQEILRLVSIVEPIMSLTLFVQFFIAAAILGTTMINIFIFADFATRIASGTYIFCVLLQTFPTCFYATHLQSDCEQLSMSIFHCNWLSQGKRFNTMLLYFLHRTQADIPLFALKLVPINLSTNVSIAKFSFTLYTFIQKMGVGKNLK

>BlatOR47b

MISLSSKATTSNTIASHNSYLTNHSYTHLKHTVSKLQTILAPYRVLKEMLRRGEAVQPPHTCLYYFRSYIRLLGLWPAKRDAENQLYYFYNLLIMVLFSFFMLYLIFFKMILFRMGNVDTDIIINEFDALHIKHGRGLSESPRNRRILQWRRSFFFGEICFFSGFYILSLLLFAAMSLQPLLSQQTLPFRCKFPFGLNDPDEHPIAFVCVYFFQCFCTLYMLVAIVVMDSLGGNSFNQTTLNLRILCENIRHLGIVAAGASSSTSEAVAWRELREAVLFHQKIIGLMNRINQTFYWNYVSQMGASTFMICLTAFEALLAQDKPMVAMKFQTYMFSAFMQLLYWCWMGNRTYYDSMEVATVAYEIRAWYEHSPLLQRQLMFIIKRAQKPLEFRAKPLFGFTFASFTSILSTSYSYFALLRTMSD

>BlatOR67c-like

MPSFKSSEPAPTVPDFVDIPLFQIKFMGAKLFKWIPDEPNSKLQIILLGTFCIFATFNFTSMLLFVIKDQLETSLDITEFILFWGFALNAMMKGGTMVCFRHEIESILKGLIAKHPKTAEERAAYQLVPYFRTINISNKYLSIWHLSITSIFVVHPLIASIHGYVSREDKNKSFDFTLPFMMTYFYDINQPIAYAISYLLQCCGAFHVSLLFLSGDLLLISMVHLVNMHFGYLIYKIESFQPTGTDADMKVLGPLMVYHNEMLNYAERIDNTFGVATLLNYVGSCLVLCLIGLQIAMGSEAVIVIKFIGFLVSTIVQVFFVSYFGNNMKDLSTGISDAFYNHPWYDANYKYMRMLVLPIARSQRYAHLTAFKFFEISMDSFKSLCTTSYQFFTLLRTSMEEEDS

>BlatOR94a-like

MAIKKLANFRTLEPILTVLGLWQGGDSSWFKRHYRYYQLFMHTTITFTFASLMVLEVIYSESLDYAIDVLKYMLVEMAIICKVLNAWYYAQPTAELVNELEDSAIFELHTSAEEQMWQKSQKNFNKLTXIYMGTGLNSAFCALLAAALMDAKELPYALWLPYDWRDTYFWGIYCYECIAMPFTCLCNITIDLFQAYLLLHLTLCFRVISMRLERLESAGTEDAITTELLNNIKMHQRVKELALKCEQVISLALLSQIMLTFLILCFIIYNMQNVETANDIAHFSENPAHFLAMLQYALIISMEMFLPCYYGNELTVESEKLGIHLYSCDWTAMSAVNRRLIYVYMESLKKPVVLCAGRFFEIGIPIFSKAMNNAYSVLALLLNVNDDEEH

>BlatOrco

MQPSKYVGLVADLMPNIRLMKYSGLFMHNFTGGSGLFKKIYSSVHLVLVLVQFLLILVNLALNAEEVNEL

SGNTITVLFFTHSITKFIYLAVSQKNFYRTLNIWNQVNSHPLFAESDARYHGIALAKMRKLFTLVMLTTVASAVAWTTITFFGESVKFAFEKETNSTITVEIPRLPIKSFYPWNAGAGMFYIISFAFQCYYLLFSMVHANLCDVLFCSWLIFACEQLQHLKGIMKPLMELSASLDTYRPNSAALFRSLSANSKSELINNEEKEPTDLDISGVYSSKADWGAQFRAPSTLQTFNGMNGTNPNGLTRKQEMMVRSAIKYWVERHKHVVRLVAAIGDTYGGALLLHMLTSTIMLTLLAYQATKITGVNVYAFTTIGYLGYALAQVFHFCIFGNRLIEESSSVMEAAYSCHWYDGSEEAKTFVQIVCQQCQKAMSISGAKFFTVSLDLFASVLGAVVTYFMVLVQLK

>BlatOR59a-like

MAFEDVKEIFRSHWTIWKWMGQVTHPRYPKLYNAYSILVNIIFSLGYPVHLVIGLLQEKTLQGSLLNLTISLPSIICVLKFYNTWRNFDKVRHLEQMYNTLYARLDHAEDLTYYRKVTAPNAFRVVNAFKVICVGMAVTAELTQLYVGFVYGWRLMYPGYFPFDPHGSTAGYVTAHIFQFIGLLTQISQNLMSDTYGAVCLALLAGHTHLLGQRLARIGYDKGKTQEQHNQDFVDFIVDHNMLLNCQRTLVDIIGMGLFALIISTSLLLAIVIIYPMFFVDNALEYAYYVFFMIGALMEVFPTCYYATHFEYEFEGLTYKIFSCNWVDQNRAFKKNLIVCLEQSLKARYVFVGGMFRINMQIFIAICKGAYSVFTLALNYK

>BlatOR42b-like

MSKILRFRSAIVYKSRDALTYLFNIFTFVGTNPLENRSQRYYRLYYFYSFTVNFICCLFCPLSFHIGYIKLRHVLTNSQLLAAIQNAVQVSGIPIKILVITWYMKRLRHAFDILDELDVNYTRREDLAKIRECVRRCRKIVLIFCFPYYSFELSTIALGVVQNRAPLAAWVPFLNGQRAAWEYWTIVLWDAFVMFFLLCHQLGSDTYPPIFINIIRTHIQLLITRVNRLGRAGALTADEHYEELLGCIRTHVQIVSIAKIVAPVISVTLFTQFATTATTLLNWLGNVEYPENIISLAFFSCQLLQILPCCSSASQLIADCERLPDAIFHCNWVDLDRRFRRAILFFLQRTQNPIRFSCLKLFDVKLETSVAIGKFAFSLYTLIEETTVGTNTDN

>BlatOR92a

MNAIERNTNFARFTAGPVRYLKFFGILLQQPEIPCSKYQRILIVVTIALMFLHQIGYILEPGRTFAEQSAAAGLLNYTTVSGGKILFLVYNRRLLLRSHCQLAALYPSAAVERHYKLEHYLRIYAHVQTLLYNFFKYILIVYLTYPIMQSFYDLWSSGVYSYIMPTLFWYPVPLEQSLFCAAFIILSADLCLFSSVSQLMLHLDLLAQRIKELQPAEEGSLNALKSIIEYHQQILTIAKDVNSIFAPSILFSLASSSFILCFSAYQLLDDVSFVFALKVFLLLGYEMKQVVITCYYGDKLMDSSANLFTAVYAHNWTDGSPVYKRLVLFMMVRTYRPIALNVAGISDVSLITLKQVLSTAYQIFTVLKTT

>BlatOR67d-like

MTIKHIKPTASFAKLVKTVRFISSLVGADVSTENYQVNIITIIVIVCIIIYFIFTATTVASVFSENWTYLLEASCMVGSVLQGITKLISGISRTNEVSGMRLELEELYRVYESKGESYCKVMNACCDRVWQLIKMVGLIYGAAIVGNLLLTSFMVFFANQKIYIMHFFIPGVDVETSFGYLLTTALHSLCFLAGAFGLFGGDLFFLIYLGQPELFRDILILKVHELNEAAAQKNNKTESLLISIIEWHQYYTDYNERCNELFYYIITMQILTSGVSIVFTMYIILMGDWPGAYLYILIALSGLYLYCIIGTNIQTCNETFFEELYNINWYELDVKGRKMMILVLMKSQNPSEIKIGGVLPLSVQTALQITKTIYGIFTMMLGFLDEEQ

>BlatOR82a

MPEDLFRIQRNCLRVMGHQDIFDNNEVSSNDEQKSKSKRQRQRRCFRHCQALKYVLLLLFMVSAQLPMMNYIIYHIDDLALATACLSIVFTNVLTVIKTSTFLTYKREFKSLMAEFESMYDELHEAGAKRCLVTVNVGAKRFVKLYFGACTSTGLYFTINPLVSMIWAKFQAKPIPLELPMPMRFPFDFESTPGYEIAYIYTIFITIVVVMHATSVDGLFVSFTTNLRGHFQALQYFIETNTFNKSEALLQRELGNYVQYHVRLLGLVQSVQRVFKPIIFGQFLMTSLQVCVIIYQLVTNMGVIMEMVVYCTFLSSILLQLLIYCYGAEFLKTESSAVSTAIQMSQWYNLPPRHRHVLRLMMLRSQREIIISAGFYEASLANFMSILKAAMSYITFIQSIE

>BlatOR85c

MSTIIKFEQFLGLSSFFCYNIGIKLRGPNDGFWLNFWLYLTSINLFLTVFAECIYIIMTIRSDFIVAIMTLSYVSFIVVAYVKWYYLYNYQTERNAFFQRLEALFPHTKSEQENIKLREYFRLNKLATRGYTITFMVVIWLYNLYTISQRFIYTKLLHVHIERVLPYQALYPWDWRDNWTYYVIYVTQDFAGFHATCAQIAYDLLLCILSIQLIMHYDHISRSLEGYQTKFAEVHGVDINNGLPQLACAAVELRAVKEDIQFISNIVSYHNELLSLSKSLNKLFGMPLFVNFFTSSAIICFLSFQMSVTREVDLLMKLAVFLFFSVMQVYLICHFGQLLSDASTNVASAAYFQDWSYADIRFQKMTILVAERAQEAAALKATNFITISLDTMTVIMQMSYKFFTLLRTMYAD

>AludOR59a-like

RLWLLLGCVREPVRYQLLYRLYSTGINTLIIVVYPGTILLALCNSTNSKDLLQALPICAGAFACSAKYVSYQRSLRLVRQVEQLFNVLDERIQLAEDRNYFAAITRNTNVLLNVFRSVCVFFFIVTIISFISSIGERGLAFAVALPFDWRASTLRYVGVVTLELLLLTCDLLQSMANDSFPAIALCVLSSHTRLLGVRLARIGYKSKDEWANVVELQRCIVDHQHLHRLHATLQEIISMPVFVQYAVTAFQDCFTVITLVFYARNASDRLLYCMYLLALQLQIFPTCYYGTVCANSMDDLRQHAYASNWVEQSRLYRRLMCIFAARSMKITMAYAAGIIPIHVGAFLATLKAAYSFYTFVEGVRK

>AludOR43a3

MEIWVFFNFSSDISIWNKIGHIMHNYQNLQLYSVNVKIFLKFGFIGSASKMKMSLMALVPFITEIGQLFNLFVTLDKDISETGLNFFFLGVVTSGIIRQFIITALKDEKYVRLLRWVECWYHELERSGNPQILGKLQDITKRAQTLTRIGFYAACIGMVGAFSFPFSFEERKFVVDVSYPFFDVKQTPFYEFFFLLQALVLVPTFCCVYLPFTNLLITFLMFGEVVLLDLRLKLNNINKNDETVMLRELKECIAYHKKIIEFRNDVEDLVALVNFFDVALFGLMFCMMLFFISMVDDLQSILTALIFIGFTFYTIAISYYHANKFTNESFEICYAAYNTPWYNGNLQMRKCVAIMIARSQKPLQITAGGLYPMTLETFQAIVRISYSYFSLLQGLNQ

>AludOR83a1

MLPARNQNFSATQVCANPNGCISNVKRQDLFKFVRWLMWFTAFCSIPLENCLPRRLKDYSAHLNVALEIFLLLAVFHIFVLYIFTFYFNYENGDLEFLVSCSIEMLLYSWALSVKIYFRRIRPDLVCGIMEYVNREHITHSAVGFSYVTMKDCLEKSNAGTKIFVGCCFLGVTFRLFLPIFYEERSLPLPCWYPIDYKTTFIYPMAYFLQIVAQLQLAAAFALSSVYFMVLCFLLSAQFDILNCSLKNIVATAYIYMGASRDELIKLREQQYIADAEVNQYFVVKEVAVDLDCLQHLLNPQIPSSANFREGFQRALKQCAEHHRFILCALQKIESLYSMLWFFKTLEVTFAICLIAFDVVKSSNGKSILQVLSLGQYMVLVLWEMLMICYGGEIIYVNSQRCDEALLRSPWYLHSREVRA

DILFFLMHAQRAFKLTGGKFYPLQLQKFREIVTTSFSFFTLLQNMDMRN

>AludOR24a

MLPKFLFQSYPTEKGLFLIPKFTLQVIGFYPEHGKSTRIQAWAIFNMVVLIYGCYAEFAYGLHYLPIDAIRALDALCPVASSIMSAFKISFIWWHRVELERLIKRVGELIAEQNNPRKLACKRSYFTIATRLSASVIFCGFCTSTLYTIRAGIVNYLSYWRGEIIPYETPFKMIFPYPLLSMPLFPFTFTLSHWHGYITVAGCTGADCFFLCFCFYFGTLLKALQYDLQELLDDVDSEGSKNYAECEIEESLKHIVARHNEIIDLLKKFSTLMSGLTLGHFVTSSVIIGTCVVDMLLFSDYGIFVYVVHTMTVFSELFLYCLGGTVVIECSSQIATTIYCTKWYTHNLRVQKMVLLIMIRSQRSLVVKVPFFALSLPTLTSILRFSGSLIALVQSMI

>AludOR33ab1

MLRDLFISKAVRNVPVDSDSFFKIFWMCWKLMGIGIESNKWYIWVYDIMVNLFITIFYPIHLTIGLFLVPTLADVFKNLTINITDVTCSVKHYLFRYKLGKIRKLQGLLKQLDQRVVAEEERDYFHRDIRIAVRNIMCVFCISYFADALASTVDVLTKKERELMYPAWFPFDWAANRYRYYAAVFYQIFGVSMQITQNLAHDTFAPISLCVMSGQVRLLAMRVSRIGYDKSKSRLTHERELNECIEDHKILLRSFDLLQDIFWYTQMVQFSSVGLNICMTVVFMLLFVDNLFGYVYYSVYFFSMAIELLPACYYGSKMQEEFQDLPYAIFRCNWLEQRKSFQQNLRIFTELSRKQLTPTAGGIINIHLTSFVATCKSAYSLYTVLMNMK

>AludOR63a-like

MYSATEMEELKTRNRHGIRELLRISYIVGVNLTTQTSYKKRLLLINVLLIIASSIGLYPHWILIQKAEGNIPLIAETATTALQTTTIIVRMVYYLFAQHKFQNLLHKAETHEILQSCEIFKTDMPITIQLKQEVNAIMSTAWQEARWQLLLCISCTCCILSNYFFCALLKNLYHQIMGTPDYVYILPFTGYPMFLDKGVFCYAMDMFFGACCLIVAGMGGVALYCPFLILCKNACGLVRSLSMLLERSTAPLVPKHRRDEYLRYCVIQHQRTLDFINDVNQLFKHISLSQFLHSLVIYGLVLYEMNFGLESNKVIFVRMIMYICAALTGDCMYYINGQQLATELETIPVACYRCEWYNESEEFKKTLQMIIMRSNKSFFFEISWFGIMSLTTLLGIARASFSYFVLLQDIEEK

>AludOR69a2

MTFRIYKLQNFLEYPELAFKWTFFEPFVWSGNQHRHQPSYGLQMKRLIYIFITSTLAAQVLALFFNLYVTHNSLETESNESGESDLFEALALMCFFSSGLYKMWNIFWRRDDIGRVLEELKELFPSATIQTKLAEANDYKTELKRGTGGCYRFTYYDQKSRTLMGRLARYFTFSYTYYNLVPVVQLIYEMFSPNQSITYTAQTNAWYPFHNQNKHSTFIGFVMSFFVQSAAEYAGAGFVMCGIFLILFLTTQMKLHFDYLASALETLDASAPDALKQLKRLINYHNHLLRLCKDINDIFNLTFTLDLIVATIAISLMGLTMIMISFSRAVMYSAGVSFFLINTYVFCKNGDELTTVTEKLSTALYYSNWYEGSVEYRRMIIFFIMRTSRPCEYQAYGYTVISMETYMRILKLSYQLFTAFGALD

>AludOR7a6

MLDLLRGRYRGIYQSRDALTYLFNIFTFVGTNPLEQRSPKYYHLYYLYSVTVNFICCFFCPLSFHIGYIKFLKVLNTTELLAAIQNAVQVTAIPIKIVAITWHMKSLLSARAVLDKLDANYKRAEDLLRIRKCVQSCCKIIVVFCVPYYSFEVTTIAFGVLQNRVPLAAWVPYLDADRAAWEYWTIVGWDTFVMFILLAHQLGSDTYPAVYISIIRTHMQLLVERAKRLGTDRALNAEENYAELLACIRTHGQIKSLAKIVAPVISITLFTQFATTAITVLNWFGDVEFPENIISFAFFSCQLMQILPCCYCASHLIADCDLLPNAIFHSNWIEQDRRLRKTVLFFIQRAQSPLRFSCLKLFSVSLATGVAIGKFAFSLYTFIQEVGDKSE

>AludOR59b-like

MSNFPKPQLKQLLCSGAATKAPTVVEERPPAEAPMSNAQRIRVQFERATRTPDNNKTLEEHKERSYGAMHDIHSKDGLTYLFRSFMALGVLMPEKHKFLYCIYAILPLGLITFYLPTAFALSYFTLDYSTIKIGNLLTSVQVGIASVVGGVKLLVMAFKLPKLRACEAIMTELDARCKDEDEIEVLRKVVRQGNRVLVLILICNLIYSTSTFLGAALKGRPPYNLYNPVVDWRNSKWEFVWAALWEFILMDGLCAEEAITDSYAPIYVCIMRAHMKTLLMRIQKLGTKPERTPEENYEDLKMCIKDHKSLLKLFDIVHPIISTTYFLQFMTTSLMVGCTLLNIMIFAVDNSARVGHLAYVVALLMEVYPLCYYGQSLLDDSNRLANTIFHGNWMEQNEKFRKMLVVFTQHTQKPMELLAGKLIPINLTTFVGIAKFSFTLYSFINNMGVKERFEGL

>AludOR71a2

MSFDTIANSRLLSRALTLLGLWPVAMGTGSCLQRYYRYYQLFLHITLTFTLTLLMWLDVIFSENLDHATQVLKFLLTEMGLVVKIFNIWYYARMASEFLIEWETSELFVLQTCAERAMWQRAQRTFHKVVIVYVFCSFNTAICALIAVPFMNTRELPFPFWLPRKWREDYYWAMYSYEFIAMPFTCLCNIKTDLFQCYFLLHLTLCLRVIGMRLERLSNAVASESVTEEFVKIIKIHQRVDEMAKCCERIVSLPVLMQIMLSSFIICFIIYRLQNVRFSDNPAEFFSMLQYVVAMSLQIFLPCYYANELTVESQNLSHHLYNCDWTGMPVYNRRMIFVYMEYLKQPLVLHAGNFFEIGLPIFSKTMNNAYSLLALLLNVSDENQ

>AludOR74a2

MRYLPSTYYKPLLPDGRAPPMDWQLYGFLSANGWPLASNVSKKRLIADAVSVICQFISESSVGFGEMRLMYESIDDISFVCTILAPFLILLEMMLRAFNIVYKRNSFRSHLDEFYKKIYIECDWNPELFEKIRRQQLPTKYSTFGYIITLVTYVYVPVSGLVLGQRLLPFPVSYNFDTTASLPGYLLLLAMGLWTGVAVVGPLVAEPNILAMQILHLNARYTLLLRDLSNIAKNTIEMHTRGKGEDTVSVTSRFCYGLSDIIRTNVKLNDFAKSLQAQYSFRVFVMMALSATLLCVLGFLTATLGLSTDNIRFISWIIGKVVELLIFGRLGTTLSTTTNALSTSYYSCGWEDIVLHSTDAAENAKIVKLMTLAIHLNSTPFELKGLNFFSVNYESVVAILRGAFSYFTVIYAYR

>AludOR85c

MALIMRFEEFLHLPNFFYRCVGLVLWGPSGGLWQRLFFHFSVHNLFLTFLAELYFIVVTFKTDLIEATMSLSYASYVAVALVKYYYMLRRKKNMIIFLQRLEVIFPRTKLQQENLRLSHYLQLSKFVTKSYTAMYMVLISIYNLYAIGTRLIYTNWLHVNVIDPTLPYSASYPWNWHDHWSYYVLYVSQGLAGWHATCTQMALDLLLCTSATQLIMHYDYISLSLEKYKSKYAEVYALDVPQRVRVVMELKAFREDMQYISGIVAYHAELLSLSQLLNELFGVPLLVNLFTSSALICFLGFQLSVTRQLDLLIKLALFFFGSILQVYLICHFGQLLIDSSTNVVNAVYFHDWIHADIRYQKMLILIAKRAQRPAILNATSFISVSRGTMTDIMQLSYRIFALIRTMYNE

>AludOR7a7

MRVKKHSCCYQIYVPIYINMYIIFLQKALAPIIRQMFDLIKKHGRPVFASRDAVIYLFNSFRYLGINPPNKYRIPYFFYSFITCFIVLFSPVISNVGWLRDRNKLSVMEILTCVQASLNIVAIPIKSIALAMSKNRLRAIEPLVTELDKYYTRSEDRGKIRKCGITGNRLVFGFAVAYFTYETLTVVAALVGGHAPLSLWIPNVDFHRSAWEYWLQVSYDAFILLLLLYHQVVNDSYPAVYIYIIRTHIQLLAARVERLGYDEKKSVDDNYKELLECIVIHQEILKVASIVESIMSVTVFTQFFVAAAILGVTMINIFIFADLTTKIASITYFFCVLLQTSPTCYHASYLLADCDELRLAIFKCNWVDQNKRFNNLLIYFLHRSQDSIPLFAMKLVPINLATNLSVSYFGL

>AludOR33ab2

MLMRQIDTIAIFRRLFITWRVLGIILWPFNKYLRIVYDLLMNIFVTFGFPLHLVLGIIFSTNQEQFFINLVIGIASVSCTLKHLLLRYRLSEMLMVNEILARLDERVHTNTDYDYYKRFIERPCNFMINFFTRCYFAVSITALLTALVTGELLYPAFVPLAWRTSISQYIVAILFQFAGVSLQIVQNIANDAYGPVVLCMLSGHVHLLANRVSRVSHSSEENIAGNSTDLALCIEDHKLLRSTSNTVERIVSASYLVQFVGVGINLCIGLVYLLFFADNYFAYVYYSLHVTAIMIELFPCCYFGSMLECEFHDLSYAIFRSNWPTQPRAFRRNVVNFTELTLREVTLYAGGMIRINLDSFFATCKMGYSFFTVIQSMK

>AludOR49a

MGIKEFVQVFWGPNTLYQLVGYDFQLLPRPYWRQVLMNAFLIFTTISGICTRIYMLMQLRELIISGDILNTFRLGVYISYAIDSNVKFLVFLLNAHSLRKIYESLAAEYPQTYSDRKLYKVNKYSFRRMRLMICAYLLVSNSILLGPMLQSISMYIIDLFRYGYANATFPYLHPTPMPYNFDYCTPRYYIPIYLSEYLNGHFCTTTNLGTDLYVSTFAGQFCMQLEYLGYSLETYEPSVENSKADCEFLMKWIRKHQLMLSLCADLNEVFGTTLLFKLVSNCTVFCIIVVQLKLEGFGWGFLNFLSFFFVTVAQFFMVCQYGQKLITISENLALSVYKNRWYNGSKEYKKLLFTIFTRAQKPAKLTARGFQPISLVTFQIVMTMTYRVFAVLQRALD

>AludOR74a1

MLYRPRLSNGKYISLSWPITAYRLLNNICWPLRDDASLLEWLFDRFCWVLCFIIFIQHNDAELRYMIVNKNNLDLMLICGPTYLILIEIHIRAFQLGLNKEGFKRFLQRFYAEIYIDKPTHPKHYAKIQQRLRPIWFYSALYFSTLFSYVIIPLTNFFNDTKAPLYKMYYPFDITPYPIYIAVILSNIWVGYTVISMIAGEDNIISEVLLHLNGRFVLLQQKLQEDTERLLHEADGKNIADELSRHIVEATRENVRLYKFCEDLEREFSFRIFISLSFSAALLCVLGFKVFTNPMDSFGFIFWICAKILEMLLIGQLGSTLIATTNQISSAFYESNWERVIQKSTDSYANVRLMKLLLLAIGTSQKPFVLTGFKYFSVSLTAVLKILQGAGSYFTFLTSMR

>AludOR74-like

MHYRPLLPNGKEAPLSWQIKSSFIFGTWPTTENTTNLEKRLHRLIFLWCALLLCAAFCGQFYEVVLQKDDIVIMIQVMLNLLLTLECIIRIGWLALKKDNFQEFLKELYSKIYFDENIDKDMHRKIRKHLRPANIFSVWYLLTMISFYVEPIQGIIIGERYLPYRMTIPFDYRPWPIYTAILIICLNVGCLVLTVVMSESYMLATSIFNLNGRFLLLEEEISNLGDAVLKYENSKTMADHFNQRLIALIKRNVDLIKFADRVESQFTLPLFVMMADSAVMLCLVAFNLNEMGLVPQSIKYIFWLLAKVLELVVLGYLGSVISTRIDGLGTTYYASGWEKVIHKSPNTKANVRTMKLITLAISFNQRPFMLTGMRFFYVSLETTVTILQVAGSYFTFLRSIR

>AludOrco

MQPSKYVGLVADLMPNIRLMKYSGLFMHNFTGGSGLFKKIYSSMHLVLVLVQFVLILVNMALNADEVNELSGNTITVLFFTHCITKFVYLAVNQKHFYRTLNIWNQVNSHPLFAESDARYHAIALAKMRKLFTLVMLTTVASAVAWTTITFFGESVKFAFDKDTNSSITVEIPRLPIKSFYPWNAMSGMFYIISFAYQFYYLLFSMVHSNLCDVLFCSWLIFACEQLQHLKGIMKPLMELSASLDTYRPNSAALFRSLSASSKSELINNEEKEPTDLDISGVYSSKADWGAQFRAPSTLQTFNGMNGGNPNGLTRKQEMMVRSAIKYWVERHKHVVRLVAAIGDTYGGALLLHMLTSTIMLTLLAYQATKVTGVNVYAFTVIGYLGYALAQVFHFCIFGNRLIEESSSVMEAAYSCHWYDGSEEAKTFVQIVCQQCQKAMSISGAKFFTVSLDLFASVLGAVVTYFMVLVQLK

>ZtauOR94b.2

MDKLAVLSSRIFPSDPSKGKIGSIEYNVWLAQLFGVPVVGLKAESPLLRIALGIYGVLITLLVTFIYTGFEIYDMILCWPNLDDLSQNICLSLTHIAGVLKVINILYRLDEVAHVVRRIEYSAKTYVVSKSQLVAFYRGEFENKIPLTIYASLVGFTGVLGIIYLFYNPVGVAGQIFPYRVKLPDWMPFGLQLAYMGISVLVFALQIVSIDYLNVTMINQIRFQLKILNLAFEELKLDCGTKKKAHINHDKRLQTIVEHHCLLRDLRIDVEEIFRMPVLLQFFTSLIIFAMTGFQAIVKTENSNGAALIYCYCGCIFCELFVYCWFGHEVSEQSKTLTTSGYSSHWFEFDQRFKKSLLIFMCNSQTPFVFTAGGFMSLSLPSFTGILSKSYTVIALLRQVYSR

>ZtauOR94b.1

MAAKKCSSNSSTSISRISAARFIIFTLKAIGLWQWTADSSQLANSRLSYLFKLQRVHGLILHIPISFTYITLMFTAVLLSQDLEEISSVLHVLLTEFALVVKILHIWRLRNAAWRYMDELANEPMYAFRRQSEWTKWQRAQRSFAIIANSYTLGSLAVVVFGCISVIMTPADVYILPFNFYIPFEWRHPRKYWYAWTYSSLCMLLTCISNVSLDMIFCYFMFHLSLLYKLIGWRLTALRRPKSTRGRVRDGGGEVESEVIKQMAEIFQMHMKLKRLTTKCEALVSVPVLAQIVLSAFILCFSGYRLQQMDNMENVGILISTIQFASVMILQIFLPCHYGDAVTEYSNALTNDIFNSDWTTFDIPARKFMILYMELLKRPANLKAANFFKIGLPVFAKTMNNAYSIFALLLNMNK

>ZtauOR92a

MNAIERNTNFTRFTAGPVRYFKFIGICLQPPEMRSYKYTRILIVLTILLMFLHQIGYILTPGRTFAEQSTAAGLLNYTTVSGGKILFLIYNRHLLLSSHCQLAALYPSAALERRYKLEHYLRIYARVQTLLYNFFKYILIIYLLYPLVQSCYDLWSNGVYSYLMPTLFWYPVPLEQSLLVYIVYFLFACFCSFCAGLIILSADLCLFSSVSQLMLHLDLLAQRISELQPAEPESLSALKAIIEYHQKILILAHDVNSTFAPSILFSLASSSFILCFSAYQLLDDVSFIFALKVLLLLGYEMKQVVITCYYGDKLMDSSANLFNAVYAHNWVDGSPVYKRLVLFMLVRTYRPIALKVAGISDVSLITLKQVLSTSYQIFTVLKTA

>ZtauOR85c

MSKIIRFEAFLRIPNFFYRSVGVDLWNTDGGPLQDAVFYFGLLNVNVWLLSELVFAVLMVSKNFIQATMTLSYAGFVLVGSIKMYFMWRKKADMTRFLQLMNDIFPRTEQQQKIMHLRRHLRQSTIVMSGFAMMFMVLIWTYNLYPYMQRQIYDRLLDVRSVNKTLPYESYIPWNWHEHWTFYLYYTLQSIAGYHSASAQIASDLVLCAMATQMIMHYEYVAQKITEYQPQVGADSGSKTELSNDKELNSQSFATLEHEAYCKDMQFLCDIIAYHANVLSLSDIMNEVLGVPLLVNFMTSSFVLCFVGFQMTMDAEPDYMVKLFLFLFSSLVQICLICQYGQSLIDSSSNIAHAIYNHDWVHSHVHYQRMLVLVAARAQKPAMLQATSFVRISRGTITDNSTFYHLTISIRLTSPLPNFLRFTGKEGC

>ZtauOR82a

MPEDLFRIQRNCLRLMGHQDIYDDDNEAAGDEQKSKSWQQRCFRHSQTLKYALLLLLMMSAQLPMMDYIIYHIDDLALATACLSIVFTNVLTVIKTSTFLTYKREFKSLMAEFERMYDELHEAGAKRCLVTVNVGAKRFVKLYFYSVSCTGLYFTIKPLVSMIWAKFQVKPLVLELPMPMRFPFDFESTPGYQFAYIYTILITIVVVMHATSVDGLFVSFTTNLRGHFQALQYFIETNTYDKSDALIQRELRIYVHYHVRLLGLSQSVQRIFKPIIFGQFLMTSLQVCVIIYQLVTNMGVIMEMVVYCTFLSSILLQLLIYCYGAEFLKIESSAVGTAVQMSQWYNLPPRHRHVLRLMMLRSQREIIISAGFYEASLANFMSILKAAMSYITFIQSIE

>ZtauOR67d.4

MTAERVRPTDSFAKLIKMVRLISSLVGADVSDVNYRVNIITVILILCIVIYFIFTATTVASVFSEDWTVLLEASCMLGSGLQGCTKLISGIFRTKDVSAMRLELEEIYRTYESKGQSYCKVLNESCDRVRQVIKMVGHIYASNTAGIILLTTVLMLTSDRKIYIMQFFIPGIDADTNFGYLLTTAVHMVVFLAGSFGLFGGDLFFLIYLGQPELFRDILILKVEELNVAAAQKDDNTESLLINIIEWHQYYMDFNNRCNDIFYYIITMQILTSGISIICTMYIILMGDWPGAYLYILVALCGLYLYCIIGTRIQTSSETFFEELYNINWYELDIKKQKMMILILMKSQNPSEIKIAGVLPLSVETALQITKSMYGIFTMMLQVMAEEP

>ZtauOR67d.3

MAVKAIRPTETFTKILNFFHLICSLVGADLSNDNYRVNIITVIVILSIIIYFIFTATTVASVFSEDWTYMLEASCMVGSVLQGCTKLISAFIFKNKICGMRAELERLYAEYEVKGDEYVKTLNKSCERMWQVTKVVGQMYLYAAVGIILTLIYFVIATTQKVYVMHFFIPGIDVNTQTGYLITLGVHAVVFMSGAFGLFAGDLFILLFLTQPMLFVDLLVLKVKALNEAAAQKTNAVQRLLIDIIEWHQYYTDYNKRCNHLFYYIISVQIVTSGISIICTLYIILMGDWPGAYMYILIAFSGLYLYCILGTKIQECNSAFCKELCNINFYDLDIKSREMMVLIIMKAQNPVEIKIGGFLPLSVQTALKITKTIYGLFTMMIRFLEEGQ

>ZtauOR67d.2

MKRQTSPSDIYYKMLFVIRFCSRRIGCDIIAEDYKINLNTMVVIVAIGAYYLCSVHTIMKYIATDWTVLLDVFSPVSCTTQGMVKLISVLLYPELYRKLALDIGLIYEKYQELGAAYKEKLLEWNTNMKKLLIAIAIVYFLTALLILCTPIVLYIFKGERHLILLCQVPGFEVDTFHGYWVNNAFNALCVFIAAFGLYAGDLYLILFLTHSIFFRDILTLKIDDLHKLIEADDKEDRQTKLVKDIVEWHQYYLEFNDKCNLLFFWTISAHIICTTLGILSTLLIAMLKDWPGAYAYLLVCFIWLYMYCILGTRVEINNDRFCTGIYDINWYALDVRNQNTIRLMLMQSQAPKNITIAGVEPLSVSTALKITRTIYSLVMMVLRFQNKQ

>ZtauOR67d.1

METKTRPSDNFHKLLKIIRLSSSLIGVDVIDENYKFNYVIGFVMVAIAWNFMCSIYSIYKDVTTDWTVLLDVFSPISCAAQGTIKLCSLMLYPKLYRELAMDLVEIYKKYQAVGQKYETKLFEWNKSMKNILIIGALVYFVSSLLALVTPIFLYIFKGERHLIIMCQMPYVDVATDHGYFITIGYNLLCVFVAAFGLYGADLYVFLFLTHSIFFYDIFALKVDDLHEILRQDNKDKRIKPLINDIAEWHQYYLDFNDKCNQIFFWSITAHILCTTLGILSTLLIIMLKYWPGAYPYIFVCFVWLYMYCILGTRVETCNDQFCDGIYDINWYDLDISDQKTVSLMLLQSQVPRLITIAGIEPLSVNTALKITRSIYSLVMMVMQFNE

>ZtauOR67c.1

MKPAFKSSEFSPTVPDFVDIPLYLLTFIGAKLFKWTPDDPKSKSRIILLVIFSGLTIYNFSSMIRFILFENLETLLDITECILFWGFALNGLMKGGTLLCFRHEIESILKGLIVRHPKTKEERLEFQLVPYYKTINASNKYLSIWHLSITSIFILHPVLTAIYGYVNREDENEDFDYVLPFMMNYFYDVNHPLPYAISYFMQCCSAYHMSLLFLSGDLLLISMVQLVNMHLGYLIYRIESFQPTGMDADMKVLGPLLEYHNEILDYAERIDSTFSLATLLNYGGSCLVLCLIGLQIVLGSEALSVIKFIGFLVSTIVQVYFVSHFGNNLKDLSTGISDAYYNHPWYTGNYKYMRMLVLPIARSQRYAHLTAFKFFVISMDSFKSLCTLSYQFFTLLRTKIENNGA

>ZtauOR49a

MDFVHFFWFPNALYRVVGYDFQQLARAHWRQVIMKAFLIFTTISGICTRIYMLFQLRELILNGDILNSIRLGVYISYAIDSNVKFFVFLLNAQRLRVIYQTLYNEYPVTPIERKLYQVDKYSFKRAHLMMVVYLSVTNSILLGPMLQSIFMYFVNLFHYGYAVAEFPYLHPTPVLYNFNYCTPHYYILIYISEYLNGHFCTTTNLGADLYVCTFAGQFCMQLEYLGYSLETYEPRVENSKTDCEFLMEWIRKHQLMLDLCCELNEVFGTTLLFKLISNCTVFCAIVVQLKLEGFGIGFFNFLSFFFVTVAQFFMVCQYGQKLITISEDLALCAYKNRWYNGSQTYKILLFNIIARAQKPVKLTARGFQPISLATFQIVMTMTYRVFAVLQRALD

>ZtauOR45a

MFKSDLGIKGYFHLQKFTFRRLGIDMTTREARVTQISFLIIQVFALASILIPIGVYSWQHIDDIAEVSNAMAPFMQATITLWKVWRVIYRRKEMAELCENIYLISAKASKLELTHLIQENNRERLMNTAYYYSVLNTGVMALTAPVVVSFIQYLRLGEFSYITALKATYPIDYARPLNYFLIWLWSAVAIYGVIYVSVPVDSLYSWYIHNLVGNFKILQSKLVSAESIAESTADLGKRRELVYYCVAYHQRLIAMSEQLNIIYQPIVFVQFSLNGLQICFLAYQIGSGVVAMVDLPFLLLFLISVGIQLMIYCYGGQHLQNESVNVSKSIYQTINSSPWPNELRKVLLISLMRAQKPCKLTGIFFDVDLPLFLAVWRTAGSYVTLLRSVDQKTM

>ZtauOR42a

MSKLPPQLTAVMAEDVKTTQRNTIVKRNPLSVLEVKNPVKTLSEETVEEDVVQPASTQDTTKYLFKAAFLMGLVMPSRYRALYVLHSFWVNFLTTFYFPIGFTLIFFTLSDEINISNLLTSLQVTFDVYGGSTKFVVMFFMLEKLRATQVITQQLDKRCRAADEVAELQKMVRFGQKVVIFYLTIFLCYSGSTFMASFFSGYPPYSLYFPFLKWRRSHSEFIIASFLEFVIMDLACLQQTVNDGYPVIYINMMLCHMKILQLRVQKLGTNTALTLEEHLSELKLCIKDHQLLIELYDIISPIVSVTLFIQFTLSAVCIGTTLINIVIFANEFQTQVACCFFILAVLIEIYPACYFSQCLIDESDNLSDVIFHSKWVEQSKEYRKLMIFFLQRSQRPMFLTAGKLFPVTLSSFVSIAKFSFSLYTFIEKMNLKERLGIE

>ZtauOR19a

MSLIINSWDAFKYHWRVWDLSGFRGPRRQSIWYIPHKLYMIVITLLFPIYYPTCFTVESLLADNLNDFCEVIYIAMADVTLNIKFLTLFIVRQQLLELRPILKRLDARAKTEEEIGVLQDGIDSAKKCFLIILRLFYSAFVTSQLMVIFSGEARLMYPAWYPFNYKATRTKFWIAYGYQTIGFLVQCTQACSVDTYPQAYMRVLTAHIRALSLRIEAIGRKSFNCDSSEFIPLTKDEMKHNYDELVSCIKDHKTIIELFSTIQKPISGTAMAQFVCTGVAQCTIGVYMLYVGFNISIMLNMAVFFVSVTMETLILCYYGDMFSQECEQLSKSIYNCNWTVQSSEFKKALCFFLLRSQRVNVLMAGNWIPVRLPTFVMVVKSSYSIFTLLSSFK

>ZtauOR10a

MNFRFLSRTFPLRDYYFYVPKLCLGALGFWPLDTCEPGASNVWAWVNLIILAIGVVTEMHAGCSALRTDLELALDTLCPAGTSAVTLLKMALIYYYRQDLAWVLKRMRDLVYERDVSINTVKKHIVRAHAVMAARLNFIPFVMGFITCTSYNLKPLLMTLILYVQGQEPMWKLPFNMTMPPFLLRAPYFPLTYIFTAYTGYITIFMYGGCDAFYYEFCSNTAALLELLQNDLKSILSFGGDKFTLTAEESTVLEWRLVQFIKRHNDIIELTRFFCKRYTVITLAHFVSAGLVIGASIFDLMTFTGFGIVIYIGYTIAVLGQLFIYCYGGSMVAESSVQLATVAFACDWHACNPRLRRYVLMIIIRSQRAISMSVPFFSPSLVTFTSILQTSGSIIALASSFK

>ZtauOR7a.8

MFELVTGRGTGNATSKDAFVYFFKGCTIVGISPPKNAGPLYYMWSFIVNLICIITSPITGTVGFVIKYLQDIITTVQFLSGLQAGLNLIGIPVKCATVTFALKRLRGMEPTLAIMDARYTRPEDVALIRNAAVMGNRLVLFFGTSYFLYMLFTVLPPLINGNPPLSVWIPFVDENQSTLHFCVQIFYDLFIMFFVLFHQALYDSYGAVYIYVISTHFQLLVRRVGNLGTDATKSKDDNMKELVDCVVTHQQILELLATIEPVISTTMFTQFLIISSILCVTMVNMFFFADRSTQLASTLYFLCVLLQTSPCCYFATELKADSEKLPLAIFHCNWVEQDQRFRKVIIYFMHHAQMSVELMAMKLFPINVGTNISLAKFSFTLFTFIKEMGIGQEATN

>ZtauOR7a.7

MFDLIKGRGRTVFASRDAVIYLFNSFRYIGFNPPATYRVPYFMYSAIITFFAVLFSPVIFNVGWLRDRNKLSVMEILTCVQASLNVMAVPLKCITLAMAHKRLRGIEPMVTELDERYTTAEDKAKIKQCAVTGNRLVFGFAVSYFLYETLTVVSALVGGHAPLSLWIPNVDWHRSTWEYWLQVSFDSAVLFFLLYHQVLNDSYPAVYIYIIRTQVQLLASRVEKLGYDEQKSVDENYQELLECIVIHQKILKIVSIVESVVSITVFTQFLVAALILGVTMINIFIFADLTTKIASSTYFFCVLLQTSPTCYHASYLLADCDELRIAIFHCNWIAQNKRFNNLLIYFLHRSQDSIPFFALKLVPINLATNLSIAKFSFTLFTFIQEMGLGENLKG

>ZtauOR7a.6

MPNLIRVGGACIYKSRDALTYLFKIFTFVGINPSEQQSRKYYWLYYSYSLTVNFICCLFSPLSFHIGYIKLWHVLNNNQLLAAIQNAVQVTGIPIKILVITWYMKRLRQAFKILDQLDVNYTQHEDLAKIRECVRRCRKIVLIFCLPYYSFELSTIALGLLQKRAPLAAWVPFLDGQRAAWEYWTIVLWDTFVMFILLSYQLGGDTYPLIFLNIIRTHVQLLVTRVSRLGRDGALSADEHYAELLACIRTHVQIVSIANIVAPVISVTLFTQFATTATTLLTWFGNVEYPENIISFAFFSCQLLQIFPCCSSASQLIADCERLPDAIFHCNWVDQDRRFRRAILFFLQRTQKPMRFWCLKLFVVKLETSVAIGKFAFSLYTFIQESDVGRKSNN

>ZtauOR7a.5

MRKITDLFYGRGKDDYDTNESFVLLFHSWSLVGFIPKKPTRISEIISQFICWTCAITSPITYFAGLIATMGDLPITIVLSNLGVAINCVALPLKAIHIKVNIDRLHDVGLIFKRLDARYQRPEDQLEVREAVKVSTRIYAIFFFLYWFYGTASWLAALFAHEYPHGSYFPIIDWLPESVFQFWLHFVFEVFYLQILLQINLTNDAFPGIYIRALRTHIKLLTERVSRLGLNPDLSDQENFEELVDCIVSHQELIQISDTVGSILSLTTFFQFTIYAAILCVCMLNMFVFGDATTKVVTVVYLIPAFWQTIPTCYQASMLESDCAKLPLAIFHCNWLALDKRCHKLIIYFMQRTQEEISFTAIKLFRINLGTNLSIAKFSFTLYTFINEMGFGETLKERLE

>ZtauOR7a.4

MRTITDLLYGRGAAKFESNESFQPIFQSWSMIGITTLKSNRTRDVLHMCFCWFCILLSPFSFYMGYVQTLRTKPITDQLSLLQAIFNVTGLPLKAIFIKISQAHLRTTEAIFVRLDERCQSAESREQIKKCVVLSARIFTVVGSVYHLYGSTTILQAFFTDNYPLQTWLPFTDYIPQPTIKYYSHFMFEVFHIYFLLTVQFTNDVFPAIYIRNLRTHMNLLTERVSRLGSNPEFTDDQNFNELVDCIATHQDLLAVKDIVESVCSVTVFIEFVAVATAHCICMLNFFVFADRFQQMVILSYYLGVIMQIMPVCYQASMIMEDSAKLPDAIFHCNWLAMDKRSRKLIIYFIHRAQENMTFVALKLFKIDMTTNLSIVKFAFTLYTFMNNMGFGQNMKELLE

>ZtauOR7a.3

MQRLSELLYERVESDCETNKPFKLLFYFWTWIGIKSKPRGFLSTLHMVCVWIMFFFTPFLATVGFIRKLKVSTVTECLSSLQAFINAIAASAKALAVLMHFKRIKNVEPIMKDLDERYKKPHERQQISDCVASCTRLYASIWFIYYLYGNMSILIAIVLHKQPFGGWYPFLDVIPNPTVHFYSCFIFETCYMYLLLTAQYLNDLFPTLYMRTIRTHIQLLRERVSQVGADPDMSDEEKHQQLIDCIDIHQQILKVVNIVGSVCSPTIFIQFSVVAIVHCICMVNIFIFADNLNLMITIIYYITVAMEILPTCYEASTLEMESSKLPVSIFHCNWLALDKRARKLIIVFIHRAQVDVTFVAMQMFEINMRTYLSIAKFSFTLYTFVNEMGFGQNIKELME

>ZtauOrco

MQPSKYVGLVADLMPNIRLMKYSGLFMHNFTGGSGLFKKIYSSIHLVLVVVQFLLILVNLALNAEEVNELSGNTITVLFFTHSITKFVYLAVSQKNFYRTLNIWNQVNSHPLFAESDARYHAIALAKMRKLFTLVMLTTVATAVAWTTITFFGESVKFAVDKETNSTITVEIPRLPIKSFYPWNAGVGMFYMISFAFQCYYLLFSMVHSNLCDVLFCSWLIFACEQLQHLKGIMKPLMELSASLDTYRPNSAALFRSLSANSKSELINNEEKEPTDLDISGVYSSKADWGAQFRAPSTLQTFNGMNGTNPNGLTRKQEMMVRSAIKYWVERHKHVVRLVAAIGDTYGGALLLHMLTSTIMLTLLAYQATKVTGVNVYAFTTIGYLGYALAQVFHFCIFGNRLIEESSSVMEAAYSCHWYDGSEEAKTFVQIVCQQCQKAMSISGAKFFTVSLDLFASVLGAVVTYFMVLVQLK

>RpomOR47b-like

LYLILFKMFLYRSSIAEIDSIVNEFDTLHAKYAHALLPKTPSNLRIRQWQRNFFIGEIVLTTGFFILSFLLFAAMSLQPLFSHQTLPFRSKYPFGLDNRDEHPIAFACIYAFQCFCVLYMLVSIVVMDSLGGNCFNQTTLNLRILCENIRNIRDGATEEIIWHELRATVEFHQKIIGLIDRLNDVFYWNYVSQMGASTFMICLTAFEALLAKDQPMVAMKFQMYMFSAFMQLFYWCSMGNRTYYESMEVATAAFQVHTWYTHSPRLQRNLMFMIKRAQRPLEFRAKPFFGFTFASFTSILSSSYSYFALLRTMSD

>RpomOR7a-like

MFELLTGRGIANFRSSDAFFYLFKSYTILGSNPPKDTGPLYYIWSILLNGSIIFILPIVCVVGSIIQYARHDIGTMRFLNGIQAGLNVLGMPPKIIIMALSLNRLRSIEPTLAAMDARYKKPEDVALIRRSALMGNRMISAVFIAYMCYMLFSLMPNVIKGGVPFGFWIPFLDWNRSRIDFLAQSVADVFFFFFSLYYQALNDTYGAVYIYVIRAHLQLLRRRVQQLGTDAEMSSEDKMEELVDCIVTHQQILELVAVVEPVISKTIFTQFLIFASILCVTMINIFIFADLSTQISATVYLLCVLLQTSPCCYYATQLMTESEKLPDDIFHCNWVDQDRCFRKAIIYFMHHSQRSIEIKAMKLFTINMATNVSIAKFSFTLYAFIKQMGIGQNAND

>RpomOR67d-like

MPRLKPERPSDVYDKILLVIKICSRLLGVDVTSKDFKVNSKTAFVMVAIFAYYLCSVYTINKYIATDWTVMLDVFSPVSCNAQGMVKLMSALLYAKLYRKLAVQLGEIYEKYQVMGRRYEEKLVQWNRNMKRILITIGVAYSITALIIVCTPVVLYILKGERHQVLLCEVPGFDINSPHGYWVNNGFNAMCVLIAAFGLYAGDLYLFLYLTHSIFFFDIFKLKVSDLHEVIEQNAHDKRITKMVDDIVEWHQFYLTFNDNCNLLFFWTISAHIVCTTLGILSTLLIIMLKDWPGAYVYILVCFLWLYMYCILGTRIETCNDQFCSGIYDINWYSLDVRNQKAIRLMLMQSQAPRNITIAGIEPLSVSTALKITRTIYSIVMMVLRFQNK

>RpomOR63a-like

MIQQAQGHIPLIAETVTTTLQTTTAIVKLIYFIFMQHRFSVILHKAETHELLQCLEIFKTDMPVKMKLKQEINAIMVTTWLESRRQLIYCVILIVCILSNYFFYAIFINLYHQIQGTPDYVYILPFTGYPMFLDKGMNSFYYALDMFLGACSLHVAGMCAISFNCAFMVFCKHACGIVRVLSLMLQHSTSPLVPAERRVEYLRYCIIQHQRIVNFLDEVNQLFKHIILSQFFHSMAIYGLVLYEINFSLESSKVTLIRMIFYICAAFSGDCMLYVNGQFLVTELEAIPLSCYGCDWYNESAEFKKTLQMIIMRSNKEFCFQISWFGVLSLVTLMGVS

>RpomOR33b-like

MLFVMENVKSGISAFRFHDITWRYVGQSPPSPRYRYIYYLYSLILNIIVTFGYPTHLMIGLIQSESKADIFKNITINLTCLGCTIKTLAFWWRLADVRKIYALIQRLDQQIIQSDDVQFYNSNAFRRTKQVLYFDVCVGLGAAIASEVATLIAGVLGNWRLMYPAYFPFDYERSVLGYIAAHFYQCFGVTVQIFQNIINDSFPPMALAMLAVHVRLLNMRLVRIGQRPKRVREINDAELLQCVEDYKALLDFRAAIQRIASIGTFVQILVTAINMGVVIVYLIFYVNDIFSYVYYLLFLVAMPLEIFPHCYYGTVVEMEFNQVTYAIFSCNWMDQSAAFKKNLLIFGEQSLRKQIIIAGGMFAVNLDTFFATLKRAYSLFALVFQMK

>RpomOR30a-like

MVDLHKSPIYEICYLLQALWMIPVGVCSYNAYVNSFLISMSFGTSMMKDLQQKLENLSEMDDVDALRNIKECIKRHIKIIMFRDDLEELYSAMSLIDVMLYCIVLATMLVYSSMDYDGALVFKGLQLLLVQTSLMYLTFHFSNAFSNESLNIANAAYNTNWMQRSTEFRKCIVLIIARSQRPLQLTAGGLKPLTLEAFVGIMRASYSLFSVLQGTM

>RpomOR49b-like

MYFCEQLYNINLVYMKRVGYLGDPKRALFMLAMPTFCCFATIYRIYLIWHNFDEVVANLFKVSGMITITVRTFVVLSKQKKFLDFFNDIDNWYHKLRSENDEVTMKNLEEYTRKTRRASKTIFGIMIVCIFYISAIQLLTISSSTNKSKKNIKNKSSSRTTRYFFTEYLIEVRLPFVDLYTSPYYEIISVLQALWLTPIVLLSYVSYLCVILISISFGIFLMKDLQQKLGNMHELNELEGLKCIKKCIQQHVLIIKFHRDLEVLFSAGNFVDVSIFCIIPCVIIVYANMEYNLAFMITDVQLVFVVIFSTYIIFWLANSFYIEGLNIAHAAYNCNWVDRGKDFRKYIVVIMAVGQKPLELTAGGLKPVNMQFFLAIVRVSYSIFTVLQGTKKDY

>RpomOrco

MQPSKYVGLVADLMPNIRLMKYSGLFMHNFTGGSGLFKKIYSSMHLVLVLVQFLLILVNMALNADEVNELSGNTITVLFFTHCITKFVYLAVSQKHFYRTLNIWNQVNSHPLFAESDARYHAIALAKMRKLFTLVMLTTVASAVAWTTITFFGESVKFAFDKETNSTITVEIPRLPIKSFYPWNAMSGMFYIISFAYQFYYLLFSMVHSNLCDVLFCSWLIFACEQLQHLKGIMKPLMELSASLDTYRPNSAALFRSLSANSKSELINNEEKEPTDLDISGVYSSKADWGAQFRAPSTLQTFNGVNGTNPNGLTRKQEMMVRSAIKYWVERHKHVVR

>RpomOR10a

MNFKFLSRSFPLCDYYFCVPKLCLGSMGFWPGDTCRQNAANIGARINFFILAIGVVTEIHAGCSVLKSDLELALDTLCPAGTSAVTLLKMGLIYYYRKDLAWVLKRMQVLVYERGVRINPAKKHIIRAHSVVAARLNFIPFVMGFITCTSYNLKPLLITLILYLQDQELMWKLPFNMTMPAFLLHAPYYPLTYIFTAYTGYITIFMYSGCDAFYFEFCSNTAALLKLLQEDLKSIIGYGGDKLSLTAEELTMLEWRLVHFIKRHNEIIELTRYFRKRYTVITLAHFVSAGLVIGASIFDLMTFTGFGIVIYIGYTIAVLGQLFIYCYGGSMVSENSVALATVAFGCDWYACSPKLRRYILMIIIRSQRAISMSVPFFSPSLVTFTSVLQTSGSIIALVSSFK

>RpomOR59a-like

MSAHSPLPSPLALPLPAALDTRSFFKLHWTCFKVLGIIAPTSNAYYFGYSVLLHLLVTFCYPLHLALALFSNTNASANIQNLAVCVTCVACSMKFIIYATKMERIRELEAIMAALDERVNSSRERRYLAQLRKEIRRITVGFLSIYTLVGVTAELRFIFRNEHNLLYPAWFPFDWRASDLKFYAAHLYQIVGISFQLLQNFVNDCFPTMTLVLLSAHIKLLGIRVSQIGYANRSLDENEEELLRCIKDQEQLYSMLNVIQNIISLPMFLQFTVTAFNICLAMAALFFFVDAPFDRLYYFTYFLSMPLQIFPTCYYGTDFQLLFEKLHIEMYSSNWVEQTQKFRKHMILFCERSLKQHTAMAGGIVRIHLDTFVSTCKGAYSLLAVIMKMNE

>RpomOR43a-like

MPTPIEDNPLLSINVKLWKFLSVLLVRDWRRCVAFVAPVCLMNAMQFVYLYQQWGDLATFILNTFFAVSIFNALLRTCLVIRNRDKFEALMQELVTLYDNIQATGDEHAKSVLAEATRSARNISIFNLAASFSDLIVAMGYPLFRDQRIHPFGVALPGIDVTCSPLYEILYVAQLPSPFTLSSMYMPYVSLFASFAMFGKAALQILQNNLRNLCENMQHKSEPELFDTLRANIAYHARIAKYVNDFNELVTYMVLVEFLLFSCVICSLLFCINITNSAAEKMSIVMYIGTMLYVLFTYYWQANGILEMSLLVSDAAYEMQWYNCSQRFKRTLLIFIARTQRPLQIRVGQMSPMTMEVFQSLLNTSYSYFTLLYNLYND

>RpomOR46a

MERAKEIVDSFYKEQFIFFKCLGLFDLPAHYPARVHFLFKMYFWYVAIFWIFLFDASMWIKIISNITDLN

EIIKVFYVCSMAIAVMAKFVHIRIKTSSFLALFARMHDDDLLPVNKFEMETFTQSLQLSCKVRNCYMYLSLTSLSLVFVRQLVSDPGELPLSIYIPMNVENIWFFILAYLFQFIGASLCCFINISFDSLSASFFIYLRGQLNILSNRLENIPTDKDTTQDVINMQLKDCIRHYNKLLDITEIMEDLLSTPMSVQVISSVFVLVANFYAMTFLTDPNDYATFMKFLVYQLCMLTQIFILCYFANEVSIRSSELPFSLYSSEWTHCNRINRRLMLLMMAQFDIPIRIKTINRCYSFNLSAFTSLISSPEIQKDVTTSLLKWKRS

>RpomOR24a-like

MLPKFLLQSYPTEKNLFLVPKFALGVIGFYPELGKSTLMNAWALFNLVILVYGSYAEFAFGLSFLSTDAMRALDALCPVASSIMSVFKVCFIWWHRAEIERLVRRVAELTAEQNSSRKLDCKRKYFAIGTRLSSSVLLFGFLTSTLYTIRAGIVNYSAYVRGDEIPFETPFKMIFPQPLVSMPIFPLTFTLSHWHGYITVAGFAGSDGLFLCFCMYFGTLLKALQYDLSDLLADVDSEQGSNRYAERDIEEGLKKIIARHNEIVDLIDRFSAVIALMTLGQFVSSSLIIGTCVVDLLLFSDYGVFVYVTHTMTVSTELFLYCIGGTMVIECSSELATTVYSSKWYTHTVRVQRMVLLIIIRAQRSLVVKVPFFAPSLPTLTSILRFTDSLIALVKSMI

>RpomOR69a

MPFQVYKLQKFLTYPELAFKWAFFEPFIWSGNRHRHQPTAWYYIKHCIYIFGLFTLVGQIMALFCNLYMPHTTPEAEAYENSESKVFEATALICYFSCCLYKMWNIFWRRKDIGPLLEKFNELFPSVVIQKKLAEASDYKTEPKRGSGGYYRLAYFEEKSRARMRFMTKYFAFAYFYYNMIPILQLIYEILSPNQTVTYKTQSNAWFPWHNHNKHSTFVGFIFNYLVQASAEFAGINFIMCGEYLFGFFNTQMQLHFDYLAIALETLDATAPDAMKQLKTLISYHANLLRLFNEINSIFNFTFALDLINATFAISLMGLAMVMIEFGRAVMFSAGFSFFLILSYVFCKNGDELTDVSNKLSSASFYSNWYEGSLEYRRMIIFFIMRTNKPCEYQAYGYTAISMVTYMRMLKLSYQLFTSFRAIE

>RpomOR42b-like

MLDLLRGRGLDACKSRDALTYIFNIFTLVGTNPQGQRSNKYYYLYYLYSVTVNFICCFFSPLSFHIGYIKYWNVLSTTELLAAIQNAVQVTGIPVKIIAITWHMKSLLSAREILDELDANYRRADDLLRIRQCVQRCCKIIVIFCVPYYSFEITTIAFGVLQHRVPLAAWVPYLDAQAGAEWEYWTIVVWDIFVMFILLAHQLGSDTYPPVYISIIRTHMQLLVERAKRLGGDRKLNADENYAELLACIRTHCQILRLARIVAPVISITLFTQFATTAITVLNWFGNVEFPENIISFAFFSCQLVQILPCCYCASHLIDDCDQLPNAIFHSNWMDQNRRYRKTILFFIQRTQNPIRFWCLKLFGVNLATGMAIGKFAFSLYTFIKESKVGDKLDK

>RpomOR67c-like

MPLLARTFSEFIHIPIVFYKTIGEDLYEHRSSNRVRRLLLKLLLHIGFVNFNLLVMGEIIFFVKALRSSATILEATGVAPCIGFSFVANFKQIAMTVHRVTLRQHFDQMEEIFPKTVKQQNAYKLPQRERVMRRVMAVFTLLCLAYTSTFSIYPALKASVQYWLLDAPVFERGFGFAIWYPYKTTEKNWVYWLTYLGQVHGAYLAGVAFLSADLVLVASVTQLCMHFDFISRCLEEFGGASQSDVQKDLEYLKALIVKHAKCLKLSEHVNSIFSFSLLLNFLTASLTICFIGFQVTASSKEDIVKYIIFLTASLVQVFVVCYYGDELMTASMRVGDAAYNQNWFDCDKRYKRLLTILIMRSQKPASIRAPFMPPISFRAYMKVISMSYQFFALLRTSMERKGTKFT

>RpomOR24a

MLPKFLLQSYPTEKNLFLVPKFALGVIGFYPQLGKSTLMNAWALFNLIILVYGSYAEFAFGLSFLSTDAMRALDALCPVASSIMSVFKVCFIWWHRAEIERLVHRVAELTAEQNSPRKLACKRRYFAIGTRLSSSVLLFGFLTSTLYTIRAGIVNYSAHVRGDEIPFETPFKMIFPQPLVSMPIFPLTFTLSHWHGYITVAGFAGADGLFLCFCMYFGTLLKALQYDLSDLLADVDSEQGSNRYAEREIEEGLKKIIARHNEIVDLIDRFSAVMALMTLGQFVSSSLIIGTCVVDLLLFSDYGVFVYVTHTIAVSTELFLYCIGGTVIIEFSSELATTVYSSKWYTHSVRVQRMVLLIIIRAQRSLVVKVPFFAPSLPTLTSILRFTGSLIALVKSMI

>RpomOR2a-like

MAARVNSWDAFKYHWRVWDLSGFRGPKTSSPWYIPYKVYTIVITILFPIYYPICFTVKSMLADNLNDFCEVIYIAMSVVTLNVKFLTLFIVRQKLLELGPILKQLDARAKSDEEINVLQKGIDSANKCFLTVLQLFYSAFVTSQLMVIFSAERRLMYPAWYPFEYKASWTMFWIAYGYQTIGFLVQCTQACSVDTYPQAYIRVLTAHMRALSIRIQRIGFQTKSCDSPDTSHLTKAEMKHNYAELVSCIKDHKTIIELFSTIQKTLSGTSLAQFICTGLAQCTIGVYMLYVGFNLSIMLNMAVFFIAVTIETLILCYYGDLFCQECEELSNAIYNCNWTVQSNKFKKALCTFLLRSQRLNVLMAGNWIPVKLPTFVMVSNLCYIKVGMAKKNNIIG

>RpomOR88a

MGLTSGSTKTDKKLCTIDDLCAIYHPLQSYMGLNFMDYKRINGRFAIPSSKLLQVGLVLAAYDCIGNAIKCGAAIASGNVTLAQEIFAVFGMAVVMTMRGFSLELNRNKLSKLYNDLDRIFPRSAFLQQRMEVEKCHRYIKRRFFFFHNFVSAELVPFCTIPLIKFMYAYDFEEKGPVADEFHLNASWMPFGVKENLSVYPFIYAYETILALIAVNMIITWDQVFAVTISHLCMYYQYLAKLLEEIDVREANDPQQRQAFFKQLHHYIYTHQCLNRIASDLNDIFNLSILISDMCIAASICFNLFLVSDASDYLAVATYISPCLTEMWLLYDVAKWGTLLETVTSRINEVLYEQPWYESSLQFEKYTMMWMQGTHEPIRLTAFNIFNVNMKHFQDMMMLAYQMLTFMKSKS

>RpomOR59a

MLNMSILVTVCYPLHLALALFRNSSLAGDIKNLAVCVTCIACSLKFVIYTRKLRIVRAIEQTFAALDARVQSELERKYYFTTMRRSVRNILYLFLCLYAAVGVTAELAFLLREERSLLYPARFPFDWRASKRNFFAANIYQIVGIFYQLLQNFVDDSFPPITSCLLAGHIKLLGMRIARIGYDAADVEEHEKELVQCIKDQKNLYRLFDLLQEVISLPMLIQFTVTAFNICVAMIVLLFYIDTPLERLYYLTYFLAMPLQIFPICYYGSSLQQLFGQLQYEVFRCNWPDQTRRFKREMILFTERALKETTAMAGGMIRIHLDTFFSTLKGAYSLFAVIMKAK

>RpomOR85b-like

MNAIQRKSNFICFTALPVKYFKFIGICLQPPEMLSIKCARVVTVVSIILMFLHQFVYLFTPELTFSERIEAIGLLNYTNVAIGKIFSLIYNRGLLLKNYCELERIYPSVAVERHYKLGRYLRIYARVKSFLHTFFVYILIVYLLFPIAQSFYDLFSTGAYTYRMPAKFWYPLSVEESLLAYLFYISLQCFACVCAAIVILSSDLCLFNSVAQLLLHLDMLAQRILELQPAEKGSMRALKAIIEYHQTILLLAQDVNDIFAPSIIFSLASSSFILCFSAYQLLGDVSFVFGAKVLLLLGYEMKQVVITCYYGDKLIESSSRVFDVVYAHDWTVGSPAYKRLALIMMLRTHKPIALNVAGIADVSLNTLKQVLSTAYQVFAVLKTA

>RpomOR82a-like

MPEDLFRIQRNCLRLMGHQDISDNNELPPRAEQKSRHSLCTRQCQIMKHALLLLFMISAQWPMMDYIIYHIDDLPLATACLSILFTNVLTVIKTSTFLAYKHEFNQLMSEFEMMYDELHEANAKRLLVAANVGAKRFVKLYFYSVSCTGVYFTINPLVGMIWDKLQGKPLLLELPMPMRFRFDFESTPGYQLAYAYTILITIVVVIHATSVDGLFVSFTTNLRGHFQALQHFIETNTFDKSDALLQQELRAYVQYHVRLLSLAKSVQRIFKPIIFGQFLMTSLEVCVIIYQLVTHIGVFMEMVVYCTFLSSILMQLLIYCYGAEFLKIESSAVGTAVQMSQWYNLPPRHRHVLRLILLRSQREIIIRAGFYEASLANFMSILKAAMSYITFIQSIE

>RpomOR82a

MPEDLFRIQRNCLRLMGHQDISDNNELPPRAEQKSRHSLCTRQCQIMKHALLLLFMISAQWPMMDYIIYHIDDLPLATACLSILFTNVLTVIKTSTFLAYKHEFNQLMSEFEMMYDELHEANAKRLLVAANVGAKRFVKLYFYSVSCTGVYFTINPLVGMIWDKLQGKPLLLELPMPMRFRFDFESTPGYQLAYAYTILITIVVVIHATSVDGLFVSFTTNLRGHFQALQHFIETNTFDKSDALLQQELRAYVQYHVRLLSLAKSVQRIFKPIIFGQFLMTSLEVCVIIYQLVTHIGVFMEMVVYCTFLSSILMQLLIYCYGAEFLKIESSAVGTAVQMSQWYNLPPRHRHVLRLILLRSQREIIIRAGFYEASLANFMSILKAAMSYITFIQSIE

>RpomOR49a

MDSVDFGQVFWGPNALFRAVGYDFQRLPRPYWRQILMKAVLVFMILSAICIRIYMFMSLRELVIRDDILNSFRLGAFIAYGIDSNVKFAYFIFNAHRLRQIYDFLAAEYPQTASEQKLYKIDIYGFQRAPVMICAYMAVVASIMLSPLLQSIVTYIIDIYRFGYDAAEYPYLHPIPMPYNFDYCTPRYYIPVYMVESLNGHFSSTTNLGTDLFISIFSGQLCMQLEYLGYSLETYQPSMEKSEEDCDFLRKWIRKHQLMLGLCADLDEVFGTTLLCKLITNCTYFCIIVAQLMLEGYGYGFLNFGSFFFLTVAQFFMVCQYGQNLITISEHLSFSAYKNRWYNGSKAYKKMILTIITRAQTPANLTAKGFQPISLLTFQIVMSVTYRVFAVLQQVFD

>RpomOR94a

MLTKNSPNSSTVISTTRGDRIASVRSLIIVLKVIGLWQWPEDRTQWERTQFLLKLQRVYGLALHLPLTFTLITLMVCAALLSHDLEEISNVLYILLTEFALVVKIFSIWQHGTLAWRYLDELVHSSKYEFRQQAELTQWMREQRLFVIVAYSYIFGSCSIVVFSCFGALYTAADVYVLPYAYYVPFEWRDPHNYWYAWSYCCISVSLTCVANVTLDMIFCYFMLHLSMLYKLIGWRLAALRQRRDAAGAAEVLNEMRHIFQLHESVNRLAAQCETLVSVPVLSQIILSAFILCFSGYRLQHLQIMENVSVFFATILFVSSMTLQIFLPCYYGNMVTVNSNALTNDMFNSDWTAFEIPARKFMILYMELLKRPTTLKAGNFFLIGLPIFTKTMNNAYSFFALLLKMNK

>RpomOR83c

CKALQECCERVWQVIKMVGNIYAATAVGILLLTAILVIVTEKQVYVMHFFIPGLDVGTQFGYLATMAVHTVVFLAGIFGLFAGDLFFLIYLGQPELFRDFLVLKVKALNEAAAEKSKTAEKLLVDIIEWHQYYTDYNERCNYVFYYIITMQIVTSGISIICTLYILLLGDWPGAYLYILVAFCGLYLYCIIGTKIETCNHAFYEELYNINWYDLEVKNQKMIIFILMRSQNPSEIKIGGVLPLSVQTALSITKSIYGIFTMMLGFLDEEQ

>CstyOR115

MAGKYSERFFNIFYLTRRFSELCGADVIKNDYKICWKTGAIFLLINFAIAFTFYTNYVEVIVNGNYYNLLKSASILGTGLQGYTKLINILLQQKSLRFLYQEITEIYEIYELKSTAYKDCLRYSITLVKKLLSTLLTLIVITTLIIIGIPVFMLIFLDTRIEIMPFKIPYIDIETDIGYYVTFVVHTISVFFGGFGNFVIDSWLFIFAAHVPLIKNILKCKFDELDKILEANPKDVEKSRAPLKDIFEWHQKYMLFCKIIKEAFFWVIFVQVGTEFFGIISTIVCIFLGIWPPAPAYLLYLFAMFYSYCSLGNIVEVSNDDVTLIIYDSCWYNLTASEQKMVLIMLRESQQATGISIGGVSPLSMSTALQLTKTVYTLSMMLKEFLN

>CstyOR111

SSLLYIVAHVYQLAAVSILILQDIASDSFAAMNLTLLSGQLRTLSMRVTKLGGDKTKAKTQNNKELLECIQDHKDLLQYRHKLEEVISFYMFFQILFTSINMCSTIVFLILFANDPFTWIYYTVYFLSMAAEIMPVCYYGTIIEIEFQNITYAIFSSNWLDQDATFKKHMRIFAEATKKPLCIMAWLFHINLSTFVFACKNAYSMFALIMNMK

>CstyOR110

YTNNPSTSKEMPLTKAKQNLEFLTTQHLNLLKMGIDINTLKRRELFNNYWKFLWLISCTIYLEYALINFVAHSLSDIDEATGALSIFNQGCLILIKVSMFLAKGDRFLKLIWDMNLLAEKANPEEHKKWLEENRRSQLIGKMYFYACCVAVACASVVPLLFMAYDYQQNSGFNKKLPFGGKFPFDKGGVTAFAINYMLSLIYIYSLLNMTVGIDTLYGWYIYSISAHFRILRCKLESTARKLQNNDCSDFTRDIGLIVNYHNATLQFVEGLNTNFGEILWAEVMLSCLQMCFVAYTLNNDTDVSNMPFNVMVLVAVMMQMIIYCFGGEKIKNESLMLSSEFYLNFPWHKMSAQQKKLMLLPLLRSQKLSVLRGLFFEVDRNLLVYILKTSFSFNALLSAMKE

>CstyOR107

MVLDKTIPHIGKFFAIPLNLFFILGFCIIRWKPNEKPKELQYLFILFLVFNSVYNVAGMLSYTIYEPLETSLEKTAYIIYTTFAGNSVMKFVCCLCNLKKLHQCFKSLEKYYPRTAKEREDYRLDEHLKKMERFNLLLTIYHFLVTSIFSWFPLIQSTVLYYKNEERSFPYMLPFPMHYIFNERTNLGYAFAYTTQCTGSYAASCMCQGADILLLTCVHLINMNLTHLAKTIRDFKPTGTLSDLKQLKQFVTYHNDILSTVNLIDDTFSLSILLNYLCTVTIMCLIGFQMVIGTNIFHLLKFLLFFMSVLTPVYFISKFGTDMMELSSDINEAFMHHSWYDGHILYQRSLIMSIRISQKPVHLNAFKFFIISMETFKSLISISYQFFTMIKTSYVEE

>CstyOR105

MTTKYSHDFAKLFNFTRNFSRMCGADLIREDYKMTALTWFIIAIVNGAIAFTFYTMYVGVAVNHDWTELLKCLCMFGTGVQGYAKLVNGLLRPEMVRFIYRTITGMYSVYELKTANYHKLLKQSVSLVRKLIIILLSSVILVCLAIIGTPVIYKIIFNERIFIMPFFFPYIDYNTDFGYYLTSVFHVICVIFGIFGNFVSDSWCFAFAAHIPLMKNILQAKFNELDELLQENDESKDLKQVAEKLFEIFKWHQKYQEFCNTVKELFFWVIFVQVATEFVSIVCTIVCIFLSIWPAAPVYLVYSFVLFYLHCALGNLVEISNDDVILMIYDCCWYNLSVPEQKMLLIMLRESQQADGMSIGGIAPLSMSTALQLTKTFYTFSMMLRQFLN

>CstyOR104

KDFENTATICSGWQKQPSFILRSCKQMDPSTITNSKYSKQFEKVFNFTRFFSEICGADVVSEDYRMTWVTWSLIGLVNGAIVFTFYTMYVGVAINNDWSEILKCLCMFGTGIQAYAKLINGLTRKDKFCFLTKEINGIYSTYELKSKNYHKLLEQSISLVKKLITILLSIVVLVSLAIIGTPIFYSIVFKERIFIMPFLFPYIDYETDFGYYLTSAFHVVCVFFGAFGNFVSDSWCFVFATHIPLIKNVLQAKFEELDEMLEEEPQDASKVMDLLMDIFKWHQKYIVFSNTVKELFFWVIFVQVSMEFVSIVCTIVCIFLGIWPAAPVFLIYSFVLFYFHCALGNLVETSNDDVTLMIYDSCWYNLNVSQQKMILIMLRESQQAEGMSIGGVAPLSMSTALQLTKTIYTFSMMFREFLN

>CstyOR102

MMLNTEAEVETWQAFKNHWILWKFFGLQPPKRDSKWFKPYIAYAIFLNVTVTLLFPTTLIVNLILSKNLTELCENLYMTTTDVICNIKFLNVFVMRHKLLKVRNILQRLDARAKTHKEVAILEEGLKLARKCFMTFARMFCCAIISSQMMVYLSSERILMYPAWYPWDWKASKRNFLYAHSYQLYGLVLQAIQNLGNDTYPPTYLIILTAHIKALASRIKDLGTNETTTDEDLYKELTDCINDHNTINELFLNIQEIISPTCIAQFMATGLAQCTIGVYLIYVGLHPSKTLNIIIYFSAVTMEIFILCYFGDLYCQANAHLTESIYDCNWMDRDKKFKQAFLVLLQRSQKNSSIMAGSLIPVRMPTFVSVMKTAYSVFTVLNKVN

>CstyOR85b

KEEVSKRQALLASVIFYSGVINMNYVLLSEILYVIMALVKGENFLEATMTMSYIGFVLVGNFKMFFVYRRKDDLTKFVHGLQRIFPDTPELQVEYNMPHYLKQCSRITMSFSWLYMILIWTYNLFSIIQYVVYELWLNIRQVGQTLPYFMYISWNWQNHWSYYLLYAIQDFAGYTSAAGQIAGDLLLTACATQLIMHYDFISYKLASYQVQRSLEGVDKELAYCQDMQFLKNIIQYHTNLLHLTDQLNDVFGKPLLLNFAASSFVICFVGFQMTIGATPETILKLLLFLFSSITQVYLICHYGQHLIDSSTNISNAVYNQNWTAADVRYKKMLILIAKRAQKPAILKATSFVLISRGTMTEIMQISYKFFALLR

>CstyOR82a

MIDLFGRQRQCLRIMGHHFVRDKSLLLRKWRNIVYFGVLMLVMSAQWPMINYAIYYIDNLELATASLSICFTNVLTVIKISTFLSYKWRFVALMTKLETMYQESKDPAAKKILKTANRSALMMVKLYWMSVCSTGMYFMMSPVLKILWSQIHKTEMVLDLPMPMRFAFDIESFPGYEIAYIYTGLVTLAVVMYAVATDGLFVSFAINLVSHLKILQKSIEENTFLKSDEELHGDLKSYIEYHNLILSLYNELREIYSPIVFGQFLMTSLQVCVIVYQMVTHMDTILVLIINITFLTSILLQLFIYCYGGEILKLESLMVGISVQISNWYNLKPSHRRMLVLLMLRSQREAIIKAGFYEASLANFMAILKAALSYITLIQSIE

>CstyOR74a

MFNILYRPRLPNGKHVPLNWSLKLYRWTNIICWPLEDNAPYWCHLFDRFLWFLGFVTFVVHNDAELRYLSVYFNNLDEMLTGVPTYLVLIELHLRAFSLGWRKDDFKRLLKKFYAEIYIEESMNSQLFKKIQRQIWPILTFSLLYFLALNSYIVTGVYVLSTNKRELLYKMIPPVDYKNNFYIFFPLLMSNIWVGFIVTTMMFGEGNTLGILIFNLNGRYIMMREAFNNKVETLLRSNLNSNIVEKYERVLTETLKENIRLNKFAREIQDEFSFRIFVMFSFSAISLCALGFKVYTSPVNSIGYAFWAIGKIQEILAFGQLGSTIISTTDQLSSMYYESKWETIIERSSHSPDNVNLMKLVTLSIVTNRNPFHFTGLNFFNVSLVSVVAILQGAGSYFTFLISLR

>CstyOR63a

KEYDIICAKFKSFYTMSQMLKDILKSQEIVNDKILSTFYKISFMTGVNVKYKTGFKDPVKLVNALLVSVSLVGLCAQYCLVWHNRKESFVESADAICTANQAWISVFKLIYFIFVQHEFYDLLHTATDGSLLYELGIFDLAINCKQKLLQEIKDILEDSWLHIKHQLNFFTFSCMMACGFYMFSCIFANYYYTHIQPQNFTLQLPMPALFPMWHDYGMTLPYYPIQYIIAGIENYICGMCAVCFDGIFIIIVVHCSALFEVLHKLLEYATDEDIPQSERVKYLICCVRLHEQIYRYYLKINSMYRNPSLAQCLLSMLVLCVVMFMANVGLEEDITLFFKMLCFLGAAGFQIVIYCYNGQKIITQSEKTPSLWYISSWYNESKQFKYIINMMILRTNRTLYLQVSGFTTMSHMTLLSIVQTSGSYFLLLKNLSGMD

>CstyOR59a.2

MSTANTEILSKPSSINCREFFIINWKSWKLLGIIMPQRDARNRLLHIFWNIFINITVTCMFPIHLMLGIFLVKATKSELFENVSIFITSAGAALKLIIFASNVKRIIKMEQILQTLDERIQHIDDQLYYTQHIKRHLIYVQRMYIVVYLAVGFFASLAFIASGEQKLFYPGWMPFNFHQSWWHYSAALGYQLHGIFFQIMHNFANDSFSPKALCALSGHIQLLYKRVARIGYDPSLTSQENERELNRCVTHQKDLYELFDAIQEIISWPIFCQLFVSLANLCVAMVAVLFFVTDIFYRIYYVMYFFGMIMQLFPVCYYGSDFVILFEKLHYAVFSCNWTGQSKRFKRHMILFTERTLKMSMAMAGGMFPIHLTTFFATCKGAYSMFAVVITMK

>CstyOR59a.1

MTTTKVNVVQQHAEPNSCAFFKPHWLCWKILGITLHIDNSHRHRDIYMLYSIILNILVTICYPLHLALQLFRSDSMADNIKNLAVCVTCVACSTKFIIYSTKLSTIWQFEQILERLDARITDDVETNYFRRMRNRLRNVGIVFLSVYLPVGITAELSFMFREERSLLYPAWFPFNWIESTGWFYVANVYQIVGIFFLLLQNYADDTFPPMALCMLSGHIKILSIRVASIGYDQNSLHQNEEELNRCVEDQLNLYELYTTIENIISWPMFIQFCVTATNICVAMAALLFYVSAPLDILYYFVYFLAMPLQIFPACYYGSDFQCLFDQLHLAIFASNWTTQMLKYKKHMLLFTERSLKQNVALAGGMVRIHLDTFFTTCQGAYSLFAIIMRMK

>CstyOR49a

LKSVNKMSPKSANPLVAQKDFQDFCNLPNVLLSRIGYDFQNKPRPIWLVVLGKCYFLFASISHLYIFMYIAKATYSMIISADFELSLLLRLISGFNYAVFSTAKFITFHWHRKELKFIYETLKEIFPKTRKEKLIYRVRDNFWPKWILFIVYFYLGAVAFIATSPLMEGIVLYLGNVSKVGWRRAEFGYFKLYEIEYSFDHRSAFSYLITYSMELMHAHIMVTCNICADVWLLCLALQLCMHFDYVARTLEAYEPDEKEFVKDQEFIAELIKRHQILLNIGDGLHSVFGVLLLLVLMATAATLCCAGIYAITQDLGREFIEYAAFLPITIGQYYLICFYGQQLIIRSNSVADAAYNHSWYNGSKSYKKSIFIIIMRSQKEVELNALGLQPICLEAFKMLMGATYRVFALMKETML

>CstyOR47b

LKPFKLNNMTKVAIQKFTNYQQDEEDEEYGIKNASVDTILAAYKDLRKVLFQRQAPDKISLVYMRNYMRLLGILPRTWRGETLSYRIINKFIMIMLASFAVSITFDLYDAAQDVLQFGEDLVVLIGIYLIFLKLVLCAYHAQDIEQIIREFSKMHQYFGQLKHSSNIGKIRKLQRLFYMAELISFFLYISLGILFTAAICLPPILTPNGTPYRAKYPFEWQTYSDHPLRFTSIYLFQCIMTQFVLLAIVVIDNMGCHIFTQTTLNLQIFCMRIREMGSQPLGDRDLLEELHKAIQFHQYIIDLIAKINDVYYYNYAAQMAASTFMICLTAFEAMLAQDQPMLAIKFQIYMFSAFAQLFYWCCTGNMVYYNSLDVADAAYEIDRWYDQSKEFKYCLRFLIQRAQSPLVFRPKPMFCFNFESFSSILSTSYSYFALLRTMND

>CstyOR10a

KYILKLCAKPTRKMPLQFLSSNYPLHDYYFYVPNFCLRVMGFWPQAPNTQTKRLWASSNFLMLLIGVLTEMHAGLTALTYNLEKGLDTLCPAGTSAVTLLKMILISYYRQDLQYVLKKMQTMLYGESTNRQILQQHKKIIRQFSVLAARFNFAPFLTGFITNTAYILKPLIMAWIFWSKGKEIQWTTPFNMTMPSILLRAPLFPLAYIFTAYTGFLTIFMFAGCDAFYFEFCSHIAALLKMLQADIVSLFAVFENKLILTEEENKYVENRLKIIISRHNEIIDLTHFFRKRYAVITLAHFVSAALVIGASIFDLMTYTGFGRILYVAYTMAALCQLMVYCYGGSMVAENSVQISTVIFGCNWFICNPQVRRMILLIMIRSQRTLTMSVPFFSPSLATFASILQTSGSIIALASSFQ

>CstyOR2a

MVLTSNNPDTNLAFYYHWKVWHWVGIKAPQESNLQLYRVYAVLINSLVTFLFPLTLIVNVFFAQNTQQLCENLTITITDTIANLKFVNVYLVRGELERIKAILGKLDKRAKNTEEQKILKSAIRISQLSFLIFVRLYTVGTCLSILKVIFAAERCLLYPAWFGLNWFDNTFIYILVMTYQLFGLIVQALQNCANDSYPPAYLIILTAQMKALEVRVRAIGRAENGEERMCLTKEEYLRNLNEFNECIKDYKNILKLFTIIENIISKACLAQFVCSALVQCTVGLHFLYVVDAADYGAQILSIIFFVAVTLEVFIICYFGHCMSTQSWNLTYAFYSCGWLAQTPCFKKNLLITLMRTQRHSIIYAGSYIPVDLPTFVQLMKYAYSTFTLLIRFK

>CstyOrco

MQSNLQPTKYVGLVADLMPNIKLMKYSGLFMHAFTGGSPLLKKVYSSIHLVLILAQFIFILVNMALNADEVNELSGNTITALFFTHCVTKFIYLAVNQKNFYRTLNIWNQVNSHPLFAESDARYHSIALAKMRKLFFLVMLTTVASAVAWITITFFGESVKFAFDKETNSSITVEIPRLPIKSFYPWDASQGMFYTISFALQGYYLLFSMVHSNLCDVLFCSWLIFACEQLQHLKGIMKPLMELSASLDTYRPNSAALFRSLSANSKSELIINEEKEPPSDLDMTGIYSTKADWGAQFRAPTTLQTFNGVNGGNPNGLTKKQEMMVRSAIKYWVERHKHVVRLVAAIGDTYGAALLLHMLTSTIKLTLLAYQATKITGVNVYAFTVIGYLGYALAQVFHFCIFGNRLIEESSSVMEAAYSCHWYDGSEEAKTFVQIVCQQCQKAMSISGAKFFTVSLDLFASVLGAVVTYFMVLVQLK

>BcucGR1

MAHRTVQPLLVHFGTLFNFCKMIGLYPHDLQAFRSVHTLQQSKRGALIVMATMFGVVVFYNLLIFFFSGEDHDLKASQSTLTFVIGIFLTYIGLGMMITDQWSALRNQAKVGEIYERIRAVDEQLMKENVIVDNSKTTKSIHFMIVLTVACELIILISTYFALVKYNEWKSILWLFSCFPTLYNSLDKIWFVSTLNGLKHRFTVINTALEDMVVSHERLKRWTENGSGGGEVFRRASIANVSIDPSLEYLYKELTHVEAVKAYNMARNKISPIAHSLNSFGDAMETPKKLQKFTLQPPTFNMVYESELSKDIEKVEEKLNNLCQLHDEICEIGKLLNELWSYPILVLMAYGFLIFTAQLYFLYCATQGQNIPSLFRSAKSAIITTIFLSYTAGKCIYLIYLSWKTSLESKRTGICLHKCGVVADNNLLYEIVNHLSLKLLNHSVDFSACGFFTLDMETLYGVSGGITSYLIILIQFNLAAQQAKDASNAVDANLYAQQFTTASGNTTALMDFFTTTFMPTSQTELY

>BcucGR2

MSDSNGQPPPFLHSFNTIFYLCKLFGIYPYDFQKFYYQGILQGSRLGSCVVMTVMCVTFLLFNLTLFTFGDEEVIARNNHLSVIVTIVFTYITPATMFTDQITAMRNQKRLPELFERIDYVDEDLRQLGISVDNRRVQRGIWLMVAFTFGCEFFIFISSIYLLVDELKWSTALWIFTSLPTLYNTMDKIWFLGILLGLRDRFDAINAELEKIAEELEKRQRQQLKGQQYHESELVLQTSLPLRTEQIGDIKLERLVRDAFGELLHNREPIKSCMLSMSHESSLYTFKALQERFISLCQLHDSTCRIAKLLNELWSYPILILMAFGFVIVTSQLYFVYCATQKDHVIPLVFRSAKKRTISTVFLAYIGGKCISLMFYSWKTSQASRRAGICLHKCGVAADSNEVYEIVNHLSLKLLNHAINFSACGFFTLDMGTLYAVCGAITSYLIILIQFDMAGQQVRISKELAAANETTAVSLLPIENYTVMPLETTTLWE

>BcucGR3

MKVAAHVTKNWLQRQEVRLQNIKVWQRGRKVGSGVLELKEWSAKPKSGKQRKRQICMLRVRNLFRRGTKKDYEHSGSFLEAIGPVLLLAQFFALMPVCGILSKTASNMYFSWKSVRTCYALLAIFCLGPASLCTITFAFREHFNFDTVEAIVFYVSIFLIALAFFQLARKWPALMVQWESIECQLPPLKTEMQRAALAHRIKMITLVASMCSVVEHLLSMLGIIYYVNACPAIPGHPIQSFLYSNWSQYFYFFDYTDLAGIFGKVLNVISTFAWNFNDIFVMAVSVALSARFRQLNEHMMRVAKRPTTEKFWIENRINYRNLCKLCEATDDTISIITLLCFSNNLFFICGKILKSLQKKPSFSHTMYFWFSLGFLLMRTLMLSLYSAEIHDESKRPLVVFRSVPSGSWCRELKRFSEEVTTDVVALSGMKFFHLTRGLVLSVAGSIVTYELVLLQFNKEDKVNDCYEG

>BcucGR4

MRFWKELFSPNDAIGAAQTLIWFNFLLGLTPFRVQATAGSQERILHISQLGYLNTLLQVIFFMYCFIHSLAEQASIVGFFFKSEISQIGDTLQKFIGLLGMLTLFGISLSECRVVVSLCNTIAAVDRRFHNLGVAFNYQYIMKLTHLKMFLVITLNVSYMSSCFWMLFHNDIWPTFQAMITFFMPHVFLLSVVVLFSTFLFRLQQHFDLINKVLKNLAHQWDNSIVKPMPKQRSLQCLDSFSMYTIVTNNPCEIIQESMEIHHMICDAASTANKYFTYQLLTIISIAFLIIVFDAYYVLETLLGKSAHESKFKTVEFVTFFSCQMILYLIAIISIVEGSNRAIKKSEKTGGIVHSLLNKAKNAELKEKLQQFSLQLLHLKIHFTAAGLFNIDRTLYFTISGALTTYLIILLQFTSNNEPEQQLTTMGPNGTTPLQNLVSNLTADA

>BcucGR5

MCSKTTKTVLRQMRKLVADTCISTQFLFSTALGLFPYKYNTGTRRLTIARWLNYYWPLINIAIALMTLYIYFLKSKMNEIHFISDKPLNKLLAHIHYILGFCTLYVIVITNWCRRKELFRLHNELVQMQRRQQRWQRRWTVKSNNQIEAFYYNCIIAKSVMTLLQEVSSISGKLGINPHPSLKYVLYVVFMFAMKNVTYLTVTNFHFALLNIYRQLQQVNWNFQEVVRLWGERAPNAFDMPFEHVTDIAFGAWPSQSHSAKRRYGRRNFDDSAITDLCRQYVRICGLAKRVCKHYEWQVLLFLAIILFGNVMSTFYFLVYLGGNVLPQELFSPTLFLQIYFINTLDLGFFMVICERSMASSKDTGFLLKKLSQLKSLPPALQHEFEMLSIFMAGETVRFRFCGLLEWNFRTGASYMTATILYLIVLVQFDYYNL

>BcucGR6

MSKLVSRCLEFMNYTAIFVGFTSLRIDYSSQKIYESRLINCYVVIANIITVFLLPGAHALSIKYITVNFKNNLLAFTDLV

NLFIVYTVVVFSVLSRFRRERIYKEISRDIFKLDRSYFNKLTTNARIEKHANVVIFIKMITVCLEQFVPITGILYQAIRV

DIYVWMLALYTSLIESILNAVLFLFFYMLWQVQKRVWRLNAHLKELLHSLQKLHKSAAGMLPPDSSTLQQLSALAEEELSEITGVQARLIAMLLRLNSVYRWQVIVVLLTYLSCNIAYGYYWVVSFNSQISRPQNVPSIIASLGASVIVFIDINLLYWGADAITSACQDTGQILRRFQELPLMSAAFERQCEHFALQLKQQQMNINIAGMFSLNRQTSLALWAFSVRHIVILVQFDYEARKQSNRTNGVLDHINHMLQFGDDYLEL

>BcucGR7

MEITEPTLCIFYVSKVLALAPFSVRRNSKGVLDIRRSVMFSVYSASLCLLMVFLTYQGLLFDANSQVPVRMKSATSKVVTALDVSVVVLACSAGVGCGLWGYRATRELNTRLRKIDDSLHSFSNFKRDRILAILMLALPLIAITSILGLDLSTWLRFAICMRTPTDDTELNVQWYIPFYSLYFILTGLQINFANTAFGLGRRFRRLNVMLRSSFLKDVDNNQKYIPSKPLITTVKVVSQHPLSLHQSLSKLSHLPTQESAKSKVALLRLLEENHESLGKCMRLVSNSHGVAVLFILVSCLLHLVATSYFLFLELLNKKDSGMVWLQVLWIVFHALRLILVVEPCHLATVESKKTIQIVCEIERKIHDPILNEEVKKFWQQLLVVDVEFSASGLCRVNRTLLTSFSSAICTYLVILIQFQNTNG

>BcucGR8

MSFWERHKHNIYKYGHIYATLYGLMVINYIPQRPTNTFTHRLAVIYGHALSVCLIVVLPIYFARNISALTDARDQRGHLLLLVNFANTLLKYITVVVTYVANFAHYAAIRAVTRVRQQLEDDFDRSLRAMPAYDDERPRQQFEAMLLFKFGLINAMMAVQVANILYQHFNGAHPVRVHIAVYTFVLWNYTENMADYFYFINSSALKFYQQLNQQLRQVLRENKLLHYFRLRGQRRGTIPHLCGLLCDRLDTLAQRYQQINRLYQDSLTMHQFQILGLVFITLISNLTNSFILFNLFVKHSEARASPAIVLNALHAIIFYVDTYIVALVSENISLELRNINQTMRQFNQVAALDARLQQTLEGFALYVMNNRVEVRICGLFVLDRSLTYLTAAAALSYFITLVQFDLNLT

>BcucGR9

MKPRKTTNSELQQPVLLLDTFTLLQLRLHQLFGMCAVPLGRQCDLATVNQRPQSAGIWQQRFLLLWHCLHFILFFLVHLWTSVNHDSILYNSDSFGKFNDLLKLIGTIISHFVILTETIIRRRAMEKFLTVYTQLHYKWHGAVGVRAEFQLYRKFFCRSSIFIFVVILIDVAYLREISRNRKWMTFFIPFIPSGLICNLRSVQIMLFMEMLRTEVVHLNGNIERLVLFSERNYRVKFQKRDQFERKICAELQTFMECYQEIYEMFTLLKKAVGMSMTCNYVKDYVMILSECYWSYWMVYNGEHITEYLLIVPTAVSIFLLLITSRNCMRSTNFLAHNIHKIRHDIEDFNISTRLQSFALQMLHQQIIIDGFGFFVLNCNMARDILGSIATYMIFFIQFMPKFKSF

>BcucGR10

MPKYNRAERFAYRTLIVFYNYGRLLSVFSWKLQKHTRKMELQQAKCTNRLLRIIWRTLLTLIFLTLMPKMMAPFLRLSAKGFLLFFANIQVVTVTLFSVISFVIHERSDRKIFHIINQLVTMYERISAKSGVQQLLGRTFVVSVIAKFLLSILGLVYEIPLLLGDKEIVKSLSGIYLWLGTIYTLDCCFLGFLVIRQMYVAMATHLEYMLEEMSAIESEEPRKRLSKYQRIKLLCMYSESIDDSNNIYSILYELTKQFQHIFRWQVLYYIYYNFVIILMLMHRFIWRYFESNFVDIMSFFSSVFKFCNLAFLILSTNGVVEKSQLPDLLNLDLVCSDIDARWDESVETFICQRKVENLEIKVMGFFHLNNEFILVIISAIMSYLFILIQFGLTSKYKYTKNLRN

>BcucGR11

MYYRKIFASFARYHLLALMYLLLLQPVPRLPWVTSICRLIWLAWLTIPGHTILYSLLTDSFEKLGHIVGSVLCTFKMLTNFVTYVESMCKAKHYQQLRRLEDEVDAMLRNHIDVTATFERKRWLRVFYCTLIQITYDILQLVVIFKDYMSPVFYYSMPMLLIERARYAQITFTIERQNERSLSLTALLRVLVKANRPKNKYTSDVWQPYASWEYENLNSVRLLQGRLCELYQCVGTCYGWSIIVLLFTTFFTAVANIFWCIEIVSSDFRLGQFMYDALTMLRLTTLAVVLLITADKARQHNIQISGLIFKLAKPIGNKTYNDLVSNFSLQCLQQRLFINVKGFFTLNLPLLGDSVREETELEEDDSNEIRKRNFDIEKEHCVGLITVYTKPDEHGDA

>BcucGR12

MCKQVPFVVRFHRRLFQIIGCCDVSWHTQPRENRLAEQRLVAWTILILLIYTFTFINTFVHPAEFLFTSESFGYFVDALKVTMAYVTVVIIYMETVLRRQALQHFWQRYAVLNAAISQKVQSTKIDWRTQLRAYQRFLYVFYGITVFDFILEIVYYVMREKNDHMLQFMLMFTPYTYMIHLRNMQIIYHIVIINHELVKLRHDVSLLAEYTRFTRTVMPFEGFEGFVRQKLAEKQLQYQRIYEMCDYFQQSFGISAIAVLLFTYVRLVVDAYFTLYSYHITNRPEVIENLLLLPAYLEIPALLLTSQKCMNEVKFIAFELHNIRSSVDNSLISIQIQNFSLQILHQKIRIDGLGISALDGKMLVSVCLQKNINKLMSFKCKCNNINLLK

>BcucGR13

MAGLKVFIFHMWYYYLLAIGFSSLYFDFRRKRLLTPRWLRCYCFFTNGVLLLTLPFYCRNGMVYLDAFIEKPLLVIAGRFNVVMQSFVILYTLTMRHHHDQRYYGIAQRLLRLEWIYFEKWLEAPKLSYDRLFYLKLFTLFFQNITLVLGCVTILEKRSLTWDDYLLNVYFFIIWNTLYSITLTYFLTLLHIVRRYDVLNIYLQRRLRWLNAAPHDSSVQITDAWLSELRRLLDVHEQLQTIVVQTNNLYRSHITAMLLIFFLSSTVTFYLAFMNLAKWSTKQLYIITSFLLFALKSFDVFLTNHCCEILVARHSEALSLLKRHQHRLHCTNFSLNVALSRFTINIYGMFNLNHRATLAVLRNAVMHALILIQFDYIVDPEKRHEN

>BcucGR14

MSSHWRIGLARFLYNSTIWFSIAFGILPFRYNTKLRRISTSKCSLVYSVCIDALIVIISLFSWQTEDFLEENMLRHNKLMELLTKAIILSNIYTLIAIVVINWREYKSVLYIFNEFAAIERHYFSKHRVLARNCRSFYDYIIWKGVATLLQNLSFIFIICAHAAEINQRAVIVLTLALTLGNVIFLVVLHFYNFVITTYRCLWILHQRLQYLANRSQFRRPLYDVTYEVYEITGIYLRLIKICRRFASVYGQQLLFSIFAIMCANIQSIYYLRVIWSDKVYELSAFDIFYTGQAVLINIFDFWLTISACELALGMARDIAQLLRSFNDVARSDVELEQSVSFALPYFGDSGCRLQKQPTGVSIVRLN

>BcucGR15

MFLGLLPFKYNREKNTFRLSRFALSYSIVMNSLIFFWVVKNWPDMSNYELFIEKPLHYIVEIAITYVNSVGMILLFCTTWTGRKRLLALFNEIVAVKQLCDAKDFLRNYDASKQEKRIIVKFCSMLLQNLVFVMAMFFVSSIQDWSFILVLILAMIMLNEMYLISNQFYHNALFINHSILAVNHRLRCLSTKRQPASAGEINDIFGVYMRLIRLITQIMKAYEQQLLVIISVRLSVVVQCLFYTCMLFGGKELNVQMLDLLYYLEIILLTSLDYWLIMGICESVWRAQQQTEVELQNFSNFHILQTRVARDINTFSIFCTIHKFRFPLCGMFDINFATARNLCTSVMTALLWLVQYDFSLNM

>BcucGR16

MNTSTSRVVTALDVSVVVLSSVAGIGCGIFSVKPTRELNLRLRKFDAALHSFSNFKRDRITSRIMAIIPMTISAALIVFDIWTWVLQVEFSPKKSSKANIKWYIPFYLLYLVMIGFHILFANTAFGLGRRFRRLNAMLKCHYLSDSKPYSLVRTQINSIKITPDQAMSLHDSMDRLNTESLPKEGLGKTRVVLLKSLADNHESLGKCVQIFSNTYGIAVLCILVSCLLHMVATAYFLFLGLLNRNISGYVWGQVMWIILHILRLLLVVEPCHIATLESKKTIQIVSEIERKVHEPVLVEEIKKFWQQLLVIDVEFSALGLCRINRNILTSFSSAIATYLVILIQFQKASG

>BcucGR17

MCARYRRHSTDFILKATVSAANFLGILPFRYNKIKRRVESSKFLINYTIGINVITVILLILSWPTQDHLNVDILQRKPIVALVNSVNLYSTMYTIVLIIWANWRDHRRLLDFFNACWFMECECFRIFNEYQQECVQFDKYIIWKGVLTVLQNISFVNTVYDFENYSLFFRVLLIIFVYFLLNVMLITIQLFNICVLYQYRGFWVLNRRLEHISELELRQWNVVHIEEEISRLSGIYLRLLRICRRSTIMYEQQTLLMIAVLLGANILGLFFAKIIWTGKVLEMSAWSIFFNLQMILINLIDFWLTITICELTVNTSKKTSDLLRKFNDYRHLGKGLERNVSLM

>BcucGR18

MLKRHRSSPFSFSIISAVSVSSFLQLARKWPNLIKHMHRCEVVFLQKSYANGESRNFTKNVRKFGAILLFGAAMEHSVYIGTAIFNNDFQIKKCNLTVDFWKNYYMRERLQIFSIFNYHAWLIPLVQWITISTTFAWNYVDIFLSMIFRCFAIRFRQMHWRIKRHAKKQMPDEFWQQVRTDFMGLVDLLHRYDDGLSILILVSCCNNLYFICVQIFHSFNNRDNFMKEFYFWFSLLFVLVRILTMMLSAGAVHDEANQIMSTMYEIPTKFWCLELKRLNEIIVHDLVSFSGKGFFYLTRRLIFAMAGTIVVYELVLIDQVEDKDVATDFCTSRNV

>BcucGR19

MTFLLPIFSDFDDAFQSGDPLAELLNRLTIYISFCGILTTWWINWTGRQRLQRLFNEFLNIEIDYFHRYKHLSDECATFDYCVMWKVFATFLQNMSFFYSTALADNPAALFITFMCLTVLLTNVIFLVQSHFFLAVLFTYRFIWVLNRRLECIIDNEVTPRQHRGLSIEIDNIADIYTRLISLCERYTRIHQYQLLLVIGAMTMCNIEVLFYVRLLWSGKVSELNALNVLATLQIFVVNVLDFWLTITICELALVTSRSTQELLRSFNGKHRLATQLERSLECFAIICSSKKLRFHMCGLFDINHASGLKVLLTMILYLIYLVQYYHKNL

>BcucGR20

MEKIANQSYNTTIELANTLQQPTAASRVQRLRQYFISQEVFATLQPLFLFTYIYGLTPFRIVKRRNGTSEIRASCFGFCNTAAYVILYGVCFLNSLLNAESVVGYFFRTNISIVGDTLQICNGIVTGIVIYTTALTQRCKMRRIIEVLNELDLNFANIGVRVKYSRIYRYALVLIVAKTLIIAIYCAGVYLLLRSVHVNPSICVCIAFVLQHSVLFLAICLFCFIARGFERRLVILNKV

>BcucGR21

MVILVGIGFEHNCKVQLRVNSSTLKVVTAMDVGMVALTCVAGIVCGLCSVKSTQQLNLRLRKLDESLQFFLNHENDRFASRILATVPATLFGALISYDYFVWMRVVPEHFLEATFNWYLPFYGIYLILIGSHILYASNALGLGRRFRRLNTILKSRFLLEQKSCCKMKIWVTNLTADQELSSFHKFDRLKTQQFKPTVELLTLLVDNYESLYKCVEIFSK

>CcapGR64e

MAKVNPVEVSHEAARQNTNTMHLALRPFMMISQLLATLPVTGTWQKSSLEHVHFSWCTLIAFLSLIMITFSIIDVVLSTKVVVELGLKLYTVGPFSFSIISALSVSSFLQLARKWPNLIKHMDRCEQIFLQKSYGNKESR

NFSHNVRKFGGILLFGAALEHSVYIMTAIFNNDFQIKQCNLTVDFWKNYYMRERLQIFSIFNYHAWLIPLVQWITISTTFAWNYVDIFLSMIFKCFAIRFRQMHWRIKRHVNKHMEDDFWHEVRNHFMVLVELLHLFDDGLSTLILVSCCNNLYFICVQIFHSFNNRDTFIKEFYFWSSLLFVLLRILTMMLSASAVHDEANKIMSTMYEIPTKFWCLELKRLNEIIVHDLVAFSGKSFFFLTRRLIFAMAGTIVVYELVLIDQVEDKDVVTDFCTSRNHCPALDYRKTALSDNRNECIMDLKSWSAKPKIGKRHKRIIGVLRVRNIFRRGTKLDYQHSGSFLEAIGPVLLLAQIFALMPVCGILSKSASELYFSWKCVRTLYAMIIIFCLGPASLCTIAFAFRESFNFDTIEAIVFYVSIFLIALAFFQLARKWPALMVTWESIESKLPPLKTEMQKAALAHRIKMITLVATVCSVVEHLLSMLGIIYYVNGCPAMPGHPIQSFLFSNWAQFFYFFEYTNLAGIFGKVLNVISTFAWNFNDIFVMAVSVALSARFRQLNEHMLRAAKQPTTEKFWMDNRINYRNMCKLCEATDDTISIITLLCFSNNLFFICGKILKSLQKKPSWSHSVYFWFSLGFLLMRTLMLSLFSAEINDESKRPLVVFRSVPSKFWCPELKRFSEEVTTDVVALSGMKFFHLTRGLVLSVAGSIVTYELVLLQFNKEDKVNDCYEG

>CcapGR22f

MARFIYNATMWSSIAFGILPFGYNGKLQKISPSKYSLAYSVCVNVLQVVVSIPALPTEDFLNINVVKRNKWMDLLTRVITIANIYTLLVIVYINWRHYKSVLNIFNEFADIECQYFAKYGLLAWKCSAYEKYIILKGLATLLRNLSFIYFLSGLAKVVTWNILLVLALALLLGNRRLEYLSEHDVVHTHSRQLANEVYEIIGIYLRLIKISKCFGRIYGKQLLFSFAIIACGNIQALYYLGLLWADQFVGLSLLEIFNVLHGVSVNIFDFWLTITVCEQALGMPKEVTQLLRRFTIVAELDVEFEKSLEILSVVSKTHKLEIRLCGIIELNHLMGLKALLTMTLYLIYLVQFSYNDF

>CcapGR28b

MSQSRRADEDANSIEAAAAAAVADQDVCATHPQRVSGLRRFFHAHQLYESVQPIFIITFWHGLTPFFIKSDGTGNKKLKESIFGYLNTLLHITIYVGCYVMTLMNNFETIAGYFFNTGISRFADTMQIVSGLIGITVIYFTAILPKHRLQHSLRIMQDMDVLLHSVGVKILYSKLLRYSYLSLLVVFAVDTVYSCGNFLLLKSAELEPSTPLYIVFTLQHTVISIATALFQGIAKMLELRLVMLNKVLKNLAHQWDNSIVKPLPKQRSLQCLDSFSMYTIVTKDPCEIIQESMEIHHMICDAASTANKYFTYQLLTIISIAFLIIVFDAYYVLETLLGKSKRESKFKTGEFVTFFSCQMVLYLIAIISIVEGSNRAIKKSEKTGGIVHTLLNKAKNAEVKEKLQQFSMQLLHLKINFTAAGLFNIDRTLYFTISGALTTYLIILLQFTSSNTSDDHAAAHTHLNASSTAGIK

>CcapGR33a

MNLLLQTCSLIFGLMPFNRTQVDSNIAVDYSLVLVMPLLYLFCYYTVNFSGFSVRHLPTCNSICSLSSKLLMHVGCFLYLTIYFLTLLRQKGFYIEFEKRLTEIDELVGECIKVADTRNKLFVPKKKRLYYFTWIIILATFAFAIFYDINEMYYGPYCFISNMVFTFPYVAGSIVQGMFASYVSVISERFSTLNILFEKINHESDKRNLPIAVMDIENDAHKDNTYSGPAVISDLARRKNRFQQKQKSVKSDDRSNNNDSDFAEDEDSADEAYSFDEYDETRLSDGKTSEENLPSLFKLHDKILSLSVLVNAEFGAQCVPYMAACFVITIFSIFLETKVMFIVGGKNHVMDYVIYLYVIWSFTTMMVGYFVLRLCCNTNAHSKQSAMIVHEIMQKKPTFMLGNDVYYNKMKAFTLQFLHWEGYFQFNGIGLFALDYTFIFSTVSAATSYLIVLLQFDMTATLKNEGLTPKVFSTESN

>CcapGR22a

MWAQCRRAISDFTLNSAIYCSIALGILPYGYNRPRRGMVPSKAALVYSVCIDLLIICMALYAWPSARLIHFERIRHWSLLELLSQFVILVNVSALLNIMWTNWSEYKCILGLLNEYSSIERSYFIRHTSLMYECAAYEHYIILKGLAMLLKNLSFVYVALVLFKDVTWPIVAVHIMTMLLVNIISLVVMHFYIFILVTYRYIWIMRERLKCIANDLGLPHSSNLQSIRYELSEITVIYMRLLRFCEQFVRIYGKQMLLCISGVAGVNVLTLILLLFVWREVVHKLNVAYSWYVLVVNTLDFWLIISACELALGTAFDFVALQRSFSNYAPLSAALERELEMLALVCATSTPQFRLCGLLDLDYSTGLKVLMTTILYVIYLVQMNYKNL

>CcapGR22b

MMMEYRYDKVNKMFNCSENIFMSFKYRSAVALLSVVEFEMSSRWSRDELSCCIINSTVWLSIALGIFPYDYTGRRIVQSKCCLVYTLVVDVALAVFALHLWHEQQYLNLWRIANWSLMEVFTQFLYAVNIYGILAILWTNSWEYTYVWKVFKEFAALERSYFGKHQSLSAKCMTFHNLLTCKGLIVLMNIFFYLLIFTAMLIKARFIRTRIFVAVFSKFLNIVIILVIMHFYTHVMVTYRYIWVLKERLKHLSNFDTPHAQAQNLWREINEIVRIYMRLQQLSREFTRIYGKQVFFGIAALTAENAQNVLVLLFMLTPPAETWKIIFSAFFIAKNIVYFWLIICACELAVEAARELGQLLRCFNAMPQVDVEAERALQLLSFNCIINKPKFRVWGFVELSSSMGLEIILILVLQIIYLLQSNYTKMSLFSN

>CcapGR22e

MIIVVLMAFLLAVYASLDDAFKTGDPLAELLNDLTIAISFSALLPMWWVNWSSRQRLQNLFNEFAAIEDDFFYPYRHLLADCTTYDNYLLWKGLASLLQNLSFLNSASTVETPSILLIIFMCLLTILTNVVLLVATHFYLVVLHTYRLIWALNRRLEAIAADNIMPRLCQRRHLLSVEIDTMASIYARLISLCERYTRMHQLHLLLVIGSVTACNIEVLFYVRLLWSGKIPERTAFNVFAVFQIFVVNLLDFWLTITICELALVESRKTSAILRGFSAKPKLTLNVERSLECFAIICSSTKLRFHICGLFDINHLTGLKVLSTMILYLIYLVQYYHDNL

>CcapGR64a

MLNSNKLREILTKYQQDIRRNKTKPSKQLNECDRKTYIAKPNEKLFIENIEDPDVNALEQIGRQTPIEYFALKDKDFNYVKPVGPTDYSKLDTFHRAVGPILIIGQCFSLMPVLGVGQPNPRHVRFSLRTVRVMITLLFLVASTTLNLSMIKHLAKIGVNAKNLVGVMFFTCVQSSSILFFSLAPRWPRLIRFWTRTEMIFIRKPYERPQPDLSSRVRRAAATILSLSAVEHLLYLASAVVSQYRRAHLCAALNNTTVHFTFEDYTYKNYDYVYELLPNTTAVGSFILVGNFFCTFVWNYMDLFIMMVGKGIAYRFEQIKMRINKLLDKEVPESIFMEIRDHYIKLIELLEYVDEDLSGIILLSCANNLYFVCYQLLNIFNKLRWPINYVYFWFSLLFLIGRTAFVFLTAAAINDEAKEALGVLRRVSDKTWCVEVERLIFQMATTTVALSGKKFYFLTRRLLFGMAGTIVTYELVLLQFDEPNRSKGLP

ELCS

>CcapGR64f

MKFTFKVGWKIGNVTADARLRVQQQQRKLKTQRLWRRQCERAKRRQSQSGSNAATKCDPLAQKHNRKALLIKRTCTEFIAQMELLHRQIDKPPKALPKSKKSNFQQDGSFHQAVGKVLLFAEFFAFMPIKGVTTAHPRQLSFSLKNLRTWYCLIFIMTTTIDLGLTMIKVLPKPINFNGVEPLIFRLSIIVVCSSAIVLARKWPALMLDWHEVECDLPEYLTQIEKGRLAYKLKMVTVVAMALSLAEHLLNITSHIIYSNSCPQTNDRIRDFFILTNQHLFELFPYSIYLALWGKTQNILCTFIWNYMDVFVMIVSIGLAAKFKQLNDNLYKFKGMRMPEEYWSTRRKQYRNLCELCTRVDAAISVITMISFSNNLYFICVQLLRSLNKMPSFAQAAYFYFSFFFLIGRTLAVSLYSASINDESRKPLRILRCVPKESWCTEVKRFSEDISSDLVALSGMKFFYLTRKLVLSVAGTIVTYELVLIQFHQESKLIECNSPFMRNGSRH

>CcapGR66a

MRTRSIVLSIINYMHTNTGIYPYDFHKFYYQGILQGSRTSCCVVIAVIFIIFVLFNITLFTFGDQHVVAKYNHLNLIINVVFTYMTPIAMFTDEITAMRNQKLLPKLFARIDYVDEDLGQLGITVNNKRVYRRIWLMIVLTFVCEFYMFFAMMWLIVDEFEWFNVLWIFTSIPTIYNSLDKIWFLGILLGLRDRFEAINTALDDITEEIEKNNLQKRKGLVYEQSVGVGELMLHTSLSVGSERISDIKLERLVCNAFGELVCERESNKSAAVIVSYENSSYNFAALQERFISLCQLHDSICRIAQQLNQLWAYPLLVLMAFGFVFITSLLYFAYCATWNQSIPLIFSPAKNIYTSFIVIGYIAGKCVSLMFFSWQTTQASRRAGICLHKCGVAADTNEVYEIVNHLSLKLLNHDINFSACGFFKLDMGTLYAVCGAITSYLIILIQFNMAGEQVRISKELAAANESTALTLAAENYTVMPLQTTMEVH

>CcapGR43a

MEITEPTLCIFYVNKFLALAPFSVRRNSKGALDIRRSVMFSVYSGSLCLIMVILTYQGLLFDANSQVPVRPSFRMKSATSKVVTALDVSVVVLACSAGVGCGLLGYRATRELNTRLRKIDDSMHSYSNFKRDRTMAILMMVLPLTAITSILGLDLSTWLRFAIGVRTPQDDTELNVQWYIPFYSLYFILTGLQINFANTAFGLGRRFRRLNVMLRNSFLKDNNQKDAPMKPPITTVKVVSQHPLALHQSLAKLTNDTLQGSGKQKVNLLRLLEENHESLGKCMRLVSNSHGFAVLFILVSCLLHLVATSYFLFLELLSKKDSGMVWLQVLWIIFHALRLILVVEPCHLATVESKKTIQIVCEIERKIHDPILAEEVKKFWQQLLVVDVEFSASGLCRVNRTLLTSFSSAICTYLVILIQF

QNTNG

>CcapGR10a

MSFWERHKHNIYKYGHIYATLYGLMVINYIPQRSTNTFAYRLANIYGHALSICLIVVLPIYFVRNISALTAAHDQRGQLLQLVNFANTLLKYITVVVTYIANFAHYAAIRAVTRERQQLEDDFDGSLRAATGYDEHPRRQFEAMLLFKFALINAMMVVQVGNILFQHFTGAHSVRVYIAIYTFVLWNYTENMADYFYFINSSALKFYQQLNQQLRQVVREAKLLHYFRLCGQRRGTVPHFCGLLCDRLDTLVQRYQQINRLYQDSLTMHQFQILGMVFITLISNLTNSFILFNLFVKHSGASASPAIVLNALHAIIFYVDTYIVALVSENISLEMQNINQTLRQFNQFSMLDVRLQQTLEALTLFVMNNKVEVRICGLFVLDRRLTYLTAATGLSYFITLVQFDLNLT

>CcapGR93a

MSSYSRSERFAYLTLVALYYYGRLLCVFDWKLEKSKMLVRPTKCTNRILLLIWRIFITMIFISVMPSMMAPFQRLSIDSFLAFFANLQVITVTLFSVISFLIQELSERKIFKIIEKIVKIYKRIYGKSHVRQILGRTFVFSVITKLLLSFLGLIYEVPLILEGDQSVKSLCGIYLWLGTIYTLDACFLGFLIIGQMYTAMAAHLGEMVQAMSDYESEEPLSTLSKHERMKRLCVHSESIDDSNNIYFVLYGLTKQFHHIFRWQLIYSIYYNFVIILMVMHSFIWQYIYAGYVDFLALFSSLFKLWNLAMLILAAHGVVEKSQLPDLLNLDLVCSDIDARWDESVEAFICQRKVENLEIKVLGFFHLNNEFILVIISAIMSYLFILIQFALTKQQ

>BcorGR64f

MKFAFKIGWKIGNVAADARLRVQQQHDARKNRRLWRRQCERGIRRLSQNGSNAAATYGPQKLNHKSTLIRRTCTEFVAQRELMQLQIDKPPKILAKSVKEDFQYDGSFHQAVGKVLLFAEFFAIMPVKGVTAAHPQQLSFSWTNIRTLYCLLFITTTTIDLGLTMNKVLHKPINFDSVEPLIFRLSIIVVCISAIVLARKWPALMLDWYEIECDLPEYLTQMEKGRLAYRIKMVTVVAMTLSLGEHLLNILSNIHYSKYCPQTEDPIENFFILTNQQLFMIFSYSVPLAFWGKLQNILCTFIWNYMDVFVMIVSIGLAAKFRQLNDNLFKFKGMRMPEMYWSTRRKQYRNLCELCTRIDGAISLITMISFSNNLYFICVQLLRSLNKMPSLAHVAYFYFSFFFLIGRTLAVSLYSASINDESRKPLRVLRCVPKESWCTEVKRFSEDISTDLVALSGMKFFYLTRKLVLSVAGTIVTYELVLIQFHQDSKLAECVSSIRTLSSPGNNTLH

>BcorGR63a

MFNSYNRRKKHDTVFLNVKPTFNSQGNGLRKYSTGLLDKEDNPFYDVNSSSGSRASVGTITTLNENFRANIFYNNIAPIQWFLHMLGVLPITRREPGKAKFRINSIAFGYSLAFFILLSVFVTYVAKNRISIVTSLSGPFEEAVIAYLFLVNILPLILIPILWWEARKIAKLWNDWDDFEILYYQISGHSMPLNLRRKTTMIAVVLPILSILSVVITHITMADFQIIQVIPYCILDNLSAMLGAWWFIICESLSMTANILGERFQRALRHIGPAAMVADYRALWLRLSKLTRDTGNATCYTFTFINLYLFFIITLSVYGLMSQLSEGFGIKDIGLAITAIWNVFLLFYICDKAHYASFNVRTNFQKKLLMVELNWMNSDAQTEINMFIRATEMNPSNINCGGFFDVNRNLFKGLLTTMVTYLVVLLQFQISIPTDTGRHMNVSVAELATDMMLESAEVELTTTSTTSTTTTTTTKMPPPARGRKG

>BcorGR5a

SQIFFVFDYSTWLAWFGKILNVLMTFGWSYMDVFLMIIGIGLSSLFEQVQRSLERVKGQVMPESYWTRTRLQYRLICDLIEQVDSAVSAITVLSFANNLYFVCIQLLKSMNTMPSVAHFVYFYASLGFLLARTLAVSLYLSEVNDRSREPLKIIKKVPKEGFHPEVDRLAHEIGMDTVALTGLKFFNITRGLVLTVAGTIVTYELV

>BcorGR28bc

MDEDTNGIEAQEVRATRPQRVSGLRRFFQAQQLYESVQPLFLITFWHGLTPFFIKSDGAGNKKLKESIFGYINTFLHITIYVACYLLTVMNDFETVAGYFFNSGVSRFGDTLQIVSGLIGVTIIYITAMLPKHRLKYSLRIVQDIDLMLHKVGVKIIYTKLLHYSYFSILLVFAVDTVYSCGNFMLLKSANLEPSTPLYIVFTLQHTVISIATMMYHGFVKMVEMRLTMLNKVLKKLAHQWDNSIVKPMPKQRSLQCLDSFSMYTIVTNNPCEIIQESMEIHHMICDAASTANKYFTYQLLTIISIAFLLIVFDAYYVLEILLGKSAHEGKFKTVEFVTFFSCQMILYVIAIVSIVEGSNRAIQKSEKTSGIVHSLLNKAKNAELKEKLQQFSLQLLHLKIHFTAAGLFNIDRTLYFTISGALTTYLIILLQFSNSNVPEPPFPSMEENDTIPIRSLVSNLTIGG

>BcorGR21a

MAYWAIATRKGQSPPMKITPVLNPNQREFLEDELLYREKLEILAENNTISTDLFVRKFEDIDDPVLLDKHDSFYHTTKSLLVLFQIMGVMPIHRNPQKPGMPRTGYSWTSKQVFWAVCVFSMQTTIVVMVLRERVNTFLNDSDRRFDEAIYNVIFISLLFTNFLLPVASWRHGPQVAIFKNMWTNYQLKFLKVTGSPIVFPNLYPLTWSLCFFSWGVSIAINLSQYYLQPDFKLWYTFAYYPIIAMLNGFCSLWYINCTAFGTASRALSASLELTLMSDKPAKKLTEYRHLWVDLSHMMQQLGRAYSNMYGMYCLVVFFTTIIATYGSLSEIMDHGATYKEVGLFVIVFYCMSLLYIICNEAHYATQSVGLDFQTKLLNVDLTAVDSATQKEVEMFLMAITKNPPIMNLDGYANINRELITSNISFMATYLVVLLQFKITEQRNYAMKQSRAELLA

>DmelGR5a

MRQLKGRNRCNRAVRHLKVQGKMWLKNLKSGLEQIRESQVRGTRKNFLHDGSFHEAVAPVLAVAQCFCLMPVCGISAPTYRGLSFNRRSWRFWYSSLYLCSTSVDLAFSIRRVAHSVLDVRSVEPIVFHVSILIASWQFLNLAQLWPGLMRHWAAVERRLPGYTCCLQRARPARRLKLVAFVLLVVSLMEHLLSIISVVYYDFCPRRSDPVESYLLGASAQLFEVFPYSNWLAWLGKIQNVLLTFGWSYMDIFLMMLGMGLSEMLARLNRSLEQQVRQPMPEAYWTWSRTLYRSIVELIREVDDAVSGIMLISFGSNLYFICLQLLKSINTMPSSAHAVYFYFSLLFLLSRSTAVLLFVSAINDQAREPLRLLRLVPLKGYHPEVFRFAAELASDQVALTGLKFFNVTRKLFLAMAGTVATYELVLIQFHEDKKTWDCSPFNLD

>DmelGR28b

MIRCGLDIFRGCRGRFRYWLSARDCYDSISLMVAIAFALGITPFLVRRNALGENSLEQSWYGFLNAIFRWLLLAYCYSYINLRNESLIGYFMRNHVSQISTRVHDVGGIIAAVFTFILPLLLRKYFLKSVKNMVQVDTQLERLRSPVNFNTVVGQVVLVILAVVLLDTVLLTTGLVCLAKMEVYASWQLTFIFVYELLAISITICMFCLMTRTVQRRITCLHKVLKNLAHQWDTRSLKAVNQKQRSLQCLDSFSMYTIVTKDPAEIIQESMEIHHLICEAAATANKYFTYQLLTIISIAFLIIVFDAYYVLETLLGKSKRESKFKTVEFVTFFSCQMILYLIAIISIVEGSNRAIKKSEKTGGIVHSLLNKTKSAEVKEKLQQFSMQLMHLKINFTAAGLFNIDRTLYFTISGALTTYLIILLQFTSNSPNNGYGNGSSCCETFNNMTNHTL

>DmelGR66a

MAQAEDAVQPLLQQFQQLFFISKIAGILPQDLEKFRSRNLLEKSRNGMIYMLSTLILYVVLYNILIYSFGEEDRSLKASQSTLTFVIGLFLTYIGLIMMVSDQLTALRNQGRIGELYERIRLVDERLYKEGCVMDNSTIGRRIRIMLIMTVIFELSILVSTYVKLVDYSQWMSLLWIVSAIPTFINTLDKIWFAVSLYALKERFEAINATLEELVDTHEKHKLWLRGNQEVPPPLDSSQPPQYDSNLEYLYKELGGMDIGSIGKSSVSGSGKNKVAPVAHSMNSFGEAIDAASRKPPPPPLATNMVHESELGNAAKVEEKLNNLCQVHDEICEIGKALNELWSYPILSLMAYGFLIFTAQLYFLYCATQYQSIPSLFRSAKNPFITVIVLSYTSGKCVYLIYLSWKTSQASKRTGISLHKCGVVADDNLLYEIVNHLSLKLLNHSVDFSACGFFTLDMETLYGVSGGITSYLIILIQFNLAAQQAKEAIQTFNSLNDTAGLVGAATDMDNISSTLRDFVTTTMTPAV

>DmelGR21a

MSFWAVSRGLTPPSKVVPMLNPNQRQFLEDEVRYREKLKLMARGDAMEEVYVRKQETVDDPLELDKHDSFYQTTKSLLVLFQIMGVMPIHRNPPEKNLPRTGYSWGSKQVMWAIFIYSCQTTIVVLVLRERVKKFVTSPDKRFDEAIYNVIFISLLFTNFLLPVASWRHGPQVAIFKNMWTNYQYKFFKTTGSPIVFPNLYPLTWSLCVFSWLLSIAINLSQYFLQPDFRLWYTFAYYPIIAMLNCFCSLWYINCNAFGTASRALSDALQTTIRGEKPAQKLTEYRHLWVDLSHMMQQLGRAYSNMYGMYCLVIFFTTIIATYGSISEIIDHGATYKEVGLFVIVFYCMGLLYIICNEAHYASRKVGLDFQTKLLNINLTAVDAATQKEVEMLLVAINKNPPIMNLDGYANINRELITTNISFMATYLVVLLQFKITEQRRIGQQQA

>DmelGR43a

MEISQPSIGIFYISKVLALAPYATVRNSKGRVEIGRSWLFTVYSATLTVVMVFLTYRGLLFDANSEIPVRMKSATSKVVTALDVSVVVMAIVSGVYCGLFSLNDTLELNDRLNKIDNTLNAYNNFRRDRWRALGMAAVSLLAISILVGLDVGTWMRIAQDMNIAQSDTELNVHWYIPFYSLYFILTGLQVNIANTAYGLGRRFGRLNRMLSSSFLAENNATSAIKPQKVSTVKNVSVNRPAMPSALHASLTKLNGETLPSEAAAKNKGLLLKSLADSHESLGKCVHLLSNSFGIAVLFILVSCLLHLVATAYFLFLELLSKRDNGYLWVQMLWICFHFLRLLMVVEPCHLAARESRKTIQIVCEIERKVHEPILAEAVKKFWQQLLVVDADFSACGLCRVNRTILTSFASAIATYLVILIQFQRTNG

>DmelGR63a

MANYYRRKKGDAVFLNAKPLNSANAQAYLYGVRKYSIGLAERLDADYEAPPLDRKKSSDSTASNNPEFKPSVFYRNIDPINWFLRIIGVLPIVRHGPARAKFEMNSASFIYSVVFFVLLACYVGYVANNRIHIVRSLSGPFEEAVIAYLFLVNILPIMIIPILWYEARKIAKLFNDWDDFEVLYYQISGHSLPLKLRQKAVYIAIVLPILSVLSVVITHVTMSDLNINQVVPYCILDNLTAMLGAWWFLICEAMSITAHLLAERFQKALKHIGPAAMVADYRVLWLRLSKLTRDTGNALCYTFVFMSLYLFFIITLSIYGLMSQLSEGFGIKDIGLTITALWNIGLLFYICDEAHYASVNVRTNFQKKLLMVELNWMNSDAQTEINMFLRATEMNPSTINCGGFFDVNRTLFKGLLTTMVTYLVVLLQFQISIPTDKGDSEGANNITVVDFVMDSLDNDMSLMGASTLSTTTVGTTLPPPIMKLKGRKG

>DmelGR33a

MIQIMNWFSMVIGLIPLNRQQSETNFILDYAMMCIVPIFYVACYLLINLSHIIGLCLLDSCNSVCKLSSHLFMHLGAFLYLTITLLSLYRRKEFFQQFDARLNDIDAVIQKCQRVAEMDKVKVTAVKHSVAYHFTWLFLFCVFTFALYYDVRSLYLTFGNLAFIPFMVSSFPYLAGSIIQGEFIYHVSVISQRFEQINMLLEKINQEARHRHAPLTVFDIESEGKKERKTVTPITVMDGRTTTGFGNENKFAGEMKRQEGQQKNDDDDLDTSNDEDEDDFDYDNATIAENTGNTSEANLPDLFKLHDKILALSVITNGEFGPQCVPYMAACFVVSIFGIFLETKVNFIVGGKSRLLDYMTYLYVIWSFTTMMVAYIVLRLCCNANNHSKQSAMIVHEIMQKKPAFMLSNDLFYNKMKSFTLQFLHWEGFFQFNGVGLFALDYTFIFSTVSAATSYLIVLLQFDMTAILRNEGLMS

>DmelGR64f

MKILPKLERKLRRLKKRVTRTSLFRKLDLVHESARKKAFQESCETYKNQIENEYEIRNSLPKLSRSDKEAFLSDGSFHQAVGRVLLVAEFFAMMPVKGVTGKHPSDLSFSWRNIRTCFSLLFIASSLANFGLSLFKVLNNPISFNSIKPIIFRGSVLLVLIVALNLARQWPQLMMYWHTVEKDLPQYKTQLTKWKMGHTISMVMLLGMMLSFAEHILSMVSAINYASFCNRTADPIQNYFLRTNDEIFFVTSYSTTLALWGKFQNVFSTFIWNYMDLFVMIVSIGLASKFRQLNDDLRNFKGMNMAPSYWSERRIQYRNICILCDKMDDAISLITMVSFSNNLYFICVQLLRSLNTMPSVAHAVYFYFSLIFLIGRTLAVSLYSSSVHDESRLTLRYLRCVPKESWCPEVKRFTEEVISDEVALTGMKFFHLTRKLVLSVAGTIVTYELVLIQFHEDNDLWDCDQSYYS

>DmelGR64a

MKGPNLNFRKTPSKDNGVKQVESLARPETPPPKFVEDSNLEFNVLASEKLPNYTNLDLFHRAVFPFMFLAQCVAIMPLVGIRESNPRRVRFAYKSIPMFVTLIFMIATSILFLSMFTHLLKIGITAKNFVGLVFFGCVLSAYVVFIRLAKKWPAVVRIWTRTEIPFTKPPYEIPKRNLSRRVQLAALAIIGLSLGEHALYQVSAILSYTRRIQMCANITTVPSFNNYMQTNYDYVFQLLPYSPIIAVLILLINGACTFVWNYMDLFIMMISKGLSYRFEQITTRIRKLEHEEVCESVFIQIREHYVKMCELLEFVDSAMSSLILLSCVNNLYFVCYQLLNVFNKLRWPINYIYFWYSLLYLIGRTAFVFLTAADINEESKRGLGVLRRVSSRSWCVEVERLIFQMTTQTVALSGKKFYFLTRRLLFGMAGTIVTYELVLLQFDEPNRRKGLQPLCA

>DmelGR64e

MARTTGDPAKRRRCMSRIKFWRRSRVGSEVVEKDTKRFKLSLIKAWLLRIRQEDYKYSGSFQEAIKPVLIIAQIFALMPVRKVSSKFAEDLTFTWFSVRSYYALVTILFFGVSSGYMVAFVTSVSFNFDSVETLVFYLSIFLISLSFFQLARKWPEIAQSWQLVEAKLPPLKLPKERRSLAQHINMITIVATTCSLVEHIMSMLSMGYYVNSCPRWPDRPIDSFLYLSFSSVFYFVDYTRFLGIVGKVVNVLSTFAWNFNDIFVMAVSVALAARFRQLNDYMMREARLPTTVDYWMQCRINFRNLCKLCEEVDDAISTITLLCFSNNLYFICGKILKSMQAKPSIWHALYFWFSLVYLLGRTLILSLYSSSINDESKRPLVIFRLVPREYWCDELKRFSEEVQMDNVALTGMKFFRLTRGVVISVAGTIVTYELILLQFNGEEKVPGCFEN

>DmelGR64c

MQQSGQKGTRNTLQHAIGPVLVIAQFFGVLPVAGVWPSCRPERVRFRWISLSLLAALILFVFSIVDCALSSKVVFDHGLKIYTIGSLSFSVICIFCFGVFLLLSRRWPYIIRRTAECEQIFLEPEYDCSYGRGYSSRLRLWGVCMLVAALCEHSTYVGSALYNNHLAIVECKLDANFWQNYFQRERQQLFLIMHFTAWWIPFIEWTTLSMTFVWNFVDIFLILICRGMQMRFQQMHWRIRQHVRQQMPNEFWQRIRCDLLDLSDLLGIYDKELSGLIVLSCAHNMYFVCVQIYHSFQSKGNYADELYFWFCLSYVIIRVLNMMFAASSIPQEAKEISYTLYEIPTEFWCVELRRLNEIFLSDHFALSGKGYFLLTRRLIFAMAATLMVYELVLINQMAGSEVQKSFCEGGVGSSKSIFS

>DmelGR22e

MFRPSGSGYRQKWTGLTLKGALYGSWILGVFPFAYDSWTRTLRRSKWLIAYGFVLNAAFILLVVTNDTESETPLRMEVFHRNALAEQINGIHDIQSLSMVSIMLLRSFWKSGDIERTLNELEDLQHRYFRNYSLEECISFDRFVLYKGFSVVLELVSMLVLELGMSPNYSAQFFIGLGSLCLMLLAVLLGASHFHLAVVFVYRYVWIVNRELLKLVNKMAIGETVESERMDLLLYLYHRLLDLGQRLASIYDYQMVMVMVSFLIANVLGIYFFIIYSISLNKSLDFKILVFVQALVINMLDFWLNVEICELAERTGRQTSTILKLFNDIENIDEKLERSITDFALFCSHRRLRFHHCGLFYVNYEMGFRMAITSFLYLLFLIQFDYWNL

>DmelGR93a

MFSSSSAMTGKRAESWSRLLLLWLYRCARGLLVLSSSLDRDKLQLKATKQGSRNRFLHILWRCIVVMIYAGLWPMLTSAVIGKRLESYADVLALAQSMSVSILAVISFVIQARGENQFREVLNRYLALYQRICLTTRLRHLFPTKFVVFFLLKLFFTLCGCFHEIIPLFENSHFDDISQMVGTGFGIYMWLGTLCVLDACFLGFLVSGILYEHMANNIIAMLKRMEPIESQDERYRMTKYRRMQLLCDFADELDECAAIYSELYHVTNSFRRILQWQILFYIYLNFINICLMLYQYILHFLNDDEVVFVSIVMAFVKLANLVLLMMCADYTVRQSEVPKKLPLDIVCSDMDERWDKSVETFLGQLQTQRLEIKVLGFFHLNNEFILLILSAIISYLFILIQFGITGGFEASEDIKNRFD

>DmelGR22b

MFGSSREIRPYLARQMLKTTLYGSWLLGIFPFTLDSGKRIRQLRRSRCLTLYGLVLNYFLIFTLIRLAFEYRKHKLEAFKRNPVLEMINVVIGIINVLSALIVHFMNFWGSRKVGEICNELLILEYQDFEGLNGRNCPNFNCFVIQKCLTILGQLLSFFTLNFALPGLEFHICLVLLSCLMEFSLNLNIMHYHVGVLLIYRYVWLINEQLKDLVSQLKLNPETDFSRIHQFLSLYKRLLELNRKLVIAYEYQMTLFIIAQLSGNIVVIYFLIVYGLSMRTYSIFLVAFPNSLLINIWDFWLCIAACDLTEKAGDETAIILKIFSDLEHRDDKLEMSVNEFAWLCSHRKFRFQLCGLFSMNCRMGFKMIITTFLYLVYLVQFDYMNL

>DmelGR10a

MTSPDERKSFWERHEFKFYRYGHVYALIYGQVVIDYVPQRALKRGVKVLLIAYGHLFSMLLIVVLPGYFCYHFRTLTDTLDRRLQLLFYVSFTNTAIKYATVIVTYVANTVHFEAINQRCTMQRTHLEFEFKNAPQEPKRPFEFFMYFKFCLINLMMMIQVCGIFAQYGEVGKGSVSQVRVHFAIYAFVLWNYTENMADYCYFINGSVLKYYRQFNLQLGSLRDEMDGLRPGGMLLHHCCELSDRLEELRRRCREIHDLQRESFRMHQFQLIGLMLSTLINNLTNFYTLFHMLAKQSLEEVSYPVVVGSVYATGFYIDTYIVALINEHIKLELEAVALTMRRFAEPREMDERLTREIEHLSLELLNYQPPMLCGLLHLDRRLVYLIAVTAFSYFITLVQFDLYLRKKS

>DmelGR22f

MKMFQPRRGFSCHLAWFMLQTTLYASWLLGLFPFTFDSRRKQLKRSRWLLLYGFVLHSLAMCLAMSSHLASKQRRKYNAFERNPLLEKIYMQFQVTTFFTISVLLLMNVWKSNTVRKIANELLTLEGQVKDLLTLKNCPNFNCFVIKKHVAAIGQFVISIYFCLCQENSYPKILKILCCLPSVGLQLIIMHFHTEIILVYRYVWLVNETLEDSHHLSSSRIHALASLYDRLLKLSELVVACNDLQLILMLIIYLIGNTVQIFFLIVLGVSMNKRYIYLVASPQLIINFWDFWLNIVVCDLAGKCGDQTSKVLKLFTDLEHDDEELERSLNEFAWLCTHRKFRFQLCGLFSINHNMGFQMIITSFLYLVYLLQFDFMNL

>DmelGR28a

MAFKLWERFSQADNVFQALRPLTFISLLGLAPFRLNLNPRKEVQTSKFSFFAGIVHFLFFVLCFGISVKEGDSIIGYFFQTNITRFSDGTLRLTGILAMSTIFGFAMFKRQRLVSIIQNNIVVDEIFVRLGMKLDYRRILLSSFLISLGMLLFNVIYLCVSYSLLVSATISPSFVTFTTFALPHINISLMVFKFLCTTDLARSRFSMLNEILQDILDAHIEQLSALELSPMHSVVNHRRYSHRLRNLISTPMKRYSVTSVIRLNPEYAIKQVSNIHNLLCDICQTIEEYFTYPLLGIIAISFLFILFDDFYILEAILNPKRLDVFEADEFFAFFLMQLIWYIVIIVLIVEGSSRTILHSSYTAAIVHKILNITDDPELRDRLFRLSLQLSHRKVLFTAAGLFRLDRTLIFTITGAATCYLIILIQFRFTHHMDDTSSNSTNNLHSIHLGD

>DmelGR22d

MFRPRCGLRQKFVYVILKSILYSSWLLGIFPFKYEPKKRRLRRSMWLILFGVVISSSLLILMVKQSAEDREHGIMLDVFQRNALLYQISSLMGVVGVVSICTVHLRTLWRSKHLEEIYNGLMLLEAKYFCSNAVECPAFDGYVIQKGVVIVVGLLAPWMVHFGMPDSKLPVLNVLVVSMVKLGTLLLALHYHLGVVIIYRFVWLINRELLSLVCSLRGNHKGSSSRVRFLLKLYNKLVNLYSKLADCYDCQTVLMMAIFLAANIIVCFYMIVYRISLSKMSFFVMLIMFPLAIANNFMDFWLSMKVCDLLQKTGRQTSMILKLFNDIENMDKDLEISISDFALYCSHRRFKFLHCGLFHVNREMGFKMFVASVLYLLYLVQFDYMNL

>DmelGR59d

MADLLKLCLRIAYAYGRLTGVINFKIDLKTGQALVTRGATLISVSTHLLIFALLLYQTMRKSVVNVMWKYANSLHEYVFLVIAGFRVVCVFLELVSRWSQRRTFVRLFNSFRRLYQRNPDIIQYCRRSIVSKFFCVTMTETLHIIVTLAMMRNRLSIALALRIWAVLSLTAIINVIITQYYVATACVRGRYALLNKDLQAIVTESQSLVPNGGGVFVTKCCYLADRLERIAKSQSDLQELVENLSTAYEGEVVCLVITYYLNMLGTSYLLFSISKYGNFGNNLLVIITLCGIVYFVFYVVDCWINAFNVFYLLDAHDKMVKLLNKRTLFQPGLDHRLEMVFENFALNLVRNPLKLHMYGLFEFGRGTSFAVFNSLLTHSLLLIQYDVQNF

>DmelGR59e

MDSSYWENLLLTINRFLGVYPSGRVGVLRWLHTLWSLFLLMYIWTGSIVKCLEFTVEIPTIEKLLYLMEFPGNMATIAILVYYAVLNRPLAHGAELQIERIITGLKGKAKRLVYKRHGQRTLHLMATTLVFHGLCVLVDVVNYDFEFWTTWSSNSVYNLPGLMMSLGVLQYAQPVHFLWLVMDQMRMCLKELKLLQRPPQGSTKLDACYESAFAVLVDAGGGSALMIEEMRYTCNLIEQVHSQFLLRFGLYLVLNLLNSLVSICVELYLIFNFFETPLWEESVLLVYRLLWLAMHGGRIWFILSVNEQILEQKCNLCQLLNELEVCSSRLQRTINRFLLQLQRSIDQPLEACGIVTLDTRSLGGFIGVLMAIVIFLIQIGLGNKSLMGVALNRSNWVYV

>DmelGR22a

MSQPKRIHRICKGLARFTIRATLYGSWVLGLFPFTFDSRKRRLNRSKWLLAYGLVLNLTLLVLSMLPSTDDHNSVKVEVFQRNPLVKQVEELVEVISLITTLVTHLRTFSRSSELVEILNELLVLDKNHFSKLMLSECHTFNRYVIEKGLVIILEIGSSLVLYFGIPNSKIVVYEAVCIYIVQLEVLMVVMHFHLAVIYIYRYLWIINGQLLDMASRLRRGDSVDPDRIQLLLWLYSRLLDLNHRLTAIYDIQVTLFMATLFSVNIIVGHVLVICWINITRFSLLVIFLLFPQALIINFWDLWQGIAFCDLAESTGKKTSMILKLFNDMENMDQETERRVTEFTLFCSHRRLKVCHLGLLDINYEMGFRMIITNILYVVFLVQFDYMNLKFKTD

>BdorGR63a

MFNSYNRRKKHDTVFLNVKPTFNGQGNGLRKYSTGLLDKEDNPFYDVNSSSGSRASVGTITTLNENFRANIFYNNIAPIQWFLHMLGVLPITRREPGKAKFRINSIAFGYSFAFFILLSVFVTYVAKNRISIVTSLSGPFEEAVIAYLFLVNILPLILIPILWWEARKIAKLWNDWDDFEILYYQISGHSMPLNLRRKTTMIAVVLPILSILSVVITHITMADFQIIQVIPYCILDNLSAMLGAWWFIICESLSMTANILGERFQRALRHIGPAAMVADYRALWLRLSKLTRDTGNATCYTFTFINLYLFFIITLSVYGLMSQLSEGFGIKDIGLAITAIWNVFLLFYICDKAHYASFNVRTNFQKKLLMVELNWMNSDAQTEINMFIRATEMNPSNINCGGFFDVNRNLFKGLLTTMVTYLVVLLQFQISIPTDTGRHMNVSVAELATDMMLESAEDELTTTSTTSTTTTTTTKMPPPARGRKG

>BdorGR64e

MPVCGILSKTASKVYFSWKSVRTCYAMLVIFCLGPASLCTIAFAFRERFNFDTVEAIVFYVSIFLIAMAFFQLARKWPALMVKWESIESKLPPLKTEMQRAALAHRIKMITLVATMCSMVEHLLSMLGIIYYVNACPTMPGHPIRSFLYTNWSQYFYFFDYTDWAGIFGKVLNVISTFAWNFNDIFVMAVSVALSARFRQLNEHMLRVAKRPTSEKFWIENRINYRNLCKLCEATDDTISLITLLCFSNNLFFICGKILKSLQKKPSFSHTMYFWFSLGFLLMRTLMLSLYSAEINDESKRPLVVFRSVPSVSWCRELKRFSEEVTTDVVALSGMKFFHLTRGLVLTVAGSIVTYELVLLQFNKEGKVNDCYEG

>BdorGR22b

MLLGLLPFKCNKEKRTFRSSRVALSYNVVSNTLLFAWVVKNWPDMSNYELFVAKPLLYVVENAVSYLNCVGMILIFCSTWAGKNRLLRLFNEIATVRQLCDSKAFLRDFDASMMEKRIILKFCSMFLQNLLFFSSTFYWANRMDVNYALVLMLTIILLNEIFLISNQFYHNTLSINHSILAVNHRLRCLNTRKQLLAAGEINEIFGIYIRLIRLIAHTTKAYEQQLLVILSVRLSVVVQCLFSTCMQFGGRGLQVNKLELLYYLHIIMLTSLDYWLIMAVCESVWRAQQQTEVELKHFNASRSLSVSMARDLNTFSIFCSIHKFRFPLCGLFEINFATARNLCTSVVTALIWLVQYDFATSM

>BdorGR22d

MHTAHAARRKFANFIHCYIFKISMLLGLLPFKYSKKKRTFRSSRVALSYSVVSNTLLFAWVVKNWPDMSNYELFVAKPLLFIVQNAVSYLNCVGMILIFSSTWAGRKRLLRLFNETVTVRQLCDSKAFLRDFDASMMEKRIILKFCSMFLQNLLFFSSTFYWANRMDGNYFLVLMMSIILLNEIFLISNQFYHNTLSVNHSILAVNHRLRCLNTRKQLLAAGEINEIFGIYIRLIRLIAHTTKAYEQQLLVILSVRLSVVVQCLFSTCMQFGGRGLQVNKLELLISLHIIMLTSLDYWLIMAVCESVWRAQQQTEVELKHFNPFRTLSVSMARDLNTFSIFCSIHKFRFPLCGLFEINFATARNLCTSVVTALIWLVQYDFATNM

>BdorGR21a

MAYWAIATRKGQSPPMKITPVLNPNQREFLEDELLYREKLEILAENNTISTDLFVRKFEDIDDPVLLDKHDSFYHTTKSLLVLFQIMGVMPIHRNPQKPGMPRTGYSWTSKQVFWAVCVFSMQTTIVVMVLRERVNTFLNDSDRRFDEAIYNVIFISLLFTNFLLPVASWRHGPQVAIFKNMWTNYQLKFLKVTGSPIVFPNLYPLTWSLCFFSWGVSIAINLSQYYLQPDFKLWYTFAYYPIIAMLNGFCSLWYINCTAFGTASRALSASLELTLMSDKPAKKLTEYRHLWVDLSHMMQQLGRAYSNMYGMYCLVVFFTTIIATYGSLSEIMDHGATYKEVGLFVIVFYCMSLLYIICNEAHYATQSVGLDFQTKLLNVDLTAVDSATQKEVEMFLMAITKNPPIMNLDGYANINRELITSNISFMATYLVVLLQFKITEQRNYSLKQSRAELLA

>BdorGR64f

MKFAFKIGWKIGNVAADARLRVQQQHDARKNRRLWRRQCERGIRRLSQNGSDAAATYGPQKPNHKSTLIRRTCTEFVAQRELMQLQIDKPPKILAKSVKEDFQHDGSFHQAVGKVLLFAEFFAIMPVKGVTAAHPGQLSFSWTNIRTLYCLLFITTTTIDLGLTMNKVLHKPINFDSVEPLIFRLSIIVVCISAIVLARKWPALMLDWYEIECDLPEYLTQMEKGRLAYRIKMVTVVAMTLSLGEHLLNILSNIHYSKYCPQSEDPIENFFILTNQHLFMIFSYSVPLAIWGKVQNILCTFIWNYMDVFVMIVSIGLAAKFRQLNDNLFKFKGMRMPEMYWSTRRKQYRNLCELCTRIDGAISLITMISFSNNLYFICVQLLRSLNKMPSLAHVAYFYFSFFFLIGRTLAVSLYSASINDESRKPLRVLRCVPKESWCTEVKRFSEDISTDLVALSGMKFFYLTRKLVLSVAGTIVTYELVLIQFHQDSK

LAECVSSIRTLSSPGNNTLH

>BdorGR64a

MLSRNKLREILSKHQQAFEGSKILPSKSANEKIKNISLGKPIEKIFLENIEDSDVMKPVGMTGRRTPIEYFTYKEDDFHYKKPSEHTDYSRLDIFHRAVSPILIIGQCFSLMPVQGVGQPNPRHVRFSFKSLRVLITLIFLVASSALNLAMMKHLARIGVNAKNLVGVVFFTCVQSSTILFLSLAQRWPRLIRFWTRTEMIFIRKPYETPKRDLSTRVRRAAVTIIFLSAVEHLLYLASAVVSQYRRANFCATLQNSTVHFTFEDYTYKNYDYVYELFPNTTLVGSLILVVNFVCTFVWNYMDLFIMMVGKGIAYRFEQMKMRINNLLNKEVPESIFMEIRDHYVKLLELLEYVDDDLSGIILLSCANNLYFVCYQLLNIFNKLRWPINYVYFWFSLLFLIGRTAFVFLTAASINDEAKDALAVLRRVSAKTWCVEVERLIFQMATTTVALSGKKFYFLTRRLLFGMAGTIVTYELVLLQFDEPNRSKGLPHLCA

>BdorGR66a

MAHQTVQPILVHFGTLFTFCKLLGLYPHDLQAFRGIHTLQSSKIGTAIVVVTMLAVVVLYNLLIYFFSSEDHDLKASQSTLTFVIGIFLTYIGLGMMITDQLSALRNQSKTGELYERIRAVDEQLLKENVYVDISKTAKNILLMIVLTVVSELIILISTYITLVDLTDWKSILWLFSCFPTLYNSLDKIWFANTLSALKQRFFVINTALEDMVESHERLKRWTENGGDGGGQIFRRPSIANVSIDPSLEYLYKELTHMEAVKAYNMARNKISPIAHSLNSFGDALETPKKPYKYTSQPPTFNMVYESELNKDIEKVEEKLNNLCQLHDEICEIGKLVNELWSYPILVLMAYGFLIFTAQLYFLYCATQGQAIPSLFRSAKSATITTIFLSYTAGKCIYLIYLSWKTSLESKRTGICLHKCGVVADNNLLYEIVNHLSLKLLNHSVDFSACGFFTLDMETLYGVSGGITSYLIILIQFNLAAQQAKDASNAAETNYNTMLTTEAGNTTALMDYFTTTFMPYAQTDLY

>BdorGR43a

MEINESSLSVFYLSKLLALAPISIQQNAKGVIEIKRSIMFSIYAIALGLIMVILCYEGLLFDANSKVPLRMNSSTSRVVTAMDVTVVVFSSVAGIGCGIFSVTPSRELNLRLRKFDASLHSFSNFKRDRITTRIMAIIPMTISGALIAFDIWTWVLQIEISPNMSSTANVKWYIPFYLLYFVMIGFHILFANTAIGLGRRFRRLNAMLKCQYLSESKPYSLVRAQINNIKIIPDKAMSLHESIDRLNTESLPKDGLGKTRVMLLRSLAENHESLGKCVQIFSSTFGIAVLCILVSCLLHLVATAYFLFLALLNPNVTGYAWGQVLWIFLHILRLLLVVEPCHMATLESKKTIQIVCEIERKMHEPVLVEEIKKFWQQLLVIDVEFSALGLCRINRNILTALSSAIATYLVILIQFQKASG

>BdorGR93a

MSKYNRTELFAYRLLRVLYKYGHFLSVFGWKLQKQKMELRQGKRWIRIIRIIWRIWLSLIFASLMPKMMAPYVRYIGNSFLMFFADVQVTTVTLFSILSFVIHECSERKIFQIINKLVNMYERISTKSGFEQILGRTFVISIILKFLLSAFGLMYEIPLLVEGTGLMSFLAGIYLWLSTIYILDCCFVGFMVIRQMYIAMATYLERMLERMSRIESEEPQQRLSKHQRMKELCLCSESIDDSNNIYSVLYELTKEFHHIFRWQVLYYIYYNFVIILMLMHRFISRYIETGAVDLMAFFSTVFKFCNLAFLIFSTDGVVLKSQLTDLLNLDLVCSDIDARWDESVETFICQRKVENLEIKVLGFFHLNNEFILVILSAIMSYLFILIQFGLTNSG

>BdorGR22e-like

MCSETTLRVLRQTRQIVADTCISTQFLLSTVHGLFPFKYDSGTRRLTSTKLLNCYWPLVNIAIVLITPYIYFLKPQVNQVRFIYDKPLNKLLAHVHYMLGVCILLVIIIANSYHRKELFKLHNDLVQMQRRQQRWQQKWTVKSNSQIEAFYYNAIIAKSLMSLLQVASNVGGKLTMNTNPSLEYFIYVFLVLVLKNVTLLTVANFHFALLNIYRQLQQVNWNFQEVLRLWSVRAPAVSNIPLEHVTDIAFAAFAGEAKQPNGSRNFGVSAIADLCGQYVQICRLARRVCKHYEWQVLLFLIIILFGNVMSTFYFLVYLGGKVVPKELFSPTLFLQFYLINILDLYCYMLICERSMASSKETGFLLKELTQLKSLPKGLQHEFEMLSIFMAGETVRFRFCGLLEWNFRTGASYMTATILYLIVLVQFDYNNL

>BlatGR64f

RKWPALMLDWYEIECDLPEYLTQMEKGRLAYRINMVTVVAMTLSLAEHLLNILSNIHYSNYCPQTEDPIENYFILTNQHLFMIFSYSVPLAIWGKVQNILCTFIWNYMDVFVMIVSIGLAAKFRQLNDNLFKFKGMRMPEMYWSTRRKQYRNLCELCTRIDGAISLITMISFSNNLYFICVQLLRSLNKMPSLAHVAYFYFSFFFLIGRTLAVSLYSASINDESRKPLRVLRCVPKESWCTEVKRFSEDISTDLVALSGMKFFYLTRKLVLSVAGTIVTYELVLIQFHQDSKIAECVSSIRTLSSSG

>BlatGR64e

LSKTAAKVYFSWKSVRTCYAMLVIFCLGPASLCTIAFAFRERFNFDTVEAIVFYVSIFLIAMAFFQLARKWPALMVKWESIESKLPPLKTEMQRAALAHRIKMITLVATMCSMVEHLLSMLGIIYYVNACPTMPGHPIRSFLYTNWSQYFYFFDYTDWAGIFGKVLNVISTFAWNFNDIFVMAVSVALSARFRQLNEHMLRVAKRPTSEKFWIENRINYRNLCKLCEATDDTISIITLLCFSNNLFFICGKILKSLQKKPSFSHTMYFWFSLGFLLMRTLMLSLYSAEINEESKRPLVVFRSVPSVSWCRELKRFSEEVTTDVVALSGMKFFHLTRGLVLTVAGSIVTYELVLLQFNKEDKVNDCYEG

>BlatGR22d

MHTARRKFANFIHCYVFKISMLLGLLPFKYNKEKRTFRSSRMALSYSVVINTLLFAWVVKNWPDMSNYELFVAKPLFFIVQNAVNYMNFVGMIIIFCSTWAGRKRLLRLFNEIVTVRHLCDSKAFLRDFDASTLENRIILKFCSMFLQNLLFFSSTLYFAYRMDESYFWVLMLSIILLNEIFLISNQFYHNTFSINHLILAVNHRLRCLNTRKQLLAAGKINEIFGIYIRLIRLIAHTTKAYEQQLLVIISVRLSIVVHCLFSACMQFGSKGLQVNILELLYYLHVIMLTSLDYWLLMAVCESVWRAQQQTEVELKHFNTFRTLSVDLARDLNTFSIFCXIHKFRFPLCGLFEINFATARNLCTSVVTVLIWLVQYDFATILFKDDIPIIAIPSSIPK

>BlatGR28b

MDGDTNGIEAQEVRATRPQRVSGLRRFFQAQQLYESVQPLFVITFWHGLTPFFIKNDGAGNKKLKESIFGYMNTFLHITIYVACYVLTLMNDFETVAGYFFNTGVSRFGDTLQVLSGLIGVTIIYITAMLPKHRLEYSLRTVQNIDLMLHKVGVKIIYTKLLHYSYFSILLVFAVDTVYSCGNFMLLKSANLEPSTPLYIVFTLQHTVISIATAMYHGFVKMLEMRLTMLNKVLKNLAHQWDNGIVKPLPKQHSLQCLDSFSMYTIVTNNPCEIIQESMEIHHMICDAASTANKYFTYQLLTIISIAFLIIVFDAYYVLETLLGKSAHESKFKTVEFVTFFSCQMILYLIAIISIVEGSNRAIKKSEKTGGIVHSLLNKAKNAELKEKLQQFSLQLLHLKIHFTAAGLFNIDRTLYFTISGALTTYLIILLQFTNSNPPEHPFVPMEENDTMPIRSLVSNLTIGG

>BlatGR63a

MFNSYNRRKKHDTVFLNVKPTFNSQGNGLRKYSTGLLDKEDNPFYDVNSSSGSRASVGTITTLNENFRANIFYNNIAPIQWFLHMLGVLPITRREPGKAKFRINSIAFGYSFAFFILLSVFVTYVAKNRISIVTSLSGPFEEAVIAYLFLVNILPLILIPILWWEARKIAKLWNDWDDFEILYYQISGHSMPLNLRRKTTMIAVILPILSIVSVVITHITMADFQIIQVIPYCILDNLSAMLGAWWFIICESLSMTANILGERFQRALRHIGPAAMVADYRALWLRLSKLTRDTGNATCYTFTFINLYLFFIITLSVYGLMSQLSEGFGIKDIGLAITAIWNVFLLFYICDKAHYASFNVRTNFQKKLLMVELNWMNSDAQTEINMFIRATEMNPSNINCGGFFDVNRNLFKGLLTTMVTYLVVLLQFQISIPTDTGRHMNVSVAELATDMMLESAEVELTTTSTTSTTTTTTTKMPPPARGKKG

>BlatGR28b-1

ARVKMLVVRRTFANLFGAHEYYKSIEPLLFFTHTLGITSYVVVSNERGFKSLTNSRYSYVVIFLTLSLFAYCCVYIVYCKVTYIGHFVNTDITNVGEMAIVLASGLEAVIVLITNAIRRRRLLWVLENFPKIDKSFERIGIRWNYRNLLKNVIWRLIFTSFIYSINCIIYISCWIRLQALPSWQLIFIVLCQQIGICFSLCLFSHLMMSTKLRIKALNKVLKNLAHQWDNGIVKPLPKQHSLQCLDSFSMYTIVTNNPCDIIQESMEIHHMICDAASTANKYFTYQLLTIISIAFLIIVFDAYYVLETLLGKSAHESKFKTVEFVTFFSCQMILYLIAIISIVEGSNRAIKKSEKTGGIVHSLLNKAKNAELKEKLQQFSLQLLHLKIHFTAAGLFNIDRTLYFTISGALTTYLIILLQFTNSNPPEHPFVPMEENDTMPIRSLGSNLTIGG

>BlatGR21a-1

MAYWAIATRKGQSPPMKITPVLNPNQREFLEDELLYREKLEILAENNTISTDLFVRKFEDIDDPVLLDKHDSFYHTTKSLLVLFQIMGVMPIHRNPQKPGMPRTGYSWTSKQVFWAVCVFSMQTTIVVMVLRERVNTFLNDSDRRFDEAIYNVIFISLLFTNFLLPVASWRHGPQVAIFKNMWTNYQLKFLKVTGSPIVFPNLYPLTWSLCFFSWGVSIAINLSQYYLQPDFKLWYTFAYYPIIAMLNGFCSLWYINCTAFGTASRALSASLELTLMSDKPAKKLTEYRHLWVDLSHMMQQLGRAYSNMYGMYCLVVFFTTIIATYGSLSEIMDHGATYKEVGLFVIVFYCMSLLYIICNEAHYATQSVGLDFQTKLLNVDLTAVDSATQKEVEMFLMAITKNPPIMNLDGYANINRELITSNISFMATYLVVLLQFKITEQRNYSLKQSRPELLA

>BlatGR39b

MEECLRLWLRGCAVFGIYVIPIQEHYTAPHWQRLSRKRIKRQLAWTEERATRLTYLLQRLYLATLAVIVCVLYLHGLYAREIEHGFVLTWLVATLVYTSQVLTHLSIFIAALWKREQHESFLQLLQQIDVSLKLRLKCNTRQSALLHSLRLLLFSLILLSVVGICVFTVVSVWLNDIGYYWHAAWTIVTLRVRILQLLIYARILRHYLDCVCVKLRQVVACRTSSASQLLDINYERFESLEFLLAIKENYTLIFKAVQLFNDFAGWSLFGIISSYMLDFTCHVYWSLLGLDGYGSPYTYLVGMPAALPFSVIVCHLCYVCGNCKQLGAIITDLVSKLATVRSTPAMKRYSCVVYQFSTQLQLQRIEMTAQHFFVLDLHLIMSISTAIATNLVIMIQFLKSENSESSGA

>BlatGR93a

MPKYYRTELFAYRLIGVLYKYGHFLSVFGWKLQKHKMELRQGKRWIRIIRIIWRISLSLNFAWLIPKMMAPYIKHVDNSFLIYCADVQVITVTLFSIMPFVINECSERKIFQIINKLVNMYERISIKSRIEQILGRTFVVSVILKILLSAFGLLYEIPLLVEGTELMSFLAGIYLWLSTIYILDCCFVGFMVIRQMYIAMATHLERMLERMSQIESEEPQQRLSKHQRMKELCVCSERYIEMGYVDLMAFFSTVFKFCNLAFLIFSTDDVVQKSQLTDLLNLDLICSDIDARWDESVETFICQRKVENLEIKVLGFFHLNNEFILVILSAIMSYLFILIQFGLTNKHTVVSMT

>BlatGR66a

MQNGAAAPAELDVVLGIYPYDLQKFYYQGILQPSRLGSCVVVTIMCITFMLFNLSLFNFSDEDVITKNNHLSIIVTIVFTYITPATMFTDQIMALRNQRRLPELFERIDCVDEDLRQLGITVDNRRVQRGIWLMITFTFFCEFFIFFSSIWFLVDELRWTTVLWIFISLPTFYNTLDKIWFLGILLGLRDRFDAINAELERIAEELERRQNQQLKGEPYQEHDLVLQTSLPLRTEQIDIKLERLVRNAFGESLLEPVPIRNYMLNISHESSLYTFKALQERFISLCQLHDSTCRIAKLLNELWSYPILILMAFGFVVFTSQLYFVYCATQPDRTIPLIFRCAKEPSISTVFLTYIGGKCVSLMLYSWKTSQAARRAGICLHKCGVAADTNEVYEIVNHLSLKLLNHAINFSACGFFTLDMGTLYAVCGAITSYLIILIQFDMAAQQVRISKELAAANETTAVALVTENYTIGMMESTTPCD

>BlatGR43a

MEITEPTLCVYYVSKALALAPFSVRRNSKGVLDIRRSVMFSVYSASLCLLMVFLTYQGLLFDANSQVPVRMKSATSKVVTALDVSVVVFACSAGVGCGLWGYRATLELNTRLRKVDDSLHSFSNFKRDRILAILLLGLPLIAITSILGLDLSTWLRFAIEMRTPTDDTELNVQWYIPFYSLYFILTGLQINFANTAFGLGRRFRRLNVMLRNSFLKDVDNSQKYVPLKPLITTVKVVSQHPLALHQSLSKLTHVPTQDSAKNKVALLRLLEENHESLGKCMRLVSNSHGVAVLFILVSCLLHLVATSYFLFLELLSKKDSGMVWLQVLWIIFHSLRLILVVEPCHLATVESKKTIQIVCEIERKIHDPILTEEVKKFWQQLLVVDVEFSASGLCRVNRTLLTSFSSAICTYLVILIQFQN

TNG

>BlatGR5a

MHKSYLNYTFLKQIVRYLKAKEITELEKDDGRKYSVKRFLTGQPQQRRKRLQIINGILXGPRYLSKRQTTIAVDEKQELTKELYEQSKHPKVRGIRRGTRADFIHNGSFHEAVGPLLVIAQCFCLMPVRGILAASPKGLSFRWKSFRTWYCILYTLVTIADTGLTVNMVVKGVLDVRNIEPLIFHANILLASIGFLRLAAKWPQLMRKWQRVERHMPPFQSWREREALAVRVHKVTFVLITLSLTEHLLSTISAIHFANYCPSRVDPIESYFMTVVSQIFFIFDYSTWLAWFGKILNVLMTFGWSYMDVFLMIIGIGLSSLFEQVQXSLERVKGQVMPESYWTRTRLQYRLICDLIEKVDSAISAITMLSFANNLYFVCIQLLKSMNTMPSVAHFVYFYASLCFLMARTLAVSLYLSEVNDRSREPLKIIKKVPKEGFHPEVDRMAYEIGMDTVALTGLQFFNITRGLVLTVAGTIVTYELVLIQFHEDQ

NLWNCN

>AludGR63a

MFNSYNRRKKHDTVFLNVKPTFNGQNSGLRKYSTGLLDKEDIPYYNTNSSQGSRASVGTITTLNENFRPNVFYNNIAPIQWFLHIIGVLPITRRAAGKAKFRLNSIAFGYSLVFFVLLAIYVFYVAKNRIKIVTSLSGPFEEAVIAYLFLVNILPLLMVPILWWEARKIAKLWNDWDEFEILYYQISGHSVPLNLFRKTTMIAVVLPVLSILSVVITHITMADFQFIQVIPYCILDNLTAMLGAWWFIICESLSMTANILGERFQRALRHIGPAAMVADYRALWLRLSKLTRDIGNATCYTFTFLNLYLFFIITLSVYGLMSQLSEGFGIKDIGLAITAIWNVFLLFYICDKAHYASFNVRTNFQKKLLMVELNWMNSDAQTEINMFIRATEMNPSNINCGGFFDVNRNLFKGLLTTMVTYLVVLLQFQISIPSDSGKQMNVSVAELVTDMFVETTELTSTSTTPTIKTTTTKMPPSARGRKG

>AludGR64f

MKFALRIGWKIGNVTADARLRVQQQQQARKKQRLMRRQYERGTIRRLSQTVTNEPAACGSLKLKHNRKSALIKRTCTEFVAQMELLHLQIDRPPKVLAKSVKENFQHDGSFHQAVGKVLLFAEFFAVMPVKGVTASHPKQLSFSWTNIRTLYCLLFIIMTVIDLGFSINKLLHKPINFNSVEPLIFRSSIIVVCISAISLARKWPALMLYWYEIESDLPEYLTQMEKGRLAYKIKMVTIVAMMLSLGEHLMNIISSIHYSNYCSQTNDPIEDFFLLTNQQLFLVFPYTIYLGIYGKIENILCTFIWNYMDCFVMLISLGLAAKFKQLNDNLFKFKGMQMPECFWSTRRKQYRNLCELCLRIDGAISLITMISFSNNLYFICVQLLRSLNKMPSFVHAAYFYFSLSFLIGRTLAVSLYTASIHDESRKPLRVLRCVPKESWCTEVKRFSEEINSDLVALSGMKFFYLTRKLVLSVAGTIVTYELVLIQFHQDSQLGDCASIVRPLPSSSNGSAH

>AludGR66a

MAQQTVKPILLHFGQLFLLCKLLGLYPHNLEAFRHKHALHSSKIGTAVVIATMFAINIFYNLLIYSFSEEDHALKASQSTLTFVIGIFLTYIGLVMMMTDQWSAIRNQSKIGELYERIRAVDEQLLRENITVDNSAISKRILIMIGLTVACELTILVSTYVTLVDYTEWISILWFFSGFPTFYNSLDKIWFAITLNALRQRFFRINTALDEMVAFYEKRGFRCGKDTTSLKTSITHEDFGQSLEYLYKELTHMEAIKRPSYGKNKISPVAHSLNSFGEAIEMPKNQQKLSSQPPTFNMIYETELNNEIDKVEEKLNNLCQLHDEICEIGKMLNELWSYPILVLMAYGFLIFTAQLYFLYCATQRQSIPSLFRSAKNAIITTIYLSYTAGKCIYLIYLSWKTALESKRTGICLHKCGVAADNNLLYEIVNHLSLKLLNHSVDFSACGFFTLDMETLYGVSGGITSYLIILIQFNLAAQQAKDAVHNDDETTSLLPTTGEGNATTILDLFTTAFMPTTHSAIN

>AludGR21a

MAYWAIATRKCESPPMKITPVLNPNQREFLEDELLYREKLEILAEHNTISTDLFVRKFEDIDDPILLDKHDSFYHTTKSLLVLFQIMGVMPIHRNPQKPQMPRTGYSWTSKQVFWAIFVFSVQTTVVVMVLRERVNTFLNDSDRRFDEAIYNVIFISLLFTNFLLPVASWRHGPQVAIFKNMWTNYQLKFLKVTGSPIVFPNLYPLTWALCFFSWGVSIAINLSQYYLQPDFKLWYTFAYYPIIAMLNGFCSLWYINCTAFGTASRALSASLEMTLMSDKPAKKLTEYRHLWVDLSHMMQQLGRAYSNMYGMYCLVVFFTTIIATYGSLSEIMDHGATYKEVGLFVIVFYCMSLLYIICNEAHYATQSVGLDFQTKLLNVDLTVVDSATQKEVEMFLMAITKNPPIMNLDGYANINRELITSNVSFMATYLVVLLQFKITEQRNYTLREG

>AludGR28b

MDEDTNSIEAQEACATHPQRVSGLRRFFHAQQLYESVQPLFVVTFWHGLTPFFIKSDGAGNKKLKESICGYLNTLIHITVYVACYVLTLLNDFETVASYFFHSRISRFGDTMQIVSGLIGITVIYFTAILPKHLLQHSLRTMQDMDVLLRSVGVQILYSKVLRYCYFSLLVVFAVDTVYSCGNFILLKSANLEPSTPLYIVFTLQHTVISIATAMFQGIVKLLEMRLVMLNKVLKNLAHQWDNNGVVKPIPKQRSLQCLDSFSMYTIVTKDPCEIIQESMEIHHMICDAASTANKYFTYQLLTIISIAFLIIVFDAYYVLETLLGKSKRESKFKTVEFVTFFSCQMVLYLIAIISIVEGSNRAIKKSEKTGGIVHSLLNKAKSAEVKEKLQQFSMQLLHLKINFTAAGLFNIDRTLYFTISGALTTYLIILLQFTSSNMPDQQSPQEGQNTTTLMNILQNSTAGP

>AludGR33a

MNLLLQTFSLIFGLVPFNSNELDSNVAVDYSLVVIMPLLYIFCYYYVNFRGINAYQLTDCNSICHLSSILFMHVGCFLYLTIYLLTLLRRKAFYVEFGKRLQEIDELNVACIKVADTSNKLIVPQKKRLFYITWIFVLTAFGFAIFYDVNKVFKYYGPYCLILNMVITFPYVAASTVQGIFISYVSVISERFRILNILFNKINQESDRKNSPLSVLDIENEYRKYNHRGPAVISNLAKSKTKKEIQTGEKSVNSSNNYGSDSAEEDSGDDMYNSGAYDEPRLNDGKTSETNLPRLFKLHDKILSLSVLVNSEFGPQCVPYMAACFVITIFGIFLETKVMFIVGGKNELMDYVIYLYVIWSFTTMMVGYLVLRLCCQTYAHSKQSAMIVHEIMQKKPTFMLGNDIYYNKMKAFTLQFLHWEGYFQFNGIGLFVLDYTFIFSVSNSLKIKKPIMRVLVNVMEKIFQTVSAATSYLIVLLQFDMTAILKSEGLTPKALGSGAI

>AludGR64e

MKVAANITKNWLQRQELRLRHIKLWQRGRKVGSEIVQSKPRSATLKMPKERKQHRRMLQVRNIFRRGTKQDYEHSGSFLEAIGPVLVLAQLFALMPVCGVLSKTASEIYFSWKCVRTWYALLVIACLGPASLCTITFAFHEKFSFDTVEAIVFYVSIFLIALGFFQLARKWPALMLQWERIESKLPPLKTQMQRAALAQRIKMITLVATMCSVVEHLLSMLGIIYYVNACPAMPGHPIQSFLHSNWSQYFYFFEYTNLAGILGKVLNVLSTFAWNFNDIFVMAVSVALSARFRQLNEHMLRVAKRPTTEKFWIENRINYRNLCKLCEATDDTISVITLLCFSNNLFFICGKILKSLQKKPSYSHTAYFWFSLGFLLMRTLMLSLYSAEIHDESRRPLIVFRGVPSEFWCPELKRFSEEVTTDLVALSGMKFFHLTRGLVLSVAGSIVTYELVLLQFNKEDKVGDCYEN

>AludGR28b-2

MDEDTNSIEAQEACATHPQRVSGLRRFFHAQQLYESVQPLFVVTFWHGLTPFFIKSDGAGNKKLKESICGYLNTLIHITVYVACYVLTLLNDFETIASYFFHSRISRFGDTMQIVSGLIGITVIYFTAILPKHLLQHSLRTMQDMDVLLRSVGVQILYSKVLRYCYFSLLVVFAVDTVYSCGNFILLKSAKLEPSTPLYIVFTLQHTVISIATAMFQGIVKLLEMRLVMLNKVLKNLAHQWDNNGVVKPIPKQRSLQCLDSFSMYTIVTKDPCEIIQESMEIHHMICDAASTANKYFTYQLLTIISIAFLIIVFDAYYVLETLLGKSKRESKFKTVEFVTFFSCQMVLYLIAIISIVEGSNRAIKKSEKTGGIVHSLLNKAKSAEVKEKLQQFSMQLLHLKINFTAAGLFNIDRTLYFTISGALTTYLIILLQFTSSNMPDQQSPQEGQNTTTLMNILQNSTAGP

>ZtauGR63a

MFNSYNRRKKHDTVFLNVKPTFNGQGGGLRKYSTGLLDKEDNPFYDVHSSNGSRASVGTITTLNENFRANIFYNNIAPIQWFLHILGVLPITRREPGKAKFRINSIAFGYSFAFFILLSVFVAYVAKNRITIVTSLSGPFEEAVIAYLFLVNILPLILIPILWWEARKIAKLWNDWDDFEILYYQISGHSVPLNLRRKTTMIAVILPILSILSVVITHITMADFQMIQVIPYCILDNLSAMLGAWWFIICESLSETANILGERFQRALRHIGPAAMVADYRALWLRLSKLTRDTGNATCYTFTFINLYLFFIITLSVYGLMSQLSEGFGIKDIGLAITAIWNVFLLFYICDKAHYASFNVRTNFQKKLLMVELNWMNSDAQTEINMFIRATEMNPSNINCGGFFDVNRNLFKGLLTTMVTYLVVLLQFQISIPTDTGRHMNVSVADLATDMLMDSAEVELTTTSTTSTTTTTTTKMPPPARARKG

>ZtauGR21a

MAYWAIATRKGSPPMKITPVLNPNQREFLEDELLYREKLEILAENNTISTDLFVRKFEDIDDPVLLDKHDSFYHTTKSLLVLFQIMGVMPIHRNPQKPGMPRTGYSWTSKQVFWAICVFSMQTTIVILVLRERVNTFLNDSDRRFDEAIYNVIFISLLFTNFLLPVASWRHGPQVAIFKNMWTNYQLKFLKVTGSPIVFPNLYPLTWSLCFFSWGVSIAINLSQYYLQPDFKLWYTFAYYPIIAMLNGFCSLWYINCTAFGTASRALSASLELTMMGDKPAKKLTEYRHLWVDLSHMMQQLGRAYSNMYGMYCLVVFFTTIIATYGSLSEIMDHGATYKEVGLFVIVFYCMSLLYIICNEAHYATQSVGLDFQTKLLNVDLTAVDSATQKEVEMFLMAITKNPPIMNLDGYANINRELITSNISFMATYLVVLLQFKITEQRNYSLKQSRMESPMEE

>RpomGR39b

MLHEWQPLFKWLAIVGLVPYAQRNRNCKQHYEQWQRIYTSALLVANWVLTIYGVYNQPLDDEVLVSNFVSVMVFLSQSLALTICLLEAMCTYRQYFAFMQQSQLIVRLFRQRLQTGLCRWSLRRLQRIKYLLYSGFAYGSLPISMVVISIKYYYGYFWYALGSIFVLRTRCLLAIIYLDYIDFYMQNLNMKLHSVASCRVSKQRLCLDVNYKMLESFDYLLQLKLLFDEIRKLTLMFDDLFGWSVCALFTVIFLDICVNSYWIFLTLSNVFEYYFLYLTASTVLPLIAITTLLCQAGENCKRQSLKTGILIQRLLHSKYKYTNAKLYNDLLFEFAMEIQQDPMKIAVKEFVTVDLRLLMKIFTAIVTYLVILLQFRWTYP

>RpomGR66a

MKKMAQQTVQPILVHFGNLFLLCKILGLYPHDLQAFRRTHTLQSSKIGTFLVAVTMIVVVVFYNLLIYSFSEEDRDLKATQSTLTFVIGIFLTYIGLGMMLTDQCSALRNQSKIGELYERIRAVDENLLRENIFVDISATSKRIFIMIALTVVCELTILLSTYIMLVDHSEWISLLWIFSCFPTFYNSLDKIWFAMTLSALRQRFCMINSALEEMVESHERLQRWMENGADGSAFKKPSFTNVPFDPSLEYLYKELTHMEPVKAFSYGKNRISPIAHSLNSFGEGFETPKKQPKPSSQPPPFNMVYESELNGDIQKVEEKLNNLCQLHDEICEIGKVLNELWSYPILVLMAYGFLIFTAQLYFLYCATQNQVIPSLFRSAKNAIITTIYLSYTAGKCIYLIYLSWKTSLESKRTGICLHKCGVVADNNLLYEIVNHLSLKLLNHSVDFSACGFFTLDMETLYGVSGGITSYLIILIQFNLAAQQAKDAIHNYDDESDSLVPTTAEGNTTTFLDFFTTTFMPTA

>RpomGR64f-like

MKFALKIGWKIGNVTADARLRMQQQQQMRKSQRMWRRQCDKGGTRRLSQSATNVAAIFGPSTPQRDHKALLIKRTCTEFLAQMELLHLQIDKPPKVLAKSEKKKFQPDGSFHQAVGRVFLFAEFFAFMPVKGVTASQPSQLSFSWTNLRTLYCLLFILTTTIDLGLSINKLLYKPINFNSVEPLIFRMSIIVVCISAIVLARKWPALMLYWDEMESDLPEYLTQMEKGRLAYRIKIVTIVAMMLSLAEHLLNIISNILYSNACPQSDDPIKDYFILTNQQIFDVFPYSVYLAICGKLENIICTFIWNFMDVFVMIVSLGLAAKFKQLNDNLFKFKGMQMPEIFWLTRRKQYRNLCELCARIDGAISLITMISFSNNLYFICVQLLRSLNKMPSFAQLVYFYFSLFFLIGRTLAVSLCSASINDESRKPLRVLRCIPKESWCTEVKRFSDDINSDLVALSGMKFFYLTRKLVLSVAGTIVTYELVLIQFHQDGALGECVSNLRPLSSASNGSAH

>RpomGR64e-like

MTSWLHMAIGGNLFALALVYIPKEAKGPPHTPLRNMMHNFRGGLAPIGGDANAHHAQRIVELKQRSATPNMLKQRKRHKRMLQVLLLAQLFALMPVCGVLSKSASEMYFSWKCVRTWYALLVIACLGPASLLTISVAFRASFSFDTVEAVVFYVSIFVISIAFFQLARKWPALMLQWERTESQLPPLKTEMQKAALAYRIKMITLVATMCSVVEHLLSMLGIIYYVNACPAMPGHPIQSFLYSNWSQFFVYFDYTNLAGIFGKVLNVISTFAWNFNDIFVMAVSVALSARFRQLNEHMLRAAKRVAGSIVTYELVLLQFNKEDKVNDCYEF

>RpomGR64a

MISLVLIIGQCFSLMPVRGVGHSNPRQVRFTFRSIQVMITLIFLFASSTFNFAMIKHLAKIGIVNFVCTFVWNYMDLFIMMIGKGIAYRFEQMKMRIHTLLDKEVPESIFMEIRDHYVKLIELLEYVDEDLSGIILLSSANNLYFVCYQLLNIFNKLRWPINYVYFWFSLLFLIGRTTFVFLTAASINDEAKDALSVLRRIPANTWCVEVERLIFQMSTTTVALSGKKFYFLTRRLLFGMAGTIVTYELVLLQFDEPNRTKGLPDLCS

>RpomGR43a-like

MKKSAKIPSFRMKSATSKVVTALDVSVVVFACTAGVGCGLWGLRSTRELNTRLRKIDDSLHSFSNFKRDHILAIVMMSVPLIAISLLLALDISTWLRFAIGERTPEDDTEINVQWYIPFYSLYFILTGLQMNFANTAFGLGRRFRRLNVMLKSTFLKVENSKKDASSMPLITTVKVVSQNPLALHQSLNKFANETMQKDGGGGKESKNKVVLLKMLEENHESLGKCMRLVSNSHGVAVLFILVSCLLHLVATAYFLFLELLSKKDSGMVWLQVLWIIFHSLRLILVVEPCHLATVESKKTIQIVCEIERKVHDPILAEEVKKFWQQLLVVDVEFSAIGLCRVNRTLLTSFSSAICTYLVILIQFQNTNG

>RpomGR10a

MWTSKNNSGWNKVATTPMSSHNNSNYGHIYATLYGLTVINYIPQRPTNTCTHRLAVIYGHTISVLLIVVLPIYFARNISALTEAHDQRGQLLLLVNFANTLLKYVTVVVTYVANFAHYAAIRAVTRRRQQLEDDFNSSLQVQPVYDERPRRQFETMLLFKFGLINAMMAVQVANILYQHCTGAHPVRVYIAVYTFVLWNYTENMADYFYFINSSAMKFYQQLNQQLRQVVREAKLLHYFRMRGQRRGTVPHLCGLLCERLDTLAQRYQQINRLCQDSLTMHQFQILGLIFITLVSNLTNSFILFNLFVKHSQARASPAIVLNALHAIIFYVDTYIVALVSENISLELRNINQTLRQFNQVSELDVRLQQTIECFTLFVTNNHVEVRICGLFVLDRSLTYLTAATALSYFITLVQFDLNLM

>RpomGR47b

FKWIYIILYYAGCLCFQPARSALQLTRFNIAYTHFIRIVILLGFIWSVTMKISSNEGSKAMIGHLSPVLKFVLIMECFISAIAFTVSSATMERKKYKHLQLTKSFQDLDNRMENDFPNINWNYYKTERKFTTFTICVIVYFYMIAFIYVYKLSNCSCDYATTFVLAFSHATCTAAPSCIIFLNIGMMDLQRIRYRLIQRLLKQQYTSGGDVRRQAEFQFRLARLIDYCKSYIQLILQINDVFGVVGGIDLFHDFVVLTNLTFLMCQKATEPNTRLKEYVFIFLFMLPRIYKVTIYAVYGYVTNKEQTKCIHEVKMCENYFRGSKAIKNNLDTFIHWQMQNTYSLLVGKATFCNLTILYLIVKSILSSVLILIQLQLQQNSITNRMKNHGMIKDVEFIYGKS

>RpomGR22e-like

MHGVNTYRLRKRISRVLIKATFVLSICFGLLPFYYNQQQKRFTTSKSITIYNIFINILFASIMVFVLQIFSNAEDPFQTRDPLAELLNQMVTYASFCAILITWWINWTGRKELQKLFNEFAAIDADYCYRYPNLVNECAHFDSYLIWKGASILLQNASAFANMWLLVDAGWLFILCICLATVLTNVLFLVMTHFFIAVLYTYRFAWVSNMRLQIMADSGNEVQQRAQCRLVSLEIDAIAHTYLRLIRLCEGYTKIHQYQLLLVVGCITACNIEVLFYMRLLWGGKAYEMDASNIFGIIQIFFVNILDFWLTMTICELAVVTSQKTLQILRVFNEKWDLAVPVERSLERFALICCSKKLRFHLCGIFDINHLTGLQVLFTMILYLIYLVQYYHDNL

>RpomGR93a

MCIILKLILSGLGLIYEIPLIFESGVSGSLLAGVYLWLGRIYMLDCCFVGFLVIRQMYVEMAAHLAQMIDTMRIIDAEQPPHRRLTNHQRMKLLCIHAESIDESSVIYTSLYKVTQRFYHIFRWQISFYIYYNFVIILMQLHQFILRYFHTGYIDLLSLFSSVFKLCNLVLLIMCADAVVAKSQLPDSLDLDLICSDIDERWDVSVETFICQRKVENLEISVLGFFHLNNEFILVIISAIMSYLFVLIQFGMTKNSVAAGHAYPHHTLNHS

>CstyGR64e

MKIAKNKIYLNSPKRRLMEIKFWKTHKVDAVENDSLYYRIKSANMTQNQRKSKIFKLKHKLLRRGTKKDYTHTGSFQEAIKPVLVLAQIFAIMPLAGVTSNSAYDLKFSWRFIRTWYSVLTLISFAFFMGITVAFAFSGV

FNFDSVEGLIFFSSNFFIALTFFNMTRKWPALMQQWQQVEHGLPQQRTIVDRSWLAHKIKIITLVATVCSLGEHTLSMLNIIYYVNRCPTFKDHPIDSFLYTNFSQYFFFFEYNTLAGILGKIINLLSTFAWNFNDIFLMCVCVALASKFRQLNVYMAAHLKKATPATFWMECRRNYRLLCRLNEAVDNTIAGITMLCLSNNLYFICNKILKSLQKKPSITHTLYFWYSLIFLLGRTFTMALYAAEINEESRKPLVIFRKVKREFWCPELKRFSEEVNADMIALTGLKLFNLTRSMVLSVAGTILTYELVLLQYNKQSVIGDCAAN

>CstyGR63a

MWNNYNRKKKQDAIFLNVKPVATNISVRKYSNGLLDRLDSGFGNYNKEKRTSRQSISTIDSMNQQFLPNVFYRNVAPIKWFLSMIGALPMKRAGPGKAKFVIGSIAFAYSIVFFTFLMIYVAYVANNRIIIVTSLSGPFEEAVIAYLFLVNILPIFLIPIMWWETRKICALVNDWDDFEILYYQISGHSVPLNLRGRSRTIAIALPILSILSVVITHVTMADFDLIQVVPYCILDNLTAMLGAWWYLICEALSTTANILAERFQKALRHIGPAAMVADYRALWLRLSKLTRDTGTATCYTFTFLNLYLFFIITLSIYGLMSQLSEGFGIKDIGLAITALWNVCLLFFICDQAHYASFNVRTNFQKKLLMVELNWMNSDAQTEINMFLRATEINPSNINCGGFFDVNRNLFKGLLTTMVTYLVVLLQFQISIPTDVRTVNRNITISDDISMSDASMNSDEMDADIMTTIMTTLAATTTTAAVPPVRGRKG

>CstyGR28b

IMKFRHSKLVMVTIVCVGYLSSSLWMLFHNQIWPSFQAVVAFFVPHIFLLCVVVLNVSFVMRFWQHFDLLNKVLKNLCHQWDTRSIKTITHKQRSLQCLDSFSMYTIVSKNPAEIIQESMEIHQLICEAASTANKYFTYQLLTIISIAFLIIVFDAYYVLETLLGKSKRESKFKTVEFVTFFSCQMILYLIAIISIVEGSNRAIKKSEKTGGIVHALLNKAKSADVKEKLQQFSMQLMHLKINFTAAGLFNIDRTLYFTISGALTTYLIILLQFTSNSPSVTQNGCDPPLAVTANHTQT

>CstyGR21a

MAFWASVSTKSPLKIAPVLNPNQKQFLQDELRFREKLDILARSDVNNLTDYYVRKHETIDDSDLLDKHDSFYHTTKSLLVLFQIMGVMPIHRNPPKSNLPRTGFSWHSKQVLWAILIYCIQTTVVVLVLRERVNKFVVNSDKRFDEAIYNVIFISLLFTNFLLPVASWRHGHQVAIFKNMWTNYQLKFLKVTGTPIVFPNLYSLTWALCICSWALSILINLSQYFLQPDFELWYTFAYYPIIAMLNCFCSLWYINCTAFGTASKALLNSLKTTLRGDKPAEKLTEYRHLWVDLSHMMQQLGRAYSNMYGMYCLVVFFTTIIATYGSFSEIIDHGATYKEVGLFVIVFYCMSLLYIICNEAHYASQKVGLDFQTQLLNVNLTAVDTSTQKEVEMFLVAIAKNPPIMNLDGYANINRELITSNVSFMATYLVVLLQFKITEQRGIRSQQAMASM

>BcucIR1

MKLFLKSAKAKTKATKTTHKYVLNMCMPSMQQRSLPTHQHRVPRCCWRVATRTPECFNNKIKMALLILIWTAIWCRRECVAVERHSNQAVYANNNNKSNNISNLHEQLEINAVDDNVDVEVLLSNVEDELFRVVAPIDLVDEGSLEQVLFKRDTAKATDGVSEELNATTGYEFTTLDAAMTGVADTKRMMTQQITEIQTETETTTTANDINSVATTMNSSVEINEYENVKNNGNENVERAKVVDRKIIAATTTRTAATALFAESVSATAAATPQTGEQQNIAAEGRESEKEEAIAPEMQTDDGEEPVDVTAALQQQLIYEFAIKYKHIPRVTYFTCRWQSSEGSGGNTFGEQMTYLNMQTLAMMQRMYGSEVVDKMRSEPDTKASQKAKKASAADKVTTKRSSAPITATTARAAPPSRAVETNTPKVRNNGNTRSKRTTTAAVTRAQTAAALLNNGNVTQSGKGSNRASRVTSDASAATTTAQWDILIKVVHIDQLINKRPTAANNKNGKNNRNYNWNVQRTFGGGFAAGGGYGRSAAGNAGARNAYTNANWLAQVLRDDGSRQLVVLNLACGAASRRLLEMASNKALFNATYHWLLIEDYTFNRNADIDDNVDNDRNGNNNGNANCKRTSAHQYNETVDSDKNSNKNKFSEQTETQQLQESKEEIEKKKQQQLTATATTSITPHPKATLTAVAALTVTANNENTMEIIEKYLEKLNININTELILAKRHTRRIRDDAAQIGDNKKCAATNDDAGDGGGSGSGGCCTSSDSESATNNTLQRKDYYQLYDVWNPGLQYGGQ

LNISEIGYFALDDGLQIALWYRRSTTITRRMDMKMARIRCLIVVTNKNHTDTLEHYLTTHYDTHVDSMNRFNFALLSSVRDLFNFSFVLSKTASWGYLKNGKFDGMIGALVRKQADIGGSPIFFRIERAKVIDYTTRTWVARPCFIFRHPPSTKKDRIVFLQPFSNMVWILLGLCGIFTICLLWLLTSVERRLEAVGVVEQLGHSTRSSSGSNATTTTANNKQLCAIGGNDELPPPVGCRCSTRMPLSAGTMPSCGACNAPSVGLATKEQRKQKLEKLMQRRGKTEARRKKVHGISCRRSMLGCGSACCGQTGSVAAQQRTGLFFESVLFYVGSICQQGLTFSTSFFSGRCIVITSLLFAFAIYQFYSASIVGTLLMEKPKTIRTLRDLIHSSLAVGVEDIPYNRDYFLRTKDPIAIELYAKKVTSVPTDNEALSETTADNVTTLSPLQPGVELTAAEKAKTYREILHSHETGAHAKTNEASNWYDPEYGVKRIRKGKFAFHVDVATAYKIIADTFSEKEICELTEIQLFPPQKMVAIVQKGSPLRKTITYGLRRVTESGLMDYQRKVWHSPKPRCVKQIHTDDLRVDLQTFASALLVLIFGYAVSLLALSIEIIQHKLWQRYRAEQEDEQEYE

>BcucIR2

MKFLLLHITCALLYASAYAADERPKFNVGIIFASKNDETEIAFRTAIERANVFERSFELEPIVEYADTDDSFMVEKTVCKLIAQGVIAIFGPNTAGGTDVVTSICNTLDIPHIVFDWTPSEALSNRQHSSMTLNVHPNNILLSRGLAEILQSFSWRSYTIIYETERELQQLQDILQVGEPSSNPTSIRQLSEGPDFRPFLKNIKLSTDNCIVLHCSTDNVMKILNQANELKMLGEYQSVFVSVLDTHTLDYQELLTVTANITTVRLMDPTDYQVKNVAHDWEEHEKREGRYYRSDPSQVKTNMILANDAVTMFVKGLAELGIAEELNPPKLECRKNRAWAHGRRIIEFLKARSVEGATGRVDFNEYGERNFFTLRFMELTPAGFLDLSTWDPVNGVDALEKEDASEKRVGEKLSNKTFIITSRIGAPFLLNREPKDGEILQGNARYEGYSMDLIDAIARLLNFKYEFVLAPDGKYGSFNKLTQSWDGLVKQLLEGNADLGICDLTMTSTRRQAVDFTPPFMTLGISILYAKPEQPPPDLFSFLSPFSLDVWVYMATAYLCVSLLIYGLSRMAPADWENPHPCKEPEEVENPWCMSNTTWLAVGSIMGQGCDILPKAASTRLVTGMWWFFALMMLNSYTANLAAFLTMSRMESSIESAEDLAAQSKIKYGALLGGSTMGFFRDSNFSTYQRMWTAMETARPSVFTKNNDEGVDRVLKGKGRYAFLMESTTLEYIIERNCELMQVGGWLDYKTYGIAMPFNSPYRKQISGAVLKLGESGMLSELKRKWWKEMHGGGSCSQTESSGGDTPELDLENVGGVFLVLGIGLLTAILIGMCEFLWNIKAVAIEEKISLSEAFKAELMFALRFWIQTKPVHTASTSSGGSSSSSSSKSTKSSKSSSSSSSSASKRSKRSSKSYARSISQSTKSITNATHDPDKNDLSVHDKLRKISSMFSLKSARSEPSVANVHLEAPPALKHNSHQSHNPVNKSTHTPMVREVAQQTTLTTDDGEEEQAPQLKIDEIPIVEPHHHHNHNHHHHHHHQKHHEHNHQQPEEDHQPAIPLIERTQNGKVSRNGHAGFGNAEV

>BcucIR3

MLNHNLNVSSRRFELQAYVDVINTADAFKLSRLICNQFSRGVYSMLGAVSPDSFDTLHSYSNTFQMPFVTPWFPEKVLTPSSGFLDYAISMRPDYHQAIIDTIQFYGWRKIIYLYDSHDGLLRLQQIYQGLKPGNESFQVEMVKRIANVTMAIDFLHTLEDLGRFTNKYIVLDCPTEMAKQILIQHVRDISLGRRTYHYLLSGLVMDDRWESEIIEFGAINITGFRIVDTNRRFVRDFFDSWKRLDPATSIGAGRESISAQAALMYDAVFVLVEAFNKILRKKPDQFRNNIQRRGQQTLMAAASTSLNGTMGMAVGGGVGGIGNGIGGSGGSGGGGAGIGNGNGGNGGGGTPRALDCNTSKGWVNPWEHGDKISRYLRKVEIEGLTGDIKFNDDGRRVNYTLHVVEMTVNSAMVKVAEWSDDAGLQPLSAKYVRLRPHVEIEKNRTYIVTTLLEEPYMMLKRPGIGEQLDGNDRFEGYCKDLADLLAKKLGINYELRLVKDGTYGSENPTVRGGWDGMVGELVRREADIAIAAMTITAERERVIDFSKPFMSLGISIMIKKPVKQTPGVFSFMDPLSQEIWMSVIFSYIGVSIVLFFVSRFSPYEWRIVQYQTDSHAHHDQMANQQPPGIIGGVPVPGPMTGATSATASPANIGVVSAGGVAGSVGLGSSGNVAVNEFSILNSFWFALAAFMQQGCDISPRSISGRIVGAAWWFFTLILISSYTANLAAFLTVERMVTPINSPEDLAMQTEVQYGTLLHGSTWDFFRRSQIGLHNKMWEYMNSRKHVFVSTYDEGIRRVRTSKGKYALLMESPKNEYVNAREPCDTMKVGRNLDTKGFGIATPIGSPLRDPINLAVLSLKENGELIKLRNKWWYDKTECNLNKDNQETSRSELSLSNVAGIFYILIGGLLVAVFVAIIEFCFRSKASVQKANGSMLSSASGGGSHQRNSLTDAMHSKAKLTIQASREYDNGRVGYLNCASLQYYPAGQLSAASEAETMHMNAHSQV

>BcucIR4

MAVLDGMSVVFLLFCGIHLGVTAQKHTQHSDNPSTYNIGGVLADPESESHFRTIISNLNFDQQYVPRKVTYYDKTIRMDKNPIKTVFNVCDKLIEKRVYAVVVSHEQTSGDLSPAAVSYTSGFYQIPVIGISSRDAAFSDKNIHVSFLRTVPPYYHQADVWLEIMFHFGYTKVIIIHSSDTDGRAILGRFQTTSQTNYDDIDVRATVEMIVEFEPKLDSFTEHLIDMKTAQSRVYLLYASTEDAQVIFRDAALNNMTEGGHAWIVTEQALHANNTPVGVLGLVLEHANNDKEHIRDSVYVLASAIKEMMSNETITEAPKDCGDSGVNWESGKRLFQYLKTRNITGNTGQVAFDDNGDRIYAGYDVINIHEKQKKYVVGKFYYDPEKAKMRLRINDSEILWPGKQKKKPEGIMIPTHLKILTIEEKPFVYTRRLTDDEVNCDEDEIPCPLFNATDGSENENCCRGYCIDLLNALSHRINFTFDLALSPDGQFGHYTLKNVSSSSSGAITSRKEWSGLIGELVNERADMAMPLTINPERAEFIEFSKPFKYQGITILEKKPSRSSTLVSFLQPFSNTLWILVMVSVHVVALVLYLLDRFSPFGRFKLSHTDSNEEKALNLSSAIWFAWGVLLNSGIGEGTPRSFSARVLGMFWAGFAMIIVASYTANLAAFLVLERPKTKLSGINDARLRNTMENLTCATVKGSSVDMYFRRQVELSNMYRTMEANNYDTAEQAIQDVKKGKLMAFIWDSSRLEYEASKDCELVTAGELFGRSGYGIGLQKGSPWTDAVTLAILEFHESGFMEALDKHWIFHGNAQQCELFEKTPNTLGLQNMAGVFILVAAGVAGGVGLIIVEVIYKKHQVKKQKRLDIARHAADKWRGTIEKRKTLRASLAMQRQYNVGLNANPGTISFAVDKRRYPRMGPRAPEQAWKSDADILRNRRYLDEAAKGGHSPAVHMPILGKMRPPTNMLPPRYSPAYTSNVSHLVV

>BcucIR5

MLLLQKLVILISFSYICANELKIAFWIDPLQSGIELDVASAVKEIKALQLETEIQYYVVVIENTGRQKEEKNMEKLCEHLATDGVSVVIDFTYHIWHNGLDLLRMYQIPFLRVDRILAPYLKMFSQFVLEKSGHECIMIFQNARDTEEAVIQVVEGYPFRTLIMNAFDKQDFIQRLRKIRPMPSCYAIFAEGTAMNSIFERISKANVFERPREWHFIYLDPRDRVFKFKKQVDFATKFTINPKTLCRSLRMKDAYCLSGFSLQRAIILEILRGLIELKQTNLYWLQTFVMECNYTSPNENGTNGFDILEQFPLNEFLYFTTDVRFPNDEFDHVPRLTYSPTININLYSSEHDAVTELAIWQNDNLRKINETISKPRRFFRIGTVEAIPWNYMKRDPKTDELILDAYGNPIWEGFCIDSIKKLSERLNFGYMLVPPTSGEFGRRDVINDRWDGIVGDLVTGETDFAVTALKMYSEREEVIDYIAPYFEQTGISIVMRKPVRQTSLFKFMTVLRVEVWFSIIAALVGSAIMIWLLDKYSPYSYRNNRAAYQYPCREFTLRESFWFALTSFTPQGGGEAPKAVSGRIMVAAYWLFVVLMLATFTANLAAFLTVERMQTPVQSLEQLARQSRINYTVVEGSSTHQYFINMKFAEDTLYRMWKELTLNVTEDFQKYRIWDYPIKEQYGTILLAINGSEPVKDAKEGFRKVNEHENADFAFIHDSSEIKYELTRNCNLTEVGEVFAEQPYAIAIQQGSHFADELSYALLELQKDRFFEDLKAKYWNMSRIKACSVNEEQEGISLESLGGVFIATLFGLGLAMVTLVLEIIYYRRKYSAMHRFNEITKVKPASGTSIKQLLPKKKKKRIAVWHTSTSKRDNSPEHQTPPPAFDAVKFRGKKVPPSITLGGQEFKPRRAGLRQLSESLDSEEYRKEGIPANRDDELPPYTE

>BcucIR6

MRFSNIFGTFLLLIVVKLTHEYTNNGDIEVKVGVLFFRDEYEIELSFDEAFREINANKLFGLTFQLIKRFVPSDDSLLLQQLTCELLSDGVVAIFGPSSKASSDIVAVIANATGIPHMEFDWRIESPTQDRLNNRMTVNVAPSVSMISKAYYSIIKANYKWNKFTLIYETKAGLARLQDLMNIEALDNELIKIRNIEDYKSDLRVLWKEASETLHEHRVILDCEADSLQSLLLIAKDFKMLGAFKYLFLSHLNTHNSPLQSMYNADYKANITSARLKLVEGNPFGRKKTRLRPIDEIFQNQTLLPIIMYDTVVLFANAARNVITKIRTYVEPQRRCDFSYPGRPWHIGRKIVREMKSISEDDVEPPFKTENLKIDENGQRSVFHLEIYKPTTNEILGIWKPDGTISPPTTTQFGSDNAVSAPDFSLGRKMFIVTTRFEEPYFMLREDYEALRGKDRYEGYAVDLIQKLSNIMNFDYEFLVERTTGKLNPQTGEWDGMIRRLIDHQAQIAISDITITQARRQVVDFTVPFMQLGISILYFRRPKEAKNEFAFLEPFAEEVWYYLMLTQLIMTLLFVLLARFSHYEWTNPNPGKPDPDELENIWNNSNSFWLMIGSIMQQGCDLLPKGPPMRILSSMWWFFTLMIVNAYVANLAASLTNNKIPTEFDSLEGLVDQNKVKYGTLAGGSTSVFFSESNETVYKRAWNQMISFTPSAFTSSNKEGVERVRKGNGSYAFLMETTSLSYNVERSCKLKAVGEQFSEKHYALAVPLGAEYRSNLSVSLLQLSEKGELYTLKRRWWKLKEELNCDDSDNTDGDELSIIELSGVFVVLGAGVVVAFIIGICEFLWNVQTVAVDENTTPWQAFKAELCFVLKFWVTKKPANISESTKSTTSSKSSSKRSLDRYRSRSHSHVSGHSRRTTKSKTRDRERERERSVHSRRYYD

>ZcuIR7

MPSAHLKFRKEILIFLKIISLASLATGQTNQNINVFFVNDADNEPAAKAVTVVSTYLKKNPSYGISIQIDQVEANKTDAKTLLEAICSKYAESIERKQPPHVIFDTTKSGISSETVKSFTKALGLPTISASYGQEGDLRQWRDMDESKQKYLLQIMPPADLIPEVVRSIVRKMNITNAAILYDDTFVMDHKYKSLLQNIQTRHVITGIAKEGKREREEQIEKLRNLDINNFFILGNLMSIRMVLESVKPSYFERNFAWHAITQSEGEVSSQRDNATIMFLKPMSYAQNRDRFGRLKTTFNLNEEPQITSAFYFDLALRTFLAIKDMLQSGAWPKNMEYIGCDEFLGGNTPERNIDLRTAFTMVQEPTSYGVFELVTQPGKAFNGYSYMKFEMDINVLQIRGGNSVNTKSIGTWTAGLDSPLVVKDEEVMKNLTADTVYRIFTVVQAPFIIKDEKAPKGYKGYCIDLINEIADIVHFDYTIQEVEDGKFGNMDEKGEWNGIVKKLMDKQADIGLGSMHVMAEREIVIDFTVPYYDLVGITIMMQRPQVPSSLFKFLTVLETNVWLCILAAYFFTSFLMWIFDRWSPYSYQNNREKYKDDDEKREFNLKECLWFCMTSLTPQGGGEAPKNLSGRLVAATWWLFGFIIIASYTANLAAFLTVSRLDTPVESLDDLAKQYKILYAPLNGSSAMTYFQRMANIEQRFYEIWKDLSLNDSLTPLERSKLAVWDYPVSDKYTKMWQAMQEAQLPATLEEAVERVRNSTSATGFAFLGDATDIRYLVMTNCDLQIVGEEFSRKPYAIAVQQGSHLKDQFNNAILTLLNKRQLEKLKEKWWKNDETQAKCDKPEDQSDGISIHNIGGVFIVIFVGIGMACITLVFEYWWYKYRKNPRIVDVIEANSGGKDGKTIDSVILGQAGKEYDKGGNTVLRPRFHQYPTTFKPRF

>BcucIR8

MPPNWTCFLVGVALILCGLQTVYSVPPTEAKVEKIGCVSPTLIRRHNLNPQLYGSCEYGQRNATLPLLQRLRREIKPIFRGHPKPRAEVLANKFHMNAFGSDQTDSLVRLINKIAIEYLHKCPPVIYYDSFVKKSEGMILESLFKTFPITFYHGEINEHYKPINPRLKRRIDSQCKSYILFLSDPEMTRKIIGPQIESRVVLVARSTQWKLRDFLSSESSSNIVNLLVIGESLTETPLRERPYVLYTHKLYTDGLGSNTPLVLTSWIGGALSRPHVNLFPPKFSNGFAGHSFQVSAINQPPFIFRIQSLSRGGTSHTAWDGLEYRLLNMIAAKLNFTIDVIEPPRRPNVKSIIDNIMYQVSTKAADVGMCGLYITDERITETDMSIGHSRDCASFITLASKALPKYRAIMGPFQWPVWVCIVVIYLGAIFPIVYSDRLTLRHLIGNWGEMENMFWYVFGMFTNSLTFSGKYSWTSTQKTSTRLLIGSYWLFTIIITACYTGSIIAFVTLPAFPNTVDSVNDLLGLFFRVGTLNNGGWETWFQNSTHVPTIKLYKKMEFVSNLEEGIGNVTQSFFWNYAFLGSAAQLEFMVQKNFSDDNISRRSALHLEECFALFQVGFLFPRDSVYKRKIDSMILLAQQSGLMNKILNEVKWSMQRSASGKLLQASSANALRERIQEERQLTTADTEGMFLLMGIGYLLGAIALLSEIVGGITNKCRQIVRRSRKSISSAWSSKRNSEDEESLRTAAEQLAHDQRKEAKRKAEKQGFGVREFNLTKKTLQELYGNYYKQEPNYVLKDGKLLLETEALSASSTDCNSRGSSGDVPANRLTHLHSKKKAMLVAEIDVERERERMAAAAEESLAALDACLKMQEDANSSERSDDDDATYEYELFGSLVEPEGPLSTKLNELNLLTDGAALLKALEDESVVVEKS

>BcucIR9

MWKYCLIVTLTCLCVQNVAALPHINIGAIFYESELDLERIFIATVESINSEKVNNFKMVPLVRRVSESDGSMILQRQACDLIDNSVVSIFGPSAKADSDIVALICNATGIPHLQFDMSAEETEAESKNHQMTLNVFPTQQMLSKAYADIVLTFGWTKFTIVYDADDSKALTRLQDLIQLREIHNDVVRVRTFQRGDDYRTMWKSIKGERRIVLDCEPDLLVDLLNTSIEFKLTEQFNNLLLTNLETHNADLEELRGNETFEVNITATRLKMNGNFYYANAWQLASLYDIDPSTEQPKRTLLHDLLYDAVHVFANALRNVSYSYLIRTPRVRCDFSDVSDYEQMQPWPMGRYIYRVMLATSGVNNTDYRTSDLQFDDEGQRTNFGIEIYEPLENYGIAFWDTKGQITPQHIELDSTKKLVYRVATRLGEPYFMLNPEMVGQNVTGNELYMGYAVDLIDELSKLMNFEYIFMPVADNSYGKYDKETKQWDGIIGELINNNAHMGICDLTITQARRTVVDFTVPFMQLGVGILAYNEDEKLELLKFLEPFKDEVWICVIVAIFVISFLFVCSARLADDEWENPHPCNKDPDMLENKWGLFNTFYLTAASIMQAGCDILPKSAPFRTFTATWWIIAVIIPNSYTANLAAFLTSSKMVYDVSDLKSLVEQVDIKFGTIAGGSTYTLFAESNETVYRMAYNMMNNEDPSVYMKNNTEGVNRVIKNNGKYMFLMETTSLEYNTERNCKLSMIGEKFGEKHYAIAVPFGAQYRYNLSVNILKLSETGKLFELKDRWWKGEKNCGDEEEDNEALGFQHVRGIFYTLFLGLFAAYFLGIMEFLLHCHSRASEEKLRFKEVLVNEMRFVLRLWNNRKPVSCTPTASIDASSRRSSNRTTRSLTKKNSRQSSGSGEELRDLPQKKVKKNGSIIKEGTM

>BcucIR10

MARPYRQSMPQFVATTHCTRITLIFLVALSFLQIAATQKTNVGLIYESTNPDMEKIFQIAIDKANEESGGTVELHGIAVAIEPGNAFETSKKLCKMLRQNLVAVFGPTTDLAAKHAMSICDAKELPFIDTRWDFEVQMPTVNLYPHASQLALALKDLVVALEWTDTFTIIYETGEFLPTVNELLEMYGTTGPTITVRRYELDLNGDYRNVLRRIKNSGDYSFVVVGSMDTLPEFFKQAQQVGLMTSDYRYIVGNLDFQTMDLEPFQHGDTNITGIRLVSPEAQKVQELSKTLYETEDPFQNVSCPLTTSMALVYDGVQLLAETFKHVMFRAVPLNCNDASSWDKGYTLVNYMKSLSLTGLTGEVKFDYEGLRTDFALDVIELTMSGMQKVGEWKTEDGFVANRPPPKIVEQDQRSLVNKSFVVITAISEPYGMLKETAAKLEGNDQFEGFGIELIEELGKKLGFTYTFRLQVDNKYGSFNPKTGKYDGMMLEIIEGRADMGITDLTMTSIREEGVDFTIPFMNLGIAILFRKPMKEPPKLFSFMSPFSGTVWMWLGIAYLSVSLTLFILGRISPTEWDNPYPCIEEPTELENQFSFPNCLWFSTGALLQQGSELAPKAYSTRTVASIWWFFTLILVSSYTANLAAFLTIESLSSPIENAEDLAVNKGGVKYGAKVGGSTFTFFQDAKYPTYQKMYEFMRDHPEYMTSTNAEGVDRVENENYAFLMESTTIEYITERRCSLTQVGSLLDEKGYGIAMRKNWPYRDTLSQAVLELQEQGVLTKMKTKWWKEKRGGGACSLEGANDGAEELGIANLGGVFFVLCVGSVFASVYGLLEWLCHVYGTARRNKVSFKTELIEEFRFVMQCSGNTRPVKYPKNSSRSRSRSSRSRSRSHSRSSSQSSTLSVDSLPMDESKLHHISEHTKHAK

>BcucIR11

MRVLLYVLLSFVFFAQTAQFTKDFQVVKVGVIFFHDEMELVQAFDTAIRNINELELDVRLEPIKHYVSYDDSLTLQEFACELIERGVAAIFGPSAKASSDIVEVICNTTGIPHLQFDWHQEDVHIHSRNHKLTVNVAPAENMLATAFLDILRLKELDWKSFTIVYENSRSLGRMEHLFGWRQVHKTGIKLWRFNRGDDYRTLWKLVCSTREKNIILDCPADIITEVLNASIYFNMTGQFNQWFLTSLDTHTSNIHSLDRHNFMATIVAVRVRSYMPPPVHDETDVFEDEEEDPINTIRAKLLYDAVVLYFNAIRNQIRSSRYQEPRVRCQRGYWRSGMDILDQMKLLTSRNVTPPYKTQKMQINTYGTRDEFNLEIYNPLFERITYIWNKNKNLTAFEDLVEAKEIKKQKFKENEDFTPKRVKYTVSTRFGEPYFMWRKEPEGVHYEGNERFEGYAVDLIYALAEECNFDVIFQPVPDNKYGSYDKEKDEWNGIIRQLMDNNAQIGICDLTITQARRTVVDFTVPFMQLGVSILFYEEPPPPKNLFGFLSPFSLTVWIYLLVAIMVTALVLVVMGRFSHRDWIGAIAHDPNPTEVENIWNLKNSLWLNVGSILNQGCDLLPRGSAMRIFTAFWWIFSLLLSQTYIAQLAAFITTSKMDSSISSLHDLINQNKVQFGMLKGGSTQFLFSESNESEYRVAWNKMIGMKPDAFTASNKEGVDRVKRSPGRYAYLLETTALQYYLNRNCELKQIGEPFNEKHYAIAVPLNAPFRSNLSVGILKLSEKGELYKIKNKWFTTNETDCNAEKAANANEDGTYTMESVGGLFIVLLAGILVSIIIGIFEFLWNVEQITVHDNVPAMVVLKAELKFFLRFWQTRKPLRTYAESRGSTSTGYSSFDQTASTSTVKKKKKKKCKKCSD

>BcucIR12

MHIQNNWIIGVLYAFIFHVNGFINIKENEGIAVGIITDHNTEQIRKTFEYAINVANTELGVPLIGYNEEIKFGDSIEGYTKICKFMQNGIGAIFGPSSRQTSAHLLTVCDAKDVPYMYPHMSDHVEGFNLYPNPLDLARILHNVIDLFKWTRFIFLYESSDYLSILNGLMSFYISDGPLINVLRYDLKLNGNYKAVLRRVRKSEDGQIVVVGSTPSVAELLRQAQQVGIMNDKYSYIIGNLDLQTFDLEEYKYSEANITGFRMFSPTQSIVQDLISHLDYSGSINDRIINDSCPITLEMALTYDAVQVFAATTKKIMYRPQALNCSDQSSQVQTDGSTFKNYMRSTNIEEKTITGPVYFDGNIRKGYTLDIVELQTTGLVKIGTWDERNNLTIQRPPQSKLWSEVDANSLVNQTFRVLISVPNKPYASLVESHKKLVGNNQYEGYSIDLIKELSAKLGFNYILINGGSNYGNFNKTTNKTTGMMKEINEGRAELAITDLTITSEREEIIDFSIPFMNLGIAILYTKPQKSPANYFSFLDPFSREVWIYLGLVYIGISLCFFILGRLSPTEWDNPYPCIEEPEELENQFTLNNSFWFTTGAFLQQGSEIAPKSLSTRTLASIWWFFTLIILSSYTANLAAFLTIEKPAAVITKVDDLANNVDGVKYGAKRTGSTRTFFSASEHEVYKKMNEFMIKNPDLLFETNFQGVDRVKNGKNYAFLMESTSIEYNIVRECNLMKVGEPLDEKGYGIAMVKNWPYRDKLNNALLELQEQGVLAQLKNKWWNVVGAGVCKKKSDSQVASLDLKSLGGVYLVLGVGSGLSLIYGLIMWCIYVARKANYYEVPFGAAFLEELRIAINVANNERILKSAQSVYSRSRNSLLSIDSIDTDSEVEKSSKTDEESENTY

>BcucIR13

MFIKQNIYLVLIFLSKQFWFSTAQYEKWSNKEYENDKYSFSKPIQIGLITDQYSEEMGIIFEHAIEVANGELKIPLEATKEEVNYGDSFQAYGILCKLLQNGVGGIFGPSSKHTARHLTTICDAKDVPYFFSEMNENPEAFNLYPHPMDFSKALYFLLTTYKWSRFIFLYESVEYLNILNGLQSLYGNNSTTITVLRYDIEMNNNYKSVLRRVKKSGDNCIVVTGSSDTMPEFLKQAQQVGIINKDYKYIIANLDFHAFDLEEYKYTEANITSFRLFSPEQKAVQEILEKMGQKSSSENLRNGSCPITVSMALTYDSVQLFAETTKHLRVRNVPLNCSDRSESVLDDGSTFKNYMRTLKLAKRTLTGPIYFDGNVRKGYALDIIELHPSGIMKIGTWNDYSNLTIQRVAQTNSVIDNVDNSLVNQTFTILLNVPNEPYASLVESFEKLEGNSQYHGYGVDLIKELAQKLGFNYVLKNGGNDYGSYNATTNTTTGMLKEIIDKRAHLAITDLTITAARQEVVDFSIPFMNLGIAIIHVEPQKETQAYFKFMDPFSNGVWWLLGLSFLLVSFSFFILGRLSPSEWDNPHPCIEEPTELVNQFTIGNSLWFTTGALLQQGSEMEPKAISTRTVASIWWFFTLLMVSSYTANLAAFLTIEKPTILINSIDDLVEHKHGVVYGAKKMGSTREFFEKSEDSRYKKMNKFMNEHPEYLTETNMDGVKRVSMTYAFLMESTSIEYHTMRMCNLSKIGNTLDEKGYGIAMRKNWEFRDKFNNALLELQEQGTLEKMKNKWWNEMGAGICTSGKQRSEETELQMKNLIGIYIVLGVGSGLAFVYGIVSWFFYIRTKAKHYHLSMKEVFLEEFKFALDFNSHTRILKNTASIYSRGRNSLPTDSSKEEQKSLQH

>BcucIR14

MMNNEQFGHSTASAQYFLQLAGYLGIPVISWNADNSGLERRASQSTLQLQLAPSIEHQSAAMLSILERYKWHQFSVVTSQIAGHDDFVQAVRERVAEMQDHFKFTILNSIVVTRTSDLMELVNSEARVMLLYATQSEAVTILRAAEEMKLTGENYVWVVSQSVIEKKDAHPQFPIGMLGVHFDTSSAALMNEISNAIKIYAFGVEAYLTDPANRGRRLTTQSLSCEDEGRGRWDNGETFFRYLRNVSIEGDLNKPNIEFTADGDLKSAELKIMNLRPGANNKNLVWEEIGVWKSWETQKLDIRDIAWPGNSHAPPQGVPEKFHLKITFLEEAPYINLSPADPISGKCLMDRGVLCRVAADHEMAADIDVGQAHRNESFYQCCSGFCIDLLEKFAEELGFTYELVRVEDGKWGTLENGKWNGLIADLVNRKTDMVLTSLMINTEREAVVDFSEPFMETGIAIVVAKRTGIISPTAFLEPFDTASWMLVGIVAIHAATFMIFLFEWLSPSGYNMKLYLQNASVTPYRFSLCRTYWLVWAVLFQAAVHVDSPRGFTSRFMTNVWALFAVVFLAIYTANLAAFMITREEFHEFSGLNDSRLVHPYSHKPSFKFGTIPYSHTDSTIHKYFKDMHHYMRQYNKTSVAEGVAAVLNGNLDSFIYDGTVLDYLVAQDEDCRLMTVGSWYAMTGYGLAFSRNSKYVQMFNKRLLEFRANGDLERLRRYWMTGTCRPGKQEHKSSDPLALEQFLSAFLLLMAGILFAALLLLLEHIYFKYVRKRLAKKDGCHCCALLSLSMGKALTFRGAVFEATEILKKHRCNDPICDTHLWKVKHELDMSRLRVRQLEKALDQHGIKPPQLRLASSSDLLNHHHLKERPPLLGNLSLAASAQDLYRWSYKTEIAEMETVL

>BcucIR15

MCAMRFHAFLWSLWLPLCLLALQPQNAEANDFSSFLTANASLAVVVDQEYMQRRGENILANFQKILSDVIRENLKNGGIEVKYYSWSQIRLKKDFLAAMTVMDCKSTWQMFDSTQQNSILLFAITDANCPRLPLNRAIMIPIVDEGQELSQIILDIKVQRLLRWKTAAVLLDQTILHDNPTLVESVVLESAKNHITPFSLLLYQIDDTLRSQKKRTAIRQMLNIFQDSTQTPRQFIVLSEFYEDIVEIAASMKLFHVYNQWVFFVLNEEQRSHDPMSVTQNLEEGANIAFMLNTTEPTCTNSINCTITELSMAFVKSISRMIVEEQSIYGEISDEEWEAIRFTKQEKQDEILGYMKEYLREYSKCTSCAHWKIETALTWGKSEEHRRYQSNLELRDTRNRNFEFIDVGYWTPTLGFNTHEVMFPHIVHFFRNITLDILTVHSPPWQILERNSRGDIVRHNGISMEILKEMSRLLNFSYILHEVKVNAADLAAEDMQHTNNVTDDLSGSLTFNIPYQVIETMQSSRYFMAALAATIDEPDKKSFNFTVPISVQMYTFISRQPDEVSRIYLFAAPFTTEIWGCLVAIIIITAPVLYFINRWVPMDHLRITGLSTLNSCFWYIYGALLQQGGMYLPKADSGRLVIGVWWIVVIVLVTTYSGNLVAFLTFPQFQPGIDYFFQIFSSNAVQQFGLRNGSYFEKYATQITTRDDFRDYIQRANIYNNVQGEDIGAVQDGKRINVDWRINLQLIIQKQFEKDKECKFSLGRDNFVAEQIGLIVPRDSPYLQLINDQITRMFQMGFIERWHQINLPSMDKCSGHGGMRQIMNHKVNLDDMQGCFMVLLFGFIIALFILFVEYWYRWYFVERKKGVFAT

>BcucIR16

MQSPLLCPSWCWHYLRLSCWLAVLSTLLRHTLALPDVIKIGGLFHPTDDTQELAFRQAVEHINSDRLILPRSKLVAQIERISPFDSFQAGKRVCGLLNIGVAAIFGPQSSNTASHVQSICDNMEIPHLENRWDYRLRRESCLVNLYPHPNTLAKAYVDIVKHWEWKTFTIIYENNDGIVRLQELIKAHDNSPYPITVRQLSLSGDYRPLLKQIKNSAEAHIVLDCSSDKIYEVLKQAQQIGMMSDYHSYLITSLDLHTINLDEFRYGGTNITGFRLINEKVVSDVVRQWSYDDKGLQRSANLSTVKAETALMYDAVHLFAKALHDLDTSQQIDIHPISCDGQNTWQHGFSLINYMKIVEMKGLTNVIKFDHQGFRTDFVLDIIELGPLGIRKIGTWNSTLPEGINFTRTYSQKQREIEANLKNKTLTITTILSNPYCMRKESAVPLTGNDQFEGYVVDLIHEISKALGFNYKIQLVPDGNYGSFNKQNGEWNGMIRELLEQRADLAVADLTITFEREQAVDFTMPFMNLGVSVLYRKPVKQPPNLFSFLSPLSLDVWIYMATAYLGVSVLLFILARFTPYEWPAYSDAHGEKIESQFTLMNCMWFAIGSLMQQGCDFLPKALSTRMVAGIWWFFTLIMISSYTANLAAFLTVERMDSPIESAEDLAKQTRIKYGALKGGSTAAFFRESKISTYQRMWSFMESARPSVFTSTNAEGVDRVAKGKGSYAFLMESTSIEYVTERNCELTQVGGMLDTKGYGIAAPPNSPYRTAINGVILKLQEEGKLHILKTKWWKEKRGGGSCRVETSKSSSAANELGLANVGGVFVVLMGGMGVACVIAVCEFVWKSRKVAVEERLSAILHE

>BcucIR17

MKMKVFILYLLGWLPDGLVHGAITYNADRNISDVAIALSEIINGLKPRQLAILAAPQFHFTTRHAPLPTSAEIPDDTQLEGMQMDIDDFIYQLHKLNFKSVIYDKTDAFFKFVEDSLQGSIESVNLIFSAPYELSARIQERKLSHRLSLFIFYWGAKHPPKAQEVRFEEPMRAVVITRPRKKAFRIYYNQAVPDGVSNLRLVNWYDGDNLGLQKVPLLPNAATVYSNFNGRVFRVPVFHSPPWFWVNYENDSTNSTLLDDYIDATNDYTELTEVNVTGGRDHCLLNLLAHHMNFQFIYIEAPGRTQGSLRNDDTGEENETFTGGIGLLQNGLADFLLGDVSLSWERRKAVEFSFFTLADSGAFATHAPRRLNEAFAIIRPFKRDVWPYLILTVIFSGPIFYAIIAIPYKWHLPCQRRGEVRRLTQRQQQQRDVERADELFFHMAYVKEITGDNEMTRRLLRQQQQQQQQHQSEVCEQLTVGGRVQGLTEIPNNLFDKCIWFTVQLFLKQSACKELYHGYRAKFLMIVYWIAATYVLADVYSAQLTSQFARPPHEAPINTLQRLQTAMLRDGYQLFVEKESSSLEMLENGTEVFRQLYALMKLQNPDMEGYLIDSVEAGILLIADGLENKAVLGGRETLYFNIQQFGSKTFQLSHKLYTRYSAVAVQIGCPFLDSLNDVIIHLFEGGILDKMTNAEYATQSRMLGKEYNAMHPTNPTETNGNNEPPPNDDNRNTNGVGDANGKGEENAEATAKSLDSQIIQPLNLRMLQGAFIVLICGYAAATGILVLELCCHRLNWHFFE

RTQARLLRRYRWCSRKFRRITHLLFARIIR

>BcucIR18

MSFDLKRTHHIYLLILVVVKASSTLPPVIRIGALSSDMPDGPIDLAFKYAIQSINSDVNLLPDTKLTFDLQHVGKDDSFHACQKVCGQIETGIHAFFGPFNASLGAHIHSICDALDIPHMESRIDQKEANTEFSINLHPAKQYVNLAFEDIIRYLNWTKAGILYEKGFGVLQLRKFGQMYDIETYLRLVTPTTYVKILNELKDKEIHNLIIDTNAAHISILLKGILQLQMNEYKYHYLFTSFDIETFDLEDFKYNFVNITSFRLVDVGDVSVRNILKGMEAYNYDYNYKTTYYRKLRTIKTEPAVVYDSIYVFAIGLTSLEQSLTLGVSNASCANESPWDGGLSLINYINSVEWRGLTGPIQFKEGRRVKFKLDLVKLRQHSLVKVGEWTPQTRLNITEPSLFFDGGTMNVTLVVITILETPYVMMHYGKNYTGNERFFGFCVDILELIARDVGFDYILDLVPDRKYGAQDPFSGEWNGMVAQLMKYKADLAVGSMTITYARESVIDFTKPFMNLGISILFKVPSSPASRLFSFMNPLAYDVWIYVLAAYFLVSFTIYIVAKLSPIEWRDKYACDKKNPIITNQFTLANSFWFTIGTLMQQGSDINPKSLSTRIVSAIWWFFTLIIIASYTANLAAFLTVERMITPIENAEDLASQTEISYGTLESGSTMTFFRDSIIETYKKMWRNMENKKSIALTSTYEEGIRRVNQGNYAFLMESTMLDYIVQRDCNLTQIGGLLDTKGYGIATPKGSPWRDKISLSILELQEKGDIQMLYDRWWKNAGDTCVRKSNNKQTKANALGLDNIGGVFVVLFVGIGVAACVAVFEFWYNYRSRKRESVYYEKEHNAIESDMTAMALSAFNFADSTDKENIGEGHTEDQYFIQTHKQPCKHVTAYCCTEPPAQRSLCGEMLDEFRYALRCRDSHRRPALKRRCPTCHLFKDVKEFMDIETRTPTYVRGSTTNDLQIHHTQVKSTIEPQTSPSPIALYPCNAYKSNKIMD

>BcucIR19

MKTFNFNPHFILLICCIFRVATSSGAAADEFATAEQTGHSIAEEPTTQSSDAQIFMEYFRWHGVHNILLIVCPQDAATSEEHHKLKTLLRQFIANGFSTRIFNGADYDEGGGAPSAMKAQTEPEPVNMTVSPTVDSTRGAPELNRATYGPPRTFRSDNSTRRPLRLQLPALTYKSGILLWRFGSSCALNVLRWAAAPEHNYFTTNRFWLLHTDESTHISLLEDEDIFLPPDGEVRAVLQQPDAQLFALVDVYKIAADRPLRRTSVGGRDLRNAGDMLQALSKFGSAISYRQNLEGLTFKTGLVIAFPDMFTNIEDLSLRHIDTISKVNNRLTLELANKLNLRFNTHQVDNYGWHQPNGSFDGLMGRFQRYELDFGQMAIFMRLDRIAIVDFVAETYRIRAGIMFRQPPLSAVANIFAMPFASDVWIAILLLMIFTIGIFMVELVYSPHTHEIDILDCVVFVWGAMCQQGFYANLLNRSARVIIFTTFVSTLFLYTSFSANIVALLQSPSEAIQTLSDLTQSPLEIGVQDTVYNKIYFNESTDPVTNHLYHKKIAPKGENIFMRPPIGMEKMRTGLFAYQVELQAGYQIISNTFSEPEKCGLKELEPFQLPMIAVPTRKNFPYKELFRRQLRWQREVGLMNREELKWFPQKPKCEGGMGGFVSIGITECRYALGIFGFGLLLSAFSFILELAVNYAWNLVKKIHRNKKQREASGATNGYTGDFLY

>BcucIR20

MWPTQTHVTTLLQLLIQRYFAQFSSVLIVHDGRVDADSALQREYLEAVQLAFRNLSQQGRVIGLQWIDVSQLDGQGSSRDGDNSGFCGSSAGKDSRNNSVDNLAYDDELELCVLRAVDIVTEGFITILSDTVRFLHARYFATRNAELRLKDKFYLFLCEHEHPEELLSTEILQFYQHHLMVTPEKIGAPQRDNPQAATATRRKRNATKPTTVTTTNPLSTLTTATASAHRDINIQLWTQKFVGARGNLEALLLDAFLPNETFARNVELYPNKVSDLRGRNIRVGSITYIPYVVANYVPAGTGDVDALNSSDYSRTLSYLGSEAELMKSFCEVRNCHLRVEPYGADNWGVIYENESATGMLGDVYTQNVEVAVGCIYNWYNNITETSNVIARSSVAILGPSPAQFPAWRAIIMPFSTELWIFLILTILLCAAVMYFIRFVASTLDKWQRSLQQDFQHTAAIGQAILDMFAVFIQQPSGPTSLHTFAARFFLAMILCATITLENTYSGQLKSILTVPLFTEAVDTMEKWSKTDWTWSAPSIVWTQTIDSSHIEKEQIMSEKFVVHDYDFLYNASFRPDYGLGIERLMSGSFTFGDFITAPALETKIISKDDLYFDWTRAVSIRGWPLMPLFDEHISACIETGLFVHWERQNVAKYLDRQTQQIMLNLASGHINKSPPQKLTIENISGATFALIFGCLIASFVFLLELSIYNFNKFQDFRNKTK

>BcucIR21

MTVSRRALTARRQIFATTLTCGHTRFITILILLLSKWNLCVLTAANAYEFRAFADVLKQQHLQHAIIAYNSDTEQTQQQAGLLKDNALRALLNVASLHFYDVHQAKSTKNYTDFERLFYHDSPRVGIYVAQLEDVLLQQFVLGSNVISVDTIDAGGYRVRVDVGARFNNSRVWFIMSKQRAVATALANVRRVLTPLAVNISADITVGVRLVDNNTIELFDIYKIQKDWLEIEPKGYWSSTVGLKLNLRFHQTFVSRRRNFKGLTLVGGIVIREQPVGMDDLEYLNSLDYKNFDPMQRKTYQLMKLMEPVFDVSFQPILKNIWGEQSTNGSWDGVMKLLLSGEAEFSLCPMRFVMNRVHLIHYTIAVHTEFVFFIFRHPRRNDIRNIFFQPFVEEVWYTVIAIIVITTLLLQLHIHHENRFFINKDPHFQTRFDYAILSILEAFFMQGPANDAFNATSTRTLIFSVCLFSLLLQQFYGAFIVGSLLAASPRTITNLEALYNSSLEIGIENIPYNIDTFEKTTVPLGIAIYKERVCKNRERNIISIEEGAERIKKGGYAFHVSANRIYYILKELLTEKEFCDLQDIPFIPPYRIGIGITKTSPFREYFTTSIANFHTSGLLQYHDNQWQLPEMDCSLSQNHEVEVDLQHFLPALMFLISAMLLSLAVLILEIIYYNLEHSARLARLCPRIMPKPKLEFIN

>BcucIR22

MLPTNLLYSFYWSRMFNVIMQNYLLSTTTCIIWPENEDFSISWQQDKPPDAAIISIRLHDLAQSFSKDVVDFAAKREELLNDYVVLNPFVEKLTLSIEKSHCQNFIAFQMDIPIFIDAVINASRFSIWRSSNNKFLFVYNKDDLQQQLFENRFFEDQSAILLIERSITNPAVFDLKTNKFVGPRADNPKQLYLLDTFNAETNTFLHGNDLFPGKLSDLQGREVILAAFDYRPDVVLKYYPGAPSRDRAFAANDTNGDVELDGTEERILKTFCEKHNCLVDIDTSEADDWGIAYRNMTGEAALGMIARGKAEVGMSAMYTWYADYVALDMSMYIGRSGITCVVPAPKRLASWLLPIEPFQPALWAFVFVCLCVEIIALLFIDHARPIIIALSERMHASEEQQNSWVRNFQYAFTTTMLLFVSQSNKGTMVNFTPLRVMLFASFLNDIVITSIYGGGLSSILTVPSFGQAADSVERLYAFQLKWGADSEAWVAAIRDDESEIMKGLLRNFDIYSAEQLMELAQTEEMGFTIERLPFGHFAVQEHLTRSVLGKMKIMVEDIYFQYTVAFTARMWPMLEGFNEMVVMWHSSGLDKFWEWRIVADNLDGAIQKELMASQYSNLDDIGPVKLGMSNFVGMLLLWLLGITCAFLAFLAELLMDHMKRAKKVAEFEVIEIGEGSV

>BcucIR23

MTGFELILSAALCLSCTNLTNTRLPEGLIELDENGSVVTISPDLAVDEPSLDDAPLETVKTIIAKKEKTDKLKQWIKGRKLIIATLEDYPLSYTVMENDTRVGKGVAFELIDFLQEQLEFTYEVVVPEDNIIGSREDYEKSLIKMLNNSEADLAAAFIPTLSEQHSFVFYSTTTLDEGEWIMVMQRPRESATGSGLMAPFDFWVWILIFISLLAVGPIIYILIILRNRLTGDKEQKPYSLGHCAWFVYGALMKQGSTLSPIADSTRLLFATWWIFITILTSFYTANLTAFLTLSKFTLPYNTVSDILYKNKHFVSARGGGVEYAIRNTNESLSMLTNMIRNSHAVFSKSSNDTFNLQNFVEKDGYVFVRDRPAINHVLYADYRYRKTISMNDEKLHCPFAMAKEPFLKKNRSFAYPMGSNLSELFDPKLLNLVESGIIKYLSTKDLPNAEICPQNLAGTERQLRNTDLMMTYYIMFAGFATAMVVFFTELIFRYLNNRHEGNKWARHGIGRTTNGLSVRAPRWLRQLETDSDKQRLTASPTESTITPPPPYQSIFSSNHRHHHEHEATHLSKDSSLHRWRRAGQFGIGAGGSNFGALAAGVGGGAGVLLGNGQLQGASGAGGMRRLINGRDYMVFRNPNGQSQLVPVRAPSAALFQYTYTE

>BcucIR24

MSTLSPAVNHWTTLLNFIIQTYFIDSHATCILWHRDFPFELQTPANSEFIAYINIWPDNLSQQSLQQDIFNFTAFAETQLAYGMQPDALVQKLTIAIRETHCETFVAFQEDIPSFARSFYNASRISVWRSLRNKFLFVYRKDLQPDTAAYFDDSLFKDQPNVLIVEAECGNCSTFALKTNKFIGPLAEHPEQLYVLDRYNAVDGKFEYGVNLYMDKVKNLQGREVTVGIFDYRPFTAVDYDRQPQVKDHSPENIRGTVHIDGTEVRMLFALCEVINCTVDADTSEDDWGTSYANLTADGIFGLITSRKSQYIVGALYFWPDDYRYLDMSLFIGRSGVTCLVPSPHRLTSWLLPLRPFQLTLWLGVFASLGLEAIALFFTRHLAPSPTEPQYGLMESFQFGYITTLKLFVSQGSDYVVNSNTVRMVLFACYMMDTIVTSVYGGGLSAILTLPTMEEASDSVERLYRHGIPWTATSPDWVISLRGSGADRDVVVESLLENYHVYTYEQLTQFAKTENMGFILERLAFGHFGNVDFLTDESFKRLKLMIDDIYFQYCFAFVPRLWAMLPKLNEVIMSVHSTGLDIFWEWEVAATYMDGQQQEEIQASMYMDFDVGPVKLDMGNFIGLVLPLIIGIVFSVFAFIGELIYYRYSQKKAQTVVNVN

>BcucIR25

MDLKLFNLILYYFLKLNMKVLVSFNCWDVETQLAFYKLAAENSFYIDYINLNDSKALQGIEYRLIAKKPTMGIFMDLNCESAEELLSIASHERLFSDHFFWLIYDDMANVTYFRGLFKRQNLAVDAEITYAYLNTPEDDGNSSAIASYTLYDVYNNGYYHGGKLNMTLDREIYCNSEECYVNKYLSKLHLRNKYGNRNTLHDATLRLTVVVTKIPITSTPEQIFAFLRSVNGTNYDAIARFGFQALSILVEFLGCKTNHTFVNRWTINETHGGLIGALAIQSADLISTPFIPTAPRMEFFTIIAETSSFRSICLFRTPRNSGIQGDVFLKPFNTTVWSLFGVLLLLTAVVLWSIFRLERYRMYKRYIDYMPSLLATFLISFGSACSQGSDMVPGSIGGRMVFYTLYLLTFLMYNYYTSIVVSSLLGSPVKSDIKTMGQLADSSLEVGLEPLPFTLTYLNNSLLPEVRRFKHKIDSVPNPQAIWMPLEKGILRVRDQPGFVFGFEASTGFLLVKRYYKPYEICDLNEVLFRPEKSLYSAVHKNYSYKEITKQKVIRILETGVNLKLHRYWVQTTLECFDSNFIVEVGMEYMAPLFMLLACTYVLVLMILLCEILHKKYWTERKMRLENILFGQNWHNE

>BcucIR26

MELMLLNFILHHFLSANINSIVVLNCWSAQTQCDFSKMLNGHSLYSRFVNIESIDMSADFEYRYLLHNRPGMGAYLDMNCKSSEQVMGTLNRSHLFNAHYNWLLYDRIADLNNFRRLFADANLDVDAELTYAILKPTRLGPVSEINITSFVKYDVYNNGYNLGGKLNMTVDREVECNVTSCYIKRYLSQLHTRSKYGNRDKLHDITMRVSVVITQLPLSTPVPMMLDFLTSENNSDVDPISRFGYRIMLIFKDLFGCKMRYTFRSHWGINETYGGSIGDLISSEADFLSTPFLSTAARVKYVSPLLETGGFSSICIFRTPRSSSMKGEAFVQPFDGSVWLVFVILLIVVAIFLWYTFILEMRNCRTYLPYTPSLLSTGLLAFGSACYQGSHIVPSSVGGRLAYLSLYLATFIVYNYYTSILLSTLLGTPPKSDIKTLGQLADSSLTVSLEPLPYNYVYLNASQLPDVRRFVSRKIESRKNPQKVWIPVKEGVLRVRDEPGFVYVLETSYAYPFLERNFLPHQICDLNEVNLRPDKSLFTQLHKNSSYRELTRIRGIRMLETAVFRKHRRYWVRNKLNCVPSNYLFAVGMEYTAPLFLMLAFSYILCLLLLGLELLIKRLSA

>BcucIR27

MNSHQGQFNTEVFSELLRGIDYLNLHLFNERYFENSIVQQFLQQTYCPLQLSVGDMQDQVYSSQRHWALQLLNLARRQWIERGALQIFYLIPNSANNSGIECFFLNPFPNKGAQRGVMTRLGDESYKSIFRNFHEYPLRTYIFHSVYSDLQIFMNETSKQIIGTTGADGKVADLLASKMNFTMDLQWPDDAFFGTRTKNGSYDGAIGRMIRFETDIILSGFFIKDYLTRDIAFSSPVYMDELCCYVKKASRIPQSIVPLFAVKVDIWISFLFVGMLTPFMWMLLRRVNLGAMAKGSTRLKLQKLQAQETRLLTQNHKLQYLRIFIDTWVMWVRVNIVSYPPFISERIFIASLCLVSVIFGALFESSLATVYIRPLHYKDINTMKELDEANIMIYIKHGAMRDDLFYGHSSQIYQNLEKKLLLIAELEERLIHTMSRGGKFASVTRASSLELDDIHYFITKKIHKIPECPKSYHIAFVLPSNSPLEKSFNILLLKFVQAGLIDHWIADMMYNAKIKTRNFVGFLDETGDKWKVLTLNDLQLSFYTVICGSVLATIVLLLEFIVHCKKIRNLLGKIA

>BcucIR28

MIIMAYLQEPTKCHVIYITMPNLIIKTNMLLINFSFAGYEFSRTRYKCALSHILLIYYLSNFFCAAALPAVIPLGAIFTE

DQRDSSIEYAFKYAVYRVNKDKLLLSNTQLIYDIEYAPRDDSFRTTKKICRQLESGVQVIFGPSDPLLSAHVQSICETFGIPHIESRIELDASTKEFSINLYPSQRLMNLAHRDLMIFLNWTKTAIIYEDDLRIFNHQDLVHVTADIRTELYIRQTSPNTYRQVLRAIRKKDIYKIIVDTNPTNINAFFRAILQLQMNDHRYHYMFTTFDLEIFDLEDFKYNGVNITAFRLVDVESQRYKEVIEQMQKLPHSGLDYINDQPYIQTQSALMFDSVYSVAAGLMELDRSHLLTWHNISCKSDMPWRDGLSLYNYINSASMNGLTGRVHFAKGRRNIFQIDLLKLKREKIQKVGFWKPDVGVNITDATAFYDTHTSNTTLIVMTRQEKPYVMVKSDITQSGNDRFEGFCIDLLKAIATQVGFQYKIELVPDNMYGVFNPDTKVWNGIVRELMEKRADLAVASMTINYVRESVIDFTKPFMNLGIGILFKVPTSQPTRLFSFMNPLAMEIWLYVLAAYVLVSLTLFVMARFSPYEWNNPHPYVKESDIVENQFSVSNSFWFITGTFLRQGSGLNPKAVSTRIVGGIWWFFTLIIISSYTANLAAFLTVERMITPIEGASDLAEQTDISYGTLEGGSTMTFFRDSKIDTYQKMWQYMEARRSSVFVKTYEEGIKRVIEGNYAFLMESTMLDYAVQRDCNLTQIGGLLDSKGYGIATPKGSTWRDPISLAILELQEKGIIQILYDKWWKNTGDVCNRDEKSKESKANALGVENIGGVFVVLLCGLALAVVVAILEFCWNSKKTLQLTETQILCSEMAEELRYATHCHESKQRQSLKGSSAKFPPDSTYVPADTGSVIPNRGGVHYNYFD

>BcucIR29

MLNCGLIMEIIDINEKYKLDNVLTRESYGKSSVIADCICVNTSSLLSLASAGRYFNKTYQWFLWYKERKEVELLTLHDINYLGPNAQVTFINGSNSTIQIWDVYSIGKHLRNPLKLSLINFTPFTSYKAISKKLMKFQYSRSRNKFHGAVLRGATVIDLDNVINKTEIYSLLSDPVKHSGISAFTKYYYELLQILKEHINFRIEFRVSRGWAGRLDNTSYRLGFLGIMARNEADVGASGIFNRINRFADFDIIHQGWKFETAFVYCNTPDLSTKIKGTNFLIPFERDVWLAMIWLFAVMCLIYWLLQNVNVKLCFLKKEDLQEQRLMRFIKSGLLQGRQNRLLDEYSVDYLNSYIKETSSISNLFLIFIAAICQQSTNLISRSLAIRVLYFVIFLNTLLLYNYYTSSVVSGLLSSSVQGPANVDEIIASTLQLSFEDIGYYKILFKIIDF

>BcucIR30

MIIMAYLQEPTKCHVIYITMPNLIIKTNMLLINFSFAGYEFSRTRYKCALSHILLIYYLSNFFCAAALPAVIPLGAIFTE

DQRDSSIEYAFKYAVYRVNKDKLLLSNTQLIYDIEYAPRDDSFRTTKKICRQLESGVQVIFGPSDPLLSAHVQSICETFGIPHIESRIELDASTKEFSINLYPSQRLMNLAHRDLMIFLNWTKTAIIYEDDLRIFNHQDLVHVTADIRTELYIRQTSPNTYRQVLRAIRKKDIYKIIVDTNPTNINAFFRAILQLQMNDHRYHYMFTTFDLEIFDLEDFKYNGVNITAFRLVDVESQRYKEVIEQMQKLPHSGLDYINDQPYIQTQSALMFDSVYSVAAGLMELDRSHLLTWHNISCKSDMPWRDGLSLYNYINSASMNGLTGRVHFAKGRRNIFQIDLLKLKREKIQKVGFWKPDVGVNITDATAFYDTHTSNTTLIVMTRQEKPYVMVKSDITQSGNDRFEGFCIDLLKAIATQVGFQYKIELVPDNMYGVFNPDTKVWNGIVRELMEKRADLAVASMTINYVRESVIDFTKPFMNLGIGILFKVPTSQPTRLFSFMNPLAMEIWLYVLAAYVLVSLTLFVMARFSPYEWNNPHPYVKESDIVENQFSVSNSFWFITGTFLRQGSGLNPKAVSTRIVGGIWWFFTLIIISSYTANLAAFLTVERMITPIEGASDLAEQTDISYGTLEGGSTMTFFRDSKIDTYQKMWQYMEARRSSVFVKTYEEGIKRVIEGNYAFLMESTMLDYAVQRDCNLTQIGGLLDSKGYGIATPKGSTWRDPISLAILELQEKGIIQILYDKWWKNTGDVCNRDEKSKESKANALGVENIGGVFVVLLCGLALAVVVAILEFCWNSKKTLQLTETQILCSEMAEELRYATHCHESKQRQSLKGSSAKFPPDSTYVPADTGSVIPNRGGVHYNYFD

>CcapIRkainate2

MLKYFFSLLLACFCVKNAQALQPLNIGAIFFENELDLERTFIATVESINTARETNFKLLPLIRRVSETDGSLILQREVCDLIDNSVVAIFGPSSKADSDIVSVICNATGIPHIEFDISQEETGEEKINHQMTLSVFPAQLILSKAYSDIVHSNGWRKFTIVYDADDPKAPTRLQDLLQLRDIHNDVVRVRQFRRGDEYRVMWKSIKGERRVVLDCEPDLLIDLLNSSIPFDLTGQFNNLLLTNLEAHNANLEVLRDNETFALNITAARLKMNGNFYNTNPWQQASLYNLDELPEQPMRTLLHDLLYDAVNLFTNALRNVSYSYQIRPPRVRCDFSEYGRRQPWAMGRYIHRVMLATSGVNNTDYRTSDLQFGEDGQRINFGIEIFEPLDNYGIAFWDTKGQIVPQHVVVSNLKKLRYRVATRIGEPYFMEIPEMVEQNVTGNERYEGYAVDLIKELAERMNFEYIFVPVADQQYGKYDPTTKQWNGIIGEIINNDAHMGICDLTITQARRTAVDFTVPFMQLGVSILAYEQTVEPKALAFLDPFRAEVWIYVLIAIFVISFLFVVSARIAEDEWENPHPCNKDPELLENKWGLFNTFYLTAASIMQAGCDMLPKSAPFRTFTAMWWIIAVIIPNSYTANLAAFLTSSKMVNSITDLKSLVDQNEVKFGTLKGGSTYNFFSESNETVYRLAFEMMKNAEVSAYTKDNKEGVERVLSKAGKYMFLMETTSLEYNIERHCELRMVGEKFGEKHYAIAVPFGAEYRYNLSVNILKLSETGKLFEMKNKWWKNKDGACEKNDDGDSSAMGFAEVRGIFYTLFLGLFAAYFLGICEFLMHVHSRASEEKLRFKEVFVNEMRFVLRIWNNRKPVSCTPTASLSASSRRSSNRTIKSSKKKNSRQSSSSSKELAELASIKVKKNGRVVKEDDV

>CcapIRkainate1

MKLLLFTLFSLAVFQHTAQFTNNFDVVKIGAIFFHDEMKLVEAFDTAVRDINNLELDVRLEPIKHYLSSDDSLTLQELACDLIENGVAALIGPSSKASSDIVEVICNTTGIPHLQFDWYGEDIYGHRRNHKLTVNVAPVEQLLAKALLDIVRFKKLESFMIVYENSRNLGRMEYLFSWRKMNNKTGIRLWHFSRGDDYRVMWKQINSLREKYIVLDCPADILIEVLNASIIFNMTGQFNQWFLTSLDTHTANFSSSYFTAFLAAIYAVRLRPYIPPPVHDESDVFEEDEPDLISSARGKLLYDAVVLYFNAVRNQIRSQQYREPRARCHRGFWRTGLEIIEQMKILTARNVTPPFKTQKMQLNEYGTRDEFNLEIYNPLIDRITHIWNKNQNLVPYETLVAAKESKKKKFDEVQDYTVKRVKYIVATRLGEPYFMYRPEPEGVHYEGNDRFIGYAVDLIYKLAEELDFDFVFELVPDNNYGSQDPKTGEWNGIIRQLIDNSAQIGICDLTITQARRTVVDFTVPFMQLGVSILFYREPPPPKSPYGFLAPYSLDVWICLLLAIMVSALALVVVGRISHADWVSPIAHDPDPAEVENIWNLNNTLWLNVGSILCQGCDLLPRGSAMRVFTAFWWIFSLLLSQTYLANLAAFITASKMQSSVDSLHDLINQNKIQFGMLKGGSTQLLFSESNESEYRLAWSKMVSMKPDAFTASNAEGVDRVKRSEGRYAFLLETTTLQYYLARNSELKQIGTPFNEKHYGIAVPLHAPYRSTLSVGILKLSEKGELFKLKKKWFSANESSKNAKEAKNDESQYTMESVSGIFIVLLGGICVSLLIGILEFLWNVEQIVVQEKLPAMVVLKAELKFFIRFWETRKPLHTYAESRGSTSTGYSSYDQMASGSTVKKKKKKKKKKGKSNKVSSD

>CcapIRNMDA2C

MKGLIFSRKANKPRKNDETNRTPKRTVKTATATIAAACASTKTQTVKICKAAKTKTETAATTKTTPTHNTHNGNSKQLTTPLKCYKNNMQQQRSSCNGSVNNSYDNYSNNNNNSSSSKGDTSQCSRYVNYGSRHCGRKGDSSSLIPTYNQQQHHQEQQHQHCLAPCAELTHKMPATISVTTQKPNVSVCVHQQHQRMVPNPLEGVHHRQRLQQQHQLTTTTKPTATSLPPTNHHAQPQQRHKQKQQKQQKQEQPATTATTVIACLRKSWSTFLSSRLNFHQHLGHISLTVLVLASILIYSCPCTSALRLTNGGNTKTLSANKEQLNIGLIAPHTNFGKREYLRAINTAVQGLAKTRGAKLTFLKDYSFEPRNIHFDMMSLTPSPTAILSTLCKEFLQANVSAILYMMNNEQFGHSTASAQYFLQLAGYLGIPVISWNADNSGLERRASQSTLQLQLAPSIEHQSAAMLSILERYKWHQFSVVTSQIAGHDDFVQAVRERVAEMQDHFKFTILNSIVVTRTSDLMELVNSEARVMLLYATQSEAVTILRAAEEMKLTGENYVWVVSQSVIEKKDAHPQFPIGMLGVHFDTSSAALMNEISNAIKIYAFGVEAYLTDPANRGRRLTTQSLSCEDEGRGRWDNGEIFFRYLRNVSIEGDLNKPNIEFTADGDLKSAELKIMNLRPGANNKNLVWEEIGVWKSWETQKLDIRDIAWPGNSHAPPQGVPEKFHLKITFLEEAPYINLSPADPISGKCLMDRGVLCRVAADHEMAADIDVGQAHRNESFYQCCSGFCIDLLEKFAEELGFTYELVRVEDGKWGTLENGKWNGLIADLVNRKTDMVLTSLMINTEREAVVDFSEPFMETGIAIVVAKRTGIISPTAFLEPFDTASWMLVGIVAIHAATFMIFLFEWLSPSGYNMKLYLQNTSVTPYRFSLCRTYWLVWAVLFQAAVHVDSPRGFTSRFMTNVWALFAVVFLAIYTANLAAFMITREEFHEFSGLNDSRLVHPFSHRPSFKFGTIPYSHTDSTIHKYFKEMHHYMRQYNKTSVAEGVAAVLNGNLDSFIYDGTVLDYLVAQDEDCRLMTVGSWYAMTGYGLAFSRNSKYVQMFNRRLLEFRANGDLERLRRYWMTGTCRPGKQEHKSSDPLALEQFLSAFLLLMAGILFAALLLLLEHIYFKYVRKRLAKKDGCHCCALLSLSMGKALTFRGAVFEATEILKKHRCNDPICDTHLWKVKHELDMSRLRVRQLEKALDQHGIKPPQLRLASSSDLLNHHHLKERPPLLGNLSLAASAQDLYRWSYKTEIAEMETVL

>CcapIR25a

MQYAYTKSCRNIFIFFIFLKIILLGSFATAQTNQNINVFFINDADNEPASKAVTVVSTYLKKNPSYGISIQVDQVEANKTDAKTLLESICSKYAESIDRKQPPHVVFDATKSGIASETVKSFTQALGLPTISASYGQEGDLRQWRDIDENKQKYLLQVMPPADLIPEVVRSIVRKMNITNAAILYDDTFVMDHKYKSLLQNIQTRHVITGIAKEGKREREEQIEKLRNLDINNFFILGNLMSIRMVLESVKPSYFERNFAWHAITQSEGEVSSQRDNATIMFLKPMSYAQNRDRFGRLKTTFNLNEEPQIMSAFYFDLALRTFLAIKDMLQSGAWPKNMQYIGCDEFQGGNTPERNIDLRSAFSMIQEPTSYGLFELVTQPGKPFNGYSYMKFEMDINVLQIRGGNSVNTKSIGTWTAGLDSPLVVKDEEIMKNLTADTVYRIFTVVQAPFIIKDDKAPKGYKGYCIDLINEIADIVHFDYTIQEVEDGKFGNMDEKGEWNGIVKKLMDKQADIGLGSMHVMAEREIVIDFTVPYYDLVGITIMMQRPQVPSSLFKFLTVLETNVWLCILAAYFFTSFLMWIFDRWSPYSYQNNREKYKDDDEKREFNLKECLWFCMTSLTPQGGGEAPKNLSGRLVAATWWLFGFIIIASYTANLAAFLTVSRLDTPVESLDDLAKQYKILYAPLNGSSAMTYFQRMANIEQRFYEIWKDLSLNDSLTPLERSKLAVWDYPVSDKYTKMWQAMQEAQLPATLEEAVERVRNSTSATGFAFLGDATDIRYLVMTNCDLQIVGEEFSRKPYAIAVQQGSHLKDQFNNAILTLLNKRQLEKLKEKWWKNDETQAKCDKPEDQSDGISIHNIGGVFIVIFVGIGMACITLVFEYWWYKYRKNPRIVDVIEANSGVKDGKVDSVILGQAGKEYGKSGNTVLRPRFHQYPTTFKPRF

>CcapIRkainate4

MTGFELILSAALCLSCAQQNDTSLPAGLIELDANGEVVTISPELAASEPSLDDAPLETVKTLLAKKEKIDKLKEWIKGRKLVIATLEDYPLSYTVMENDTRVGKGVAFELIDFLQDQLQFTYEVVVPEDNIIGSREDYEKSLIKMLNNSEADLAAAFIPALSEQHSFVFYSTTTLDEGEWIMVMQRPRESATGSGLMAPFDFWVWILIFISLLAVGPIIYMLIILRNRLTGDDEQKPYSLGHCAWFVYGALMKQGSTLSPIADSTRLLFATWWIFITILTSFYTANLTAFLTLSKFTLPYNTVSDILYKNKHFVSARGGGVEYAIRNNFVEKDGYVFVRDRPAINHVLYADYRYRKTISMNDEKLHCPFAMAKEPFLKKNRSFAYPMGSNLSELFDPKLLNLVESGIIKYLSTKDLPNAEICPQNLAGTERQLRNTDLMMTYYIMFAGFATAMVVFFTELIFRYLNSRHEANKWARHGIGRTTNGLSVRAPRWLRQLETDSDKQRLTSSSNESTITPPPPYQSIFSSTNRHQENHHKDATHLSKDRSLNRWRRAGQFGNMIGGASGSQFGALAGGGGAGVLLGNGHFSGGNGGAGGVRRLINGRDYMVFRNPNGQSQLVPVRAPSAALFQYTYTE

>CcapIR40a

MKVLLLNLILWLPAALVLGGITYNAERNISDVAIALSEIINALKPRQLAILSAPQFHFNTRHAPLPAAAEIPNDSQLESMQMDIDDFIYKLHKLNFKSVIYNKADLFFKFVEDSLLGSIESVNLIFSAPYELSARIQERKLSHRLSLFIFYWGAKHPPKANEVRFEEPMRAVVITRPRKKAFRIYYNQAVPDGVSNLRLVNWYDGDNLGLQKVPLLPNAASVYSNFNGRVFRVPVFHSPPWFWVNYDNDSINSTMLDDYIDSSNSYMELTEVNVTGGRDHCLLNLLAQHMNFQFVYIEAPGRTQGSLRIDDIGGENETFTGGIGLLQNGLADFLLGDVSLSWERRKAVEFSFFTLADSGAFATHAPRRLNEAFAIIRPFKRDVWPYLILTVIFSGPIFYAIIAIPYKWHLPCQGRAGKRANELVFHVAYIREITGDNEMAKRLLRRQRQQQQLLGETNAQQERLGDGALGMAEMPYNLFDKCIWFTVQLFLKQSCKELYHGYRAKFLMIVYWIAATYVLADVYSAQLTSQFARPPHEAPINTLQRLQTAMLRDGYQLFVEKESSSLEMLENGTEIFRQLYALMKQQNPDVEGYLIDSVEAGILLIADGLENKAVLGGRETLYFNIQQFGSKTFQLSHKLYTRYSAVAVQIGCPFLDSLNDVIIHLFEGGILDKMTNAEYATQSRMLGKEYNNQHPTSAAETNGNNEPPPSDDNRNGNGGSTDVGGKADESAGTATKSQDTQIIQPLNLRMLQGAFIVLLCGYAAATVILVLELCCHRLNWNFMERFQVRLLRRYRCVCRNFRRIAHSVFVRVFR

>CcapIR93a

MRFYATIWLFLLPFFLSLLPRNAEANDFSSFLTANASLAVVVDQEYMQRRGENILANFQKILSDTIRENLKNGGIEVKYFSWSQIRLKKDFLAAMTVTDCKSTWQFYESTQQTSILLIAITDANCPRLPLNRAIMIPIIDEGQELSQIILDIKVQQILRWKTAAVLLDQTILSDNPTLVESVVHESVKNHITPFSLLLYRIDDSLRSQKKRTVIRQMLMNFQDNVQTPRQFIVLSKFYEDIVEIAASLKLFHVYNQWLFFVLNEELRNYDAVSVTQNLEEGANIAFILNATEPTGTSSINCTIAELSMAFVTSISRMIVEEQSIYGEISDEEWEAIRYTKQEKQDEILGYMKDYIRMNSKCASCSHWKIETALTWGKSEEHRRYQTNLELRDTRNRNFEFIDVGYWTPTLGFITHEVMFPHIVHSFRNITMDILTVHSPPWQILERDSRGDIVRHSGITMEILKEMSRMLNFSYILHEVKTNPAELAAEEMQQTSNLTDDLLGSLTFNIPYQVIETMQSSRYFMAALAATIDEPDKKSFNYTVPISVQTYTFIARQPDEVSRIYLFTAPFTTEIWGCLVAIIIITAPVLYFINRLVPMEHLRITGLSTLNSCFWYIYGALLQQGGMYLPKADSGRLVIGVWWIVVIVLVTTYSGNLVAFLTFPQFQPGIDYFFQIFSSNDVRQFGLRNGSYFEKYSTITTRDDFRDFVQRAIIYNNVQGEDIGAVQDGKRVNVDWRVNLQLIVQRQFEKNKECKFALGRDSFVDEQIGLMVPKKSPYLQLINDQIMHMFRMGFIERWHQINLPSMDKCNGHGGMRQIMNHKVNLDDMQGCFMVLLFGFMIALFVLFVEYWYRWYFVEKKKATFTS

>CcapIRdelta-1

MTGFELILSAALCLSCAQQNDTSLPAGLIELDANGEVVTISPELAASEPSLDDAPLETVKTLLAKKEKIDKLKEWIKGRKLVIATLEDYPLSYTVMENDTRVGKGVAFELIDFLQDQLQFTYEVVVPEDNIIGSREDYEKSLIKMLNNSEADLAAAFIPALSEQHSFVFYSTTTLDEGEWIMVMQRPRESATGSGLMAPFDFWVWILIFISLLAVGPIIYMLIILRNRLTGDDEQKPYSLGHCAWFVYGALMKQGSTLSPIADSTRLLFATWWIFITILTSFYTANLTAFLTLSKFTLPYNTVSDILYKNKHFVSARGGGVEYAIRNTNESLSMLTNMIRNNHAVFSKSSNDTFNLQNFVEKDGYVFVRDRPAINHVLYADYRYRKTISMNDEKLHCPFAMAKEPFLKKNRSFAYPMGSNLSELFDPKLLNLVESGIIKYLSTKDLPNAEICPQNLAGTERQLRNTDLMMTYYIMFAGFATAMVVFFTELIFRYLNSRHEANKWARHGIGRTTNGLSVRAPRWLRQLETDSDKQRLTSSSNESTITPPPPYQSIFSSTNRHQENHHKDATHLSKDRSLNRWRRAGQFGNMIGGASGSQFGALAGGGGAGVLLGNGHFSGGNGGAGGVRRLINGRDYMVFRNPNGQSQLVPVRAPSAALFQYTYTE

>CcapIRNMDA2B

NNSSSSKGDTSQCSRYVNYGSRHCGRKGDSSSLIPTYNQHQHHQEQQHQHCLAPCAELTHKMPATISVTTQKPNVSVCVHQQHQRMVPNPLEGVHHRQRLQQQHQLTTTTKPTATSLPPTNQHAQPQQRHKQKQQKQQKQEQPATTATTVIACLRKSWSTFLSSRLNFHQHLGHISLTVLVLASILIYSCPCTSALRLTNGGNTKTLSANKEQLNIGLIAPHTNFGKREYLRAINTAVQGLAKTRGAKLTFLKDYSFEPRNIHFDMMSLTPSPTAILSTLCKEFLQANVSAILYMMNNEQFGHSTASAQYFLQLAGYLGIPVISWNADNSGLERRASQSTLQLQLAPSIEHQSAAMLSILERYKWHQFSVVTSQIAGHDDFVQAVRERVAEMQDHFKFTILNSIVVTRTSDLMELVNSEARVMLLYATQSEAVTILRAAEEMKLTGENYVWVVSQSVIEKKDAHPQFPIGMLGVHFDTSSAALMNEISNAIKIYAFGVEAYLTDPANRGRRLTTQSLSCEDEGRGRWDNGEIFFRYLRNVSIEGDLNKPNIEFTADGDLKSAELKIMNLRPGANNKNLVWEEIGVWKSWETQKLDIRDIAWPGNSHAPPQGVPEKFHLKITFLEEAPYINLSPADPISGKCLMDRGVLCRVAADHEMAADIDVGQAHRNESFYQCCSGFCIDLLEKFAEELGFTYELVRVEDGKWGTLENGKWNGLIADLVNRKTDMVLTSLMINTEREAVVDFSEPFMETGIAIVVAKRTGIISPTAFLEPFDTASWMLVGIVAIHAATFMIFLFEWLSPSGYNMKLYLQNTSVTPYRFSLCRTYWLVWAVLFQAAVHVDSPRGFTSRFMTNVWALFAVVFLAIYTANLAAFMITREEFHEFSGLNDSRLVHPFSHRPSFKFGTIPYSH

TDSTIHKYFKEMHHYMRQYNKTSVAEGVAAVLNGNLDSFIYDGTVLDYLVAQDEDCRLMTVGSWYAMTGYGLAFSRNSKYVQMFNRRLLEFRANGDLERLRRYWMTGTCRPGKQEHKSSDPLALEQFLSAFLLLMAGILFAALLLLLEHIYFKYVRKRLAKKDGCHCCALLSLSMGKALTFRGAVFEATEILKKHRCNDPICDTHLWKVKHELDMSRLRVRQLEKALDQHGIKPPQLRLASSSDLLNHHHLKERPPLLGNLSLAASAQDLYRWSYKTEIAEMETVL

>CcapIRNMDA1

MKVLNEINVVCLLTCSIFLGVTAQKHIQHSDNPSTYNIGGVLADPDSEAYFRRIISHLNFDQQYVPRKVTYYDKTIRMDKNPIKTVFNVCDKLIEKRVYAVVVSHEQTSGDLSPAAVSYTSGFYQIPVIGISSRDAAFSDKNIHVSFLRTVPPYYHQADVWLEIMFHFGYTKVIIIHSSDTDGRAILGRFQTTSQTNYDDIDVRATVEMIVEFEPKLDSFTENLIDMKTAQSRVYLLYASTEDAQVIFRDAALYNMTEAGHAWIVTEQALHANNTPVGVLGLVLEHANSDKGHIRDSVYVLASAIKEMMSNETITEAPKDCGDSGVNWESGKRLFQYLKTRNITGDTGQVAFDDNGDRIFAGYDVINIHEKQKKHVVGKFYYDGEKAKMRLRINDSEILWPGKQKKKPEGIMIPTHLKILTIEEKPFVYTRRLTDDEVTCDEDEIPCPLFNATDGSENENCCRGYCIDLLNALSHRINFTFDLALSPDGLFGHYTLKNVSSSSSGAIKSRKEWNGLIGELVNERADMAMPLTINPERAEFIEFSKPFKYQGITILEKKPSRSSTLVSFLQPFSNTLWILVMVSVHVVALVLYLLDRFSPFGRFKLTHAESNEEKALNLSSAIWFAWGVLLNSGIGEGTPRSFSARVLGMFWAGFAMIIVASYTANLAAFLVLERPKTKLSGINDARLRNTMENLTCATVKGSSVDMYFRRQVELSNMYRTMEANNYDTAEQAIQDVKKGKLMAFIWDSSRLEYEASKDCELVTAGELFGRSGYGIGLQKGSPWTDAVTLAILEFHESGFMEALDKHWIFHGNAQQCELFEKTPNTLGLQNMAGVFILVAAGVAGGVGLIIVEVIYKKHQVKKQKRLDIARHAADKWRGTIEKRKTLRASLAMQRQYNVGLNANPGTISLAVDKRRYPRMGPRAPEQAWKTDADILRNRRYLVEATKGGHTPVVHMPILGKMRPPTNMLPPRYSPAYTSDVSHLVV

>BcorIR8a

MWLLQKLVILFTFGYVCANELKIAFWIDPLQADIELDVASTVKEIEALQLGTKIQYYVVVITNVGKKKEAKNMEKLCEHLATDGVSVVIDFTYHIWREGLELLRTYQIPFLRVDRILAPYFKMFSQFVLQKSGHECIMIFQNARDTEEALIQIVEGYPFRSLIMNAFDNKQDFIKRLRKIRPMPSCYAIFADGTAMNSIFDRISKANFFERPREWHFVYLDPRDRVFKFKKQVDYATKFTINPKTLCRALRMKDTYCLSGFSFQRAMILEILRGLIELKQANLYWLPSFVMECNATSPIENGTAGLDILEQFPMSEFLDFTTDVTFADDEFDHVPRLTYTPTININLYSSEHDAVTELAIWQNDHLRKINETISPPRRFFRIGTVEAIPWNYMKRDPKTDELILDSFGNPIWEGFCIDSIQKLSERLDFGYMLVPPTSGEFGRRDVVNDVWDGIVGDLVTGETDFAVTALKMYSEREEVIDYIAPYFEQTGISIVMRKPVRQTSLFKFMTVLRVEVWFSIIAALVGSALMIWLLDKYSPYSYRNNRAAYQYPCREFTLRESFWFALTSFTPQGGGEAPKAVSGRIMVAAYWLFVVLMLATFTANLAAFLTVERMQTPVQSLEQLARQSRINYTVVEGSSTHQYFINMKFAEDTLYRMWKELTLNVTEDFQRYRIWDYPIKEQYGTILLAINGSEPVKNAKEGFRKVNEHENADFAFIHDSSEIKYELTRNCNLTEVGEIFAEQPYAIAIQQGSHFADELSYALLELQKDRFFEDLKAKYWNTSRIKACSVNEEQEGISLESLGGVFIATLFGLGLAMVTLVLEIIYYRRKYSTMQRFSEITKVKPASGTSIKQLLPKKKSKKRIAVWHTSTSKRDNSPEHKTPPPAFDAVKFRGKKVPPNITLGGQEFKPGRAGQRQLSESLDSAEYRNEGIPNRDDELPPYTE

>BcorIR92a

MWPTHTQTHVTTLLQLLIQRYFAQFSSVLIVHDGRVDANSALQREYLEAVQLAFRNLSQQGRLIGLQWIDVSQLDGDGAGKGSCRSSNCGDSYNNTCADNMAYNDELELCVLRAVDIVTEGFITILSDTVRFLHARYFATRNAELRLKDKFYLFFCEHERPEELLSTEILQFYPHHLMVTPETLTAQQSDNPQGFPNKTKVNSKYTHQITNTAATTIPTKPTTTTTPLSTFATASPSAHRDINIQLWTQKFVGASGNLEALLLDAFLPNETFARNAELYPNKVNNLRGRTIRVGSVTYIPYVVANYVPAGKGDVDALNSSDYSRTISYLGSEAELMKSFCEVRNCHIRLEPYGADNWGYIYENESATGMLGDVYTQNVEVAIGCIYNWYNNITETSNIIARSSVAILGPAPAQFPAWRANIMPFSNALWIFLILTILLCAAVMYLIRFVASLLDKWLRGVKCEFQHLTAFGQATLDMFAVFIQQPSGPTSLNTFAARFFLAMILCATITLENTYSGQLKSILTVPLFTEAVDTMEKWSKTDWTWSAPSIVWIQTIDSSNIEKEQIMSDKFEVRDYDFLYNASFRSDYGLGIERLMSGSFSFGDYVTAPALETKIVSKDDLYFDWTRAVSIRGWPLMPLFDKHIRACVETGLFVHWERKIVAKYLNQQTQEIMLNLASGHINKLPPQKLTIENISGATFALFFGCLIASFVFMLELTAYYFNKFQGLCIQRNGKSEN

>BcorIR84a

MAVPIGATTARRQTFASALLCGRSSLITTLILLLSKWNLCVATATNAYEFDAFADVLKQQHLHHAIIAYNGDTEQAQQQAGLLKDNALRALLNVASLQFYDVHQAKSATNATDFQSIFYHDSPRVGIYVAQLEDVLLQQYVLGSNVISVDTIDAGGYRVRVDVGSRFNSSRVWFIMSKQRKVTAALANVRRVMTPLPVNISADITIGVRLDDNNTIQLFDIYKIQKDWLDIEPKGYWSSAEGLKLNLRFHRTFVNRRRNFKGLQLVGGIVIREQPADMTELDYLNSLYHKNFDPMQRKTYQLVKLMEPVFDVSFQPVLRKTWGEQAPNGSWDGVMKLLLSGEAEFSLCPMRFVPNRVHLIHYTIAVHTEFVFFIFRHPHRNDIRNIFFEPFVEEVWYTVIAIVVLTILLLQLHLHHENRFFLNKDPHFQNRFDYAIFSILEAFFQQGPSTDAFTATSTRTLIFSVCLFSLLLQQFYGAYIVGSLLSVSPRTITNLEALYNSSLEIGIENIPYNIETFEKTTVPLGMAIYKERVCKNRERNILYIEEGAERIKKGGFAFHVSANRMYYMLKELLTEKEFCDLQDVPFIPPYRIGIGITKSSPFREYFTTTIAKFHTSGLLQHNDNQWQLPQMDCSLSQNYEVEVDLQHFLPALLFLVSAMLLSLAVLILEIIYYNLEKSTKLARLCPRIMPKPKLEFIN

>BcorIR76a

MSVLSPAVNHWTALLNFIMQTYFIDSHATCILWHHDFPFELQTPANGEFIQYINIWPDNLSQSLQQDIYNFTAFAETQLAHGVQPDALVQKLAIAIRESHCETFVAFQEDILSFARSFYNASRISVWRSLRNKFLFAYRKDLQQDTTAYFDDFLFMDQPNVLIVEAECGNCSTFALKTNKFIGPLAEHPEQLYVLDRYNGADGKFELGVDLYMDKVQNLQGREVTVGVFDYRPFAVVDYERQPQIKDHSPENLRGMAHVDGTEVRMLLALCEVVNCTVNADTSQDDWGTSYANLTADGIFGLVTSRKSHYAVGALYFWPDDYRYLDMSLFIGRSGVTCLVPSPHRLTSWLLPLRPFQLTLWLGVFACLGVEALALFFTRHLAPSDTEPQYGLMESFQFGYITTLKLFVSQGSDYVVNSHTVRMVLFACYMMDTIVTSVYGGGLSAILTLPTLEEASDSVERLYRHGIPWTATSPDWVISLKGADDDPIVEKLLQRYHVYTYEQLTEFAKTENMGFILERLAFGHFGNVDFLTDESFKRLKLMIDDIYYQYCYAFVPRLWALLPKLNDVIMQVHSTGLDIFWEWEVAATYMDGQQQEEIQASMYMDFDVGPVKLDMGNFIGLVLPLIIGSVFSIIAFIGELVYYKYTQKKAQAVIDVN

>BcorIR75d

MTPHFMLLICHIFLVAISSGAEPSDFAGAKQLGNSTLKEPTGEGYDAQIFMEYFRWHGVHNIMLIVCPQDVGTSEKHHKLKSLLRQFIAHGFSTRVFNGQDYDDDNESTRSAVQAQAEADPFNTTHTSIADSTSAAPAMSHFTFGPPRTFRSDNNTRRPLRLQLPALTYKSGILLLEFASACSINVLRWAAAAEHNYFTTNRFWLLFTDEPTHISLLDDGDIFLPPDGEVRVMLWQPGVQFFTLVDVYKVAADKPLRHTLVGGRELRDAEDMLQALGKFGSAISYRQNLEGITFKTGLVIAFPDLFTNIEDLSLRHIDTISKVNNRLTLELANKLNLRFNTHQVDNYGWHKPNGSFDGLMGRFQRYELDFAQMAIFMRLDRIAIVDFVAETYRIRAGIMFRQPPLSAVANIFAMPFASDVWIAILLLMIFTIGIFIVELVYSPHLHEMDILDCVVFVWGAMCQQGFYANLLNRSARVIIFTTFVSTLFLYTSFSANIVALLQSPSEAIQTLSDLTQSPLEVGVQDTQYNKIYFNESTDPVTNHLYRKKIAPKGENIFMRPAVGMEKMRTGLFAYQVELQAGYQIISNTFSEPEKCGLKELEPFQLPMIAVPTRKNFPYKELFRRQLRWQREVGLMNREELKWFPQKPKCEGGMGGFVSIGITECRYALGIFGFGLLLSAFSFILELVANYAWNLAKKIHRNKKQRMASSAADGYQGNFVH

>BcorIR75a.2

MEQLLLNFILHHFLNANVNSIVVLNCWSLQTQCDFSKMLNEHSLYSRFVNIETIDMSNDFEYLYLRHKRPGLGVYFDMNCKRAEQLLSILNHSRLYNGHYKWLLYDHTADIRNFKRLFDDANIGVDAELTYANLKPTLLGAFTDVNLTSFVKYDVYNNGYFLGGKLNMTVDCEVECNMKTCYTQRYLSELHTRSKYGNRDKMPDITMRVSVVVTKLPLSTPVPVLIDFLKSENNSNVDPITRFGFRILLIYKDLFGCKMRYTFRSHWGINETYGGGIGDLVTGKADFLSTPFLSTAERIKHVTPLIETGGFSSVCLFRTPRSSSTKGEAFLQPFDGTVWLVFVVLLLVVAVLLWRIFALELCNLRTHLPYKPSLLATVLLAFGSACYQGSSIVPASAGGRMAYFSLYFATFIVYNYYTSILLSTLLGTPPKSDIKTLGQLADSALPVGLEPLPYTYVYLNGSQLPDVRRFVYRKIELSKNPQKVWIPVEEGVLRVRDEPGFVFVLETSYAYPFLERNFLPHQICDLNQVNFRPDKSLFTQLHKNSSYKELTRLRAIRMLETGVFHKHRRYWVRNKLNCVPTNYLFAVGMEYTAPLFLMLVFSYLICLVILGVELLVKRF

>BcorIR25a

MPRAYLKFYNNIVIFLKILSLVSLATGQTNQNINVFFINDADNEPAAKAVTVVSTYLKKNPSYGISIQIDQVEANKTDAKTLLESICSKYAESIDRKQPPHVVFDTTKSGISSETVKSFTQALGLPTISASYGQEGDLRQWRDMDESKQKYLLQVMPPADLIPEVVRSIVRKMNITNAAILYDDTFVMDHKYKSLLQNIQTRHVITGIAKEGKREREEQIEKLRNLDINNFFILGNLMSIRMVLESVKPTYFERNFAWHAITQSEGEVSSQRDNATIMFLKPMSYAQNRDRFGRLKTTFNLNEEPQIMSAFYFDLALRTFLAIKDMLQSGAWPKNMEYIGCDEFQGGNTPERNLDLRTAFTMIQEPTSYGVFELVTQPGKSFNGYSYMKFEMDINVLQIRGGNSVNTKSIGTWTAGLDSPLVVKDEDVMKNLTADTVYRIFTVVQAPFIIKDEKAPKGYKGYCIDLINEIADIVHFDYTIQEVEDGKFGNMDEKGEWNGIVKKLMDKQADIGLGSMHVMAEREIVIDFTVPYYDLVGITIMMQRPQVPSSLFKFLTVLETNVWLCILAAYFFTSFLMWVFDRWSPYSYQNNREKYKDDDEKREFNLKECLWFCMTSLTPQGGGEAPKNLSGRLVAATWWLFGFIIIASYTANLAAFLTVSRLDTPVESLDDLAKQYKILYAPLNGSSAMTYFQRMANIEQRFYEIWKDLSLNDSLTPLERSKLAVWDYPVSDKYTKMWQAMQEAQLPATLEEAVERVRNSTSATGFAFLGDATDIRYLVMTNCDLQIVGEEFSRKPYAIAVQQGSHLKDQFNNAILTLLNKRQLEKFKEKWWKNDETQAKCDKPEDQSDGISIHNIGGVFIVIFVGIGMACITLVFEYWWYKYRKNPRIVDVIEANSGGKDGKTIDSVILGQAGKEYDKGGNTVLRPRFHQYPTTFKPRF

>BcorIRkainate 2

MARPYRQSMLHFITPTRCSRITLILLLALSCLQIAASQKINVGLIYESDNPDMEKIFQIAIDKANEESGGALELHGIAVAIEPGNAFETSKKLCKMLRQNLVAVFGPTTDLAAKHAMSICDAKELPFIDTRWDFAVQMPTVNLYPHASQLAVALKDLVVALEWTDTFTIIYETGEFLPTVNQLLEMYGTMGPTITVRRYELDLNGDYRNVLRRIKNSGDYSFVVVGSMATLPEFFKQAQQVGLMTDDYRYIVGNLDFQTMDLEPFQHGDTNITGIRLVSPDEKLVQDIAKTLYETEEPFQNVSCPLTTSMALVYDGVQLLAETFKHVMFRAVPLSCNDASSWDKGYTLVNYMKSLSLTGLTGEVKFDYEGLRTDFVLDVIELTMSGMQKIGEWKTEEGFFANRPPPKIVEVDQRSLVNKSFVVITAISEPYGMLKETPAKLEGNDQFEGFGIELIEELGKKLGFTYTFRLQVDNKYGSFNPKTGKYDGMMLEIIEGRADMGITDLTMTSIREEGVDFTIPFMNLGIAILFRKPMKEPPKLFSFMSPFSGTVWMWLGIAYMSVSLTLFILGRISPTEWDNPYPCIEEPTELENQFSFPNCLWFSIGALLQQGSELAPKAYSTRTVASIWWFFTLILVSSYTANLAAFLTIESLSSPIENAEDLANNKGDVKYGAKMGGSTYTFFQDAKYPTYQKMYEFMRDHPEYMTSTNAEGVDRVENENYAFLMESTTIEYITERRCSLTQVGSLLDEKGYGIAMRKNWPYRDILSQAVLELQEQGVLTKMKTKWWKEKRGGGACSDTSKEGGAVPLELSNLGGVYLVLIVGSCFGVLVAFLEMVLGVKERSDENKVSFKTELIEEFRFVMQCSGNTRPVKYPKNSSRSRSRSSRSRSHSRSSSKSSTLSVDSLPMDESKLHHISEHTKHAK

>BcorIRNMDA

MPVLNGINVVYLLFCGIHLGVIAQKHSQHSDNPSTYNIGGVLADPESESHFRTIISNLNFDQQYVPRKVTYYDKTIRMDKNPIKTVFNVCDKLIEKRVYAVVVSHEQTSGDLSPAAVSYTSGFYQIPVIGISSRDAAFSDKNIHVSFLRTVPPYYHQADVWLEIMFHFGYTKVIIIHSSDTDGRAILGRFQTTSQTNYDDIDVRATVEMIVEFEPKLDSFTEHLIDMKTAQSRVYLLYASTEDAQVIFRDAAINNMTEAGHAWIVTEQALHANNTPVGVLGLVLEHANSDKEHIRDSVYVLASAIKEMMSNETITEAPKDCGDSGVNWESGKRLFQYLKTRNITGKTGQVAFDDNGDRIYAGYDVINIHEKQKKHVVGKFYYDPEKAKMRLRINDSEILWPGKQKKKPEGIMIPTHLKILTIEEKPFVYTRRLTDDEVNCDEDEIPCPLFNATDGSENENCCRGYCIDLLNALSHRINFTFALALSPDGQFGHFTLKNVSSSSSGAITSRKEWSGLIGELVNERADMAMPLTINPERAEFIEFSKPFKYQGITILEKKPSRSSTLVSFLQPFSNTLWILVMVSVHVVALVLYLLDRFSPFGRFKLSHTDSNEEKALNLSSAVWFAWGVLLNSGIGEGTPRSFSARVLGMFWAGFAMIIVASYTANLAAFLVLERPKTKLSGINDARLRNTMENLTCATVKGSSVDMYFRRQVELSNMYRTMEANNYDTAEQAIQDVKKGKLMAFIWDSSRLEYEASKDCELVTAGELFGRSGYGIGLQKGSPWTDAVTLAILEFHESGFMEALDKHWIFHGNAQQCELFEKTPNTLGLQNMAGVFILVAAGVAGGVGLIIVEVIYKKHQVKKQKRLDIARHAADKWRGTIEKRKTLRASLAMQRQYNVGLNATPGTISFAVDKRRYPRMGPRAPEQAWKSDADILRNRRYLDDATKGGHSPAVHMPILGKMRPPTNMLPPRYSPAYTSNVSHLVV

>DmelIR25a

MGSRLDWGVADVALWAIADQIDYHQVFINEVDNEPAAKAVEVVLTYLKKNIRYGLSVQLDSIEANKSDAKVLLEAICNKYATSIEKKQTPHLILDTTKSGIASETVKSFTQALGLPTISASYGQQGDLRQWRDLDEAKQKYLLQVMPPADIIPEAIRSIVIHMNITNAAILYDDSFVMDHKYKSLLQNIQTRHVITAIAKDGKREREEQIEKLRNLDINNFFILGTLQSIRMVLESVKPAYFERNFAWHAITQNEGEISSQRDNATIMFMKPMAYTQYRDRLGLLRTTYNLNEEPQLSSAFYFDLALRSFLTIKEMLQSGAWPKDMEYLNCDDFQGGNTPQRNLDLRDYFTKITEPTSYGTFDLVTQSTQPFNGHSFMKFEMDINVLQIRGGSSVNSKSIGKWISGLNSELIVKDEEQMKNLTADTVYRIFTVVQAPFIMRDETAPKGYKGYCIDLINEIAAIVHFDYTIQEVEDGKFGNMDENGQWNGIVKKLMDKQADIGLGSMSVMAEREIVIDFTVPYYDLVGITIMMQRPSSPSSLFKFLTVLETNVWLCILAAYFFTSFLMWIFDRWSPYSYQNNREKYKDDEEKREFNLKECLWFCMTSLTPQGGGEAPKNLSGRLVAATWWLFGFIIIASYTANLAAFLTVSRLDTPVESLDDLAKQYKILYAPLNGSSAMTYFERMSNIEQMFYEIWKDLSLNDSLTAVERSKLAVWDYPVSDKYTKMWQAMQEAKLPATLDEAVARVRNSTAATGFAFLGDATDIRYLQLTNCDLQVVGEEFSRKPYAIAVQQGSHLKDQFNNAILTLLNKRQLEKLKEKWWKNDEALAKCDKPEDQSDGISIQNIGGVFIVIFVGIGMACITLVFEYWWYRYRKNPRIIDVAEANAERSNAADHPGKLVDGVILGHSGEKFEKSKAALRPRFNQYPATFKPRF

>DmelIR8a

MELPLLVLLLALRFAGSEVLKITFWIEPVQRAEFDTDIAMVLKELDALRLDVKVDDTTLTLTRSEDGLDMQRFCEILSTVGASAVIDLTYSHWEEGYNLVRSLGIGYVRLERIMRPFLDMFGDFMRQKRANNVAMVFMNARDAVEAMQQMLVGYPFRTLIMDASQTDPGQHFLERIRSLRPAPTYIALFARAAAMNGIFEKVQKADLFQRPLEWHFVFLDTRDRVFKYRRQAELCTRFTLNPRAICRSMPMPDLYCGSGFTMQRAMLLNVLRSLINAAQVSPGYPLAIYQDCNATASSSEVSDPLEKDDYNWLDMVHWSNFLAYAPPLPHIQDQFQSPVPGLTFAVNISAGYYSSEHEAKTDLAAWSSVGEMRLLNETISPARRFFRIGTAESIPWSYLRREEGTGELIRDRSGLPIWEGYCIDFIIRLSQKLNFEFEIVAPEVGHMGELNELGEWDGVVGDLVRGETDFAIAALKMYSEREEVIDFLPPYYEQTGISIAIRKPVRRTSLFKFMTVLRLEVWLSIVAALVGTAIMIWFMDKYSPYSSRNNRQAYPYACREFTLRESFWFALTSFTPQGGGEAPKAISGRMLVAAYWLFVVLMLATFTANLAAFLTVERMQTPVQSLEQLARQSRINYTVVKDSDTHQYFVNMKFAEDTLYRMWKELALNASKDFKKFRIWDYPIKEQYGHILLAINSSQPVADAKEGFANVDAHENADYAFIHDSAEIKYEITRNCNLTEVGEVFAEQPYAVAVQQGSHLGDELSYAILELQKDRFFEELKAKYWNQSNLPNCPLSEDQEGITLESLGGVFIATLFGLVLAMMTLGMEVLYYKKKQNALEITQVRPVNDSSGSGGNSSTAPPTATSTTKQAWHIPVLEAEEKPAKVSPPPSFETATFRGKKLPARITLGD

GKFKPRHGLYARRNLGASDSHSGYME

>DmelIR84a

MIKLQVKVISWPLIILTAFLRVLQIESINTNFLELAAFEDFLRSEHLSHVLVVRGDDADGDWKIECHQKLLANYRVQFYRPEMSANFEDLMFYGSPRTAVLVLNSEHVLVRRQVFGVASEAGYFNNSLAWFILGSGRESLPVEQLIDQLLSGYRMGIDADITVALRGPDNASMLFYDVYRISRQANTPLIIEKKGLWTHSGGYQKFGNFKNTWVIRRRNFLNVTLIGSTVLTEKPPGFGDMEYLADDKQLQQLDPMQRKTYQLFQLVERMFNLSLAISLTDKWGELLDNGSWSGVMGQVTSREADFAVCPIRFVLDRQPYVQYSAVLHTQNIHFLFRHPRRSHIKNIFFEPLSNQVWWCVLALVTGSTILLLFHVRLERMLSNMENRFSFVWFTMLETYLQQGPANEIFRLFSTRLLISLSCIFSFMLMQFYGAFIVGSLLSESARSIVNLQALYDSNLAIGMENISYNFPIFTNTSNQLVRDVYVKKICKSGEHNIMSLQQGAERIIQGRFAFHTAIDRMYRLLLELQMDEAEFCDLQEVMFNLPYDSGSVMPKGSPWREHLAHALLHFRATGLLQYNDKKWMVRRPDCSLFKTSQAEVDLEHFAPALFALALAMVASALVFLLELFLHWLPDFRRRLGTMST

>DmelIR76a

MENLLVESYYFSTVLSFFAQQFFADSHATCIFWHPAFDFRLETVHPMPLIIMDWHRWANRSDQDVYDYKIKEDEFEGKGIPYNDWTLRLTVAIERSHCETFIAFQEQIPEFARYFYHASIYSIWRSLRNRFMFVYTKEFEDKKDSYLSGYIFQDQPNILVITSQYLNSSTFEIKTNRFVGPRNFNKNPEPVEFYILQRFDAKGTKATWETQSAMSSKMRNLKGREVVIGIFDYKPFMLLDYEKPPLYYDRFMNTTDVTIDGTDIQLMLIFCELYNCTIQVDTSEPYDWGDIYLNASGYGLVGMILDRRNDYGVGGMYLWYEAYEYMDMTHFLGRSGVTCLVPAPNRLISWTLLLRPFQFVLWMCVMLCLLLESLALGITRRWEHSSVAAGNSWISSLRFGCISTLKLFVNQSTNYVTSSYALRTVLVASYMIDIILTTVYSGGLAAILTLPTLEEAADSRQRLFDHKLIWTGTSQAWITTIDERSADPVLLGLMEHYRVYDANLISAFSHTEQMGFVVERLQFGHLGNTELIENDALKRLKLMVDDIYFAFTVAFVPRLWPHLNAYNDFILAWHSSGFDKFWEWKIAAEYMNAHRQNRIVASEKTNLDIGPVKLGIDNFIGLILLWCFGMICSLLTFLGELWRGQG

>DmelIR64a

MHWWLLVFLPLSCQGLPEHELLELELDYGLAEPQRTSLLQSSLILQFSQDYKHIPRITYFTCQKPHLQTPNQIPNAAEHRDAFAAKNFQLIKSLYESELFVRIVLLDVLAQSPTSGRPNRPGNGPTGGFSQTPSQAQSNSEWLEGVLRMEALRQIAVVDLACGAVSRRFLELASAKMLYSEKFHWLLIEDFAWHGRTQTAEGSGKRDDGEMEEEEPPGQQIQATDDEDLPSIESFLGGMNLYMNTELTLAKRMSEAAHYTLFDVWNPGLNYGGHVNLTEIGSFTPTEGIQLHTWFRTTSTVRRRMDMQHARVRCMVVVTNKNMTGTLMYYLTHTMSGHIDTMNRFNFNLLMAVRDMFNWTFVLSRTTSWGYVKNGRFDGMIGALIRNETDIGGAPIFYWLERHKWIDVAGRSWSSRPCFI

FRHPRSTQKDRIVFLQPFTNDVWILIVGCGVLTVFILWFLTTIEWKLVPHDGSALIKPKGGAPPRHHYQQQQQQEQVEAPVRPITAVSVVVSKEKVEEKQEEYEDSTPIDAGTLWQRCYQKLNKYIKDRKAKQKKAPERVGLFLESVLFFVGIICQQGLGFSTSFVSGRCIVITSLLFSFCIYQFYSASIVGTLLMEKPKTIKTLSDLVHSSLKVGMEDILYNRDYFLHTKDPVSMELYAKKITSVPTTKENEADEDEPVDPNPVSTDPAKSYRDIVHSHETGAHAKDNAASNWLDPETGLLRHLGFAFHVDVAAAYKIIAETFSEQDICDLTEVSMFPPQKTVSIMQKNSPMRKVISYGLRRVTETGILTYHFNVWHSRKPPCVKKIETSDLHVDMDTVSSALLILLFSYAITLMILGTEILYSKWHNRIQLKWVGAT

>DmelIR93a

MNPGEMRPSACLLLLAGLQLSILVPTEANDFSSFLSANASLAVVVDHEYMTVHGENILAHFEKILSDVIRENLRNGGINVKYFSWNAVRLKKDFLAAITVTDCENTWNFYKNTQETSILLIAITDSDCPRLPLNRALMVPIVENGDEFPQLILDAKVQQILNWKTAVVFVDQTILEENALLVKSIVHESITNHITPISLILYEINDSLRGQQKRVALRQALSQFAPKKHEEMRQQFLVISAFHEDIIEIAETLNMFHVGNQWMIFVLDMVARDFDAGTVTINLDEGANIAFALNETDPNCQDSLNCTISEISLALVNAISKITVEEESIYGEISDEEWEAIRFTKQEKQAEILEYMKEFLKTNAKCSSCARWRVETAITWGKSQENRKFRSTPQRDAKNRNFEFINIGYWTPVLGFVCQELAFPHIEHHFRNITMDILTVHNPPWQILTKNSNGVIVEHKGIVMEIVKELSRALNFSYYLHEASAWKEEDSLSTSAGGNESDELVGSMTFRIPYRVVEMVQGNQFFIAAVAATVEDPDQKPFNYTQPISVQKYSFITRKPDEVSRIYLFTAPFTVETWFCLMGIILLTAPTLYAINRLAPLKEMRIVGLSTVKSCFWYIFGALLQQGGMYLPTADSGRLVVGFWWIVVIVLVTTYCGNLVAFLTFPKFQPGVDYLNQLEDHKDIVQYGLRNGTFFERYVQSTTREDFKHYLERAKIYGSAQEEDIEAVKRGERINIDWRINLQLIVQRHFEREKECHFALGRESFVDEQIAMIVPAQSAYLHLVNRHIKSMFRMGFIERWHQMNLPSAGKCNGKSAQRQVTNHKVNMDDMQGCFLVLLLGFTLALLIVCGEFWYRRFRASRKRRQFTN

>DmelIRGluRIIA

MRLCPVVIYAFIIIIGFLEGIIALGGDDRNEITVGAIFYENEKEIELSFDQAFREVNNMKFSELRFVTIKRYMPTNDSFLLQQITCELISNGVAAIFGPSSKAASDIVAQIANATGIPHIEYDLKLEATRQEQLNHQMSINVAPSLSVLSRAYFEIIKSNYEWRTFTLIYETPEGLARLQDLMNIQALNSDYVKLRNLADYADDYRILWKETDETFHEQRIILDCEPKTLKELLKVSIDFKLQGPFRNWFLTHLDTHNSGLRDIYNEDFKANITSVRLKVVDANPFERKKTRLTKVDQILGNQTMLPILIYDAVVLFASSARNVIAAMQPFHPPNRHCGSSSPWMLGAFIVNEMKTISEDDVEPHFKTENMKLDEYGQRIHFNLEIYKPTVNEPMMVWTPDNGIKKRLLNLELESAGTTQDFSEQRKVYTVVTHYEEPYFMMKEDHENFRGREKYEGYAVDLISKLSELMEFDYEFMIVNGNGKYNPETKQWDGIIRKLIDHHAQIGVCDLTITQMRRSVVDFTVPFMQLGISILHYKSPPEPKNQFAFLEFAVEVWIYMIFAQLIMTLAFVFIARLSYREWLPPNPAIQDPDELENIWNVNNSTWLMVGSIMQQGCDILPRGPHMRILTGMWWFFALMMLSTYTANLAAFLTSNKWQSSIKSLQDLIEQDKVHFGSMRGGSTSLFFSESNDTDYQRAWNQMKDFNPSAFTSTNKEGVARVRKEKGGYAFLMETTSLTYNIERNCDLTQIGEQIGEKHYGLAVPLGSDYRTNLSVSILQLSERGELQKMKNKWWKNHNVTCDSYHEVDGDELSIIELGGVFLVLAGGVLIGVILGIFEFLWNVQNVAVEERVTPWQAFKAELIFALKFWVRKKPMRISSSSDKSSSRRSSGSRRSSKEKSRSKTVS

>DmelIR75a

MQLVQLANFVLDNLVQSRIGFIVLFHCWQSDESLKFAQQFMKPIHPILVYHQFVQMRGVLNWSHLELSYMGHTQPTLAIYVDIKCDQTQDLLEEASREQIYNQHYHWLLVGNQSKLEFYDLFGLFNISIDADVSYVKEQIQDNNDSVAYAVHDVYNNGKIIGGQLNVTGSHEMSCDPFVCRRTRHLSSLQKRSKYGNREQLTDVVLRVATVVTQRPLTLSDDELIRFLSQENDTHIDSLARFGFHLTLILRDLLHCKMKFIFSDSWSKSDVVGGSVGAVVDQTADLTATPSLATEGRLKYLSAIIETGFFRSVCIFRTPHNAGLRGDVFLQPFSPLVWYLFGGVLSLIGVLLWITFYMECKRMQKRWRLDYLPSLLSTFLISFGAACIQSSSLIPRSAGGRLIYFALFLISFIMYNYYTSVVVSSLLSSPVKSKIKTMRQLAESSLTVGLEPLPFTKSYLNYSRLPEIHLFIKRKIESQTQNPELWLPAEQGVLRVRDNPGYVYVFETSSGYAYVERYFTAQEICDLNEVLFRPEQLFYTHLHRNSTYKELFRLRFLRILETGVYRKQRSYWVHMKLHCVAQNFVITVGMEYVAPLLLMLICADILVVVILLVELAWKRFFTRHLTFHP

>DmelIR21a

MSYYWVALVLFTAQAFSIEGDRSASYQEKCISRRLINHYQLNKEIFGVGMCDGNNENEFRQKRRIVPTFQGNPRPRGELLASKFHVNSYNFEQTNSLVGLVNKIAQEYLNKCPPVIYYDSFVEKSDGLILENLFKTIPITFYHGEINADYEAKNKRFTSHIDCNCKSYILFLSDPLMTRKILGPQTESRVVLVSRSTQWRLRDFLSSELSSNIVNLLVIGESLMADPMRERPYVLYTHKLYADGLGSNTPVVLTSWIKGALSRPHINLFPSKFQFGFAGHRFQISAANQPPFIFRIRTLDSSGMGQLRWDGVEFRLLTMISKRLNFSIDITETPTRSNTRGVVDTIQEQIIERTVDIGMSGIYITQERLMDSAMSVGHSPDCAAFITLASKALPKYRAIMGPFQWPVWVALICVYLGGIFPIVFTDRLTLSHLMGNWGEVENMFWYVFGMFTNAFSFTGKYSWSNTRKNSTRLLIGAYWLFTIIITSCYTGSIIAFVTLPAFPDTVDSVLDLLGLFFRVGTLNNGGWETWFQNSTHIPTSRLYKKMEFVGSVDEGIGNVTQSFFWNYAFLGSKAQLEYLVQSNFSDENISRRSALHLSEECFALFQIGFLFPRESVYKIKIDSMILLAQQSGLIAKINNEVSWVMQRSSSGRLLQASSSNSLREIIQEERQLTTADTEGMFLLMALGYFLGATALVSEIVGGITNKCRQIIKRSRKSAASSWSSASSGSMLRTNAEQLSHDKRKANRREAAEVAQKMSFGMRELNLTRATLREIYGSYGAPETDHGQLDIVHTEFPNSSAKLNNIEDEESREALESLQRLDEFMDQMDNDGNPSSHTFRIDN

>DmelIRNMDA1

MAMAEFVFCRPLFGLAIVLLVAPIDAAQRHTASDNPSTYNIGGVLSNSDSEEHFSTTIKHLNFDQQYVPRKVTYYDKTIRMDKNPIKTVFNVCDKLIENRVYAVVVSHEQTSGDLSPAAVSYTSGFYSIPVIGISSRDAAFSDKNIHVSFLRTVPPYYHQADVWLEMLSHFAYTKVIIIHSSDTDGRAILGRFQTTSQTYYDDVDVRATVELIVEFEPKLESFTEHLIDMKTAQSRVYLMYASTEDAQVIFRDAGEYNMTGEGHVWIVTEQALFSNNTPDGVLGLQLEHAHSDKGHIRDSVYVLASAIKEMISNETIAEAPKDCGDSAVNWESGKRLFQYLKSRNITGETGQVAFDDNGDRIYAGYDVINIREQQKKHVVGKFSYDSMRAKMRMRINDSEIIWPGKQRRKPEGIMIPTHLRLLTIEEKPFVYVRRMGDDEFRCEPDERPCPLFNNSDATANEFCCRGYCIDLLIELSKRINFTYDLALSPDGQFGHYILRNNTGAMTLRKEWTGLIGELVNERADMIVAPLTINPERAEYIEFSKPFKYQGITILEKKPSRSSTLVSFLQPFSNTLWILVMVSVHVVALVLYLLDRFSPFGRFKLSHSDSNEEKALNLSSAVWFAWGVLLNSGIGEGTPRSFSARVLGMVWAGFAMIIVASYTANLAAFLVLERPKTKLSGINDARLRNTMENLTCATVKGSSVDMYFRRQVELSNMYRTMEANNYATAEQAIQDVKKGKLMAFIWDSSRLEYEASKDCELVTAGELFGRSGYGIGLQKGSPWTDAVTLAILEFHESGFMEKLDKQWIFHGHVQQNCELFEKTPNTLGLKNMAGVFILVGVGIAGGVGLIIIEVIYKKHQVKKQKRLDIARHAADKWRGTIEKRKTIRASLAMQRQYNVGLNSTHAPGTISLAVDKRRYPRLGQRLGPERAWPGDAADVLRIRRPYELGNPGQSPKVMAANQPGMPMPMLGKTRPQQSVLPPRYSPGYTSDVSHLVV

>DmelIRNMDA2

MMPSRVKLKRGTDGPTPTPTPMPTTMRKHTPIATLNTASCQHNSTTSRRKRILTPPSGPISLLLLTVLTLLILDTRSCQGLRLTNGGGSLSKGAAANKEQLNIGLIAPHTNFGKREYLRSINNAVTGLTKTRGAKLTFLKDYSFEQKNIHFDMMSLTPSPTAILSTLCKEFLRVNVSAILYMMNNEQFGHSTASAQYFLQLAGYLGIPVISWNADNSGLERRASQSTLQLQLAPSIEHQSAAMLSILERYKWHQFSVVTSQIAGHDDFVQAVRERVAEMQEHFKFTILNSIVVTRTSDLMELVNSEARVMLLYATQTEAITILRAAEEMKLTGENYVWVVSQSVIEKKDAHSQFPVGMLGVHFDTSSAALMNEISNAIKIYSYGVEAYLTDPANRDRRLTTQSLSCEDEGRGRWDNGEIFFKYLRNVSIEGDLNKPNIEFTADGDLRSAELKIMNLRPSANNKNLVWEEIGVWKSWETQKLDIRDIAWPGNSHAPPQGVPEKFHLKITFLEEAPYINLSPADPVSGKCLMDRGVLCRVAADHEMAADIDVGQAHRNESFYQCCSGFCIDLLEKFAEELGFTYELVRVEDGKWGTLENGKWNGLIADLVNRKTDMVLTSLMINTEREAVVDFSEPFMETGIAIVVAKRTGIISPTAFLEPFDTASWMLVGIVAIQAATFMIFLFEWLSPSGYDMKLYLQNTNVTPYRFSLFRTYWLVWAVLFQAAVHVDSPRGFTSRFMTNVWALFAVVFLAIYTANLAAFMITREEFHEFSGLNDSRLVHPFSHKPSFKFGTIPYSHTDSTIHKYFNVMHNYMRQYNKTSVADGVAAVLNGNLDSFIYDGTVLDYLVAQDEDCRLMTVGSWYAMTGYGLAFSRNSKYVQMFNKRLLEFRANGDLERLRRYWMTGTCRPGKQEHKSSDPLALEQFLSAFLLLMAGILLAALLLLLEHVYFKYIRKRLAKKDGGHCCALISLSMGKSLTFRGAVFEATEILKKHRCNDPICDTHLWKVKHELDMSRLRVRQLEKVMDKHGIKAPQLRLASSSDLLNHHHLKERPPLLGNLSLAASAQDLYRSYKTEIAEMETVL

>DmelIRGluRIIC

MWQRILLLGCMWSAFFMCRSRGQQINIGAFFYDDELELEKEFMTVVNAINGPESEQTMRFYPLIKRLKPEDGSVTMQEHACDLIDNGVAAIFGPSSKAASDIVALVCNSTGIPHIEFDISDEGIQAEKPNHQMTLNLYPAQAILSKAYADIVQNFGWRKFTIVYDADDARAAARLQDLLQLREVHNDVVRVRKFHKDDDFRVMWKSIRGERRVVLDCEPNMLVELLNSSTEFGLTGQYNHIFLTNLETYTDHLEELAADNETFAVNITAARLLVNPDPPPYSLPYGYVTQRDNIVYESSDPPRTLIHDLIHDALQLFAQSWRNASFFYPDRMVVPRITCDFAASGGRTWAMGRYLARLMKGTSGVNNTNFRTSILQFDEDGQRITFNIEVYDPLDGIGIAIWDPRGQITQLNVDVKAQKKMIYRVATRIGPPYFSYNETARELNLTGNALYQGYAVDLIDAIARHVGFEYVFVPVADQQYGKLDKETKQWNGIIGEIINNDAHMGICDLTITQARKTAVDFTVPFMQLGVSILAYKSPHVEKTLDAYLAPFGGEVWIWILISVFVMTFLKTIVARISKMDWENPHPCNRDPEVLENQWRIHNTGWLTVASIMTAGCDILPRSPQVRMFEATWWIFAIIIANSYTANLAAFLTSSKMEGSIANLKDLSAQKKVKFGTIYGGSTYNLLADSNETVYRLAFNLMNNDDPSAYTKDNLEGVDRVRKNRGDYMFLMETTTLEYHREQNCDLRSVGEKFGEKHYAIAVPFGAEYRSNLSVAILKLSERGELYDLKQKWWKNPNASCFEEPDPDATPDMTFEELRGIFYTLYAGILIAFLIGITEFLVYVQQVALEERLTFKDAFKKEIRFVLCVWNNRKPIVAGTPISSVRTTPRRSLDKSLDRTPKSSRRVVIGRSSEEMREMAQGSGSSSGSNNAGRGEKEARV

>DmelIR41a

MFIDLSWSLVLSAIVGKYLNESTICIFWNDKFEFQLLHKSDYISFVGINIKSFDDNGGHYIIDTGLKKKELQNKHLFLDELVIKIIISIEVTHCETFVVFDKDIDRFVNAFNKASVYSIWRSLHNKFVFAHIANESPESRNHFFEDQPNILFVVRDHSSASSFDIKTNKFVGRKAENPSQMILVDRYLASEQRFQFGKSLFADKLNNLQGREVIIAGFDYPPYTVIKHNMSTNAQDMGVSGESDFKNVYIDGTETRIVLNFCEQFNCTIQIDSSAANDWGKVYPNMSGDGALGMLINRKADICIGAMYSWYEDYTYLDLSMYLVRSGITCLVPAPLRLTSWYLPLEPFKETLWAAILLCLCAEATGLVLAYKSEQALYVLPGYREGWWTCTSFGVCTTFKLFISQSGNSKAYSLTVRVLLFACFLNDLIITSIYGGGLASILTIPSMDEAADTVTRLRFHRLQWAANSEAWVSAIRASDEALVKDILYNFHIYSDDELLRLAQDQHMRIGFTVERLPFGHFAIGNYLGPQAIDQLVIMKDDIYFQYTVAFVPRLWPLLDKLNTLIYSWHSSGFDKYWEYRVVADNLNLKIQQQVQETMTGTKDIGPVPLGMSNFAGFIIVWILGSAIATLTFLLELSLTYILKQSNLK

>DmelIRGluRIIB

MHGLQFLVLLALAIASGANEDTLVIKIGAIFFDTEMKLADAFSAALEEVNAINPALKLDAIKRYVTVDDSIVLQDISCDLIGSGVAAIFGPSSKTNSDIVEVLCNMTGIPHLQFDWHPQQSNRERMNHQLTVNVAPMELFLSAAFSDILASKTFDWKSFTIAYERSSHLIRLQHILAWKQLHKAGIKMQEFERGDDYRILWKRINNAREKFVLLDCPSDILVDVINASIGYNMTGSFNHLFLTNLDTHLSGIDGFYSRDFTVAVAAVRIRTYVPPPVHDEIDVFDNSVDTRFSSLGSQLVYDSIVLFYNALLEISQRPGFYIPNFSCGRGFWQPGPRLVEQMKQITPKMVKPPFKTQRLQINADGQREDFNLEVYNPIIDRVTHIWNKEFQLVDFEKLRENSTQALKQKRLQNKEDFSQKPIRYTVATRVGKPYFSWREEPEGVHYEGNERFEGYAVDLIYMLAQECKFDFNFEPVRDNKYGSYDANTDEWDGIIRQLIDNNAQIGICDLTITQARRSVVDFTVPFMQLGISILSYKEPPPKADIYAFLNPYNAEVWLFVMIAMMITAFALIFTGRIDQYEWDQPVENVNREMERQNIWHLSNALWLVLGSMLNQGCDLLPRGLPMRLLTAFWWIFALLISQTYIAKLAAFITSSKIAGDIGSLHDLVDQNKVQFGTIRGGATSVYFSESNDTDNRMAWNKMLSFKPDAFTKNNEEGVDRVKLSKGTYAFLMETTNLQYYVQRNCELTQIGESFGEKHYGIAVPLNADFRSNLSVGILRLSERGELFKLRNKWFNSNESTCDSNVPTIDDGQFDMDSVGGLFVVLIVGVVVGLVIGVAEFLWHVQRISVKEKIPPMLALKAEFYFVIRFWLTRKPLHTYRQSRDSTSTGYSSLEQITSASSAKKKKKTRRIEK

>DmelIR94c

MSKVFKLLVLPLIYLSLTKGSKNPQLKFLRELINVIEEGREIRTIMVIKHSRDEYCHLDQWNPRGSPILRTNEMGSIRISGYFNDQAVILACMGENSDYGLLKSLANAMDNMRQERIILWSEREPTKMLMDYISQQADRYNFAQIIIVTMNEDVDAVPSLHQLNPYPTPRFRQITNISNIRRTSFFGCGLSFQGKTAILKESVVSNIRFKVWSPSGPIPLSELKDYEIVQFAVKYNLSLKLYDQNESKSDHFDIQLGPLFITKDFPTQMAFVSPNTACSLIVIVPCSPKWRFMDVLHKLGVLKLIGCLLIAYAVFVLIETLILWLTHRISGREVRLTSLNQLLNPRAFRGILGLPFPEFRRSSISLRQLFLVISVFGLVYSNFVSCTLSALLTKPAQNPQVRNFKELRDSGLITIMDKYTHSFIEKHIDPEFFDHVLPHYLILQKKEALRMIWNFNDSYSYVMYTTTWKSLNTVQKSFDERVFCESESLTIAWNLPRMYVLGNNSVLKWMLSRYITYMPQTGIPDSWTEQLPKVLKLLYNVTSPRRIKEGAVPLSIQHLSWIWHLLFIGESIATLVFIVEILLQKSNQHTSNMRERSSEDDDFV

>DmelIR54a

MWTVITGIVLWAPVLVAGSAVDFIFRAAAEHSLSVIMIRIDYCPYNWAKDIFENQTIPVVVLSDSETFINIRMFSRPLHVACLPGHELQKDLALLENFTSSLMDFPSQKKIVYISNNFSDPTRMDYIFETCYHRRIWNIVGLLASDEHRYFYRYHLYPSFRTEYRSLESSTIFDKDFPNMHGHPLTVMPDQWLPRSVLYVDRRTGKQILAGSVGRFFHVLSWKLNATLQLSKKVTTGRFLNATALKELSESFSVDVPASLTIMERVEQLASTSYPMEVTHVCLMVPVARRIPIKDIYFILSSASNMFLAIVIVSSYGLALNLLRNMTHRDVRLVDFVLNDKALRGILGQSFNLPLSRSFSTRLIFLMLGIVGLNVSSIFGAGLDTLMAHPPRQFQARSFAGLRRTKIPLVTTEEDFPTWMKLRVPMLVVNVSEYNHLRNGRNTSNAYFASRLYWNLFSEQQKRFTRELFIYSTDDCLWSLALLSFQWPQNSLFTEPVSQLILEVNANGLYDFWVGMHYYDMTAAGLSGLEDPSLQLKEREHPTSLRIVDFQWMWQAYGTFMVIAILVFLLEVSWHRITSLFVSLVY

>DmelIR10a

MAVLGTVFLLFMLDLKTLNLTRLNGLLVEPTRDLPQLELWLRAGSDHQDAENPYVQWFLLRTEIPLSIVTYQENRYWMDDPFGRRNLVLVMSLDQLLTNRGAAAPIQKASTFFYILADQDKDLSADEQLRLEGSCRQLWTQHKVYNRFFLTRDGVWIYDPFKRRDSAFGRLVRYYGSETLDKLLFRDMAGYPLRIQMFRSVYTRPEFDKETGLLTRVTGVDFLVAQMLRERLNFTMLLQQPEKKYFGERSANGSYNGAIGSIIKDGLDICLTGFFVKDYLVQQYMDFTVAVYDDELCIYVPKASRIPQSILPIFAVGYDIWLGFVLTAFACALIWLTLRVINLKLRIVSLGNQHIVGQALGIMVDTWVVWVRLNLSHLPASYAERMFIGTLCLVSVIFGAIFESSLATVYIHPLYYKDINTMQELDESGLKVVYKYSSMADDLFFSETSPLFASLNKKLSWNRDLRADVIDEVARFRNKAGVSRYTSLILESSHFTLLRKIWVVPECPKYYTISYVMPRDSPWEDAVNALLLRFLNAGLIVKWIQDEKSWVDIKMRSNILEADAESELVRVLTIGDLQLAFYVVIGGNLLAFLGFLAEHFRWKLQKKGV

>DmelIR62a

MYLQFLFALFLSRYQIVATENFDRAFELALFLDRIGRVHRLHAITIVNSLGSVDPSYLDDLHRGLMCNSSNHFYMLPQMTATDKDSSHVHFSSLQDEETIYLVFARDSKDAVIYLQAERARGRRYTRTMFLLRKQESQKDIKYFFELLWKLQFRSALVVVAARNFYQMDPYPTVRVIRMRRLSSYDPHHVFPPANRKNFRGYRMRLPVQQDVPNTFWYKNRRTKAWELAGLGGILINQLMMHLNVTMDLFRFEVNGSSLLNMAALTDLIVKGKVELSPHLYDTLQSNTSVDYSYPTQVAPRCFMIPLDNEISRSLYVFLPFSLTMWLCLLFVLLVVHFVYVRRLIPDGHFWAILGVPGAGQVRYGNRKPVRRFSTFLILFGIFILGQTYSTKLTSSLTVTLIRRPDNSLEELFLLPYRILVLPTDVYAIVDSLGHAEQFSTKFSCTDAENFSQKRISMHPEYIYPISTIRWRFFDMQQRFLRKKRFYFSKICHGSFPYQYQLRVDSHLKDALHRFLLHVQQAGLHDLWLDTCYRKAHRMGYLKDFSTLAELEEKLRLRPLALNLLVPAFSLFLCGMLGSGIAFLVEIRHSFGCRQKPPSINRNPGD

>DmelIR100a

MATTLQLIMLALVGGTLGQANNTDHKQVLTSIVKQLEGGLELHLRTSEDGGNDLVQFLMQEKSSIIISAKQEEVPSRAKIMRHHFFIFDGVHQMQEIRTSLFNTDGFYILALENNTIEDDVLLMEFAADVWLQHGHSRIYYVQLSKKSVLLFNPFLQRLVVVQDSKTYSRIYKDLEGYHLRIYIFDSVYSSVIGDGENKVLSVTGADAKLAKTVARQLNFTADFVWPDDEFFGGRLANGEYSGGVGRAHRGEVDIIFAGFFIKDYLTTHIQFSAAVYMDELCLYVKKAQRIPQSILPLFAVHMDVWLCFLLVGLLGALVWLILRAVNLILGIEGVPDGSRATRISYFGAARRIFVDTWVIWVRVNVGRFPPFHSERIFVASLCLVSVIFGALLESSLATVYIRPLYYRDVNTLRELDESGQPIYIKHPAFKDDLFYGHNSEVYRRLDAKMMLVAEGEERLIEMVSKRGGFAGVTRSASLQLSDIRYVMTKKVHKIPECPKNYHIAYVLPRPSPYLEEVNRIVLRLVAGGIVGLWTGEAKERAKWSIQRFPEYLAELDVGRWKVLTLSDVQLAFYALTIGCLLSAIVCMAEILLGRQRRLHSPK

>DmelIR75c

MTSWPLYRLIVFNLLEINLSNLMVFHCWSIKEAFPLVEMLNQNGIFSQYIDVQNPDNLANVHKEYLDSDLVRLGVFLDLGCDKAELVTNQSSRARLYNQNLHWLLYDEAGNFTKLTQLFEGANLSLNADVTYVSREDEERFILHDVYNKGSHLGGKLNITVDQTLQCNRSHCQVKEYLSELHLRPRLQHRMDLSSVTFRLAALVSVLPINSSEEELLEFLNSDRDSHMDSISRIGNRLIMHTQEILGFKLHYIWCGTWSVQDAFGGAIGMLTNESAELCTTPFVPSWNRLHYLHPMTEQAQFRAVCMFRTPHNAGIKAAVFLEPFMPSVWFAFAGLLIFAGVLLWMIFHLERHWMQRCLDFIPSLLSSCLISFGAACIQGSYLMPKSAGGRLAFIAVMLTSFLMYNYYTSIVVSTLLGSPVRSNIRTIQQLADSSLDVGFDTVPFTKTYLVSSPRPDIRSLYKQKVESKRDPNSVWLSPEEGVIRVRDQPGFVYTSEASFMYHFVEKHYLPREISDLNEIILRPESAVYGMVHLNSTYRQLLTQLQVRMLETGITSKQSRFFSKTKLHTFSNSFVIQVGMEYAAPLFISLLVAYFLALLILILEICWARYAKKKFSTIIPQNQ

>DmelIR7c

MLHSAVHNVSLVYALVWAIDNYYGMATSTPLAVVQFPTSRESRRLHNDLIDAALGRSSGTGRIQFLLEDDRVEMTETDTDPPPPSGLTGRPIAIWFLDSLRSYFRLEMYLNQLGSPYKRNGFFLVIYTGLEDQPMESLKIMFRRLLNMYVLNVNVFLQRDGTVHLYTYYPYGPHHCQSSLPVYYTAFQDLAAPANGFGLTKPLFPRKLTNMHGCEMVVATFEHRPYVIIEDDPKTPGGRSIHGIEGLIFRSLAERMNFTIKLVEQKDKNRGEILPDGNFTGILKMMVDGEVNLTFVCFMYSKARSDLMLPSTSYTSFPIVLVVPSGGSISPMGRLTRPFRYIIWSCILVSLIFGFVLICLLKITALPGLRNLVLGRRNRLPFMGMWASLLGGLALYNPQRNFARYILVMWLLQTLILRAAYTGQLYLLLQDVEMRSPIKSLSEVLAKDYEFRILPALRTIFKDSMPTTNFHAVLSLEESLYRLRDEDDPGITVALLQPTVNQFDFRSGPNKRHLTVLPDPLMTAPLTFYMRPHSYFKRRIDRLIMAMMSSGIVARYRKMYMDRIKRVSKRRNLEPKPLSIWRLSGIFVCCAGLYLVALIVFILEILTTNHRRLRRAFNVINRYAA

>DmelIRsubunitIB

MRFGLKLSCLWPSFLLWLTWSSGGGGGRWVGVSAQPSLTEKIPLGAIFEQGTDEVQSAFKYAMLNHNLNVSSRRFELQAYVDVINTADAFKLSRLICNQFSRGVYSMLGAVSPDSFDTLHSYSNTFQMPFVTPWFPEKVLTPSSGFLDFALSMRPDYHQAIIDTIQFYGWRKIIYLYDSHDGLLRLQQIYQGLRPGNESFQVELVKRISNVSMAIEFLHTLEQIGRFENKHIVLDCPTEMAKQILIQHVRDLRLGRRTYHYLLSGLVMDDRWESEIIEFGAINITGFRIVDTNRRLVREFYDSWKRLDPQMSVGAGRESISAQAALMYDAVFVLVEAFNKILRKKPDQFRNNVQRRSQTLMVAQAAASTSSDGYNYSASGGGGGNGGAGGGFAGSDSGGSGGMASRALDCNTAKGWVNAW

EHGDKISRYLRKVEIEGLTGDIKFNDDGRRVNYTLHVVEMTVNSAMVKVAEWNDDAGLQPLNAKYVRLRPHVEFEKNRTYIVTTVLEEPYIMLKQVAFGEKLHGNNRFEGYCKDLADLLAKELGINYELRLVKDGNYGSEKSSAHGGWDGMVGELVRKEADIAIAAMTITAERERVIDFSKPFMSLGISIMIKKPVKQTPGVFSFMNPLSQEIWVSVIFSYIGVSIVLFFVSRFSPHEWRLVQQQPQQSQSPDPHAHHEQLANQQPPGIIGGAPLPAPPGPPTPGAQTAAGAAALQAALSAGSPGSGGSSSAVVNEFSVWNSFWFSLAAFMQQGCDLSPRSVSGRIAAASWFFFTLILISSYTANLAAFLTVERMVTPINSPEDLAMQTEVQYGTLLHGSTWDFFRRSQIGLHNKMWEYM

NSRKHVFVPTYDEGIKRVRNSKGKYALLVESPKNEYVNAREPCDTMKVGRNLDTKGFGIATPLGSALKDPINLAVLTLKENGELIKLRNKWWYEKAECSTHKDGETSHSELSLSNVAGIFYILIGGLLVSVFVAILEYCFRSRDSRSASSGSGMGLGMGLGGGMSGGSLGKANGSMMLGPSSAVPGGMPSSHQRSTLTDTMHAKAKLTIQASRDYDNGRVGYLNCASLQYYPPAQLSATPPDAGDSLHMNAHGQV

>DmelIRsubunitIA

MHFCWISLIILSLSRVQAQFYGGNAYEASSGQSIRLGLITDDATDRIRQTFEHAISVVNNELGVPLVGETEQVAYGNSVQAFAQLCRLMQSGVGAVFGPAARHTASHLLNACDSKDIPFIYPHLSWGSNPDGFNLHPSPEDIANALYDIVNQFEWSRFIFCYESAEYLKILDHLMTRYGIKGPVIKVMRYDLNLNGNYKSVLRRIRKSEDSRIVVVGSTTGVAELLRQAQQVGIMNEDYTYIIGNLNLHTFDLEEYKYSEANITGIRMFSPDQEEVRDLMEKLHQELGESEPVNSGSTFITMEMALTYDAVRVIAETTKHLPYQPQMLNCSERHDNVQPDGSTFRNYMRSLEIKEKTITGRIYFEGNVRKGFTFDVIELQTSGLVKVGTWEEGKDFEFQRPPQAVNFNDIDDGSLVNKTFIVLISVATKPYASLVESIDTLIGNNQFQGYGVDLIKELADKLGFNFTFRDGGNDYGSFNKTTNSTSGMLKEIVEGRADLAITDLTITSEREEVIDFSIPFMNLGIAILYVKPQKAPPALFSFMDPFSSEVWLYLGIAYLGVSLCFFIIGRLSPIEWDNPYPCIEEPEELENQFTINNSLWFTTGALLQQGSEIAPKALSTRTISAIWWFFTLIMVSSYTANLAAFLTIENPTSPINSVKDLADNKDDVQYGAKRTGSTRNFFSTSEEPIYIKMNEYLNAHPEMLMENNQQGVDKVKSGTKYAFLMESTSIEFNTVRECNLTKVGDPLDEKGYGIAMVKNWPYRDKFNKALLELQEQGVLARLKNKWWNEVGAGVCSAKSDDDGPSELGVDNLSGIYVVLVIGSIISIIISILCWCYFVYKKAKNYEVPFCDALAEEFRIVIRFSENERPLKSAQSIYSRSRNSSQSIESLKTDSEENMPVED

>DmelIRkainate2

MLLRYLLVLLLPGIFGFKNIRIGVIFDGDPIIEDIFNISVKMVNRNRNSDHFLPAEKIIAEKRLVAADDVYDTSEKVCELLDVGVAAIFGPQSKASSSHVQSMCDAMEIPHVSTNMEPYAERLKAINMYPHVKTLSMVFYHLIKNFKWKTYTILYDTDDSLIRMTPLLTNYGTTGYTVTVRRLEGENYRPVLRDVKKSVEQNVILDCDIGILLEVLTQAQQVGLIADKYNFIVCTLDLHTLNIQPFQYSGMNFTAVRIVDPDDYTTRKIIEDFFQPKGITDASQFTLEQALMFDGVQLFARAFKQLSDAVKINIKKLPCNGLENWEHGISLSNFMRASEMRGLTGLVKFDTSGFRSDFQLDILNVGWNGIRKIGTWNSTTNIDWIPETTLFDPNADLSLRNVTFRVLISLTKPYAMLKESSFKKTGNDQFEGFGIDVIVELSKALGFNYTFILHDPEYGKFDNKTGQWTGMIRKIMDNDADLAITDLTITAERESVVDFTMPFMNLGISILFTKPKKASPSLMSFLSPFSNEVWLYVIGVYVFVSIEFFIIGRMCPDEWTNPYPCVEEPENLYNQFSLKNALWFTVGAMMQQGTELAPIGVSTRMLAGSWWFFCIIIVNSYTANLAACLTAETLVKRIENVEDLANQNEIKYGAKAGGSTLSFFANSANPTYKKMFKYMTDNAASVQFESNEEGRDKVLKGDYAFLMESASIEYLSERECDLTQVNGLLDQKGYGIAMRKNSTYRNELSSGVLKLQENGMLASLKTQWWKQKRGGGSCEKDTSTGAPTPLNLSNVGGVYLVLVVGIVFSFFYSLVELLWEIGYNSFKHNASIKEELMDEFKFILQFSGTSKPVKNRKSISSKSEKDSQDSTPPYGFVPAVVTTGPRNETET

>DmelIRkainate1

MTNKLFIFALLSIAFLMEVKTNLVGLEYAQLISYVHNIYDTSSVLIIHNDKTDNIAALGSIFDIVQQLSIEGIPSLTAKITQLLAVKNYYGQSGRRELFVIFIRQYETMKEFERIMNESFTIIGPVWLIIFRPLNNKQLLIDYCQSPRGNPFNVAFNTEMLIKCYDDPIIKEWYSIYKNETVMFNLAFWQPNEAFQLFTEKSLYQRRNNIGGITLRVITIEGSPMVENQFGKLGGFFGKIMEELSQSMNFKIEIIRNETLYGNWDERKKEWSGVIKHLYEKNVDFAVSDMVMSTRRLKAVDFTIPLITSRAILCFKEPNITSIQQLEYFKTFHNHVWSIIMLTTIVTTLILTTVKAKMRRIKSLRILFCENYLNVWRIFCQQGLLEVPKIMPLRLTYFSIFITALIISSVYSATLISYLTVPSTVLPFTTLEGFVKDGSYKLVVLKESVHYAMFQVCSSKTALYSTEETIILQKLPPNCKLITIQTGDINCLAMPISKKSQYTGIMNFHLGRFLDNGMLQHFKYKKKQINSINLQQKYNVVTFSSIETILKIVFIGIVSSAFIFIIELTHFHVTKNKLC

>DmelIRdelta-1

GFDLILSAALCLTCANLTDIRLPEGLIELDENNTVVTISPDLAVDEPSLDDAPLETVKTIVAKKEKMDKLREWIKGRKLVIATLEDYPLSYTVMENDTRVGKGVAFELIDFLQEQMQFTYEVVVPEDNIIGSREDYEKSLIKMLNNSEADLAAAFIPTLSEQHSFVFYSTTTLDEGEWIMVMQRPRESATGSGLMAPFDFWVWILIFISLLAVGPIIYMLIILRNRLTGDKEQKPYSLGHCAWFVYGALMKQGSTLSPIADSTRLLFATWWIFITILTSFYTANLTAFLTLSKFTLPYNTVSDILYKNKHFVSARGGGVEYAIRNTNESLSMLTNMI

>BdorIRkainate 2

MFFSFKRTFQMYSLILIVVKPYSSLPPVIRLGVLQLRKFGHMYDIETHLRLVNPTTYTKILSELKDKEIHNLIIDTNAANISILLKGILQLQMNEYKYHYLFTSFDIETFDLEDFKNNFANITSFRLVDVGDLSVRNILKNMEAYNYNYNNKTTYYRKLRTIETEPAVAYDSVYIFAIGLTSLQQSLTLSVSNASCASEIPWDGGLSLINYINSVEWRGLTGPIQFKEGRRVKFKLDLVKLRQHSLVKVGEWTPQTRLNITEPALFFDGGTINVTLVVITILYACSLQETPYVMMHYGKNYTGNERFYGFCVDILELIARDVGFDYIIDLVPDRKYGAQDPFTGEWNGMVAQLMKYKADLAVGSMTITYARESVIDFTKPFMNLGISILFKVPSSPASRLFSFMNPLAYDVWLYVLAAYFLVSFTIYVVAKLSPIEWRDKHPCDIKNPIVTNQFTLANSFWFTIGTLMQQGSDINPKSLSTRIVSAIWWFFTLIIIASYTANLAAFLTVERMITPIENAEDLASQTEISYGTLESGSTMTFFRDSIIETYKKMWRNMENKKSIAFTSTYEEGIKRVNQGNFAFLMESTMLDYIVQRDCNLTQIGGLLDTKGYGIATPKGSPWRDKISLSILEFQEKGNIQMLYDRWWKKAGDTCLRKSNSKQTKANALGLDNIEFSIEGGVFVVLFVGIGLAAWVAVFEFWYHYRSRRRASVYYENEHCTVERVMTEMTLNDFDSVYNSKKENVEEIQVKDQKLTCKHITAYCCKEPTAQRSLCGEMLDEFRYALRCMDSHRRPALKRSCPTCHILNDVKEFIDTESRSSMAVHGSLIEGTPKYL

>BdorIRdelta-1

MTGFDLILSAALCLTCANLTDIRLPEGLIELDENNTVVTISPDLAVDEPSLDDAPLETVKTIIAKKEKMDKLREWIKGRKLVIATLEDYPLSYTVMENDTRVGKGVAFELIDFLQEQMQFTYEVVVPEDNIIGSREDYEKSLIKMLNNSEADLAAAFIPTLSEQHSFVFYSTTTLDEGEWIMVMQRPRESATGSGLMAPFDFWVWILIFISLLAVGPIIYMLIILRNRLTGDKEQKPYSLGHCAWFVYGALMKQGSTLSPIADSTRLLFATWWIFITILTSFYTANLTAFLTLSKFTLPYNTVSDILYKNKHFVSARGGGVEYAIRNTNESLSMLTNMIRNNHAVFSSSSNDTFNLQNFVEKDGYVFVRDRPAINHVLYADYRYRKTISMNDEKLHCPFAMAKEPFLKKNRSFAYPLGSNLSELFDPKLLNLVESGIIKYLSTKDLPNAEICPQNLAGTERQLRNTDLMMTYYIMFAGFVTAMVVFFTEL

IFRYLNQRNEGSKWARHGVGRTTNGLSVRAPRWLRQLETDSDKQRLTASPSGSTITPPPPYQSIFSSNHRHHQQDEAGHLSKESSLHRWRRAGQFGAGGSNFGTLAAGAGSGAGVLLGNGQLHEGSGAGGVRRLINGRDYMVFRNPNGQSQLVPVRAPSAALFQYTYTE

>BdorIRNMDA2B

MIRKMKGLSFSQKANKRRKNVQTNDQTPSGNTALPTRAACTNNNNNKVNKLKICKTTAITQAHNGNAEQQLTTPLICRNNNKKNSCNSVGSQRRYCSSNSSKSTPPPPTFNQHHNHQHQHCLAPGQLTYKMPLPLLSISKTTTTTTALAVSANQQPQQQRQQQREQLESVQNRCLAHYKEQQRQRKTTTTTCAPTKLTQHQRQRQQPATTRALSCLSKSLATLSLRSLLSRHHQLFHFQQHLQQLHLHLHFGIAVIVLASILMYSCPCASALRLTNGGNTKTLSANKEQLNIGLIAPHTNFGKREYLRAINTAVQGLAKTRGAKLTFLKDYSFEPRNIHFDMMSLTPSPTAILSTLCKEFLQANVSAILYMMNNEQFGHSTASAQYFLQLAGYLGIPVISWNADNSGLERRASQSTLQLQLAPSIEHQSAAMLSILERYKWHQFSVVTSQIAGHDDFVQAVRERVAEMQDHFKFTILNSIVVTRTSDLMELVNSEARVMLLYATQSEAVTILRAAEEMKLTGENYVWVVSQSVIEKKDAQTQFPVGMLGVHFDTSSAALMNEISNAIKIYAFGVEAYLTDPANRGRRLTTQSLSCEDEGRGRWDNGEIFFRYLRNVSIEGDLNKPNIEFTADGDLKSAELKIMNLRPGANNKNLVWEEIGVWKSWETQKLDIRDIAWPGNSHAPPQGVPEKFHLKITFLEEAPYINLSPADPISGKCLMDRGVLCRVAADHEMAADIDVGQAHRNESFYQCCSGFCIDLLEKFAQELGFTYELVRVEDGKWGTLEDGKWNGLIADLVNRKTDMVLTSLMINTEREAVVDFSEPFMETGIAIVVAKRTGIISPTAFLEPFDTASWMLVGIVAIHAATFMIFLFEWLSPSGYNMKLYLQNASVTPYRFSLCRTYWLVWAVLFQAAVHVDSPRGFTSRFMTNVWALFAVVFLAIYTANLAAFMITREEFHEFSGLNDSRLVHPYSHKPSFKFGTIPYSHTDSTIHKYFKDMHHYMRQYNKTSVAEGVAAVLNGNLDSFIYDGTVLDYLVAQDEDCRLMTVGSWYAMTGYGLAFSRNSKYVQMFNKRLLEFRANGDLERLRRYWMTGTCRPGKQEHKSSDPLALEQFLSAFLLLMAGILFAALLLLLEHIYFKYVRKRLAKKDGCHCCALLSLSMGKALTFRGAVFEATEILKKHRCNDPICDTHLWKVKHELDMSRLRVRQLEKALDQHGIKPPQLRLTSSTDMLNHHHLKERPPLLGNLSLAASAQDLYRWSYKTEIAEMETVL

>BdorIR93a

MRFHAFLWSLWLSLCLALLLQNAAANDFSSFLTANASLAVVVDQEYMQRRGENVLASFQKILSDVIRENLKNGGIEVKYYSWSQIRLKKDFLAAMTVADCKSTWQLFDSTQQNSILLIAITDANCPRLPLNRAIMIPIVDEGQELSQIILDIKVQRLLRWKTAAVLLDQTILHDNPTLVESVVHESAKNHITPFSLLLYQIDDTLRSQKKRTAIRQMLSAFQDGGQTPRQFIVLSQFYEDIVEIAASMKLFHVYNQWVFFVLNEELRNHDPISVTQNLDEGANIAFALNTTEPTCSSSINCTITELSLALVTSISRMIVEEQSIYGEISDEEWEAIRYTKQEKQDEMLGYMKEYLREYSKCTSCSHWKIETALTWGKSEEHRRYQSNSELRDTRNKNFEFIDVGYWTPTLGFNTHEVMFPHITHFFRNITLNILTEHSPPWQILERNSRGDIVRHSGISMEILKEMSRMLNFSYILHEVKVSASDSAEDMQHTNNVTDDLFGSLTFNIPYQVIETMQASRYFMAALAATIDEPDKKSFNYTVPISVQMYTFISRQPDEVSRIYLFAAPFTTEIWGCLVAIIIITAPVLYFINRLVPMDHLRITGLSTLNSCFWYIYGALLQQGGMYLPKADSGRLVIGVWWIVVIVLVTTYSGNLVAFLTFPQFQPGIDYFFQIFSSSAVQQFGLRNGSYFEKYATQITTRDDFRDYVQRATIYNNVQGEDIGAVQDGKRINVDWRINLQLIIQKQFEKDKECKFSLGRDNFVAEQIGLIVPRDSPYLQLINDKIMRMFQMGFIERWHQINLPSMDKCSGHGGMRQIMNHKVNLDDMQGCFMVLLFGFFIALFILFVEYWYRWYFVEKKRGVFAT

>BdorIR25a

MPRAYLKFYNNIVIFLKILSLVSLTTGQTNQNINVFFINDADNEPAAKAVTVVSTYLKKNPSYGISIQIDQVEANKTDAKTLLESICSKYAESIDRKQPPHVVFDTTKSGISSETVKSFTQALGLPTISASYGQEGDLRQWRDMDESKQKYLLQVMPPADLIPEVVRSIVRKMNITNAAILYDDTFVMDHKYKSLLQNIQTRHVITGIAKEGKREREEQIEKLRNLDINNFFILGNLMSIRMVLESVKPTYFERNFAWHAITQSEGEVSSQRDNATIMFLKPMSYAQNRDRFGRLKTTFNLNEEPQIMSAFYFDLALRTFLAIKDMLQSGAWPKNMEYIGCDEFQGGNTPERNIDLRTAFTMIQEPTSYGVFELVTQPGKSFNGYSYMKFEMDINVLQIRGGNSVNTKSIGTWTAGLDSPLVVKDEDVMKNLTADTVYRIFTVVQAPFIIKDEKAPKGYKGYCIDLINEIADIVHFDYTIQEVEDGKFGN

MDEKGEWNGIVKKLMDKQADIGLGSMHVMAEREIVIDFTVPYYDLVGITIMMQRPQVPSLFKFLTVLETNVWLCILAAYFFTSFLMWIFDRWSPYSYQNNREKYKDDDEKREFNLKECLWFCMTSLTPQGGGEAPKNLSGRLVAATWWLFGFIIIASYTANLAAFLTVSRLDTPVESLDDLAKQYKILYAPLNGSSAMTYFQRMANIEQRFYEIWKDLSLNDSLTPLERSKLAVWDYPVSDKYTKMWQAMQEAQLPATLEEAVERVRNSTSATGFAFLGDATDIRYLVMTNCDLQIVGEEFSRKPYAIAVQQGSHLKDQFNNAILTLLNKRQLEKFKEKWWKNDETQAKCDKPEDQSDGISIHNIGGVFIVIFVGIGMACITLVFEYWWYKYRKNPRIVDVIEANSGGKDGKTIDSVILGQAGKEYDKGGNTVLRPRFHQYPTTFKPRF

>BdorIR40a

MKVLLLWLIGWLPGALVQGAITYNADRNISDVAIALSEIINGLKPRQLAILAAPQFHFTKRHAPLPASAEIPDDSQLEGMQMDIDDFIYQLHKLNFKSVIYDKADLFFKFVEDSLQGSIESVNLIFSAPYELSARIQERRLSHRLSLFIFYWGAKHPPKAHEVRFEEPMRAVVITRPRKKAFRIYYNQAVPDGVSNLRLVNWYDGDNLGLQKVPLLPNAATVYSNFNGRVFRVPVFHSPPWFWVSYENDSTNSTMLDDYIDSTNDYNEFTEVNVTGGRDHCLLNLLAQHMNFQFVYIEAPGRTQGSLRNDDTGEENDTFTGGIGLLQNGLADFLLGDVSLSWERRKAVEFSFFTLADSGAFATHAPRHLNEAFAIIRPFKRDVWPYLILTVIFSGPIFYAIIAIPYKWHLPCQKRGARRRLRRQQQCDVERADELVFHMAYIKEITGDNEMTRRLLRQQQQQQRQGVVQQMRSGRVQGLAEIPNNLFDKCIWFTVQLFLKQSCKELYHGYRAKFLMIVYWIAATYVLADVYSAQLTSQFARPPHEAPINTLQRLQKAMLRDGYQLFVEKESSSLEMLENGTEVFRQLYALMKLQNPDMEGYLIDSVEAGILLIADGLENKAVLGGRETLYFNIQQFGSKTFQLSHKLYTRYSAVAVQIGCPFLDSLNDVIIHLFEGGILDKMTNAEYATQSRMLGKEYNALHPTNPSETNGNNEPPPSDDNRNANGGGDINGKGEENTEATPKSLDSQIIQPLNLRMLQGAFIVLICGYAAATGILVLELCCHRLNSNFMERTQARLLRRYRWCSRKIRRMTHMLFVRIMR

>BdorIR1

MAVPKGATTARRQTFAAALLCRRTSLITTLILLLSKWNLYVSAVANFYEFDAFADVLKQQHLHHAIIAYNGDTEQTQQQAGLLKDNALRALLNVASLQFYDVHQAESAKNATDFQRLFYHDSPRVGIYVAQLEDVLLQQYVLGSNVISVDTIDAGGYRVRVDVGSRFNSSRVWFIMSKQRTVTAALANVRRVMTPLPLNISADITIGVRLDDNNTIQLFDIYKIQKDWLDIEPKGYWSTAEGLKLNLRFHQTFVNRRRNFKGLQLVGGIVIREQPADMADLDYLNSLYHKNFDPMQRKTYQLVKLMEPVFDVSFQPALRKTWGEQAPNGSWDGVMKLLLSGEAEFSLCPMRFVPNRVHLIHYTIAVHTEFVFFIFRHPHRNDIRNIFFEPFVEEVWYTVIAIVALTTLLLQLHLHHENRFFINKDPHFQTRFDYAIFSILEAFFQQGPSTDAFTATSTRTLIFSVCLFSLLLQQFYGAYIVGSLLSVSPRTITNLEALYNSSLDIGIENIPYNIDTFEKTTVPLGMAIYKERVCKNRERNILYIAEGAERIKKGGFAFHVSANRMYYILKELLTEKEFCDLQDVPFIPPYRIGIGITKSSPFREYFTTTIAKFHTTGLLQHNDNQWQLPQMDCSLSQNYEVEVDLQHFLPALLFLVSAMLLSLAVLILEIIYYNLEKSTKLARLCPRIMPKPKLEFIN

>BdorIR75a

MWFSTSVATVIGTFITISIFIVQSDYHAGAGFIITGFVTNLNMAMNVIIFACNKNNLLQITKKMLRYGLLIEIIDINGKYKLDSAFTRANYRKSSVLTDCICVNTNKLLNQASARRYFNKTYQWFLWYKEGKQVESLISPGIDYLGPNAQITLINGSNSMIQIWNVYSVGKHLRNPLKLSLINLSPIKESSYKHISKNIMQLQYSRKRNQFHGAILRGATVIDVNDVISNIKIFSLLSDPGKQSGISAFTKYYYELVQILKDHINFRIEFRVARGWAGKLANTSYRLGFLGIMARNEADVGASGIFNRLNRFSDFDIIHQGWKFETAFIYRAYASELSFQIKGDNFLIPLKRDVWLAIIWLFAVISLVYWLLSNVNLKLQLKNNKQLLDARCLQHVQTELTQEEHKRTFGEYTNDYYSSDLRDTLPVSNIFLIIIAAICQQSIISLSRSSAIRILYFVIFINTLLIYNYYTSSVVSGLLSSSLQGPANIDEIITSSLKVSFEDIGYYKVLFKESNSPVVARLIHKKLLSSRKYYDLPVFTNIKTALPFIKDGSYAFHCEV

VDAFAEIAKNFEAKELCSLRVIKGLMEMELMNGIVHKNSQYTEIFRFTMHWAREIGLVGRLNNRQPKRLICQSVYFVFPVNLSNMPGLYFILLGTILSLLTSLFEIVYHFYSIQK

>BdorIR21a

MPPSWTRFLVQAALLLCSLQWVCSELATAAEVDRVACVSPTLIKRHNLNPQLYGGCEYRKRNATVSQFRRLRREIKPIFRGHPKPRGEVLANKFHMNSFGADQTDSLVKLVNKIAIEYLYKCPPVIYYDSFVKKSEAMILESLFKTFPITFYHGEINEHYKAINSRLRRRIDSQCKSYILFLSDPEMTRKIIGPQIESRVVLVARSTQWKLRDFLSSEASSNIVNLLVIGESLTETPSRERPYVLYTHKLYTDGLGSNTPIVLTSWIRGALSRPHVNLFPTKFANGFAGHSFQVSAINQPPFIFRIQSLSRGGSSHTNWDGLEYRLLNMIASKLNFTIDIIEPARRTNVKSVIDNIMLQVRTKAADIGMCGLYITDDRITETDMSIGHSRDCASFITLASKALPKYRAIMGPFQWPVWVCIVVIYLGAIFPIVYSDRLTLRHLIGNWGEMENMFWYVFGMFTNSLTFSGKYSWTSTQKTSTRLLIGSYWLFTIIITACYTGSIIAFVTLPAFPNTVDSVNDLLGLFFRVGTLDNGGWETWFQNSTHVPTVKLYKKMEFVSNLEEGIGNVTQSFFWNYAFLGSAAQLEFMVQKNFSDDNISRRSALHLSEECFALFQVGFLFPRDSVYKRKIDSMILLAQQSGLMNKILNEVKWSMQRSASGKLLQASSANALRERIQEERQLTTADTEGMFLLMGIGYLLGAIALVSEIVGGITNKCRQIVRRSRKSISSAWSSKRNSEDGEGLRTAAEQLAHEQRKEVKRKAEKQGFGMREFNLTKKTLKELYGNYYKQEPTYVLKDGKLLLETEALSTSSADYHSRDSSGEVPANMLPHLHCKKKAMLVAEIDVERERERELMAAAAEESLAALDACLKLEQDTDSSERSEDDVAYEYELFGSLVEPEGPLSTKLDDLNLFTEGAAELEKVENEPVEARKS

>BdorIRNMDA1

MPVLNGINVVYLLFCGIHLGVIAQKHSQHSDNPSTYNIGGVLADPESESHFRTIISNLNFDQQYVPRKVTYYDKTIRMDKNPIKTVFNVCDKLIEKRVYAVVVSHEQTSGDLSPAAVSYTSGFYQIPVIGISSRDAAFSDKNIHVSFLRTVPPYYHQADVWLEIMFHFGYTKVIIIHSSDTDGRAILGRFQTTSQTNYDDIDVRATVEMIVEFEPKLDSFTEHLIDMKTAQSRVYLLYASTEDAQVIFRDAAINNMTEAGHAWIVTEQALHANNTPVGVLGLVLEHANSDKEHIRDSVYVLASAIKEMMSNETITEAPKDCGDSGVNWESGKRLFQYLKTRNITGKTGQVAFDDNGDRIYAGYDVINIHEKQKKHVVGKFYYDPEKAKMRLRINDSEILWPGKQKKKPEGIMIPTHLKILTIEEKPFVYTRRLTDDEVNCDEDEIPCPLFNATDGSENENCCRGYCIDLLNALSHRINFTFALALSPDGQFGHFTLKNVSSSSSGAITSRKEWSGLIGELVNERADMAMPLTINPERAEFIEFSKPFKYQGITILEKKPSRSSTLVSFLQPFSNTLWILVMVSVHVVALVLYLLDRFSPFGRFKLSHTDSNEEKALNLSSAVWFAWGVLLNSGIGEGTPRSFSARVLGMFWAGFAMIIVASYTANLAAFLVLERPKTKLSGINDARLRNTMENLTCATVKGSSVDMYFRRQVELSNMYRTMEANNYDTAEQAIQDVKKGKLMAFIWDSSRLEYEASKDCELVTAGELFGRSGYGIGLQKGSPWTDAVTLAILEFHESGFMEALDKHWIFHGNAQQCELFEKTPNTLGLQNMAGVFILVAAGVAGGVGLIIVEVIYKKHQVKKQKRLDIARHAADKWRGTIEKRKTLRASLAMQRQYNVGLNATPGTISFAVDKRRYPRMGPRAPEQAWKSDADILRNRRYLDDATKGGHSPAVHMPILGKMRPPTNMLPPRYSPAYTSNVSHLVV

>BdorIRkainateglr-3

MWLLQKLVILFTLGYVCANELKIAFWIDPLQADIELDVASTLKEIEALQLETKIQYYVVVITNVGKKKQEKNMEKLCEHLATDGVSVVIDFTYHIWREGLDLLRTYQIPFLRVDRMLAPYFKMFSEFVLQKSGHECIMIFQNARDTEEAIIQIVEGYPFRSLIMNAFDNKQDFIKRLRKIRPMPSCYAIFADGTAMNSIFDRISKANFFERPREWHFVYLDPRDRVFKFKKQVDYATKFTLNPKTLCRALRMKDTYCLSGFSFQRAMILEILRGLIELKQANLNWLQSFVMECNATSPIENGTAGLDILEQFPMSEFLDLTTDVTFPNDEFEHVPRLTYTPTISINLYSSEHDAVTELAIWQNDNLRKINETISPPRRFFRIGTVEAIPWNYMKRDPKTDELILDSFGNPIWEGFCIDSIQKLSERLNFGYMLVPPTSGEFGRRDVVNDVWDGIVGDLVTGETDFAVTALKMYSEREEVIDYIAPYFEQTGISIVMRKPVRQTSLFKFMTVLRVEVWFSIIAALVGSALMIWLLDKYSPYSYRNNRAAYQYPCREFTLRESFWFALTSFTPQGGGEAPKAVSGRIMVAAYWLFVVLMLATFTANLAAFLTVERMQTPVQSLEQLARQSRINYTVVEGSSTHQYFINMKFAEDTLYRMWKELTLNVTEDFQRYRIWDYPIKEQYGTILLAINGSEPVKNAKEGFRKVNEHENADFAFIHDSSEIKYELTRNCNLTEVGEVFAEQPYAIAIQQGSHFADELSYALLELQKDRFFEDLKAKYWNMSRIKACSVNEEQEGISLESLGGVFIATLFGLGLAMVTLVLEIIYYRRKYSTMQRFSEITKVKPASGTSIKQLLPKKKSKKRIAVWHTSTSKRDNSPEHKTPPPAFDAVKFRGKKVPPNITLGGQVFKPGRAGQRQLSESLDSAEYRNEGIPNRDDELPPYTE

>BlatIRkainate2

MARPCRQILLHFMTTTRCSCITLILLLALSCLQIAASQKTNVGLIYESDNPDMEKIFQIAIDKANEESGGTLELHGIAVAIEPGNAFETSKKLCKMLRQNLVAVFGPTTDLAAKHAMSICDAKELPFIDTRWDFAVQMPTVNLYPHASQLAVALKDLVVALEWTDTFTIIYETGEFLPTVNQLLEMYGTMGPTITVRRYELDLNGDYRNVLRRIKNSGDYSFVVVGSMATLPEFFKQAQQVGLMTDDYRYIVGNLDFQTMDLEPFQHGDTNITGIRLVSPDEKLVQDLAKTLYETEEPFQNVSCPLTTSMALVYDGVQLLAETFKHVMFRAVPLNCNDASSWDKGYTLVNYMKSLSLTGLTGEVKFDYEGLRTDFALDVIELTMSGMQKIGEWKTEDGFFANRPPPKIVEVDQRSLVNKSFVVITAISEPYGMLKETPAKLEGNDQFEGFGIELIEELGKKLGFTYTFRLQVDNKYGSFNPKTGKYDGMMLEIIEGRADMGITDLTMTSIREEGVDFTIPFMNLGIAILFRKPMKEPPKLFSFMSPFSGTVWMWLGIAYMSVSLTLFILGRISPTEWDNPYPCIEEPTELENQFSFPNCLWFSIGALLQQGSELAPKAYSTRTVASIWWFFTLILVSSYTANLAAFLTIESLSSPIENAEDLANNKGGVKYGAKVGGSTFTFFQDAKYPTYQKMYEFMRDHPEYMTSTNAEGVDRVENENYAFLMESTTIEYITERRCSLTQVGSLLDEKGYGIAMRKNWPYRDTLSQAVLELQEQGVLTKMKTKWWKEKRGGGACSDTSSEGGAVALELSNLGGVYLVLIVGSFFGVLVAFLEMVLGVKERSDENKVSFKTELIEEFRFVMQCSGNTRPVKYPKNSSRSRSRSSRSRSHSRSSSKSSILSVDSLPMDESKLHHISEHTKHVK

>BlatIR21a

MCSELVTGAEVDGIACVSATLIKRHNLNPQLYGGCEYRKRNATVPQFRRLRREIKPIFRGHPKPRGEILANKFHMNTFGADQTESLVKLVNKIAIEYLYKCPPVIYYDSFVKKSEAMILESLFKTFPISFYHGEINEHYKAINSRLRRRIDSQCKSYILFLSDPEMTRKIIGPQIESRVVLVARSTQWKLRDFLSSEASSNIVNLLVIGESLTETPSRERPYVLYTHKLYTDGLGSNTPIVLTSWIRGALSRPHVNLFPPKFANGFAGHSFQVSAINQPPFIFRIQSLSRGGSSHTNWDGLEYRLLNMIASKLNFTIDIIEPARRINVKSVIDNIILQVRTRAADIGMCGLYITDDRITETDMSIGHSRDCASFITLASKALPKYRAIMGPFQWPVWVCIVVIYLGAIFPIVYSDRLTLRHLIGNWGEMENMFWYVFGMFTNSLTFSGKYSWTSTQKTSTRLLIGSYWLFTIIITACYTGSIIAFVTLPAFPNTVDSVNDLLGLFFRVGTLDNGGWETWFQNSTHVPTVKLYKKMEFVSNLEEGIGNVTQSFFWNYAFLGSAAQLEFMVQKNFSDDNISRRSALHLSEECFALFQVGFLFPRDSVYKRKIDSMILLAQQSGLMNKILNEVKWSMQRSASGKLLQASSANALRERIQEERQLTTADTEGMFLLMGIGYLLGAIALVSEIVGGITNKCRQIVRRSRKSISSAWSSKRNSDDGEGLRTAAEQLAHEQRKQAKRKAEKQGFGMREFNLTKKTLKELYGNYYKQEPTYVLKDGKLLLETEALSTSSTDYHSRDSSGEVPANMLPHLHSRKKAMLVAEIDVERERERERELMAAAAEESLAALDACLKMEQDTNSSERSDDDVAYEYELFGSLVEPEGSLSTKLDDLNLFTEGAAELEKVDNEPVVARQS

>BlatIRkainate5

MSAMRFRASLWSLWLPLCLALLPQHVAANDFSSFLTANASLAVVVDQEYMQMRGENVLASFQKILSDVIRENLKNGGIEVKYYSWSQIRLKKDFLAAMTVADCKSTWQLFDSTQQNSILLIAITDANCPRLPLNRAIMIPIVDEGQELSQIILDIKVQRLLRWKTAAVLLDQTILHDNPTLVESVVHESAKNHITPFSLLLYQIDDTLRSQKKRSAIRQMLSAFQDGEQTPRQFIVLSQFYEDVVEIAASMKLFHVYNQWVFFVLSEELRNHDPISVTQNLDEGANIAFALNTTEPTCSSSINCTITELSLALVTSISRMIVEEQSIYGEISDEEWEAIRYTKQEKQDEMLGYMKEYLREYSKCTSCSHWKIETALTWGKSEEHRRYQSNSELRDTHNKNFEFIDVGYWTPTLGFNTHEVMFPHITHFFRNITLDILTVHSPPWQILERNSRGDIVRHSGISMEILKEMSRMLNFSYILHEVKVNAADSAAEDMQHTANVTDDLFGSLTFNIPYQVIETMQASRYFMAALAATIDEPDKKSFNYTVPISVQMYTFISRQPDEVSRIYLFAAPFTTEIWGCLVVIIIITAPVLYFINRLVPMDHLRITGLSTLNSCFWYIYGALLQQGGMYLPKADSGRLVIGVWWIVVIVLVTTYSGNLVAFLTFPQFQPGIDYFFQIFSSSAMQQFGLRNGSYFEKYATQITTRDDFRDYVQRAIIYNNVQGEDIGAVQDGKRINVDWRINLQLIIQKQFEKDKECKFSLGRDNFVAEQIGLIVPRDSPYLQLINDKITRMFQMGFIERWHQINLPSMDKCSGHGGMRQIMNHKVNLDDMQGCFMVLLFGFFIALFILFIEYWYRWYFVEKKRGVFST

>BlatIR25a

MPSAYLKFYNNIVTFLNILSLVSLATGQTNQNINVFFINDADNEPAAKAVTVVSTYLKKNPSYGISIQIDQVEANKTDAKTLLESICSKYAESIDRKQPPHVVFDTTKSGISSETVKSFTQALGLPTISASYGQEGDLRQWRDMDESKQKYLLQVMPPADLIPEVVRSIVRKMNITNAAILYDDTYVMDHKYKSLLQNIQTRHVITGIAKEGKREREEQIEKLRNLDINNFFILGNLMSIRMVLESVKPTYFERNFAWHAITQSEGEVSSQRDNATIMFLKPMSYAQNRDRFGRLKTTFNLNEEPQIMSAFYFDLALRTFLAIKDMLQSGAWPKNMQYIGCDEFQGGNTPERNIDLRTAFTMVQEPTSYGMFELVTQPGKSFNGYSYMKFEMDINVLQIRGGNSVNTKSIGTWTAGLDSPLVVKDEDVMKNLTADTVYRIFTVVQAPFIIKDEKAPKGYKGYCIDLINEIADIVHFDYTIQEVEDGKFGNMDEKGEWNGIVKKLMDKQADIGLGSMHVMAEREIVIDFTVPYYDLVGITIMMQRPQVPSSLFKFLTVLETNVWLCILAAYFFTSFLMWIFDRWSPYSYQNNREKYKDDDEKREFNLKECLWFCMTSLTPQGGGEAPKNLSGRLVAATWWLFGFIIIASYTANLAAFLTVSRLDTPVESLDDLAKQYKILYAPLNGSSAMTYFQRMANIEQRFYEIWKDLSLNDSLTPLERSKLAVWDYPVSDKYTKMWQAMQEAQLPATLEEAVERVRNSTSATGFAFLGDATDIRYLVMTNCDLQIVGEEFSRKPYAIAVQQGSHLKDQFNNAILTLLNKRQLEKFKEKWWKNDETQAKCDKPEDQSDGISIHNIGGVFIVIFVGIGMACITLVFEYWWYKYRKNPRIVDVIEANSGGKDGKTIDSVIL

GQTGKEYDKGGNTVLRPRFHQYPTTFKPRF

>BlatIRdelta-1

MTGFDLILSAALCLTCANLTDIRLPEGLIELEENNTVVAISPDLAVDEPSLDDAPLETVKTIIAKKEKMDKLREWIKGRKLVIATLEDYPLSYTVMENDTRVGKGVAFELIDFLQEQMQFTYEVVVPEDNIIGSREDYEKSLIKMLNNSEADLAAAFIPTLSEQHSFVFYSTTTLDEGEWIMVMQRPRESATGSGLMAPFDFWVWILIFISLLAVGPIIYMLIILRNRLTGDKEQKPYSLGHCAWFVYGALMKQGSTLSPIADSTRLLFATWWIFITILTSFYTANLTAFLTLSKFTLPYNTVSDILYKNKHFVSARGGGVEYAIRNTNESLSMLTNMIRNNHAVFSSSSNDTFNLQNFVEKDGYVFVRDRPAINHVLYADYRYRKTISMNDEKLHCPFAMAKEPFLKKNRSFAYPLGSNLSELFDPKLLNLVESGIIKYLSTKDLPNAEICPQNLAGTERQLRNTDLMMTYYIMFAGFVTAMVVFFTEL

IFRYLNQRNEGSKWARHGIGRTTNGLSVRAPRWLRQLETDSDKQRLTASPSGSTITPPPPYQSIFSSNHRHQHQQDEASHLSKESSLHRWRRAGQFGAGGSNFGTLAVGTGSGAGVLLGNGQLHEGSGAGGVRRLINGRDYMVFRNPNGQSQLVPVRAPSAALFQYTYTE

>BlatIRNMDA2B

MIRKMKGLSFCQKANKRRKNVETNDQTPRGNTALPTRAACTNHNNNKVNKLKICKTTTITQTHNVNAEHQLTTPLICRNNNKKNSCNSVGSQRRYCSNGSNNSTTPPPTFNQHHNHQHQHCLAPGQLTYKMPLPLLSICKTTTTTTAVDVSADQQQLQQQRQQQHEQLERVQNCCLARCKEQQRQRKATTTYAPTKLTQTHRQHLSQQPATTHALSCFSKSLETLSLRALLSRHHQLFHFQQHLQQLHLHLHFHFGIAVIVLASILMYSCPCASALRLTNGGNTKTLSANKEQLNIGLIAPHTNFGKREYLRAINTAVQGLAKTRGAKLTFLKDYSFEPRNIHFDMMSLTPSPTAILSTLCKEFLQANVSAILYMMNNEQFGHSTASAQYFLQLAGYLGIPVISWNADNSGLERRASQSTLQLQLAPSIEHQSAAMLSILERYKWHQFSVVTSQIAGHDDFVQAVRERVAEMQDHFKFTILNSIVVTRTSDLMELVNSEARVMLLYATQSEAVTILRAAEEMKLTGENYVWVVSQSVIEKKDAQTQFPIGMLGVHFDTSSAALMNEISNAIKIYAFGVEAYLTDPANRGRRLTTQSLSCEDEGRGRWDNGEIFFRYLRNVSIEGDLNKPNIEFTADGDLKSAELKIMNLRPGANNKNLVWEEIGVWKSWETQKLDIRDIAWPGNSHAPPQGVPEKFHLKITFLEEAPYINLSPADPISGKCLMDRGVLCRVAADHEMAADIDVGQAHRNESFYQCCSGFCIDLLEKFAEELGFTYELVRVEDGKWGTLENGKWNGLIADLVNRKTDMVLTSLMINTEREAVVDFSEPFMETGIAIVVAKRTGIISPTAFLEPFDTASWMLVGIVAIHAATFMIFLFEWLSPSGYNMKLYLQNASVTPYRFSLCRTYWLVWAVLFQAAVHVDSPRGFTSRFMTNVWALFAVVFLAIYTANLAAFMITREEFHEFSGLNDSRLVHPYSHKPSFKFGTIPYSHTDSTIHKYFKDMHHYMRQYNKTSVAEGVAAVLNGNLDSFIYDGTVLDYLVAQDEDCRLMTVGSWYAMTGYGLAFSRNSKYVQMFNKRLLEFRANGDLERLRRYWMTGTCRPGKQEHKSSDPLALEQFLSAFLLLMAGILFAALLLLLEHIYFKYVRKRLAKKDGCHCCALLSLSMGKALTFRGAVFEATEILKKHRCNDPICDTHLWKVKHELDMSRLRVRQLEKALDQHGIKPPQLRLTSSTDMLNHHHLKERPPLLGNLSLAASAQDLYRSYKTEIAEMETVL

>BlatIRkainate1

MRFSNIFVPLLLLIAVKLTHEYTNNGDIEVKVGVLFFRDEYEIELSFDEAFLEINNNKLFGLSFQLIKRF

VPSDDSFILQQLTCELISDGVVAIFGPSSKTSTDIVAVIANATGIPHMKFDWRIESPTQDRLNNRMTVNVAPSVAMISRAYYSIIKYNYEWQQFTLVYETKIGLARLQDLMNINPLNSEQIKIRYIEDYKSDLRVLWKEISETFHENKVILDCEADSLRDLVLTAKEFKMLGSFKYLFLSHLNTHNSPLQSMYDSGYRANITSVRLKLVDENPFRRKKTRLRPIDEIFQNQILLPVLMYDAVVLFANAARNVITKVRSYVEPQRRCEFEYGRPWYIGRQIVREMKSISEDDVEPPFKTENLKIDENGQRTQFNLEIYKPTTNEILAVWKSDGTVSPPTSIQFGTGSSSNTPDFSLERKKFIVTTRFEAPYFMLKEDYENLRGMERYEGYAVDLIQKLSDIMGFEYEFLIESRTGKLNPDTGEWDGMIRRLIDHQAQIAISDITITQARRQVVDFTVPFMQLGISILYYKRPPEAKNEFAFLEPFAEEVWYYLMLTQLIMTLLFVILARFSHHEWTNPNPADSDPEELENIWNNSNSFWLMIGSIMQQGCDILPKGPPMRILSSMWWFFTLMMVNAYIANLAASLTNNKLPSEFDSLESLIDQDKIKYGTLAGGSTSVFFSESNETEYKRAWNQMISFKPSAFTSSNKEGVDRVRKGNGSYAFLMETTTLSYNIERDCHLKQVGSQFSEKHYALAVPLGAEYRSNLSVSLLQLSEKGELFNLKHRWWTPTVKPECPSEDTTDGDELSIIELSGVFLVLGAGVVVSFIIGCCEFLWNVQTVAVNEKTTPWQAFKAELCFVLKFWITKKPAIISESTKSSISSNSSSKRSYDRSHSRSHSRVSGRSRHTKSKTRERERERSVVSRRYYD

>BlatIR75d

QLPALTYKSGILLLEFASACSLNALRWAAAAEHNYFTTNRFWLLFTDEPTHISLLDDEDIFLPPDGEVRVMLWQPGMQFFTLVDVYKVAADKPLRRTLVGGRELRDAEDMLQALGKFGSAISYRQNLEGITFKTGLVIAFPDLFTNIEDLSLRHIDTISKVNNRLTLELANKLNLRFNTHQVDNYGWHKPNGSFDGLMGRFQRYELDFAQMAIFMRLDRIAIVDFVAETYRIRAGIMFRQPPLSAVANIFAMPFASDVWIAILLLMIFTICIFIVELVYSPHLHEMDILDCVVFVWGAMCQQGFYANLLNRSARVIIFTTFVSTLFLYTSFSANIVALLQSPSEAIQTLSDLTQSPLEVGVQDTQYNKIYFNESTDPVTNHLYHKKIAPKGENIFMRPAIGMKKMRTGLFAYQVELQAGYQIISNTFSEPEKCGLKELEPFQLPMIAVPTRKNFPYKELFRRQLRWQREVGLMNREELKWFPQKPKCEGGMGGFVSIGITECRYALGIFGFGLLLSAFSFILELIVNYVWNLAKKIHRIKKQRKESNTADGYRGNFVH

>BlatIR76a

MSLLSPAVNHWTALLNFIIQTYFIDSHATCILWHHDFPFELQTPANGEFIQYINIWPDNLSQSLQQDIYNFTAFAETQLAYGMHPDALVQKLTIAIRESHCETFVAFQEDIPSFARSFYNASRISVWRSLRNKFLFVYRKDLQQDTTTYFDDFLFVDQPNVLIVEAECGNCSTFALKTNKFIGPLAEHPEQLYVLDHYNGVDGKFELGVDLYMDKVHNLQGREVTVGIFDYRPFTVVDYERQPQIKDRSPENLRGMTHIDGTEVRMLLALCEVVNCTVNTDTSEDDWGISYANLTADGIFGLVTSRKAQYVVGALYFWPDDYRYLDMSSFIGRSGVTCLVPSPHRLTSWLLPLRPFQLTLWLGVFASLGLETLALFFTRHLAPSDTEPRYGLMESFQFGYITTLKLFVSQGSDYVVNSHTVRMVLFACYMMDTIVTSVYGGGLSAILTLPTLEEASDSVERLYRHGIPWTATSPDWVISLRGADDDPMVEKLLQKYHVYTYEQLTEFAKTENMGFILERLAFGHFGNVDFLTDESFKRLKLMIDDIYFQYCFAFVPRLWALLPKLNDVIMQVHSTGLDIFWEWEVAATYMDGQQQEEIQASMYMDFDVGPVKLDMGNFIGLVLPLIIGLVFSIFAFIGELAYYKYTQKEAQAVINVN

>BlatIR8a

MWLMQKLVILVSFGYVCTNELKIAFWIDPLQADIELDVASTVKEIEALKLETKVQYYVVVITNVGRKKEEKNMEKLCEHLATDGVSVVIDFTYHIWREGLDLLRAYHIPFLRVDRILAPYFKMFSQFVLQKAGHECVMIFQNARDTEEAIIQIVEGYPFRSLIMNAFDTKQDFIKRLRKVRPMPSCYAIFADGTAMNSIFDRISKANIFERPREWHFVYLDPRDRVFKFKKQVDYATKFTINPKTLCRTLRMKDTYCLSGFSFQRAMILEILRGLIELKQANLFWLQSFVMECNATTPIENGTAGFDILEQLPMSEFLDFTTDATFPDDEFDHVPRLTYTPTININLYSSEHDAASELAIWQNDNLRKINETISPPRRFFRIGTVEAIPWNYMKRDPKTDELILDSFGNPIWEGFCIDSIQKLSERLNFGYMLVPPTSGEFGRRDVVNDVWDGIVGDLVTGETDFAVTALKMYSEREEVIDYIAPYFEQT

GISIVMRKPVRQTSLFKFMTVLRVEVWFSIIAALVGSALMIWLLDKYSPYSYRNNRAAYPYPCREFTLRESFWFALTSFTPQGGGEAPKAVSGRIMVAAYWLFVVLMLATFTANLAAFLTVERMQTPVQSLEQLARQSRINYTVVEGSGTHQYFINMKFAEDTLYRMWKELTLNVTEDFQRYRIWDYPIKEQYGTILLAINGSEPVKDAREGFRKVNEHENADFAFIHDSSEIKYELTRNCNLTEVGEVFAEQPYAIAIQQGSHFADELSYALLELQKDRFFEDLKAKYWNMSRIKACSVNEEQEGISLESLGGVFIATLFGLGLAMVTLVLEIIYYRRKYNTMQRFSEITKVKPASGTSIKQLLPKKKSKKRIAVWHTSTSKRDNTPEHKTPPPAFDAVKFRGKKVPPSITLGGQQFKPRRAGQRQLSESLDSAEYRNEGIPNRDDELPPYTE

>AludIR75a-4

ILIHFLGCNVNYTLVNRWTINETHGGLIGALAVQSADLISTPFIPTAPRMEFFTVIAETSSFRSICLFRTPRNSGIQGDVFLKPFNTTVWLLFGLLLLLTAAVLWSIFRLERKRMHQRYVDYMPSFLATFLISFGSACSQGSDLVPGSIGGRMVFFTLYLLTFLMYNYYTSIVVSSLLGSPVKSDIKTMGQLADSSLEVGLEPLPFTLTYLNNSLLPEVRRFKHKIDSTPNPQAIWMPLKKGILRVRDEPGFVFGFEASTGFLLVKRYYKPYEICDLNEILFRPEKSLFSAIHKNSSYKEIAKQRVYRILETGVSLKLHRYWVQTTLECFDSNF

>AludIR75a-2

MQPVLFSFILNHFLNANINAVVFFNCWSVQAQRHLSHLTSQRLWYTRFVNIESIDLSVDFEYQYLLHNRQILGAFLDVNCNKSEEVMNTLSRWHLYKEHYNWLLYDRSADMHKLQRLFTNANLSVDAQLTYATLRLPLHDTPPHTNLSFYTTYDVYNNGRLLGGKLNVTIDREYQCNWKVCHVSRYLSELHLRHKYGNRDKLHDITMRVSTVITNRPLSWPVPLLLAYLSSENNTEIDPISRFGYQVLLVFKDSFGCNMNLTFYDRWSTNETHGGTVGDIVTQAADFISTPVLATASRLKHLSLIAETGAFRSLCLFRTPRSGSMRGDAFLQPFNSSVWLVFSILLAILAIFLWRAFAVEMRNFRRRLPYEPSLLATFLLAFGSACYQGSNIVPFSMGGRMAYFTLYFATFIMYNYYTSILLSTLLGTPVKSDIRTLGQLADSSLEVGLQPLPYTYVYLNVSQLPEVRRFVHRKIEAKRHPGNVWMPMEEGVLRVRDEPGFVFVLETSQAYSFLERNFLPHEICDLNEVMLRPDKSLYT

>AludIR75a-1

MIDCTCTNADYLLRQASLGHYFNKTYQWFLWSYENKNAEEIVPSDINYLGPNSQVTCINGTNSTIWNVYSNGRHLQSPLKFSLISNSVSVPPFTKLPDINIGKLILKAQHARTRNQFGGLKMRAVTVIDLDNITSNTQITGLLSENVKHIGISAFTKYYFELVQILKEHINFSIEFRVARGWAGRLDNTSFRLGFLGIMARNEADVGVSGIFNRISRFAEFDIIHQGWKFETAFLYRFVPELSNNIKAGSFLVPFQRPVWILIAWLIITTSLVFWLIDNIKIRHENQKQLLDNKEQTSLANILLISIAAICQQSVNPTSKWLSIRILFLSVFVFSLLIYNYYTSSVVSGLLSSSAQGPTNIEEIICSPLKVSFEDIGYYKVLFRESKSATVKRLIRQKLLPSRKELDDLPVYTHIQKAISLIKKGSYAFHCELADAFPEIAKNFDANELCTLRVVKGLMDIELMNGIVPKNSQYTEIFRFTLIWFRETGLIGRVLYKRLPKKTNMSSSVYRFSSNFFKCTRPVFFASWWDFIICVNRIVRDLNSCVLYSRVIQFLWFVSILHFCLT

>AludIR75d

MQMLNFHRLSILLIYNYTILVAAFNAATAIELVNATRAGNLTAEVRSAADATSTYDARIFMEYFRWHGVHNIILVVCPEDVGTRAEHHKLQSILHEFIANGFSTRIFNGHDYDGSVDSPNDKQIQMKPQQLNATTTFPVDASKDPAAVTCATFGPPRTFRSDNDTRRPLRLHLPPFTYKSGILMLQFASACALNVLRWSAASEHNYFTTNRFWLLLTDEPTNISLLDDKDIFLPPDGEVRIIVRQPKAHFYTMVDVYKVSAEKPLRRTSMGVPQMQGVNDLLQALRKFGSAISYRQNLEGITFKTGLVIAFPDLFTNIEDLSLRHIDTISKVNNRLTIELANKLNMHFNTHQADNYGWHQPNGSFDGLMGRFQRYELDFAQMAIFMRLDRIALVDFVAETFRIRAGIMFRQPPLSAVANIFAMPFARDVWISILLLMIFTSGIFIVELKYSPHSHEIDILDCVVFVWGAMCQQGFYANILNRSARFIVFTTFVSTLFLYTSFSANIVALLQSPSEAIQTLSDLAQSPLEIGVQDTVYNKIYFNESTDPITNPLYHKKIAPKGDNIFMRPTVGMEKMRTGLFAYQVELQAGYQIISNTFSEPEKCGLKELEPFQLPMIAIPTRKNFPYKELFRRQIRWQREVGLMNREELKWFPQKPKCESGVGGFVSIGITECRYALGIFGFGLLLSALSFILEIAVSCAWALVKKICRNKKQ

>AludIR8a

MWLMQKLAFIVLFGYVGATELKIVFWIDPLQAGIDVDVASTVKIEVDVASAVSEIQALNLDTQIQYSVVVIEEEEKNKAQNMDKVCERLAMDGVSVVIDFTYHIWRDGLDLLRTYQIPFLRVERMLTPYLKMFSQFIMQKSGHECIMIFENSRDTEEAIIQIIEGYHFRTLVLNAFDERDFVQRVRQLRPTPSCYALFAGGTTMNTIFEKISKGKLFERPREWHFVYLDTRDRVFKFKKSVEYATKFTINPKTLCRAVRMKDTYCLSGFTFQRAMILEILRGLIEIKQTNMYWLQSFVMECNGTSPNLETPTGFDILEQFPTSDFLHFTTDVSFPKDEFDHVPRLTYSPTININFYSSEHEAVTDLAIWENDELRKINGTISPPMRFFRIGTVESIPWNYLKRDPHTDELVLDAYGNTIWEGFCIDFIKTLSERLNFGYVLVPPTTGEFGRYDATNDRWDGIVGDLVTGETDFAVTALKMYSEREGVIDYIAPYFEQTGISIVMRKPVRQTSLFKFMTVLRVEVWFSIIAALVGSAIMIWLLDKYSPYSYRNNRAAYPYPCREFTLRESFWFALTSFTPQGGGEAPKAISGRIMVAAYWLFVVLMLATFTANLAAFLTVERMQAPVQSLEQLARQSRINYTVVEGSSTHQYFINMKFAEDTLYRMWKELTLNVSEDYQKYRIWDYPIKEQYGTILLAINGSEPVKDAKEGFRKVNEHENADFAFIHDSSEIKYELTRNCNLTEVGEVFAEQPYAIAIQQGSHFADELSYALLELQKDRFFEELKAKYWNTSRIKACSVNEEQEGISLESLGGVFIATLFGLGLAMVTLVLEIFYYRRKYSAMRRFNEITKVKPASTSSLKQLVQKKKPKKRIAIWHTAKRDNSAEHTTPPPAFDAIKFRGKKVPHSITLGGEEFKPRKGGLRRLSDSLDSVNYATKEGIPANRDDELPPYTE

>AludIR76a

MLLLSPAVNYWTTLLNFIIQKYFIDSHATCILWHRDFPFELQTPADSEFIQYINIWPDNLSQSLQQDIYNFTAFNEIQRDYGMQLDALVQKLTIAIRESHCESFIAFQQDIPVFTRSFYNASRISVWRSLRNKFLFVYRKDLQQDTGTAAYFDDFLFIDQPNILIVEAECGNCSMLALKTNKFIGPLAEHPEQLYVLDHYNALSDKFELGVDLYMDKIKDLQGREVTAGVFDYRPFSVLDFNREPQVKDNNPENLRGTVHLDGTEVRMLFALCEVINCTVQADSSDKTDWGTSYANLTGDGLFGLITERKSEYIVGALYFWPDDYRYLDMSHFIGRSGVTCLVPAPQRLTSWLLPFRPFQFTLWMGVFASLLLETLALFFTRRLAPSDNEPHYSLLESFQFGYITTLKLFVSQGSDYVVNSNTVRTVLFACYMMDTIVTSVYGGGLSAILTLPTMEEASDSVERLYRHGIPWTATSPDWVISFRGEGDDIDPMVEKLLSNYHVYTYEQLTEMAKTENMGFILERLAFGHFGNVGFLTDESFKRLKLMVDDIYFQYCFVFVPRLWALLSKLNDVILEVHSTGLDIFWEWEVGAHYMDGQQQEEIQASMYMDFDVGPVKLDMDNFVGLVLPLLIGLILSAFAFIGELIYFKYTTQKKEKNTEIEERRISELKIA

>AludIR92a

MLPPHTHVTTLLQLLIQKYFAQFSSVLIIHGGQTVGGSSTLQQEYLEAVQLAFRNLSQQGRVIGLQWIDVSQLDKNSNCNEGATSSDSPSSSNSNSNTLCDDSFSYDDELQLHLLRAVDIITEGFITILTDTVRFVHARYYATRNAELRLKDKFYLFLCEHENPQDLLSTEILQFYPHHLMVIPETQNKTKKSHESTVTNIPNTNATRARSKRYPSIQITPLYTHHDLNFQLWTQKFVGTNGNLDAMLLDEFLPNGTFSQNAELFPNKVSNMRGRTLRVGSITYIPYVVTNYVPDGTGEVDALDSSAFSRTVSFLGSEAELMKSFCDSRNCRLSVENYGGYNWGYIYENESSEGMMGGVYTQNVEVAIGCVYNWYNNITETSFTIARSAAAILGPAPAQFPAWRACIMPFSGELWLFLIMAMVLCAIVMYFIQFTAFGLENWRWHQTRKFHHTTRLGQALLDMFAVFIQQPSGPTSLRTFAARLFLATILCATITLENTYSGQLKSLLTIPLFTEAMDTLRKWSTTDWTWGAPSIVWVQSIEGSDIVLEQIMTKKFEVQSYGFLYNASFRSDYGVGIERLMSGSFAFGDYITPPAMETKIVIKDDLFFDWTRAVSIRGWPLMPLFDKHIRACIETGLFVHWERESVAKYLDRQSQEILLDLASGNIKKLPPQNLTLENISGAVFALLFGCLIATKVFILELIIYHLNKLKLLDIYRKR

>AludIR40a

MKVFILCLIVWPSRLVYGAITYNADRNISDVAIALSEIINGLKPRQLAILAAPQFHFATRHAPLPATAEIPTDSQLEGMQMDIDDFIYQLHKLNFKSVIYDKADLFFKFVEDSLLGSIESVNLIFSAPYELSARIQERKLSHRLSLFIFYWGAKHPPKANEVRFEEPMRAVVITRPRKKAFRIYYNQAVPDGVSNLRLVNWYDGDNLGLQKVPLLPNAATVYSNFKGRVFRVPVFHSPPWFWVNYENDSINSTMFEDYVDNDYAEFTEVNVTGGRDHCLLNLLAQHMNFQFVYIEAPGRTQGSLRSDDTGVENDSFTGGIGLLQNGLADFLLGDVSLSWERRKAVEFSFFTLADSGAFATHAPRRLNEAFAIIRPFKRDVWPYLILTVIFSGPIFYAIIAIPYKWHLPCQTLQANRQPRGRLQQRDVERDGELVFHMAYIKEITGDNELTRRLLRQQQQQLYKEQERRGRVPALTEIPHNLFDKCIWFTVQLFLKQSCKELYHGYRAKFLMIVYWIAATYVLADVYSAQLTSQFARPPHEAPINTLQRLQTAMLHDGYQLFVEKESSSLEMLENGTEIFRQLYALMKQQNPDMEGYLIDSVESGIMLIADGLENKAVLGGRETLYFNIQQFGSKTFQLSHKLYTRYSAVAVQIGCPFLDSLNDVIIHLFEGGILDKMTNAEYATQSRMLGKEYNNLHPAESEANGNNEPPPNDDNRNSNGGSDANNKGDESAEAASKSQDSQIIQPLNLRMLQGAFIVLLCGYAAATGILVLELCCHRLNSNLMERTEARMVRRYHWCCRKFRKITRTLYARILR

>AludIRdelta1

MDIILGDLGRGGIEKTGNYTLVLAIAWTILNVYGDFPSMPLVISQYASESQTYVHQAELIDALMTYLDQRQYRIYIMEQEKLFGHEERSEWGDFCEAAIWFIDSWNSFKTLATDLEDPKSSYKRTGWFLIIYTGTERERLDTVKKIFSRLFEIFVINVNVFLLIDTTPFVYTYFPFSPKKCHSAQPELLLSFRNRKAKQKFKDYRKFFPFKVGNLYGCELAIITWHHPPFMILKEDPKTGQIVSIEGMEGMLISILSEVMNFAIRIVIPQPKERGAVYSNGTLTGVAKMIAEGDGNITIIFNMYEKKRAQVMGASFSYMSFPLTVATPHGRPLSPLQRLLRPFKHIIWSLIGSNIILAVLIIYALKLLGSKQIVSFVFGKTNRIPFSNLWASLYGGVIHNRLPYRNFSRYLLGLWLLCTLVLRSAYTGQLFIILQDGRALTPLKSFQEIVDKNYIIYTADVLAELLDSTIENAILGFVDGGNNSMPLILKRIARGSKEVLTIIEPALHYYNYEQTTDAERVAILPQKLIMTPLTMYMRKHSYLITPINLHLYNFLDLGIINKFEKRYRLPKEAEESDEPVRLSLFLLLGIFSIYLALMGLCTLIFLLELWTTRSPLTKMVIDFFNY

>AludIR1

QHFTNETPSTIIGTTGADSKVADLLAQKMNFTMDLQWPDNDFFGARTKNGSYSGAVGRIVRFETDIILTGFFIKDYLTRKLAFSAPVYMDELCCYVKKASRIPQSILPLFAVNFDIWVSFIIVGLLSPFIWILLRHVNLGAIESNSLISQLPTLQPQERRQLTQKHILQYMRIYLDTWVMWVRVNIVCYPPFISERIFIVSLCLVSVIFGALFESSLATVYIRPLYYKDINTMKELDEANIRIYIKHAAMRDDLFYGHSSYIYQNLEKKLLLVAELEERLIDIMARGGKLAAVTRASSLELDDIYYFIKKKIHKIPECPKNYHIAFIFPNNSPLEKSINILLLKIVHAGLIDHWIGDMKYKAKIRSRNFAEFQEEIGDKWKVLTLNDLQLAFY

>ZtauIR25a

MPSAHLKFRKEILIFLKIISLASLATGQTNQNINVFFINDADNEPAAKAVTVVSTYLKKNPSYGISIQIDQVEANKTDAKTLLEAICSKYAESIERKQPPHVIFDTTKSGISSETVKSFTKALGLPTISASYGQEGDLRQWRDMDESKQKYLLQIMPPADLIPEVVRSIVRKMNITNAAILYDDTFVMDHKYKSLLQNIQTRHVITGIAKEGKREREEQIEKLRNLDINNFFILGNLMSIRMVLESVKPSYFERNFAWHAITQSEGEVSSQRDNATIMFLKPMSYAQNRDRFGRLKTTFNLNEEPQITSAFYFDLALRTFLAIKDMLQSGAWPKNMEYIGCDEFLGGNTPERNIDLRTAFTMVQEPTSYGVFELVTQPGKAFNGYSYMKFEMDINVLQIRGGNSVNTKSIGTWTAGLDSPLVVKDEEVMKNLTADTVYRIFTVVQAPFIIKDEKAPKGYKGYCIDLINEIADIVHFDYTIQEVEDGKFGNMDEKGEWNGIVKKLMDKQADIGLGSMHVMAEREIVIDFTVPYYDLVGITIMMQRPQVPSSLFKFLTVLETNVWLCILAAYFFTSFLMWIFDRWSPYSYQNNREKYKDDDEKREFNLKECLWFCMTSLTPQGGGEAPKNLSGRLVAATWWLFGFIIIASYTANLAAFLTVSRLDTPVESLDDLAKQYKILYAPLNGSSAMTYFQRMANIEQRFYEIWKDLSLNDSLTPLERSKLAVWDYPVSDKYTKMWQAMQEAQLPATLEEAVERVRNSTSATGFAFLGDATDIRYLVMTNCDLQIVGEEFSRKPYAIAVQQGSHLKDQFNNAILTLLNKRQLEKLKEKWWKNDETQAKCDKPEDQSDGISIHNIGGVFIVIFVGIGMACITLVFEYWWYKYRKNPRIVDVIEANSGGKDGKTIDSVIL

GQAGKEYDKGGNTVLRPRFHQYPTTFKPRF

>ZtauIR8a

MWLLQKLVILVAFDYVCANDLKIAFWIDPLQSGIELDVASAVKEIKALQLETEIQYYVVTIENTGRQKEEKNMEKLCEHLATDGVSVVIDFTYHIWHNGLDLLRTYQIPFLRVDRILAPYLKMFSEFVLAKSGHECIMIFQNARDTEEAVIQVVEGYPFRTLIMNAFDKQDFIQRLRKIRPMPSCYAIFADGTAMNSIFDRISKANVFERPREWHFIYLDPRDRVFKFKKQVDFATKFTINPKTLCRSLRMKDAYCLSGFSLQRAIILEILRGLIELKQTNLYWLQTFVMECNYTSPNENGTNGFDILEQFPLNEFLYFTTDVRFPNDEFDHVPRLTYSPTININLYSSEHDAVTELAIWQNDNLRKINETISKPRRFFRIGTVEAIPWNYMKRDPKTDELILDAYGNPIWEGFCIDSIKKLSERLNFGYMLVPPTSGEFGRRDVINDSWDGIVGDLVTGETDFAVTALKMYSEREEVIDYIAPYFEQTGISIVMRKPVRQTSLFKFMTVLRVEVWFSIIAALVGSAIMIWLLDKYSPYSYRNNRAAYQYPCREFTLRESFWFALTSFTPQGGGEAPKAVSGRIMVAAYWLFVVLMLATFTANLAAFLTVERMQTPVQSLEQLARQSRINYTVVEGSSTHQYFINMKFAEDTLYRMWKELTLNVTEDFQKYRIWDYPIKEQYGTILLAINGSEPVKDAKEGFRKVNEHENADFAFIHDSSEIKYELTRNCNLTEVGEVFAEQPYAIAIQQGSHFADELSYALLELQKDRFFEDLKAKYWNMSRIKACSVNEEQEGISLESLGGVFIATLFGLGLAMVTLVLEIIYYRRKYSAMHRFNEITKVKPASGTSIKQLLPKKKKKRIAVWHTSTSKRDNSPEHQTPPPAFDAVKFRGKKVPPSITLGGQEFKPRRAGLRQLSESLDSEEYRKEGIPANRDDELPPYTE

>ZtauIR93a

MRFHAFLWSLWLPLCLLALLPQNAEANDFSSFLTANASLAVVVDQEYMQRRGENILANFQKILSDVIRENLKNGGIEVKYYSWSQIRLKKDFLAAMTVMDCKSTWQMFDSTQQNSILLFAITDANCPRLPLNRAIMIPIVDEGQELSQIILDIKVQRLLRWKTAAVLLDQTILHDNPTLVESVVLESAKNHITPFSLLLYQIDDTLRSQKKRTAIRQMLNIFQDSTQTPRQFIVLSEFYEDIVEIAASMKLFHVYNQWVFFVLNEEQRSHDPMSVTQNLEEGANIAFMLNTTEPTCTNSINCTITELSMAFVKSISRMIVEEQSIYGEISDEEWEAIRFTKQEKQDEILGYMKEYLREYSKCTSCAHWKIETALTWGKSEEHRRYQSNLELRDTRNRNFEFIDVGYWTPTLGFNTHEVMFPHIVHFFRNITLDILTVHSPPWQILERNSRGDIVRHSGISMEILKEMSRLLNFSYILHEVKVNAADLAAEDMQHTNNVTDDLSGSLTFNIPYQVIETMQSSRYFMAALAATIDEPDKKSFNFTVPISVQMYTFISRQPDEVSRIYLFAAPFTTEIWGCLVAIIIITAPVLYFINRWVPMDHLRITGLSTLNSCFWYIYGALLQQGGMYLPKADSGRLVIGVWWIVVIVLVTTYSGNLVAFLTFPQFQPGIDYFFQIFSSNAVQQFGLRNGSYFEKYATQITTRDDFRDYIQRATIYSNVQGEDIGAVQDGKRINVDWRINLQLIIQKQFEKDKECKFSLGRDNFVAEQIGLIVPRDSPYLQLINDQITRMFQMGFIERWHQINLPSMDKCSGHGGMRQIMNHKVNLDDMQGCFMVLLFGFIIALFILFVEYWYRWYFVERKKGVFAT

>ZtauIR84a

MPSRRAHTARRQIFATTLTCGHTSFITILILLLSKWNLCVLAAANVYEFRAFADVLKQQHLQHAIIAYNSDTEQTQQQAGLLKDNALRALLNVASLHFYDVHQAKSTKNYTDFERLFYHDSPRVGIYVAQLEDVLLQQYVLGSNVISVDTIDAGGYRVRVDVGARFNNSRVWFIMSKQRAVATALVNVRRVLTPLAVNISADITVGVRLVDNNTIELFDIYKIQKDWLEIEPKGYWSSNVGLKLNLPFHQTFVSRRRNFKGLTLVGGIVIREQPAGMDDLEYLNSLDYKNCDPMQRKTYQLMKLMEPVFDVSFQPILKEIWGEQSANGSWGGVMKLLLSGEAEFSLCPMRFVMNRVHLIHYTIAVHTEFVFFIFRHPRRNDIRNIFFQPFVEEVWYTVIAIIVITTLLLQLHMHHENRFFINKDPHFQTRFDYAILSILEAFFMQGPANDAFNATSTRTLIFSVCLFSLLLQQFYGAFIVGSLLAASPRTITNLEALYNSSLEIGIENIPYNIETFEKTTVPLGNAIYKERVCKNRERNIISIEEGAERIKKGGYAFHVSANRIYYMLKELLTEKEFCDLQDVPFIPPYRIGIGITKTSPFREYFTTSIANFHTSGLLQYNDNQWQLPEMDCSLSQNHEVEVDLQHFLPALIFLISAMLLSLAVLILEIIYYNLEHSARLARLCPRIMPRPKLEFIN

>ZtauIR75d

MKTFNFNPHFILLICCILRVATSSGAEPDEFASAEQTGHSIAEEPTTQSSDAQIFMEYFRWHGVHNILLIVCPQDAATSEEHHKLKTLLRQFIANGFSTRIFNGEDYDEGGGAPSAMKAQTEPEPVNTTVTPTVDSTRGAPALSRTTYGPPRTFRSDNSTRRPLRLQLPALTYKSGILLWRFGSACALNVLRWAAAPEHNYFTTNRFWLLHTVEPTHISLLEDEDIFLPPDGEVRVVFQQPDAQFFALVDVYKIAADRPLRRTSVGGRDLRDAGDMLQALSKFGSAISYRQNLEGLTFKTGLVIAFPDMFTNIEDLSLRHIDTISKVNNRLTLELANKLNLRFNTHQVDNYGWHQPNGSFDGLMGRFQRYELDFGQMAIFMRLDRIAIVDFVAETYRIRAGIMFRQPPLSAVANIFAMPFASDVWIAILLLMIFTIGIFMVELVYSPHTHEIDILDCVVFVWGAMCQQGFYANLLNRSARVIIFTTFVSTLFLYTSFSANIVALLQSPSEAIQTLSDLTQSPLEIGVQDTVYNKIYFNESTDPVTNHLYHKKIAPKGENIFMRPPVGMEKMRTGLFAYQVELQAGYQIISNTFSEPEKCGLKELEPFQLPMIAVPTRKNFPYKELFRRQLRWQREVGLMNREELKWFPQKPKCEGGMGGFVSIGITECRYALGIFGFGLLLSAFSFILELAVNYAWNLVKKIHRNKKQREASGATNGYTGDFLY

>ZtauIR76a

MSTLSPAVNHWTTLLNFIIQTYFIDSHATCILWHRDFPFELQTPANSEFIAYINIWPDNLSQQSLQQDIFNFTAFAETQLAYGMQPDALVQKLTIAIRETHCETFVAFQEDIPSFARSFYNASRISVWRSLRNKFLFVYRKDLQPDTAAYFDDSLFKDQPNVLIVEAECGNCTTFALKTNKFIGPLAEHPEQLYVLDRYNAVDGKFEYGVDLYMDKVKNLQGREVTVGLFDYRPFTAIDYDRQPQVKDHSTENIRGTVHIDGTEVRMLFALCEVINCTVDADTSEDDWGTSYANLTADGIFGLITSRKSQYIAGALYFWPDDYRYLDMSLFIGRSGVTCLVPSPHRLTSWLLPLRPFQLTLWLGVFASLGLEAIALFFTRHLAPSPTEPQYGLMESFQFGYITTLKLFVSQGSDYVVNSNTVRMVLFACYMMDTIVTSVYGGGLSAILTLPTMEEASDSVERLYRHGIPWTATSPDWVISLRGSGADRDVVVESLLENYHVYTYEQLTQFAKTENMGFILERLAFGHFGNVDFLTDESFKRLKLMIDDIYFQYCFAFVPRLWAMLPKLNEVIMSVHSTGLDIFWEWEVAATYMDGQQQEEIQASMYMDFDVGPVKLDMGNFIGLVLPLIIGIVFSVFAFIGELIYY

>ZtauIR75a.2

MELMLLNFILHHFLSANINSIVVLNCWSAQTQCDFSKMLNEHSLYSRFVNIESIDMSADFEYRYLLHNRPGMGAYLDMNCKSSEQVLSTLNRSRLYNAHYNWLLYDRTADLNNFRRLFADANLDVDAELTYAVLKPTLFGLVSDVNITTFVKYDVYNNGYNLGGKLNMTVDREVECNVTSCYIKRYLSQLHTRSKYGNRDKLHDITMRVSVVITQLPLTTPVPMMLDFLISENNSDVDPISRFGYRIMLIFKDLFGCKMRYTFRSHWGINETYGGSIGDLISCEADFLSTPFLSTAARVKYVSPLLETGGFSSICIFRTPRSSSMKGEAFVQPFDGSVWLVFVILLIVVAIFLWYTFILEMRNFRTYLPYTPSLLSTGLLAFGSACYQGSHIVPSSVGGRLAYLSLYLATFIVYNYYTSILLSTLLGTPPKSDIKTLGQLADSSLTVSLEPLPYNYVYLNASQLPDVRRFVSRKIESRKNPQKVWIPVKEGVLRVRDEPGFVYVLETSYAYPFLERNFLPHQICDLNEVNLRPDKSLFTQLHKNSSYRELTRIRGIRMLETGVFRKHRRYWVRNKLNCVPSNYLFAVGMEYTAPLFLMLAFSYILCLLLLGLELLIKRLSA

>ZtauIR75a.1

MDLKLFNLILYYFLKLNMKVLVSFNCWDVETQLAFYKLAAENSFYIDYINLNDSKALQGIEYRLIAKKPTMGIFMDLNCENAEELLNIASQERLFSDHFFWLIYDDMANVTYFRGLFKRQNLAVDAEITYAYLNTPEDDGNSTAIASYTLYDVYNNGYYHGGKLNMTLDREIYCNQEECYVNKYLSKLHLRNKYGNRNTLHDATLRLTVVVTKIPITSTPEQIFAFLRSVNGTNYDAIARFGFQALSILVEFLGCKTNHTFVNRWTINETHGGLIGALAIQSADLISTPFIPTAPRMEFFTIIAETSSFRSICLFRTPRNSGIQGDVFLKPFNTTVWSLFGVLLLLTAVVLWSIFRLERYRMYKRYIDYMPSLLATFLISFGSACSQGSDMVPGSIGGRMVFYTLYLLTFLMYNYYTSIVVSSLLGSPVKSDIKTMGQLADSSLEVGLEPLPFTLTYLNNSLLPEVRRFKHKIDSVPNPQAIWMPLEKGILRVRDQPGFVFGFEASTGFLLVKRYYKPYEICDLNEVLFRPEKSLYSAVHKNYSYKEITKQKVIRILETGVNLKLHRYWVQTTLECFDSNFIVEVGMEYMAPLFMLLACTYVLVLMILLCELHKKYWTERKMRLENILFGQNWHNE

>ZtauIR64a

MKLFLKSAKAKTKATKTTHKNVLNMCMPSMQQRSLTTHQHRVPRRCWRVATRTPECFNNKIKMALLILIWTAIWCRRECVAAERHSNQAVYENNKSNNISNLHEQLEINAVDDNVDVEVLLGNVEVELSRVVAPIDLVDEGSLEQVLFKRDTAKATDGVSEELNATTGYEFTTLDAATTGVADTKRMMTQQITEIQTETETETTTTANDINSVATTMNSSVEINEYENVKNNGNENVERAKVVDRKIIAATTTRTAATALFAESDTATAAATPQTGEQQNIAAEGRESEKEEASAPEMQAEDGEEPVDVTAALQQQLIYEFAIKYKHIPRVTYFTCRWQSSEGSGGNTFGEQMTYLNMQTLEMMQRMYGSAAVDKMRSESDAKAKEKAKKASAPTTTTARAAPLSKAVETNTRKVRSNGNTRSKRTTTAAVTRAQTAAVLPLNNGNVTQSGKGSNRASRVTSDASAATTAAQWDILIKVVHIDQLINKRPTAANNKNGKNNRSYNWNVQRTFGGGFAAGGGYGRSAAGNAGARNAYTNANWLAQVLRDDGSRQLVVLNLACGAASRRLLEMASNKALFNATYHWLLIEDYTFNRNADIDDNVDNDRNGNNNENANCKRTSAHQYNETVDSDNNSNNNKFSEQTETQQLQESKEEMKKKQRKELTATTSITPRPKATLTAAAALTVTANNENTMEIIEKYLQKLNININTELILAKRHTRRIRDDAAQIGDNKKCAATNDDAADGGGSGSGGCCTSSDSESATNNTLQRKDYYQLYDVWNPGLQYGGQLNISEIGYFALDDGLQIALWYRRSTTITRRMDMKMARIRCLIVVTNKNHTDTLEHYLTTHYDTHVDSMNRFNFALLSNVRDLFNFSFVLSKTASWGYLKNGKFDGMIGALVRKQADIGGSPIFFRIERAKVIDYTTRTWVARPCFIFRHPPSTKKDRIVFLQPFSNMVWILLGLCGIFTICLLWLLTSVERRLEAVGVVEQLGHSTRSSSGSNMHSNATNNKQLCAIGGNDELPPPVGCRCSTRMPLSAGTMPSCGACNAPSVGLATKEQRKQKLEKLMQRRGKTEARRKNVHGISCRRCMLGCGSACCGQTGSVAAQQRTGLFFESVLFYVGSICQQGLTFSTSFFSGRCIVITSLLFAFAIYQFYSASIVGTLLMEKPKTIRTLRDLIHSSLAVGVEDIPYNRDYFLRTKDPIAIELYAKKVTSVPTDNEALSETTADNVTTLSPLQPGVELTAAEKAKTYREILHSHETGAHAKTNEASNWYEPEYGVKRIRKGKFAFHVDVATAYKIIADTFSEKEICELTEIQLFPPQKMVAIVQKGSPLRKTITYGLRRVTESGLMDYQRKVWHSPKPRCVKQIHTDDLRVDLQTFASALLVLIFGYAVSLLALSIEIIQHKLWQRYRAEQEDEEENE

>ZtauIR41a

MLPTNLLYSFYWSRMFNVIMQNYLLSTTTCIIWPENEDFSISWQQDKPPDAAIISIRLHDLAQSFSKDVVDFAAKREELLNDYVVLNPFVEKLTLSIEKSHCQNFIAFQMDIPIFIDAVINASRFSIWRSSNNKFLFVYNKDDLLQQLFEHRFFEDQSGILLIERSIIDPAVFDLKTNKFVGPRADNPKQLYLLDTFHAETNTFLHGNDLFPDKLSDLQGREVILAAFDYRPDVVLKYYPGAPSRDRAFAANDTSGDVELDGTEERILKTFCEKHNCSVDIDTSEADDWGIAYRNMTGEAALGMIARGKAEVGMSAMYTWYADYVALDMSMYIGRSGITCVVPAPKRLASWLLPIEPFQPALWAFVFVCLCVEIIALLFIDHARPIIIALSERMRASEEQQNSWVRNFQYAFTTTMLLFVSQSNKGTMVNFTPLRVMLFASFLNDIVITSIYGGGLSSILTVPSFGQAADSVERLYAFQLKWGADSEAWV

AGIRDDESEIMKGLLRNFDIYSAEQLMELAQTEEMGFTIERLPFGHFAVQEHLTRSVLGKMKIMVEDIYFQYTVAFTARMWPLLEGFNEMVVMWHSSGLDKFWEWRIVADNLDGAIQKELMASQYSNLDDIGPVKLGMSNFVGMLLLWLLGITCAFLAFLAELLMDHMKRSKKMEEFEVIEIGEGSV

>ZtauIRkainate2

MKFVLLHITCALLYASAYAAEERPKFNVGIIFASKNDETEIAFRTAIERANVFERSFELEPIVEYADTDDSFMVEKTVCKLIAQGVIAIFGPNTAGGTDVVTSICNTLDIPHIVFDWTPSEALSNRQHSSMTLNVHPNNILLSRGLAEILQSFSWRSYTIIYETERELQQLQDILQVGEPSSNPTSIRQLSEGPDFRPFLKNIKLSTDNCIVLHCSTDNVMKILNQANELKMLGEYQSVFVSVLDTHTLDYQELLTVTANITTVRLMDPTDYQVKNVAHDWEEHEKREGRYYRADPSQVKTNMILANDAVTMFVKGLAELGIAEELNPPKLECRKNRAWAHGRRIIEFLKARSVEGATGRVDFNEYGERNFFTLRFMELTPAGFLDLSTWDPVNGVDALEKEDASEKRVGEKLSNKTFIITSRIGAPFLLNREPKDGEILQGNARYEGYSMDLIDAIARLLNFKYEFVLAPDGKYGSFNKLTQSWDGLVKQLLEGNADLGICDLTMTSTRRQAVDFTPPFMTLGISILYAKPEQPPPDLFSFLSPFSLDVWVYMATAYLCVSLLIYGLSRMAPADWENPHPCKEPEEVENPWCMSNTTWLAVGSIMGQGCDILPKAASTRLVTGMWWFFALMMLNSYTANLAAFLTMSRMESSIESAEDLAAQSKIKYGALLGGSTMGFFRDSNFSTYQRMWTAMETARPSVFTKNNDEGVDRVLKGKGRYAFLMESTTLEYIIERNCELMQVGGWLDYKTYGIAMPFNSPYRKQISGAVLKLGESGMLSELKRKWWKEMHGGGSCSQTESSGGDTPELDLENVGGVFLVLGIGLLTAILIGMCEFLWNIKAVAIEEKISLSEAFKAELMFALRFWIQTKPVHTASTSSGGSSSSSSSKSSKSSKSSSSSSSSASKRSKRSSKSYARSISQSTKSITNATHDPDKNDLSVHDKLRKISSMFSLKSARSEPSVANVHLEAPPALKHNSHHNHNPVNKSTHTPMVREVAQQTTLTTDDGEEEQAPQLKIDEIPIVEPHHHHNHNHHHHHHHHRHHEHNHQQPEEDHQPAIPLIERTQNGKVARNGHAGFGNAEV

>RpomIRkainate2

MQLRSDNLIVLVFLVQYTRIGHCDAQSRRYSSRGNDYEDNRNGASGTIPIGLITDQYTEQMGLIFEHAIEVANAELETPLKAVKEEMNYGDSFQAYGVLCKLLKDGVGGIFGPSSRHTAKHLSTICDTKDVPYFFSDMNENSEAFNLYPHPLDLSKALYFLLSAYEWSRFIFLYESVEYLDILNGILAFYGTNGPTITVLRYDMELNGNYKTGLMRVRKSGDSRIVVAGSSDTMPEFLRQVSTMSISADRYLPQLYHNPKNTYIPHSCIGYQDLTRTKYTVLFYYGSSAVLRSCPITLSMALIYDSVQLFAETTKYLTVQTVPLNCSDRSESVLDDGSTFKNYMRTLNLKERTITGKIYFEGNIRKGYALDVIELHPSGILKIGTWDEISNLTIQRVPQTNSVIDNVDNSLANKTFIVLLNVPNKPYASLVESYEKLEGNSQYEGYGVDLIKELADKLGFDFILKNGGNDYGSYNATTNTTSGMLKEIIMGRADLAITDLTITAARQQVVDFSIPFMNLGIAILHLKAQKAPPAFFTFMDPFSKEVWWLLGLSFLLVAFSFFILGRLSPSEWDNPYPCIEEPTELVNQFTIGNSLWFTTGALLQQGSEMEPKAISTRTVAYFWWFFTLLMVSSYTANLAAFLTIENPTSLIDSIDDLAENKHGVLYGARKVGSTREFFEKSEDPRHIKMNKFLNSHPELLTNDNMEGVNRVDMTYAFLMESTSIEYNTMRLCHLRKVGDALDEKGYGIAMRKNWPYRDRFNNALLQLQEQGSLEKMKNKWWNEVGAGICTVEYN

>RpomIRkainate1

MKVLLHVLLSCMLFQLTAQLTTAFEVVKIGVIFFNDEWELVSAFDTAIRDINELEMDVRLEPIKHFISYDDSLTLQELACDLIDNGAAAIFGPSSKTNSDIVEVICNTTGIPHLQFDWHANEAYGGHSRNHKLTVNVAPTEQMLSCAFLDIMRMKQLDWKSFTIVYENSRSLARMQRLFGWRQLHKAGIKLWHFNRGDDYRVIWKLISNSREKYVVLDCPSDIITEVLNASIYFNMTGQFNHWFLTSLDTHTSNIRSLYSRNFVANMIAVRVRPYMPPPVHDEADVFENEQEDQIITIRSKLLYDAIVLYFNALRRQIRNLHYQAPKVRCHRGYWRTGLELLDQMKLLTSRNVTPPYKTQKMQLNKYGEREEFNLEIYNPMVERITHIWNKNKNLVAFEDLVEAKEMKKQKYNEVEDFTPKRVKYTVATRIGEPYFMWRQEPEGVHYEGNERFEGYAVDLIYSLAQQCKFDFVFEPVPDNNYGSYDPITDEWNGIIRQLIDNNAQIGICDLTITQARRSVVDFTVPFMQLGVSILFYREPPPPKNLFGFLSPYSLDVWIYLLVAIMITALVLVVVGRVSQLDWISPIPHDPDPAEVENIWNLSNSLWLNIGSILNQGCDILPKGPAMRLFTAFWWIFSLLLSQTYIAKLAAFITASKMESSITNLHGLIDQNKIQFGMLKGGSTSFLFSESNESEYRLAWNKMVAMKPDAFTSNNREGVDRVKRSRGRYAYLLETTTLQYYLNLNCELKQIGEPFNEKHYGIAVPLNAPFRSNLSVGILKLSEKGELYKMKRKWFTTNETNCDVDLDEDADNGQYTMESVGGLFIVLIGGILVSILIGIFEFLWNVEQISVKEKLPTMVIFKAELKFFLRFWQTRKPLRTYAESRGSTSTGYSSFEQTASTSTAKKKRKKKSKKIHEQ

>RpomIR25a

MHKSSCKRLYSIFIILKILSFTSIATGQTNQNINVFFVNDADNEPAAKAVTVVTTYLKKNPSYGISIQIDQVETNKTDAKSLLESICSKYAESIDRKQPPHIVFDTTKSGIASETVKSFTQALGLPTVSASYGQEGDLRQWRDLDESKQKYLLQVMPPADLIPEVVRSIVRKMNITNAAILYDDTFVMDHKYKSLLQNIQTRHVITGIAKEGKREREEQIEKLRNLDINNFFILGNLMSIRMVLESVKPSYFERNFAWHAITQSEGEVSSQRDNATIMFLKPMSYAQNRDRFGRLKTTFNLNEEPQIMSAFYFDLALRTFLAIKDMLQSGAWPKNMEYLGCDEFQGGNTPERNIDLKAAFTMQAPFIIKDETAPKGYKGYCIDLINEIADIVHFDYTIQEVEDGKFGNMDEKGEWNGIVKKLMDKQADIGLGSMHVMAEREIVIDFTVPYYDLVGITIMMQRPQVPSSLFKFLTVLETNVWLCILAAYFFTSFLMWIFDRWSPYSYQNNREKYKDDDEKREFNLKECLWFCMTSLTPQGGGEAPKNLSGRLVAATWWLFGFIIIASYTANLAAFLTVSRLDTPVESLDDLAKQYKILYAPINGSSAMTYFQRMANIEQRFYEIWKDLSLNDSLSPLERSKLAVWDYPVSDKYTKMWQAMQEAQLPATLEEAVERVRSSTSATGFAFLGDATDIRYLVMTNCDLQIVGEEFSRKPYAIAVQQGSHLKDQFNNAILTLLNKRQLEKLKEKWWKNDETQAKCDKPEDQSDGISIHNIGGVFIVIFVGIGMACITLVFEYWWYKYRKNPRIVDVIEANSGGKDGKGGDGVILGQTGKEYDKSGNTVLRPRFQQYPATFKPRF

>RpomIRNMDA2B

MKGLIFSQKANKRRRRKNVKSSQTPTGSIALARRRVKTKTMARICIATTATKTQEHNSNSNNAQLAAPLICFNSNSCNGIANTNSRRSSSFGSNLVPTFNQQHHNQHHHHQQCLAPGQLTLKMPPYMPPIFKTTTIQIASQRIRSIDVVEVEEQLHHQQQQQQQNQLKSVQNHCFAHQHQQLQCQPLSLSPPPRFASSHRPLQLLPAATTASWPTLIWRTLSSRLNFHFGIAVIVLASILMYSCPCASALRLTNGNTKTLSANKEQLNIGLIAPHTNFGKREYLRAINTAVQGLAKTRGAKLTFLKDYSFEPRNIHFDMMSLTPSPTAILSTLCKEFLQANVSAILYMMNNEQFGHSTASAQYFLQLAGYLGIPVISWNADNSGLERRASQSTLQLQLAPSIEHQSAAMLSILERYKWHQFSVVTSQIAGHDDFVQAVRERVAEMQDHFKFTILNSIVVTRTSDLMELVNSEARVMLLYATQGEAVTILRAAEEMKLTGENYVWVVSQSVIEKKDAHSQFPIGMLGVHFDTSSAALMNEISNAIKIYAFGVESYLTDPANRGRRLTTQSLSCEDEGRGRWDNGETFFRYLRNVSIEGDLNKPNIEFTADGDLKSAELKIMNLRPGANNKNLVWEEIGVWKSWETQKLDIRDIAWPGNSHAPPQGVPEKFHLKITFLEEAPYINLSPADPISGKCLMDRGVLCRVAADHEMAADIDVGQAHRNESFYQCCSGFCIDLLEKFAEELGFTYELVRVEDGKWGTLENGKWNGLIADLVNRKTDMVLTSLMINTEREAVVDFSEPFMETGIAIVVAKRTGIISPTAFLEPFDTASWMLVGIVAIHAATFMIFLFEWLSPSGYNMKLYLQNTSVTPYRFSLCRTYWLVWAVLFQAAVHVDSPRGFTSRFMTNVWALFAVVFLAIYTANLAAFMITREEFHEFSGLNDSRLVHPYSHKPSFKFGTIPYSHTDSTIHKYFKDMHLYMRQYNKTSVAEGVAAVLNGNLDSFIYDGTVLDYLVAQDEDCRLMTVGSWYAMTGYGLAFSRNSKYVQMFNKRLLEFRANGDLERLRRYWMTGTCRPGKQEHKSSDPLALEQFLSAFLLLMAGILFAALLLLLEHVYFKYVRKRLAKKDGCHCCALLSLSMGKALTFRGAVFEATEILKKHRCNDPICDTHLWKVKHELDMSRLRVRQLEKALDQHGIKPPQLRLASSSDLLNHHHLKERPPLLGNLSLAASAQDLYRWFRNS

>RpomIR75a

MNPLLLNFILHHFWNANINAIVFFNCWSLQTQQHFMHMTNERMWYTRFVNIESMDLFGDFEHRYLLHSRSILGAYLDMNCNKSEDVLNTLSRWSLYNQHYNWLLYDRTADMHNFKRLFVNASLSVDAELTYVILKPALMDANTDTNTTCYTTYDVYNNGYALGGELNVTVDREIQCNLQDCHVRRYLSDLHLRSKYSNRLRLHDVTMRVSTVVTKRPLSLPPPLLLEFLNSEYNTEIDAISRFGFRVLLIFKDLFGCNMEHTFRDRWSMNETHGGIIGDMITKDADFVSSPFLSTKPRQKHLSLMAVTGAYRQLCLFRTPRSTSIRGEAFLQPFDTSVWVVFSIILGLLAIFLWRTFALESRNFRQRMPYEPSLLATTLLAFGSACYQGSNIVPFSLGGRLAYFSLYFATFIMYNYYTSTLLSTLLGTPVKSDIKTLGQLADSSLEVGLDPLPFTFVYLNASQLPEVRRFVYRKIASKPHPEKVWIPVEEGILRVRDDPGFVYVLETSTSYPFLERSFLPHEICDLNEVLLRPDRGLFTQLHKNSTYKELTRLRVSRMMETGVWRKHRRYWVKEKLNCVPGNFLFAVGMEYTAPLFLMLLFSFALCLVLLVLELGIKYLLDHQRDNTIGFWRSDLAEGFIE

>RpomIR93a

MRFQASFWLLILPLCLSLLPQTIEANDFSSFLTANASLAVVVDQEYMQQREENILANFHKILSDIIRENLRNGGIEVKYYSWSQVRLKKDFIAAMTVMDCKSTWRFYEYTQQTAILLIAITDANCPRLPLNRAIMIPIVDEGQELSQIIFDIKVQRILRWKTAAMLLDQTILNDNPTLVESVVHESAKNHITPFSLVLYKIDDTLRSQKKRTAIRQTLANFLDNARTARQFIVLSKFYEDIVEIAASMDLFGVFNQWVFFVLNEEQRNYDAMSITQNLGEGANIAFILNSTVPSCVSSINCTISELSMAFVTSISRMIVEEQSIYGEISDEEWEAIRFTKQEKQEEILGYMKDYLRANSKCSSCSHWKIETALTWGKSEEHQRYLSNMDLRDTRNKNFEFIDVGYWTPILGFITHEIMFPHIEHFFRNITLNVLTVHSPPWQILERDNRGDIVKHSGIVIEILKEMSRMLNFSYNLQEIKVTAIDLVADDVQQVGNITDDLLGSLTFNIPHQVIETMQSSRYFMAALAATIDEPEKKSFNYTVPISVQPYTFISRQPDEVSRIYLFTAPFTLEIWGCLVAIIIITAPVLYFINRWVPMDHLRITGLSTLKSCFWYIYGALLQQGGMYLPKADSGRLVIGVWWIVVIVLVTTYSGNLVAFLTFPQFQPGIDYFFQIFASSDIQQFGLRNGSYFEKYSTITTRDNFRDFVQRAIIYNNLQGEDIGAVQQGKRVNLDWRINLQLIIQRQFEKDKECKFSLGRETFVDEQIGLLVPRDSPYLQLINDQITRMFQMGFIERWHQINLPSMDKCNGHGGMRQITNHKVNLDDMQGCFMVLLFGFMIALFILFCEYWYRWYFVEKKKADFAY

>RpomIRdelta-1

MDIILRDLGRVEWERTGNYTLILAIAWTILNVYAKYPSTTLIISQYASELQSNLHQAELIDALMVYLEKRHHHVYIMEQEKMFDDVDRSEWGDISDTAIWFIDSWNSFGALAKDLDHPQSAYQRSGWFLIVYTGTEPERLETVKNIFSRLFELFFVNVNVFLLMDATPFVYTYFPFSRTKCHSAKPELLMSFRNKRPRYIHKSHRRFFPPKVNNLHGCELGVITWHDPPFIILKTVDETGHIVSIDGIEGMLISLLSEAMNFGIRIVDPQPHDRGAIYANGTLTGVTRMIVEGEGNISIIYFMYEKKRAQIMDASCSYMAFPLLVAIPPGRPISPLQRLFRPFKYIIWSLIGSNLVLAVLIIYALKLFGSKRVVSFVFGKANRFPFSNLWASLYGGGVIHNHLPYRNFSRYLLGLWLLCTLVLRSAYTGQLFIMLQDGRALTPLKTFQEIFEKKFVVNTAPVLAELLTSVMGNVAVVNIDGGNNSMPMILKRIARGSKEILSIIEPAVMYYNYQQETEDERVAILPQKLIMTPLTMYMRKHSYLTIPINAKLLDFIDLGIINKFENRYRLPDSGEVSQEPVQLSLFLLTGIFGVYLVLMGFCTLVFLLELWTTRSPLAKMAIDFLNYYWKGQLIFFEYILKESRRNVAFIGPSDFLYNYMHAEKKHAFFKILKQKVLNLHTTIYLTKHSYLIDEFQNQILWVQAAGLIDAWARWELYDDKKDLSGFGEDDMQQAKELYVLIIIGTYEGKMG

>RpomIRNMDA1

MKALIDISTACLLVCSIHLGAIAQKHIQHSDNPSTYNIGGVLTDPDSEAHFRTTIAHLNFDQQYVPRKVTYYDKTIRMDKNPIKTVFNVCDKLIEKRVYAVVVSHEQTSGDLSPAAVSYTSGFYQIPVIGISSRDAAFSDKNIHVSFLRTVPPYYHQADVWLEIMFHFGYTKVIIIHSSDTDGRAILGRFQTTSQTNYDDIDVRATVEMIVEFEPKLDSFTEHLIDMKTAQSRVYLLYASTEDAQVIFRDAALYNMTEAGHAWIVTEQALHANNTPVGVLGLVLEHAKSDKGHIRDSVYVLASAIKEMMSNETITEAPKDCGDSGVNWESGKRLFQYLKTRNITGETGQVAFDDNGDRIYAGYDVINIREKQKKHVVGKFYYDTEKAKMRLRINDSEILWPGKQKKKPEGIMIPTHLKILTIEEKPFVYTRRLTEDEVNCDEDEIPCPLFNATDGSENENCCRGYCIDLLNALSHRINFTFDLALSPDGLFGHYTLKNVSSSTSGAITSRKEWSGLIGELVNERADMAMPLTINPERAEFIEFSKPFKYQGITILEKKPSRSSTLVSFLQPFSNTLWILVMVSVHVVALVLYLLDRFSPFGRFKLSHTDSNEEKALNLSSAIWFAWGVLLNSGIGEGTPRSFSARVLGMFWAGFAMIIVASYTANLAAFLVLERPKTKLSGINDARLRNTMENLTCATVKGSSVDMYFRRQVELSNMYRTMEANNYDTAEQAILDVKKGKLMAFIWDSSRLEYEASKDCELVTAGELFGRSGYGIGLQKGSPWTDAVTLAILEFHESGFMEALDKHWIFHGNAQQCELFEKTPNTLGLQNMAGVFILVAAGVAGGVGLIIVEVIYKKHQVKKQKRLDIARHAADKWRGTIEKRKTLRASLAMQRQYNVGLNANPGTISFAVDKRRYPRLGPRAPEQAWKSDADILRSRRYMDETTKGVNSPAIHVPMLGKVRSPGNMLPPRNMLPPRYSPGYTSNVSHLVV

>RpomIR1

MLNGLKLFTFLWLAHAAANTWMNFNGVQAQPSSLTEKIPLGAIFEQGTDEVQSAFKYAMLNHNLNVSSRRFELQAYVDVINTADAFKLSRLICNQFSRGVYSMLGAVSPDSFDTLHSYSNTFQMPFVTPWFPEKVLTPSSGFLDYAISMRPDYHQAIIDTIQFYGWRKIIYLYDSHDGLLRLQQIYQGLKPGNESFQVEMVKRIANITMAIDFLHTLEDLGRFNNKYIVLDCPTEMAKQILIQHVRDISLGRRTYHYLLSGLVMDDRWESEIIEFGAINITGFRIVDTNRRFVHDFLDNWKRLDPATSMGAGRESISAQAALMYDAVFVLVEAFNKILRKKPDQFRNNIQRRGQQTLMAAASTSINGTMGMTGGGNAGGMGSGIGGSGGGGGGGGGGGIGPGGGSGGSNTPRALDCNTSKGWVNPWEHGDKISRYLRKVEIEGLTGDIKFNDDGRRVNYTLHVVEMTVNSAMVKVAEWSDDGGLQPLSAKYVRLRPHVEIEKNRTYVVTTLLEEPYIMLKRPSIGEQLDGNDRFEGYCKDLADLLAKKLGINYELRLVKDGTYGSENPTVRGGWDGMVGELVRREADIAIAAMTITAERERVIDFSKPFMSLGISIMIKKPVKQTPGVFSFMDPLSQEIWMSVIFSYIGVSIVLFFVSRFSPYEWRIVQYQTDSHAHHEQMANQQPPGIIGGNRLSQPSSHVPNSPVNEFSILNSFWFALAAFMQQGCDISPRSISGRKVGAAWWFFTLILISSYTANLAAFLTVERMVTPINSPEDLAMQTEVQYGTLLHGSTWDFFRRSQIGLHNKMWEYMNSRKHVFVSTYDEGIRRVRTSKGKYALLMESPKNEYVNAREPCDTMKVGRNLDTKGFGIATPIGSPLRDPINLAVLSLKENGELIKLRNKWWYDKTECNLNKDNQETSRSELSLSNVAGIFYILIGGLLVAVFVAIIEFCFRSKTSTQKANGSMLSSASGGGSHQRNSLTDAMHSKAKLTIQASREYDNGRVGITGDLDGTLGGYTAIRLLGDIFVMAALTLLNTAIVIAVRETSEGRGEVCRQYVWGAIAYVPLFSPIDLFAFSSDSKHDAALVALILVIVCLLIGALVLLFASQMPLSPPEWWGGTQRPACSSIPCRQYVAIALKYSCLHLSPYYSAHSGAAFTVFSFGHSAIRMRSPILG

>CstyIR25a

MVFSYFNIFKIIFFIILEISVKDKVVRGQTSQNINVLFINELDNDPATKAIETVQTYLKKNSNYGLSLQIDKIEANKTDAKALLESICTKYAQSIENKQPPHIVFDTTKSGVASETVKSFTQALGLPTVSASYGQEGDLRQWRDMEENKQKYLLQVMPPADIIPEVVRSIVRKMNITNAAILYDDTFVMDHKYKSLLQNIQTRHVITAVADGERARADQIERLRNLDINNFFILGSLKTIGQVLESVKPAFFERNFAWHAITQNEGEVTSKRDNATIMFLKPVVYPQNRERLGLLRTTYNLNEEPQIMSVFYFDLALRTFLAIKEMLQSGAWTANMEYLGCDDFQGGNTPERNIDLKQYFIQVTEPTSYGDFELVTQPGQTFNGYSYSKFEMDVNVIQIRGGNSVNSKSIGTWTAGLDSSLTIKDEEAMKNLTADTVYRIFTVVQAPFIMRDETAPKGYKGYCIDLINEIAEIVHFDYTIQEVEDGKFGNMDEKGDWNGIVKKLIDKHADIGLGSMSVMAEREIVIDFTVPYYDLVGITIMMQRPSTPSSLFKFLTVLETNVWLCILAAYFFTSFLMWVFDRWSPYSYQNNREKYKDDDEKREFNLKECLWFCMTSLTPQGGGEAPKNLSGRLVAATWWLFGFIIIASYTANLAAFLTVSRLDTPVESLDDLAKQYKILYAPLNGSSAMTYFERMANIEQMFYEIWKDLSLNDSLTPLERSKLAVWDYPVSDKYTKMWQAMQEAQLPSTLDEAVARVRNSTTATGFAFLGDATDIRYLVMTNCDLQVVGEEFSRKPYAIAVQQGSHLKDQFNNAILTLLNKRQLEKLKEKWWKNDEAQAKCDKPEDQSDGISIENIGGVFIVIFVGIGMACITLVFEYWWYKYRKNPRIIDVAEANTPPGKDVKLAEGVILGQSGKEYDKANAALRPRFNQYPQNFKPRF

>CstyIR8a

MSFIRVAVVLLSFLNKVQCNDLNIVFWVEPVQKDIYSDINAALKEVDNLHLEAEITDAVMVVESSGGDNNDAEEVNNRNMKTFCDILSTLEVSIILDFTYHPWHQGLDYVQSHNIPYLRVDRMLKPFLQAFSEFLTQKGGNDVVLILQNEQDRMEALLQVVEGYPFRTLIMNAGDNNVDFVQRLKDLRPSPSYYAIFAKGTNMNAIYEKLSTGNVFLRNPEWHLIYLDTKDRVFKYKKQVEKANKFGLNPRSICRSMQMKDAYCLSGFTLQRALLLDIFRSLIDIRQSNLDWLKPLAAECNSTSEEADPFVSNFEILDHFRLTNFMSFATTNPANQLEDEVDVIPPLTYTVNMTITFYSSEHDAVTDLANWQNGELKKLIPTINPAKRFFRIGTTEAIPWSYYRRDPETGEKILDSNGKPTWEGFCIDFIETLSEKINFDYELIDPAKGKFGKRDLETGKWDGIVGDLVSGETDLAVTALKMYSEREEVIDFIAPYFEQTGISIVMRNPVRQTSLFKFMTVLRVEVWLSIIAALVATAVMIWVMDKYSPYSSRNNKDAYPYPCREFTLRESFWFALTSFTPQGGGEAPKAVSGRILVAAYWLFVVLMLATFTANLAAFLTVERMQTPVQSLEQLARQSRINYTVVDGSDTHQYFINMKFAEDTLYRMWKELALNASRDFHKFRIWDYPIKEQYGHILLAINSSMPVKDAEEGFRKVNEHENADFAFIHDSSEIKYEITKNCNLTEVGEVFAEQPYAIAVQQGSHLADELSFALLELQKDRYFEELKAKYWNHTRANCPLSEEQEGITLESLGGVFIATLFGLGLAMVTLALEVFYYKKKLNKTSIKGSITQVKPLDSSQKDTDVWHTVKEKKNPTPPPSFETATFRGRKIPDGITMGSEFKPSRLGLQAH

RRLGSGDTLKESELPAYVE

>CstyIR93a

LGTLTYIIPFQVVELLQSNKFFMAAVAATVDEPDKKHFNYTIPISIQKYSFISRKPDEVSRIYLFTAPFTLETWASLICVILITSPVLYVINRLVPTQDLKVKGFCTVKNCFWYIYGALLQQGGMYLPQADSGRLVIGFWWIVVIVLVTTYCGNLVAFLTFPKFQPGLDYFFQLFKHNEYEQYGLRNSTFFEKYAMVSTRNEFHRYMEHAIIYNNMKEENIDAVKAGSRVNIDWRINLQLIIQNHFEKDKECTFALGKENFLDEQIALMMPSNSPYLNLINEQITRLSQMGFIERWHQSNLPSMDKCNGRGVLRQITNHKVNLD

>CstyIR92a

NYKYRDINFELWTQQYGGAEGNLNAIHLDTYISGNGTFAKNLQLYPNKLWNLQQRTLRIGSLTYVPYVKTYYVPTGEGDVDSINSKGPPKTVQFIGVEAELMKSFCHIRNCHLRVEPYAADNWGIIYDNGSADGMLGDLYKQRTEMAIGCIYNWYNDITETSQFIARSAVTILGPGPAQFPRWRTCIMPFSFGLWIFLAFTMIMCSLMFHFIKYTSYRFKHIYSARPNKYRSAKNYEKTLLDIFAVFIQQPSADTVLHRVATRIFLAFLLSATITLENTYSGQLKSILTMPLFNKPVDTMYKWSLTGWKWAAPSIIWVHTVENSDLAMEQRLAKQFEVRDYEFLYNATFWDNYGFGVERIYSGSFSFGDYITGSALENKVVPKDDLYFDWTRAVSIRGWPLMPLLDRHILFCLETGIYIHWERRFSYKFLDHQVQDILKMLASGSKPNSPPQKLSIEHISGPLFILLFGYILAFLVLMIELFGNRLNLKFLAGGRYLIKMN

>CstyIR76a.2

QAFPKASTYSIWRSMDNRFLFAFNKQQQKQDYFQASFFKNHANILVLEADYLNSSVFQLKTNKFVGSSTQQPEQLLYLSTFYALNRTFVPLIDLYAKDKLNNLQGREIIVGAFDYRPFVAVDFKRLPQYYDLAADNPSHLVHVDGSEIRMCHTFCALYNCTLQFDTSDKDEWGLAFDNYTGYGLIGRIIEGKIHVAMCAMYAVHSDYAAIDMTAYLGRSGVTCLVPAALPQVSWYLPLRPFRLTLWLSVLACLCLETVVLFLARQFEQSLANKPDSWWSCLEFGYMTTLKLFINQNSDYSVESHTVRTLLFACYTLDIILTSVYGGGLSAVLTLPTLGETADSVEKLYKYNLSWTGSSLAWLESINGTHEDKNSLYKRLVDNFVLQSMEEMRSKVKTVNMGFVLERLTFGHFGGGDFITPEALQRLKLMVDEIYSQFTVAMVSRLWPHLSKFNDLSLRWHSSGLDKFWEWKSTAEYLNVNEQNQVEASKHINYDMGPTKLNTQNFAGVLVLWSVGMLMSVLAFGAELAWFRLGRRMKDKHVCAIKKREKTIILANAKNSVEN

>CstyIR76a.1

LSYFYFSIDNMLPAANYWPVLINILIQKYLSHLTTTCILWHADFEVILEPAISYNVLLHINAKHLNASFSRDVYNFTQQDEYFNGNLVYYDDLVKKYTAAISNTYCEGYLVFQDDIPLFAETFRKASLYSIWRSIYSRFIFVYVKDQQEEDYFQDILFKNQASLLVVEADSLNSSTFALKTNKFVGPRNEHPEEMFLLSTFNALNKTFTPDIDLFSNERLMDLKGREVRVASLDYRPFVAVDYNRSPFYYDQAEDNPRHLVHVDGTETRVCHTFCELYNCTVQFDTSEKEEWGTSYTNFTGNGLMGLIMSGKVDMTSGAMFAWNTDYMNVDVTHFLGRSGVTCLVPAPRRIVSWDLPLKPFKMTLWVVIVAVLIVESVSLLLARQFEQTMEYPENKHQWMSSYMFGFISAFKLFLSQGTDYVVQGDTLKVILFACYMLDIIITSVYGGGLASILTMPILEEAADSVERMYTHNLKWAATSYVWVESIKDVDDDPIIMRLLANFKTYKFEDVREMAKTEYMGFAVERLEFGHFGNFDFITPESINRLKLSLDDVYYGYTVAMVSRLWAHLPIYNDLIMAWHSSGFDKIWEWRITADFLNINEQNQVIASRYYNFDTGPVELGMSNFGGMILLLVIGMGASMLVFAGELLVYKMIQRKLKLKEQEVEEGAQEIF

>CstyIR41a

MLSTTVYWSQVISAIAQLYLKDTTICLIWNSELEVDLKTVNYLDYYAVININLNNLQSCFEKDIDDFNALEKKLTEKGLSYNELTEKLTMAIEKTHCESFITFQHDILNFIESFINASQYSDWRSKRNRFVFGYIEDNFEENFQQLTFFEEQDSILLVSMDSTSSSDIFILKTNKYYGPKAEQQNSLVFLDKFYAENATFLYNHSLFPDKTANLRGREVIIAGFDYRPYFVINYGEKDNNSYDLAFGGSTLGAVQIDGTEARVVSTFCEIYNCTVWIDSSEANDWGEVYPNLTGDGSLGMVVKDMAEISIGAMYSWDVDYLQLDMSMYLVRSGITCLVPAPRRLTSWLLPLQPFQFTLWLAVMGYLLVEIISLCFAHKFESNLSTKRVSWHYSFKYAYATTLKLFVSQSGNSYVSSLTVRVLLFTCFMNDIILTSIYGGGLASILTIPSYEKAADTVDGMLYHKLQWAANSEAWVSAIRSTDDDRMNGILYNFFIYNDDELEVLATTRSDMGFTVERLPFGHFAIGDYLSSQTIEHLKIMKEDLYFQYTVAFVKRCWPLLDRFDHLIYWWHSAGLDKYWEWRIVAVNLNVQKQKQVEATMYSTMEDNGPVKLGMSNFAGILLIWVLGISVAMMAFVYEITEQYFKCKKSNV

>CstyIR40a

MLKITNIIFICELIHQATVKAMVILEPEKNISDVSIGLSQIVKSLHPNQLAILVKPSETFNQSWDDYQLLDEDNQNLQLHIDEFLQNLHTLNFKSVIFYDTELFFKFIEASLHGSIESVNLIFYKPYDLMQRIHDRKLAHRLSLFIFYWGAKYPPKRAELNFREPLRAVVITRPRKRAFRIYYNQAYADGLGRLKLVSWYDGDNLGLSKEPLLPSAKSVYSNFQGRVFRVPVFHSPPWFWVNYDNDSYNNNSLEFTISEENPLTEDTIEFKEVNVSGGRDHRLLQLLAQHMNFEFVYIEAHGRTQGSLRTDNEANDTFTGGIGMLQIGLADFFLGDVGLSWERRKAIEFSFFTLADSGAFATHAPRRLNEALAILRPFKADVWPYLILTVIVSGPVFYFVISTPYRWHPSTVKRLSKKISKHGAFHMCYIKEITRMDTQFQRRLAKMEQHFRMRQEMVELPPNLFNRCIWFTVQLFLKQSCQELYNGYRAKFLMTVYWIAATYVLADVYSAQLTSQFARPARERPINTLHRLQSVMIHDGYLLYVEKESSSLEMLENGTEIFRQLYALMKLQSPDEEGFLIDSVEAGIQLIADGLENKAVLGGRETLFFNIQQYGSKSFQLSHKLYTRYSAVAVQIGCPFLDSLNDVLIHLFEGGILDKMTTAEYETQSRMIGKDLNKKTKKTFKEDPSELATKPEEPSTSNEASGKSADNAEMKKPQDTQIIQPLNLRMLQGAFIVLIVGYTMAGLMLLLELFCHGMNLNIIRVCKTNLHKKLKFLKRKLRKSLRWLAVRFK
